# Pd-catalyzed regio- and enantioselective allylation of cyclic allylboronates

**DOI:** 10.1039/d6sc04784j

**Published:** 2026-06-24

**Authors:** Cheng Zhang, Roman Popov, Baptiste Leforestier, Céline Besnard, Clément Mazet

**Affiliations:** a Department of Organic Chemistry, University of Geneva 30 Quai Ernest Ansermet Geneva 1211 Switzerland clement.mazet@unige.ch; b Laboratory of Crystallography, University of Geneva 24 Quai Ernest Ansermet Geneva 1211 Switzerland

## Abstract

A robust protocol for the palladium-catalyzed regio- and enantioselective allylic substitution of 6-membered cyclic boronate salts is reported, enabling efficient access to complex scaffolds possessing two distinct alkenes, a synthetic boron handle, and an allylic tertiary stereocenter. This methodology is characterized by high levels of stereoselectivity and regioselectivity across a wide range of substrates. The synthetic potential of this approach has been demonstrated by several transition metal-catalyzed selective derivatizations. Computational studies at a mixed UMA/DFT level of theory reveal that the reaction is governed by a Curtin–Hammett scenario, wherein reductive elimination from hetero-bis-σ-allyl palladium(ii) intermediates constitutes the regio- and enantio-determining step of the process.

## Abstract 2

A robust protocol for the palladium-catalyzed regio- and enantioselective allylic substitution of 6-membered cyclic boronate salts is reported.

## Graphical abstract

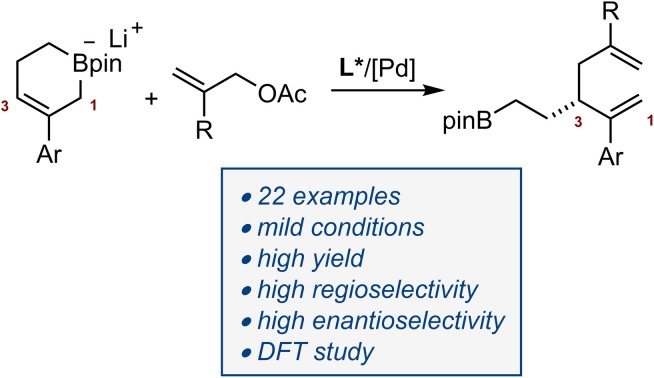

## Introduction

**Fig. 1:**
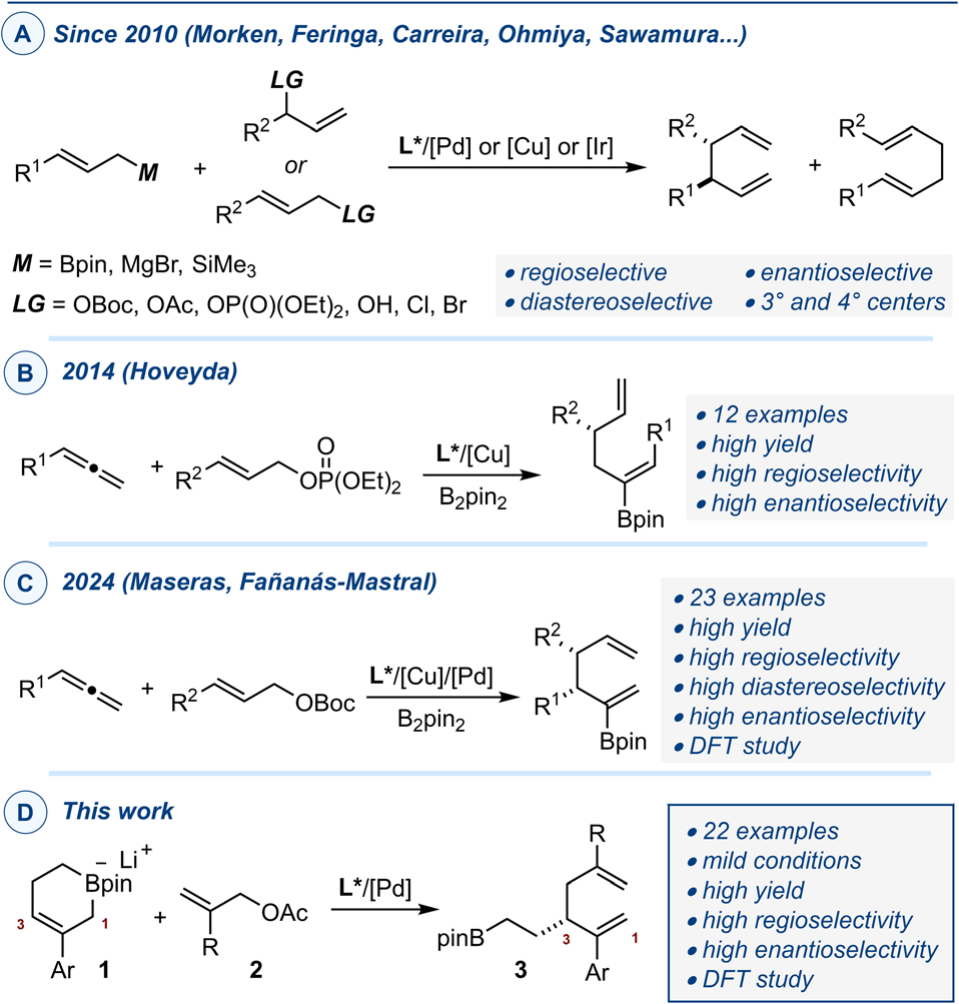
(A) Pd-, Cu- and Ir-catalyzed regio-, diastereo- and enantioselective allyl–allyl cross-coupling reactions. (B) Cu-catalyzed borylative allylation of allenes. (C) Synergistic [Pd/Cu]-catalyzed borylative allylation of allenes. (D) Pd-catalyzed allylation of 6-membered allylboronates.

Chiral 1,5-diene segments are not only highly prevalent in natural products (*e.g.* terpenes) and bioactive compounds, but they also frequently serve as versatile building blocks in organic synthesis.^1^ The catalytic enantioselective allyl–allyl cross-coupling between prochiral allyl metal reagents and allyl electrophiles offers a direct route to chiral 1,5-dienes.^2–8^ In a series of landmark contributions, the Morken group established that allylboronates and allyl carbonates or allyl chlorides could be cross-coupled with excellent levels of regio-, diastereo- and enantioselectivity using Pd catalysis.^2^ Chiral 1,5-dienes featuring either one tertiary stereocenter, vicinal tertiary stereocenters or combinations of adjacent tertiary and quaternary stereocenters were accessible by this approach. Subsequently, Feringa and coworkers disclosed a complementary Cu-catalyzed highly regio- and enantioselective cross-coupling between allyl Grignard reagents and linear allyl bromides.^3^ Ohmiya and Sawamura showed that Cu catalysis was also effective for the enantioselective cross-coupling of allyl boronates and linear (*Z*)-allyl phosphates. Noticeably, the use of a chiral NHC ligand equipped with a phenoxy group proved indispensable to favor the formation of branched 1,5-dienes over their linear isomers.^4^ Carreira reported a chiral iridium–(P,olefin) phosphoramidite complex for the cross-coupling between racemic branched allylic alcohols and allylsilanes to furnish 1,5-dienes with excellent selectivities (Fig. 1A).^5^ More recently, Zhang *et al.* described an asymmetric reductive C(sp^3^)–C(sp^3^) homocoupling of racemic branched allyl acetates operating through cooperative palladium and photoredox catalysis to afford a broad array of *C*_2_-symmetric 1,5-dienes with vicinal tertiary stereocenters.^6^ Even more attractive from a synthetic standpoint are chiral 1,5-dienes which feature additional functional groups that can be further elaborated. Along these lines, the Hoveyda group reported a (NHC)Cu-catalyzed multicomponent protocol combining mono-substituted allenes, a diboron reagent (B_2_pin_2_) and primary allyl phosphates. In this system, the allyl-copper generated by borylcupration of the allene reacts *via* a S_N_2′ mechanism with the allyl electrophile. The resulting enantioenriched 1,5-dienes display a tertiary stereocenter and a trisubstituted (*Z*)-configured alkenylboron unit (Fig. 1B).^7^ A remarkable switch in selectivity was achieved through synergistic Cu/Pd catalysis by Maseras, Fañanás-Mastral and colleagues in a related borylative allyl–allyl cross-coupling (Fig. 1C).^8^ In this process, two adjacent tertiary stereocenters and a vinylborane are installed in one operation with excellent stereoselectivity by means of a single chiral bisphosphine ligand. The success of this approach stems from an inner-sphere S_E_2′ transmetallation from a Cu-allyl intermediate to a Pd-allyl intermediate.

We recently reported a Cu-catalyzed borylation of unactivated vinylcyclopropanes to form non-symmetrical 6-membered cyclic boronate salts (1) and explored their reactivity toward a number of electrophilic reagents.^9^ In a subsequent study, we developed Pd-catalyzed regiodivergent C1- and C3-arylations that afford synthetically modular alkenyl boronates. Computational studies served to establish that C3-arylation is governed mostly by steric factors during binding of the substrate to the metal center, whereas a Curtin–Hammett equilibrium between two *η*^2^-alkene-Pd intermediates determines C1 selectivity. Even though the level of enantioselectivity remained modest, we further demonstrated that the C3-selective arylation was amenable to asymmetric catalysis.^10^ Prior to our work, the Fujihara group had described the preparation of isolable, well-defined, 5-membered cyclic allylboronates by Cu catalysis starting from 1,3-dienes and B_2_pin_2_.^11^ Of note, in a recent communication, they reported the (non-catalytic) allylation of these compounds using allyl bromides to produce a small collection of racemic boron-containing 1,5-dienes.^12^

In this manuscript, we report a protocol for the Pd-catalyzed regio- and enantioselective allylic substitution of 6-membered cyclic boronate salts (1) at C3 to deliver products featuring two distinct alkenes, an allylic tertiary stereocenter and a synthetically modular boron handle (3) (Fig. 1D). The process provides high levels of regio- and enantioselectivity across a broad range of substrates combinations. The synthetic utility of the products is highlighted through a series of transition metal-catalyzed selective derivatizations. Mechanistic investigations based on a computational study point to a system under Curtin–Hammett control where discrimination between competing reductive eliminations from hetero-bis-*s*-allyl palladium(ii) intermediates is responsible for the high levels of regio- and enantioselectivity obtained.

## Results and discussion

### Reaction development

Preliminary investigations consisted in evaluating a representative selection of chiral ligands using cyclic boronate salt 1a and allyl acetate 2a as model substrates (Table 1). While Binap (L1) delivered the targeted product quantitatively but in nearly racemic form, BenzP* (L2) gave 3a in much reduced NMR yield but with a promising level of enantioinduction (entries 1–2). The latter experiment showed substantial formation of alkenylboronate 4a, which results from a formal protonation of 1a. No reaction was observed with L3, an archetypical chiral ligand successfully employed in countless asymmetric allylic alkylation reactions (entry 3).^13^ In contrast, dienylboronate 5a was the only detectable product with bisoxazoline L4 (entry 4). This side-product was also detected using Quinap (L5) together with the desired allylation product 3a (77 : 23 er_3a_; entry 5). The most promising results were subsequently obtained with other (P,N) ligands (L6–L11), with derivatives of the phosphinooxazoline family offering the best balance between reactivity and selectivity (entries 6–11). With the best performer (L11), the level of enantioinduction could be further improved by reducing the temperature and extending the reaction time (entries 12–13). Thus, performing the allylation reaction at −20 °C for 48 h afforded 3a in 70% NMR yield and 96 : 4 er. Of important note, compound 6a that would result formally from a C1-selective allylation reaction was never observed during this optimization campaign.^10^ Finally, other allyl electrophiles did not provide improved performances and focus was therefore placed on the use of readily available allyl acetates (See SI).

**Table 1:** Reaction optimization^a^

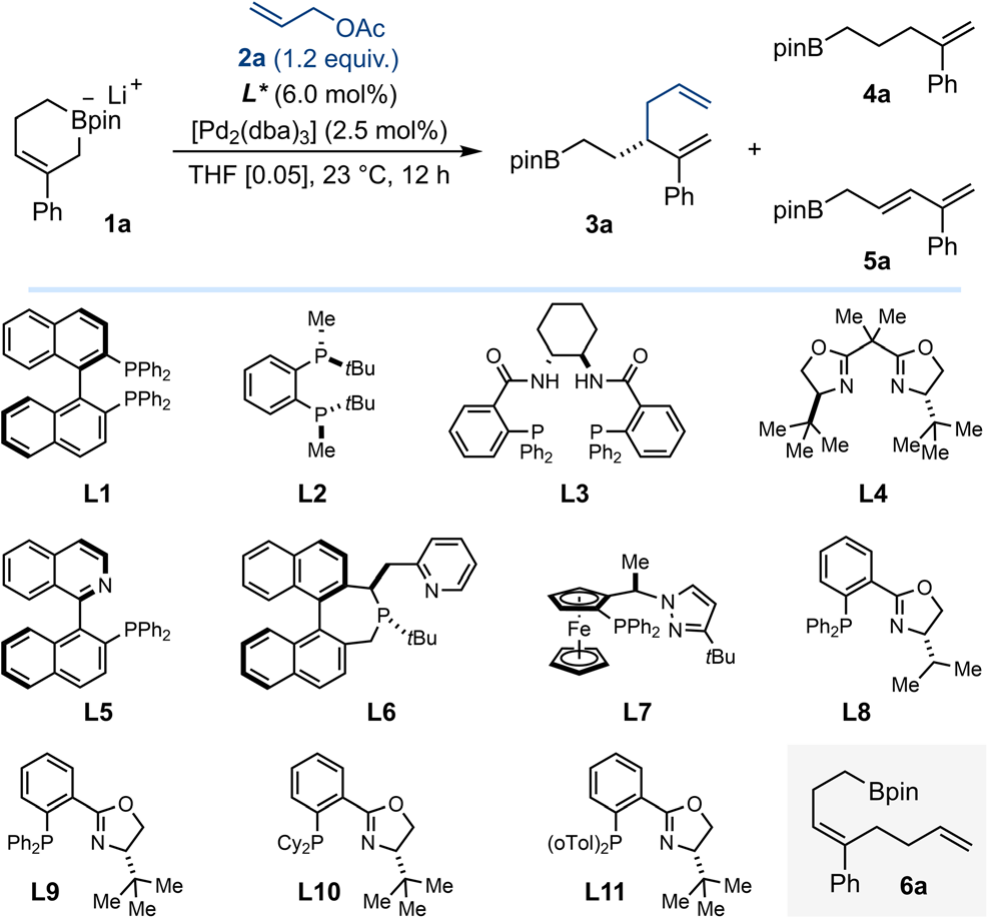					
Entry	L*	3a (%)^b^	4a (%)^b^	5a (%)^b^	er_3a_^c^
1	L1	93	—	—	53 : 47
2	L2	18	38	—	82 : 18
3	L3	<5	—	38	nd
4	L4	—	—	—	nd
5	L5	47	—	—	77 : 23
6	L6	54	—	—	82.5 : 17.5
7	L7	42	—	—	60 : 40
8	L8	86	—	—	87.5 : 12.5
9	L9	75	—	—	92.5 : 7.5
10	L10	19	—	—	75.5 : 24.5
11	L11	85	—	—	94 : 6
12^d^	L11	80	—	—	95 : 5
13^e^	L11	70	—	—	96 : 4

a Reaction conditions: 1a (0.1 mmol).

b Determined by ^1^H NMR analysis of the crude reaction mixture using an internal standard.

c Determined by HPLC using a chiral stationary phase after oxidation to the corresponding alcohol.

d At 0 °C for 12 h.

e At −20 °C for 48 h.

**Fig. 2:**
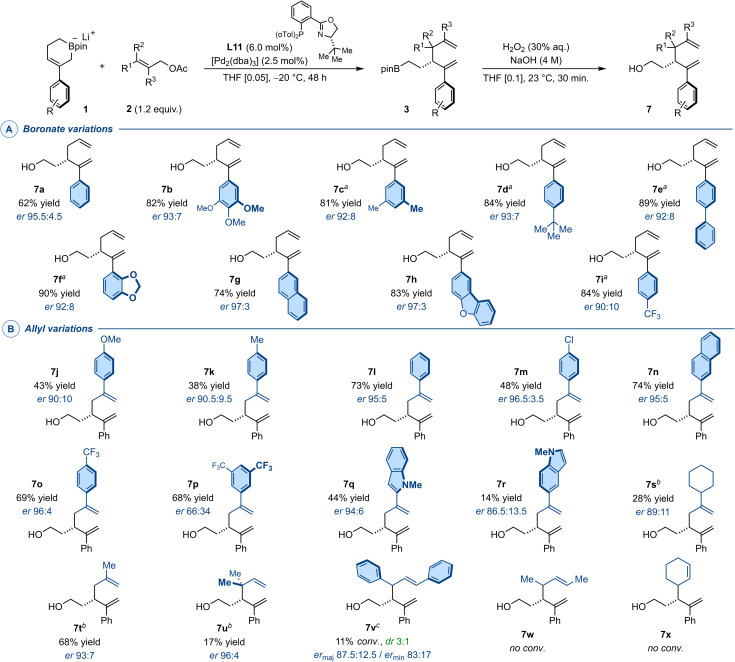
Scope of the Pd-catalyzed C3-selective allylation of cyclic allylboronates (0.3 mmol scale). Regioselectivity >25 : 1 in all cases as determined by ^1^H NMR spectroscopy after allylation using an internal standard. Yield over two steps after oxidation to the corresponding alcohol and purification by column chromatography. ^*a*^ THF/DMF (20 : 1), 23 °C, 24 h. ^*b*^At 23 °C. ^*c*^The relative stereochemistry for 7v could not be determined unambiguously.

The generality of the Pd-catalyzed allylation reaction was explored using these optimal reaction conditions. As observed with the model reaction, C3/C1 regioselectivity was systematically >25 : 1. Variations of the structure of the cyclic boronate salts (1) were first carried out (Fig. 2A). For derivatives that were only sparingly soluble in THF, we ran the reaction in a 20 : 1 THF/DMF solvent mixture at 23 °C for 24 h. For the sake of practicality, all products were isolated after nearly quantitative alkali oxidation to the corresponding alcohols (7). We observed that substrates with an electron-neutral or an electron-rich aryl group underwent catalysis efficiently, delivering the products in high yield and excellent levels of enantiocontrol (7a–7h). A slight decrease in enantioselectivity was observed when a *p*-trifluoromethyl group was introduced (7i: 90 : 10 er). Structural variations of the allyl acetate precursor (2) led us to identify 2-substituted derivatives as the most suitable candidates with the optimized reaction conditions (Fig. 2B). Whereas electron-rich electrophilic substrates led to moderate yield and slightly reduced enantiomeric ratio (7j–k), electron-neutral and electron-deficient precursors afforded the allylation product in systematically high yield and in enantiomeric ratio ≥95 : 5 (7l–7o). The level of enantioinduction dropped markedly when two trifluoromethyl substituents were introduced in *meta* positions of the aryl substituent (7p). Connecting an *N*-methyl indole by the 2-position to the allyl unit led to appreciable performances (7q), while an isomeric structure connected by the 5-position showed poor reactivity and lower level of enantioinduction (7r). 2-Alkyl substituted allyl acetates were also found to be competent substrates with the optimized reaction conditions. While 2-methylallyl acetate generated 7t in 68% yield and 93 : 7 er, 2-cyclohexylallyl acetate afforded 7s in 89 : 11 er but in only 28% yield, presumably due to more pronounced steric hindrance. Even though the yield of the product was very low, using isoprenyl acetate, we established that the catalytic method enables the installation of a quaternary center adjacent to the tertiary stereocenter whilst maintaining a high level of enantiocontrol (7u: 96 : 4 er). Allyl derivatives with identical substituents at C1 and C3 are an important substrate subclass for enantioselective allylic substitutions.^13*d*^ Using racemic 1,3-diphenylprop-2-enyl acetate, the catalytic activity was rather low and 7v was generated as a 3 : 1 diastereomeric mixture, each isomer being obtained with relatively similar levels of enantiocontrol (7v: 87.5 : 12.5 er_maj_/83 : 17 er_min_). No reaction was observed starting from racemic 1,3-dimethylprop-2-enyl acetate or from racemic cyclohexyl acetate. The absolute configuration of the allylation products was established by analogy after X-ray analysis of a derivative of 7e (See SI).

**Fig. 3:**
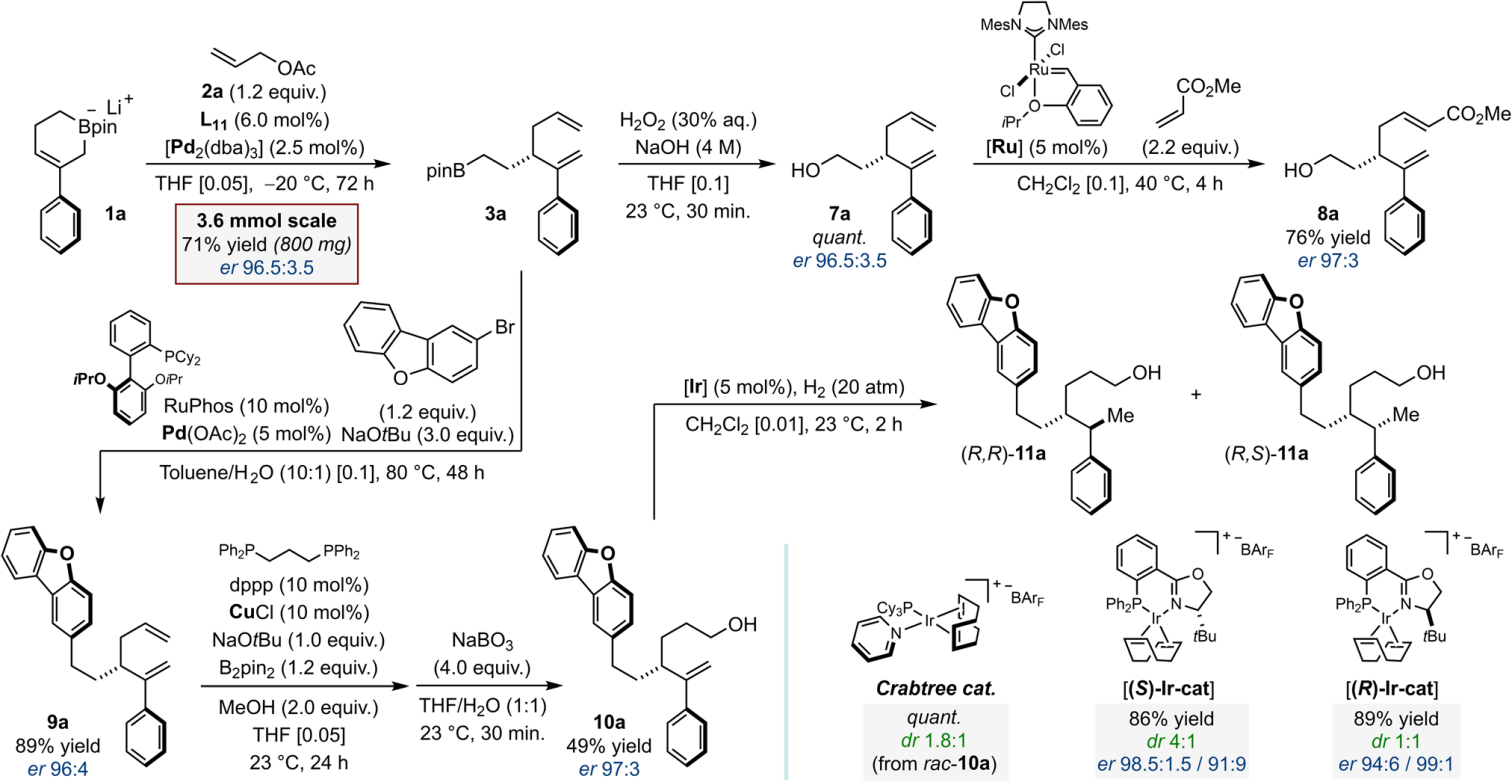
Gram-scale experiment for the Pd-catalyzed C3-selective allylation of cyclic allylboronates 1a (3.6 mmol scale) and selective transition metal-catalyzed derivatizations of 3a (0.05–0.30 mmol scale).

The robustness of the protocol was established by conducting the Pd-catalyzed enantioselective allylation of cyclic allylboronates on a 3.6 mmol scale using 1a and allyl acetate 2a to afford the product (3a) as a yellow oil without any erosion of the reactivity or the enantioselectivity (71% yield, 96.5 : 3.5 er_3a_) (Fig. 3). Next, we sought to demonstrate the synthetic utility of the product of catalysis through a series of selective transition metal-catalyzed derivatizations. After quantitative alkali oxidation of 3a to 7a, we showed that methyl acrylate reacts preferentially with the terminal alkene when engaged in a subsequent Ru-catalyzed cross-metathesis using Hoveyda–Grubbs precatalyst to generate 8a (76% yield, 97 : 3 er_8a_). In parallel, 9a was obtained in 89% yield (96 : 4 er_9a_) when 3a was engaged in a Pd-catalyzed cross-coupling reaction using 2-bromodibenzofuran as electrophile.^14^ Subsequently, a highly chemo- and regioselective Cu-catalyzed protoboration of the terminal alkene furnished 10a after oxidation of the resulting boronate to the corresponding primary alcohol (49% over two steps, 97 : 3 er_10a_). The diastereoselective hydrogenation of 1,1-disubstituted alkenes is challenging, in particular for substrates with a potentially labile stereocenter in allylic position such as 10a.^15^ Using Pfaltz's modified version of the Crabtree catalyst, 11a was generated quantitatively. The 1.8 : 1 diastereomeric mixture obtained provides a measure of the bias imposed by the stereocenter present in the substrate. With the (*S*) enantiomer of a commonly employed iridium-based hydrogenation precatalyst ([(*S*)-Ir-cat]),^16^ the diastereomeric ratio increased to 4 : 1 (match case), while with the catalyst of opposite absolute configuration, the dr was only 1 : 1 (mismatch). The variations of the enantiomeric ratio with respect to 10a measured in both cases point to a complex mechanistic scenario in which concurrent isomerization transiently generating the (*E*) and (*Z*) stereoisomers of a tetrasubstituted alkene is likely to occur.^18^

### Computational study

Mechanistic investigations were performed computationally. Building on the methodology established in our previous study on the Pd-catalyzed arylation of the cyclic boronate salts,^10^ the speciation of 1a in THF with respect to aggregation and microsolvation was examined by molecular dynamics simulations. Thus, 1a was placed in a spherical cell containing 25 THF molecules bounded by repulsive harmonic walls, with the sphere radius adjusted to match the experimental density of THF (see SI, section 8.1), and left to equilibrate over the course of 50 ps at 300 K. The lowest free energy configuration for 1a was determined to feature two explicit molecules of THF coordinated to the lithium atom, the latter being chelated by the substrate *via* one oxygen atom of the (pinacolato)boron unit and the alkene. A dimeric form of 1a featuring bridging lithium cations was similarly placed in a spherical cell containing 29 THF molecules and left to equilibrate for 50 ps. Spontaneous dissociation into two microsolvated monomers was observed rapidly, further suggesting the monomeric nature of 1a in THF. Anticipating the potential dissociation of LiOAc over the course of the reaction, the microsolvation of LiOAc was also investigated and led to the identification of LiOAc(THF)_3_ as the most favorable species.^19^

**Fig. 4:**
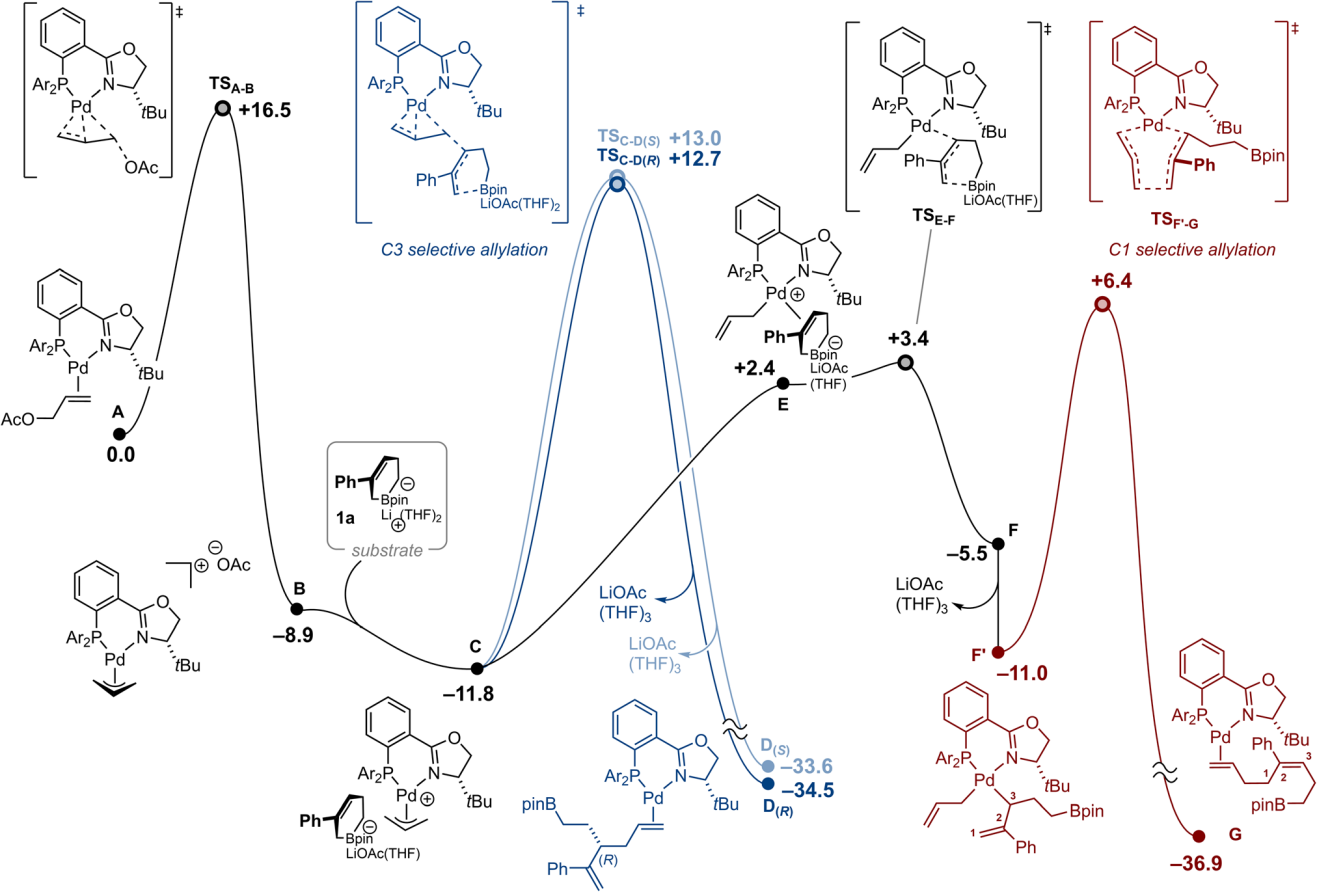
Computed free energy reaction profile (kcal mol^−1^, 253.15 K) for the Pd-catalyzed C3-selective allylation of cyclic allylboronate 1a*via* an outer sphere mechanism (blue) and alternative pathway *via* a hetero-bis-σ-allyl intermediate. Level of theory: (ωB97M-V/def2-TZVPD//UMA-*s*-1p1)ALPB(THF), ORCA 6.1.0.^17^

While the use of UMA for full studies on catalytic cycles is only emerging in the literature,^10,20^ our benchmark calculations on selected geometries showed excellent agreement with the reference DFT level of theory as well as DLPNO-CCSD(T) (see SI). Our initial mechanistic hypothesis was inspired by a scenario recently proposed by Kan, Li and Wu, whereby an oxidative addition leads to a cationic Pd-allyl intermediate that subsequently undergoes a direct outer-sphere nucleophilic attack by the alkene of the boronate salt.^21^ The corresponding computed profile is displayed in Fig. 4 (A→D) and begins with oxidative addition of allyl acetate to the phosphinooxazoline palladium(0) complex A (TS_A-B_; Δ*G*^‡^ = 16.5 kcal mol^−1^) to deliver the corresponding cationic π-allyl Pd(ii) intermediate B at −8.9 kcal mol^−1^. The magnitude of the corresponding activation energy is consistent with that calculated for similar systems by Bellido *et. al.*^22^ Subsequent association of the substrate 1a is slightly favored, with the catalyst-substrate adduct C located at −11.8 kcal mol^−1^. From C, an outer-sphere nucleophilic addition of the C=C bond of the boronate to the electrophilic π-allyl-Pd complex irreversibly provides the very exergonic product-bound Pd(0) intermediates D_(_*_R_*_)_ and D_(_*_S_*_)_. The corresponding diastereomeric transition states were calculated at TS_C-D(_*_R_*_)_ at +12.7 and TS_C-D(_*_S_*_)_ at +13.0 kcal mol^−1^ respectively (ΔΔ*G*^‡^ = 0.3 kcal mol^−1^) (Fig. 4, blue pathways).^23^ Although these results are in line with the stereochemical and regiochemical outcomes of the reaction observed experimentally, the corresponding overall barriers from C (24.5–24.8 kcal mol^−1^) are not consistent with a reaction occurring at −20 °C and underline the need to identify a kinetically more accessible manifold. We first assumed that dissociation of the LiOAc salt from the substrate would enhance the nucleophilicity of the cyclic allylboronate, but this approach only yielded transition states higher in energy (see SI, section 8.3.3).

In our previous study on the Pd-catalyzed C3-selective arylation of cyclic boronate salts,^10^ two distinct activation modes were identified, proceeding *via* (i) direct ring-opening electrophilic substitution at the Pd center or (ii) coordination of the alkene and followed by ring-opening. While electrophilic substitution did not yield a more favorable pathway (see SI, section 8.3.3), *η*^2^-coordination of 1a*trans* to the P atom of the ancillary ligand accompanied by concomitant π → σ isomerization of the allyl fragment is accessible, albeit uphill (intermediate E, +2.4 kcal mol^−1^). The ensuing ring-opening is near barrierless, with TS_E-F_ located at only +3.4 kcal mol^−1^, affording the bis-σ-allyl Pd(ii) complex F at −5.5 kcal mol^−1^. Further stabilization by dissociation of LiOAc away from the overall neutral Pd(ii) fragment generates F′ at −11.0 kcal mol^−1^. Similar bis-σ-allyl Pd(ii) complexes have been described in the literature by Echavarren,^24^ Álvarez and Espinet,^25^ Morken,^2*a*,*e*^ and more recently by Maseras and Fañanás-Mastral.^8^ In particular, they have been shown to favor 3–3′ reductive elimination over the canonical 1–1′ pathway. This preferred reactivity pattern was verified with our system, with the reductive elimination transition state TS_F′-G_ located at +6.4 kcal mol^−1^ being the most accessible to afford G irreversibly (see SI, section 8.3.6). While this pathway is favored by more than 6 kcal mol^−1^ over the outer sphere mechanism described above (A→D), it unfortunately diverts the system towards the formation of a C1-allylation regioisomer that was never observed experimentally (Fig. 4, crimson-red pathway).

**Fig. 5:**
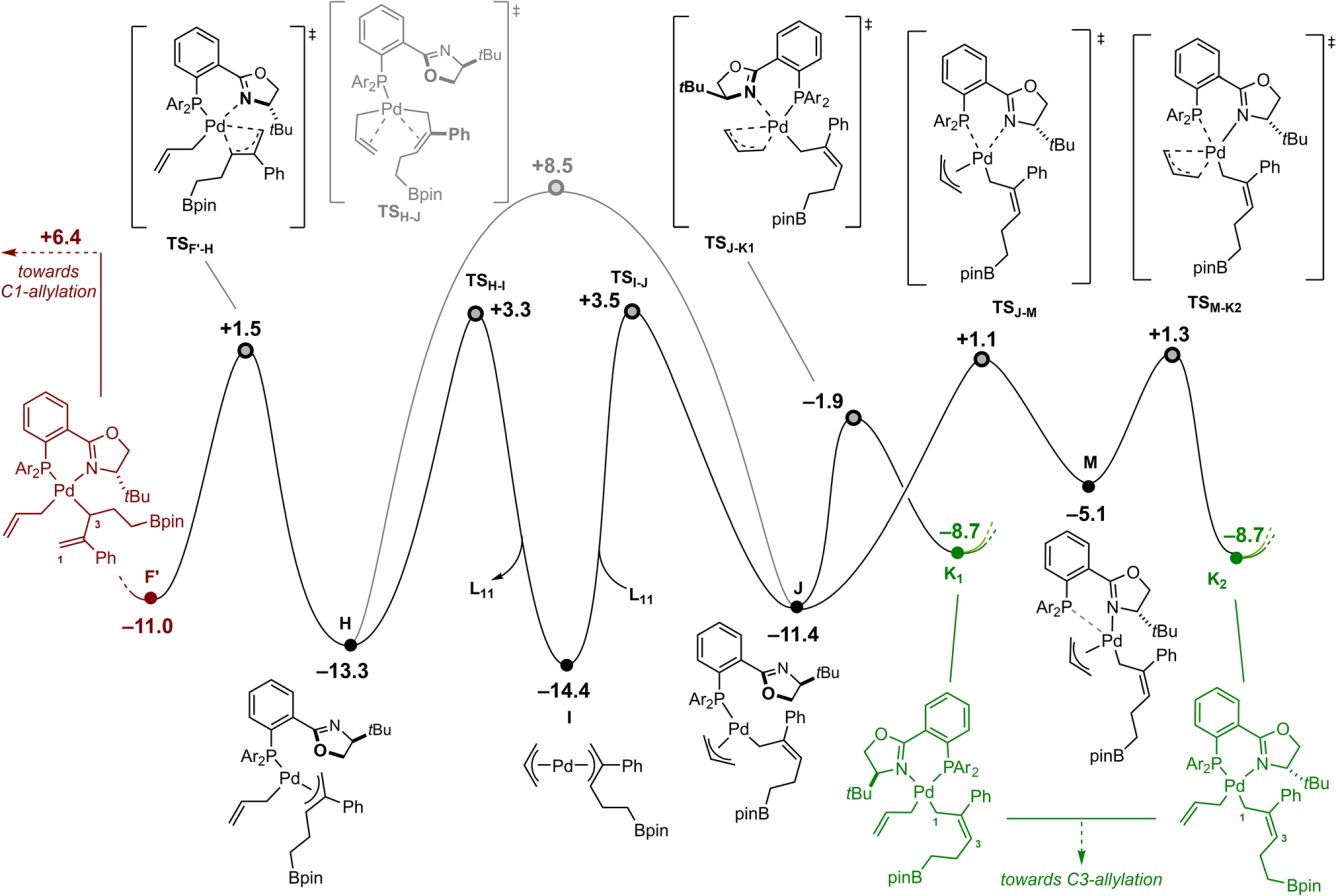
Computed free energy reaction profile (kcal mol^−1^, 253.15 K) for the reversible isomerization of pro-C1 hetero-bis-σ-allyl complex F′ into pro-C3 hetero-bis-σ-allyl complexes K_1_ and K_2_. Level of theory: (ωB97M-V/def2-TZVPD//UMA-*s*-1p1)ALPB(THF), ORCA 6.1.0.^17^

**Fig. 6:**
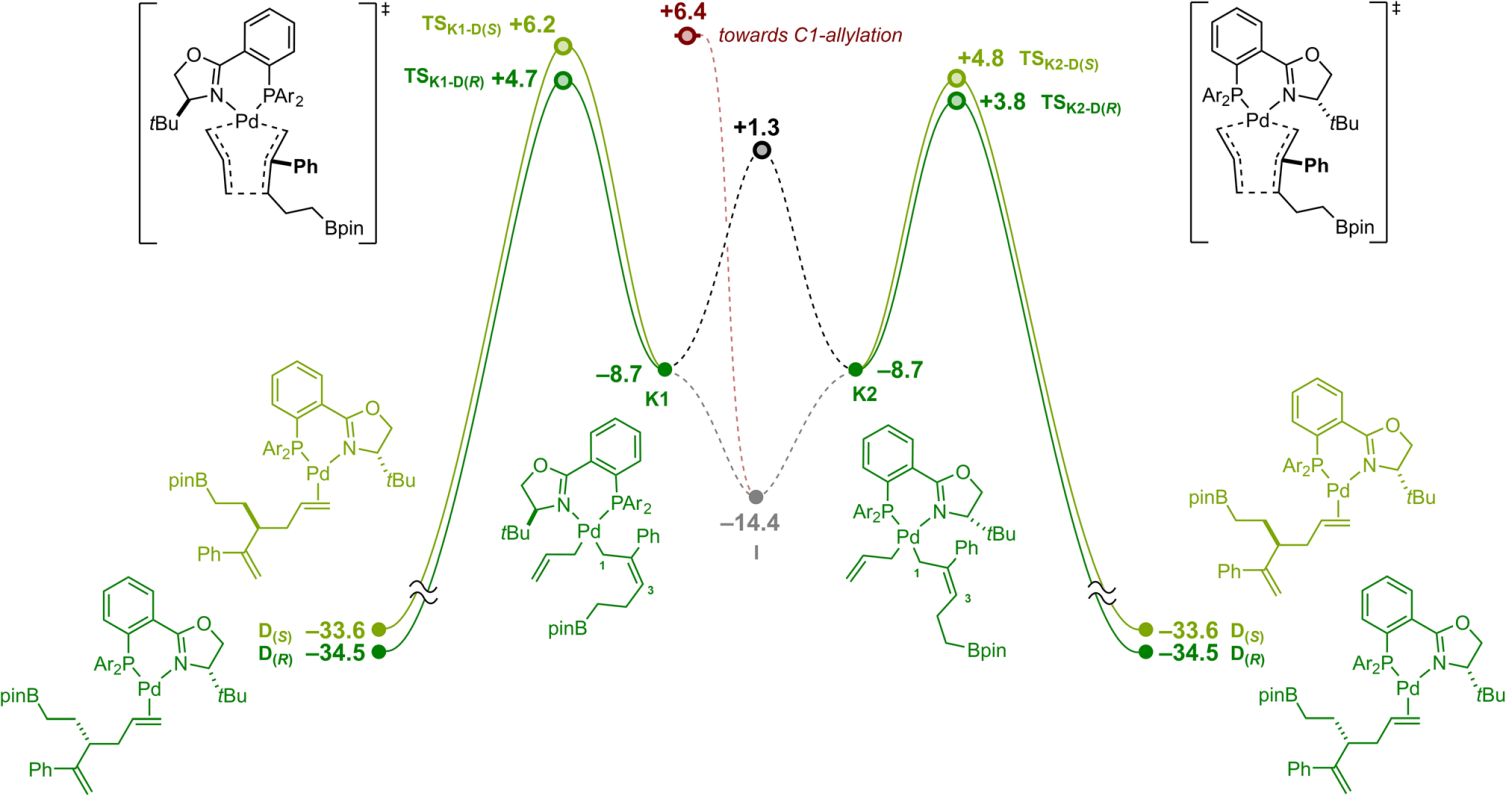
Summarized free energy profile (kcal mol^−1^, 253.15 K) for the Curtin–Hammett control between K_1_ and K_2_ and corresponding pro-(*R*) and pro-(*S*) reductive elimination transition states affording Pd-bound C3-allylation products D_(_*_R_*_)_ and D_(_*_S_*_)_, respectively. Level of theory: (ωB97M-V/def2-TZVPD//UMA-*s*-1p1)ALPB(THF), ORCA 6.1.0.^17^

At this stage, we reasoned that an isomeric structure of F′ where the two allyl fragments would be well-poised for a subsequent C3-selective reductive elimination might be accessible through successive σ/π-allyl isomerizations of both allyl units. Such a pathway could be identified and is displayed in Fig. 5. We found that σ-to-π isomerization of the substrate-derived allyl fragment was accompanied by decoordination of the N-donor of the oxazoline ring to access the slightly more stable intermediate H at −13.3 kcal mol^−1^*via* a modest barrier (TSF′-H at +1.5 kcal mol^−1^). From H, a direct intramolecular σ/π exchange in which the substrate-derived allyl returns to σ-bonding (retaining C1–Pd connectivity) concomitant with σ–π conversion of the second allyl to give J was found to occur *via*TS_H-J_ at +8.5 kcal mol^−1^ (grey pathway). Although mechanistically reasonable, this barrier is kinetically disfavored relative to the competing F′→G pathway leading to the C1-regioisomer (TS_F′-G_ at +6.4 kcal mol^−1^) (Fig. 5). Instead, perhaps counterintuitively, the lowest accessible manifold we could identify from H proceeds by σ-to-π isomerization of the second allyl fragment, which is coupled with decoordination of ligand L11 to furnish the hetero-bis(*η*^3^-allyl) palladium(ii) intermediate I (Δ*G* = −14.4 kcal mol^−1^) *via*TS_H-I_ at only +3.3 kcal mol^−1^. Coordination of the P atom of L11 from the opposite side enables formation of a σ-C1-allyl complex while maintaining π-coordination of the simple allyl unit, to afford J located at −11.4 kcal mol^−1^*via*TS_I-J_ at +3.5. Subsequent chelation by both donor atoms of the phosphinooxazoline ligand gives K_1_ (at −8.7 kcal mol^−1^) through TS_J-K1_ (−1.9 kcal mol^−1^). Equilibration of K_1_ to K_2_ (also at −8.7 kcal mol^−1^) occurs readily *via* a shallow interconversion surface that passes through intermediate M at −5.1 kcal mol^−1^ and low-lying transition structures at +1.1 and +1.3 kcal mol^−1^. Importantly, the isomeric structures of K_1_ and K_2_ both place the allyl termini in the arrangement required to access the C3-allylation product *via* a reductive elimination involving the remote carbon atoms of the σ-allyl fragments. Collectively, the above-described isomerization events enable complete interconversion between F′, K_1_, and K_2_, placing the system under Curtin–Hammett control. As a corollary, neither regio- nor enantioselectivity are determined by the relative populations of these intermediates, but rather by the free-energy differences between the competing reductive-elimination transition states accessible from each path. This relationship is summarized in Fig. 6, which shows that the C3-selective reductive eliminations originating from K_1_ and K_2_ are uniformly lower in energy than the C1-selective pathway from F′. Moreover, in agreement with experimental observations, for both K_1_ and K_2_, the pro-(*R*) transition state is consistently favored over the corresponding pro-(*S*) transition state. From D_(_*_R_*_)_, release of the product and coordination of another equivalent of allyl acetate was calculated to be downhill by 4.8 kcal mol^−1^ (See SI, Section 8.3.7). Therefore, the overall catalytic cycle is predicted to proceed with an apparent free-energy barrier of 18.2 kcal mol^−1^, defined by the energy span between I and TS_K2-D(_*_R_*_)_, a magnitude fully compatible with the optimized reaction conditions.^26,27^ Of note, within the intrinsic uncertainty of DFT, this turnover-limiting event may be kinetically competitive with the initial oxidative addition of allyl acetate.

## Conclusions

In conclusion, we have developed a protocol for the palladium-catalyzed regio- and enantioselective allylic substitution of 6-membered cyclic boronate salts. This catalytic reaction provides efficient access to valuable scaffolds possessing two distinct alkenes, a synthetic boron handle, and an allylic tertiary stereocenter. This methodology features high levels of regio- and enantioselectivity across an array of structurally diversified substrates, and its synthetic utility was illustrated through various transition metal-catalyzed derivatizations. Computational studies at a mixed UMA/DFT level of theory indicate that the reaction is governed by a Curtin–Hammett scenario, where high stereo- and regioselectivity are achieved through controlled reductive elimination from hetero-bis-σ-allyl palladium(ii) intermediates.

## Author contributions

The manuscript was written through the contributions of all authors, and all authors have given approval to the final version.

## Conflicts of interest

There are no conflicts to declare.

## Supplementary Material

SC-017-D6SC04784J-s001

SC-017-D6SC04784J-s002

SC-017-D6SC04784J-s003

## Data Availability

CCDC 2532060 (7e) contains the supplementary crystallographic data for this paper.^28^ The data supporting this article have been included as part of the supplementary information (SI). Supplementary information: experimental procedures, characterization of all new compounds, spectroscopic data X-ray crystallographic data and molecular coordinates of computed structures (*xyz*). See DOI: https://doi.org/10.1039/d6sc04784j.
