# Supplementary material for: Pd-catalyzed regio- and enantioselective allylation of cyclic allylboronates
Source: Chem Sci. 2026 Jun 24. Online ahead of print. doi: 10.1039/d6sc04784j (PMC13325626; doi:10.1039/d6sc04784j)
Supplement: SC-OLF-D6SC04784J-s002 [file SC-OLF-D6SC04784J-s002.pdf]

Supplementary Information (SI) for Chemical Science.
This journal is © The Royal Society of Chemistry 2026

Pd-catalyzed regio- and enantioselective allylation of cyclic allylboronates

Cheng Zhang,[a] Roman Popov,[a] Baptiste Leforestier,[a] Céline Besnard[b] and Clément Mazet*[a]

[a] Department of Organic Chemistry, University of Geneva, 30 quai Ernest Ansermet, 1211 Geneva, Switzerland.

[b] Laboratory of Crystallography, University of Geneva, 24 quai Ernest Ansermet, 1211 Geneva, Switzerland.

clement.mazet@unige.ch

Contents

1. **Substrates synthesis** **4**

1.1 Synthesis of 2-(1-methyl-1*H*-indol-5-yl)prop-2-en-1-ol 4

1.2 Synthesis of allyl acetates 5

1.3 Synthesis of allylboronates 7

2. **General Procedure for reaction optimization** **8**

3. **Pd-catalyzed enantioselective allylation reactions** **10**

3.1 General procedure I (GP-I) 10

3.2 General procedure II (GP-II) 11

4. **Gram scale experiment** **54**

5. **Selective transition metal-catalyzed derivatizations of 3a** **57**

5.1 Pd-catalyzed cross-coupling reaction 57

5.2 Cu-catalyzed protoboration reaction 60

5.3 Ir-catalyzed hydrogenation reaction 63

5.4 Ru-catalyzed cross metathesis reaction 66

5.5 Esterification of **7e** 69

6. **X-ray analysis** **71**

7. **Computational studies** **74**

7.1 Substrate speciation 75

7.2 Energetics benchmark – Outer sphere path 76

7.3 Pd-catalyzed C3-allylation pathway 78

7.3.1 Main mechanism – outer sphere path and C1-allylation 78

7.3.2 Isomerism of intermediate B 79

7.3.3 Alternative activation of **1a** 80

7.3.4 Main mechanism – inner sphere path and isomerization 81

7.3.5 Main mechanism – pro-C3, 3-3’ reductive elimination TS 82

7.3.6 Alternative reductive elimination transition states 83

7.3.7 Closing the catalytic cycle 84

7.4 Monitored deviation between UMA-s-1p1 and DFT 85

8. **References** **86**

9. **NMR spectra** **88**

General information

Unless otherwise noted, all reactions were carried out under an inert atmosphere of nitrogen using either a two-manifold vacuum/inert gas lines or a M. Braun glovebox. Solvents were dried over activated alumina columns and further degassed by three successive "freeze-pump-thaw" cycles. Commercial reagents were purchased from ABCR, Fluka, Acros or Strem and used without purification unless otherwise noted. Liquid reagents were transferred with stainless steel syringes or cannula. Thin layer chromatography (TLC) was performed on plates of silica precoated with 0.25 mm Kieselgel 60 F_{254} from Merck. Flash chromatography was performed using silica gel SiliaFlash® P60 (230-400 mesh) from Silicycle.

NMR spectra were acquired at the University of Geneva NMR platform (https://www.unige.ch/sciences/chiorg/nmr/) using a 500 MHz Avance III Bruker NMR spectrometer equipped with a helium-cooled cryogenic 5-mm DCH ^{13}C-^{1}H/D Bruker probe, a 400 MHz Avance III HD NanoBay spectrometer equipped with a N_2 prodigy cryogenic 5 mm CPP BB(F)-H-D probe or a 300 MHz Avance III, HD NanoBay spectrometer, equipped with a 5 mm PA BBO, BB(F)-H-D probe. ^{1}H NMR spectra were referenced to $CDCl_3$ (7.26 ppm) and ^{13}C{^{1}H} NMR spectra were referenced to $CDCl_3$ (77.16 ppm). ^{19}F{^{1}H} NMR chemical shifts are reported in ppm with absolute reference relative to ^{1}H. HRMS data were obtained on a Xevo G2 Tof spectrometer (Ionization mode: ESI positive polarity; Mobile phase: MeOH 100 µl/min). Mass spectrum is calibrated using the MS lockspray system (LeuEnk calibration solution). Infrared spectra were obtained on a Perkin–Elmer 1650 FT-IR spectrometer using neat samples on a diamond ATR Golden Gate sampler. Melting points were recorded on a Buchi SMP-20 melting point apparatus using open glass capillaries.

The enantiomeric ratios (*er*) were determined by HPLC analyses. HPLC analyses were performed on a Shimadzu CTO-20AA equipped with DAICEL OD-H, OZ-H, OJ-H, AD-H and IC columns. Retention times (t_R) are given in minutes. Optical rotations were recorded using an OMNI Lab JASCO P-1030 polarimeter using 589 nm emission band of a sodium lamp.

$Pd_2(dba)_3$ was purified by recrystallization in chloroform and acetone based on a literature procedure.[1] All the phosphinooxazoline ligands were synthesized according to the literature.[2]

1. Substrates synthesis

1.1 Synthesis of 2-(1-methyl-1*H*-indol-5-yl)prop-2-en-1-ol

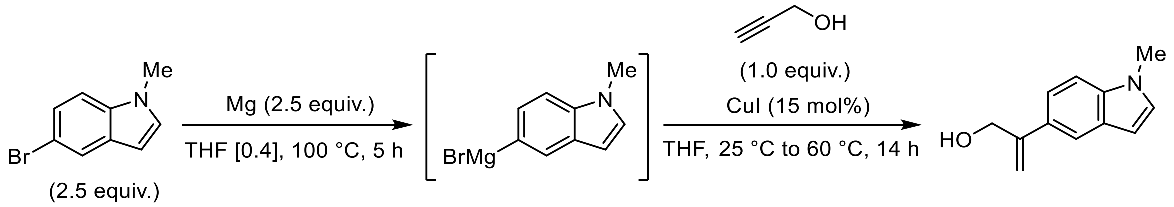

Figure S1. Synthesis of 2-(1-methyl-1*H*-indol-5-yl)prop-2-en-1-ol

To an oven-dried 25 mL Schlenk, Mg turnings (112 mg, 5.0 mmol, 2.5 equiv.) and iodine (1 mg) were suspended in THF (5 mL) at room temperature (25 °C). Next a solution of 5-bromo-1-methylindole (1.05 g, 5.0 mmol, 2.5 equiv.) in THF (2 mL) was added to the reaction mixture over 10 minutes. After addition, the mixture was stirred at 100 °C. After 5 h, the mixture was cooled to 25 °C, and CuI (57 mg, 0.3 mmol, 15 mol%) was added. The mixture was stirred at 25 °C. After 30 minutes, the 2-propyn-1-ol (115 μL, 2.00 mmol, 1.0 equiv.) was added by syringe, and the resulting mixture was stirred at 60 °C. After 14 h, the reaction was cooled to room temperature and quenched with saturated $NH_4Cl_{aq.}$ (1 mL), extracted with Et_2O (3 × 10 mL), washed with brine. After drying over Na_2SO_4 filtration and concentration, the crude mixture was purified by flash chromatography on silica gel using pentane/Et_2O as eluent, to afford the product as a yellow oil in 33% yield. Spectroscopic data were in agreement with those reported in the literature.[3]

1.2 Synthesis of allyl acetates

A series of allyl acetates were prepared according to the literature procedure.[4]

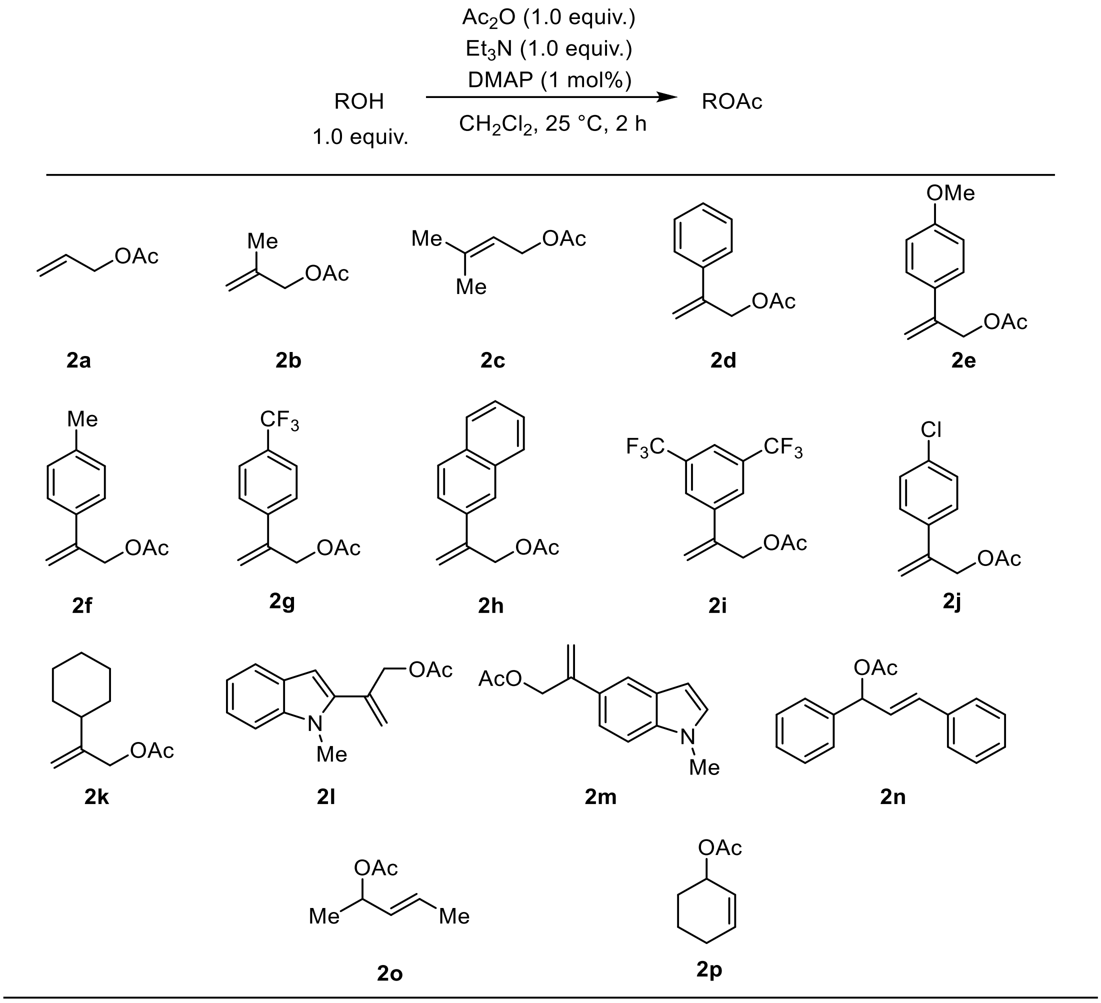

Figure S2. Synthesis of allyl acetates

General procedure:

In an oven dried 10 mL flask, 4-(dimethylamino)pyridine (2.4 mg, 1 mol%) was dissolved in CH_2Cl_2 (4 mL), followed by Et_3N (279 μL, 2 mmol, 1.0 equiv.) and the corresponding allyl alcohol (2 mmol, 1.0 equiv.). Subsequently, acetic anhydride (189 μL, 2.0 mmol, 1.0 equiv.) was added at 25 °C, and the mixture was stirred at the same temperature. After 2 h, the reaction was quenched with aq. HCl solution (2 M, 4 mL), extracted with CH_2Cl_2 (3 × 10 mL), washed with saturated aq. $NaHCO_3$ (10 mL). After drying over Na_2SO_4, filtration and concentration, the crude mixture was purified by flash chromatography on silica gel using pentane/Et_2O as eluent to afford the corresponding allyl acetate. The NMR data of compounds **2a-k** and **2n-p** were matched with those reported.[4]

2-(1-methyl-1H-indol-2-yl)allyl acetate (2l)

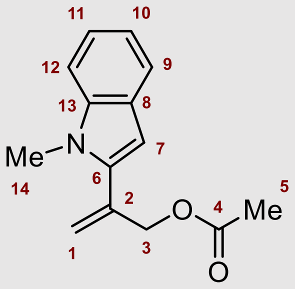

2l

Prepared from 2-(1-methyl-1*H*-indol-2-yl)prop-2-en-1-ol according to the general procedure in **2.2**, affording 2-(1-methyl-1H-indol-2-yl)allyl acetate **2l** as a yellow oil (99% yield, 454 mg,) after chromatography on silica gel (eluent: pentane/Et_2O = 10:1).

TLC: 0.4, pentane/Et_2O = 10:1

^{1}H NMR (400 MHz, $CDCl_3$) δ (ppm) = 7.60 (dt, $^3J_{H\text{-}H}$ = 7.8 Hz, $^4J_{H\text{-}H}$ = 0.9 Hz, 1H, *H9*), 7.33 (dd, $^3J_{H\text{-}H}$ = 8.2 Hz, $^4J_{H\text{-}H}$ = 0.8 Hz, 1H, *H12*), 7.25 (m, 1H, *H11*), 7.12 (m, *H10*), 6.52 (d, $^4J_{H\text{-}H}$ = 0.6 Hz, 1H, *H7*), 5.65 (q, $^4J_{H\text{-}H}$ = 1.4 Hz, 1H, *H1*), 5.39 (d, $^4J_{H\text{-}H}$ = 0.8 Hz, 1H, *H1*), 4.92 (brs, 2H, *H3*), 3.78 (s, 3H, *H14*), 2.10 (s, 3H, *H5*).

^{13}C{^{1}H} NMR (101 MHz, $CDCl_3$) δ (ppm) = 170.7 (*C4*), 138.6 (*C13*), 138.0 (*C6*), 135.3 (*C2*), 127.6 (*C8*), 122.3 (*C11*), 120.9 (*C9*), 120.0 (*C10*), 118.1 (*C1*), 109.7 (*C12*), 101.7 (*C7*), 66.5 (*C3*), 31.4 (*C14*), 21.1 (*C5*).

HRMS (ESI +**)**: calculated for $C_{14}H_{15}NO_2$ $[M+H]^+$: 230.1176; found: 230.1170.

IR (neat) ν (cm^{-1}) = 3053, 2939, 1737, 1464, 1367, 1218, 1038, 909, 787, 749, 734.

2-(1-methyl-1H-indol-5-yl)allyl acetate (2m)

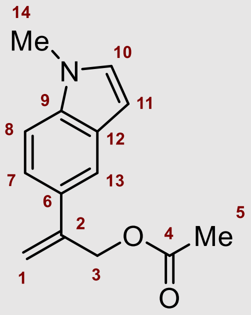

2m

Prepared from 2-(1-methyl-1*H*-indol-5-yl)prop-2-en-1-ol according to the general procedure in **2.2**, affording 2-(1-methyl-1H-indol-5-yl)allyl acetate **2m** as a yellow solid (99% yield, 454 mg) after chromatography on silica gel (eluent: pentane/Et_2O = 8:1).

TLC: 0.4, pentane/Et_2O = 8:1

^{1}H NMR (400 MHz, $CDCl_3$) δ (ppm) = 7.68 (dd, $^4J_{H\text{-}H}$ = 1.7, 0.6 Hz, 1H, *H13*), 7.35 (dd, $^3J_{H\text{-}H}$ = 8.6 Hz, $^4J_{H\text{-}H}$ = 1.7 Hz, 1H, *H7*), 7.29 (dt, $^3J_{H\text{-}H}$ = 8.6 Hz, $^4J_{H\text{-}H}$ = 0.6 Hz, 1H, *H8*), 7.06 (d, $^3J_{H\text{-}H}$ = 3.1, 1H, *H10*), 6.49 (dd, $^3J_{H\text{-}H}$ = 3.1 Hz, $^4J_{H\text{-}H}$ = 0.8 Hz, 1H, *H11*), 5.54 (bs, 1H, *H1*), 5.31 (q, $^4J_{H\text{-}H}$ = 1.3 Hz, 1H, *H1*), 5.06 (d, $^4J_{H\text{-}H}$ = 0.9 Hz, 2H, *H3*), 3.80 (s, 3H, *H14*), 2.08 (s, 3H, *H5*).

$^{13}C\{^1H\}$ NMR (101 MHz, $CDCl_3$) δ (ppm) = 171.1 (*C4*), 143.5 (*C2*), 136.7 (*C9*), 129.6 (*C6*), 129.6 (*C10*), 128.6 (*C12*), 120.2 (*C7*), 118.5 (*C13*), 113.5 (*C1*), 109.3 (*C8*), 101.6 (*C11*), 66.5 (*C3*), 33.0 (*C14*), 21.2 (*C5*).

HRMS (ESI +**)**: calculated for $C_{14}H_{15}NO_2$ $[M+H]^+$: 230.1176; found: 230.1170.

IR (neat) ν (cm^{-1}) = 3104, 2933, 1737, 1628, 1491, 1390, 1240, 1042, 807, 774, 740, 642.

m.p.: 33.8–35.4 °C.

1.3 Synthesis of allylboronates

All the cyclic allylboronates used in this study were prepared according to our previous report.[5]

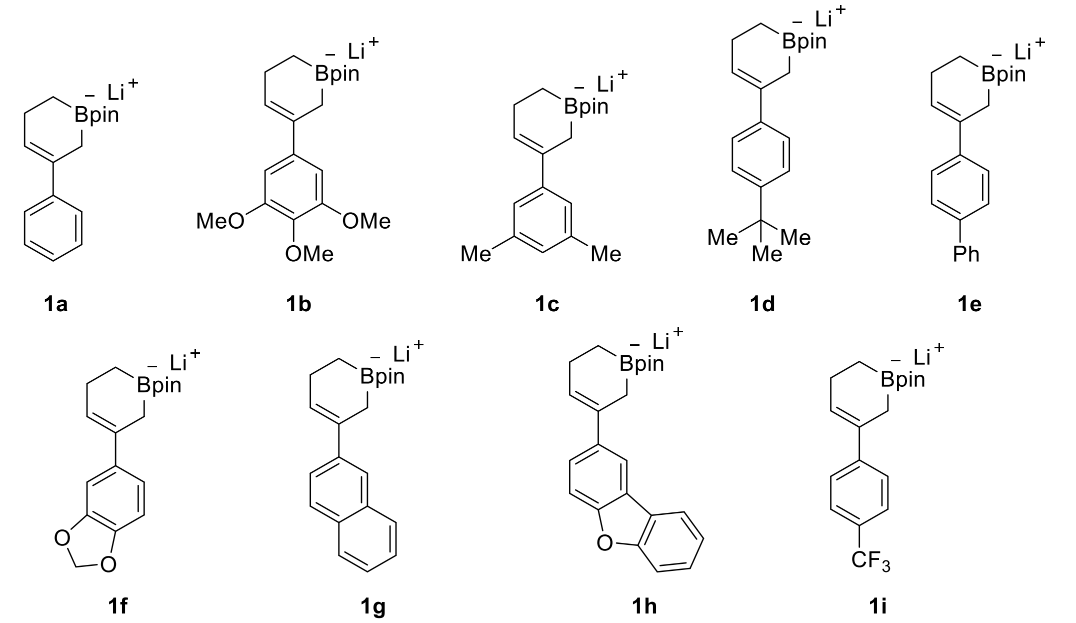

Figure S3. Cyclic allylboronates used in this work

2. General Procedure for reaction optimization

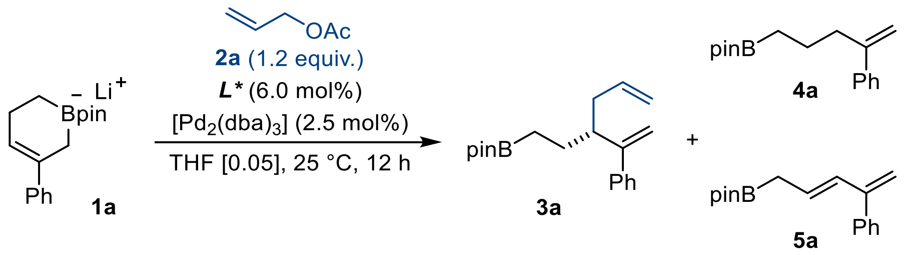

In a glovebox, in an oven dried 5 mL vial equipped with a magnetic stir bar [Pd_2dba_3] (2.3 mg, 0.0025 mmol, 2.5 mol%) and ligand **L** (0.006 mmol, 6 mol%) were dissolved in THF (0.5 mL) and stirred at 25 °C for 15 minutes. Next, this solution was added by pipette into a Schlenk containing allylboronate salt **1a** (32 mg, 0.1 mmol, 1.0 equiv.). The vial was rinsed with additional 1.5 mL of THF. Subsequently, allyl acetate **2a** (13 µL, 0.12 mmol, 1.2 equiv.) was added by microsyringe. The system was closed, and the mixture was stirred at 25 °C. After 12 h, the mixture was filtered on Celite, washed with Et_2O (3 × 5 mL) and concentrated under vacuum. The conversion was measured by ^1H NMR against an internal standard. The enantiomeric ratio (er_{3a}) was measured after oxidation to homoallylic alcohol **7a**.

Table S1. Optimization of the Pd-catalyzed enantioselective allylation[a]

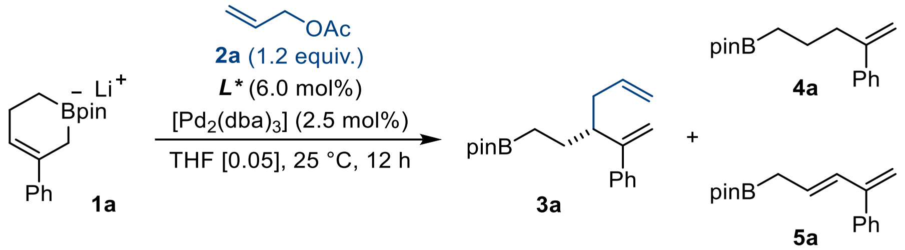

entry	L*	**3a** (%)[b]	**4a** (%)[b]	**5a** (%)[b]	*er*$_{3a}$[c]
1	**L1**	93	-	-	53 : 47
2	**L2**	18	38	-	82 : 18
3	**L3**	<5	-	-	nd
4	**L4**	-	-	22	nd
5	**L5**	47	-	21	77 : 23
6	**L6**	54	-	-	82.5 : 17.5
7	**L7**	42	-	10	60 : 40
8	**L8**	86	-	-	87.5 : 12.5
9	**L9**	75	-	-	92.5 : 7.5
10	**L10**	19	-	-	75.5 : 24.5
11	**L11**	85	-	-	94 : 6
12[d]	**L11**	80	-	-	95 : 5
13[e]	**L11**	70	-	-	96 : 4

[a] Reactions conditions: **1a** (0.1 mmol). [b] Determined by ^{1}H NMR analysis of the crude reaction mixture using an internal standard. [c] Determined by HPLC using a chiral stationary phase after oxidation to the alcohol **7a**. [d] At 0 °C for 12 h. [e] At –20 °C for 48 h

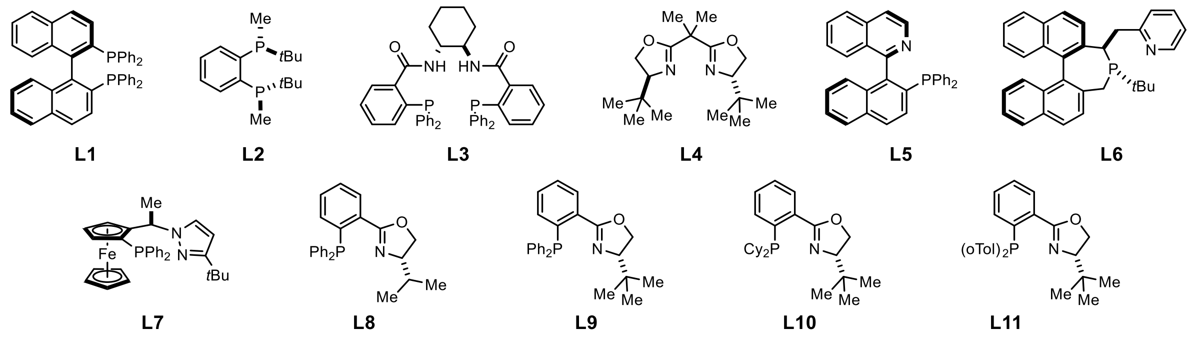

Figure S4. Chiral ligands surveyed

3. Pd-catalyzed enantioselective allylation reactions

3.1 General procedure I (GP-I)

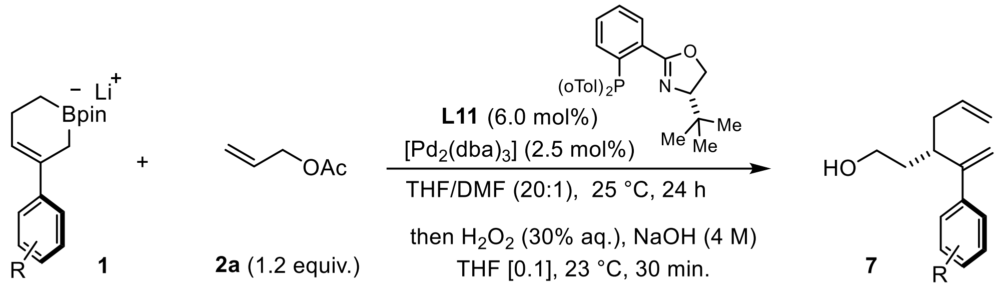

In a glovebox, to an oven-dried 5 mL vial, Pd_2dba_3 (6.9 mg, 7.5 μmol, 2.5 mol%) and ligand **L11** (7.5 mg, 18 μmol, 6 mol%) were dissolved in THF (2 mL), and stirred at 25 °C. After 15 minutes, this solution was added by pipette into a Schlenk containing allylboronates salt **1** (0.3 mmol, 1.0 equiv.). The vial was rinsed with additional 3.7 mL of THF. Subsequently, 0.3 mL of DMF and allyl acetate **2a** (39 μL, 0.36 mmol, 1.2 equiv.) were added by microsyringe. The system was closed, and the mixture was stirred at 25 °C. After 24 h, the mixture was filtered on Celite, washed with Et_2O (3 × 10 mL), and the solution was concentrated under vacuum. The alkali oxidation was conducted next. The crude reaction mixture was dissolved in THF (3.0 mL), and an aqueous NaOH solution (1.5 mL, 4 M) and 30% aq. H_2O_2 solution (1.5 mL) were added at room temperature. After 30 min. at 23 °C, the reaction mixture was extracted with CH_2Cl_2 (3 × 10 mL). The combined organic phases were dried over Na_2SO_4 and concentrated under reduced pressure after filtration. The residue was purified by flash column chromatography on silica gel using pentane/EtOAc as eluent to afford homoallylic alcohol **7**.

Note 1: some allylboronates were prepared as adducts with THF, which should be considered during weighting. The exact amount of THF was determined by ^1H NMR (400 MHz, DMSO-d_6).

Note 2: racemic products were prepared using 2.5 mol% Pd_2dba_3, 6 mol% *rac*-BINAP, 1.0 equiv. allylboronate, and 1.2 equiv. allyl acetate at 25 °C for 18 hours in THF.

3.2 General procedure II (GP-II)

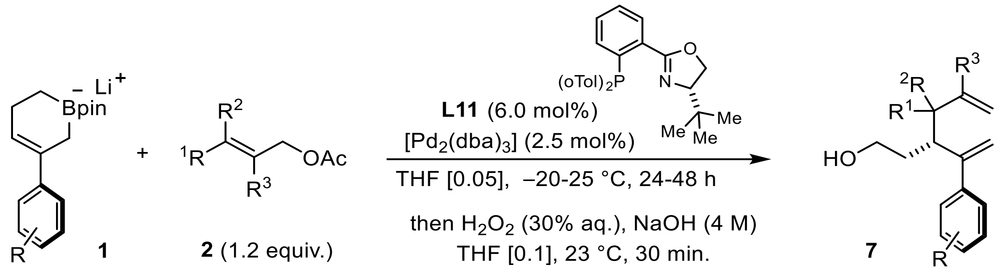

In a glovebox, in an oven-dried 5 mL vial, Pd_2dba_3 (6.9 mg, 7.5 μmol, 2.5 mol%) and ligand **L11** (7.5 mg, 18 μmol, 6 mol%) were dissolved in 2 mL of THF, and stirred at 25 °C. After 15 minutes, this solution was added by pipette into a Schlenk containing allylboronates salt **1** (0.3 mmol, 1.0 equiv.). The vial was rinsed with additional 4 mL of THF. The system was closed and kept in a –20 °C bath for 15 minutes if the reaction temperature was –20 °C. Subsequently, at the same temperature, allyl acetate **2** was added by microsyringe or with 0.5 mL THF if it is a solid. After addition, the system was closed, and the mixture was stirred at appropriate temperature. After appropriate reaction time, the reaction was filtered on Celite, washed with Et_2O (3 × 10 mL), and the solution was concentrated under vacuum. The alkali oxidation was conducted next. The crude reaction mixture was dissolved in THF (3.0 mL), and an aqueous NaOH solution (1.5 mL, 4 M) and 30% aq. H_2O_2 solution (1.5 mL) were added at room temperature. After 30 min. at 23 °C, the reaction mixture was extracted with CH_2Cl_2 (3 × 10 mL). The combined organic phases were dried over Na_2SO_4 and concentrated under reduced pressure after filtration. The residue was purified by flash column chromatography on silica gel using pentane/EtOAc as eluent to afford homoallylic alcohol **7**.

Note 1: some allylboronates were prepared as adducts with THF, which should be considered during weighting. The exact amount of THF was determined by ^1H NMR (400 MHz, DMSO-d_6).

Note 2: racemic products were prepared by using 2.5 mol% Pd_2dba_3, 6 mol% *rac*-BINAP, 1.0 equiv. allylboronate, and 1.2 equiv. allyl acetate at 25 °C for 18 hours in THF.

(*S*)-3-(1-phenylvinyl)hex-5-en-1-ol (7a)

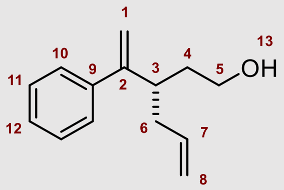

7a

Following **GP-II** using boronate **1a** (0.3 mmol, 1.0 equiv.) and allyl acetate **2a** (0.36 mmol, 1.2 equiv.). The reaction was conducted at –20 °C for 48 h to afford compound **7a** as a yellow oil in 62% yield (38 mg) after chromatography on silica gel (eluent: pentane/EtOAc, from 10:1 to 7.5:1).

TLC: 0.3, pentane/EtOAc = 5:1

^{1}H NMR (400 MHz, $CDCl_3$) δ (ppm) = 7.38 – 7.25 (m, 5H, *H10, H11 and H12*), 5.84 – 5.72 (m, 1H, *H7*), 5.30 (d, $^4J_{H\text{-}H}$ = 1.0 Hz, 1H, *H1*), 5.08 (bs, 1H, *H1*), 5.06 – 4.98 (m, 2H, *H8*), 3.76 – 3.61 (m, 2H, *H5*), 2.88 – 2.79 (m, 1H, *H3*), 2.37 – 2.27 (m, 1H, *H6*), 2.26 – 2.16 (m, 1H, *H6*), 1.88 – 1.72 (m, 2H, *H4*), 1.21 (t, $^3J_{H\text{-}H}$ = 5.0 Hz, 1H, *H13*).

^{13}C{^{1}H} NMR (101 MHz, $CDCl_3$) δ (ppm) = 152.1 (*C2*), 143.0 (*C9*), 136.7 (*C7*), 128.4 (*C11*), 127.5 (*C12*), 126.9 (*C10*), 116.5 (*C8*), 113.1 (*C1*), 61.1 (*C5*), 40.5 (*C3*), 39.3 (*C6*), 36.9 (*C4*).

HRMS (ESI +**)**: calculated for $C_{14}H_{19}O$ $[M+H]^+$: 203.1431; found: 203.1425.

IR (neat) ν (cm^{-1}) = 3337, 3078, 2925, 1639, 1493, 1441, 1047, 1028, 994, 908, 777, 700.

HPLC: 95.5:4.5 *er*, OD, 1 mL/min, hexane:*i*PrOH = 95:5, λ = 254 nm, 30 °C, t_R (major) = 9.1 min, t_R (minor) = 7.5 min.

$[\alpha]^{20}_D$: +6.5° (*c* = 0.5, $CHCl_3$).

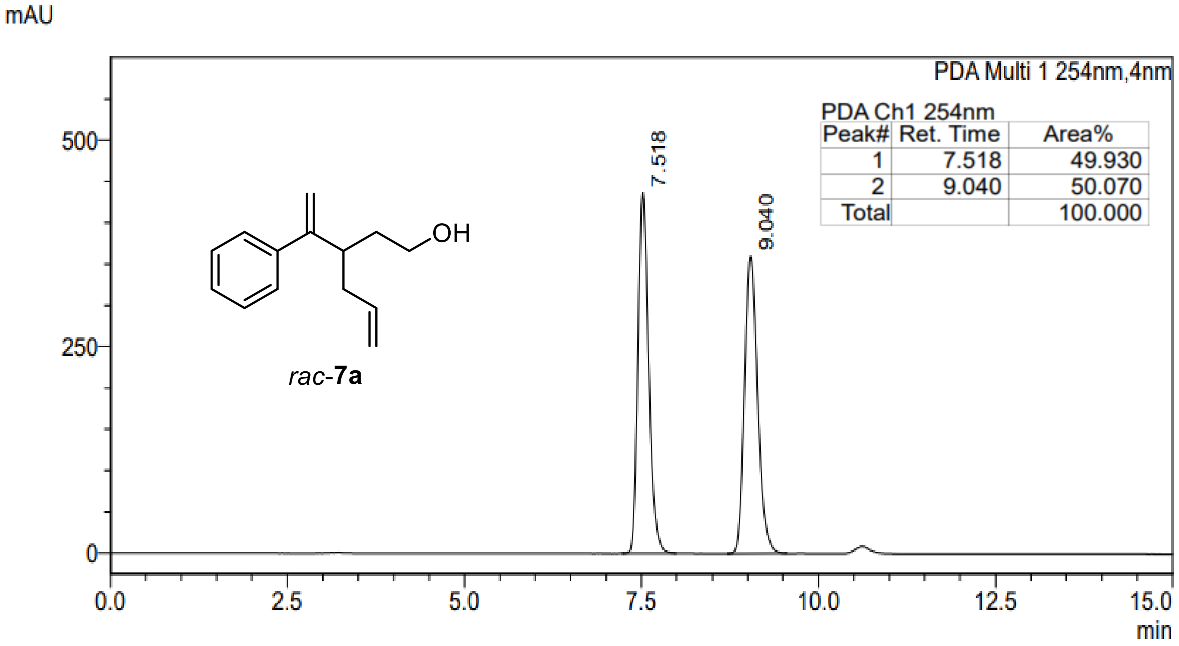

PDA Ch1 254nm

Peak#	Ret. Time	Area%
1	7.518	49.930
2	9.040	50.070
Total		100.000

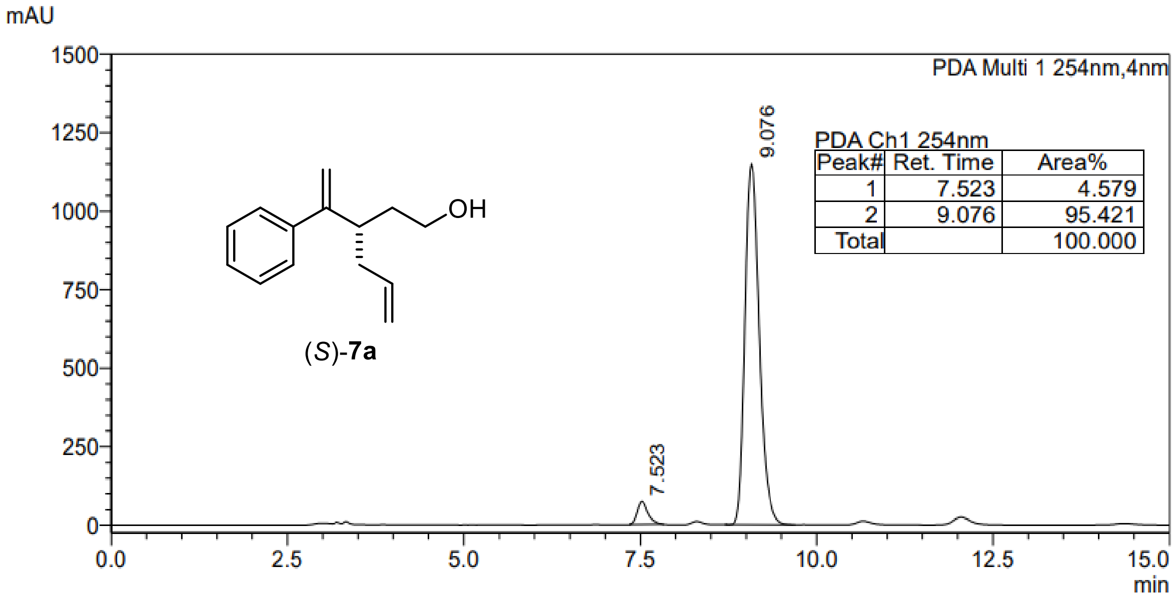

PDA Ch1 254nm

Peak#	Ret. Time	Area%
1	7.523	4.579
2	9.076	95.421
Total		100.000

(*S*)-3-(1-(3,4,5-trimethoxyphenyl)vinyl)hex-5-en-1-ol (7b)

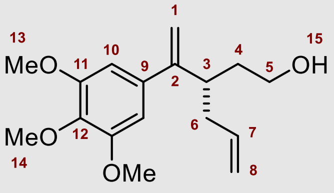

7b

Following **GP-II** using boronate **1b** (0.3 mmol, 1.0 equiv.) and allyl acetate **2a** (0.36 mmol, 1.2 equiv.), The reaction was conducted at –20 °C for 48 h to afford compound **7b** as a yellow oil in 82% yield (72 mg) after chromatography on silica gel (eluent: pentane/EtOAc, from 10:1 to 6:1).

TLC: 0.25, pentane/EtOAc = 5:1

^{1}H NMR (400 MHz, $CDCl_3$) δ (ppm) = 6.59 (s, 2H, *H10*), 5.85 – 5.73 (m, 1H, *H7*), 5.29 (d, $^4J_{H-H}$ = 0.9 Hz, 1H, *H1*), 5.08 – 4.99 (m, 3H, *H1 and H8*), 3.87 (s, 6H, *H13*), 3.85 (s, 3H, *H14*), 3.76 – 3.64 (m, 2H, *H5*), 2.99 – 2.90 (m, 1H, *H3*), 2.37 – 2.28 (m, 1H, *H6*), 2.27 – 2.17 (m, 1H, *H6*), 1.88 – 1.71 (m, 2H, *H4*), 1.24 (t, $^3J_{H-H}$ = 5.2 Hz, 1H, *H15*).

^{13}C{^{1}H} NMR (101 MHz, $CDCl_3$) δ (ppm) = 153.1 (*C11*), 152.2 (*C2*), 138.9 (*C9*), 137.7 (*C12*), 136.7 (*C7*), 116.5 (*C8*), 112.6 (*C1*), 104.2 (*C10*), 61.0 (*C5 and C14*), 56.3 (*C13*), 40.5 (*C3*), 39.4 (*C6*), 37.1 (*C4*).

HRMS (ESI +**)**: calculated for $C_{17}H_{25}O_4$ $[M+H]^+$: 293.1748; found: 293.1766.

IR (neat) ν (cm^{-1}) = 3429, 2935, 2836, 1579, 1505, 1409, 1330, 1237, 1123, 1004, 909, 842.

HPLC: 93:7 *er*, AD, 1 mL/min, hexane:*i*PrOH = 90:10, λ = 254 nm, 30 °C, t_R (major) = 13.4 min, t_R (minor) = 17.1 min.

$[\alpha]^{20}_D$: +3.4° (*c* = 0.5, $CHCl_3$).

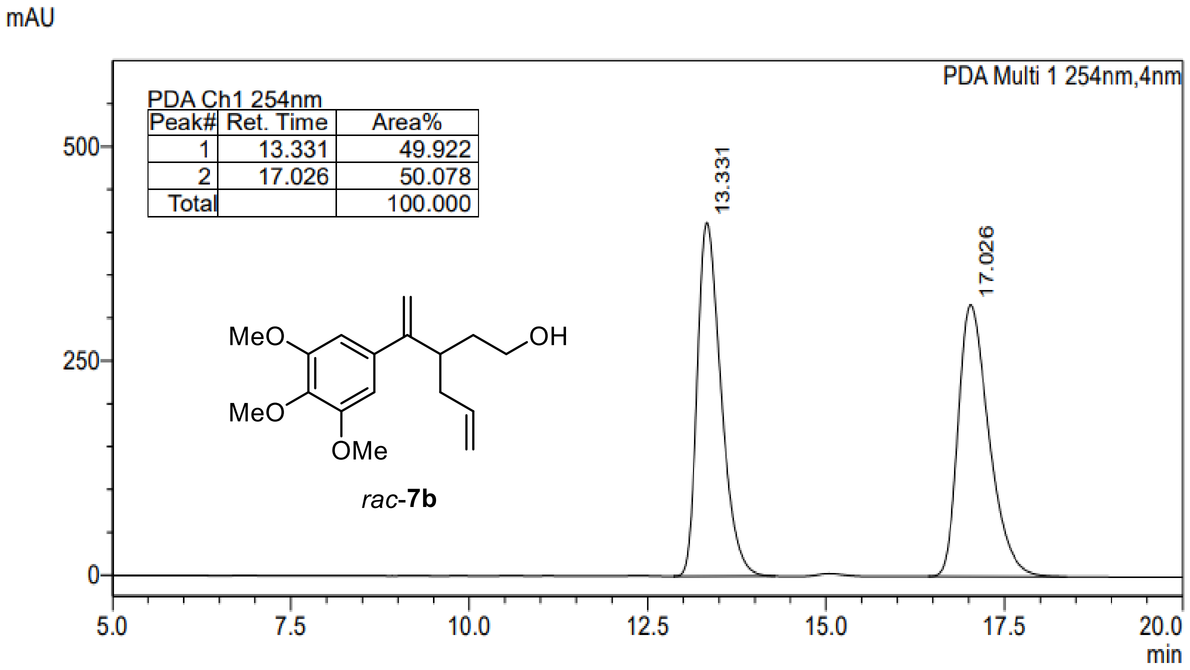
mAU
PDA Multi 1 254nm,4nm
PDA Ch1 254nm
Peak# Ret. Time Area%
1 13.331 49.922
2 17.026 50.078
Total 100.000
13.331
17.026
MeO
MeO
OMe
OH
rac-**7b**
500
250
0
5.0
7.5
10.0
12.5
15.0
17.5
20.0
min

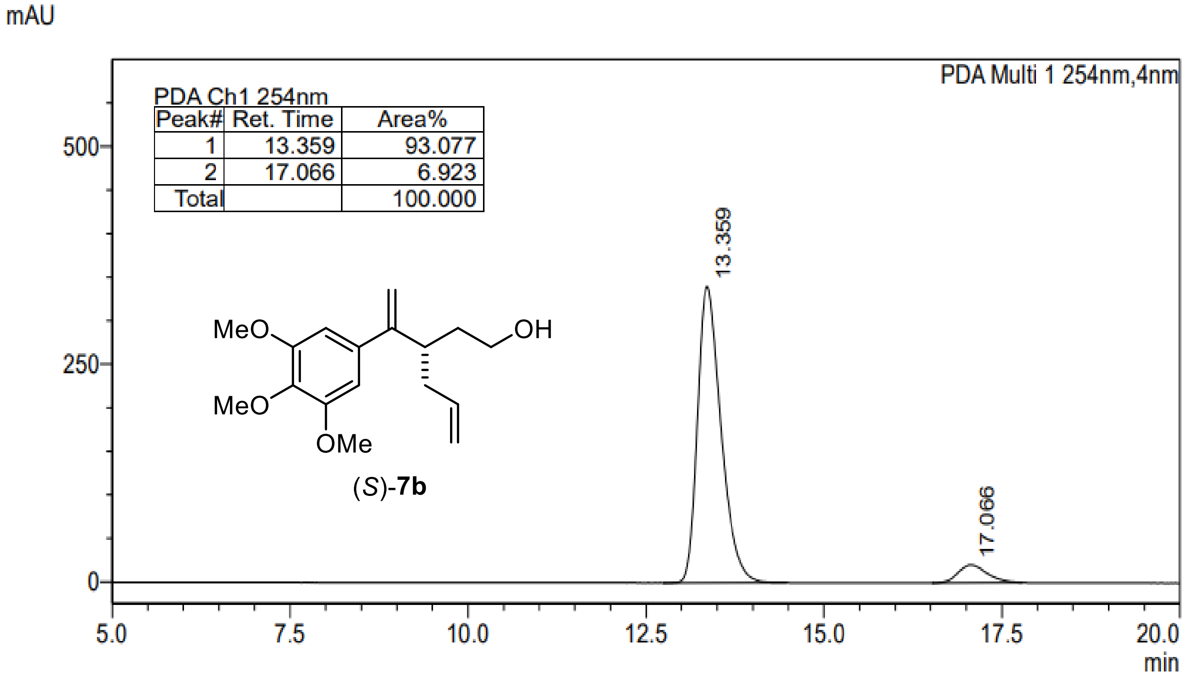
mAU
PDA Multi 1 254nm,4nm
PDA Ch1 254nm
Peak# Ret. Time Area%
1 13.359 93.077
2 17.066 6.923
Total 100.000
13.359
17.066
MeO
MeO
OMe
OH
(*S*)-**7b**
500
250
0
5.0
7.5
10.0
12.5
15.0
17.5
20.0
min

(*S*)-3-(1-(3,5-dimethylphenyl)vinyl)hex-5-en-1-ol (7c)

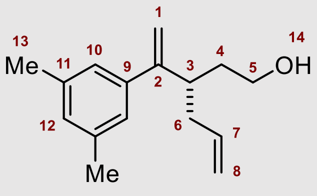

Following **GP-I** using boronate **1c** (0.3 mmol, 1.0 equiv.) and allyl acetate **2a** (0.36 mmol, 1.2 equiv.), compound **7c** was prepared as a yellow oil (56 mg, 81% yield) after chromatography on silica gel (eluent: pentane/EtOAc, from 10:1 to 7.5:1).

TLC: 0.5, pentane:EtOAc = 5:1

^{1}H NMR (400 MHz, $CDCl_3$) δ (ppm) = 6.96 (bs, 2H, *H10*), 6.92 (bs, 1H, *H12*), 5.85 – 5.73 (m, 1H, *H7*), 5.26 (d, $^{4}J_{H\text{-}H}$ = 1.1 Hz, 1H, *H1*), 5.07 – 4.99 (m, 3H, *H1 and H8*), 3.87 (s, 6H, *H13*), 3.75 – 3.62 (m, 2H, *H5*), 2.85 – 2.76 (m, 1H, *H3*), 2.36 – 2.26 (m, 7H, *H6 and H13*), 2.24 – 2.15 (m, 1H, *H6*), 1.87 – 1.70 (m, 2H, *H4*), 1.19 (t, $^{3}J_{H\text{-}H}$ = 5.5 Hz, 1H, *H14*).

^{13}C{^{1}H} NMR (101 MHz, $CDCl_3$) δ (ppm) = 152.3 (*C2*), 143.0 (*C9*), 137.8 (*C11*), 136.8 (*C7*), 129.2 (*C12*), 124.7 (*C10*), 116.4 (*C8*), 112.6 (*C1*), 61.2 (*C5*), 40.5 (*C3*), 39.2 (*C6*), 36.8 (*C4*), 21.5 (*C13*).

HRMS (ESI +**)**: calculated for $C_{16}H_{23}O$ $[M+H]^+$: 231.1744; found: 231.1736.

IR (neat) ν (cm^{-1}) = 3324, 2919, 1639, 1598, 1441, 1047, 994, 908, 893, 851, 730, 666.

HPLC: 92:8 *er*, OZ, 1 mL/min, hexane:*i*PrOH = 95:5, λ = 254 nm, 30 °C, t_R (major) = 5.5 min, t_R (minor) = 5.0 min.

[α]$^{20}_{D}$: +10.4° (*c* = 0.5, $CHCl_3$).

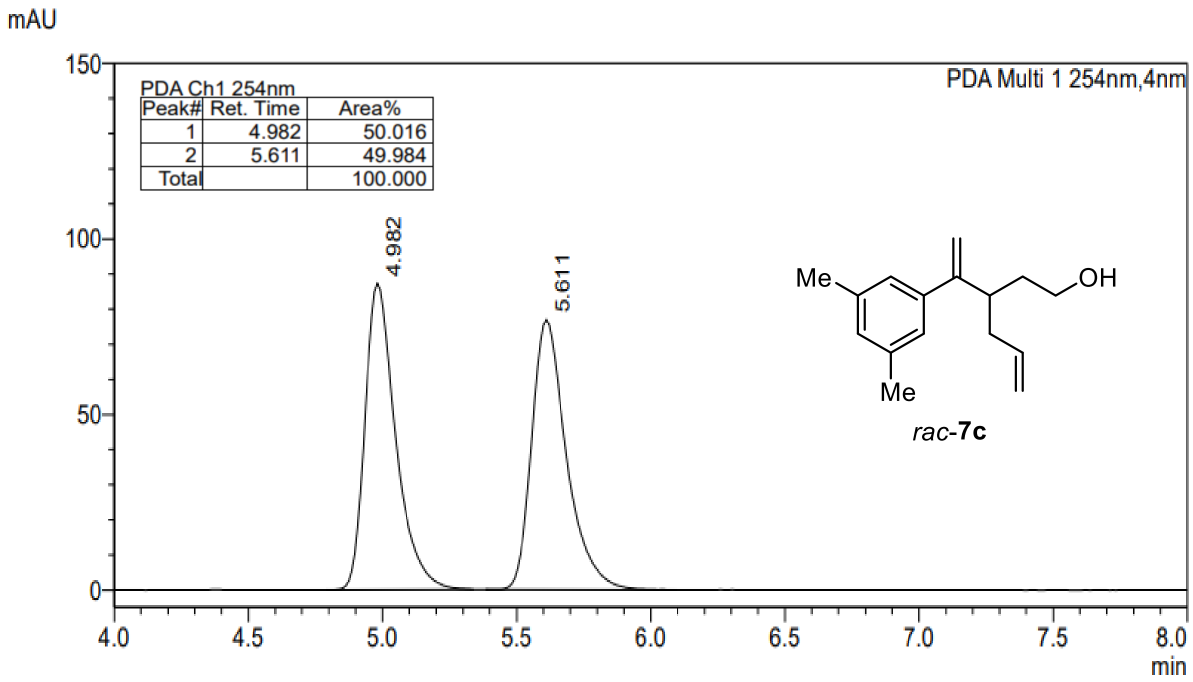
mAU
PDA Ch1 254nm
Peak# Ret. Time Area%
1 4.982 50.016
2 5.611 49.984
Total 100.000
PDA Multi 1 254nm,4nm
4.982
5.611
Me
OH
Me
rac-7c
min

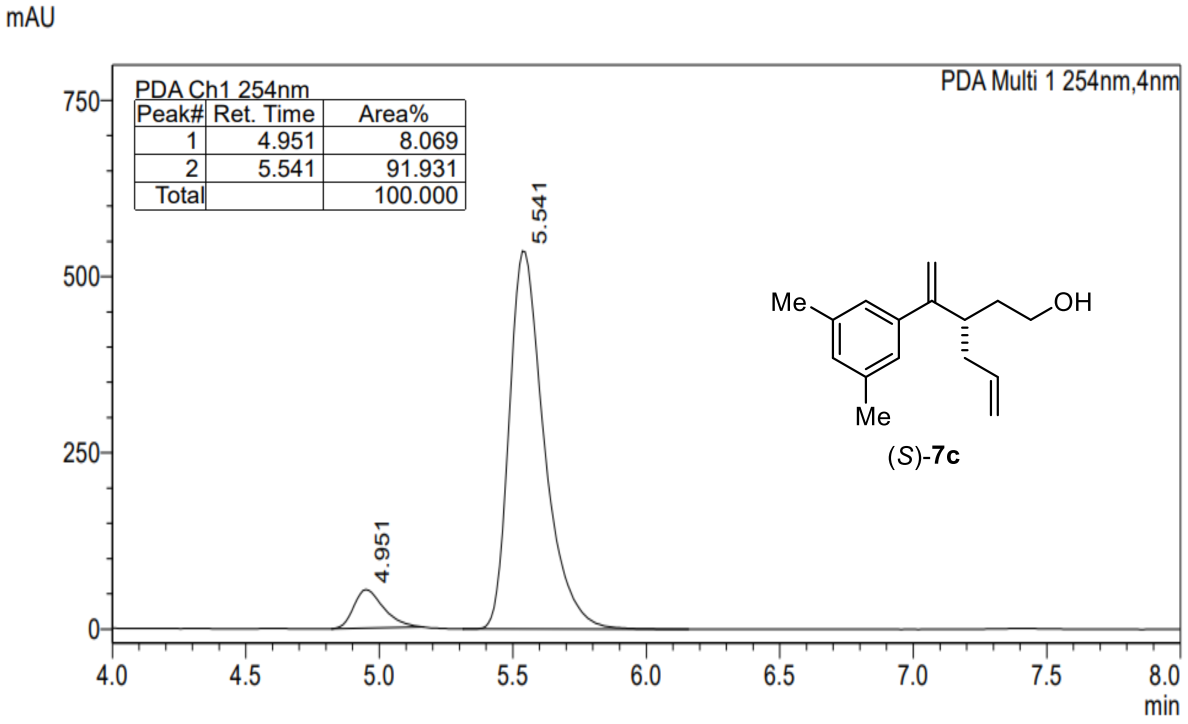
mAU
PDA Ch1 254nm
Peak# Ret. Time Area%
1 4.951 8.069
2 5.541 91.931
Total 100.000
PDA Multi 1 254nm,4nm
5.541
4.951
Me
OH
Me
(S)-7c
min

(*S*)-3-(1-(4-(tert-butyl)phenyl)vinyl)hex-5-en-1-ol (7d)

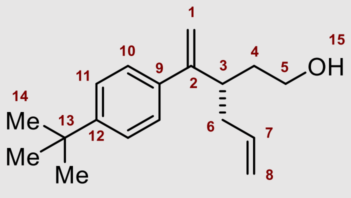

7d

Following **GP-I** using boronate **1d** (0.3 mmol, 1.0 equiv.) and allyl acetate **2a** (0.36 mmol, 1.2 equiv.), compound **7d** was prepared as a yellow oil (65 mg, 84% yield, 93:7 *er*) after chromatography on silica gel (eluent: pentane/EtOAc, from 10:1 to 7.5:1).

TLC: 0.3, pentane/EtOAc = 5:1

^{1}H NMR (400 MHz, $CDCl_3$) δ (ppm) = 7.36 – 7.26 (m, 4H, *H10 and H11*), 5.86 – 5.73 (m, 1H, *H7*), 5.31 (d, $^4J_{H-H}$ = 1.1 Hz, 1H, *H1*), 5.06 – 4.98 (m, 3H, *H1 and H8*), 3.75 – 3.61 (m, 2H, *H5*), 2.87 – 2.78 (m, 1H, *H3*), 2.38 – 2.28 (m, 1H, *H6*), 2.26 – 2.16 (m, 1H, *H6*), 1.89 – 1.71 (m, 2H, *H4*), 1.32 (s, 9H, *H14*), 1.22 (t, $^3J_{H-H}$ = 5.2 Hz, 1H, *H15*).

^{13}C{^{1}H} NMR (101 MHz, $CDCl_3$) δ (ppm) = 151.7 (*C2*), 150.4 (*C12*), 139.8 (*C9*), 136.9 (*C7*), 126.4 (*C12*), 125.3 (*C10*), 116.4 (*C8*), 112.4 (*C1*), 61.2 (*C5*), 40.3 (*C3*), 39.4 (*C6*), 37.0 (*C4*), 34.6 (*C13*), 31.5 (*C14*).

HRMS (ESI +**)**: calculated for $C_{18}H_{27}O$ $[M+H]^+$: 259.2057; found: 259.2039.

IR (neat) ν (cm^{-1}) = 3337, 3078, 2961, 2868, 1639, 1510, 1363, 1269, 1048, 1016, 909, 839.

HPLC: 93:7 *er*, AD, 1 mL/min, hexane:*i*PrOH = 99:1, λ = 254 nm, 30 °C, t_R (major) = 19.9 min, t_R (minor) = 18.1 min.

$[\alpha]^{20}_D$: +7.8° (*c* = 0.5, $CHCl_3$).

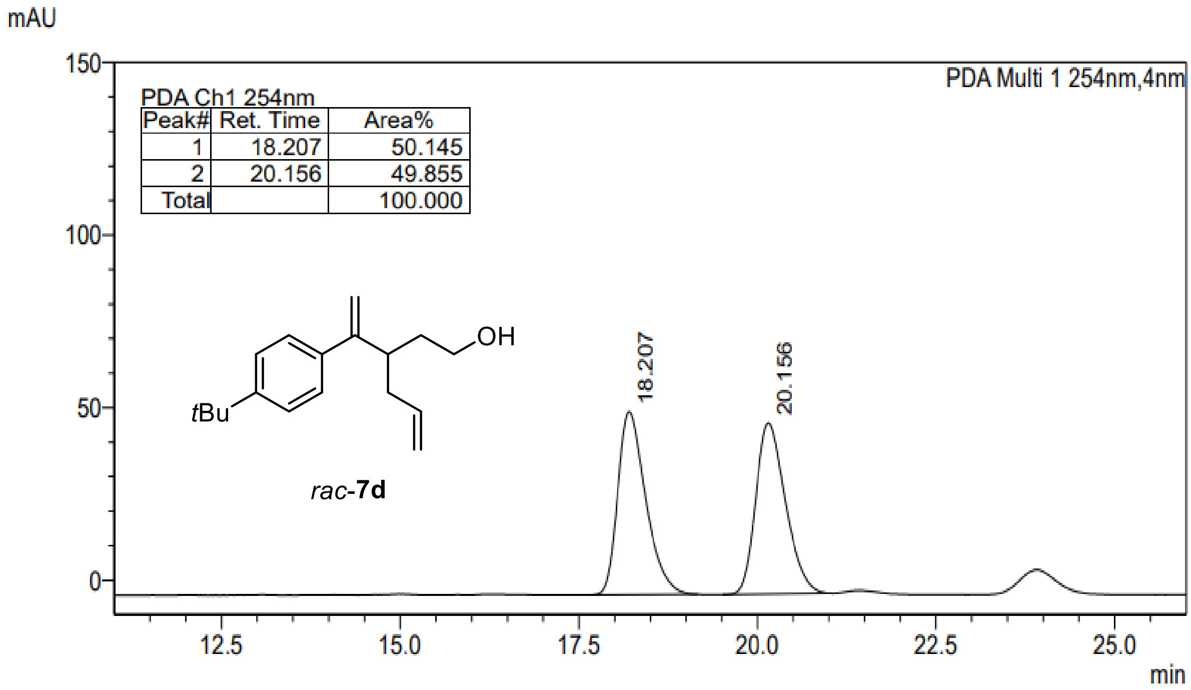

PDA Ch1 254nm

Peak#	Ret. Time	Area%
1	18.207	50.145
2	20.156	49.855
Total		100.000

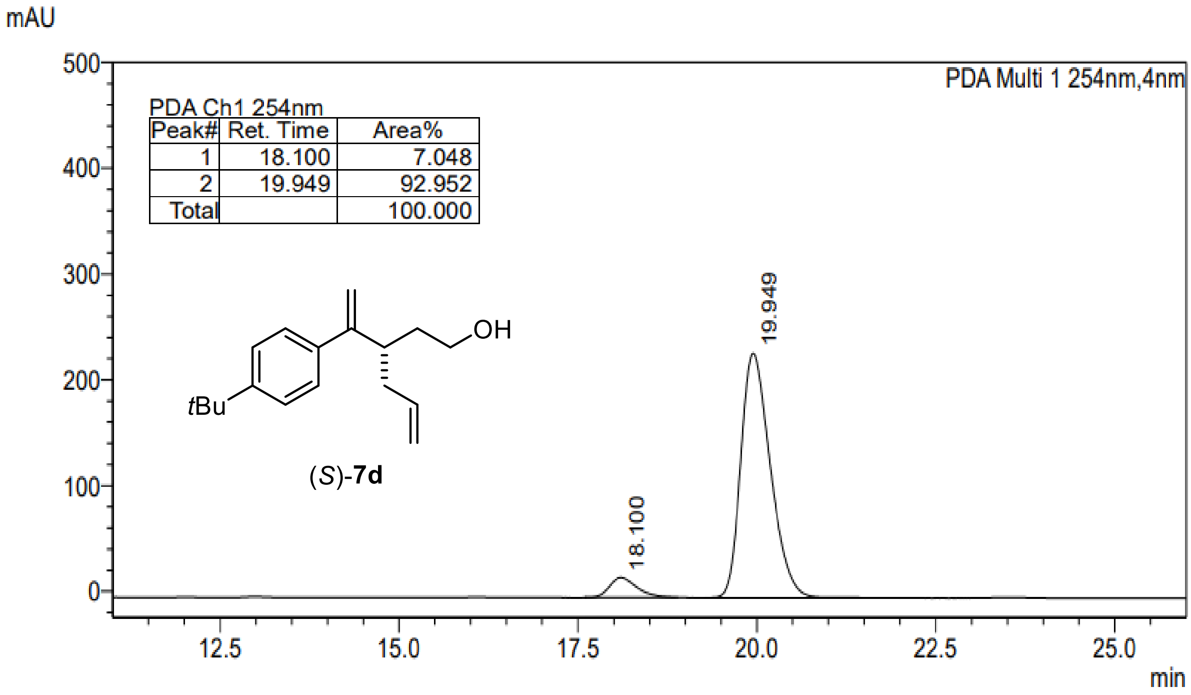

PDA Ch1 254nm

Peak#	Ret. Time	Area%
1	18.100	7.048
2	19.949	92.952
Total		100.000

(*S*)-3-(1-([1,1'-biphenyl]-4-yl)vinyl)hex-5-en-1-ol (7e)

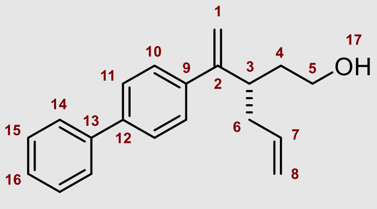

7e

Following **GP-I** using boronate **1e** (0.3 mmol, 1.0 equiv.) and allyl acetate **2a** (0.36 mmol, 1.2 equiv.), compound **7e** was prepared as a white solid (45 mg, 89% yield) after chromatography on silica gel (eluent: pentane/EtOAc, from 10:1 to 6:1).

TLC: 0.2, pentane/EtOAc = 5:1

^{1}H NMR (400 MHz, $CDCl_3$) δ (ppm) = 7.38 – 7.32 (m, 4H, *H11 and/or H14*), 7.38 – 7.32 (m, 4H, *H11*),7.36 – 7.26 (m, 4H, *H10 and H11*), 5.87 – 5.75 (m, 1H, *H7*), 5.38 (d, $^4J_{H\text{-}H}$ = 0.9 Hz, 1H, *H1*), 5.11 (bs, 1H), 5.09 – 5.00 (m, 2H, *H8*), 3.78 – 3.65 (m, 2H, *H5*), 2.93 – 2.85 (m, 1H, *H3*), 2.41 – 2.32 (m, 1H, *H6*), 2.30 – 2.21 (m, 1H, *H6*), 1.92 – 1.75 (m, 2H, *H4*), 1.27 (t, $^3J_{H\text{-}H}$ = 5.1 Hz, 1H, *H17*).

^{13}C{^{1}H} NMR (101 MHz, $CDCl_3$) δ (ppm) = 151.6 (*C2*), 141.9 (*C9*), 140.8 (*C13*), 140.3 (*C12*), 136.7 (*C7*), 128.9, 127.4 (*C16*), 127.2, 127.1 (2 peaks merge), 116.5 (*C8*), 113.0 (*C1*), 61.1 (*C5*), 40.3 (*C3*), 39.3 (*C6*), 37.0 (*C4*).

HRMS (ESI +**)**: calculated for $C_{20}H_{23}O$ $[M+H]^+$: 279.1744; found: 279.1758.

IR (neat) ν (cm^{-1}) = 3271, 2901, 1639, 1486, 1046, 997, 904, 848, 772, 738, 691.

m.p.: 55.1–55.6 °C.

HPLC: 92:8 *er*, OD, 1 mL/min, hexane:*i*PrOH = 95:5, λ = 254 nm, 30 °C, t_R (major) = 10.5 min, t_R (minor) = 11.7 min.

$[\alpha]^{20}_D$: +9.0° (*c* = 0.5, $CHCl_3$).

Note: With boronate **1e**, the use of allyl carbonate gave a comparable outcome to the acetate (99% NMR yield, 93:7 *er*) while allyl chloride led to reduced reactivity and selectivity (73% NMR yield, 87:13 *er*).

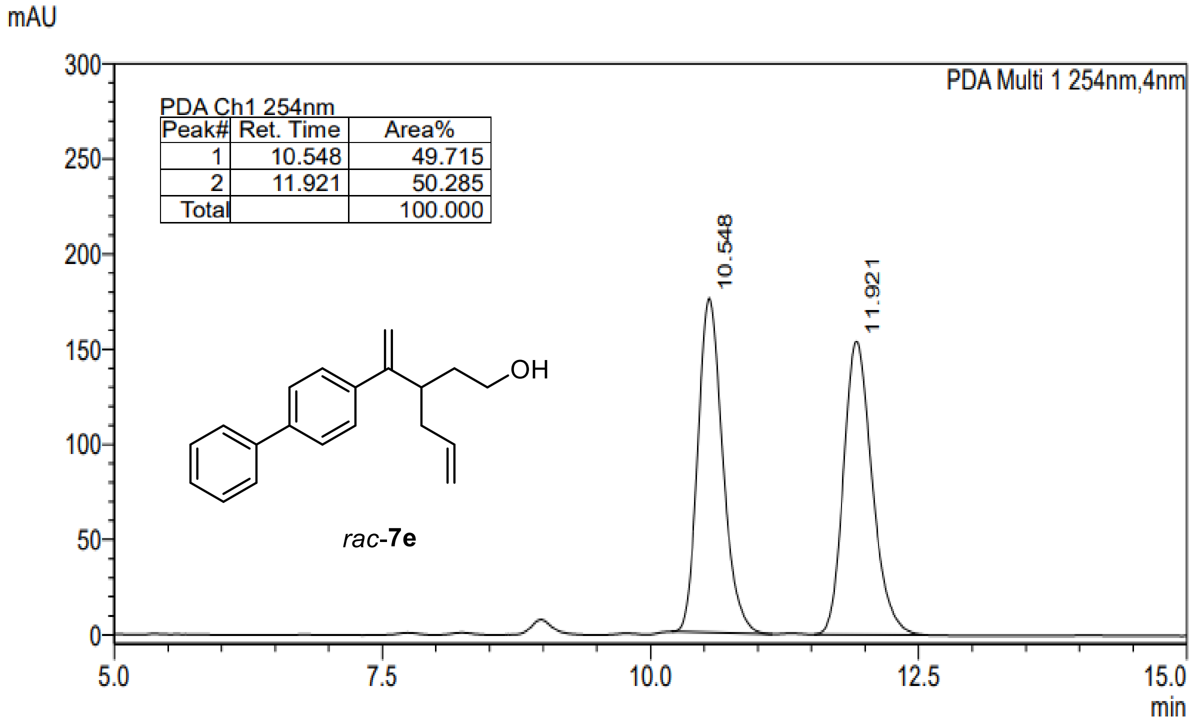
mAU
PDA Multi 1 254nm,4nm
PDA Ch1 254nm
Peak# | Ret. Time | Area%
1 | 10.548 | 49.715
2 | 11.921 | 50.285
Total | | 100.000
OH
rac-7e
10.548
11.921
300
250
200
150
100
50
0
5.0
7.5
10.0
12.5
15.0
min

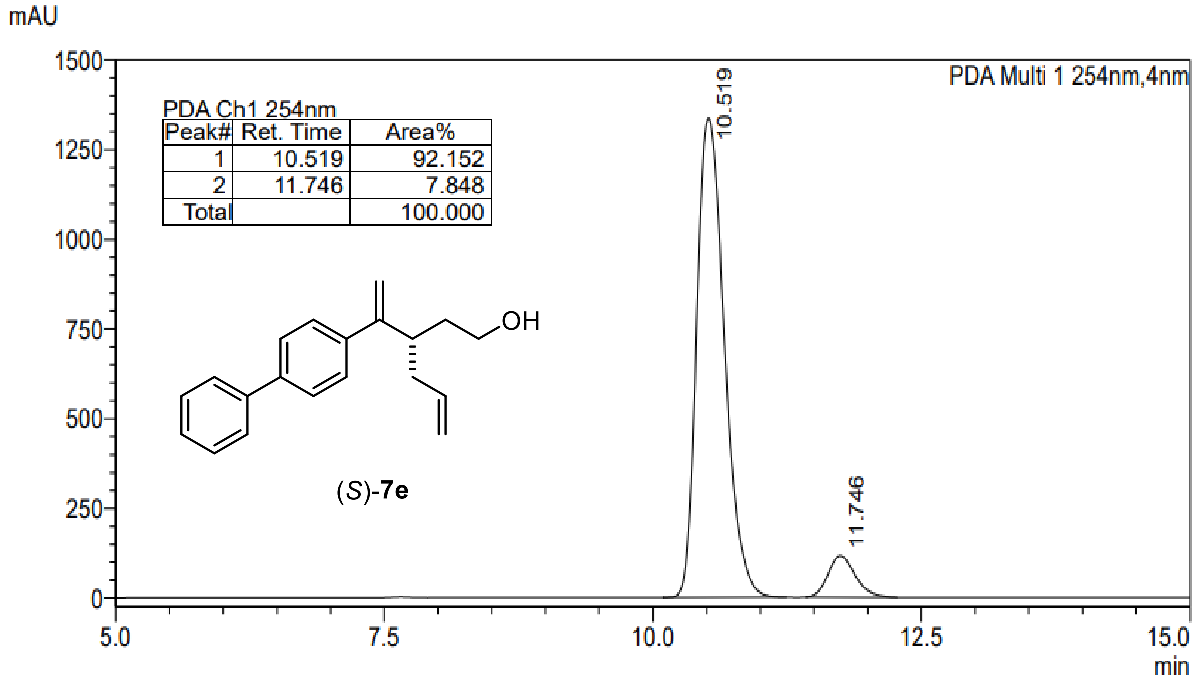
mAU
PDA Multi 1 254nm,4nm
PDA Ch1 254nm
Peak# | Ret. Time | Area%
1 | 10.519 | 92.152
2 | 11.746 | 7.848
Total | | 100.000
OH
(S)-7e
10.519
11.746
1500
1250
1000
750
500
250
0
5.0
7.5
10.0
12.5
15.0
min

(*S*)-3-(1-(benzo[d][1,3]dioxol-5-yl)vinyl)hex-5-en-1-ol (7f)

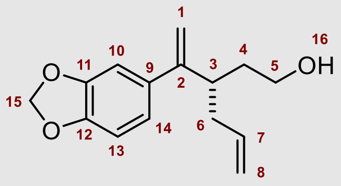

7f

Following **GP-I** using boronate **1f** (0.3 mmol, 1.0 equiv.) and allyl acetate **2a** (0.36 mmol, 1.2 equiv.), compound **7f** was prepared as a yellow oil in 90% yield (66 mg) after chromatography on silica gel (eluent: pentane/EtOAc, from 10:1 to 7.5:1).

TLC: 0.2, pentane/EtOAc = 5:1

^{1}H NMR (400 MHz, $CDCl_3$) δ (ppm) = 6.86 (d, $^4J_{H-H}$ = 1.8 Hz, 1H, *H10*), 6.84 (dd, $^3J_{H-H}$ = 8.0, $^4J_{H-H}$ = 1.8 Hz, 1H, *H14*), 6.76 (d, $^3J_{H-H}$ = 8.0 Hz, 1H, *H13*), 5.95 (s, 2H, *H15*), 5.82 – 5.71 (m, 1H, *H7*), 5.23 (d, $^4J_{H-H}$ = 0.9 Hz, 1H, *H1*), 5.06 – 4.98 (m, 3H, *H1 and H8*), 3.74 – 3.61 (m, 2H, *H5*), 2.80 – 2.71 (m, 1H, *H3*), 2.35 – 2.25 (m, 1H, *H6*), 2.25 – 2.15 (m, 1H, *H6*), 1.87 – 1.69 (m, 2H, *H4*), 1.21 (t, $^3J_{H-H}$ = 4.3 Hz, 1H, *H16*).

^{13}C{^{1}H} NMR (101 MHz, $CDCl_3$) δ (ppm) = 151.7 (*C9*), 147.7 (*C12*), 147.0 (*C11*), 137.3 (*C2*), 136.7 (*C7*), 120.2 (*C14*), 116.5 (*C8*), 112.3 (*C1*), 108.2 (*C13*), 107.5 (*C10*), 101.2 (*C15*), 61.1 (*C5*), 40.6 (*C3*), 39.3 (*C6*), 37.0 (*C4*).

HRMS (ESI +**)**: calculated for $C_{15}H_{19}O_3$ $[M+Na]^+$: 247.1329; found: 247.1324.

IR (neat) ν (cm^{-1}) = 3339, 3076, 2885, 1638, 1487, 1435, 1229, 1037, 936, 912, 813, 737.

HPLC: 92:8 *er*, AD, 1 mL/min, hexane:*i*PrOH = 95:5, λ = 208 nm, 30 °C, t_R (major) = 14.6 min, t_R (minor) = 13.8 min.

[α]$^{20}_D$: +7.3° (*c* = 0.5, $CHCl_3$).

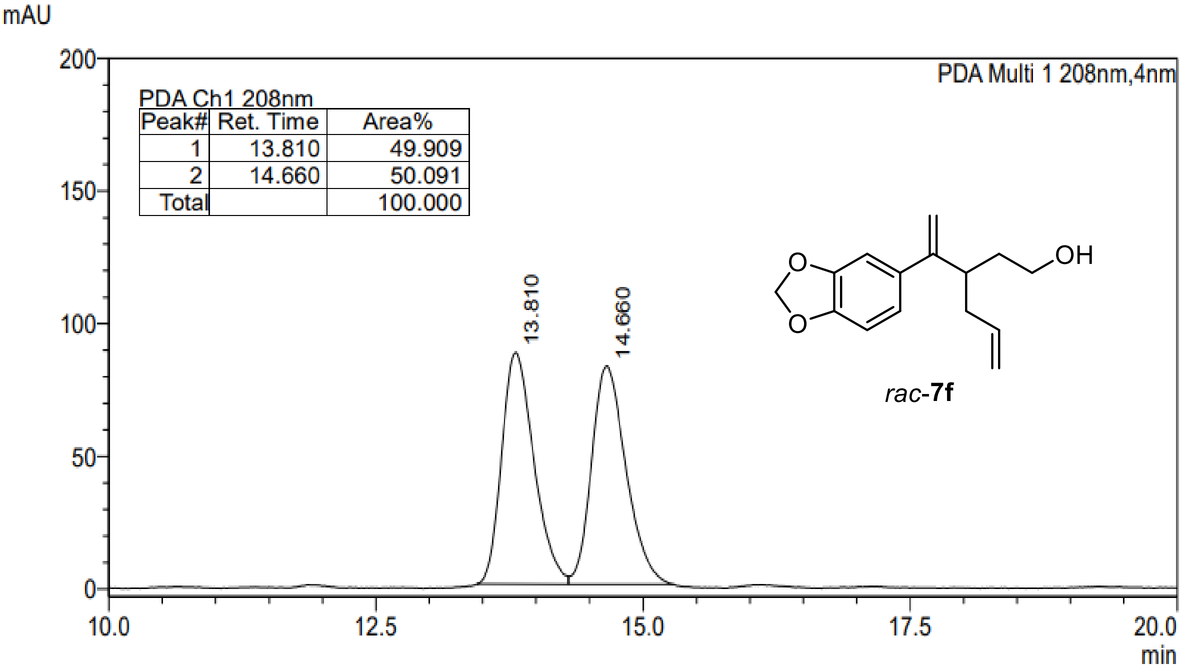

PDA Ch1 208nm

Peak#	Ret. Time	Area%
1	13.810	49.909
2	14.660	50.091
Total		100.000

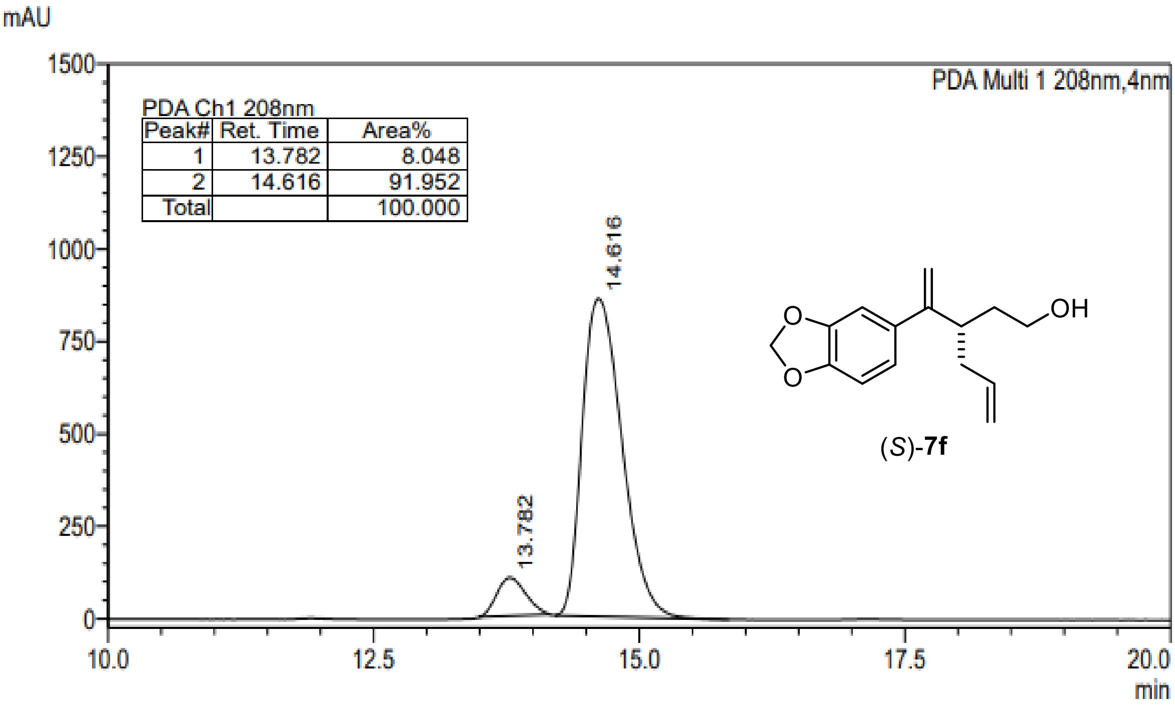

PDA Ch1 208nm

Peak#	Ret. Time	Area%
1	13.782	8.048
2	14.616	91.952
Total		100.000

(*S*)-3-(1-(naphthalen-2-yl)vinyl)hex-5-en-1-ol (7g)

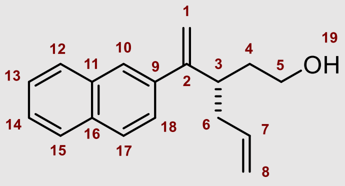

7g

Following **GP-II** using boronate **1g** (0.3 mmol, 1.0 equiv.) and allyl acetate **2a** (0.36 mmol, 1.2 equiv.). The reaction was conducted at –20 °C for 48 h to afford compound **7g** as a yellow oil in 74% yield (56 mg) after chromatography on silica gel (eluent: pentane/EtOAc, from 10:1 to 7.5:1).

TLC: 0.3, pentane/EtOAc = 5:1

^{1}H NMR (400 MHz, $CDCl_3$) δ (ppm) = 7.86 – 7.77 (m, 4H, *H10, H12, H15 and H17*), 7.52 (dd, $^3J_{H\text{-}H}$ = 8.4, $^4J_{H\text{-}H}$ = 1.8 Hz, 1H, *H18*), 7.50 – 7.42 (m, 1H, *H13 and H14*), 5.88 – 5.76 (m, 1H, *H7*), 5.44 (d, $^4J_{H\text{-}H}$ = 0.9 Hz, 1H, *H1*), 5.18 (bs, 1H, *H1*), 5.08 – 5.00 (m, 2H, *H8*), 3.79 – 3.66 (m, 2H, *H5*), 3.03 – 2.94 (m, 1H, *H3*), 2.43 – 2.33 (m, 1H, *H6*), 2.32 – 2.22 (m, 1H, *H6*), 1.93 – 1.78 (m, 2H, *H4*), 1.23 (bs, 1H, *H19*).

^{13}C{^{1}H} NMR (101 MHz, $CDCl_3$) δ (ppm) = 152.0 (*C2*), 140.3 (*C9*), 136.7 (*C7*), 133.5 (*C11*), 132.9 (*C16*), 128.3, 128.0, 127.7, 126.3 (*C13 or C14*), 126.0 (*C13 or C14*), 125.5 (*C18*), 125.4 (*C10*), 116.6 (*C8*), 113.5 (*C1*), 61.2 (*C5*), 40.4 (*C3*), 39.3 (*C6*), 37.0 (*C4*).

HRMS (ESI +**)**: calculated for $C_{18}H_{21}O$ $[M+H]^+$: 253.1587; found: 253.1582.

IR (neat) ν (cm^{-1}) = 3326, 3056, 2928, 1638, 1435, 1046, 994, 893, 858, 818, 749.

HPLC: 97:3 *er*, AD, 1 mL/min, hexane:*i*PrOH = 95:5, λ = 254 nm, 30 °C, t_R (major) = 10.7 min, t_R (minor) = 11.7 min.

$[\alpha]^{20}_D$: +11.4° (*c* = 0.5, $CHCl_3$).

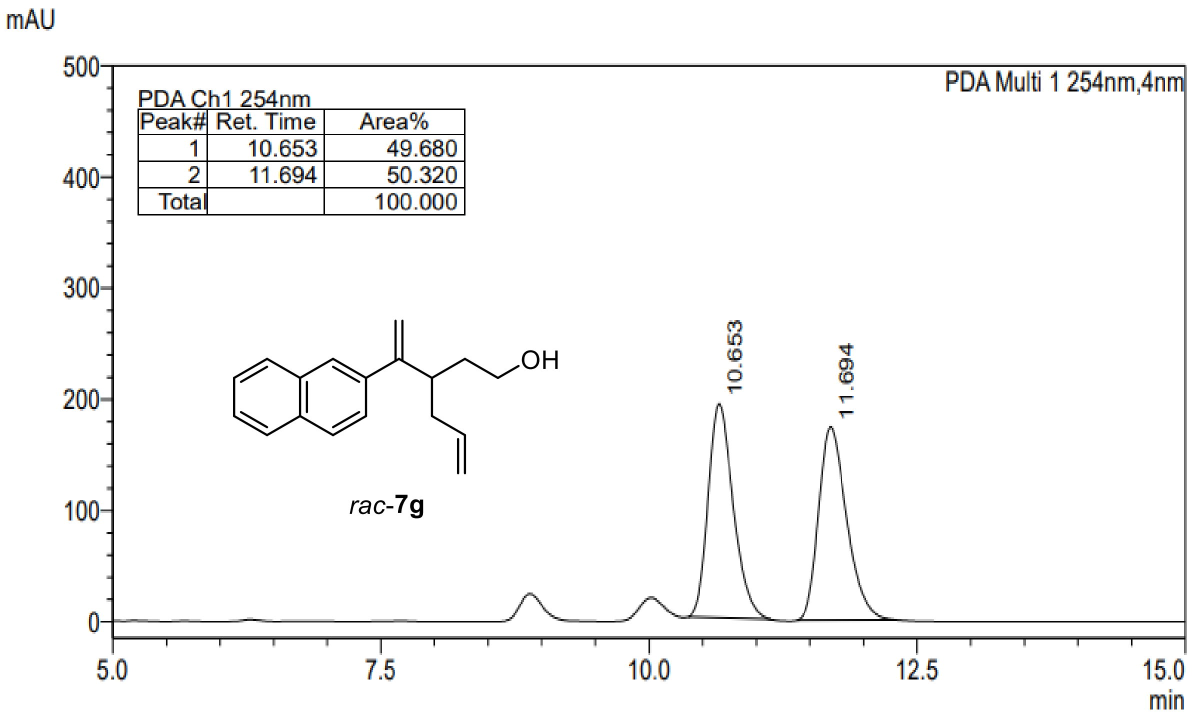

Peak#	Ret. Time	Area%
1	10.653	49.680
2	11.694	50.320
Total		100.000

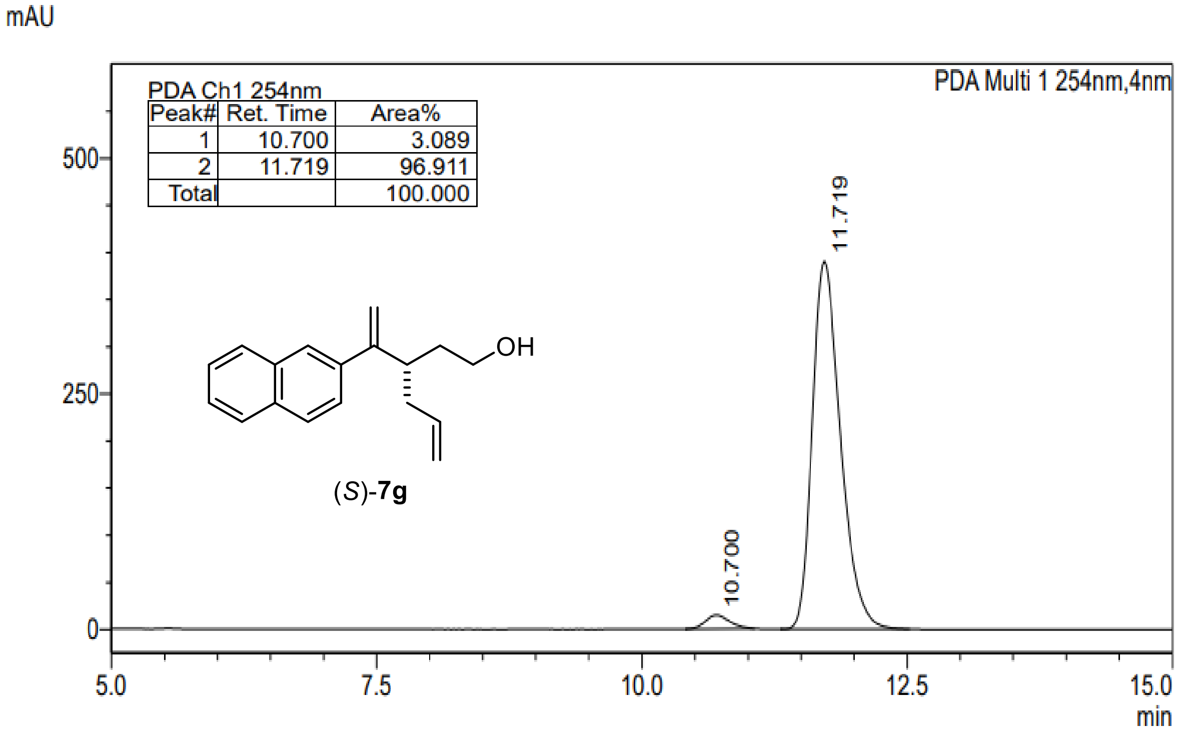

Peak#	Ret. Time	Area%
1	10.700	3.089
2	11.719	96.911
Total		100.000

(*S*)-3-(1-(dibenzo[b,d]furan-2-yl)vinyl)hex-5-en-1-ol (7h)

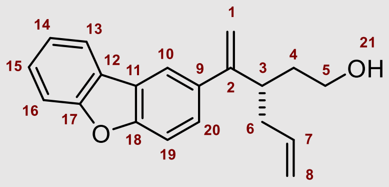

Following **GP-II** using boronate **1h** (0.3 mmol, 1.0 equiv.) and allyl acetate **2a** (0.36 mmol, 1.2 equiv.). The reaction was conducted at –20 °C for 48 h to afford compound **7h** as a yellow oil in 83% yield (73 mg) after chromatography on silica gel (eluent: pentane/EtOAc, from 10:1 to 7.5:1).

TLC: 0.3, pentane/EtOAc = 5:1

^{1}H NMR (400 MHz, $CDCl_3$) δ (ppm) = 7.98 – 7.94 (m, 1H, *H13*), 7.93 (dd, $^4J_{H\text{-}H}$ = 1.8, 0.5 Hz, 1H, *H10*), 7.57 (dt, $^3J_{H\text{-}H}$ = 8.3, $^4J_{H\text{-}H}$ = 0.7 Hz, 1H, *H16*), 7.51 (dd, $^3J_{H\text{-}H}$ = 8.5, $^4J_{H\text{-}H}$ = 0.5 Hz, 1H, *H19*), 7.49 – 7.43 (m, 2H, *H15 and H20*), 7.35 (td, $^3J_{H\text{-}H}$ = 7.5, $^4J_{H\text{-}H}$ = 0.5 Hz, 1H, *H14*), 5.89 – 5.77 (m, 1H, *H7*), 5.37 (d, $^4J_{H\text{-}H}$ = 0.9 Hz, 1H, *H1*), 5.14 (t, $^4J_{H\text{-}H}$ = 0.8 Hz, 1H, *H1*), 5.08 – 5.01 (m, 2H, *H8*), 3.81 – 3.68 (m, 2H, *H5*), 2.99 – 2.90 (m, 1H, *H3*), 2.42 – 2.33 (m, 1H, *H6*), 2.30 – 2.21 (m, 1H, *H6*), 1.93 – 1.76 (m, 2H, *H4*), 1.24 (t, $^3J_{H\text{-}H}$ = 5.2 Hz, 1H, *H21*).

^{13}C{^{1}H} NMR (101 MHz, $CDCl_3$) δ (ppm) = 156.7 (*C17*), 155.8 (*C18*), 152.2 (*C2*), 138.2 (*C9*), 136.7 (*C7*), 127.4 (*C15*), 126.4 (*C20*), 124.3 (*C11 and C12*), 122.9 (*C14*), 120.8 (*C13*), 118.9 (*C10*), 116.6 (*C8*), 113.1 (*C1*), 111.9 (*C16*), 111.4 (*C19*), 61.1 (*C5*), 41.0 (*C3*), 39.3 (*C6*), 36.9 (*C4*).

HRMS (ESI +**)**: calculated for $C_{20}H_{21}O_2$ $[M+H]^+$: 293.1537; found: 293.1520.

IR (neat) ν (cm^{-1}) = 3337, 3075, 2929, 1639, 1477, 1449, 1196, 1022, 909, 841, 818, 747.

HPLC: 97:3 *er*, IC, 1 mL/min, hexane:*i*PrOH = 99:1, λ = 254 nm, 30 °C, t_R (major) = 34.9 min, t_R (minor) = 39.2 min.

$[\alpha]^{20}_D$: +7.2° (*c* = 0.5, $CHCl_3$).

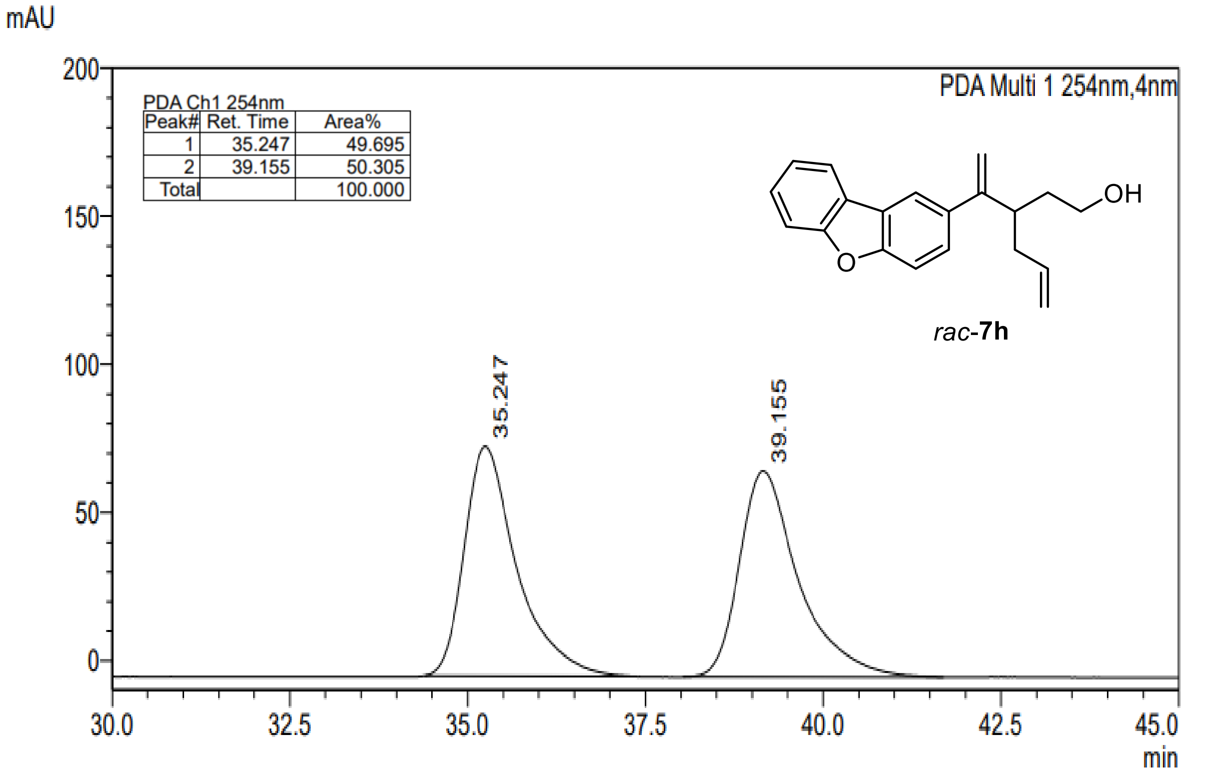
mAU
PDA Ch1 254nm
Peak# Ret. Time Area%
1 35.247 49.695
2 39.155 50.305
Total 100.000
PDA Multi 1 254nm,4nm
OH
O
rac-7h
35.247
39.155
200
150
100
50
0
30.0
32.5
35.0
37.5
40.0
42.5
45.0
min

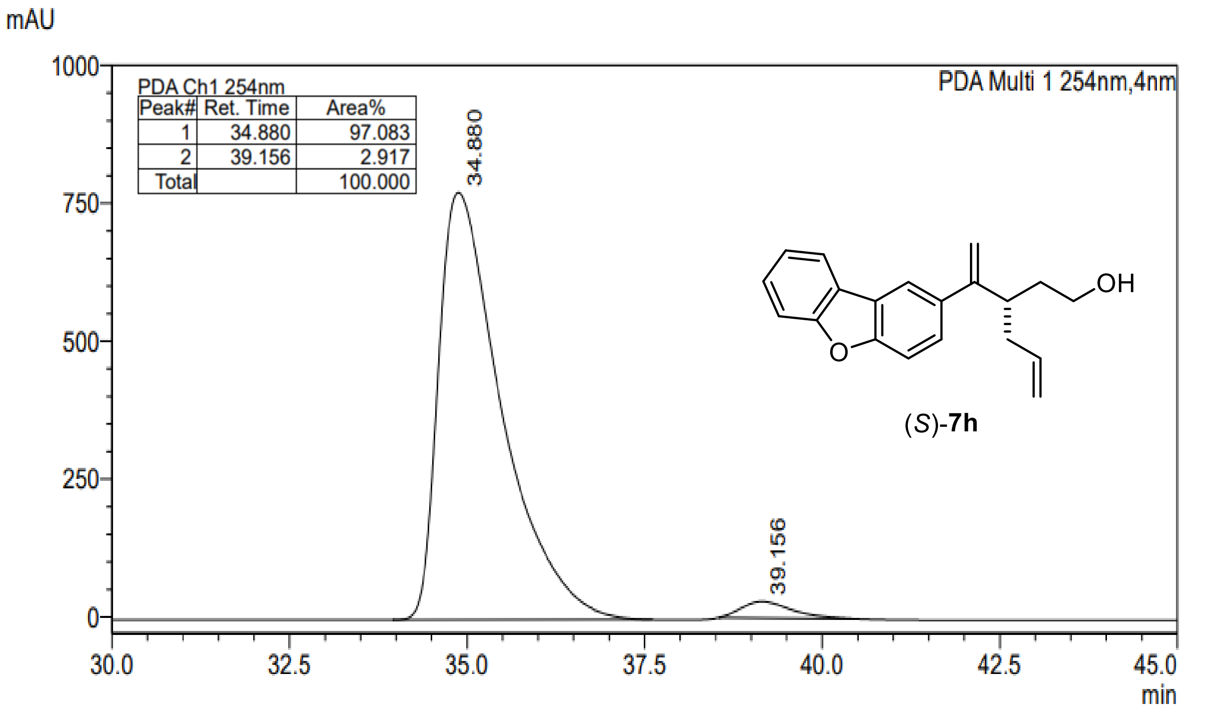
mAU
PDA Ch1 254nm
Peak# Ret. Time Area%
1 34.880 97.083
2 39.156 2.917
Total 100.000
PDA Multi 1 254nm,4nm
34.880
OH
O
(S)-7h
39.156
1000
750
500
250
0
30.0
32.5
35.0
37.5
40.0
42.5
45.0
min

(*S*)-3-(1-(4-(trifluoromethyl)phenyl)vinyl)hex-5-en-1-ol (7i)

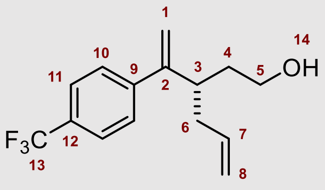

7i

Following **GP-I** using boronate **1i** (0.3 mmol, 1.0 equiv.) and allyl acetate **2a** (0.36 mmol, 1.2 equiv.), compound **7i** was prepared as a yellow oil (68 mg, 84% yield) after chromatography on silica gel (eluent: pentane/EtOAc, from 10:1 to 5:1).

TLC: 0.3, pentane/EtOAc = 5:1

^{1}H NMR (400 MHz, $CDCl_3$) δ (ppm) = 7.57 (d, $^3J_{H\text{-}H}$ = 8.1, 2H, *H11*), 7.46 (d, $^3J_{H\text{-}H}$ = 8.1, 2H, *H10*), 5.81 – 5.69 (m, 1H, *H7*), 5.35 (d, $^4J_{H\text{-}H}$ = 0.5 Hz, 1H, *H1*), 5.16 (bs, 1H, *H1*), 5.05 – 5.02 (m, 1H, *H8*), 5.00 (t, $^4J_{H\text{-}H}$ = 1.3 Hz, 1H, *H8*), 3.77 – 3.62 (m, 2H, *H5*), 2.89 – 2.90 (m, 1H, *H3*), 2.34 – 2.16 (m, 2H, *H6*), 1.88 – 1.72 (m, 2H, *H4*), 1.22 (t, $^3J_{H\text{-}H}$ = 4.3 Hz, 1H, *H14*).

^{13}C{^{1}H} NMR (101 MHz, $CDCl_3$) δ (ppm) = 151.2 (*C2*), 146.7 (*C9*), 136.3 (*C7*), 129.5 (q, $J_{C\text{-}F}$ = 32.2 Hz, *C12*), 127.2 (*C10*), 125.4 (q, $J_{C\text{-}F}$ = 3.8 Hz, *C11*), 124.4 (q, $J_{C\text{-}F}$ = 271.2 Hz, *C13*), 116.8 (*C8*), 114.7 (*C1*), 60.9 (*C5*), 40.4 (*C3*), 39.2 (*C6*), 36.8 (*C4*).

^{19}F{^{1}H} NMR (282 MHz, 298 K, $CDCl_3$) δ (ppm) = –62.48.

HRMS (ESI +**)**: calculated for $C_{15}H_{18}F_3O$ $[M+H]^+$: 271.1305; found: 271.1316.

IR (neat) ν (cm^{-1}) = 3329, 3079, 2933, 1616, 1442, 1322, 1163, 1115, 1064, 1015, 910, 847.

HPLC: 90:10 *er*, OD, 1 mL/min, hexane:*i*PrOH = 95:5, λ = 254 nm, 30 °C, t_R (major) = 7.1 min, t_R (minor) = 6.5 min.

$[\alpha]^{20}_D$: –1.2° (*c* = 0.5, $CHCl_3$).

PDA Ch1 254nm

Peak#	Ret. Time	Area%
1	6.512	49.870
2	7.096	50.130
Total		100.000

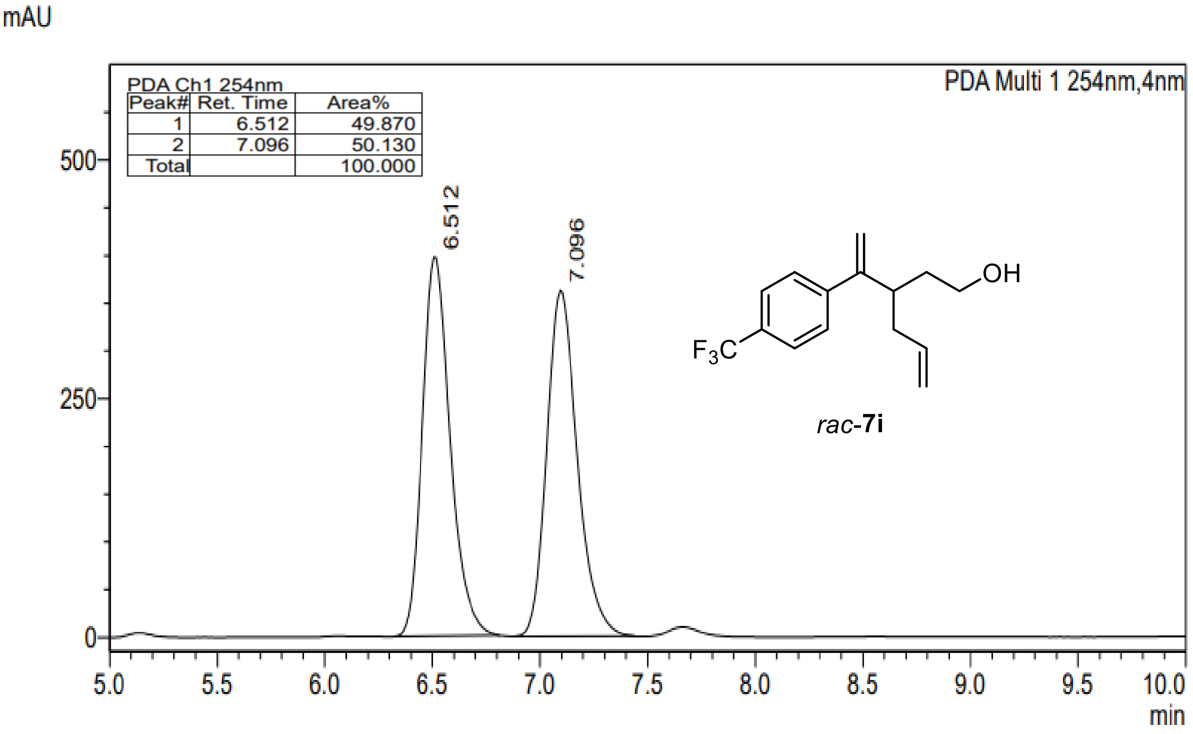

rac-**7i**

PDA Ch1 254nm

Peak#	Ret. Time	Area%
1	6.487	10.056
2	7.051	89.944
Total		100.000

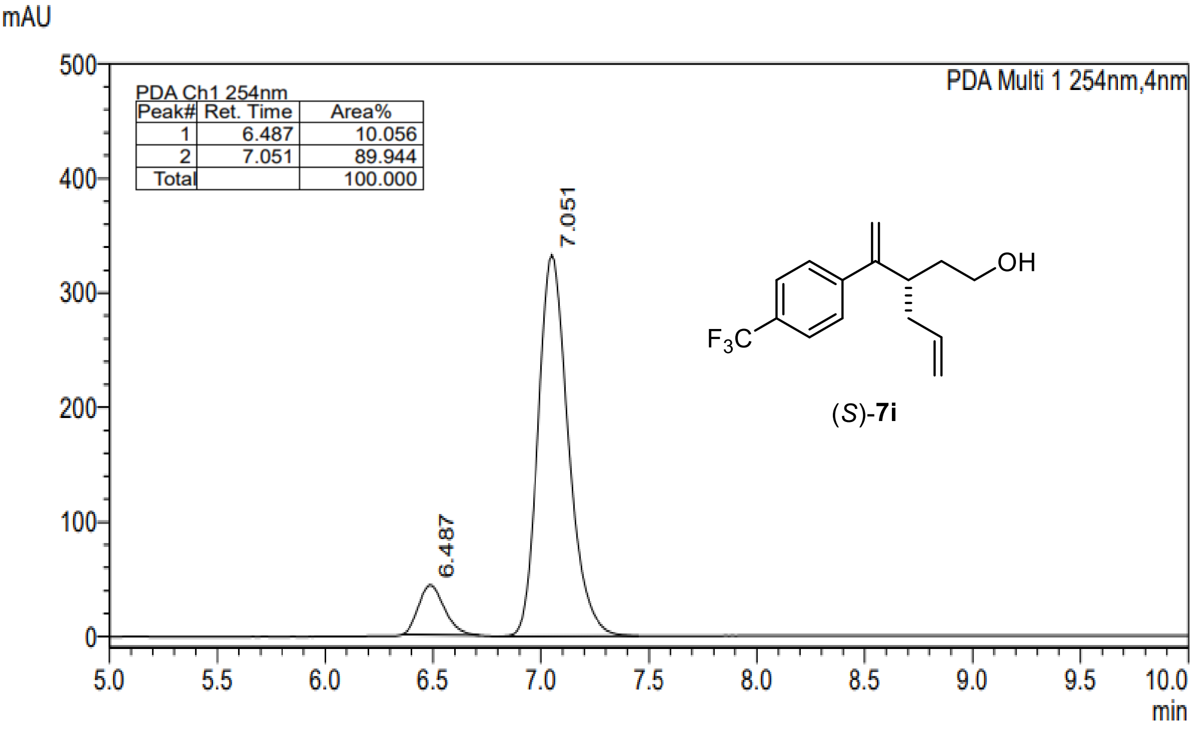

(*S*)-**7i**

(*S*)-3-(1-phenylvinyl)-5-(4-methoxylphenyl)hex-5-en-1-ol (7j)

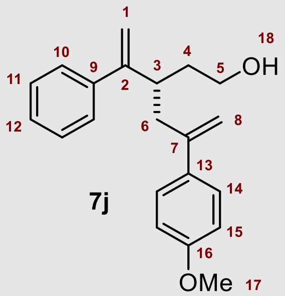

Following **GP-II** using boronate **1a** (0.3 mmol, 1.0 equiv.) and 2-(4-methoxylphenyl)allyl acetate **2e** (0.36 mmol, 1.2 equiv.). The reaction was conducted at –20 °C for 48 h to afford compound **7j** as a pale yellow oil in 43% yield (40 mg) after chromatography on silica gel (eluent: pentane/EtOAc, from 10:1 to 6:1).

TLC: 0.2, pentane/EtOAc = 5:1

^{1}H NMR (400 MHz, $CDCl_3$) δ (ppm) = 7.30 – 7.21 (m, 7H, *H10, H11, H12 and H14*), 7.22 – 7.17 (m, 2H, *H15*), 7.13 – 7.08 (m, 2H, *H15*), 5.29 (d, $^4J_{H\text{-}H}$ = 0.9 Hz, 1H, *H1*), 5.20 (d, $^4J_{H\text{-}H}$ = 1.6 Hz, 1H, *H8*), 5.08 (bs, 1H, *H1*), 4.98 (m, 1H, *H8*), 3.82 (s, 3H, *H17*), 3.69 – 3.60 (m, 1H, *H5*), 3.60 – 3.51 (m, 1H, *H5*), 2.86 – 2.75 (m, 2H, *H3 and H6*), 2.61 – 2.52 (m, 1H, *H6*), 1.89 – 1.79 (m, 1H, *H4*), 1.79 – 1.68 (m, 1H, *H4*), 1.07 (t, $^3J_{H\text{-}H}$ = 5.4 Hz, 1H, *H18*).

^{13}C{^{1}H} NMR (101 MHz, $CDCl_3$) δ (ppm) = 159.2 (*C16*), 152.6 (*C2*), 146.2 (*C13*), 142.9 (*C9*), 133.4 (*C7*), 128.3 (*C11*), 127.6 (*C14*), 127.4 (*C12*), 127.0 (*C10*), 113.8 (*C15*), 113.3 (*C8*), 113.0 (*C1*), 61.2 (*C5*), 55.4 (*C17*), 41.8 (*C6*), 39.1 (*C3*), 36.9 (*C4*).

HRMS (ESI +**)**: calculated for $C_{21}H_{24}O_2Na$ $[M+Na]^+$: 331.1669; found: 331.1667.

IR (neat) ν (cm^{-1}) = 3360, 2932, 1606, 1510, 1246, 1178, 1031, 894, 1031, 894, 834, 778, 701.

HPLC: 90:10 *er*, OJ, 1 mL/min, hexane:*i*PrOH = 90:10, λ = 254 nm, 30 °C, t_R (major) = 18.9 min, t_R (minor) = 13.0 min.

$[\alpha]^{20}_D$: –15.4° (*c* = 0.5, $CHCl_3$).

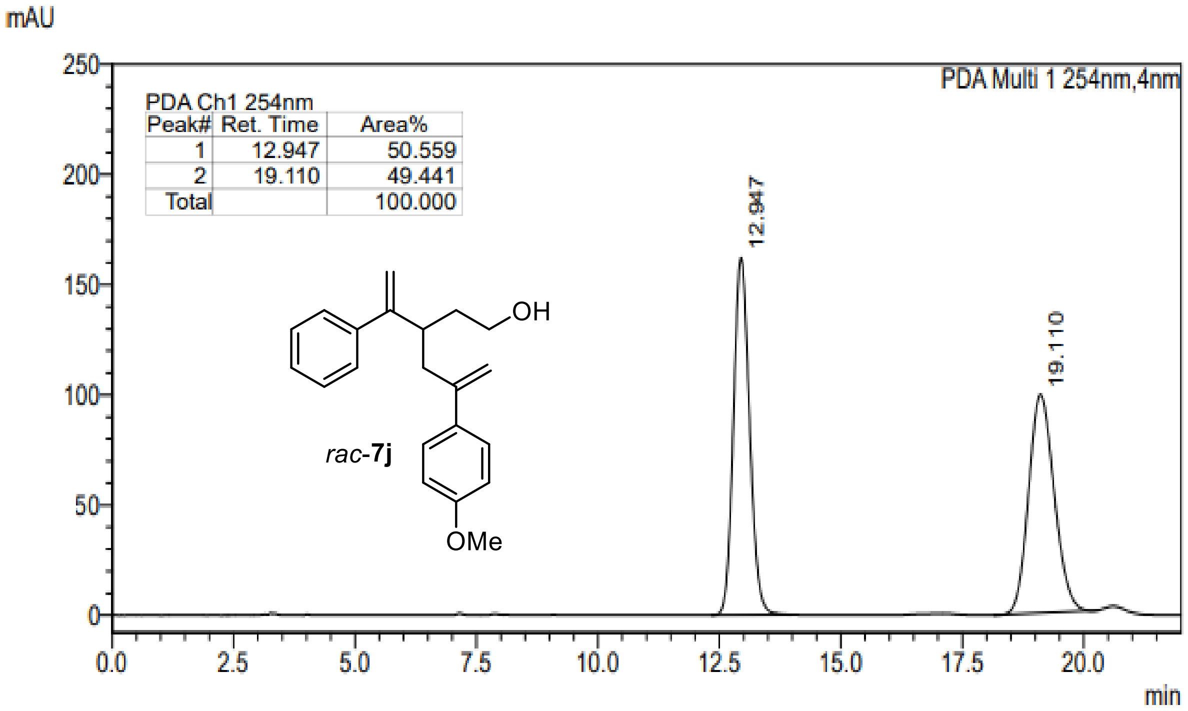
mAU
PDA Multi 1 254nm,4nm
PDA Ch1 254nm
Peak# Ret. Time Area%
1 12.947 50.559
2 19.110 49.441
Total 100.000
12.947
19.110
OH
OMe
rac-7j
min

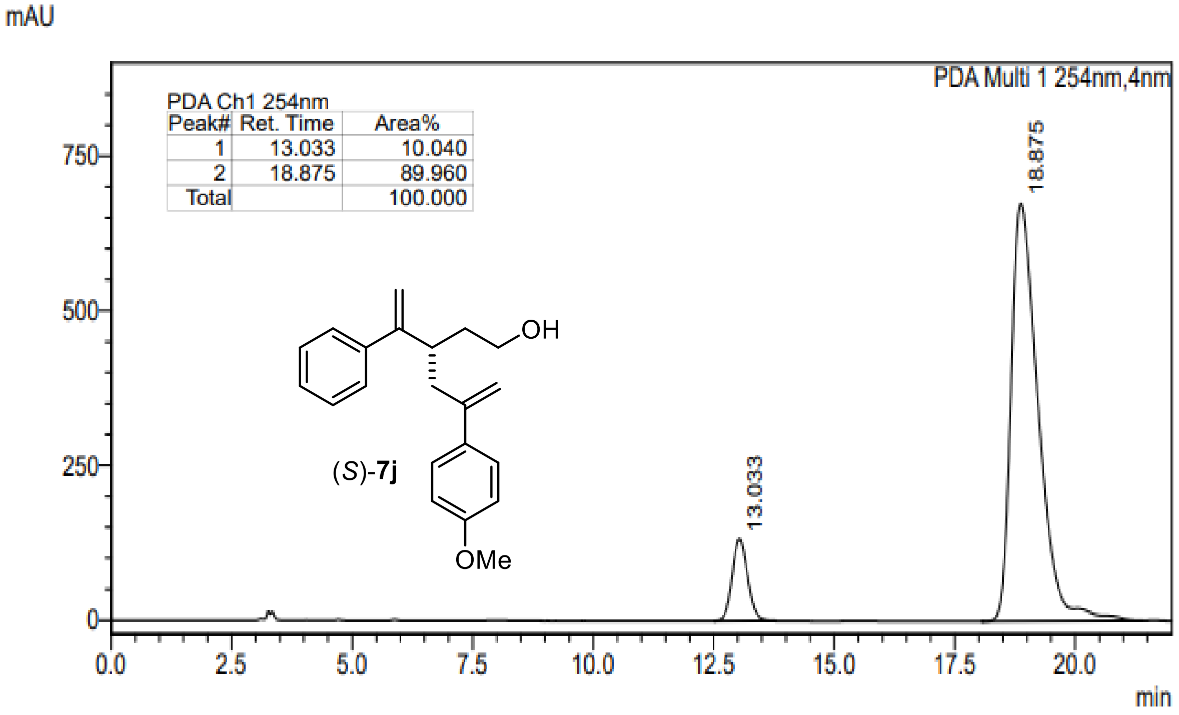
mAU
PDA Multi 1 254nm,4nm
PDA Ch1 254nm
Peak# Ret. Time Area%
1 13.033 10.040
2 18.875 89.960
Total 100.000
18.875
13.033
OH
OMe
(S)-7j
min

(*S*)-3-(1-phenylvinyl)-5-(4-methylphenyl)hex-5-en-1-ol (7k)

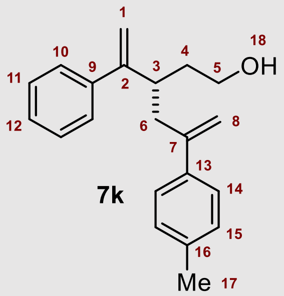

Following **GP-II** using boronate **1a** (0.3 mmol, 1.0 equiv.) and 2-(4-methylphenyl)allyl acetate **2f** (0.36 mmol, 1.2 equiv.). The reaction was conducted at –20 °C for 48 h to afford compound **7k** as a pale yellow oil in 38% yield (33 mg) after chromatography on silica gel (eluent: pentane/EtOAc, from 10:1 to 6:1).

TLC: 0.25, pentane/EtOAc = 6:1

^{1}H NMR (400 MHz, $CDCl_3$) δ (ppm) = 7.31 – 7.21 (m, 5H, *H10, H11 and H12*), 7.22 – 7.17 (m, 2H, *H14*), 7.13 – 7.08 (m, 2H, *H15*), 5.28 (d, $^4J_{H\text{-}H}$ = 1.0 Hz, 1H, *H1*), 5.24 (d, $^4J_{H\text{-}H}$ = 1.6 Hz, 1H, *H8*), 5.08 (bs, 1H, *H1*), 5.02 (m, 1H, *H8*), 3.67 – 3.60 (m, 1H, *H5*), 3.58 – 3.50 (m, 1H, *H5*), 2.86 – 2.77 (m, 2H, *H3 and H6*), 2.61 – 2.53 (m, 1H, *H6*), 2.35 (s, 3H, *H17*), 1.88 – 1.79 (m, 1H, *H4*), 1.78 – 1.69 (m, 1H, *H4*), 1.11 (bs, 1H, *H18*).

^{13}C{^{1}H} NMR (101 MHz, $CDCl_3$) δ (ppm) = 152.6 (*C2*), 146.7 (*C13*), 142.8 (*C9*), 138.1 (*C7*), 137.3 (*C16*), 129.2 (*C15*), 128.3 (*C11*), 127.4 (*C12*), 127.0 (*C10*), 126.4 (*C14*), 114.0 (*C8*), 113.0 (*C1*), 61.2 (*C5*), 41.7 (*C6*), 39.1 (*C3*), 36.9 (*C4*), 21.3 (*C17*).

HRMS (ESI +**)**: calculated for $C_{21}H_{23}$ $[M\text{–}OH]^+$: 275.1795; found: 275.1799.

IR (neat) ν (cm^{-1}) = 3233, 3052, 2934, 1624, 1512, 1442, 1045, 1028, 894, 824, 777, 701.

HPLC: 90.5:9.5 *er*, OJ, 1 mL/min, hexane:*i*PrOH = 90.5:9.5, λ = 254 nm, 30 °C, t_R (major) = 9.2 min, t_R (minor) = 7.8 min.

$[\alpha]^{20}_D$: –5.7° (*c* = 0.5, $CHCl_3$).

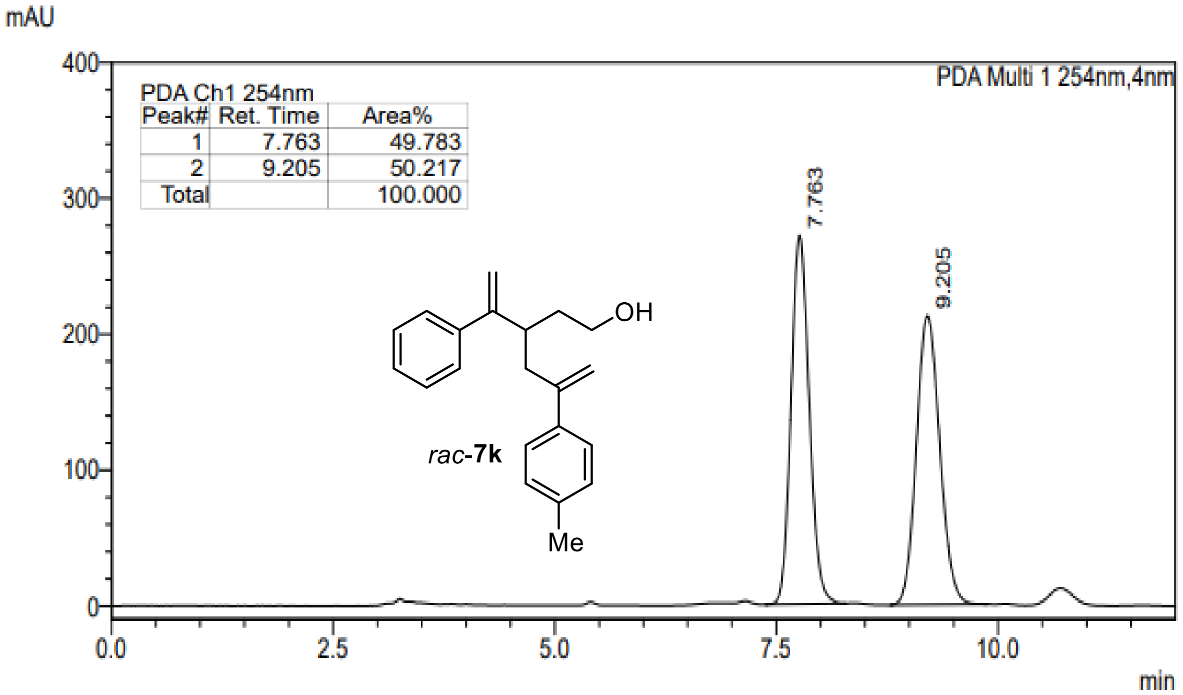

PDA Ch1 254nm

Peak#	Ret. Time	Area%
1	7.763	49.783
2	9.205	50.217
Total		100.000

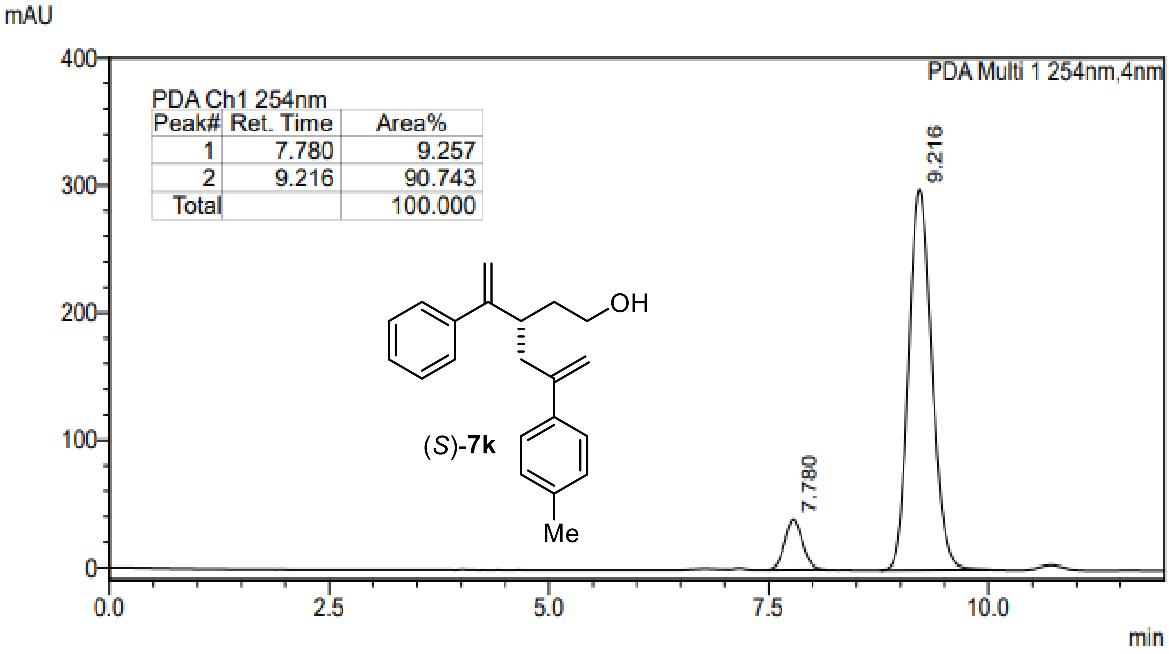

PDA Ch1 254nm

Peak#	Ret. Time	Area%
1	7.780	9.257
2	9.216	90.743
Total		100.000

(*S*)-5-phenyl-3-(1-phenylvinyl)hex-5-en-1-ol (7l)

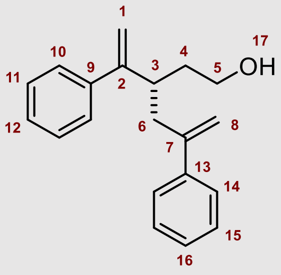

7l

Following **GP-II** using boronate **1a** (0.3 mmol, 1.0 equiv.) and 2-phenylallyl acetate **2d** (0.36 mmol, 1.2 equiv.). The reaction was conducted at –20 °C for 48 h to afford compound **7l** as a yellow oil in 73% yield (61 mg) after chromatography on silica gel (eluent: pentane/EtOAc, from 10:1 to 6:1).

TLC: 0.3, pentane/EtOAc = 5:1

^{1}H NMR (400 MHz, $CDCl_3$) δ (ppm) = 7.28 – 7.17 (m, 10H, *H10, H11, H12, H14, H15 and H16*), 5.26 (d, $^{4}J_{H\text{-}H}$ = 1.0 Hz, 1H, *H1*), 5.24 (d, $^{4}J_{H\text{-}H}$ = 1.6 Hz, 1H, *H8*), 5.05 (m, 1H, *H1*), 5.03 (m, 1H, *H8*), 3.66 – 3.57 (m, 1H, *H5*), 3.57 – 3.48 (m, 1H, *H5*), 2.83 – 2.75 (m, 2H, *H3 and H6*), 2.62 – 2.54 (m, 1H, *H6*), 1.86 – 1.76 (m, 1H, *H4*), 1.76 – 1.66 (m, 1H, *H4*), 1.04 (t, $^{3}J_{H\text{-}H}$ = 5.5 Hz, 1H, *H18*).

^{13}C{^{1}H} NMR (101 MHz, $CDCl_3$) δ (ppm) = 152.5 (*C2*), 147.0 (*C13*), 142.8 (*C9*), 141.1 (*C7*), 137.1 (*C16*), 128.5 (*C11 or C15*), 128.3 (*C11 or C15*), 127.6 (*C12 or C16*), 127.4 (*C12 or C16*), 127.0 (*C10*), 126.5 (*C14*), 114.8 (*C8*), 113.1 (*C1*), 61.2 (*C5*), 41.7 (*C6*), 39.1 (*C3*), 37.0 (*C4*).

HRMS (ESI +**)**: calculated for $C_{20}H_{23}O$ $[M+H]^{+}$: 279.1744; found: 279.1724.

IR (neat) ν (cm^{-1}) = 3337, 3079, 2933, 1626, 1493, 1442, 1047, 1028, 895, 776, 697.

HPLC: 95:5 *er*, AD, 1 mL/min, hexane:*i*PrOH = 99:1, λ = 237 nm, 30 °C, t_R (major) = 33.6 min, t_R (minor) = 37.2 min.

$[\alpha]^{20}_{D}$: +1.6° (*c* = 0.5, $CHCl_3$).

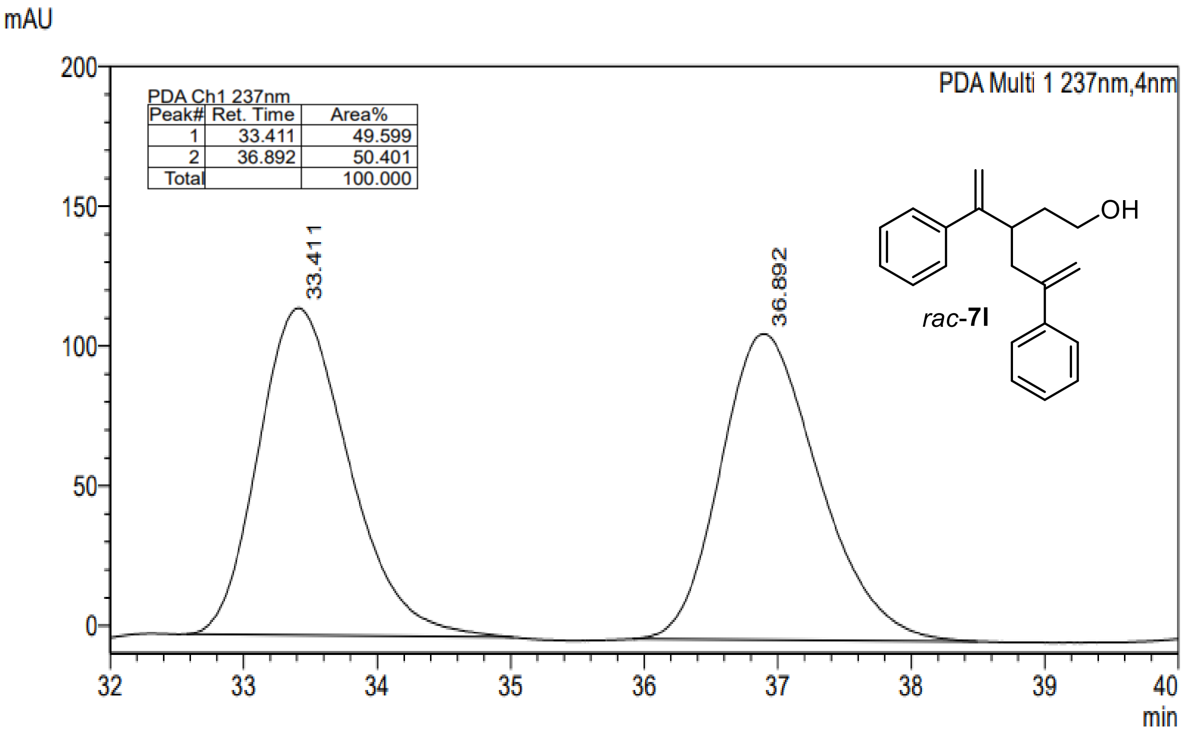

PDA Ch1 237nm

Peak#	Ret. Time	Area%
1	33.411	49.599
2	36.892	50.401
Total		100.000

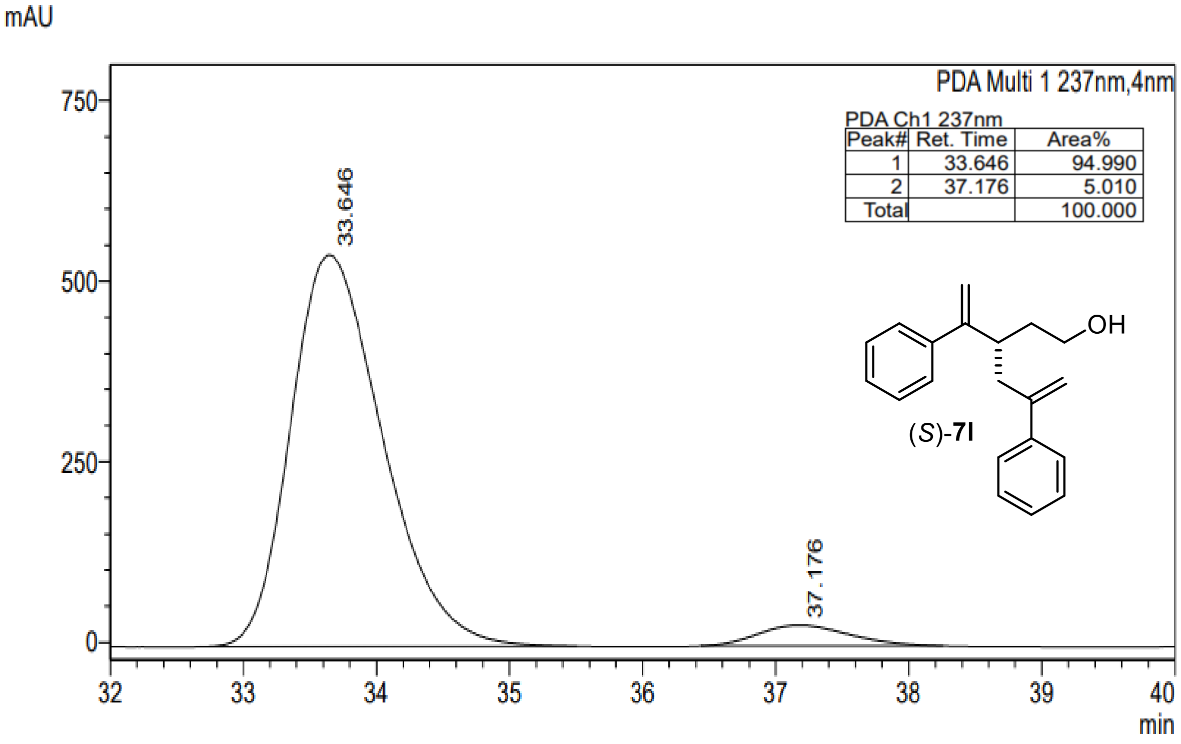

PDA Ch1 237nm

Peak#	Ret. Time	Area%
1	33.646	94.990
2	37.176	5.010
Total		100.000

(*S*)-5-(4-chlorophenyl)-3-(1-phenylvinyl)hex-5-en-1-ol (7m)

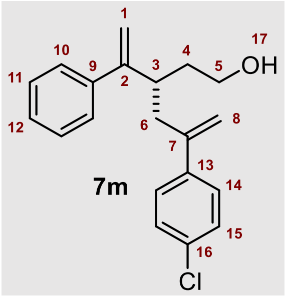

Following **GP-II** using boronate **1a** (0.3 mmol, 1.0 equiv.) and 2-(4-chlorophenyl)allyl acetate **2j** (0.36 mmol, 1.2 equiv.). The reaction was conducted at –20 °C for 48 h to afford compound **7m** as a yellow oil in 48% yield (45 mg) after chromatography on silica gel (eluent: pentane/EtOAc, from 10:1 to 7.5:1).

TLC: 0.35, pentane/EtOAc = 7.5:1

^{1}H NMR (400 MHz, $CDCl_3$) δ (ppm) = 7.30 – 7.16 (m, 9H, *H10, H11*, *H12, H14 and H15*), 5.29 (d, $^4J_{H\text{-}H}$ = 0.8 Hz, 1H, *H1*), 5.24 (d, $^4J_{H\text{-}H}$ = 1.4 Hz, 1H, *H8*), 5.08 – 5.06 (m, 1H, *H1* and *H8*), 3.70 – 3.61 (m, 1H, *H5*), 3.61 – 3.51 (m, 1H, *H5*), 2.84 – 2.72 (m, 2H, *H3 and H6*), 2.63 – 2.54 (m, 1H, *H6*), 1.87 – 1.69 (m, 2H, *H4*), 1.18 (bs, 1H, *H18*).

^{13}C{^{1}H} NMR (101 MHz, $CDCl_3$) δ (ppm) = 152.2 (*C2*), 145.8 (*C13*), 142.7 (*C9*), 139.5 (*C7*), 133.3 (*C16*), 128.6 (*C11 or C15*), 128.4 (*C11 or C15*), 127.8 (*C14*), 127.5 (*C12*), 126.9 (*C10*), 115.3 (*C8*), 113.1 (*C1*), 61.1 (*C5*), 41.7 (*C6*), 39.0 (*C3*), 36.9 (*C4*).

HRMS (ESI +**)**: calculated for $C_{20}H_{20}Cl$ $[M–OH]^+$: 295.1249; found: 295.1243.

IR (neat) ν (cm^{-1}) = 3333, 3080, 2934, 1625, 1491, 1028, 1012, 898, 834, 777, 764, 700.

HPLC: 96.5:3.5 *er*, OJ, 1 mL/min, hexane:*i*PrOH = 90:10, λ = 254 nm, 30 °C, t_R (major) = 9.4 min, t_R (minor) = 7.4 min.

$[\alpha]^{20}_D$: –18.4° (*c* = 0.5, $CHCl_3$).

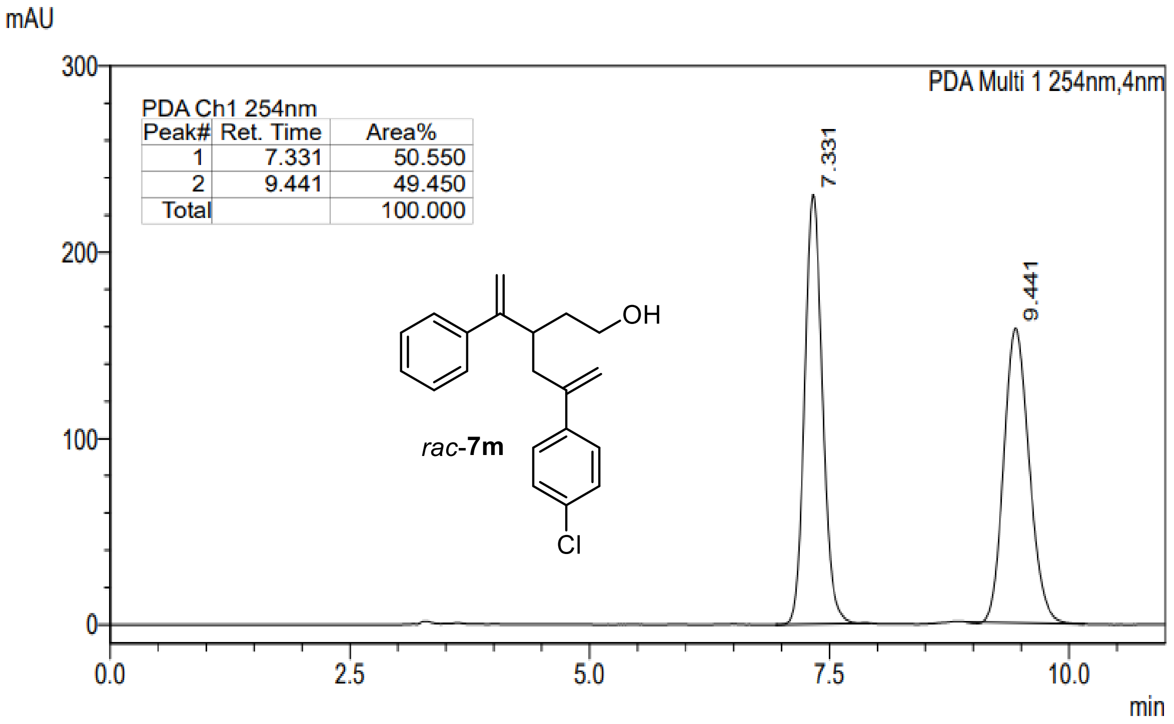

PDA Ch1 254nm

Peak#	Ret. Time	Area%
1	7.331	50.550
2	9.441	49.450
Total		100.000

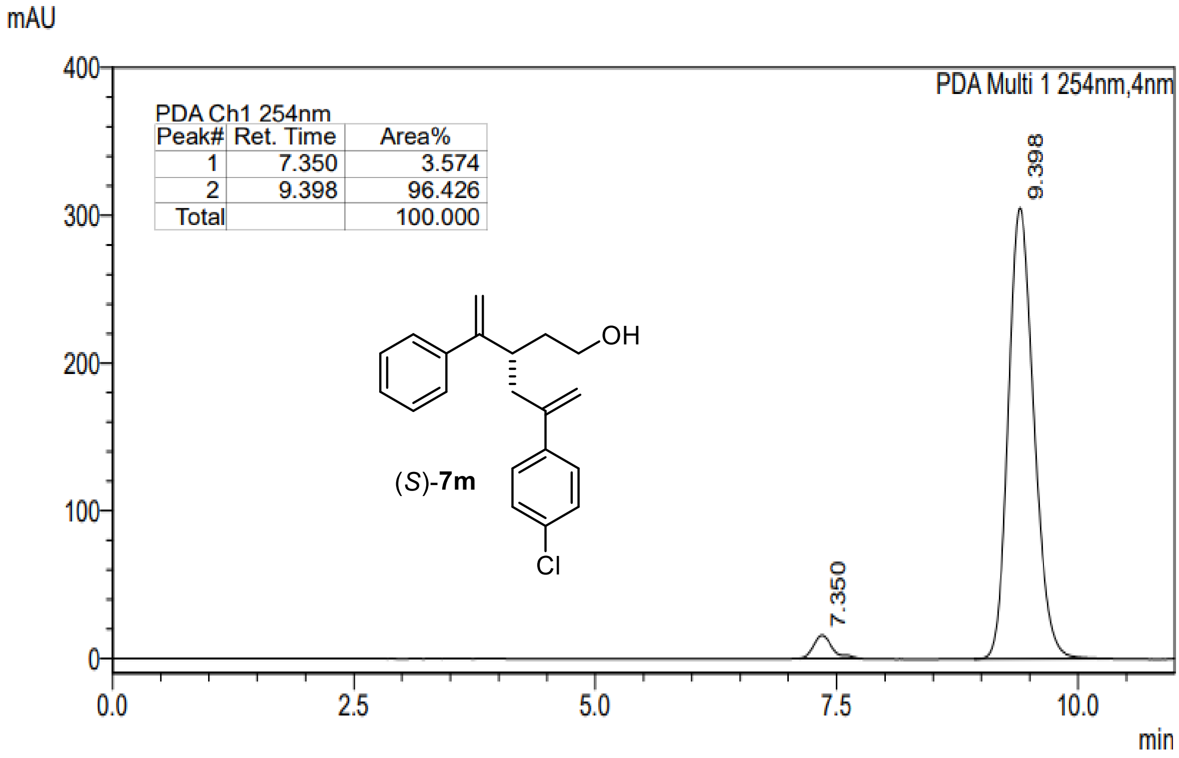

PDA Ch1 254nm

Peak#	Ret. Time	Area%
1	7.350	3.574
2	9.398	96.426
Total		100.000

(*S*)-3-(1-phenylvinyl)-5-(2-naphthyl)hex-5-en-1-ol (7n)

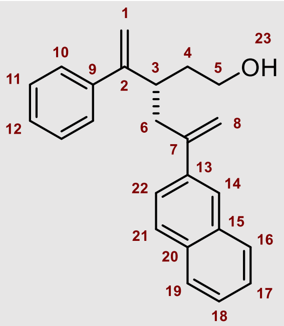

7n

Following **GP-II** using boronate **1a** (0.3 mmol, 1.0 equiv.) and 2-(2-naphthyl)allyl acetate **2h** (0.36 mmol, 1.2 equiv.). The reaction was conducted at –20 °C for 48 h to afford compound **7n** as a yellow oil in 74% yield (73 mg) after chromatography on silica gel (eluent: pentane/EtOAc, from 10:1 to 6:1).

TLC: 0.3, pentane/EtOAc = 6:1

^{1}H NMR (400 MHz, $CDCl_3$) δ (ppm) = 7.85 – 7.80 (m, 1H, *H19*), 7.78 (d, $^3J_{H\text{-}H}$ = 8.5 Hz, 1H, *H21*), 7.75 – 7.71 (m, 1H, *H16*), 7.66 (d, $^4J_{H\text{-}H}$ = 1.6 Hz, 1H, *H14*), 7.51 – 7.43 (m, 3H, *H17, H18 and H22*), 7.30 – 7.22 (m, 5H, *H10, H11 and H12*), 5.42 (d, $^4J_{H\text{-}H}$ = 1.5 Hz, 1H, *H8*), 5.31 (d, $^4J_{H\text{-}H}$ = 0.9 Hz, 1H, *H1*), 5.18 (bs, 1H, *H8*), 5.12 (m, 1H, *H1*), 3.71 – 3.62 (m, 1H, *H5*), 3.60 – 3.51 (m, 1H, *H5*), 3.00 – 2.92 (m, 1H, *H6*), 2.91 – 2.83 (m, 1H, *H3*), 2.72 – 2.64 (m, 1H, *H6*), 1.96 – 1.85 (m, 1H, *H4*), 1.84 – 1.74 (m, 1H, *H4*), 1.09 (t, $^4J_{H\text{-}H}$ = 5.1 Hz, 1H, *H23*).

^{13}C{^{1}H} NMR (101 MHz, $CDCl_3$) δ (ppm) = 152.5 (*C2*), 146.7 (*C13*), 142.8 (*C9*), 138.2 (*C7*), 133.5 (*C20)*, 133.0 (*C15*), 128.4 (*C11*), 128.3 (*C16*), 128.0 (*C21*), 127.7 (*C19*), 127.5 (*C12*), 127.0 (*C10*), 126.2 (*C18*), 126.0 (*C17*), 125.2 (*C14*), 125.0 (*C22*), 115.4 (*C8*), 113.0 (*C1*), 61.0 (*C5*), 41.9 (*C6*), 39.2 (*C3*), 36.8 (*C4*).

HRMS (ESI +**)**: calculated for $C_{24}H_{23}$ $[M–OH]^+$: 311.1795; found: 311.1795.

IR (neat) ν (cm^{-1}) = 3353, 3080, 2934, 1624, 1491, 1046, 1028, 894, 834, 777, 751, 700.

HPLC: 95:5 *er*, AD, 1 mL/min, hexane:*i*PrOH = 95:5, λ = 254 nm, 30 °C, t_R (major) = 10.5 min, t_R (minor) = 12.1 min.

$[\alpha]^{20}_D$: –11.1° (*c* = 0.5, $CHCl_3$).

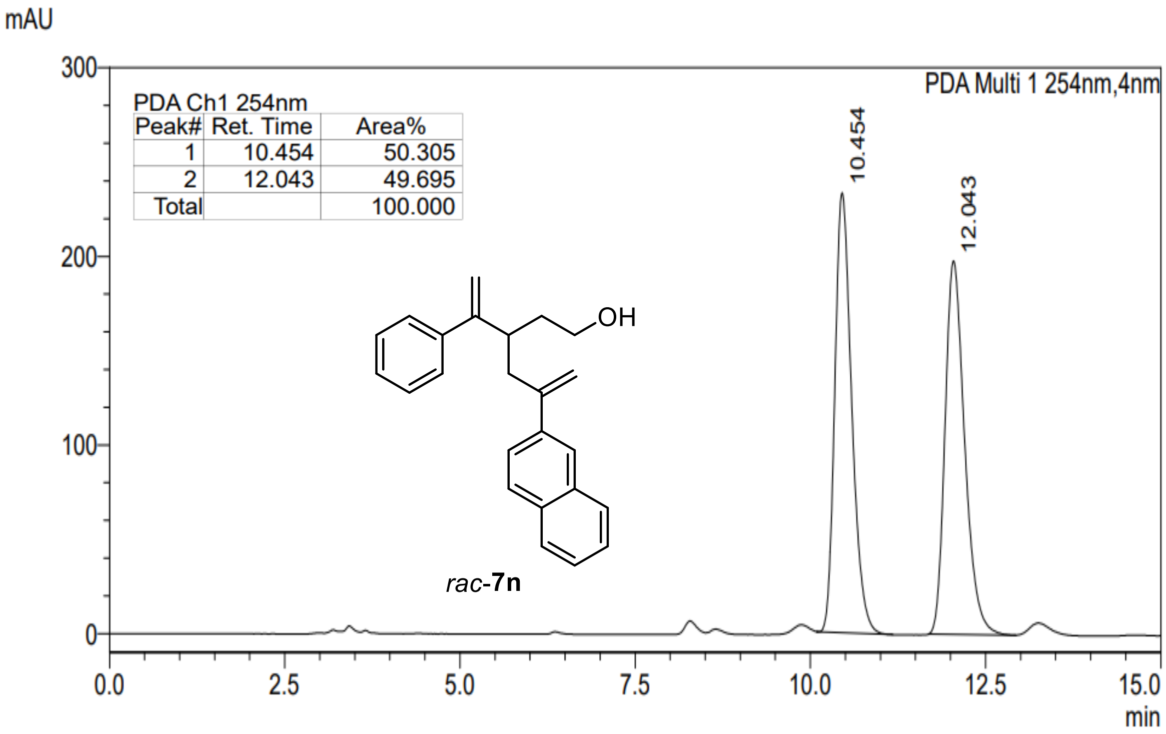

Peak#	Ret. Time	Area%
1	10.454	50.305
2	12.043	49.695
Total		100.000

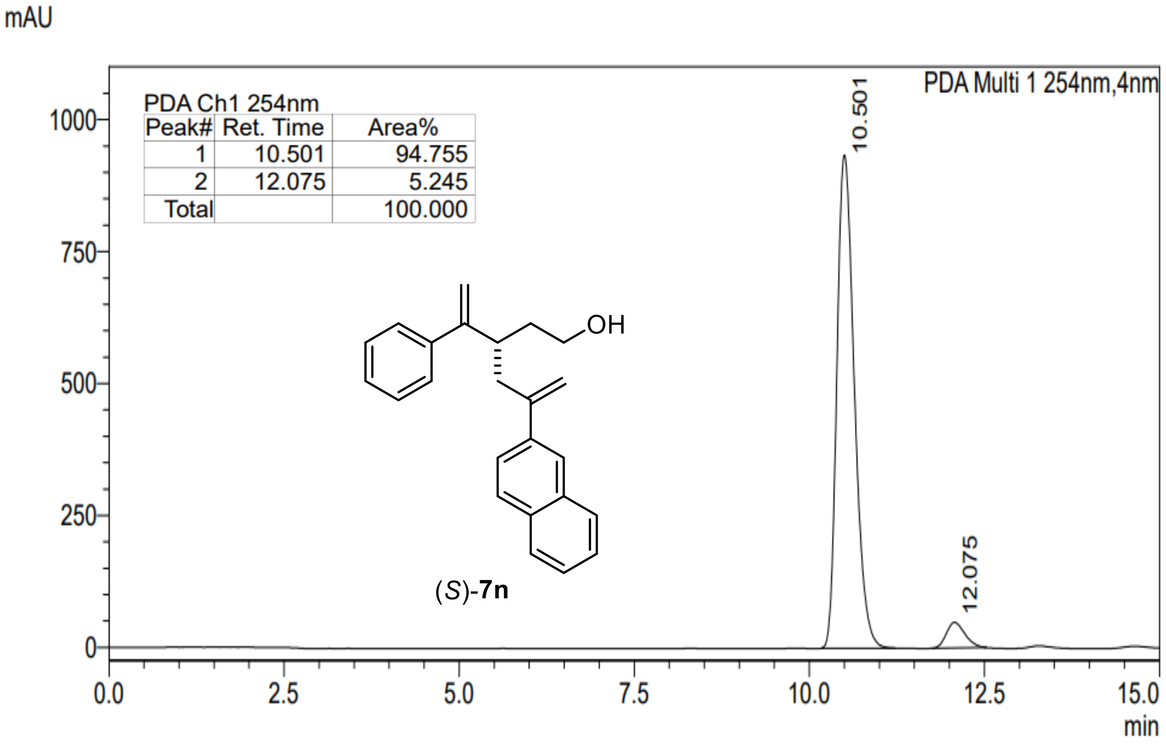

Peak#	Ret. Time	Area%
1	10.501	94.755
2	12.075	5.245
Total		100.000

(*S*)-3-(1-phenylvinyl)-5-(4-(trifluoromethyl)phenyl)hex-5-en-1-ol (7o)

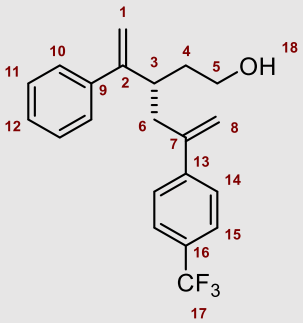

7o

Following **GP-II** using boronate **1a** (0.3 mmol, 1.0 equiv.) and 2-(4-(trifluoromethyl)phenyl)allyl acetate **2g** (0.36 mmol, 1.2 equiv.). The reaction was conducted at –20 °C for 48 h to afford compound **7o** as a yellow oil in 69% yield (72 mg) after chromatography on silica gel (eluent: pentane/EtOAc, from 10:1 to 6:1).

TLC: 0.25, pentane/EtOAc = 6:1

^{1}H NMR (400 MHz, $CDCl_3$) δ (ppm) = 7.53 (d, $^3J_{H-H,}$ = 8.1 Hz, 2H, *H15*), 7.35 (d, $^3J_{H-H,}$ = 8.1 Hz, 2H, *H14*), 7.28 – 7.24 (m, 3H*, H11 and H12*), 7.21 – 7.17 (m, 2H, *H10*), 5.31 (d, $^4J_{H-H}$ = 1.2 Hz, 1H, *H8*), 5.30 (d, $^4J_{H-H}$ = 0.9 Hz, 1H, *H1*), 5.16 (d, $^4J_{H-H}$ = 1.1 Hz, 1H, *H8*), 5.07 (s, 1H, *H1*), 3.72 – 3.63 (m, 1H, *H5*), 3.63 – 3.54 (m, 1H, *H5*), 2.85 – 2.75 (m, 2H, *H3 and H6*), 2.71 – 2.61 (m, 1H, *H6*), 1.90 – 1.72 (m, 2H, *H4*), 1.17 (bs, 1H, *H18*).

^{13}C{^{1}H} NMR (101 MHz, $CDCl_3$) δ (ppm) = 152.1 (*C2*), 145.9 (*C7*), 144.7 (*C13*), 142.7 (*C9*), 129.5 (q, J_{C-F} = 32.7 Hz, *C15*), 128.4 (*C11*), 127.3 (*C12*), 126.9 (*C10*), 126.8 (d, J_{C-F} = 3.8 Hz, *C14*), 125.4 (q, J_{C-F} = 3.8 Hz, *C16*), 124.3 (q, J_{C-F} = 271.7 Hz, *C17*), 116.7 (*C8*), 113.3 (*C1*), 61.0 (*C5*), 41.6 (*C6*), 39.1 (*C3*), 37.0 (*C4*).

^{19}F{^{1}H} NMR (282 MHz, 298 K, $CDCl_3$) δ (ppm) = –62.45.

HRMS (ESI +**)**: calculated for $C_{21}H_{20}F_3$ $[M–OH]^+$: 397.1386; found: 397.1393.

IR (neat) ν (cm^{-1}) = 3346, 3082, 2938, 1616, 1323, 1164, 1116, 1065, 1014, 901, 847, 701.

HPLC: 96:4 *er*, OD, 1 mL/min, hexane:*i*PrOH = 95:5, λ = 254 nm, 30 °C, t_R (major) = 8.9 min, t_R (minor) = 8.0 min.

$[\alpha]^{20}_D$: –4.8° (*c* = 0.5, $CHCl_3$).

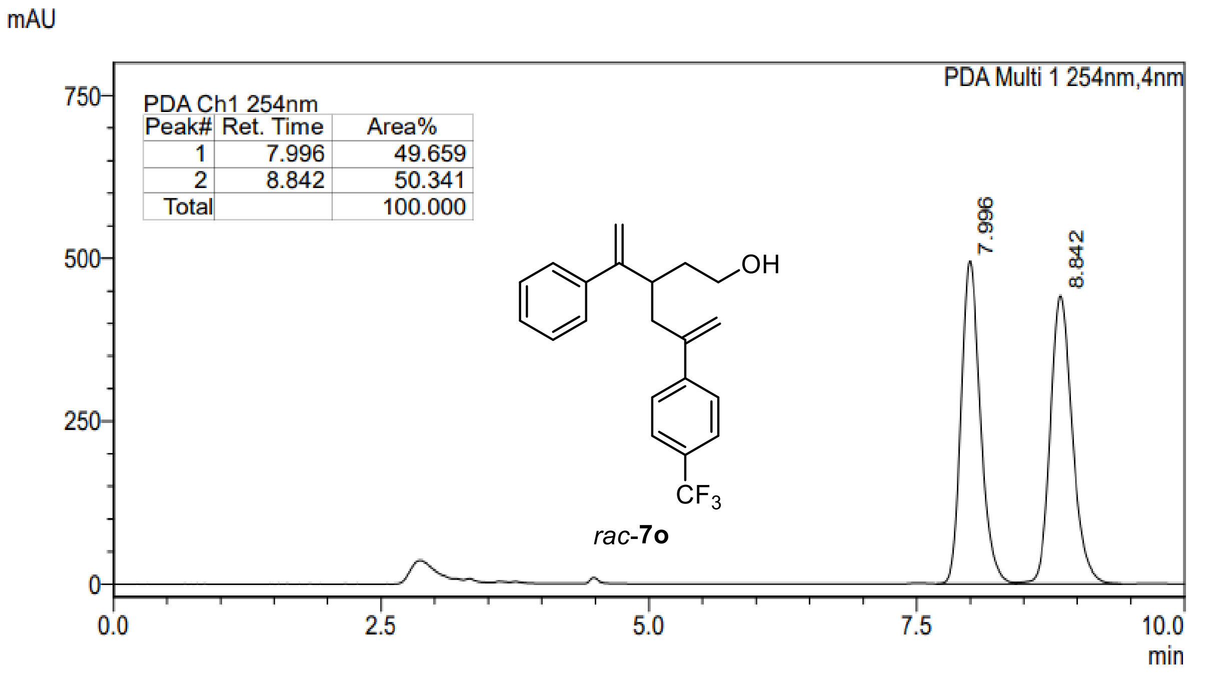

PDA Ch1 254nm

Peak#	Ret. Time	Area%
1	7.996	49.659
2	8.842	50.341
Total		100.000

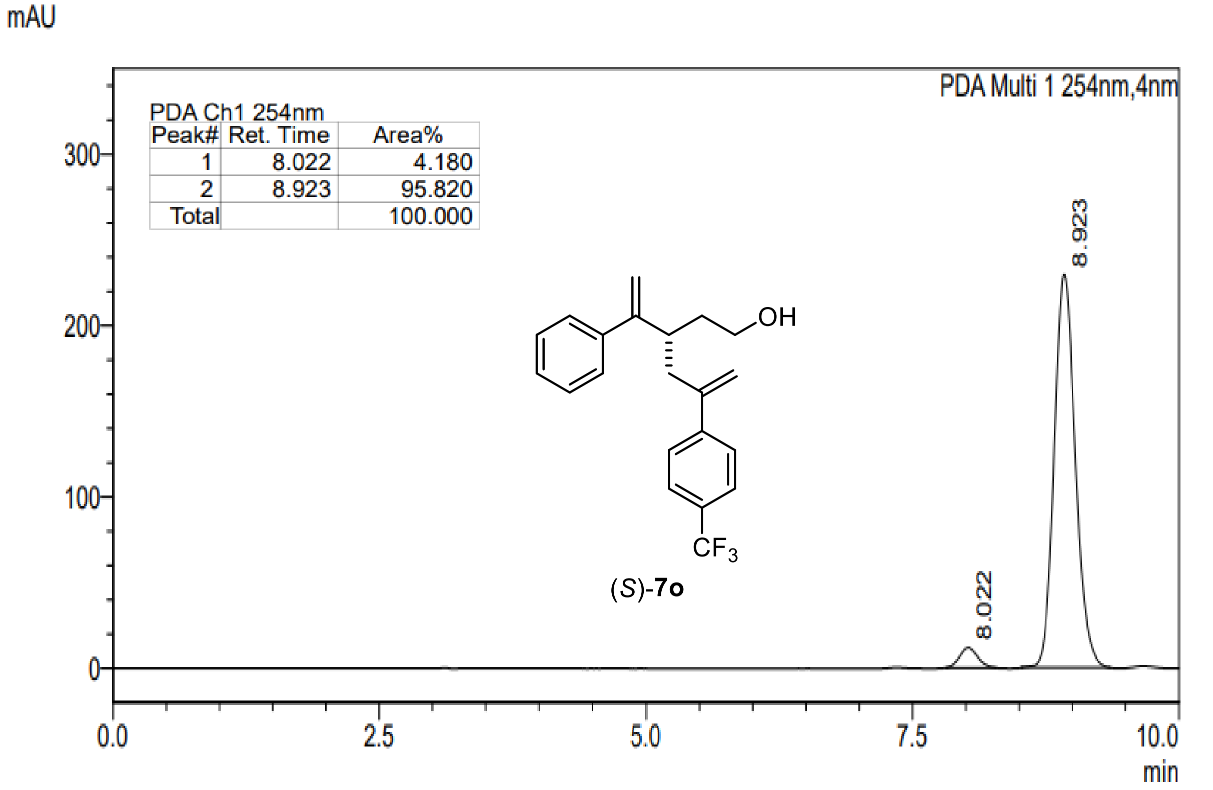

PDA Ch1 254nm

Peak#	Ret. Time	Area%
1	8.022	4.180
2	8.923	95.820
Total		100.000

(*S*)-5-(3,5-bis(trifluoromethyl)phenyl)-3-(1-phenylvinyl)hex-5-en-1-ol (7p)

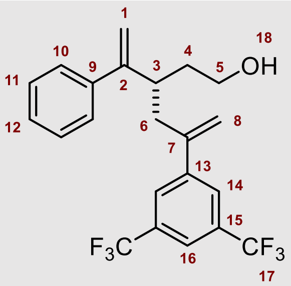

7p

Following **GP-II** using boronate **1a** (0.3 mmol, 1.0 equiv.) and 2-(3,5-bis(trifluoromethyl)phenyl)allyl acetate **2i** (0.36 mmol, 1.2 equiv.). The reaction was conducted at –20 °C for 48 h to afford compound **7p** as a yellow oil in 68% yield (85 mg) after chromatography on silica gel (eluent: pentane/EtOAc, from 10:1 to 6:1).

TLC: 0.4, pentane/EtOAc = 6:1

^{1}H NMR (400 MHz, $CDCl_3$) δ (ppm) = 7.74 (bs, 1H, *H16*), 7.62 (bs, 1H, *H14*), 7.28 – 7.23 (m, 3H*, H11 and H12*), 7.21 – 7.16 (m, 2H, *H10*), 5.34 (d, $^4J_{H\text{-}H}$ = 0.7 Hz, 1H, *H8*), 5.32 (d, $^4J_{H\text{-}H}$ = 0.7 Hz, 1H, *H1*), 5.24 (bs, 1H, *H8*), 5.08 (s, 1H, *H1*), 3.76 – 3.67 (m, 1H, *H5*), 3.67 – 3.57 (m, 1H, *H5*), 2.86 – 2.66 (m, 3H, *H3 and H6*), 1.92 – 1.77 (m, 2H, *H4*), 1.23 (bs, 1H, *H18*).

^{13}C{^{1}H} NMR (101 MHz, $CDCl_3$) δ (ppm) = 151.7 (*C2*), 144.4 (*C13*), 143.1 (*C7*), 142.3 (*C9*), 131.5 (q, $J_{C\text{-}F}$ = 33.0 Hz, *C15*), 128.2 (*C11*), 127.5 (*C12*), 126.5 (*C10*), 126.3 (d, $J_{C\text{-}F}$ = 3.8 Hz, *C14*), 123.3 (q, $J_{C\text{-}F}$ = 272.5 Hz, *C17*), 121.0 (p, $J_{C\text{-}F}$ = 3.8 Hz, *C16*), 117.8 (*C8*), 113.0 (*C1*), 60.7 (*C5*), 41.6 (*C6*), 38.7 (*C3*), 36.8 (*C4*).

^{19}F{^{1}H} NMR (282 MHz, 298 K, $CDCl_3$) δ (ppm) = –62.83.

HRMS (ESI +**)**: calculated for $C_{22}H_{19}F_6$ [M–OH]$^+$: 397.1386; found: 397.1393.

IR (neat) ν (cm^{-1}) = 3343, 3084, 2938, 1628, 1377, 1275, 1170, 1125, 1048, 896, 700, 682.

HPLC: 66:34 *er*, OD, 1 mL/min, hexane:*i*PrOH = 95:5, λ = 254 nm, 30 °C, t_R (major) = 6.9 min, t_R (minor) = 5.8 min.

[α]$^{20}_D$: –3.7° (*c* = 0.5, $CHCl_3$).

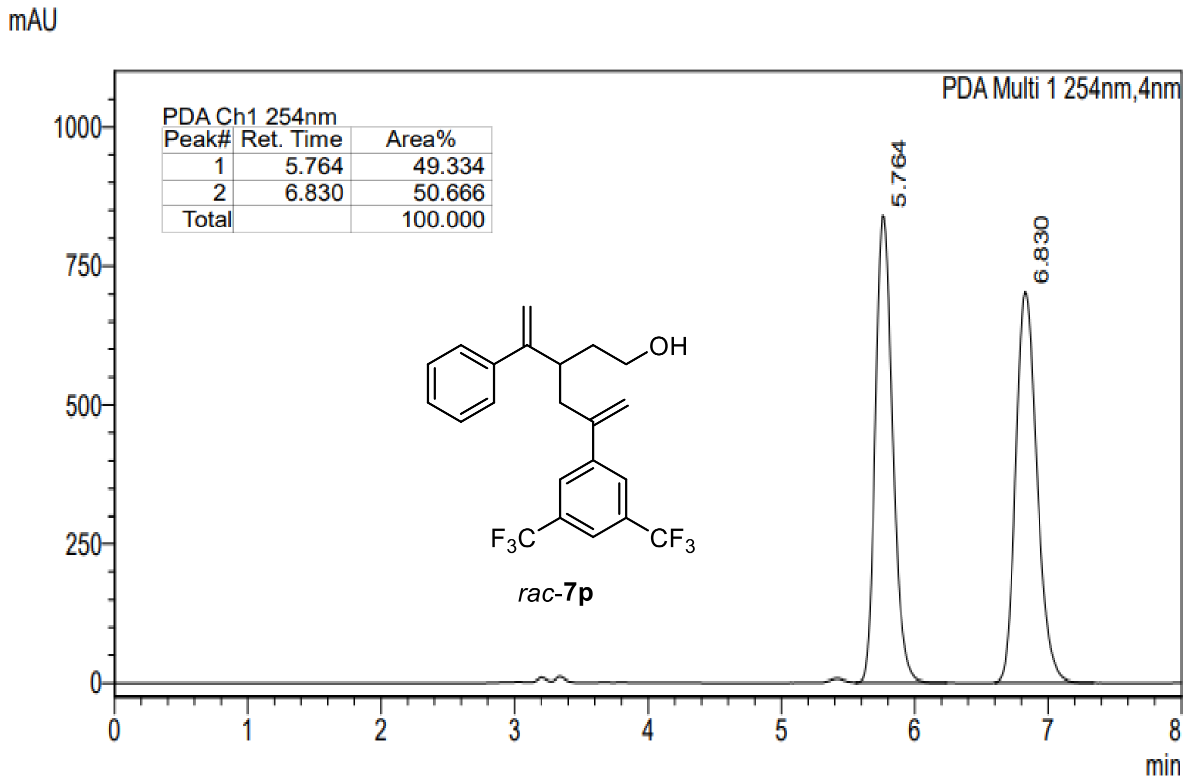
mAU
PDA Multi 1 254nm,4nm
PDA Ch1 254nm
Peak# | Ret. Time | Area%
1 | 5.764 | 49.334
2 | 6.830 | 50.666
Total | | 100.000
5.764
6.830
OH
F3C
CF3
rac-7p
1000
750
500
250
0
0 1 2 3 4 5 6 7 8
min

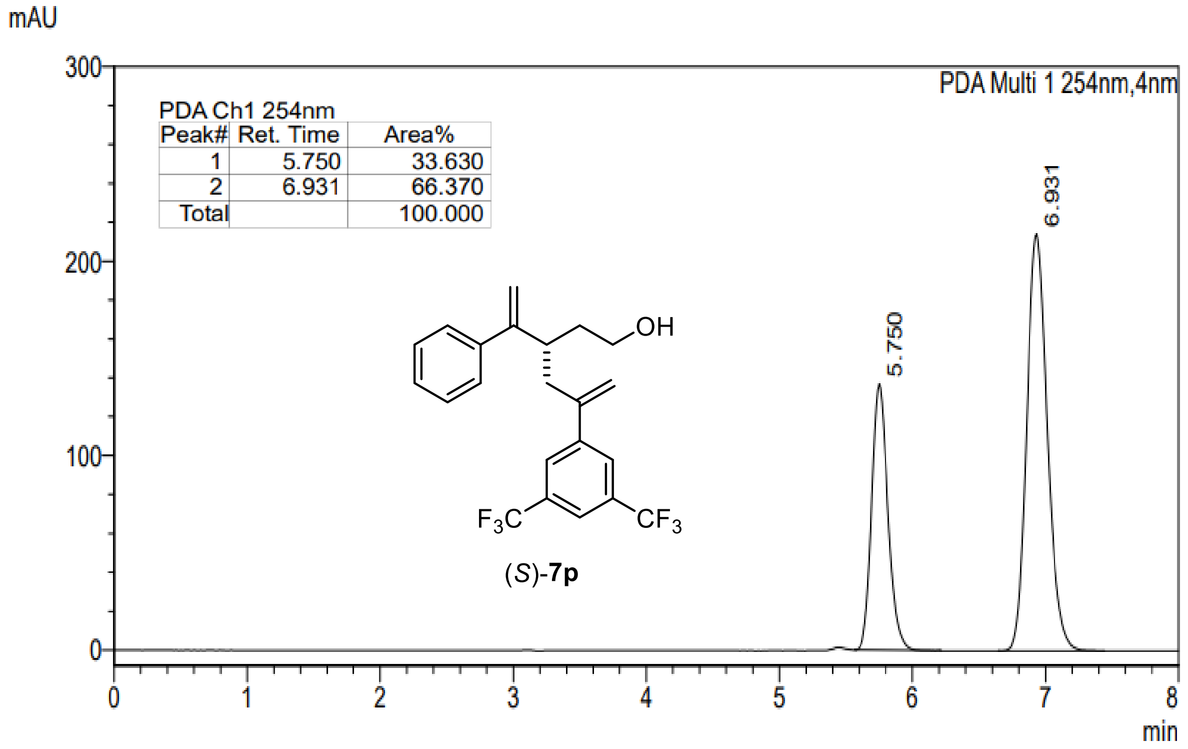
mAU
PDA Multi 1 254nm,4nm
PDA Ch1 254nm
Peak# | Ret. Time | Area%
1 | 5.750 | 33.630
2 | 6.931 | 66.370
Total | | 100.000
5.750
6.931
OH
F3C
CF3
(S)-7p
300
200
100
0
0 1 2 3 4 5 6 7 8
min

(*S*)-5-(1-methyl-1H-indol-2-yl)-3-(1-phenylvinyl)hex-5-en-1-ol (7q)

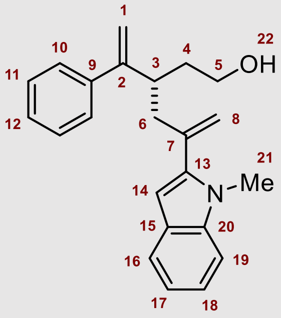

7q

Following **GP-II** using boronate **1a** (0.3 mmol, 1.0 equiv.) and 2-(1-methyl-1H-indol-2-yl)allyl acetate **2l** (0.36 mmol, 1.2 equiv.). The reaction was conducted at –20 °C for 48 h to afford compound **7q** as a yellow oil in 44% yield (44 mg) after chromatography on silica gel (eluent: pentane/EtOAc, from 10:1 to 4:1).

TLC: 0.3, pentane/EtOAc = 4:1

^{1}H NMR (400 MHz, $CDCl_3$) δ (ppm) = 7.57 (dt, $^3J_{H\text{-}H}$ = 7.8, $^4J_{H\text{-}H}$ = 0.8 Hz, *H16*), 7.30 – 7.15 (m, 7H, *H10, H11*, *H12, H18 and H19*), 7.11 (m, 1H, *H17*), 6.37 (d, $^4J_{H\text{-}H}$ = 0.6, *H14*), 5.35 (d, $^4J_{H\text{-}H}$ = 1.3 Hz, 1H, *H8*), 5.32 (d, $^4J_{H\text{-}H}$ = 0.9 Hz, 1H, *H1*), 5.14 (d, $^4J_{H\text{-}H}$ = 1.5 Hz, 1H, *H8*), 5.08 (bs, 1H, *H1*), 3.70 – 3.61 (m, 1H, *H5*), 3.60 – 3.51 (m, 4H, *H5 and H21*), 2.94 – 2.84 (m, 1H, *H3*), 2.76 (dd, $J_{H\text{-}H}$ = 14.2, 7.2 Hz, 1H, *H6*), 2.65 (dd, $J_{H\text{-}H}$ = 14.2, 7.2 Hz, 1H, *H6*),1.91 – 1.72 (m, 2H, *H4*), 1.18 (bs, 1H, *H22*).

^{13}C{^{1}H} NMR (101 MHz, $CDCl_3$) δ (ppm) = 152.1 (*C2*), 142.6 (*C9*), 141.0 (*C7*), 138.8 (*C13*), 138.6 (*C20*), 128.4 (*C11*), 127.8 (*C15*), 127.5 (*C12*), 126.8 (*C10*), 121.8 (*C19*), 120.6 (*C16*), 119.8 (*C17*), 118.2 (*C8*), 113.2 (*C1*), 109.6 (*C19*), 101.1 (*C14*), 61.1 (*C5*), 43.7 (*C6*), 39.3 (*C3*), 37.3 (*C4*), 31.2 (*C21*).

HRMS (ESI +**)**: calculated for $C_{23}H_{26}NO$ $[M+H]^+$: 332.2009; found: 332.1989.

IR (neat) ν (cm^{-1}) = 3371, 3053, 2934, 1625, 1464, 1310, 1045, 1028, 898, 778, 749, 734, 701.

HPLC: 94:6 *er*, AD, 1 mL/min, hexane:*i*PrOH = 95:5, λ = 234 nm, 30 °C, t_R (major) = 12.1 min, t_R (minor) = 13.3 min.

$[\alpha]^{20}_D$: –9.7° (*c* = 0.5, $CHCl_3$).

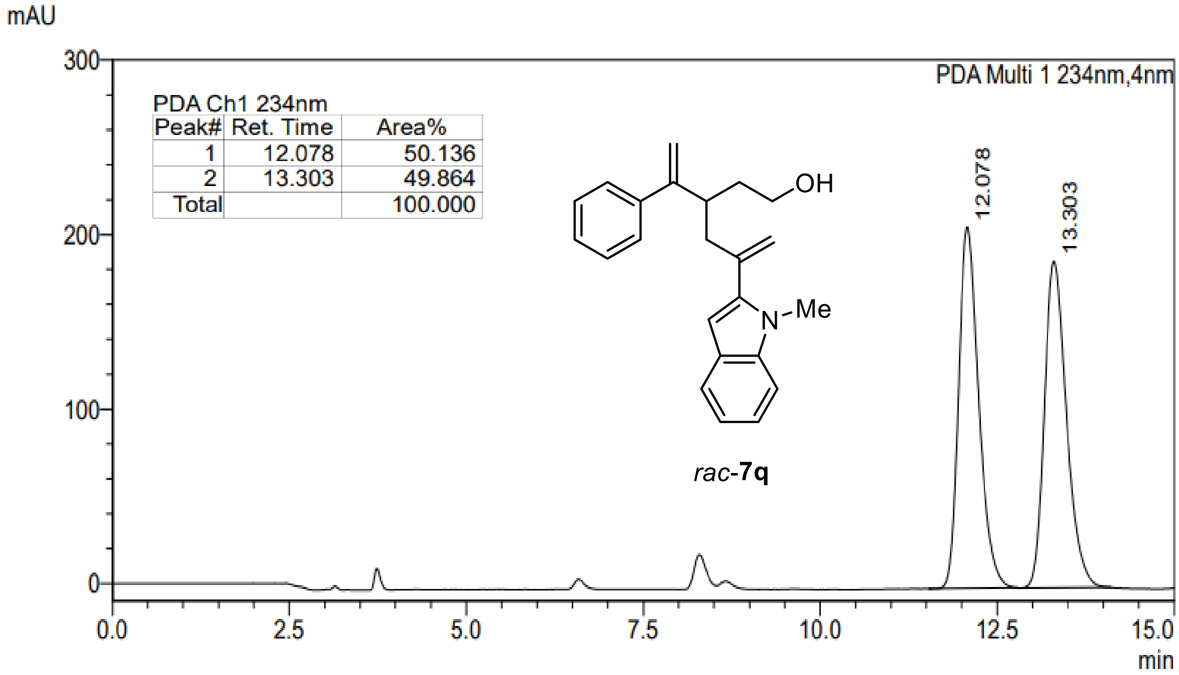
mAU
PDA Multi 1 234nm,4nm
PDA Ch1 234nm
Peak# Ret. Time Area%
1 12.078 50.136
2 13.303 49.864
Total 100.000
OH
N-Me
rac-**7q**
12.078
13.303
300
200
100
0
0.0
2.5
5.0
7.5
10.0
12.5
15.0
min

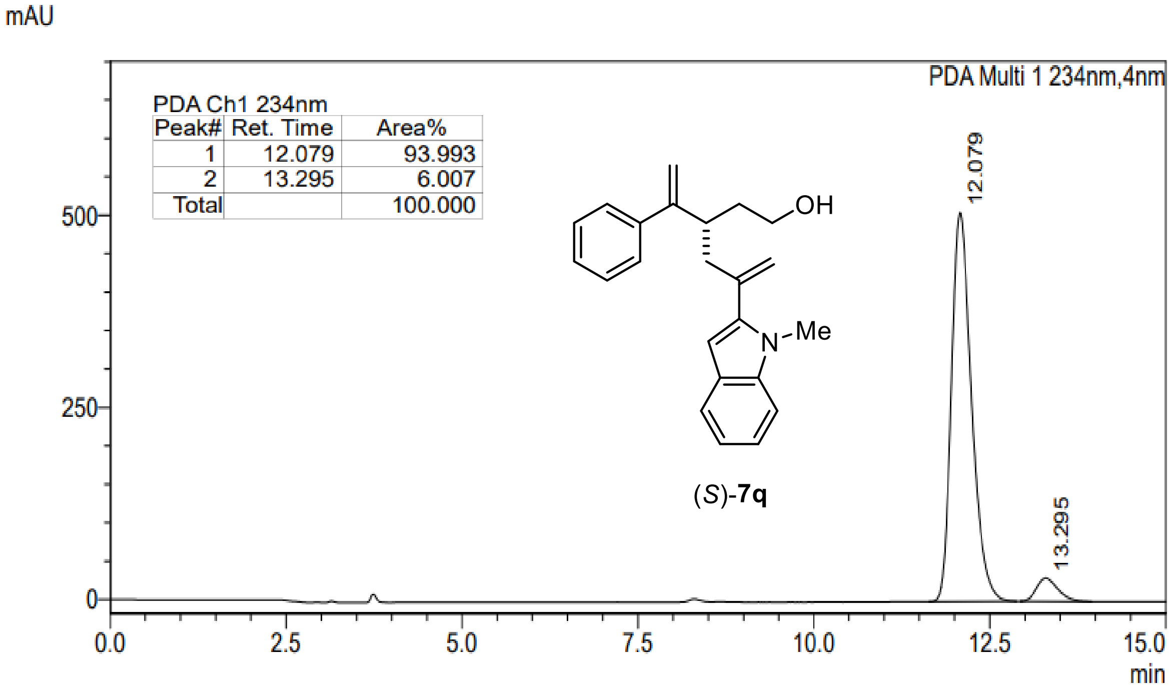
mAU
PDA Multi 1 234nm,4nm
PDA Ch1 234nm
Peak# Ret. Time Area%
1 12.079 93.993
2 13.295 6.007
Total 100.000
OH
N-Me
(*S*)-**7q**
12.079
13.295
500
250
0
0.0
2.5
5.0
7.5
10.0
12.5
15.0
min

(*S*)-5-(1-methyl-1H-indol-5-yl)-3-(1-phenylvinyl)hex-5-en-1-ol (7r)

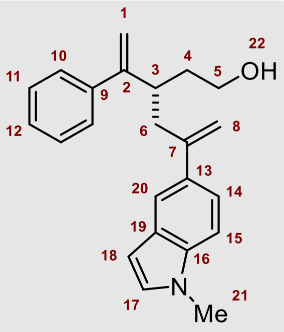

7r

Following **GP-II** using boronate **1a** (0.3 mmol, 1.0 equiv.) and 2-(1-methyl-1H-indol-5-yl)allyl acetate **2m** (0.36 mmol, 1.2 equiv.). The reaction was conducted at –20 °C for 48 h to afford compound **7r** as a yellow oil in 14% yield (14 mg) after chromatography on silica gel (eluent: pentane/EtOAc, from 10:1 to 4:1).

TLC: 0.2, pentane/EtOAc = 4:1

^{1}H NMR (400 MHz, $CDCl_3$) δ (ppm) = 7.53 (dd, $^3J_{H-H}$ = 1.7, 0.6 Hz, 1H, *H20*), 7.30 – 7.15 (m, 7H, *H10, H11*, *H12, H14 and H15*), 7.05 (d, $^3J_{H-H}$ = 3.1, 1H, *H17*), 6.44 (dd, $^3J_{H-H}$ = 3.1, $^4J_{H-H}$ = 0.4 Hz, 1H, *H18*), 5.29 (d, $^4J_{H-H}$ = 0.9 Hz, 1H, *H1*), 5.27 (d, $^4J_{H-H}$ = 1.8 Hz, 1H, *H8*), 5.11 (bs, 1H, *H1*), 5.04 (bs, 1H, *H8*), 3.80 (s, 3H, *H21*), 3.67 – 3.59 (m, 1H, *H5*), 3.56 – 3.48 (m, 4H, *H5 and H21*), 2.94 (ddd, J_{H-H} = 14.0, 5.6, 0.8 Hz, 1H, *H6*), 2.89 – 2.80 (m, 1H, *H3*), 2.61 (dd, J_{H-H} = 14.0, 8.4 Hz, 1H, *H6*), 1.92 – 1.81 (m, 1H, *H4*), 1.81 – 1.70 (m, 1H, *H4*), 0.88 (m, 1H, *H22*).

^{13}C{^{1}H} NMR (101 MHz, $CDCl_3$) δ (ppm) = 152.8 (*C2*), 147.8 (*C7*), 142.9 (*C9*), 136.5 (*C16*), 132.3 (*C13*), 129.3 (*C17*), 128.6 (*C19*), 128.3 (*C11*), 127.4 (*C12*), 127.1 (*C10*), 120.8 (*C14*), 118.8 (*C20*), 113.2 (*C8*), 112.8 (*C1*), 109.1 (*C15*), 101.4 (*C18*), 61.3 (*C5*), 42.3 (*C6*), 39.1 (*C3*), 36.7 (*C4*), 33.0 (*C21*).

HRMS (ESI +**)**: calculated for $C_{23}H_{26}NO$ $[M+H]^+$: 332.2009; found: 332.2026.

IR (neat) ν (cm^{-1}) = 3388, 3078, 2925, 1725, 1622, 1489, 1335, 1245, 1046, 883, 778, 703.

HPLC: 86.5:13.5 *er*, AD, 1 mL/min, hexane:*i*PrOH = 95:5, λ = 254 nm, 30 °C, t_R (major) = 15.9 min, t_R (minor) = 17.7 min.

[α]$^{20}_{D}$: –10.0° (*c* = 0.5, $CHCl_3$).

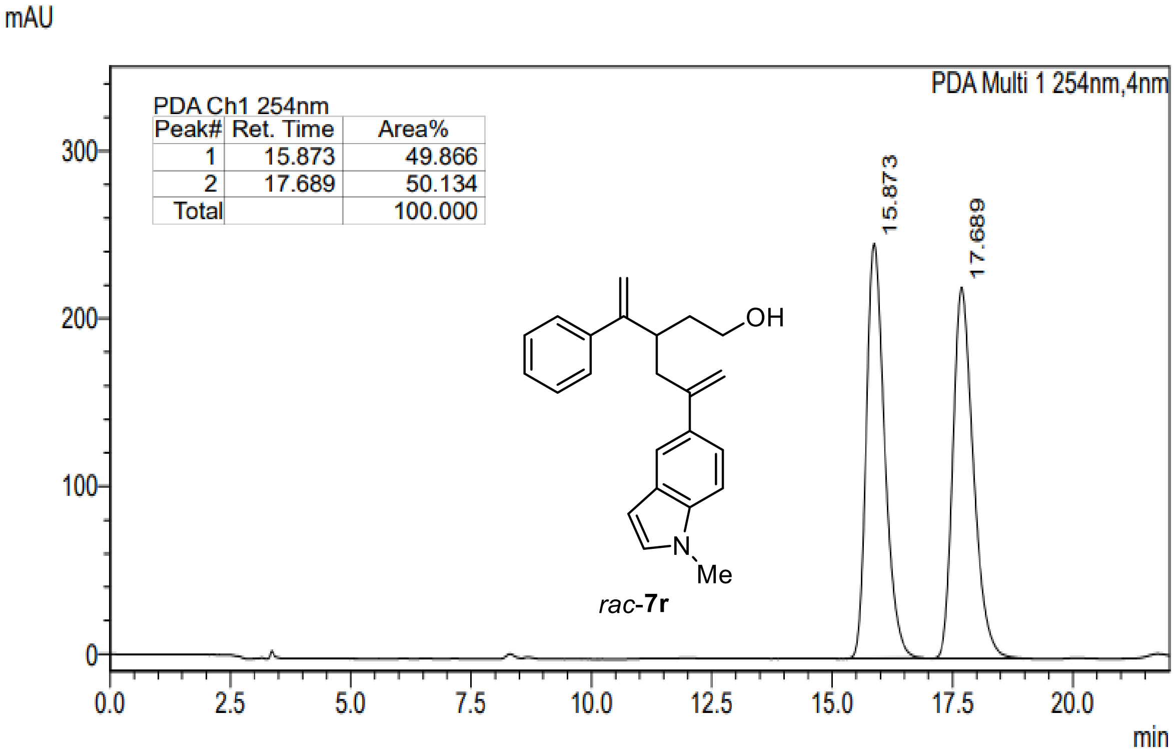
mAU
PDA Multi 1 254nm,4nm
PDA Ch1 254nm
Peak# Ret. Time Area%
1 15.873 49.866
2 17.689 50.134
Total 100.000
15.873
17.689
OH
N
Me
rac-7r
300
200
100
0
0.0 2.5 5.0 7.5 10.0 12.5 15.0 17.5 20.0
min

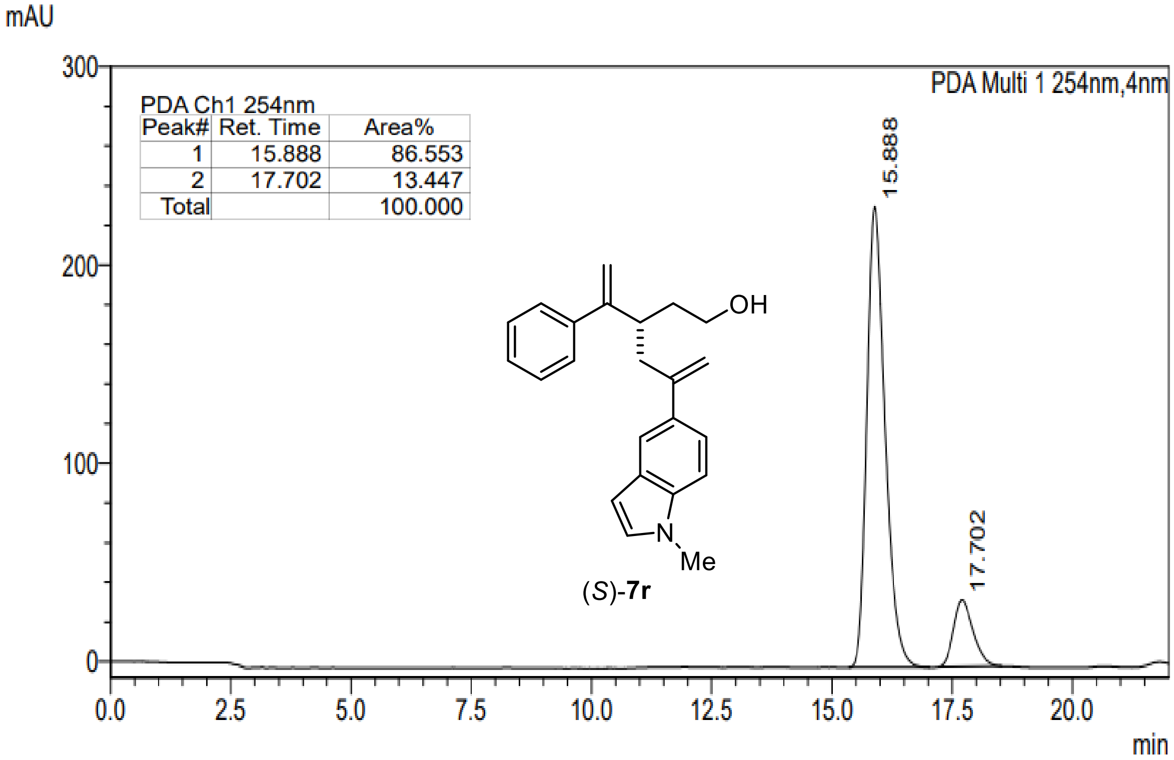
mAU
PDA Multi 1 254nm,4nm
PDA Ch1 254nm
Peak# Ret. Time Area%
1 15.888 86.553
2 17.702 13.447
Total 100.000
15.888
17.702
OH
N
Me
(S)-7r
300
200
100
0
0.0 2.5 5.0 7.5 10.0 12.5 15.0 17.5 20.0
min

(*S*)-5-cyclohexyl-3-(1-phenylvinyl)hex-5-en-1-ol (7s)

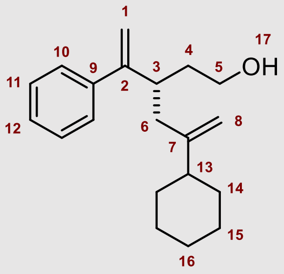

7s

Following **GP-II** using boronate **1a** (0.3 mmol, 1.0 equiv.) and 2-cyclohexylallyl acetate **2k** (0.36 mmol, 1.2 equiv.). The reaction was conducted at 25 °C for 24 h to afford compound **7s** as a yellow oil in 28% yield (24 mg) after chromatography on silica gel (eluent: pentane/EtOAc, 10:1).

TLC: 0.25, pentane/EtOAc = 10:1

^{1}H NMR (400 MHz, $CDCl_3$) δ (ppm) = 7.39 – 7.24 (m, 5H, *H10, H11* and *H12*), 5.29 (d, $^4J_{H\text{-}H}$ = 1.0 Hz, 1H, *H1*), 5.10 (bs, 1H, *H1*), 4.77 (bs, 1H, *H8*), 4.73 (m, 1H, *H8*), 3.78 – 3.59 (m, 2H, *H5*), 2.99 – 2.89 (m, 1H, *H3*), 2.34 (dd, $J_{H\text{-}H}$ = 14.7, 6.4 Hz, 1H, *H6*), 2.14 (dd, $J_{H\text{-}H}$ = 14.7, 6.4 Hz, 1H, *H6*), 1.90 – 1.82 (m, 1H, *H4*), 1.90 – 1.82 (m, 1H, *H4*), 1.80 – 1.62 (m, 6H, *H4, H14, H15*), 1.31 – 0.99 (m, 7H, *H14, H15 and H17*).

^{13}C{^{1}H} NMR (101 MHz, $CDCl_3$) δ (ppm) = 153.1 (*C7*), 152.8 (*C2*), 143.1 (*C9*), 128.4 (*C11*), 127.4 (*C12*), 126.9 (*C10*), 112.8 (*C1*), 109.0 (*C8*), 61.3 (*C5*), 43.8 (*C13*), 41.6 (*C6*), 39.0 (*C3*), 37.2 (*C4*), 32.6 (*C14 or C15*), 27.0 (*C14 or C15*), 26.6 (*C16*).

HRMS (ESI +**)**: calculated for $C_{20}H_{27}$ $[M–OH]^+$: 267.2108; found: 267.2108.

IR (neat) ν (cm^{-1}) = 3325, 3080, 2923, 2851, 1638, 1493, 1447, 1047, 1028, 887, 776, 701.

HPLC: 89:11 *er*, AD, 1 mL/min, hexane:*i*PrOH = 98:2, λ = 254 nm, 30 °C, t_R (major) = 10.5 min, t_R (minor) = 11.4 min.

$[\alpha]^{20}_D$: +4.7° (*c* = 0.5, $CHCl_3$).

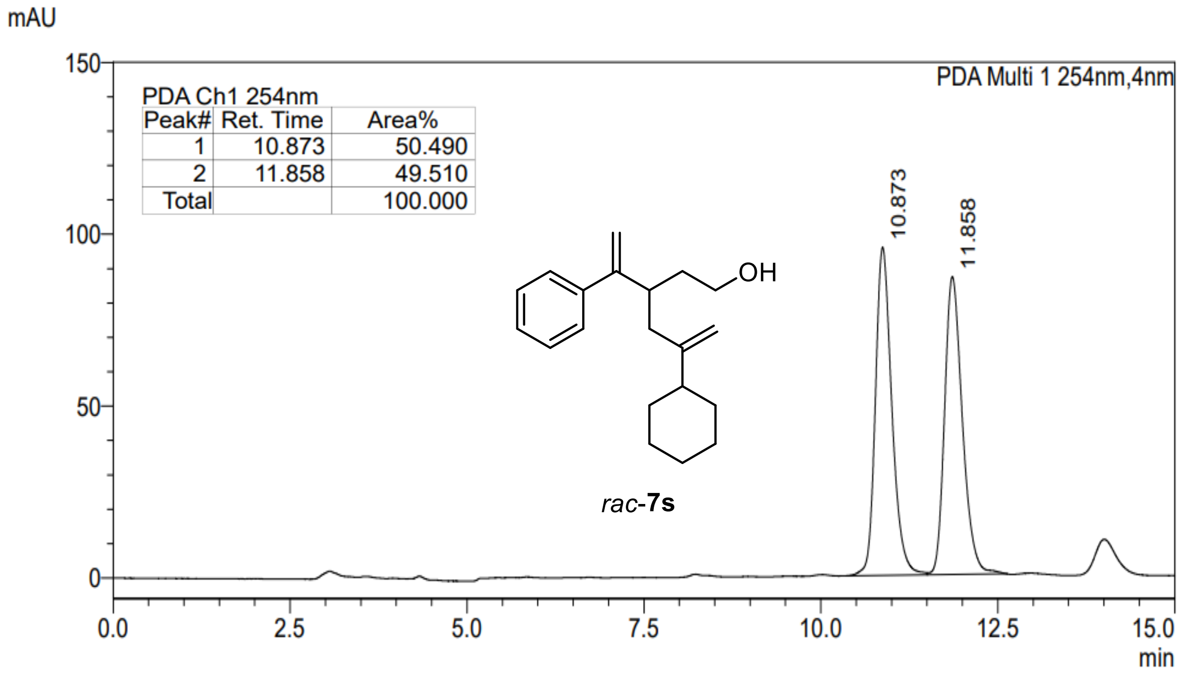

Peak#	Ret. Time	Area%
1	10.873	50.490
2	11.858	49.510
Total		100.000

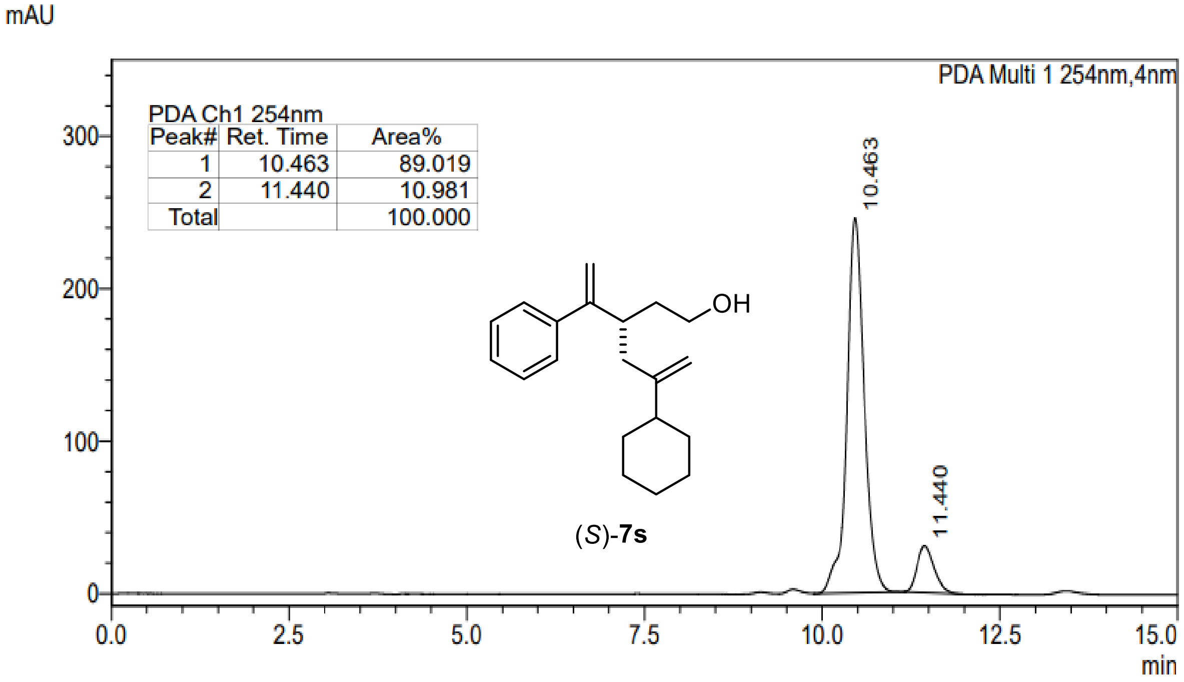

Peak#	Ret. Time	Area%
1	10.463	89.019
2	11.440	10.981
Total		100.000

(*S*)-5-methyl-3-(1-phenylvinyl)hex-5-en-1-ol (7t)

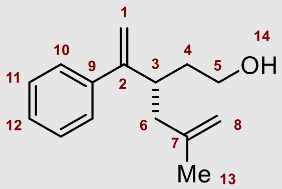

7t

Following **GP-II** using boronate **1a** (0.3 mmol, 1.0 equiv.) and 2-methylallyl acetate **2b** (0.36 mmol, 1.2 equiv.), The reaction was conducted at 25 °C for 24 h to afford compound **7t** as a yellow oil in 68% yield (44 mg) after chromatography on silica gel (eluent: pentane/EtOAc, from 10:1 to 6:1).

TLC: 0.3, pentane/EtOAc = 5:1

^{1}H NMR (400 MHz, $CDCl_3$) δ (ppm) = 7.33 – 7.18 (m, 5H, *H10, H11 and H12*), 5.23 (d, $^4J_{H\text{-}H}$ = 1.0 Hz, 1H, *H1*), 5.04 (t, $^4J_{H\text{-}H}$ = 0.8 Hz, 1H, *H1*), 4.71 (m, 1H, *H8*), 4.65 (m, 1H, *H8*), 3.69 – 3.53 (m, 2H, *H5*), 2.94 – 2.84 (m, 1H, *H3*), 2.24 (dd, $J_{H\text{-}H}$ = 14.0, 8.1 Hz, 1H, *H6*), 2.10 (dd, $J_{H\text{-}H}$ = 14.0, 8.1 Hz, 1H, *H6*), 1.82 – 1.72 (m, 1H, *H4*), 1.71 – 1.61 (m, 4H, *H4 and H13*), 1.16 (t, $^3J_{H\text{-}H}$ = 5.2 Hz, 1H, *H18*).

^{13}C{^{1}H} NMR (101 MHz, $CDCl_3$) δ (ppm) = 152.6 (*C2*), 143.9 (*C7*), 143.0 (*C9*), 128.4 (*C11*), 127.4 (*C12*), 126.9 (*C10*), 113.0 (*C1*), 112.5 (*C8*), 61.2 (*C5*), 44.2 (*C6*), 38.7 (*C3*), 37.1 (*C4*), 22.5 (*C13*).

HRMS (ESI +**)**: calculated for $C_{15}H_{21}O$ $[M+H]^+$: 217.1587; found: 257.1593.

IR (neat) ν (cm^{-1}) = 3323, 3073, 2933, 1646, 1493, 1442, 1374, 1047, 1028, 889, 776, 701.

HPLC: 93:7 *er*, OD, 1 mL/min, hexane:*i*PrOH = 95:5, λ = 254 nm, 30 °C, t_R (major) = 7.9 min, t_R (minor) = 6.8 min.

$[\alpha]^{20}_D$: +26.0° (*c* = 0.5, $CHCl_3$).

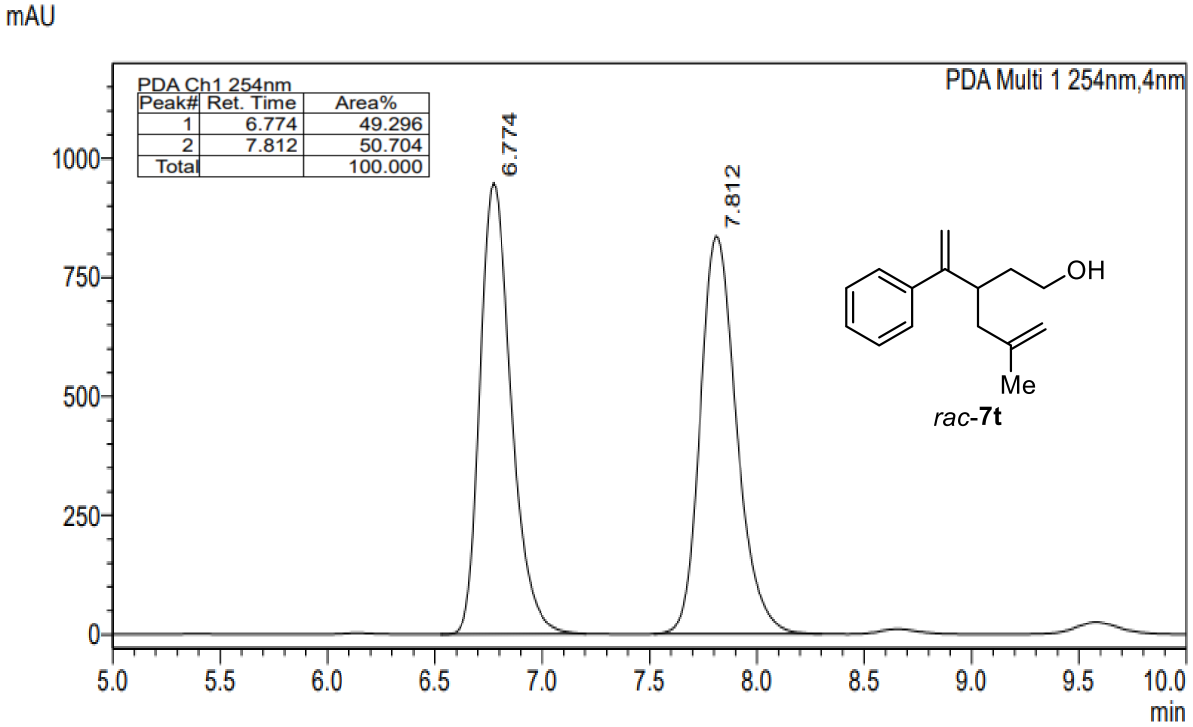
mAU
PDA Ch1 254nm
Peak# Ret. Time Area%
1 6.774 49.296
2 7.812 50.704
Total 100.000
PDA Multi 1 254nm,4nm
6.774
7.812
OH
Me
rac-7t
1000
750
500
250
0
5.0 5.5 6.0 6.5 7.0 7.5 8.0 8.5 9.0 9.5 10.0
min

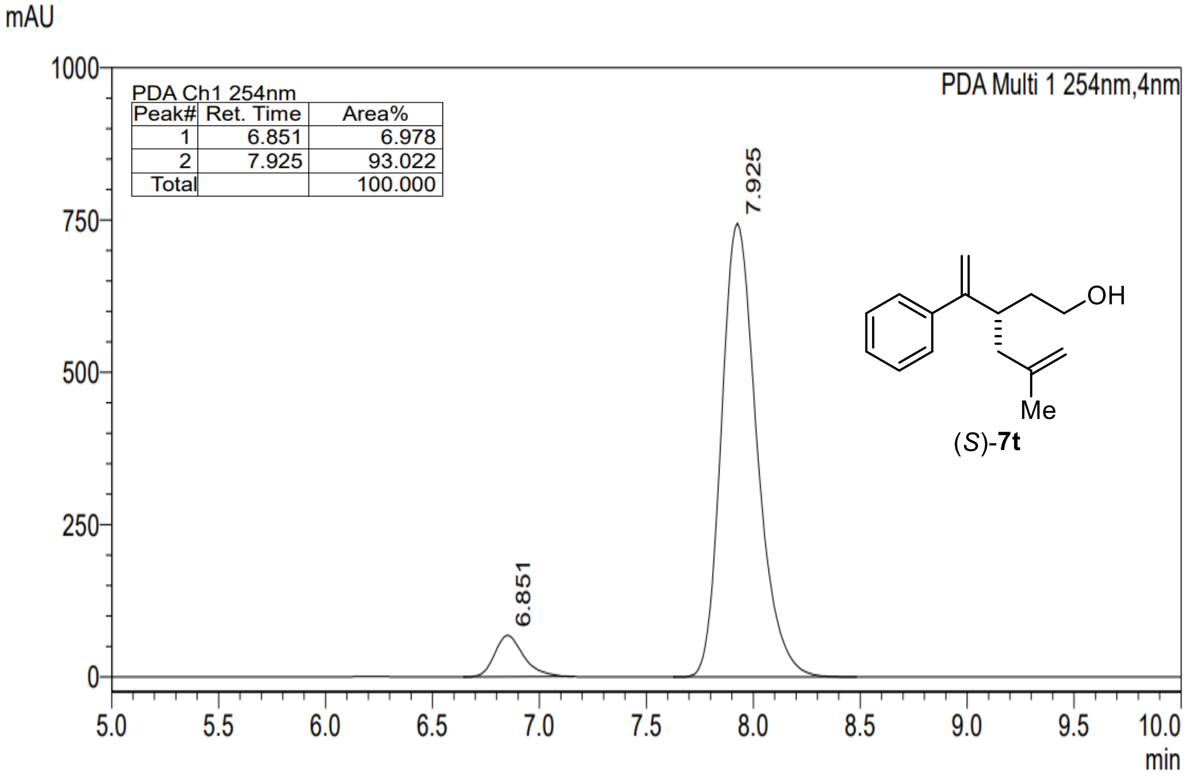
mAU
PDA Ch1 254nm
Peak# Ret. Time Area%
1 6.851 6.978
2 7.925 93.022
Total 100.000
PDA Multi 1 254nm,4nm
7.925
6.851
OH
Me
(S)-7t
1000
750
500
250
0
5.0 5.5 6.0 6.5 7.0 7.5 8.0 8.5 9.0 9.5 10.0
min

(*R*)-4,4-dimethyl-3-(1-phenylvinyl)hex-5-en-1-ol (7u)

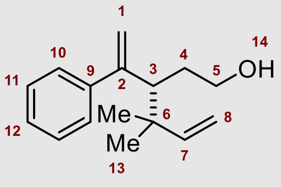

7u

Following **GP-II** using boronate **1a** (0.3 mmol, 1.0 equiv.) and allyl acetate **2c** (0.36 mmol, 1.2 equiv.), The reaction was conducted at 25 °C for 24 h to afford compound **7u** as a yellow oil in 17% yield (12 mg) after chromatography on silica gel (eluent: pentane/EtOAc, from 10:1 to 7.5:1).

TLC: 0.3, pentane/EtOAc = 5:1

^{1}H NMR (400 MHz, $CDCl_3$) δ (ppm) = 7.42 – 7.37 (m, 2H, *H10*), 7.34 – 7.28 (m, 2H, *H11*), 7.26 – 7.21 (m, 2H, *H11*), 5.86 – 5.77 (m, 1H, *H7*), 5.43 (t, $^{4}J_{H\text{-}H}$ = 0.8 Hz, 1H, *H1*), 5.07 (bs, 1H, *H1*), 4.95 (s, 1H, *H8*), 4.91 (dd, $^{3}J_{H\text{-}H}$ = 7.3, $^{4}J_{H\text{-}H}$ = 1.4 Hz, 1H, *H8*), 3.84 – 3.76 (m, 1H, *H5*), 3.73 – 3.63 (m, 1H, *H5*), 2.76 (dd, $^{3}J_{H\text{-}H}$ = 12.7, 2.6 Hz, 1H, *H3*), 2.02 – 1.92 (m, 1H, *H4*), 1.80 – 1.70 (m, 1H, *H4*), 1.21 (dd, $^{3}J_{H\text{-}H}$ = 5.7, 4.5 Hz, 1H, *H14*), 0.95 (s, 3H, *H13*), 0.89 (s, 3H, *H13*).

^{13}C{^{1}H} NMR (101 MHz, $CDCl_3$) δ (ppm) = 150.2 (*C2*), 147.4 (*C7*), 146.3 (*C9*), 128.4 (*C11*), 127.1 (*C12*), 126.8 (*C10*), 115.1 (*C1*), 111.6 (*C8*), 61.9 (*C5*), 49.1 (*C3*), 40.8 (*C6*), 33.9 (*C4*), 27.1 (*C13*), 23.0 (*C13*).

HRMS (ESI +**)**: calculated for $C_{16}H_{22}OK$ $[M+K]^+$: 269.1303; found: 269.1308.

IR (neat) ν (cm^{-1}) = 3321, 3081, 2928, 1620, 1493, 1442, 1175, 1049, 1029, 903, 778, 704.

HPLC: 96:4 *er*, OD, 1 mL/min, hexane:*i*PrOH = 95:5, λ = 254 nm, 30 °C, t_R (major) = 10.8 min, t_R (minor) = 8.3 min.

$[\alpha]^{20}_{D}$: –69.2° (*c* = 0.5, $CHCl_3$).

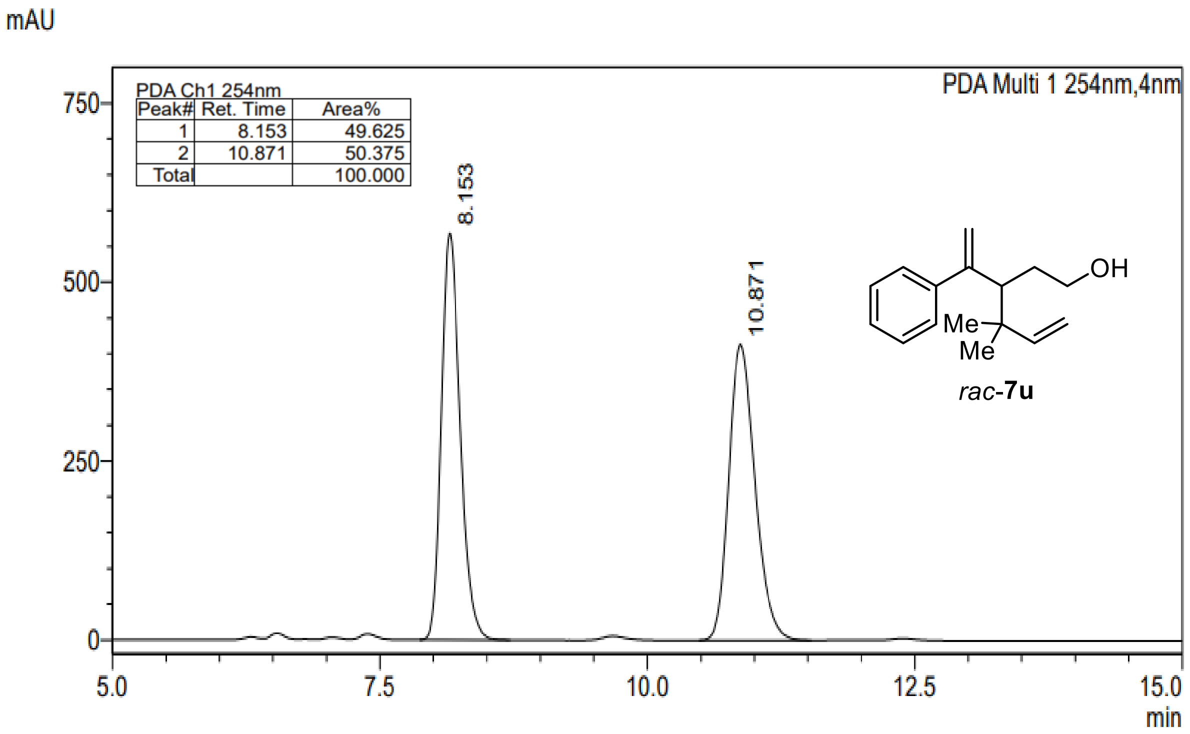

Peak#	Ret. Time	Area%
1	8.153	49.625
2	10.871	50.375
Total		100.000

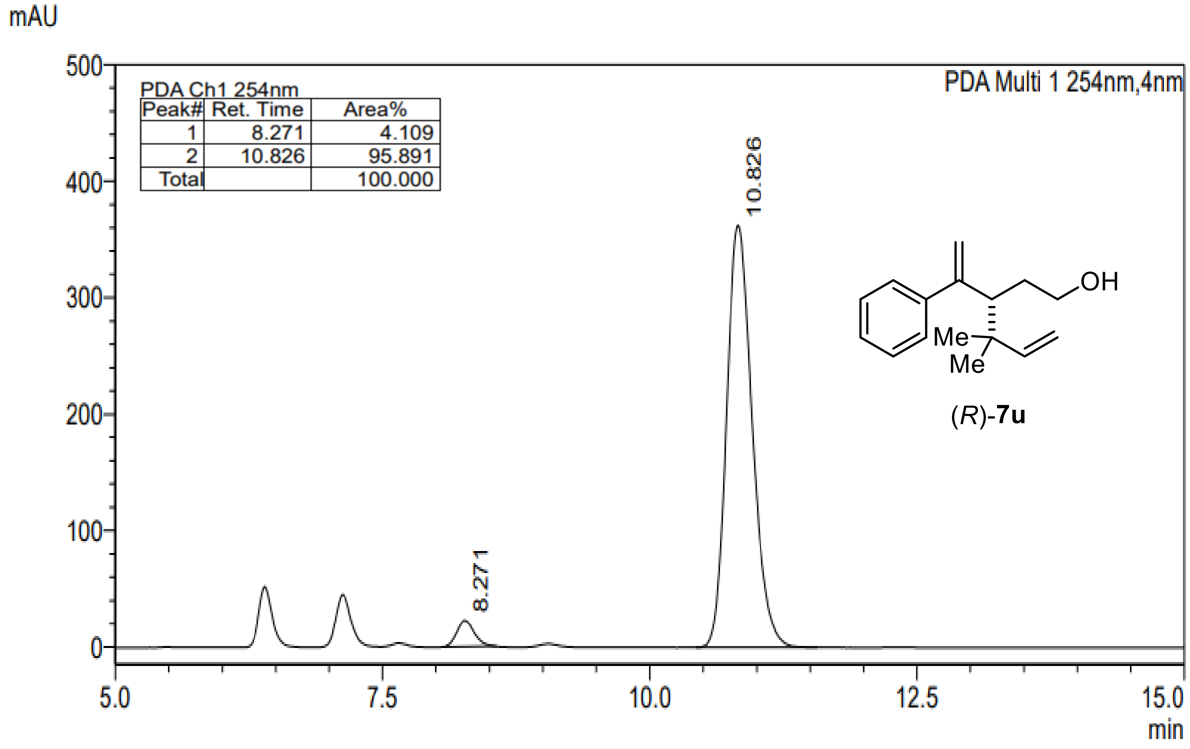

Peak#	Ret. Time	Area%
1	8.271	4.109
2	10.826	95.891
Total		100.000

4. Gram scale experiment

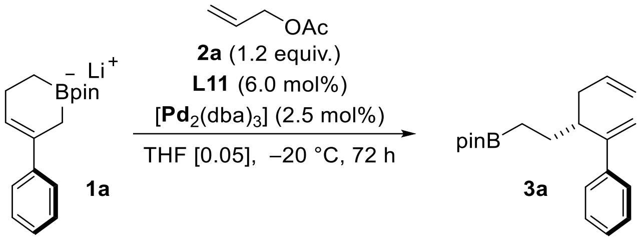

In a glovebox, to an oven-dried 10 mL vial, Pd_2dba_3 (82.4 mg, 0.09 mmol, 2.5 mol%) and ligand **L11** (89.7 mg, 0.22 mmol, 6 mol%) were dissolved in 6 mL of THF, and stirred at 25 °C. After 15 minutes, this solution was added by pipette into a Schlenk containing boronate **1a** (1.21 g, 3.6 mmol, 1.0 equiv.). The vial was rinsed with additional 6 mL of THF. Subsequently, 60 mL of THF was added into the Schlenk. The system was closed and kept in a –20 °C bath. After 15 minutes, at the same temperature, allyl acetate **2a** (466 μL, 4.32 mmol, 1.2 equiv.) was added by microsyringe. After addition, the system was closed, and the mixture was stirred at –20 °C. After 72 h, the reaction was filtered on Celite, washed with Et_2O (3 × 10 mL), dried over Na_2SO_4 and concentrated under reduced pressure after filtration. The residue was purified by flash column chromatography on silica gel (eluent: pentane/EtOAc, 100:1), affording compound **3a** as a yellow oil in 71% yield (800 mg, 96.5:3.5 *er*).

Note: the enantiomeric ratio of **3a** was measured after oxidation to the alcohol **7a**.

(*R*)-4,4,5,5-tetramethyl-2-(3-(1-phenylvinyl)hex-5-en-1-yl)-1,3,2-dioxaborolane (3a)

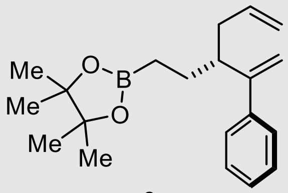

3a

TLC: 0.8, pentane/EtOAc = 100:1

^{1}H NMR (400 MHz, $CDCl_3$) δ (ppm) = 7.37 – 7.19 (m, 5H), 5.86 – 5.70 (m, 1H), 5.26 (d, J = 1.2 Hz, 1H), 5.05 – 4.91 (m, 3H), 2.59 (p, J = 6.8 Hz, 1H), 2.31 – 2.14 (m, 2H), 1.68 – 1.57 (m, 2H), 1.22 (s, 12H), 0.92 – 0.65 (m, 2H).

^{13}C{^{1}H} NMR (101 MHz, $CDCl_3$) δ (ppm) = 152.1, 143.6, 137.3, 128.2, 127.1, 126.9, 115.9, 112.9, 83.0, 46.1, 38.7, 28.2, 24.98, 24.96.

^{11}B{^{1}H} NMR (128 MHz, $CDCl_3$) δ (ppm) = 34.3

HRMS (ESI +**)**: calculated for $C_{20}H_{30}BO_2$ $[M+H]^+$: 313.2334; found: 313.2317.

IR (neat) ν (cm^{-1}) = 3077, 2977, 2928, 1370, 1316, 1143, 967, 907, 846, 777, 700.

$[\alpha]^{20}_D$: +4.7° (*c* = 0.5, $CHCl_3$).

HPLC: 96.5:3.5 *er*, OD, 1 mL/min, hexane:*i*PrOH = 95:5, λ = 254 nm, 30 °C, t_R (major) = 8.9 min, t_R (minor) = 7.6 min.

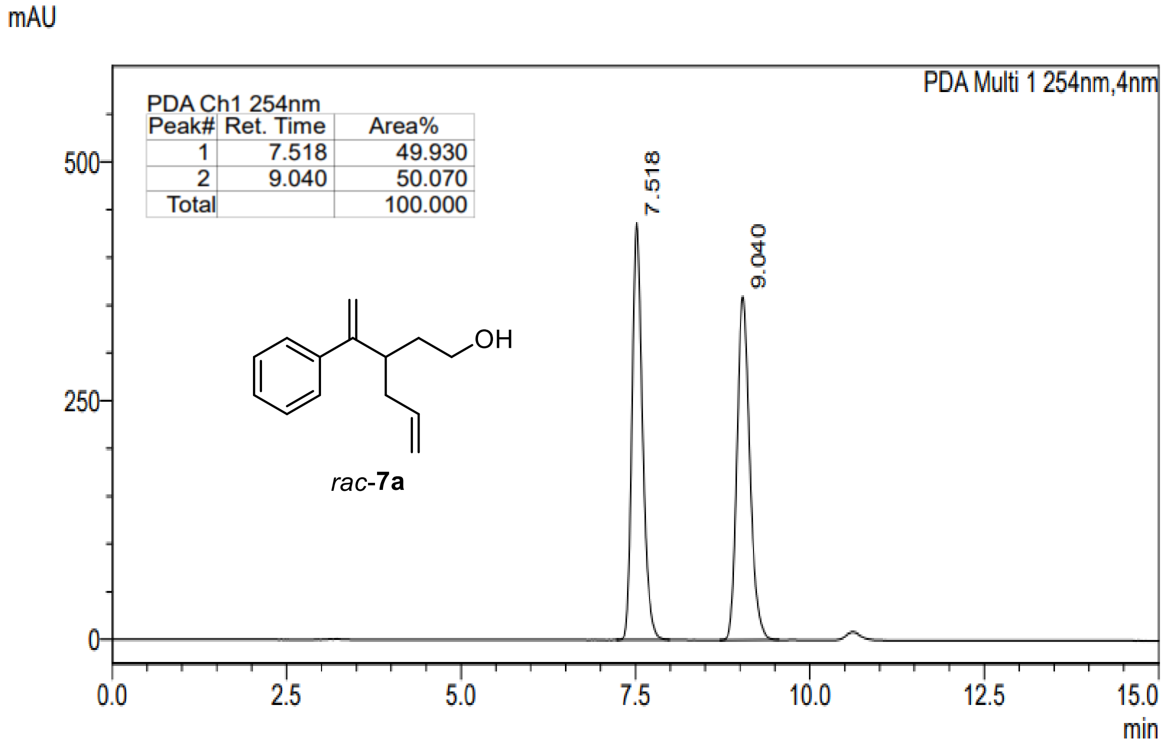

PDA Ch1 254nm

Peak#	Ret. Time	Area%
1	7.518	49.930
2	9.040	50.070
Total		100.000

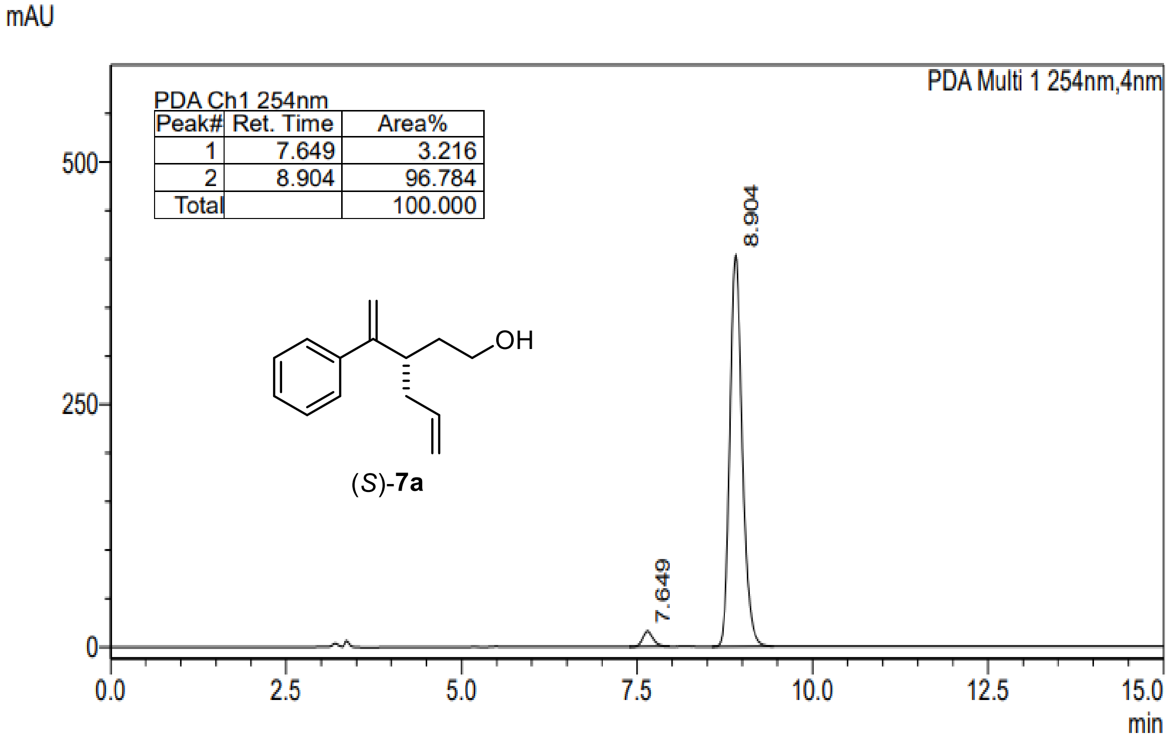

PDA Ch1 254nm

Peak#	Ret. Time	Area%
1	7.649	3.216
2	8.904	96.784
Total		100.000

5. Selective transition metal-catalyzed derivatizations of 3a

5.1 Pd-catalyzed cross-coupling reaction

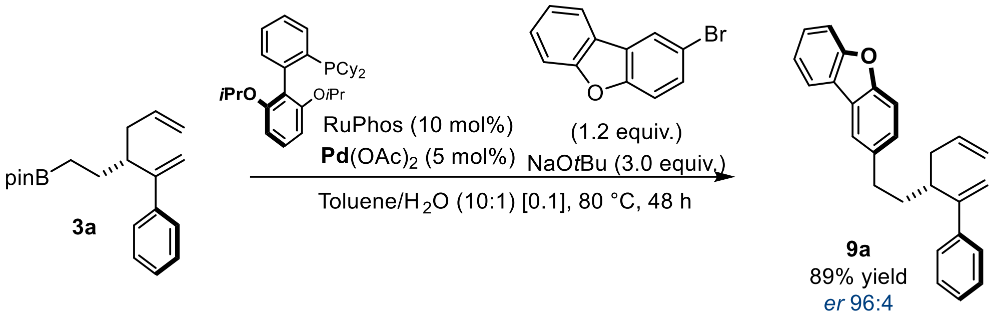

In a glovebox, to an oven-dried 5 mL J-Young valve Schlenk, $Pd(OAc)_2$ (3.4 mg, 0.015 mmol, 5 mol%), RuPhos (14 mg, 0.03 mmol, 10 mol%), NaO*t*Bu (87 mg, 0.9 mmol, 3.0 equiv.) and 2-bromodibenzofuran (89 mg, 0.36 mmol, 1.2 equiv.) were dissolved in toluene (3 mL). Subsequently, enantioenriched boronic ester **3a** (98 μL, 96.5:3.5 *er*, 0.3 mmol, 1.0 equiv.) was added by microsyringe. The Schlenk was closed and taken out of the glovebox and connected to a Schlenk line. Water (0.3 mL) was added by syringe, the system was closed and the mixture was stirred at 80 °C. After 48 h, the reaction was cooled to room temperature, filtered on Celite, washed with Et_2O (3 × 5 mL), dried over Na_2SO_4, filtered and concentrated under reduced pressure. The residue was purified by flash column chromatography on silica gel (eluent: pentane/Et_2O, 100:1), to afford compound **9a** as a yellow oil in 89% yield (94 mg, 96:4 *er*).

(*R*)-2-(3-(1-phenylvinyl)hex-5-en-1-yl)dibenzo[b,d]furan (9a)

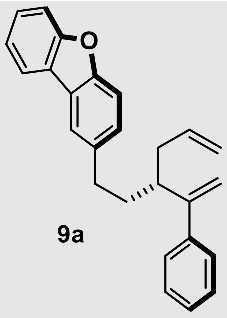

9a

TLC: 0.8, pentane/Et_2O = 20:1

^{1}H NMR (400 MHz, $CDCl_3$) δ (ppm) = δ 7.92 – 7.84 (m, 1H), 7.63 (d, J = 2.4 Hz, 1H), 7.55 (dt, J = 8.2, 0.9 Hz, 1H), 7.48 – 7.39 (m, 2H), 7.40 – 7.27 (m, 6H), 7.20 (dd, J = 8.4, 1.9 Hz, 1H), 5.88 – 5.73 (m, 1H), 5.38 (d, J = 1.1 Hz, 1H), 5.15 (s, 1H), 5.10 – 4.95 (m, 2H), 2.89 – 2.80 (m, 1H), 2.79 – 2.67 (m, 2H), 2.42 – 2.30 (m, 1H), 2.31 – 2.19 (m, 1H), 1.96 – 1.86 (m, 2H).

$^{13}C\{^1H\}$ NMR (101 MHz, $CDCl_3$) δ (ppm) = 156.6, 154.8, 152.1, 143.3, 137.2, 136.9, 128.4, 127.8, 127.4, 127.1, 126.9, 124.4, 124.3, 122.7, 120.7, 120.2, 116.3, 113.1, 111.8, 111.4, 43.2, 39.1, 36.3, 33.3.

HRMS (ESI +**)**: calculated for $C_{26}H_{25}O$ $[M+H]^+$: 353.1900; found: 353.1900.

IR (neat) ν (cm^{-1}) = 3076, 2925, 1478, 1448, 1193, 1025, 993, 898, 841, 777, 747, 699.

$[\alpha]^{20}_D$: –8.2° (*c* = 0.5, $CHCl_3$).

HPLC: 96:4 *er*, OD, 1 mL/min, hexane:*i*PrOH = 95:5, λ = 254 nm, 30 °C, t_R (major) = 8.3 min, t_R (minor) = 7.6 min.

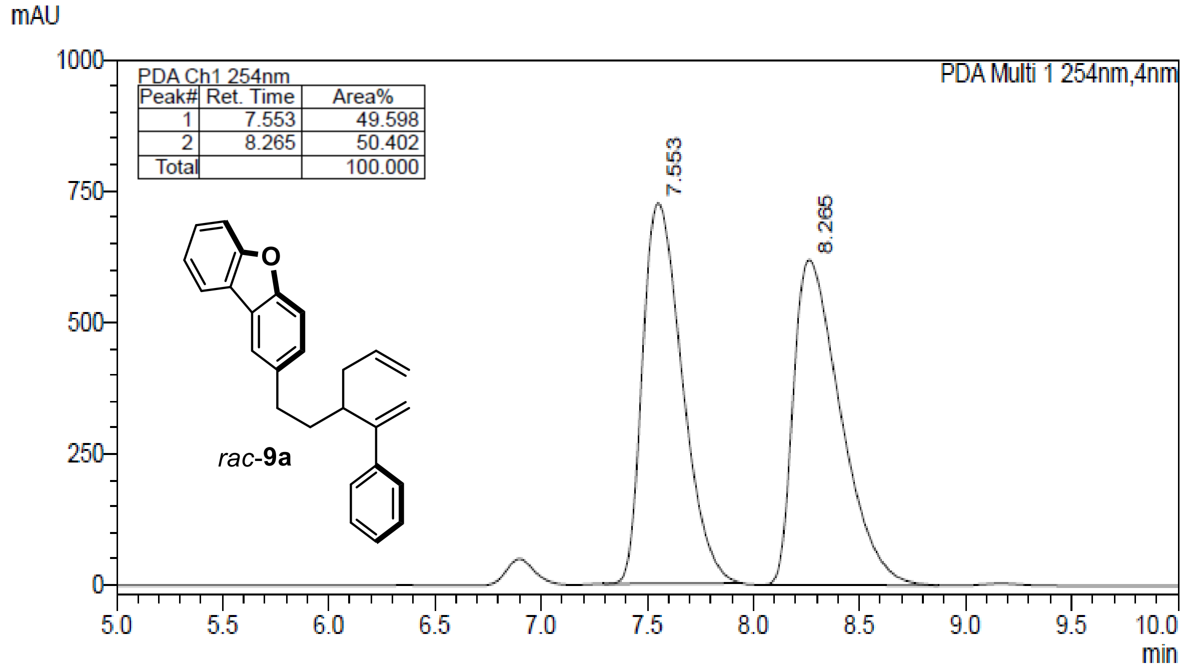
mAU
1000
750
500
250
0
PDA Ch1 254nm
Peak# Ret. Time Area%
1 7.553 49.598
2 8.265 50.402
Total 100.000
PDA Multi 1 254nm,4nm
7.553
8.265
O
rac-9a
5.0 5.5 6.0 6.5 7.0 7.5 8.0 8.5 9.0 9.5 10.0
min

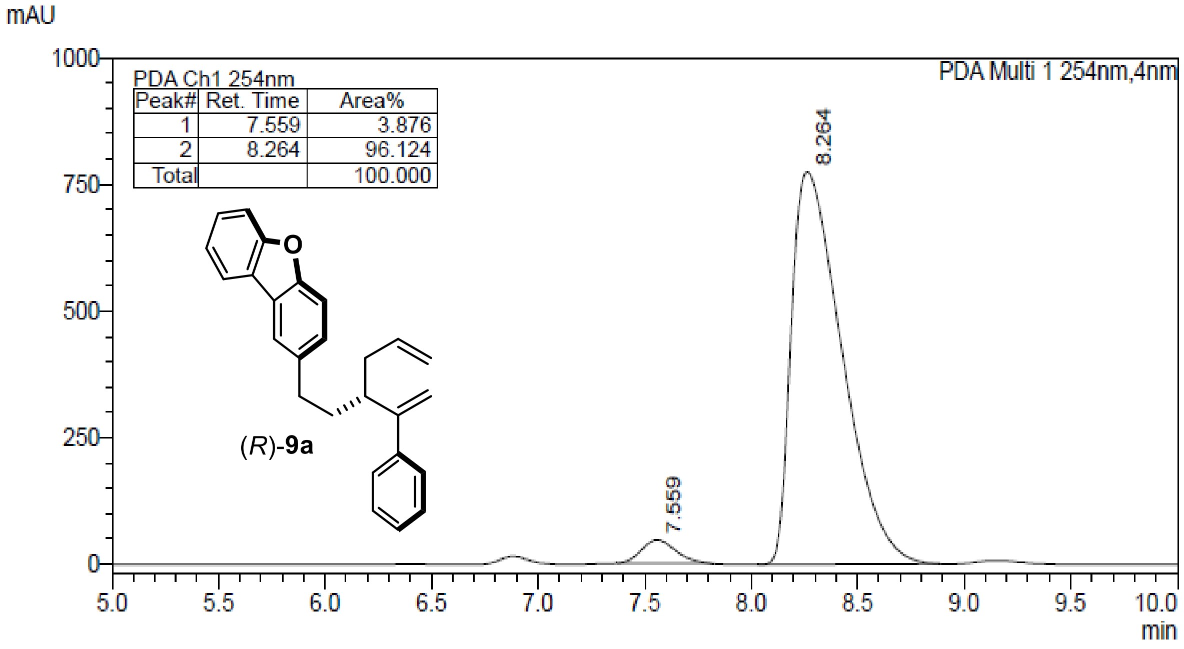
mAU
1000
750
500
250
0
PDA Ch1 254nm
Peak# Ret. Time Area%
1 7.559 3.876
2 8.264 96.124
Total 100.000
PDA Multi 1 254nm,4nm
8.264
7.559
O
(R)-9a
5.0 5.5 6.0 6.5 7.0 7.5 8.0 8.5 9.0 9.5 10.0
min

5.2 Cu-catalyzed protoboration reaction

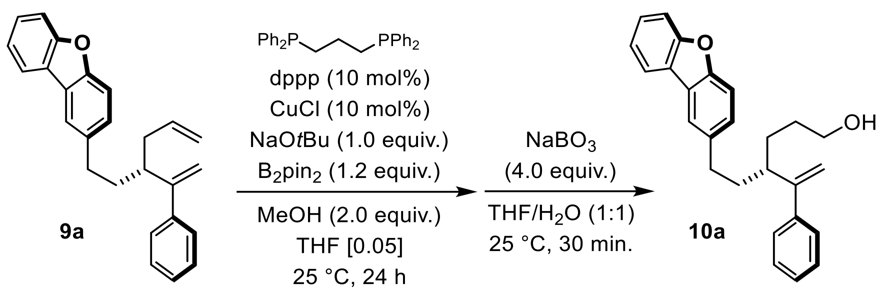

In a glovebox, to an oven-dried 5 mL Schlenk, CuCl (1.0 mg, 0.01 mmol, 10 mol%), dppp (4.2 mg, 0.01 mmol, 10 mol%), NaO*t*Bu (9.6 mg, 0.1 mmol, 1.0 equiv.) and B_2pin_2 (31 mg, 0.12 mmol, 1.2 equiv.) were added. Subsequently, compound **9a** (35 mg, 96:4 *er*, 0.1 mmol, 1.0 equiv.) was added with 2 mL of THF. Subsequently, MeOH (8 μL, 0.2 mmol, 2.0 equiv.) was added by microsyringe. The mixture was stirred at 25 °C. After 24 h, the reaction was filtered on Celite, washed with Et_2O (3 × 5 mL), dried over Na_2SO_4 and concentrated under reduced pressure after filtration. The alkali oxidation was conducted next. The crude reaction mixture was dissolved in THF (3 mL), and $NaBO_3$ (62 mg, 0.4 mmol, 4.0 equiv.) in H_2O (3 mL) was added at room temperature. The mixture was stirred vigorously at 25 °C. After 30 min., the reaction mixture was extracted with Et_2O (3 × 5 mL). The combined organic phases were dried over Na_2SO_4 and concentrated under reduced pressure after filtration. The residue was purified by flash column chromatography on silica gel (eluent: pentane/EtOAc, from 10:1 to 7.5:1), affording compound **10a** as a yellow oil in 49% yield (18 mg, 97:3 *er*).

(*R*)-4-(2-(dibenzo[b,d]furan-2-yl)ethyl)-5-phenylhex-5-en-1-ol (10a)

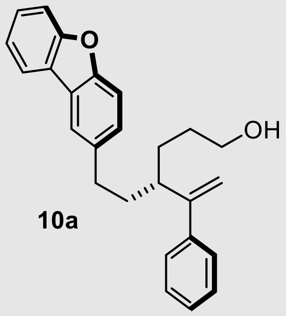

10a

TLC: 0.2, pentane/EtOAc = 5:1

^{1}H NMR (400 MHz, $CDCl_3$) δ (ppm) = 7.92 – 7.85 (m, 1H), 7.63 (d, J = 1.5 Hz, 1H), 7.55 (dt, J = 8.2, 0.9 Hz, 1H), 7.48 – 7.39 (m, 2H), 7.39 – 7.26 (m, 6H), 7.20 (dd, J = 8.4, 1.8 Hz, 1H), 5.37 (d, J = 1.2 Hz, 1H), 5.14 (s, 0H), 3.62 (brs, 2H), 2.90 – 2.69 (m, 2H), 2.62 (brs, 1H), 2.00 – 1.83 (m, 2H), 1.70 – 1.53 (m, 4H), 1.18 (brs, 1H).

^{13}C{^{1}H} NMR (101 MHz, $CDCl_3$) δ (ppm) = 156.6, 154.8, 152.5, 143.3, 137.1, 128.4, 127.8, 127.4, 127.1, 126.9, 124.4, 124.3, 122.7, 120.7, 120.2, 113.1, 111.8, 111.4, 63.3, 43.5, 37.4, 33.5, 31.0, 30.6.

HRMS (ESI +**)**: calculated for $C_{26}H_{26}O_2Na$ $[M+Na]^+$: 393.1826; found: 393.1817.

IR (neat) ν (cm^{-1}) = 3347, 2928, 1478, 1448, 1193, 1056, 897, 747, 702.

$[\alpha]^{20}_D$: –5.8° (*c* = 0.5, $CHCl_3$).

HPLC: 97:3 *er*, IC, 1 mL/min, hexane:*i*PrOH = 95:5, λ = 205 nm, 30 °C, t_R (major) = 16.8 min, t_R (minor) = 15.4 min.

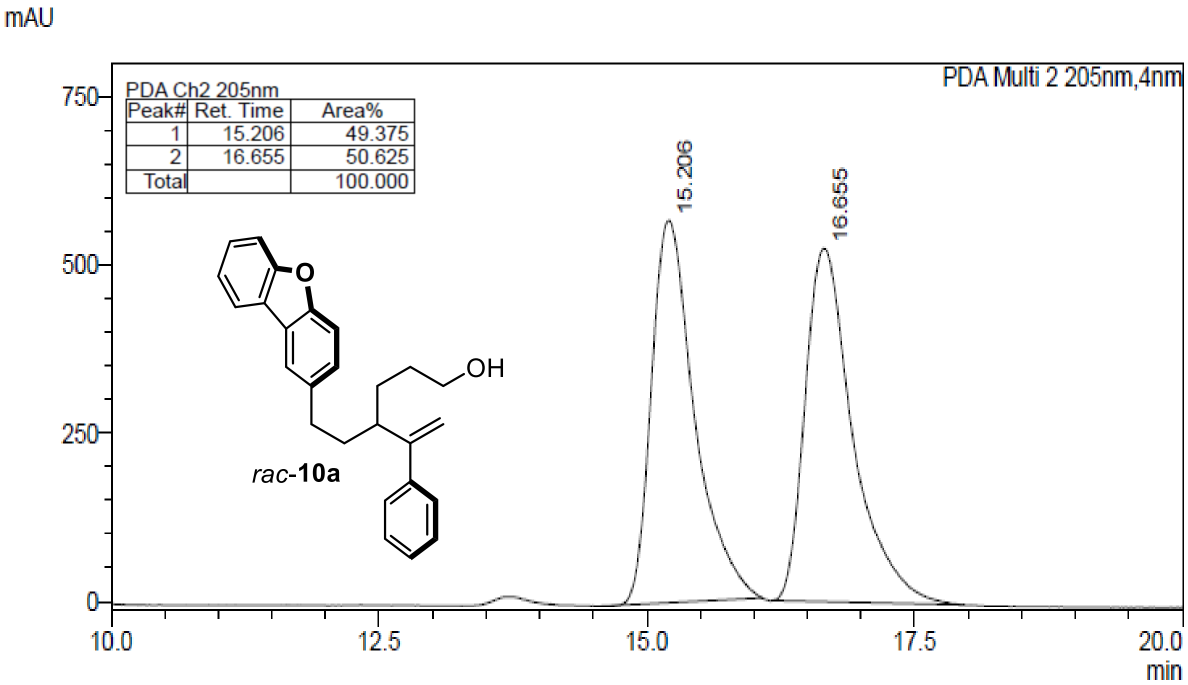

PDA Ch2 205nm

Peak#	Ret. Time	Area%
1	15.206	49.375
2	16.655	50.625
Total		100.000

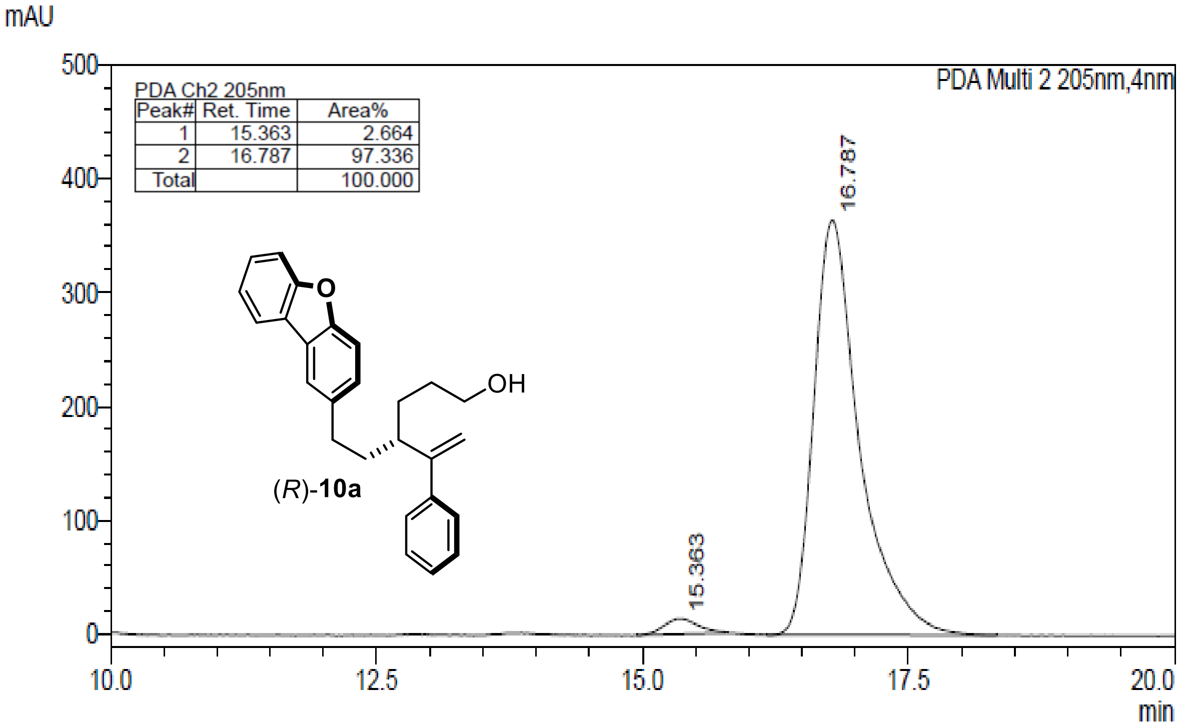

PDA Ch2 205nm

Peak#	Ret. Time	Area%
1	15.363	2.664
2	16.787	97.336
Total		100.000

5.3 Ir-catalyzed hydrogenation reaction

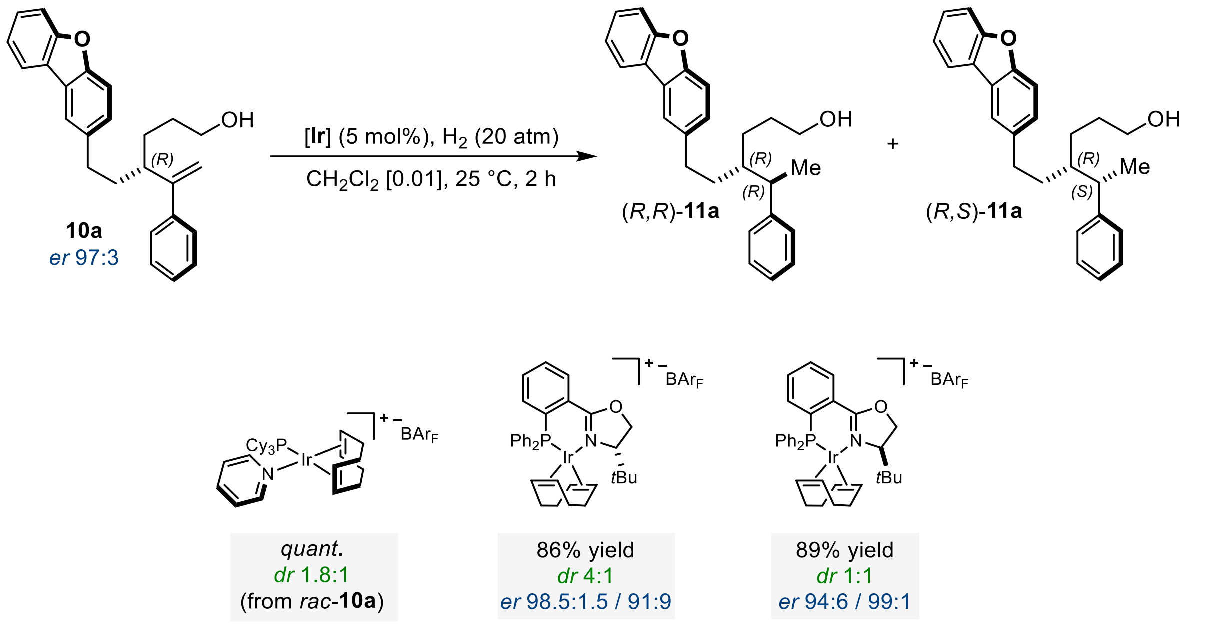

In a 10 mL oven-dried vial, compound **10a** (18.5 mg, 0.05 mmol, 97:3 *er*, 1.0 equiv.) and [**Ir**] catalyst (5 mol%) were dissolved in 5 mL of CH_2Cl_2. The vial was placed in the autoclave, and the atmosphere was changed into H_2 by three times fill-release cycles. Finally, the autoclave was pressurized with 20 atm H_2, and the reaction was stirred at 25°C. After 2 h, the pressure was slowly released by valve, and the vial was taken out. The reaction mixture was filtered on Celite, washed with Et_2O (3 × 5 mL), dried over Na_2SO_4 and concentrated under reduced pressure after filtration. The residue was purified by flash column chromatography on silica gel (eluent: pentane/EtOAc, from 5:1 to 3:1), affording compound **11a** as a yellow oil. The diastereoselectivity was measured by ^{1}H NMR of the crude reaction mixture using against an internal standard.

Full characterization for compound **11a** (*dr* = 4:1) was done:

6-(dibenzo[b,d]furan-2-yl)-4-(1-phenylethyl)hexan-1-ol (11a)

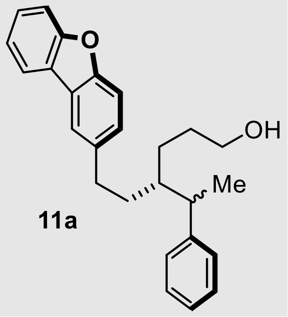

TLC: 0.2, pentane/EtOAc = 4:1

For major diastereoisomer:

^{1}H NMR:(400 MHz, $CDCl_3$) δ (ppm) = 7.95 – 7.90 (m, 1H), 7.66 (d, $^{3}J_{H\text{-}H}$ = 1.8 Hz, 1H), 7.57 – 7.52 (m, 1H), 7.48 – 7.40 (m, 2H), 7.36 – 7.26 (m, 3H), 7.23 – 7.16 (m, 4H), 3.58 (td, $^{3}J_{H\text{-}H}$ = 6.6 Hz, 0.9 Hz, 2H), 2.90 – 2.58 (m, 3H), 1.87 – 1.57 (m, 1H), 1.72 – 1.47 (m, 5H), 1.46 – 1.35 (m, 1H), 1.29 – 1.25 (m, 4H).

For minor diastereoisomer:

^{1}H NMR:(400 MHz, $CDCl_3$) δ (ppm) = 7.92 – 7.88 (m, 1H), 7.61 (d, $^{3}J_{H\text{-}H}$ = 1.8 Hz, 1H), 7.57 – 7.52 (m, 1H), 7.48 – 7.40 (m, 2H), 7.36 – 7.26 (m, 3H), 7.23 – 7.12 (m, 4H),), 3.64 – 3.53 (m, 2H), 2.90 – 2.58 (m, 3H), 1.87 – 1.57 (m, 1H), 1.72 – 1.47 (m, 5H), 1.46 – 1.35 (m, 1H), 1.29 – 1.25 (m, 4H).

Due to the complexity of ^{13}C NMR spectra all the peaks are listing, and partial peaks from minor diastereoisomer were noted in bracket:

^{13}C{^{1}H} NMR (101 MHz, $CDCl_3$) δ (ppm) = 156.6, 154.8, 146.63 (minor), 146.59, 137.4, 137.3 (minor), 128.3, 127.90 (minor), 127.86, 127.71 (minor), 127.68, 127.1, 126.0, 124.4, 124.33, 124.27 (minor), 122.7, 120.72, 120.68 (minor), 120.1 (minor), 120.0, 111.8, 111.4, 111.3 (minor), 63.5 (minor), 63.4, 43.6, 43.3 (minor), 42.0 (minor), 41.9, 33.8 (minor), 33.2 (minor), 33.1, 32.5, 30.3, 30.0 (minor), 27.3, 25.8 (minor), 17.9 (minor), 17.6.

HRMS (ESI +**)**: calculated for $C_{26}H_{32}O_2N$ $[M+NH_4]^+$: 390.2428; found: 390.2419.

IR (neat) ν (cm^{-1}) = 3328, 2927, 1478, 1448, 1193, 1054, 840, 806, 747, 700.

$[\alpha]^{20}_{D}$: –16.8° (*c* = 0.5, $CHCl_3$).

HPLC: 98.5:1.5 *er* for major diastereoisomer, 91:9 *er* for minor diastereoisomer. OD, 1 mL/min, hexane:*i*PrOH = 97:3, λ = 205 nm, 30 °C. For major diastereoisomer: t_{R1} = 41.1 min, t_{R2} = 53.1 min. For minor diastereoisomer: t_{R1} = 47.9 min, t_{R2} = 58.1 min.

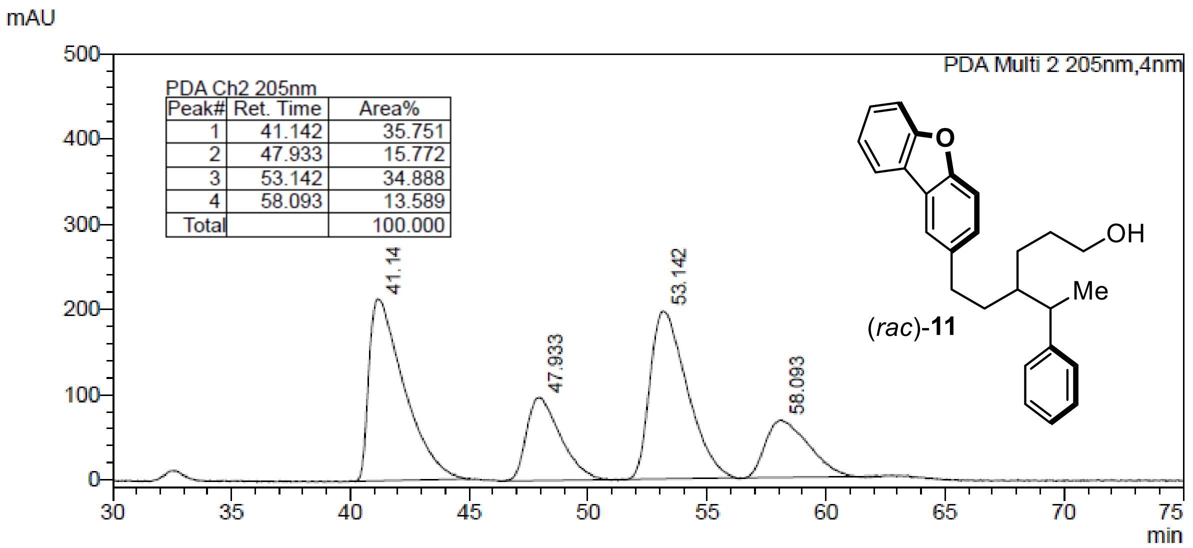

PDA Ch2 205nm

Peak#	Ret. Time	Area%
1	41.142	35.751
2	47.933	15.772
3	53.142	34.888
4	58.093	13.589
Total		100.000

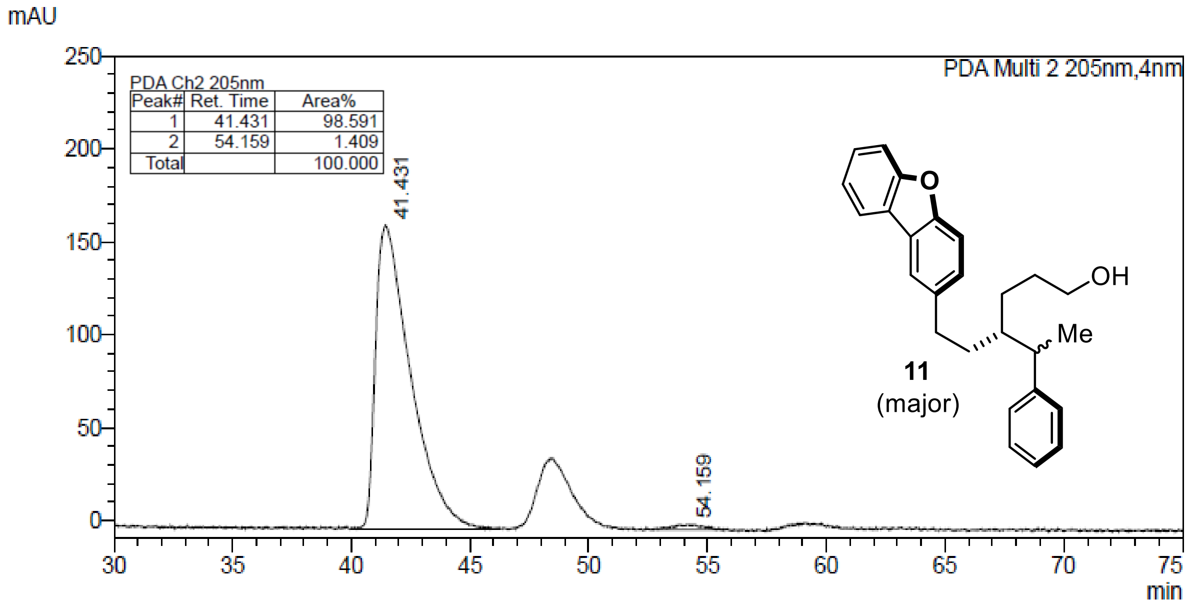

PDA Ch2 205nm

Peak#	Ret. Time	Area%
1	41.431	98.591
2	54.159	1.409
Total		100.000

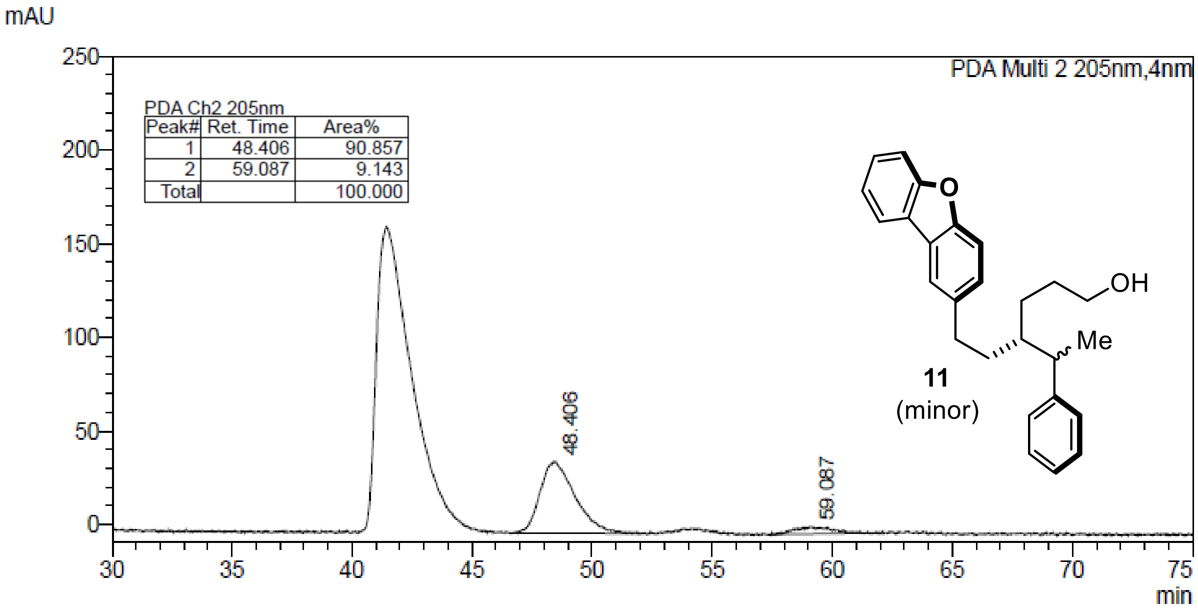

PDA Ch2 205nm

Peak#	Ret. Time	Area%
1	48.406	90.857
2	59.087	9.143
Total		100.000

5.4 Ru-catalyzed cross metathesis reaction

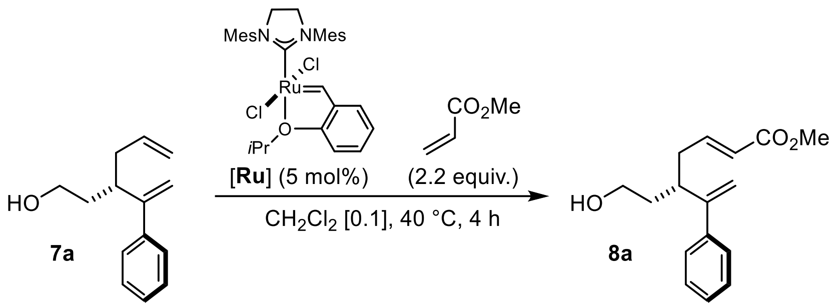

In a glovebox, to an oven-dried 5 mL J-Young valve Schlenk, Hoveyda-Grubbs precatalyst (9.4 mg, 0.015 mmol, 5 mol%) and compound **7a** (61 mg, 96.5:3.5 *er*, 0.3 mmol, 1.0 equiv.) were dissolved in 3 mL of CH_2Cl_2. Subsequently, methyl acrylate (60 μL, 0.66 mmol, 2.2 equiv.) was added by syringe. The system was closed and taken out of glovebox. The mixture was stirred at 40 °C. After 4 h, the reaction mixture was filtered on Celite, washed with Et_2O (3 × 5 mL), dried over Na_2SO_4 and concentrated under reduced pressure after filtration. The residue was purified by flash column chromatography on silica gel (eluent: pentane/EtOAc, from 5:1 to 2:1), affording compound **8a** as a yellow oil in 76% yield (59 mg, 97:3 *er*).

(*S*)-methyl (*X*,*E*)-5-(2-hydroxyethyl)-6-phenylhepta-2,6-dienoate (8a)

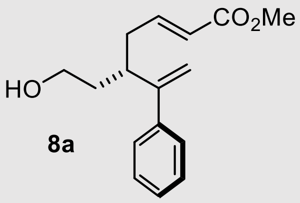

8a

TLC: 0.2, pentane/EtOAc = 2:1

^{1}H NMR (400 MHz, $CDCl_3$) δ (ppm) = 7.37 – 7.24 (m, 5H), 6.92 (dt, J = 15.6, 7.3 Hz, 1H), 5.82 (dt, J = 15.6, 1.5 Hz, 1H), 5.32 (d, J = 0.8 Hz, 1H), 5.08 (s, 1H), 3.75 – 3.60 (m, 5H), 2.96 (p, J = 6.7 Hz, 1H), 2.50 – 2.38 (m, 1H), 2.41 – 2.29 (m, 1H), 1.85 – 1.73 (m, 2H), 1.38 (brs, 1H).

^{13}C {^{1}H} NMR (101 MHz, $CDCl_3$) δ (ppm) = 167.0, 151.2, 147.3, 142.4, 128.5, 127.6, 126.9, 122.7, 113.6, 60.8, 51.6, 39.9, 37.3, 36.7.

HRMS (ESI +**)**: calculated for $C_{16}H_{20}O_3Na$ $[M+Na]^+$: 283.1305; found: 283.1301.

IR (neat) ν (cm^{-1}) = 3424, 2947, 1720, 1654, 1435, 1271, 1211, 1160, 1040, 901, 778, 702.

$[\alpha]^{20}_D$: +14.2° (*c* = 0.5, $CHCl_3$).

HPLC: 97:3 *er*, OD, 1 mL/min, hexane:*i*PrOH = 95:5, λ = 254 nm, 30 °C, t_R (major) = 21.9 min, t_R (minor) = 18.9 min.

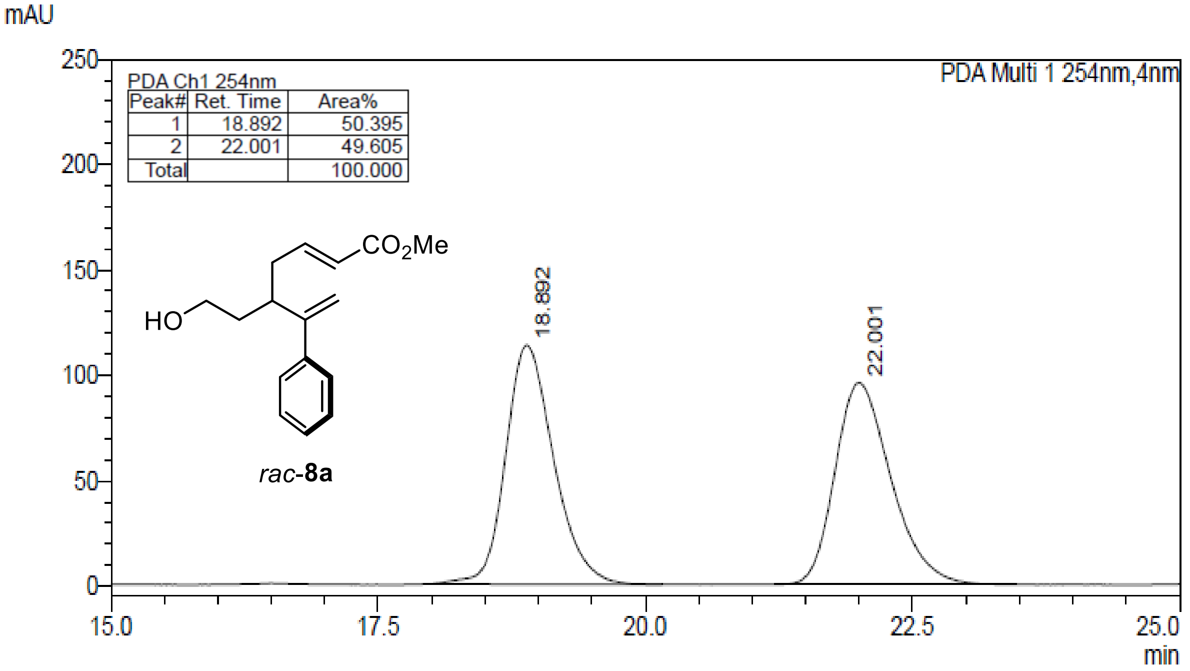
mAU
PDA Ch1 254nm
Peak# Ret. Time Area%
1 18.892 50.395
2 22.001 49.605
Total 100.000
PDA Multi 1 254nm,4nm
CO_2Me
HO
rac-8a
18.892
22.001
250
200
150
100
50
0
15.0
17.5
20.0
22.5
25.0
min

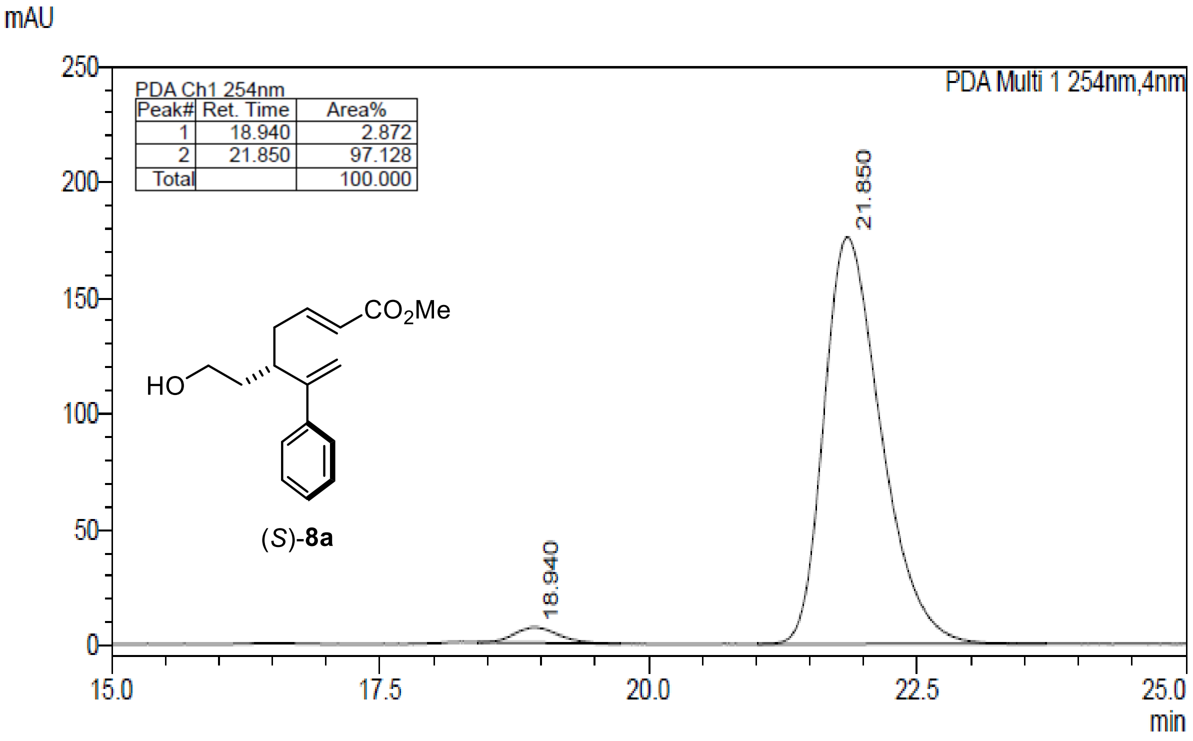
mAU
PDA Ch1 254nm
Peak# Ret. Time Area%
1 18.940 2.872
2 21.850 97.128
Total 100.000
PDA Multi 1 254nm,4nm
CO_2Me
HO
(S)-8a
21.850
18.940
250
200
150
100
50
0
15.0
17.5
20.0
22.5
25.0
min

5.5 Esterification of 7e

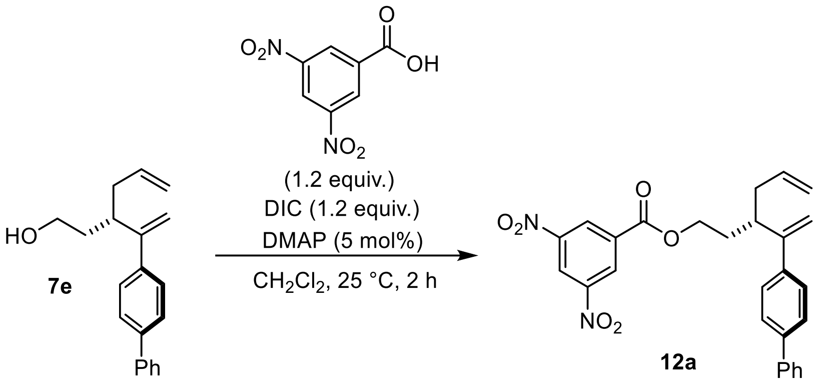

To an oven-dried 10 mL vial, the enantioenriched homoallylic alcohol **7e** (93:7 *er*) (15 mg, 0.05 mmol, 1.0 equiv.), DMAP (0.5 mg, 5 mol%) and 3,5-dinitrobenzoic acid (13 mg, 0.06 mmol, 1.2 equiv.) were dissolved in 3 mL of CH_2Cl_2. Subsequently, diisopropylcarbodiimide (DIC) (21 μL, 0.06 mmol, 1.2 equiv.) was added by microsyringe. The mixture was stirred at 25 °C. After 2 h, the reaction mixture was filtered on Celite, washed with Et_2O (3 × 5 mL) and concentrated under reduced pressure. The residue was purified by flash column chromatography on silica gel (eluent: pentane/EtOAc, from 30:1 to 20:1), to afford compound **12a** as a yellow solid in 98% yield (25 mg). Single crystals suitable for X-ray analysis were obtained by layering a solution of EtOAc with pentane.

(*S*)-3-(1-([1,1'-biphenyl]-4-yl)vinyl)hex-5-en-1-yl 3,5-dinitrobenzoate (12a)

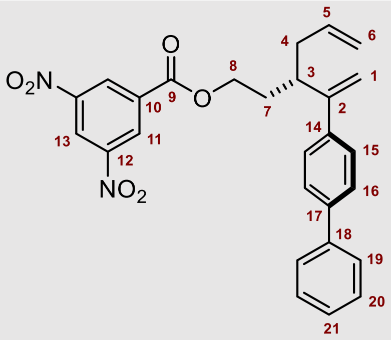

TLC: 0.4, pentane/EtOAc = 20:1

^{1}H NMR (400 MHz, $CDCl_3$) δ (ppm) = δ 9.14 (t, *J* = 2.2 Hz, 1H, H-13), 9.05 (d, *J* = 2.2 Hz, 2H, H-11), 7.56 – 7.47 (m, 4H, H-Ar), 7.47 – 7.38 (m, 4H, H-Ar), 7.38 – 7.29 (m, 1H, H-Ar), 5.93 – 5.78 (m, 1H, H-5), 5.47 (s, 1H, H-1), 5.18 (s, 1H, H-1), 5.17 – 5.04 (m, 2H, H-6), 4.57 – 4.41 (m, 2H, H-8), 3.01 – 2.82 (m, 1H, H-3), 2.52 – 2.40 (m, 1H, H-4), 2.39 – 2.27 (m, 1H, H-4), 2.23 – 2.10 (m, 1H, H-7), 2.10 – 1.96 (m, 1H, H-7).

^{13}C {^{1}H} NMR (101 MHz, $CDCl_3$) δ (ppm) = 162.5 (C-9), 150.6 (C-2), 148.7 (C-12), 141.6 (C-14), 140.4 (C-17 or C-18), 140.3 (C-17 or C-18), 136.1 (C-5), 134.0 (C-10), 129.4 (C-Ar), 129.0 (C-11), 127.6 (C-Ar), 127.1 (C-Ar), 127.0 (C-Ar), 126.9 (C-Ar), 122.4 (C-13), 117.2 (C-6), 113.4 (C-1), 65.4 (C-8), 40.6 (C-3), 39.6 (C-4), 32.8 (C-7).

HRMS (ESI +**)**: calculated for $C_{27}H_{28}N_3O_6$ $[M+NH_4]^+$: 490.1973; found: 490.1989.

IR ν (neat) (cm^{-1}) = 3106, 2921, 1730, 1628, 1537, 1341, 1280, 1072, 1004, 905, 768, 716.

$[\alpha]^{20}_D$: +40.1° (*c* = 0.5, $CHCl_3$).

m.p.: 79–81 °C.

6. X-ray analysis

Suitable crystals were selected and X-ray intensity data were collected on a. Rigaku XtaLAB Synergy, Dualflex, HyPix-Arc 150° diffractometer using Cu Kα radiation (λ = 1.54184 Å). Using Olex2, the structures were solved with the SHELXT structure solution program using dual space methods and refined with the SHELXL refinement package using least-squares minimization. Summaries of crystal data and structure refinement are given in the following tables.[19-21]

CCDC Number	**2532060**	
Empirical formula	C27 H24 N2 O6	
Formula weight	472.48	
Temperature	120 K	
Wavelength	1.54184 Å	
Crystal system	Monoclinic	
Space group	P 1 21 1	
Unit cell dimensions	a = 6.73693 (7) Å	α= 90°
	b = 21.9971 (3) Å	β= 94.2746(10)°
	c = 7.83072 (9) Å	γ= 90°
Volume	1157.23 (2) $Å^3$	
Z	2	
Density (calculated)	1.356 Mg/m^3	
Absorption coefficient	0.797 mm^{-1}	
F(000)	496	
Crystal size	0.115 x 0.084 x 0.078 mm^3	
Theta range for data collection	4.019 to 73.549°	
Index ranges	-8<=h<=8, -26<=k<=26, -9<=l<=8	
Reflections collected	31684	
Independent reflections	4518 [R(int) = 0.0190]	
Completeness to theta = 67.684°	100.0 %	
Absorption correction	Gaussian	
Max. and min. transmission	1.000 and 0.850	
Refinement method	Full-matrix least-squares on F^2	
Data / restraints / parameters	4518 / 4 / 332	
Goodness-of-fit on F^2	1.056	
Final R indices [I>2sigma(I)]	R1 = 0.0322, wR2 = 0.0863	
R indices (all data)	R1 = 0.0343, wR2 = 0.0879	
Absolute structure parameter	0.14(12)	
Largest diff. peak and hole	0.216 and -0.183 e.$Å^{-3}$	

Table S2. Crystal data and structure refinement for **12a**.

Comments

The resonant scattering is weak for this sample (Friedif 32) as reflected by the large error of the refined Flack parameter (-0.1(2)). However, all indicators confirm the absolute structure. The Hooft parameter is 0.01(3) with P3(true)=1 and the post refinement Parsons-Flack x determined using 1920 quotients is 0.14(12).

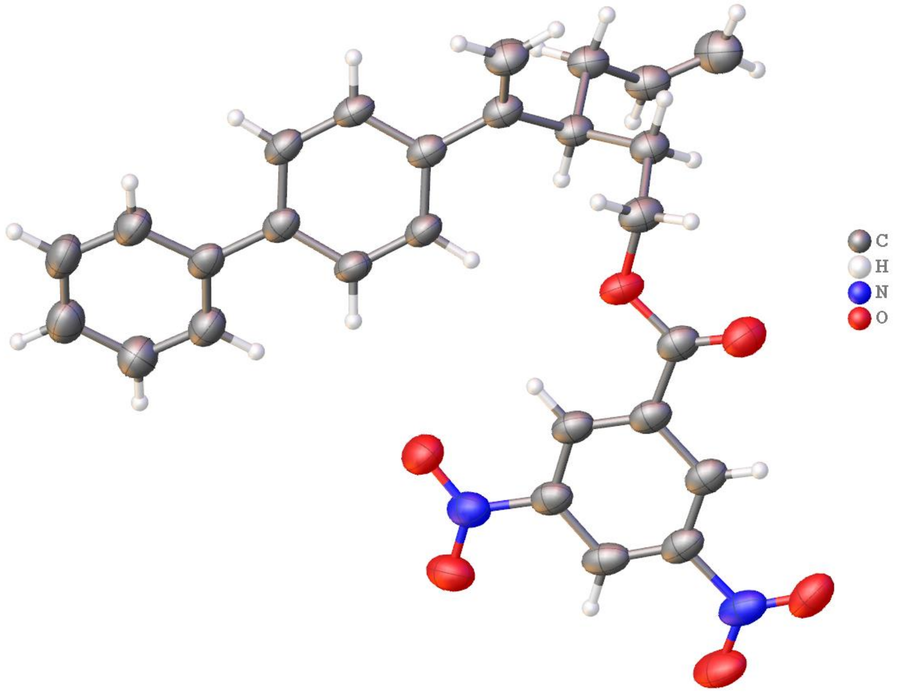

Figure S5. View of the asymmetric unit of **12a.** Displacement ellipsoids drawn at 50 percent probability level.

7. Computational studies

Level of theory. All calculations were carried out using the ORCA 6.1.0 package.[9–11] Geometries were optimized using the UMA-s-1p1 model,[12] with ALPB solvation contributions in THF from xTB version 6.7.1,[13,14] through the "ExtOpt" functionality of ORCA and making use of custom wrapper scripts. These scripts are freely available along with the necessary documentation on Github (github.com/leforesb/ORCA_wrapper_UMA). All stationary points were verified to be minima (zero imaginary frequency) or transition states (one imaginary frequency) by frequency analysis at the same level of theory (numerical hessian). Thermodynamics were computed within the Rigid-Rotor-Harmonic-Oscillator (RRHO) approximation at 253.15K and grouped in a term $G_{corr} = G - E(\text{el})$ where G is the free energy of the species after all thermodynamics corrections and E(el) is the electronic potential energy of the species.

Single point energy corrections were carried out on all geometries using the level of theory targeted by the UMA-s-1p1 model, namely using the ωB97M-V functional with the def2-TZVPD basis set on all atoms.[15–19] The integration grid was set to DefGrid3 defaults, with TightSCF thresholds modified with the block: "%scf THRESH 1e-12 TCUT 1e-13 end".

Conformational Sampling. Conformational searches were run for all species using either the GOAT sampling algorithm as implemented in ORCA 6.1.0, using the UMA-s-1p1 model with ALPB solvation contributions in THF, or CREST with GFN2-xTB and ALPB solvation in THF. For sampling around transition states, the relevant coordinate (*e.g.* bond distance) was constrained during the conformational sampling. For species involving Li cations, the number of explicit THF molecules has also been investigated systematically, up to 3 THF molecules per Li cation. The geometries presented in the full mechanism always feature the number of THF explicit molecules allowing the lowest free energy value. It was shown that solvent coordination-decoordination events and rearrangements are associated with low barriers and fast.[20] The recent racer^{TS} algorithm developed by the Jorner group was also used for conformational sampling around transitions states.[21]

7.1 Substrate speciation

Molecular dynamics parameters. The calculations were carried out with ORCA 6.1.0. The substrate monomer **1a** was centered on (0,0,0) and solvated with 25 explicit THF molecules using the ORCA SOLVATOR's stochastic mode at the GFN2-xTB level of theory. The resulting xyz geometry was placed in a spherical cell centered on (0,0,0) with a radius of 9.78 Å, corresponding to THF's experimental density of *ca*. 0.882 at room temperature. The sphere is bounded by a harmonic wall with a spring strength constant of 50 kJ/mol/Å^2. Initial velocities corresponding to a Boltzmann distribution for 100 K were set, and the system was left to equilibrate with a CSVR thermostat set to 300 K with a time constant of 10 fs. The time step was set to 1.0 fs and the trajectory was run for a total of 50 ps using the UMA-s-1p1 model. The final geometry for **1a** was taken as the last frame from the molecular dynamics simulation, together with the explicit THF molecules nearest Li, and reoptimized using the level of theory described above. Likewise, the solvation sphere of lithium acetate was investigated using 25 THF molecules with a sphere radius of 9.43 Å. A standard state correction of +2.81 kcal/mol was applied to THF, corresponding to the shift from vacuum (assimilated to ideal gas phase at 253.15 K) to the concentration of THF at 253.15 K of approximately 12.9 mol/L. The calculated data is presented in Table S3.

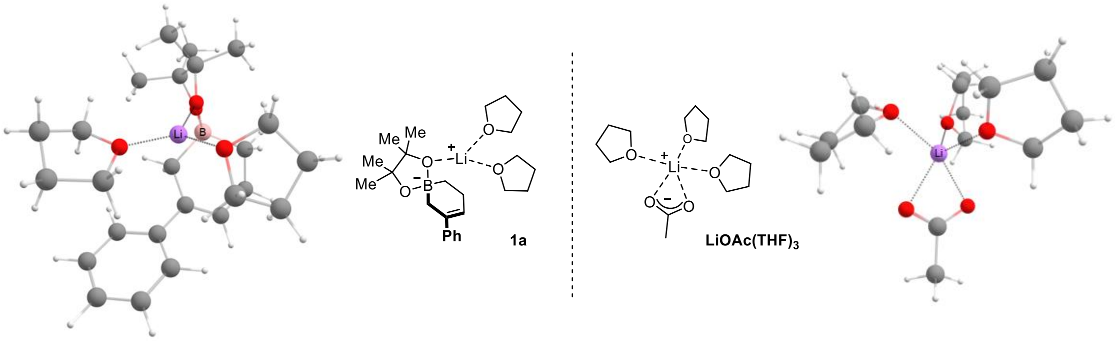

Name	*E*(UMA-s-1p1)	*E*(DFT)	$\Delta G_{\text{solv-ALPB}}$	G_{corr}	*G*(DFT)
**1a**	-1310.091384	-1310.091272	-0.041067	0.571893	-1309.560446
**LiOAc(THF)₃**	-933.461151	-933.461528	-0.040030	0.364804	-933.136753
**THF**	-232.430204	-232.4301977	-0.006883	0.098728[a]	-232.338353

Table S3. Calculated data for substrate **1a** and **LiOAc(THF)$_3$** and **THF**. [a] standard state correction included in this term as +2.81 kcal/mol = 0.004478 Hartree.

The dimer of **1a**, bridged through Li atoms, was similarly placed in a solvated sphere containing 29 explicit THF molecules, with an adapted sphere radius at 10.17 Å. Under the same conditions, the dimer spontaneously split into 2 monomers, each microsolvated with 2 THF solvent molecules per Li atom, confirming the preference for a monomeric state for **1a**.

7.2 Energetics benchmark – Outer sphere path

Early on during the investigation, dramatic changes in relative energy were noted on **TS**$_{\mathbf{C\text{-}D(}\boldsymbol{S}\mathbf{)}}$, with a strong dependance on the functional choice. The relative energy barrier given by various functionals is presented in table S4, across GGA, meta-GGA, hybrid and range-separated hybrid functionals, together with the def2-TZVPD basis set and the VdW correlation functional VV10 for comparability with UMA's training dataset method. The barrier energy value is compared against a DLPNO-CCSD(T) reference value, calculated from a QM1/QM2 protocol. The QM1 region is highlighted in purple in the figure opposite and is associated with the DLPNO-CCSD(T) level of theory with a def2 family basis set (see table), TightSCF and TightPNO thresholds. The QM2 level of theory was chosen as ωB97M-V/def2-TZVPP with TightSCF.

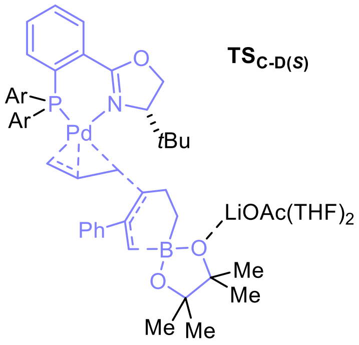

TS$_{\mathbf{C\text{-}D(}\boldsymbol{S}\mathbf{)}}$

Name	E(C)	E(TS$_{C\text{-}D(S)}$)	E(THF)	barrier (kcal/mol)	deviation from ref.
UMA-s-1p1	-3069.637016	-3302.049199	-232.430204	24.8	2.4
BP86-NL	-3068.230141	-3300.544464	-232.306548	8.7	13.8
PBE-NL	-3065.659581	-3297.768148	-232.099396	7.8	14.6
r^{2}SCAN-3c	-3069.237095	-3301.632973	-232.389219	9.4	13.1
TPSS-NL	-3068.940594	-3301.327699	-232.381696	10.1	12.3
TPSS0-NL	-3068.539308	-3300.886229	-232.350906	16.0	6.4
PBE0-NL	-3066.086145	-3298.225427	-232.140263	14.2	8.3
B3LYP-NL	-3066.694229	-3298.885099	-232.190994	13.6	8.8
ωB97M-V	-3069.631923	-3302.047542	-232.430200	22.7	0.3
ωB97M-D3BJ	-3071.651325	-3304.236146	-232.598764	22.3	0.1
DLPNO-CCSD(T) (def2-TZVPP)	-3067.308736	-3299.726862	-232.432756	22.7	0.3
DLPNO-CCSD(T) (def2-QZVPP)	-3067.664715	-3300.083303	-232.432756	22.4	0.0
UMA-s-1p1 + ALPB(THF)	-3069.726236	-3302.134363	-232.437087	31.7	-
ωB97M-V + ALPB(THF)	-3069.721143	-3302.132706	-232.437083	29.6	-
ωB97M-V + CPCM(THF)	-3069.674734	-3302.080058	-232.434674	32.0	-
ωB97M-V + SMD(THF)	-3069.691713	-3302.099574	-232.437121	31.9	-

Table S4. Calculated data for the outer sphere nucleophilic attack barrier via **TS**$_{\mathbf{C\text{-}D(}\boldsymbol{S}\mathbf{)}}$, from the preceding intermediate. Barrier calculated with Gcorr = 0.021572 Hartree added from the relevant thermodynamic corrections.

From table S4, only range-separated functionals (and therefore UMA as model trained on range-separated functional data) are able to reproduce the reference DLPNO-CCSD(T) barrier heights, with regular hybrid functionals underestimating the barrier by 6-8 kcal/mol and GGA/meta-GGA functionals underestimating the barrier even further, by a margin of 12 to 15 kcal/mol. This is likely due to the charge separated nature of intermediate **C** and, $\mathbf{TS}_{\mathbf{C\text{-}D(}\boldsymbol{S}\mathbf{)}}$. In that context, functionals lacking long-range exact exchange are likely to over-stabilize such structures due to exaggerated delocalization/self-interaction error.

Alternatively, such strong functional dependence may be a sign of multireference character. While FOD analysis gave N_{FOD}= 0.85 and 0.96 for **C** and $\mathbf{TS}_{\mathbf{C\text{-}D(}\boldsymbol{S}\mathbf{)}}$, respectively, these high values are likely a reflection of the same exaggerated delocalization yielded by the TPSS functional. Indeed, multireference character could not be confirmed by any other means, with no sign of difficult SCF convergence, low T1 diagnostics (< 0.013; i.e. HF reference is not inadequate) and a tentative CAS(6,6) calculation yielding a purely single reference ground state.

7.3 Pd-catalyzed C3-allylation pathway

7.3.1 Main mechanism – outer sphere path and C1-allylation

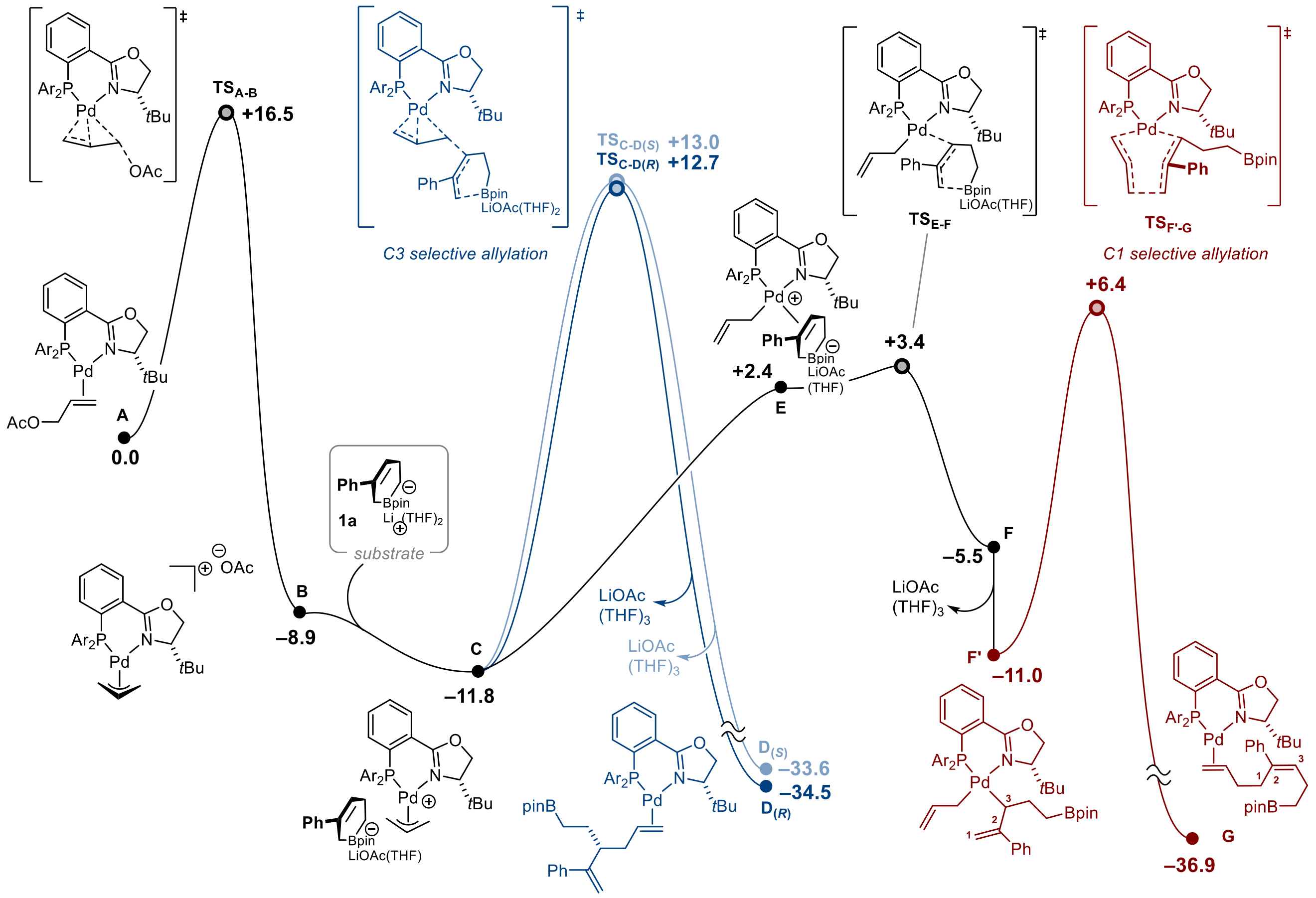

Name	*E*(UMA-s-1p1)	*E*(DFT)	$\Delta G_{solv\text{-}ALPB}$	G_{corr}	*G*(DFT)
**A**	-1991.958994	-1991.956837	-0.044091	0.577662	-1991.423266
**TS$_{A\text{-}B}$**	-1991.916165	-1991.914003	-0.057554	0.574594	-1991.396963
**B**	-1991.944646	-1991.943770	-0.067520	0.573832	-1991.437458
**C**	-3069.637016	-3069.631916	-0.089219	1.057013	-3068.664122
**TS$_{C\text{-}D(R)}$**	-3302.042745	-3302.041090	-0.090197	1.167836	-3300.963451
**TS$_{C\text{-}D(S)}$**	-3302.049199	-3302.047541	-0.085164	1.169737	-3300.962969
**D$_{(R)}$**	-2601.077400	-2601.075323	-0.059488	0.894459	-2600.240352
**D$_{(S)}$**	-2601.075555	-2601.073166	-0.060083	0.894323	-2600.238927
**E**	-3069.619906	-3069.617134	-0.081785	1.057421	-3068.641498
**TS$_{E\text{-}F}$**	-3069.622568	-3069.620368	-0.074728	1.055202	-3068.639894
**F**	-3069.638520	-3069.635650	-0.073138	1.054614	-3068.654174
**F’**	-2601.047552	-2601.045298	-0.051722	0.894158	-2600.202862
**TS$_{F'\text{-}G}$**	-2601.009464	-2601.014625	-0.053500	0.892953	-2600.175173
**G**	-2601.080047	-2601.077038	-0.060858	0.893718	-2600.244177

Table S5. Calculated data for the outer sphere C3-allylation pathway of **1a** and alternative ring-opening activation path.

7.3.2 Isomerism of intermediate B

The following scheme shows the interconversion pathways between cationic Pd-allyl complex **B** and its allyl-flipped isomer $\mathbf{B}_{\mathbf{up}}$, 0.8 kcal/mol above. Reversible coordination of the acetate counterion is facile and nearly isoenergetic. Of note, the hemilability of the oxazoline moiety can be seen here to be slightly unfavorable, but not inaccessible, as illustrated by intermediate $\mathbf{B}_{\mathbf{hemi}}$, located at –1.3 kcal/mol.

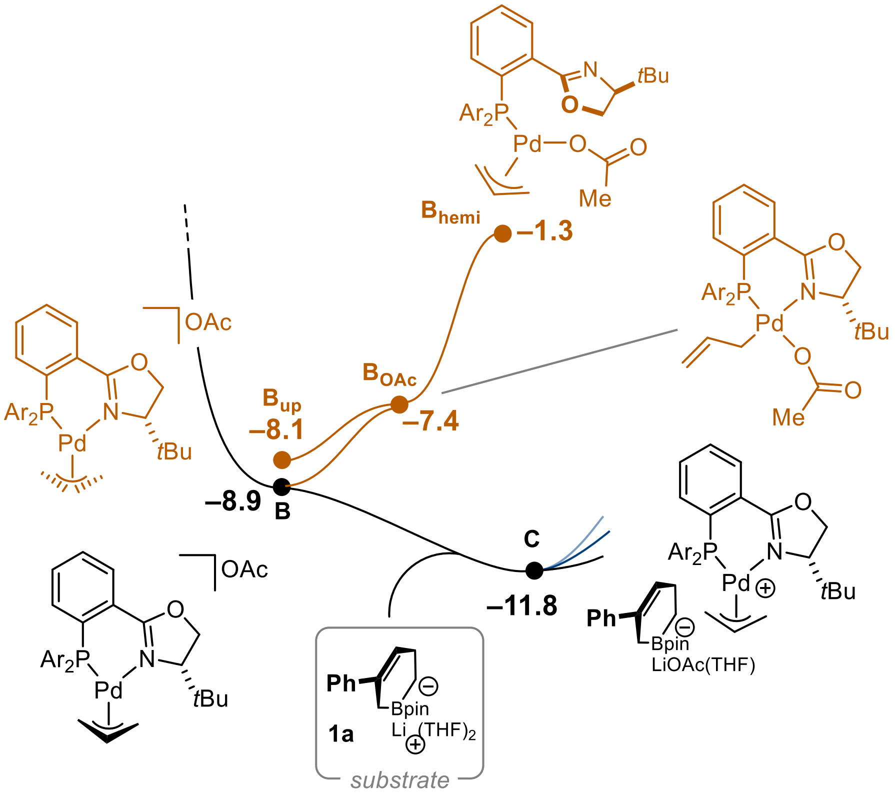

Name		*E*(UMA-s-1p1)	*E*(DFT)	Δ$G_{solv\text{-}ALPB}$	G_{corr}	*G*(DFT)
$\mathbf{B}_{\mathbf{up}}$		-1991.942703	-1991.940872	-0.070556	0.575228	-1991.436200
$\mathbf{B}_{\mathbf{OAc}}$		-1991.956960	-1991.955797	-0.055755	0.576484	-1991.435067
$\mathbf{B}_{\mathbf{hemi}}$		-1991.948202	-1991.948183	-0.052731	0.575496	-1991.425418

Table S6. Calculated data for the isomerism possibilities of intermediate **B**.

7.3.3 Alternative activation of 1a

The following scheme shows the alternative activation pathways considered in this work for substrate **1a**. Namely, electrophilic substitution and ring-opening transition state **TS$_{C-K2}$** at +15.1 kcal/mol, direct nucleophilic attack at C3 with preliminary loss of lithium acetate via **TS$_{C-D(R)-nosalt}$** at +20.1 kcal/mol and activation via coordination *cis* to the phosphine prior to ring-opening via intermediate **E$_{cis}$** at +17.0 kcal/mol. None of these options compete with the preferred path, occurring via *trans* coordination of the substrate and ring-opening through **TS$_{E-F}$**.

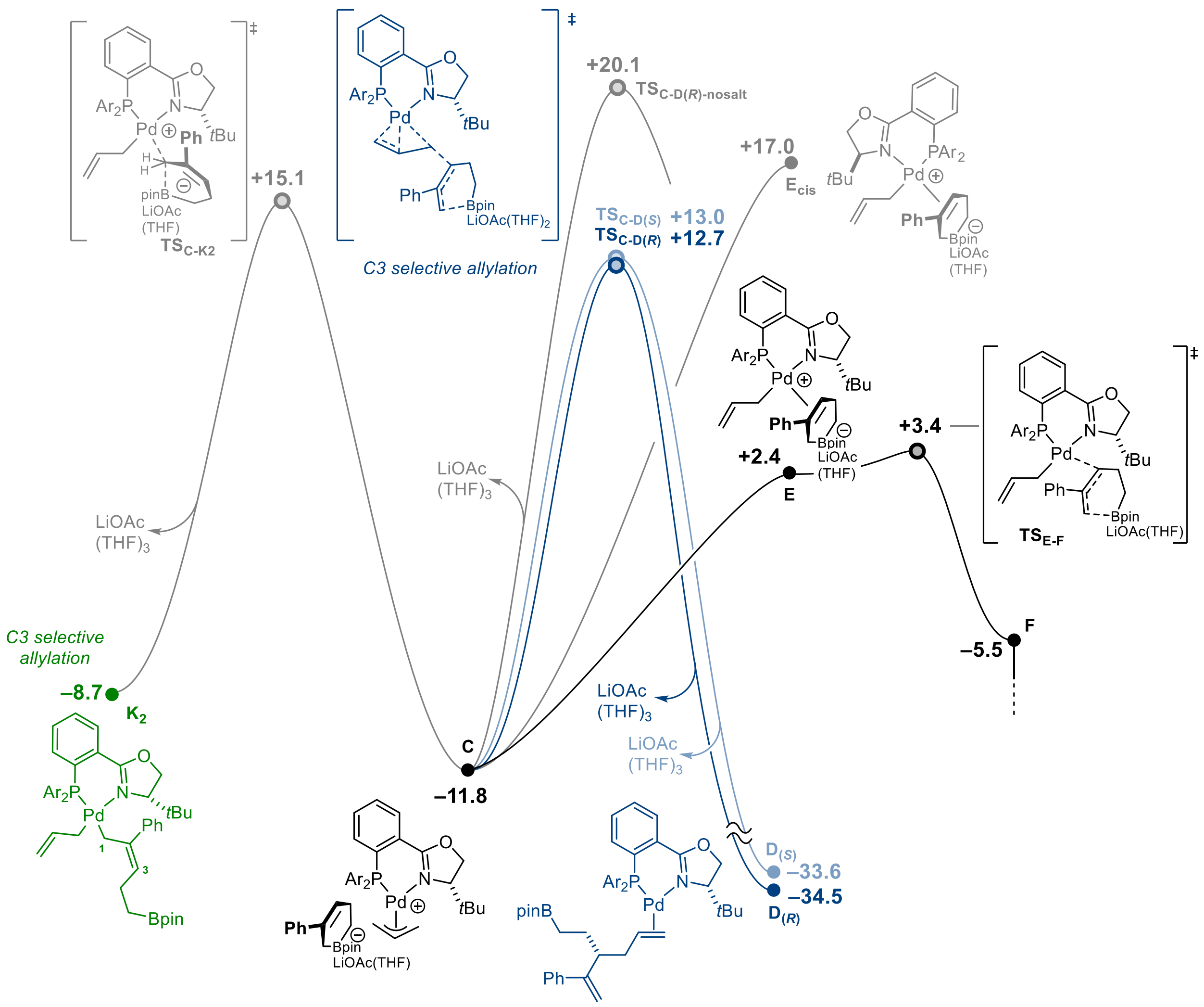

Name	*E*(UMA-s-1p1)	*E*(DFT)	Δ*G*$_{solv-ALPB}$	*G*$_{corr}$	*G*(DFT)
**E$_{cis}$**	-3069.598598	-3069.596867	-0.081067	1.059646	-3068.618289
**TS$_{C-K2}$**	-3069.611439	-3069.607576	-0.071682	1.057972	-3068.621286
**TS$_{C-D(R)-nosalt}$**	-2600.979760	-2600.975900	-0.069051	0.891602	-2600.153350

Table S7. Calculated data for the alternative activation path of boronate salt **1a** via electrophilic substitution, coordination *cis* to the phosphine or loss of lithium acetate followed by direct nucleophilic attack.

7.3.4 Main mechanism – inner sphere path and isomerization

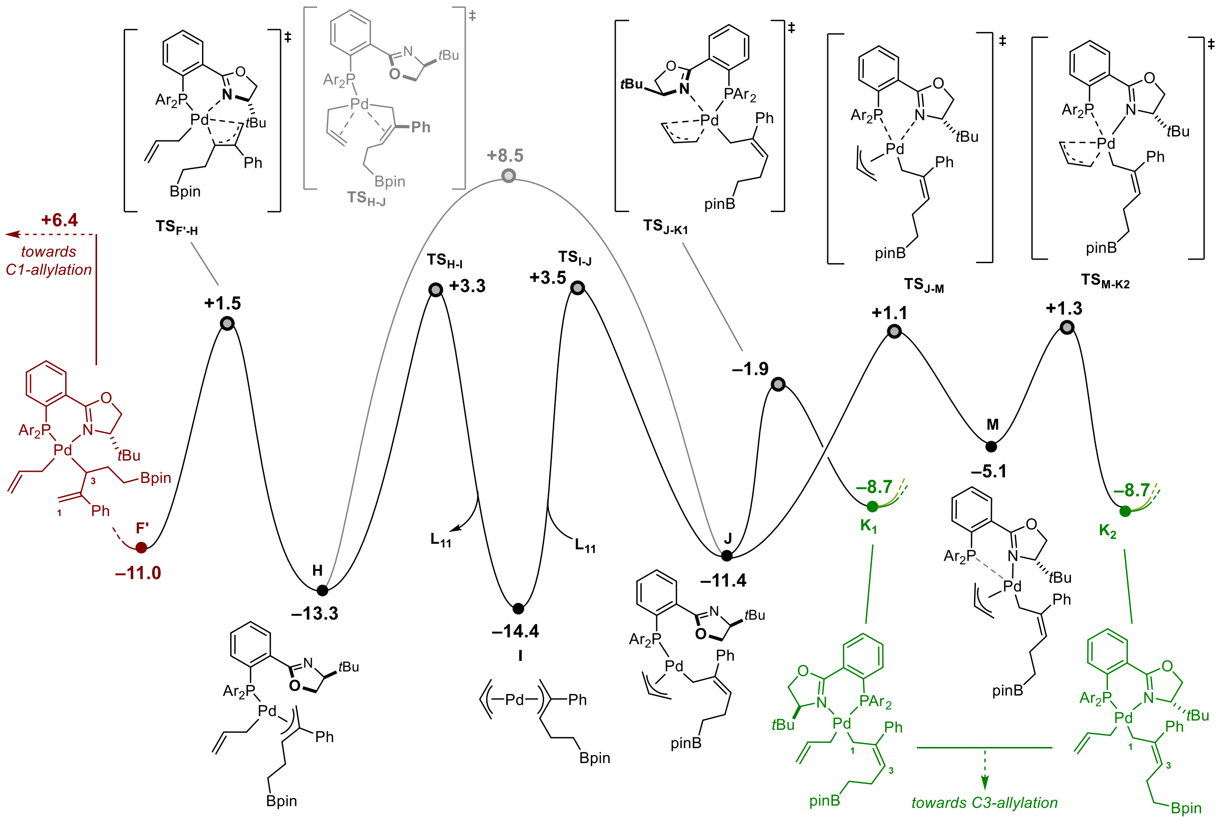

Name		*E*(UMA-s-1p1)	*E*(DFT)	Δ$G_{solv\text{-}ALPB}$	G_{corr}	*G*(DFT)
**F'**		-2601.047552	-2601.045298	-0.051722	0.894158	-2600.202862
**TS$_{F'\text{-}H}$**		-2601.017591	-2601.016587	-0.057332	0.890977	-2600.182941
**H**		-2601.040784	-2601.041340	-0.056328	0.891231	-2600.206437
**TS$_{H\text{-}J}$**		-2601.009710	-2601.005433	-0.058013	0.891630	-2600.171817
**TS$_{H\text{-}I}$**		-2601.019009	-2601.020602	-0.052990	0.893533	-2600.180059
**I**		-1082.781722	-1082.781605	-0.030334	0.408267	-1082.403672
**L$_{11}$**		-1518.235190	-1518.235171	-0.030911	0.461550	-1517.804532
**TS$_{I\text{-}J}$**		-2601.019916	-2601.020134	-0.052714	0.893058	-2600.179790
**J**		-2601.042114	-2601.041174	-0.054572	0.892317	-2600.203429
**TS$_{J\text{-}K1}$**		-2601.027948	-2601.027016	-0.053306	0.892000	-2600.188322
**K$_1$**		-2601.039035	-2601.037830	-0.055260	0.893888	-2600.199202
**TS$_{J\text{-}M}$**		-2601.023223	-2601.023498	-0.053860	0.893806	-2600.183553
**M**		-2601.031188	-2601.030496	-0.053763	0.890879	-2600.193379
**TS$_{M\text{-}K2}$**		-2601.019609	-2601.019605	-0.055501	0.891846	-2600.183260
**K$_2$**		-2601.040581	-2601.039321	-0.053880	0.894044	-2600.199157

Table S8. Calculated data for the isomerism pathways between pro-C1 allylation intermediate **F'** and pro-C3 allylation intermediates **K$_1$** and **K$_2$**.

7.3.5 Main mechanism – pro-C3, 3-3' reductive elimination TS

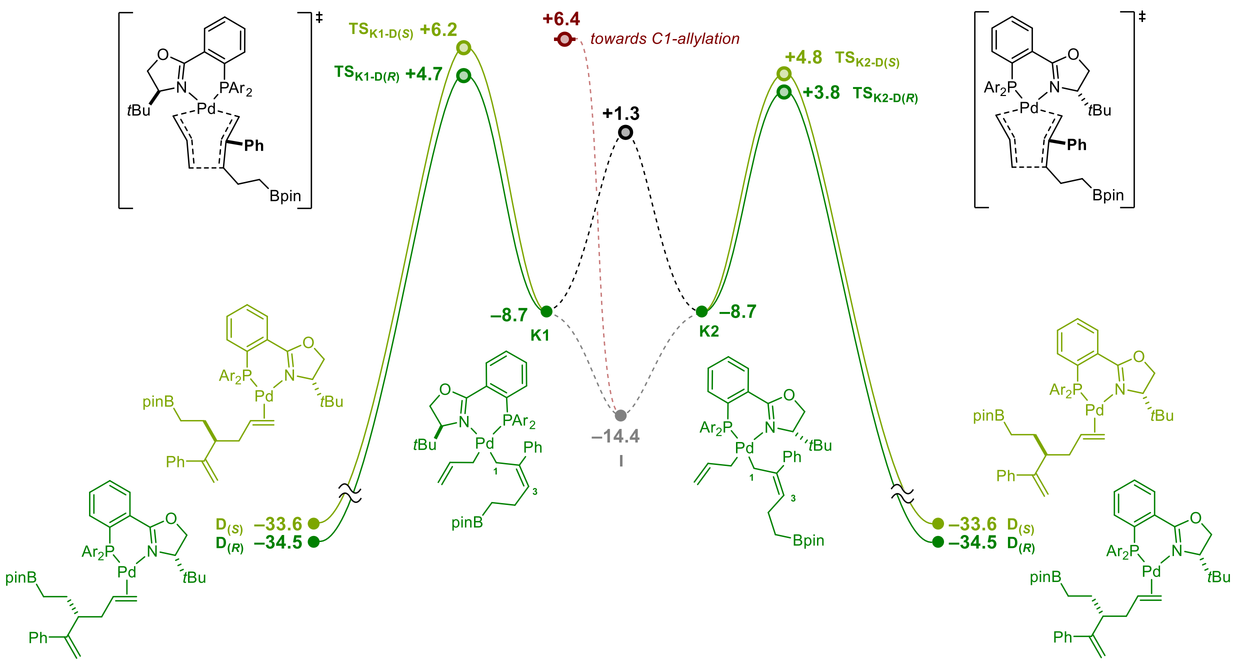

Name		*E*(UMA-s-1p1)	*E*(DFT)	Δ*G*$_{solv-ALPB}$	*G*$_{corr}$	*G*(DFT)
**I**		-1082.781722	-1082.781605	-0.030334	0.408267	-1082.403672
**K$_1$**		-2601.039035	-2601.037830	-0.055260	0.893888	-2600.199202
**TS$_{K1-D(R)}$**		-2601.012324	-2601.018037	-0.054349	0.894570	-2600.177816
**TS$_{K1-D(S)}$**		-2601.010974	-2601.016878	-0.051600	0.893002	-2600.175476
**K$_2$**		-2601.040581	-2601.039321	-0.053880	0.894044	-2600.199157
**TS$_{K2-D(R)}$**		-2601.013411	-2601.019601	-0.054023	0.894344	-2600.179279
**TS$_{K2-D(S)}$**		-2601.011591	-2601.020177	-0.052899	0.895378	-2600.177697
**D$_{(R)}$**		-2601.077400	-2601.075323	-0.059488	0.894459	-2600.240352
**D$_{(S)}$**		-2601.075555	-2601.073166	-0.060083	0.894323	-2600.238927

Table S9. Calculated data for the pro-C3, 3-3' reductive elimination transition states from intermediates **K$_1$** and **K$_2$**.

7.3.6 Alternative reductive elimination transition states

The following scheme shows the 1-3 and 3-1 reductive elimination transition states from intermediates **H** and **J**, respectively, as alternatives to the more favorable 3-3' transition states as presented in the main manuscript. Note that 1-1' reductive elimination transition states could not be found either from **F'**, **K₁** or **K₂**, all attempts resulting in decoordination of the oxazoline moiety and converging back to a 1-3 or 3-1 reductive elimination topology.

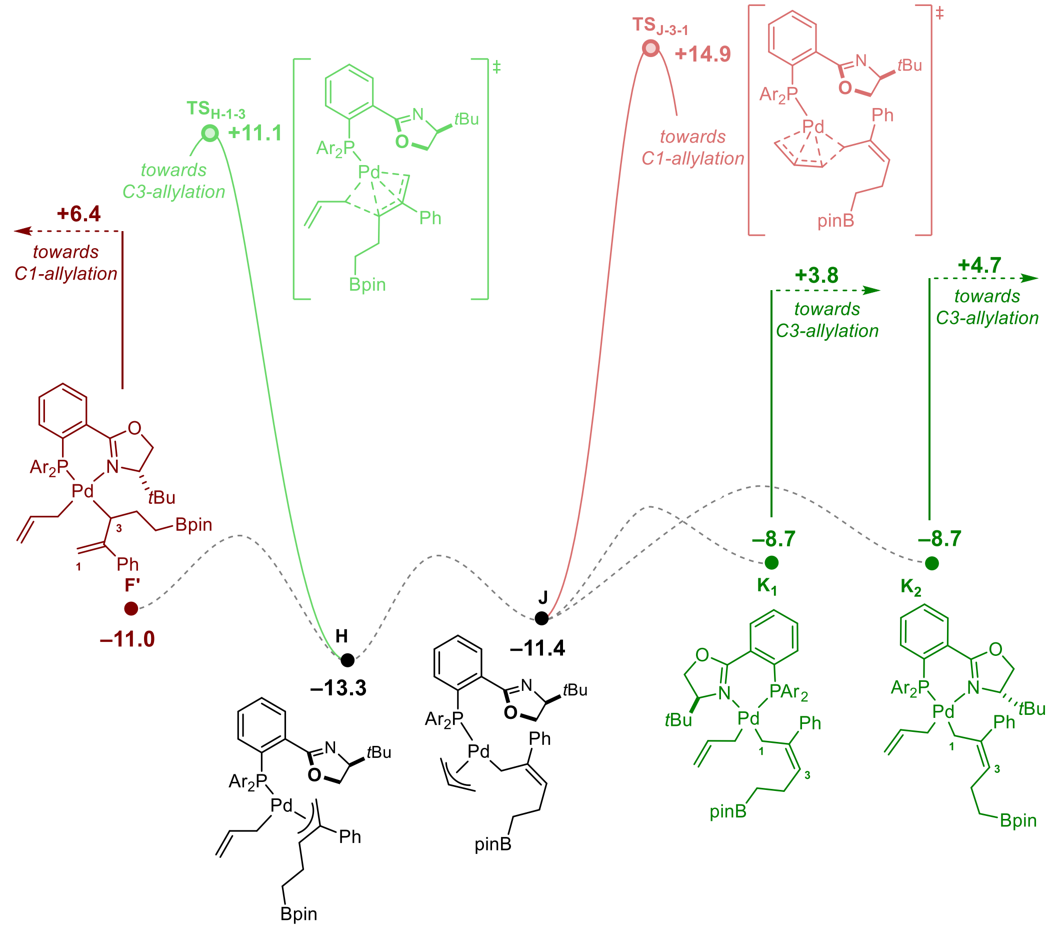

Name	*E*(UMA-s-1p1)	*E*(DFT)	Δ*G*$_{solv\text{-}ALPB}$	*G*$_{corr}$	*G*(DFT)
**TS**$_{\mathbf{H\text{-}3\text{-}1}}$	-2600.997112	-2600.999975	-0.057145	0.889439	-2600.167681
**TS**$_{\mathbf{J\text{-}1\text{-}3}}$	-2600.995352	-2600.996071	-0.057645	0.892225	-2600.161490

Table S10. Calculated data for the 1-3 and 3-1 reductive elimination transition states from intermediates **H** and **J**, respectively.

7.3.7 Closing the catalytic cycle

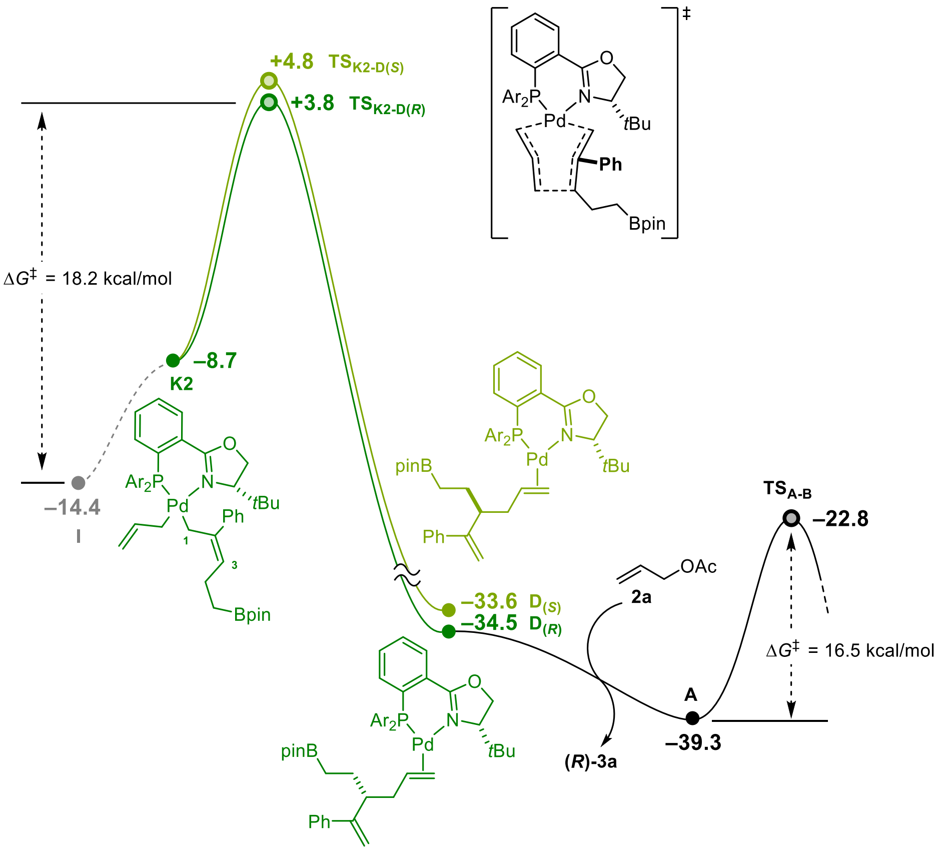

Name	*E*(UMA-s-1p1)	*E*(DFT)	$\Delta G_{solv\text{-}ALPB}$	G_{corr}	*G*(DFT)
**2a**	-345.7840978	-345.7841174	-0.014073	0.096588	-345.701603
**(*R*)-3a**	-954.9137184	-954.9133634	-0.026346	0.413433	-954.526276

Table S11. Calculated data for the product (**(*R*)-3a**) – substrate (**2a**) exchange from $\mathbf{D}_{(R)}$.

7.4 Monitored deviation between UMA-s-1p1 and DFT

Name	*E*(UMA-s-1p1)	*E*(DFT)	relative dev. (kcal/mol)	absolute dev. (kcal/mol)
**1a**	-1310.091384	-1310.091272	-0.1	0.1
**LiOAc(THF)$_3$**	-933.461151	-933.461528	0.2	0.2
**THF**	-232.430204	-232.430198	0.0	0.0
**A**	-1991.958994	-1991.956837	-1.4	1.4
**TS$_{A\text{-}B}$**	-1991.916165	-1991.914003	-1.4	1.4
**B**	-1991.944646	-1991.943770	-0.5	0.5
**C**	-3069.637016	-3069.631916	-3.2	3.2
**TS$_{C\text{-}D(R)}$**	-3302.042745	-3302.041090	-1.0	1.0
**TS$_{C\text{-}D(S)}$**	-3302.049199	-3302.047541	-1.0	1.0
**D$_{(R)}$**	-2601.077400	-2601.075323	-1.3	1.3
**D$_{(S)}$**	-2601.075555	-2601.073166	-1.5	1.5
**E**	-3069.619906	-3069.617134	-1.7	1.7
**TS$_{E\text{-}F}$**	-3069.622568	-3069.620368	-1.4	1.4
**F**	-3069.638520	-3069.635650	-1.8	1.8
**F’**	-2601.047552	-2601.045298	-1.4	1.4
**TS$_{F'\text{-}G}$**	-2601.009464	-2601.014625	3.2	3.2
**G**	-2601.080047	-2601.077038	-1.9	1.9
**B$_{up}$**	-1991.942703	-1991.940872	-1.1	1.1
**B$_{OAc}$**	-1991.956960	-1991.955797	-0.7	0.7
**B$_{hemi}$**	-1991.948202	-1991.948183	0.0	0.0
**E$_{cis}$**	-3069.598598	-3069.596867	-1.1	1.1
**TS$_{C\text{-}K2}$**	-3069.611439	-3069.607576	-2.4	2.4
**TS$_{C\text{-}D(R)\text{-}nosalt}$**	-2600.979760	-2600.975900	-2.4	2.4
**TS$_{F'\text{-}H}$**	-2601.017591	-2601.016587	-0.6	0.6
**H**	-2601.040784	-2601.041340	0.3	0.3
**TS$_{H\text{-}J}$**	-2601.009710	-2601.005433	-2.7	2.7
**TS$_{H\text{-}I}$**	-2601.019009	-2601.020602	1.0	1.0
**I**	-1082.781722	-1082.781605	-0.1	0.1
**L$_{11}$**	-1518.235190	-1518.235171	0.0	0.0
**TS$_{I\text{-}J}$**	-2601.019916	-2601.020134	0.1	0.1
**J**	-2601.042114	-2601.041174	-0.6	0.6
**TS$_{J\text{-}K1}$**	-2601.027948	-2601.027016	-0.6	0.6
**K$_1$**	-2601.039035	-2601.037830	-0.8	0.8
**TS$_{J\text{-}M}$**	-2601.023223	-2601.023498	0.2	0.2
**M**	-2601.031188	-2601.030496	-0.4	0.4
**TS$_{M\text{-}K2}$**	-2601.019609	-2601.019605	0.0	0.0
**K$_2$**	-2601.040581	-2601.039321	-0.8	0.8
**TS$_{K1\text{-}D(R)}$**	-2601.012324	-2601.018037	3.6	3.6
**TS$_{K1\text{-}D(S)}$**	-2601.010974	-2601.016878	3.7	3.7
**TS$_{K2\text{-}D(R)}$**	-2601.013411	-2601.019601	3.9	3.9
**TS$_{K2\text{-}D(S)}$**	-2601.011591	-2601.020177	5.4	5.4
**TS$_{H\text{-}3\text{-}1}$**	-2600.997112	-2600.999975	1.8	1.8
**TS$_{J\text{-}1\text{-}3}$**	-2600.995352	-2600.996071	0.5	0.5
**2a**	-345.7840978	-345.7841174	0.0	0.0
**(*R*)-3a**	-954.9137184	-954.9133634	-0.2	0.2

Table S11. Deviation of the calculated energies with UMA-s-1p1 versus the reference DFT level ωB97M-V/def2-TZVPD in kcal/mol.

8. References

1 S. S. Zalesskiy and V. P. Ananikov, Pd2(dba)3 as a Precursor of Soluble Metal Complexes and Nanoparticles: Determination of Palladium Active Species for Catalysis and Synthesis. *Organometallics* 2012, **31**, 2302–2309.

2 J. Garcia-Fortanet and S. L. Buchwald, Asymmetric Palladium-Catalyzed Intramolecular α-Arylation of Aldehydes. *Angew. Chem., Int. Ed.* 2008, **47**, 8108–8111.

3 P. Mátyus, P. Huleatt, C. L. L. Chai, B. Sperlágh, M. L. Khoo, K. Magyar, Á. Papp-Behr, R. Deme, G. Túrós and K. Gyires, New Arylalkenylpropargylamine Derivatives Exhibiting Neuroprotective Action for the Treatment of Neurodegenerative Diseases. WO2015087094A1, 2015.

4 Katsina, T.; Sharma, S. P.; Buccafusca, R.; Quinn, D. J.; Moody, T. S.; Arseniyadis, S. Sequential Palladium-Catalyzed Allylic Alkylation/retro-Dieckmann Fragmentation Strategy for the Synthesis of α-Substituted Acrylonitriles. *Org. Lett*. 2019, **21**, 9348–9352.

5 C. Zhang, and C. Mazet, Access to Cyclic Borates by Cu-Catalyzed Borylation of Unactivated Vinylcyclopropanes. *Org. Lett*. 2024, **26**, 5386–5390.

6 O. V. Dolomanov, L. J. Bourhis, R. J. Gildea, J. A. K. Howard, and H. Puschmann, OLEX2: A Complete Structure Solution, Refinement and Analysis Program. *J. Appl. Crystallogr.* 2009, **42**, 339–341.

7 G. M. Sheldrick, SHELXT - Integrated Space-Group and Crystal-Structure Determination. *Acta Cryst. A* 2015, **71**, 3–8.

8 G. M. Sheldrick, Crystal Structure Refinement with SHELXL. *Acta Cryst. C* 2015, **71**, 3–8.

9 F. Neese, The ORCA Program System. *WIREs Comput. Mol. Sci.*, 2012, **2**, 73–78.

10 F. Neese, Software Update: The ORCA Program System—Version 5.0. *WIREs Comput. Mol. Sci.*, 2022, **12**, e1606.

11 F. Neese, Software Update: The ORCA Program System—Version 6.0. *WIREs Comput. Mol. Sci.*, 2025, **15**, e70019.

12 B. M. Wood, M. Dzamba, X. Fu, M. Gao, M. Shuaibi, L. Barroso-Luque, K. Abdelmaqsoud, V. Gharakhanyan, J. R. Kitchin, D. S. Levine, K. Michel, A. Sriram, T. Cohen, A. Das, A. Rizvi, S. J. Sahoo, Z. W. Ulissi and C. L. Zitnick, UMA: A Family of Universal Models for Atoms. arXiv 2025, arXiv:2506.23971.

13 C. Bannwarth, S. Ehlert, and S. Grimme, GFN2-xTB—An Accurate and Broadly Parametrized Self-Consistent Tight-Binding Quantum Chemical Method with Multipole Electrostatics and Density-Dependent Dispersion Contributions. *J. Chem. Theory Comput.*, 2019, **15**, 1652–1671.

14 S. Ehlert, M. Stahn, S. Spicher and S. Grimme, Robust and Efficient Implicit Solvation Model for Fast Semiempirical Methods. *J. Chem. Theory Comput.*, 2021, **17**, 4250–4261.

15 O. A. Vydrov and T. Van Voorhis, Nonlocal van der Waals Density Functional: The Simpler the Better. *J. Chem. Phys.*, 2010, **133**, 244103.

16 W. Hujo and S. Grimme, Performance of the van der Waals Density Functional VV10 and (Hybrid)GGA Variants for Thermochemistry and Noncovalent Interactions. *J. Chem. Theory Comput.*, 2011, **7**, 3866–3871.

17 N. Mardirossian and M. Head-Gordon, ωB97M-V: A Combinatorially Optimized, Range-Separated Hybrid, Meta-GGA Density Functional with VV10 Nonlocal Correlation. *J. Chem. Phys.*, 2016, **144**, 214110.

18 F. Weigend and R. Ahlrichs, Balanced Basis Sets of Split Valence, Triple Zeta Valence and Quadruple Zeta Valence Quality for H to Rn: Design and Assessment of Accuracy. *Phys. Chem. Chem. Phys.*, 2005, **7**, 3297–3305.

19 F. Weigend, Accurate Coulomb-Fitting Basis Sets for H to Rn. *Phys. Chem. Chem. Phys.*, 2006, **8**, 1057–1065.

20 M. de Giovanetti, S. H. Hopen Eliasson, S. L. Bore, O. Eisenstein and M. Cascella, Morphology of Lithium Halides in Tetrahydrofuran from Molecular Dynamics with Machine Learning Potentials. *Chem. Sci.* 2024, **15**, 20355–20364.

21 S. P. Schmid, H. Seng, T. Kläy and K. Jorner, Rapid Generation of Transition-State Conformer Ensembles via Constrained Distance Geometry. *J. Chem. Inf. Model.* 2026, DOI: 10.1021/acs.jcim.5c02794 (Published online Feb 12, 2026).

9. NMR spectra

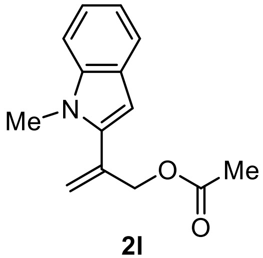

2l

^{1}H NMR (400 MHz, 298 K, $CDCl_3$)

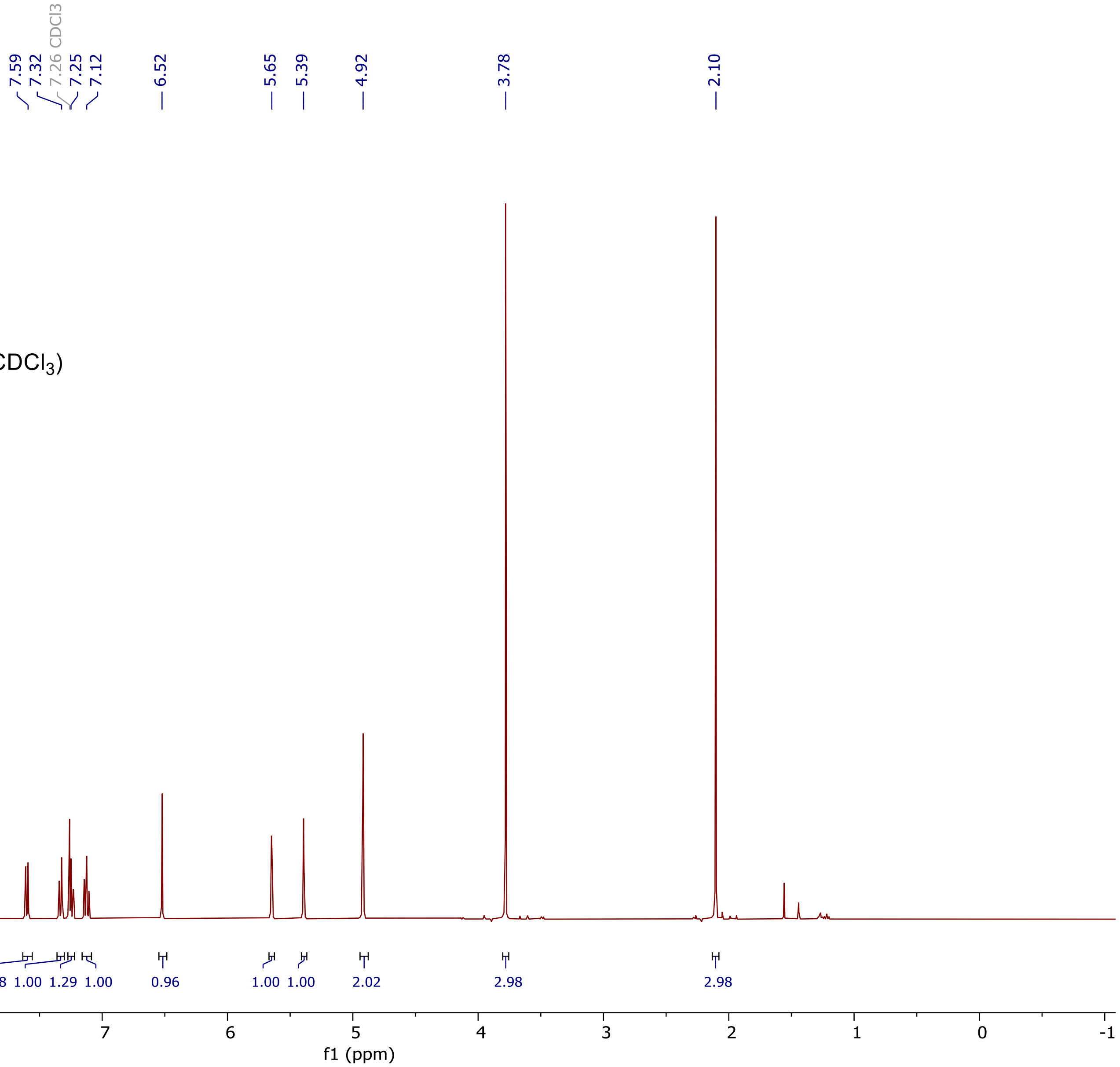

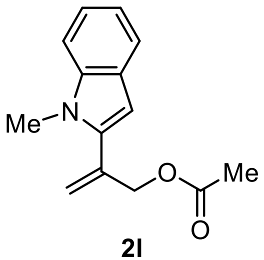

2l

$^{13}C\{^{1}H\}$ NMR (101 MHz, 298 K, $CDCl_3$)

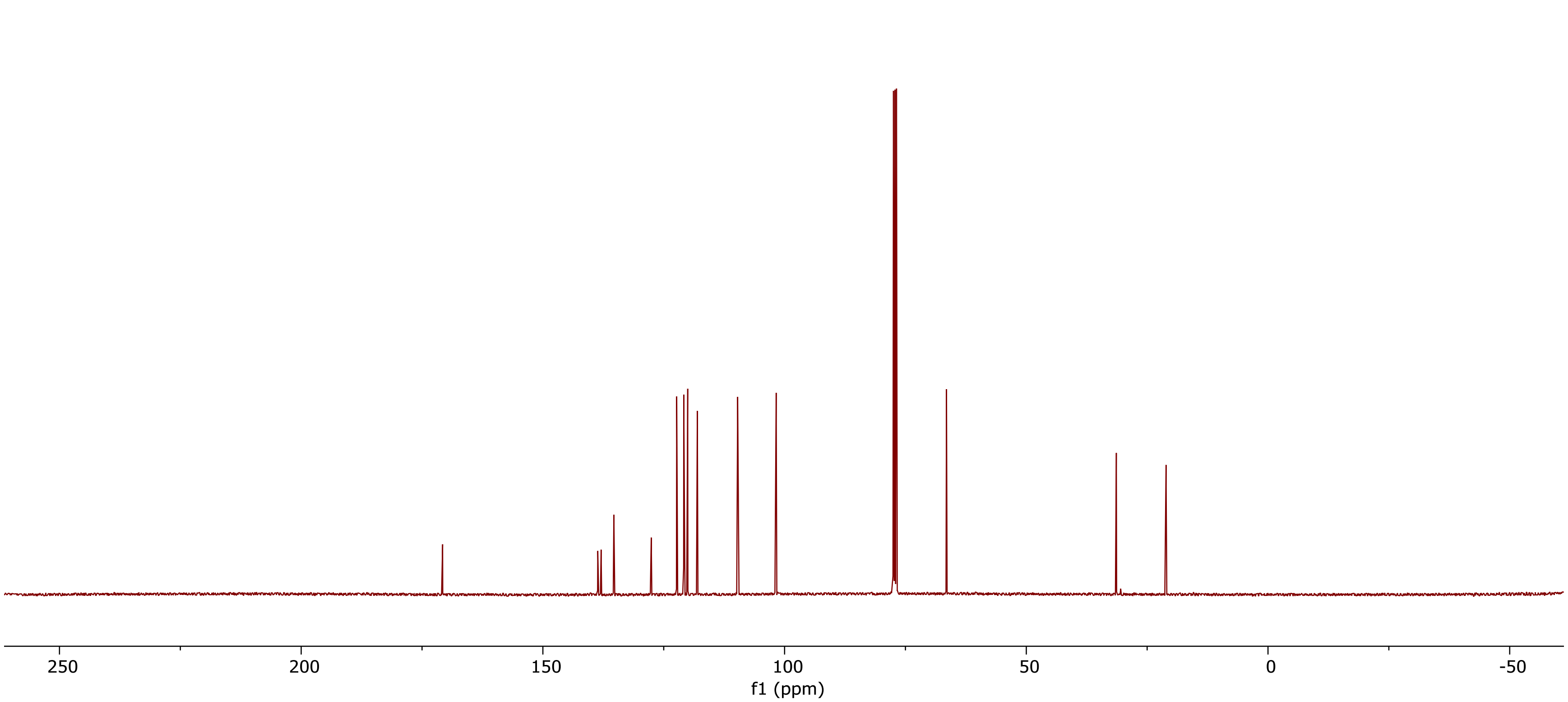

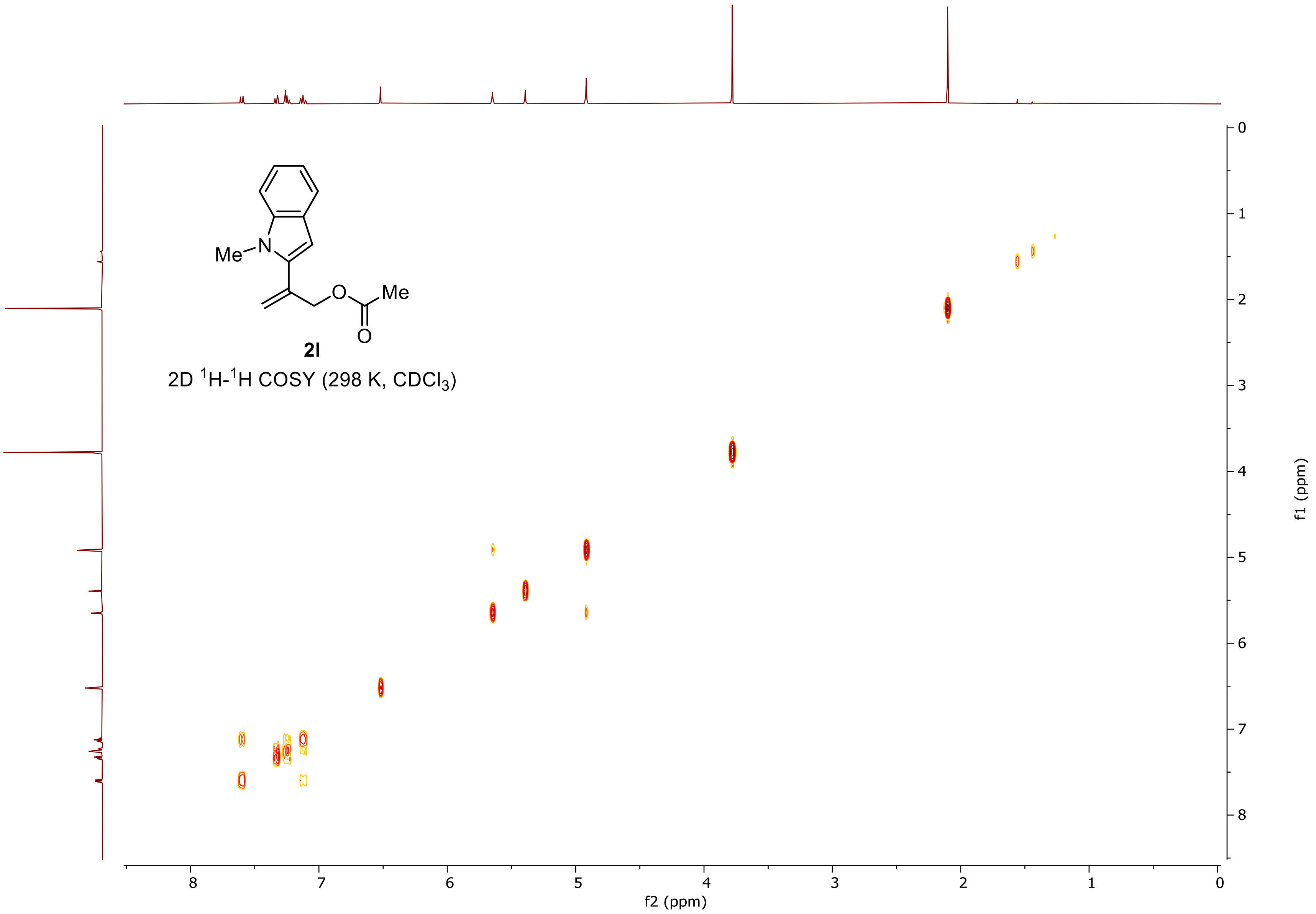
Me
N
O
Me
O
2l
2D ¹H-¹H COSY (298 K, CDCl3)
f1 (ppm)
f2 (ppm)
0
1
2
3
4
5
6
7
8

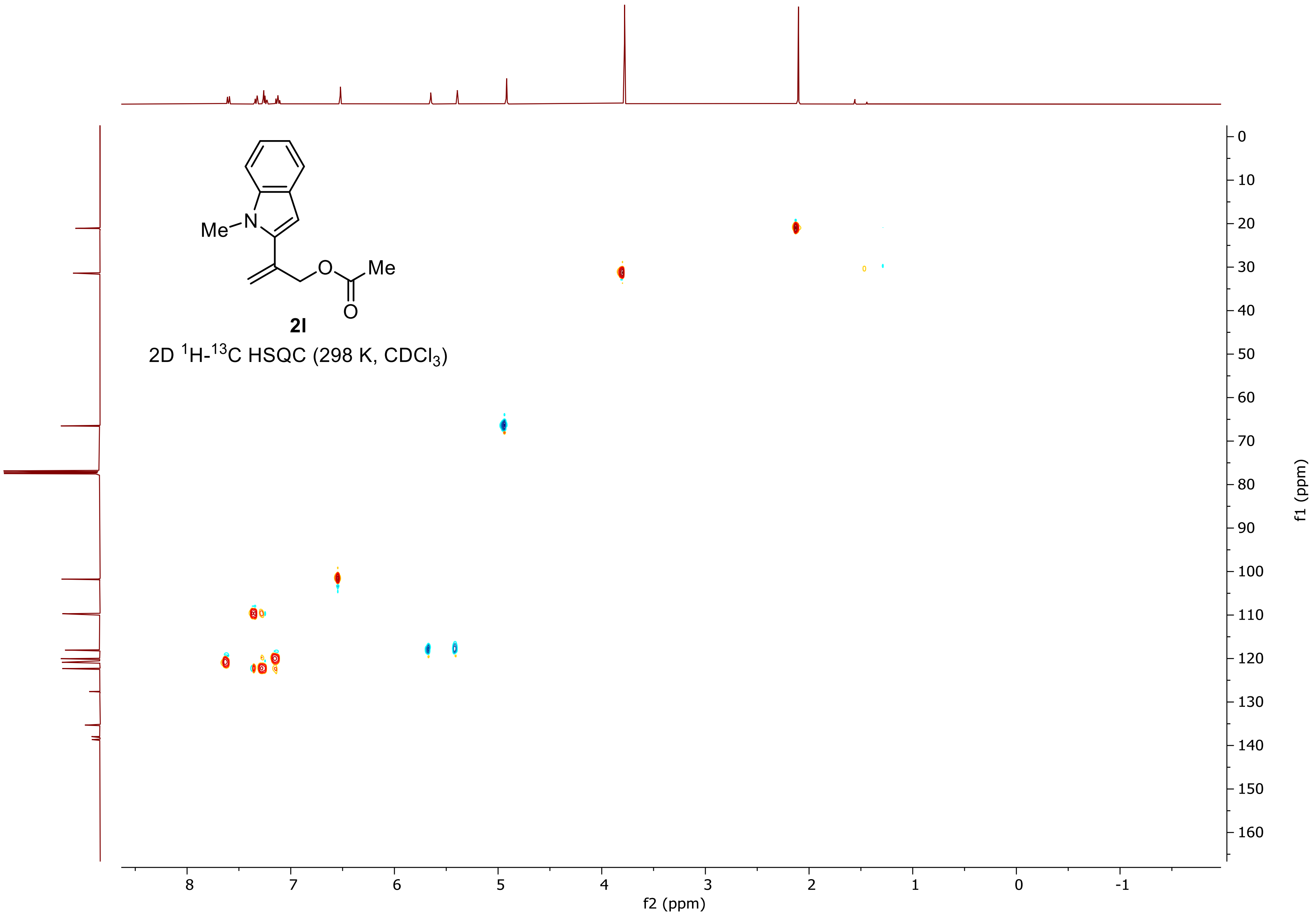
Me
N
O
Me
O
2l
2D 1H-13C HSQC (298 K, CDCl3)
f2 (ppm)
f1 (ppm)

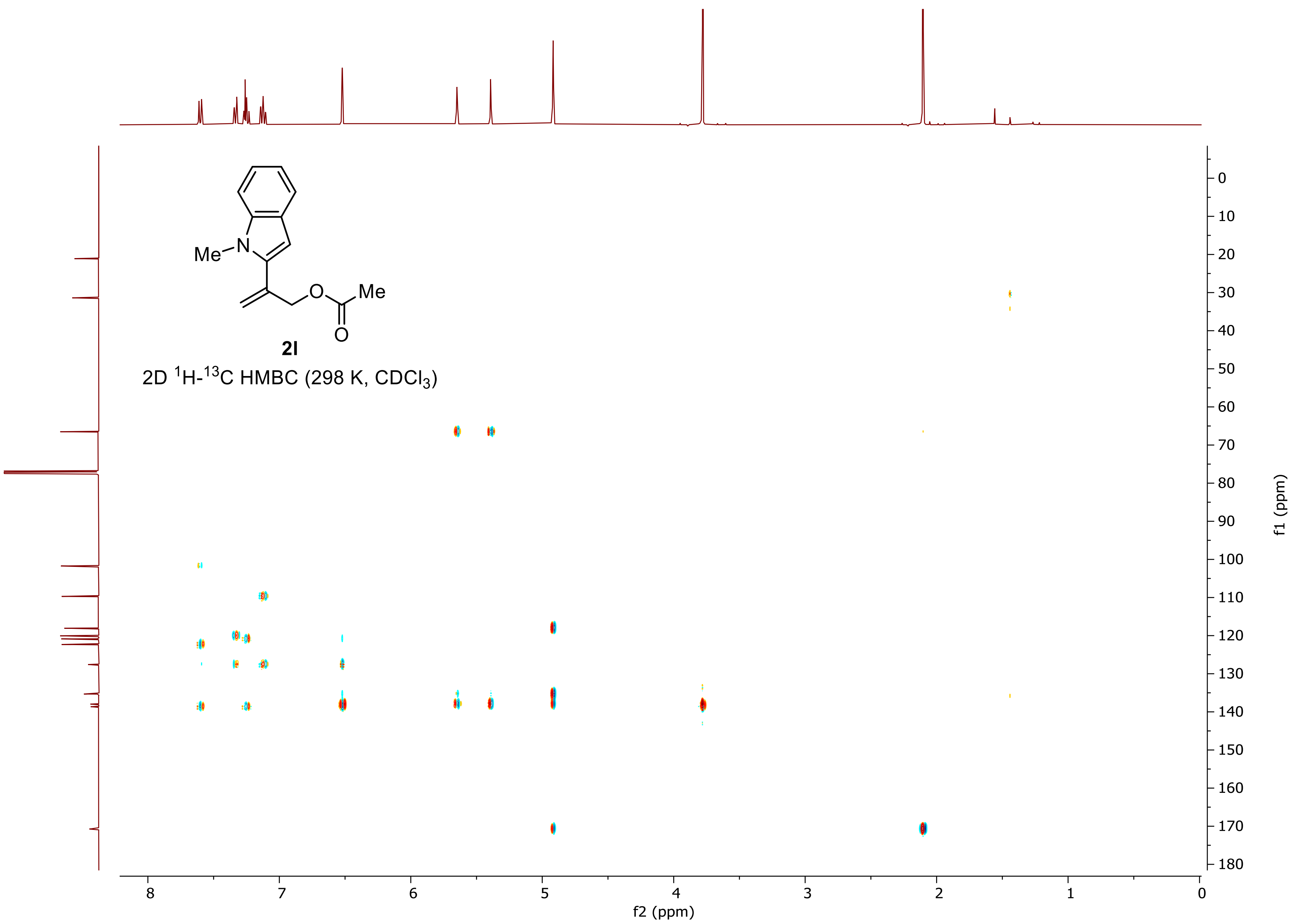

2l

2D ^{1}H-^{13}C HMBC (298 K, $CDCl_3$)

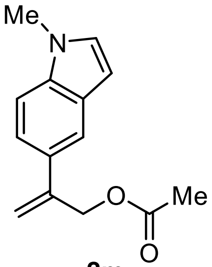

2m

^{1}H NMR (400 MHz, 298 K, $CDCl_3$)

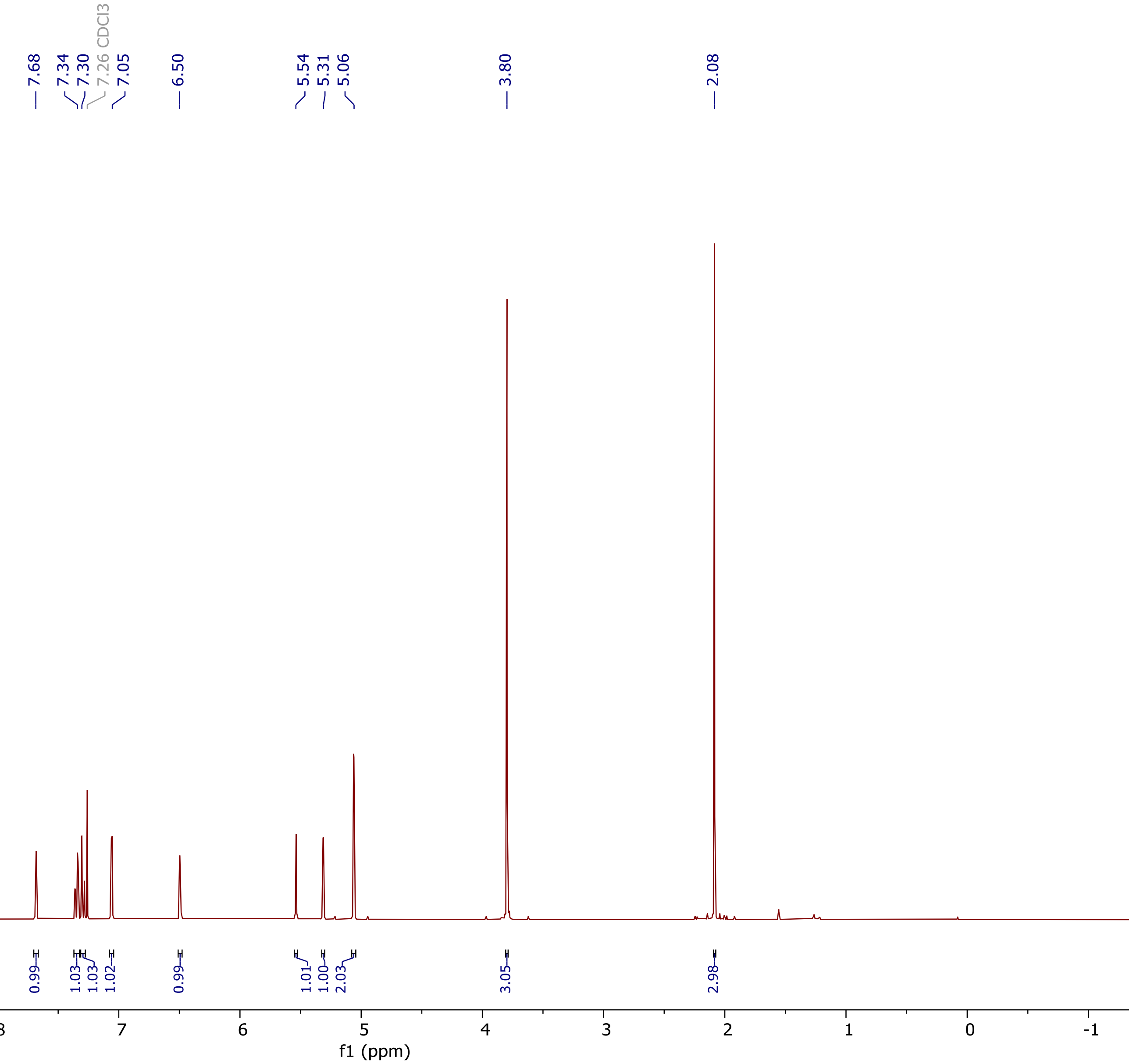

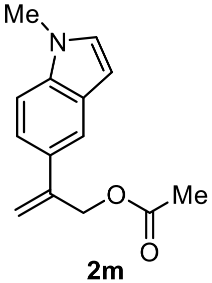

2m

$^{13}C\{^{1}H\}$ NMR (101 MHz, 298 K, $CDCl_3$)

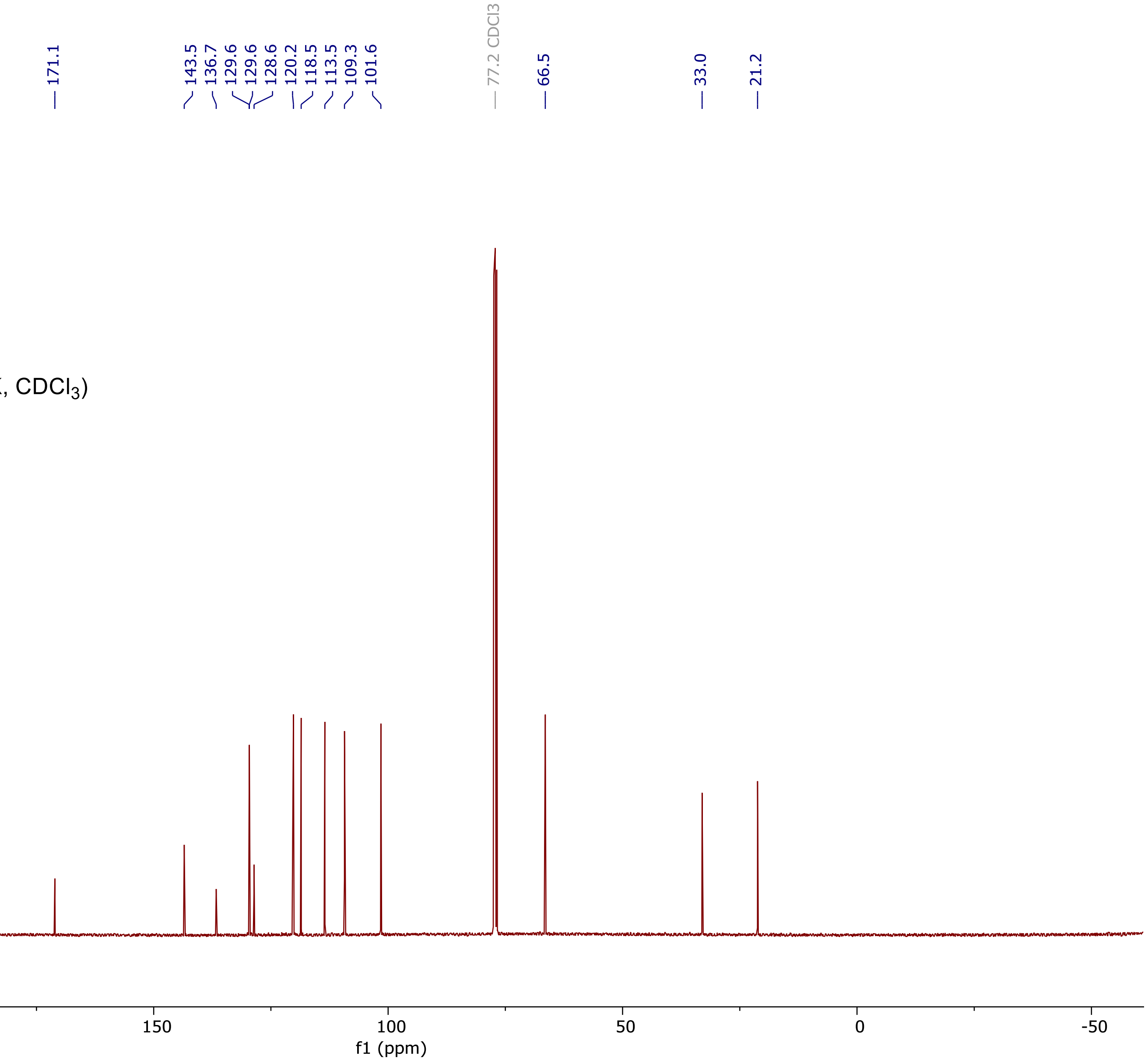

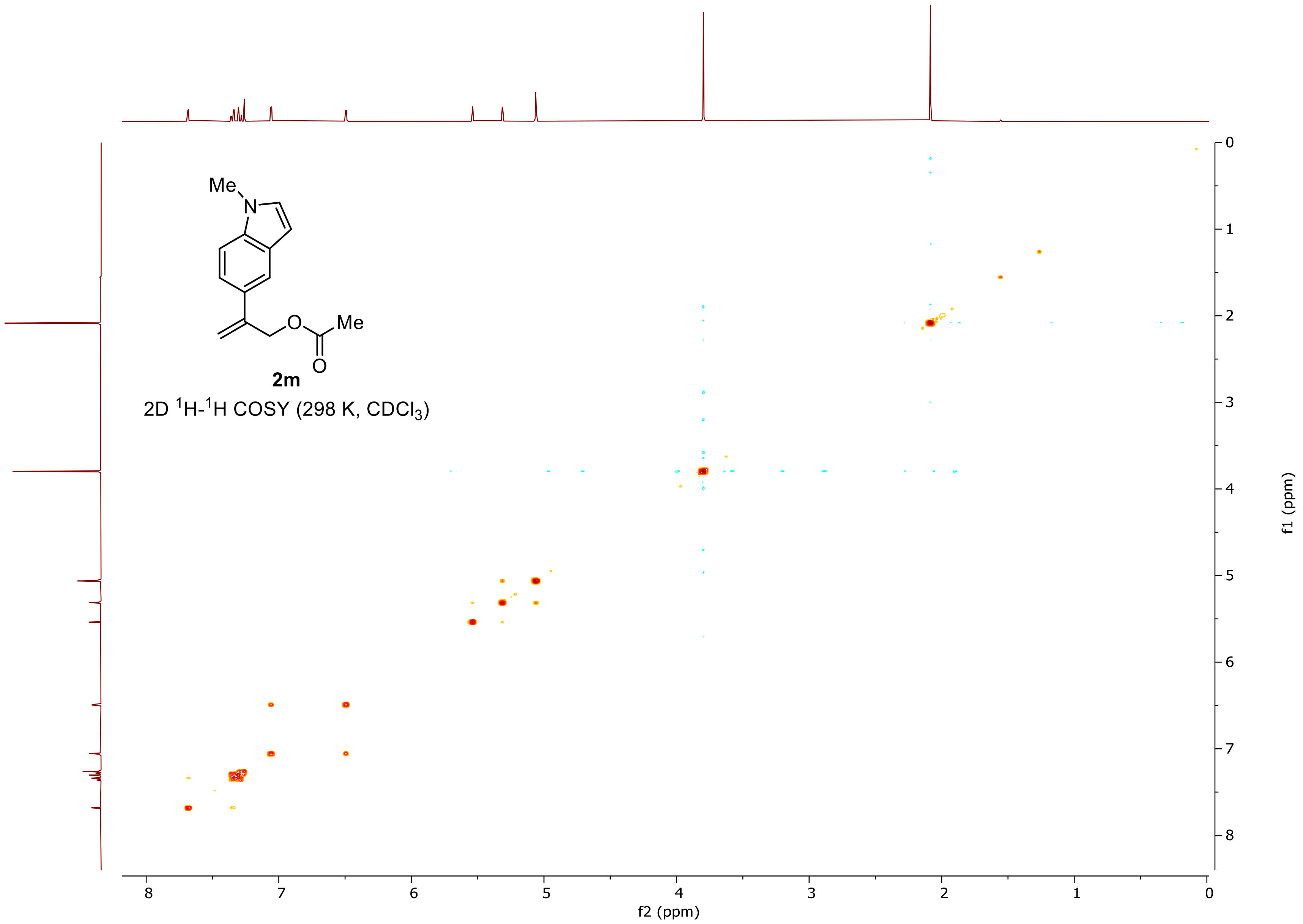

2m

2D ^{1}H-^{1}H COSY (298 K, $CDCl_3$)

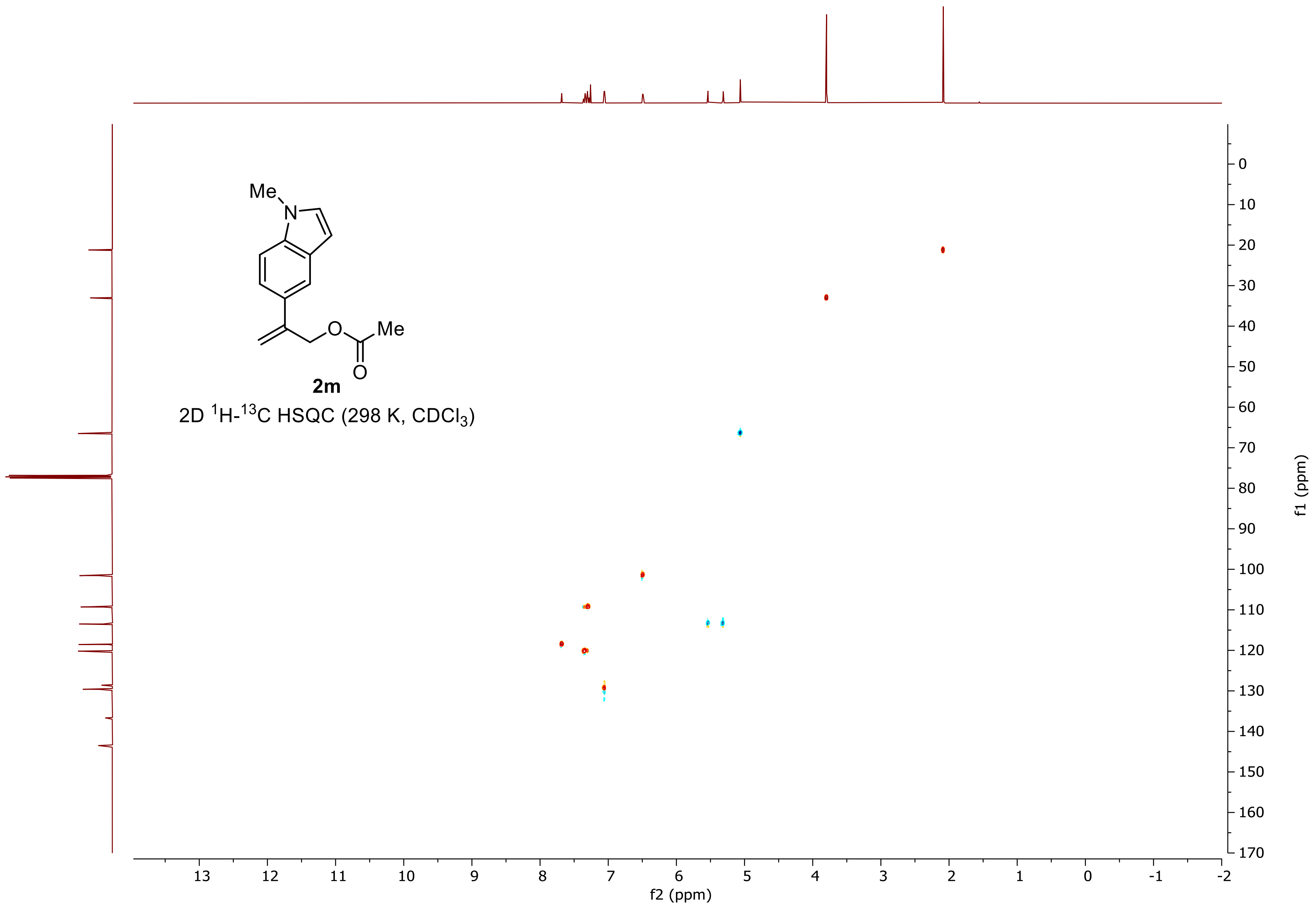
Me
N
O
Me
O
2m
2D 1H-13C HSQC (298 K, CDCl3)
0
10
20
30
40
50
60
70
80
90
100
110
120
130
140
150
160
170
f1 (ppm)
13
12
11
10
9
8
7
6
5
4
3
2
1
0
-1
-2
f2 (ppm)

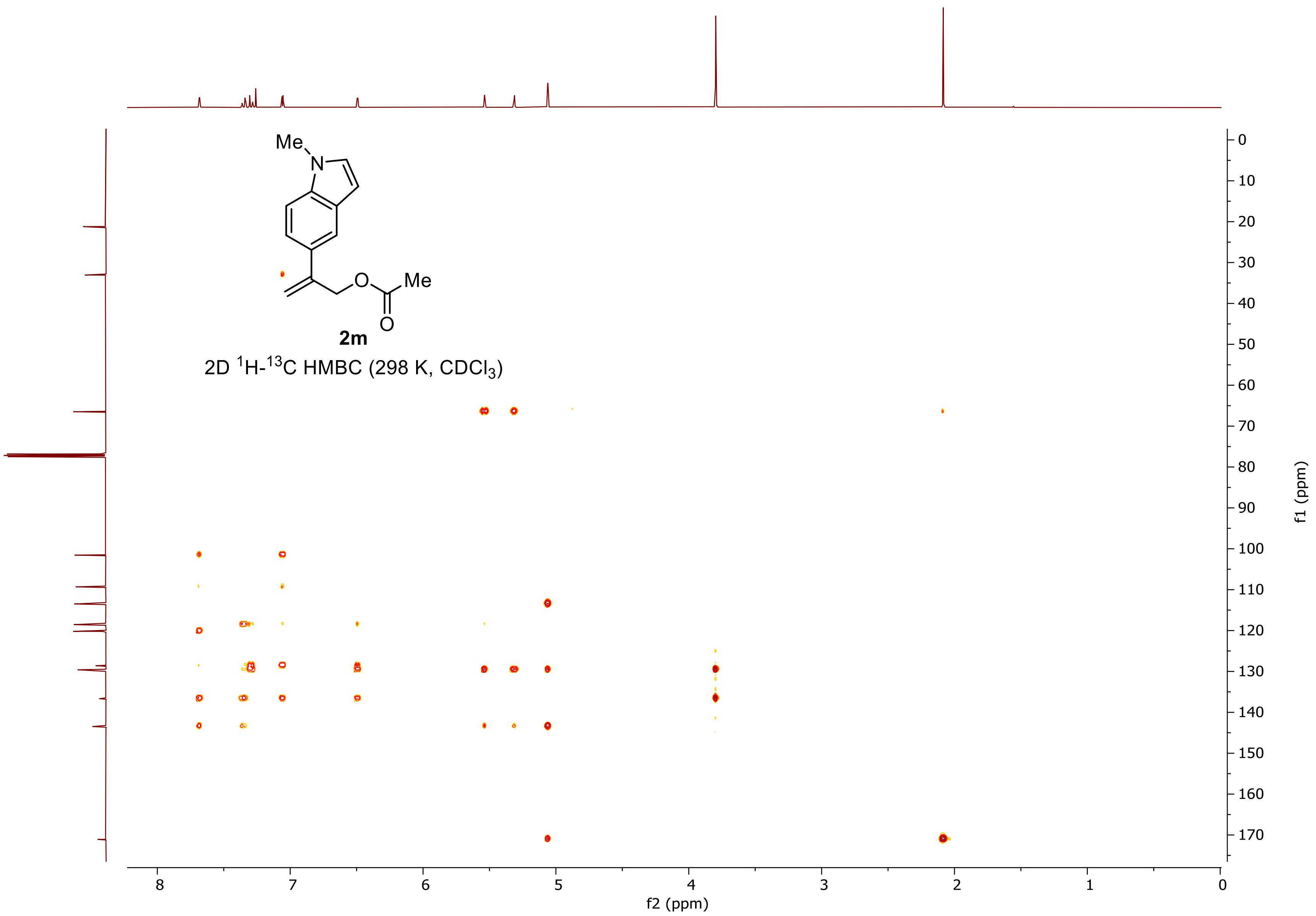
Me
N
O
Me
O
2m
2D 1H-13C HMBC (298 K, CDCl3)
f2 (ppm)
f1 (ppm)

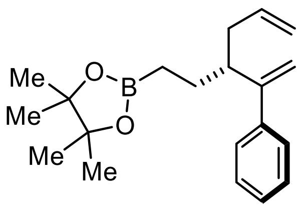

3a

^{1}H NMR (400 MHz, 298 K, $CDCl_3$)

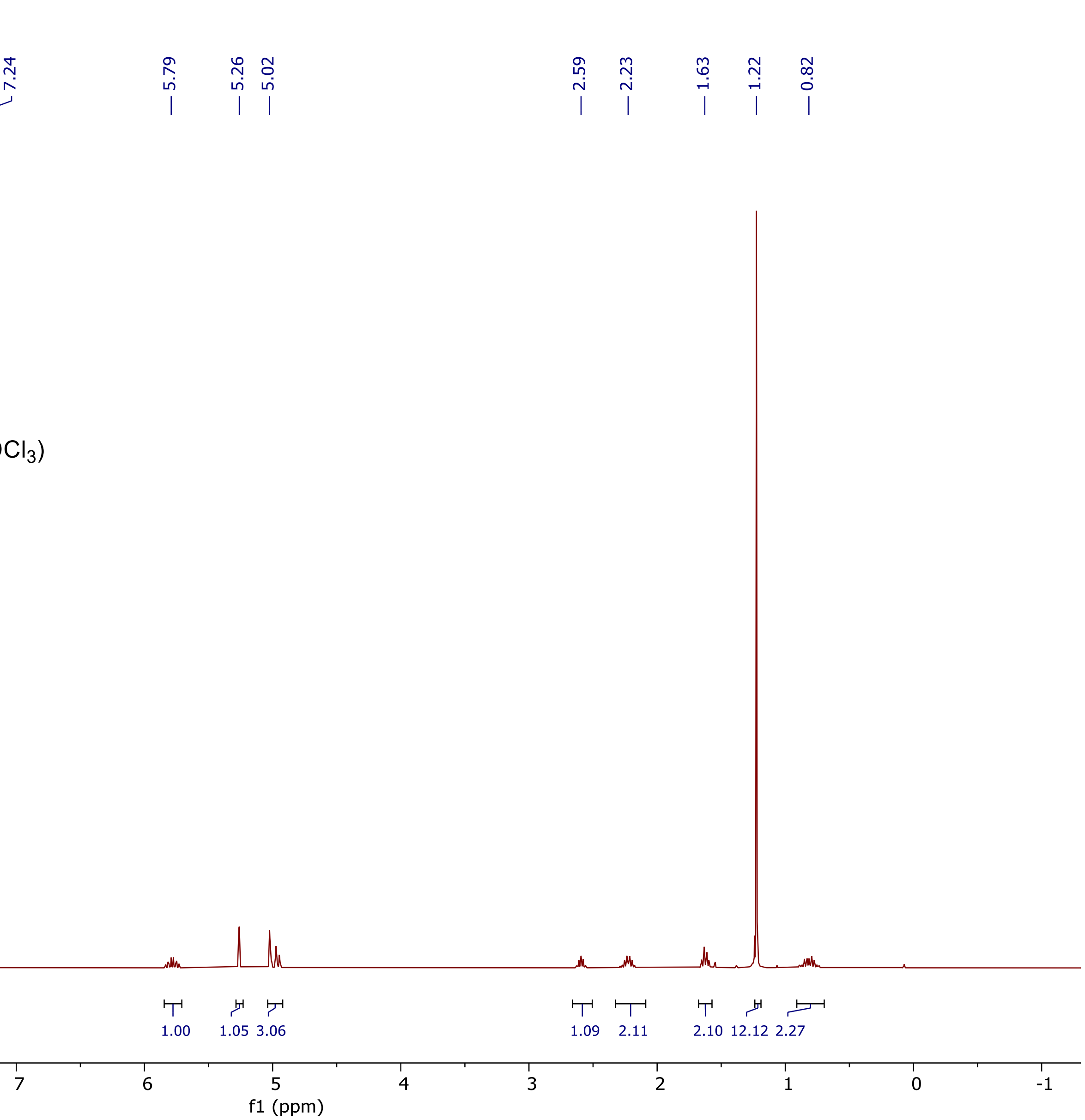

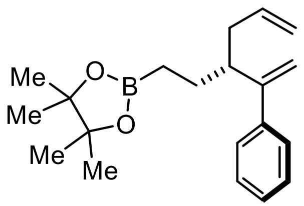

3a

$^{13}C\{^{1}H\}$ NMR (101 MHz, 298 K, $CDCl_3$)

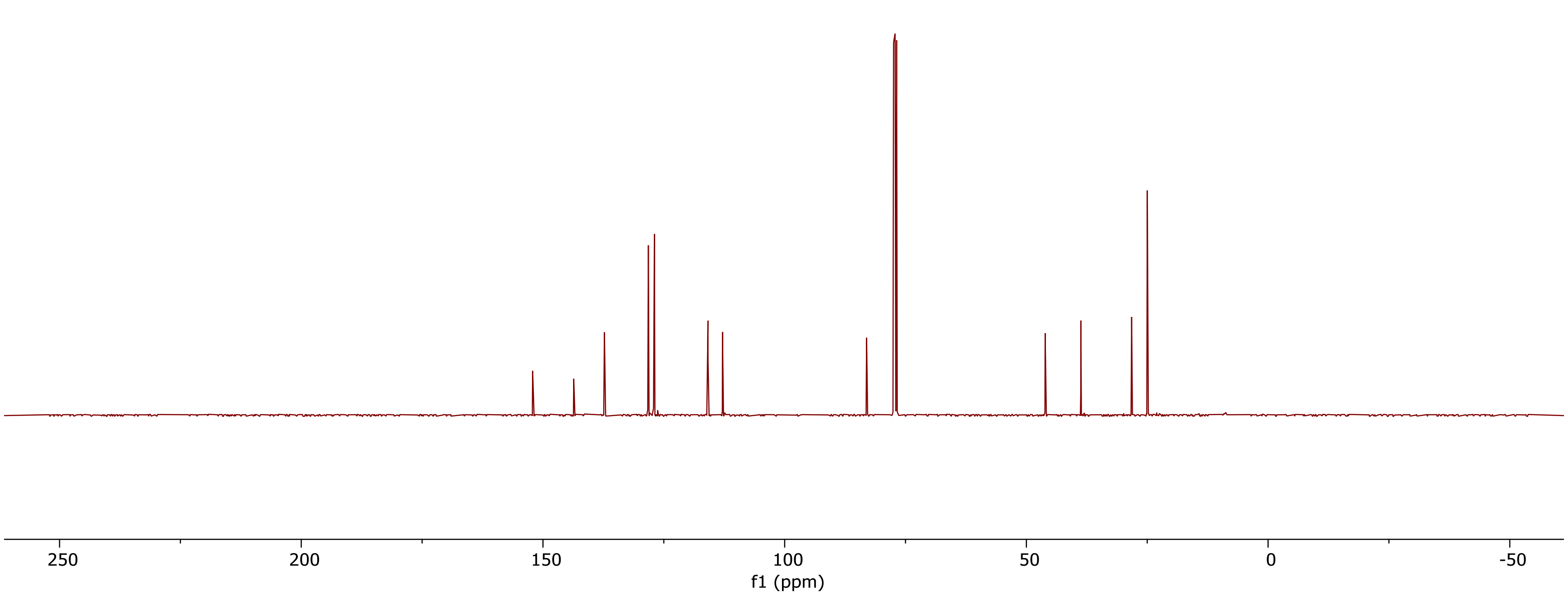

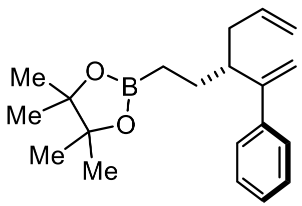

3a

$^{11}B\{^{1}H\}$ NMR (128 MHz, 298 K, $CDCl_3$)

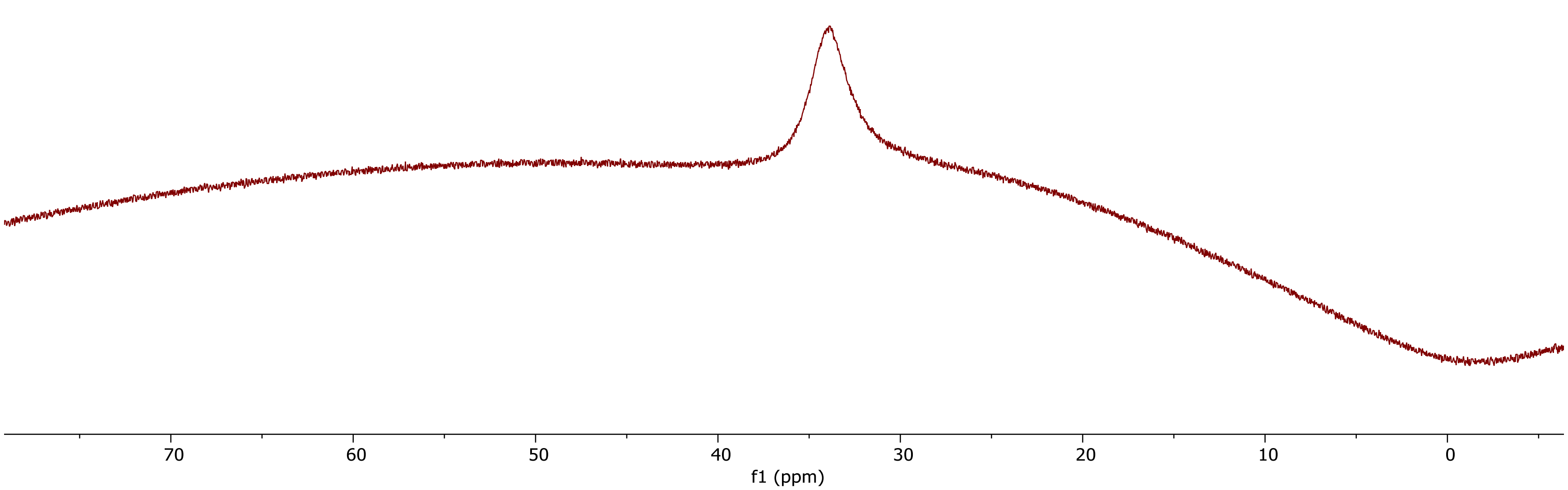

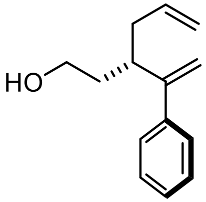

7a

^{1}H NMR (400 MHz, 298 K, $CDCl_3$)

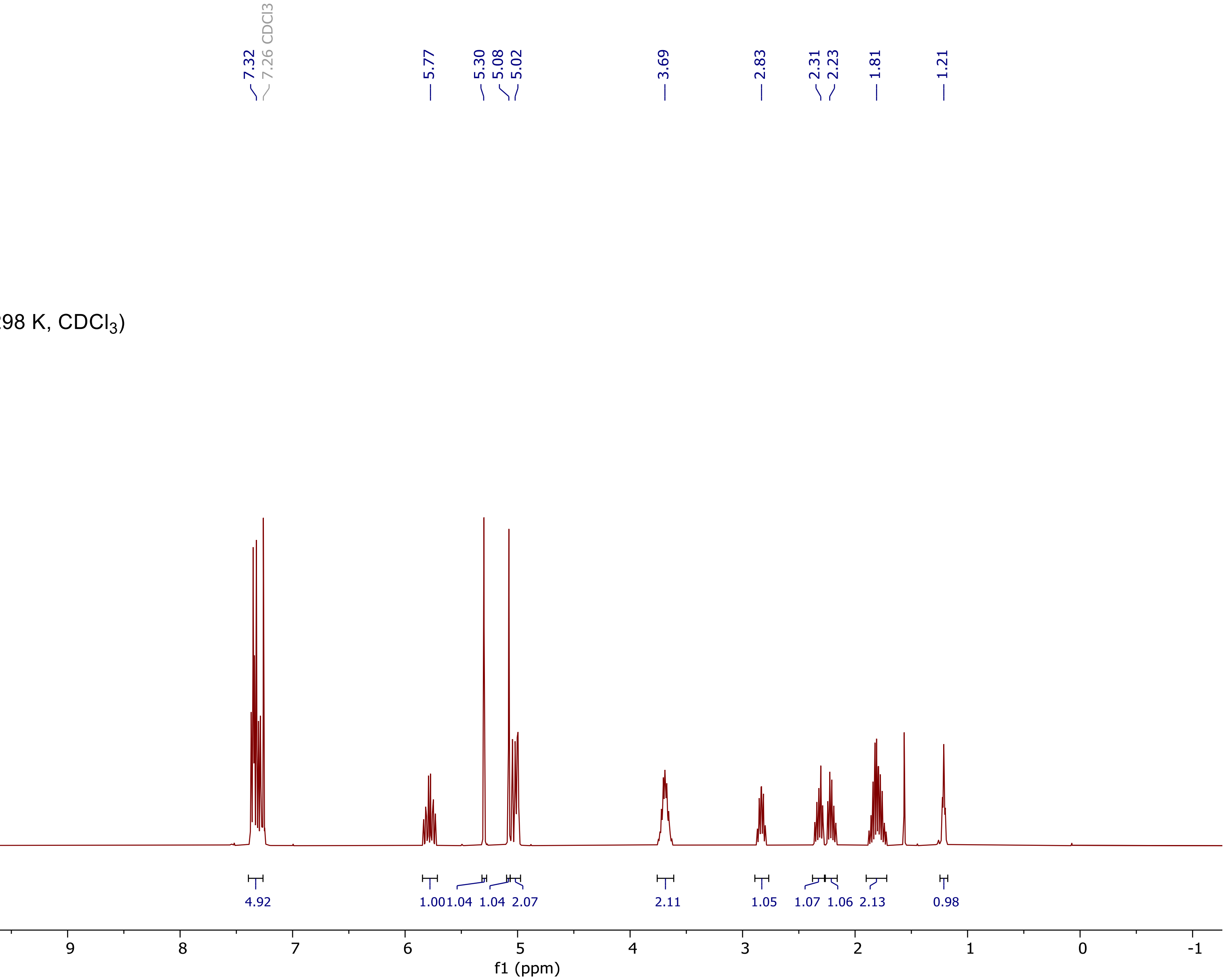

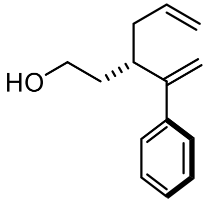

7a

$^{13}C\{^1H\}$ NMR (101 MHz, 298 K, $CDCl_3$)

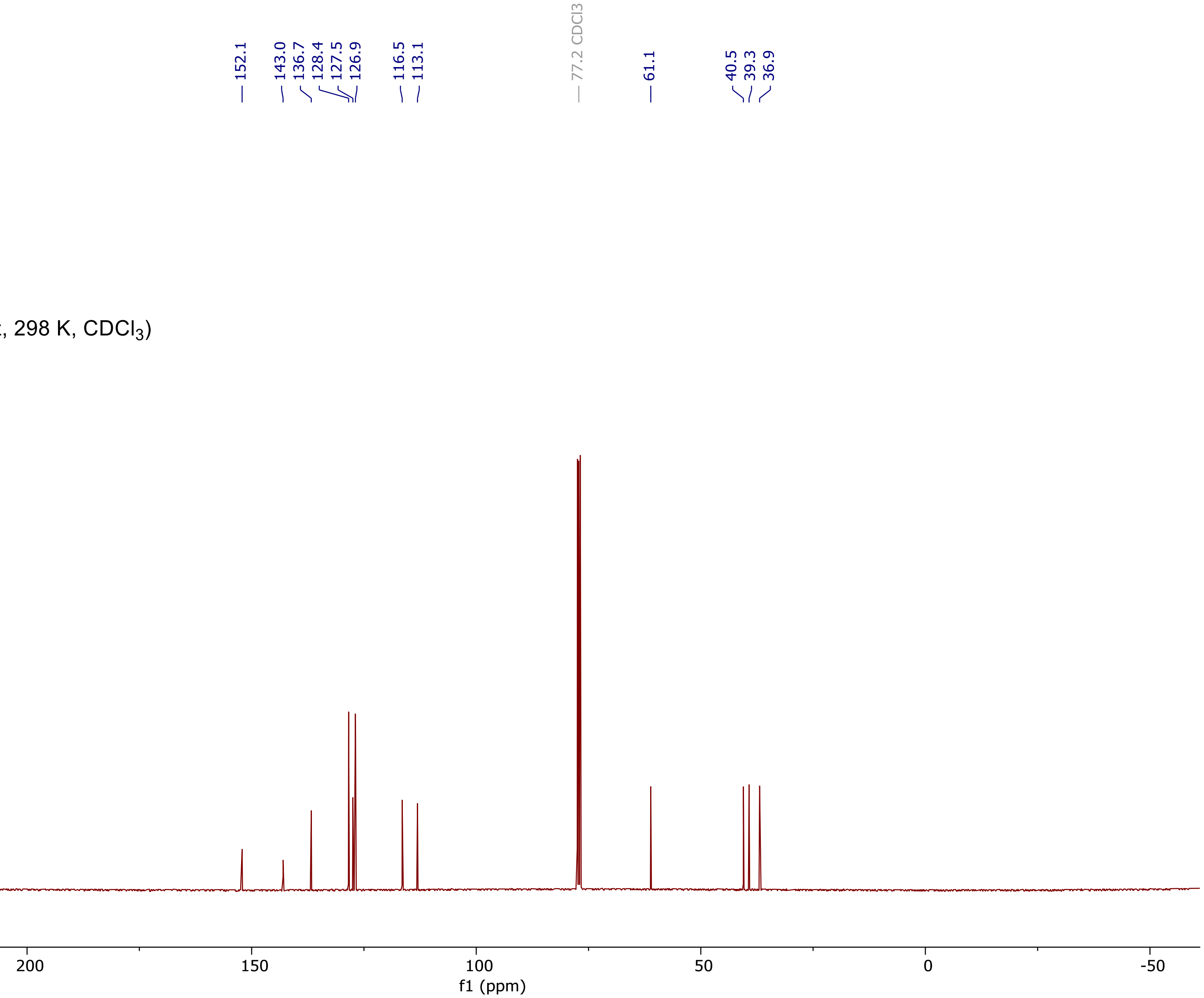

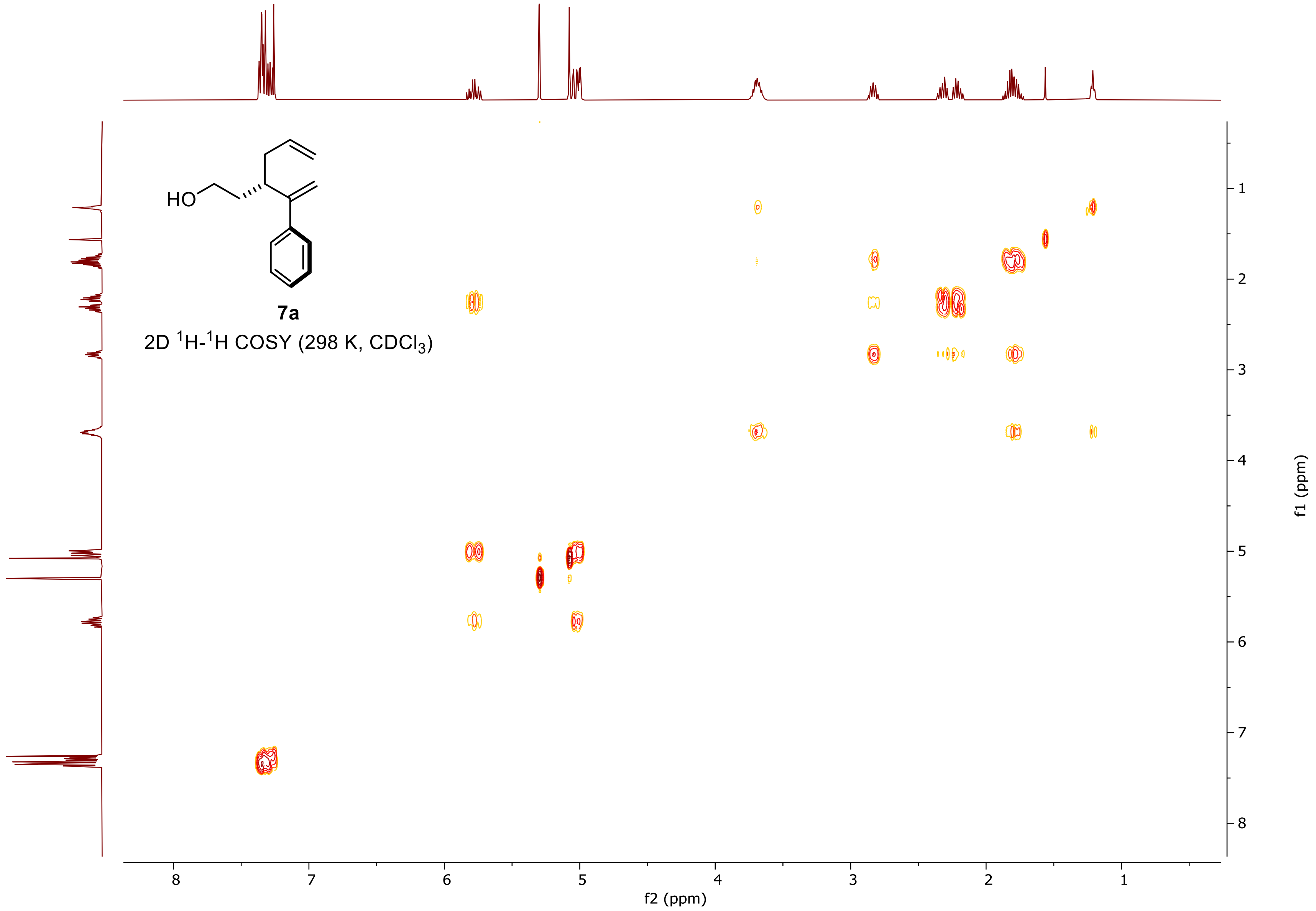
HO
7a
2D ^{1}H-^{1}H COSY (298 K, $CDCl_3$)
f1 (ppm)
f2 (ppm)
1
2
3
4
5
6
7
8

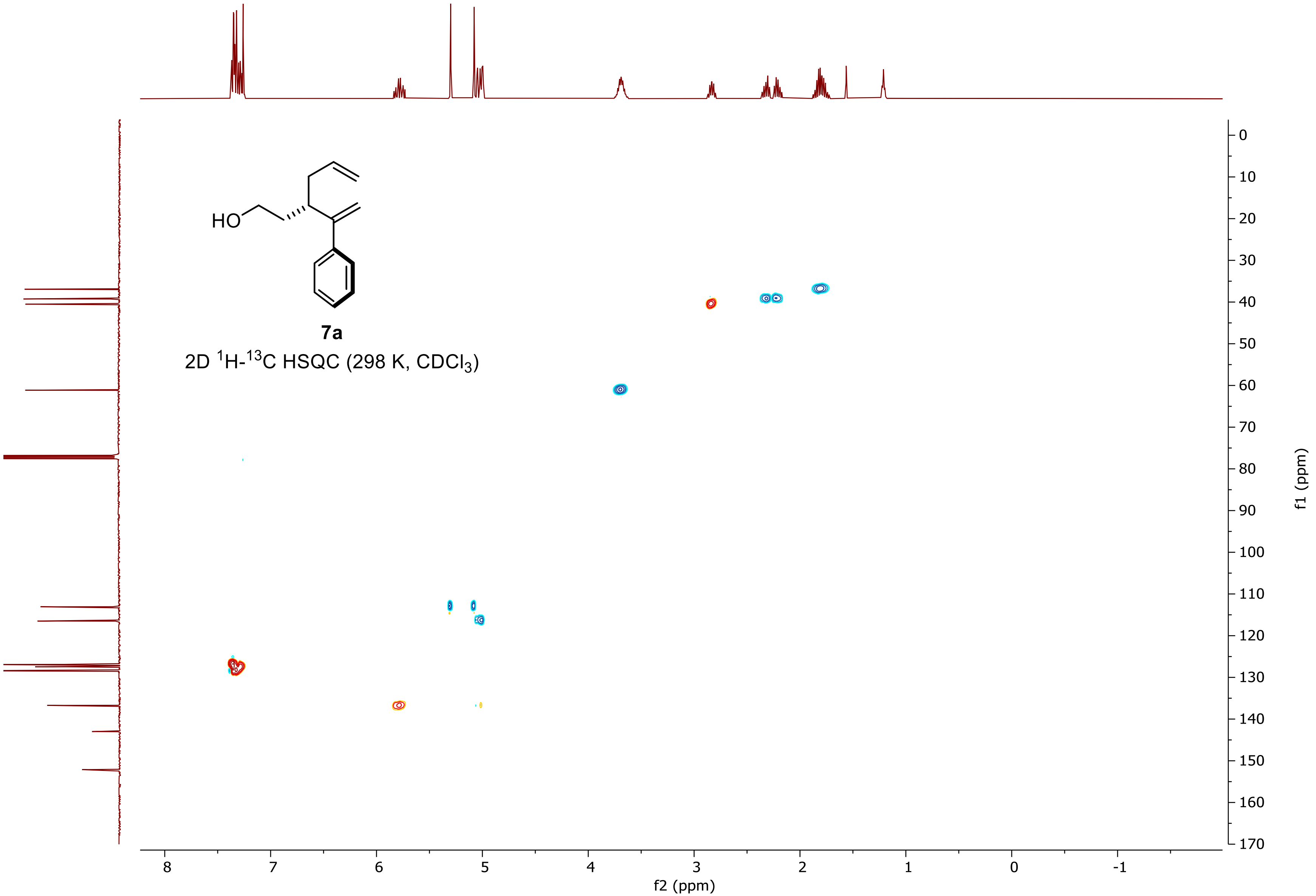
HO
7a
2D ^{1}H-^{13}C HSQC (298 K, $CDCl_3$)
f2 (ppm)
f1 (ppm)

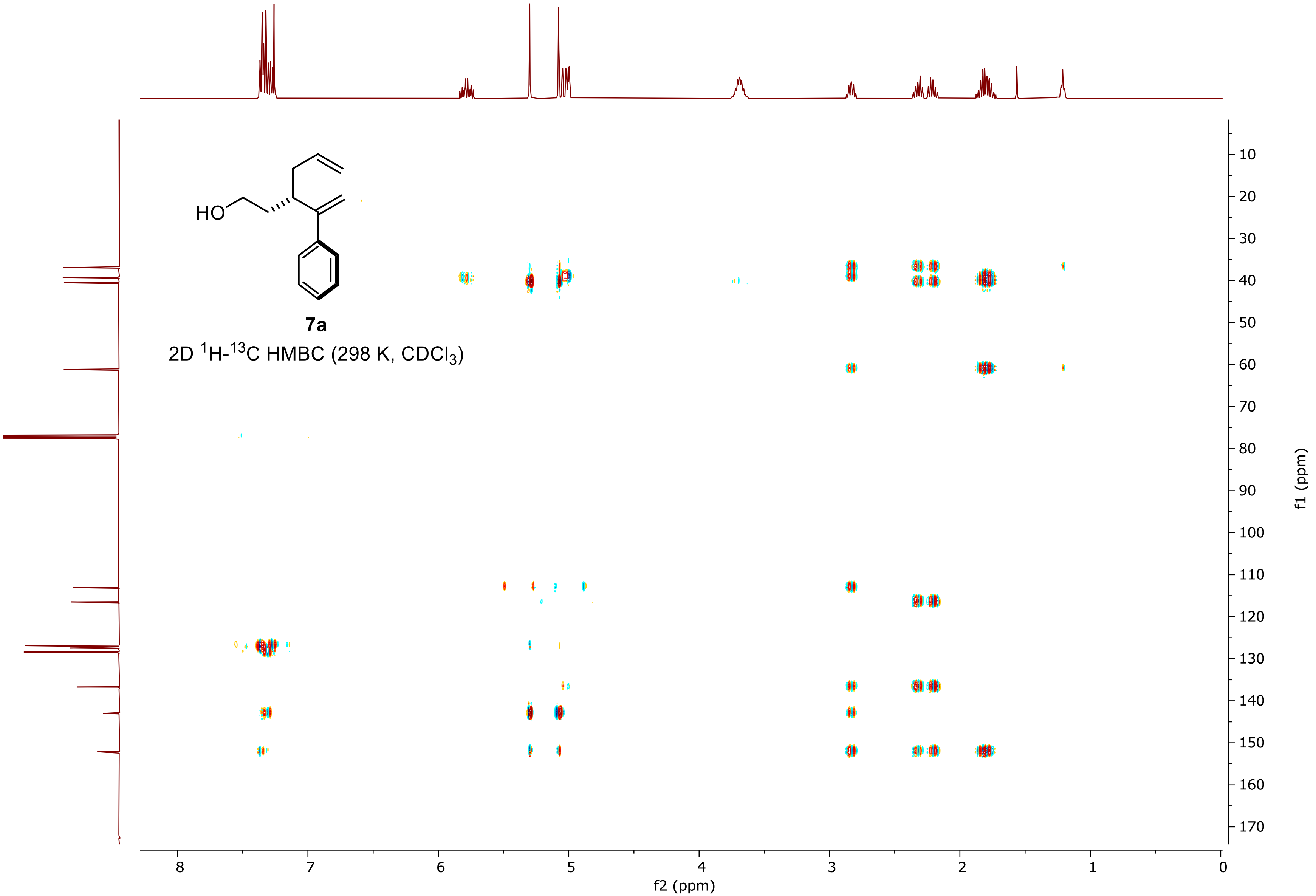

7a

2D ^{1}H-^{13}C HMBC (298 K, $CDCl_3$)

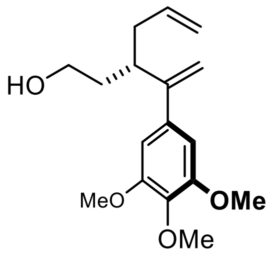

7b

^{1}H NMR (400 MHz, 298 K, $CDCl_3$)

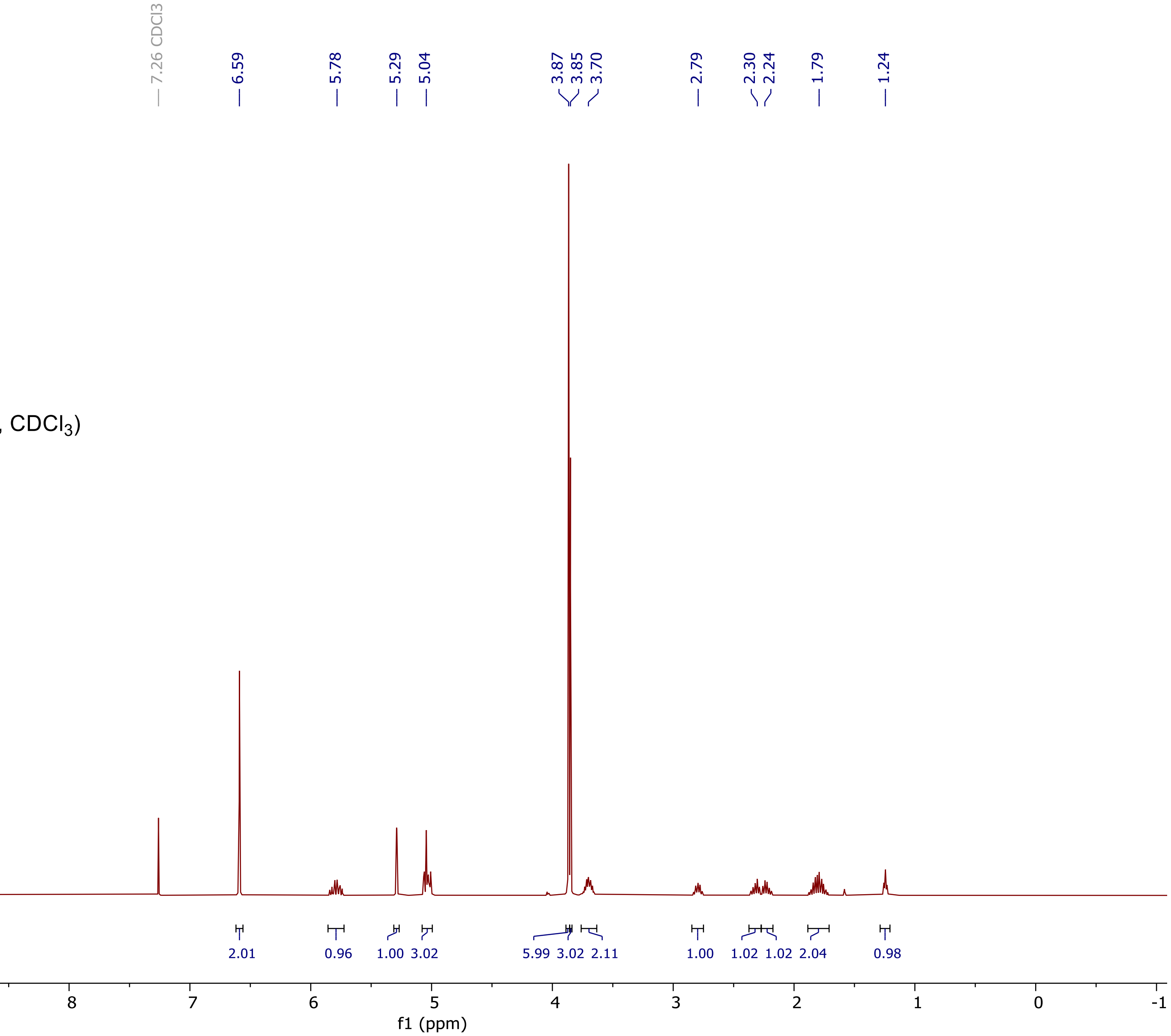

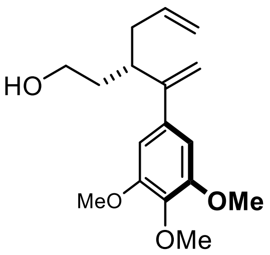

7b

$^{13}C\{^1H\}$ NMR (101 MHz, 298 K, $CDCl_3$)

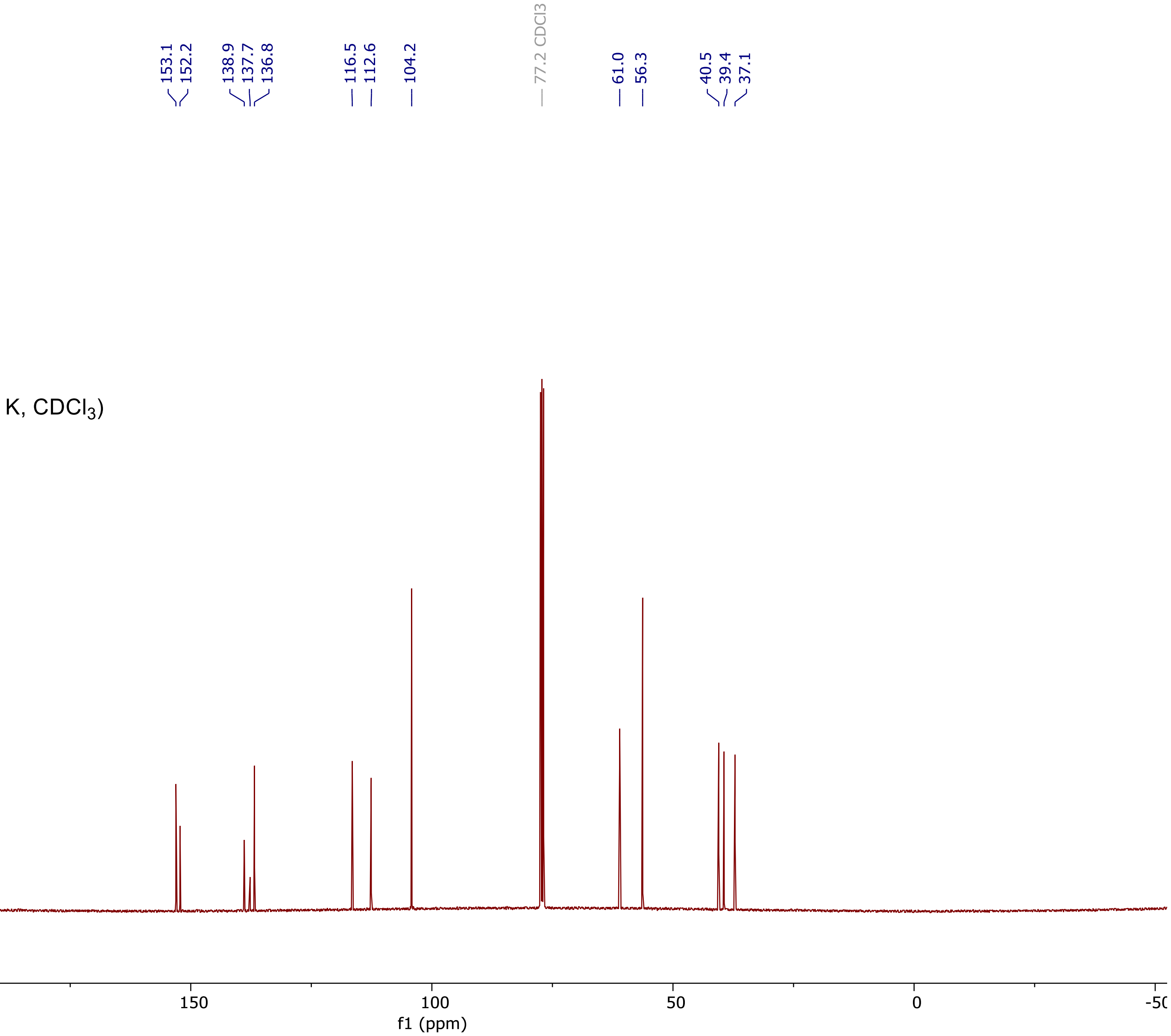

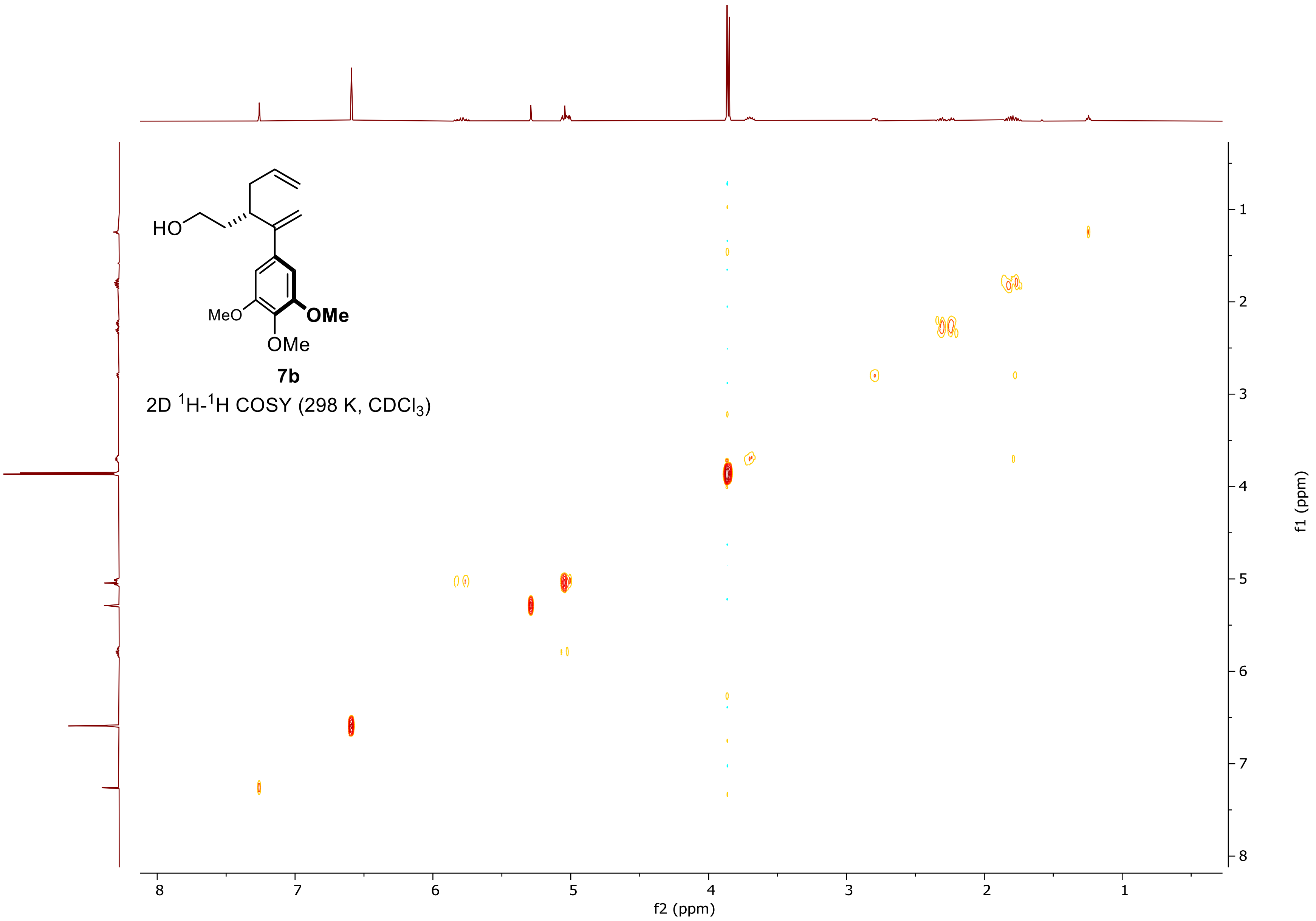
HO
MeO
OMe
OMe
7b
2D ¹H-¹H COSY (298 K, CDCl3)
f2 (ppm)
f1 (ppm)

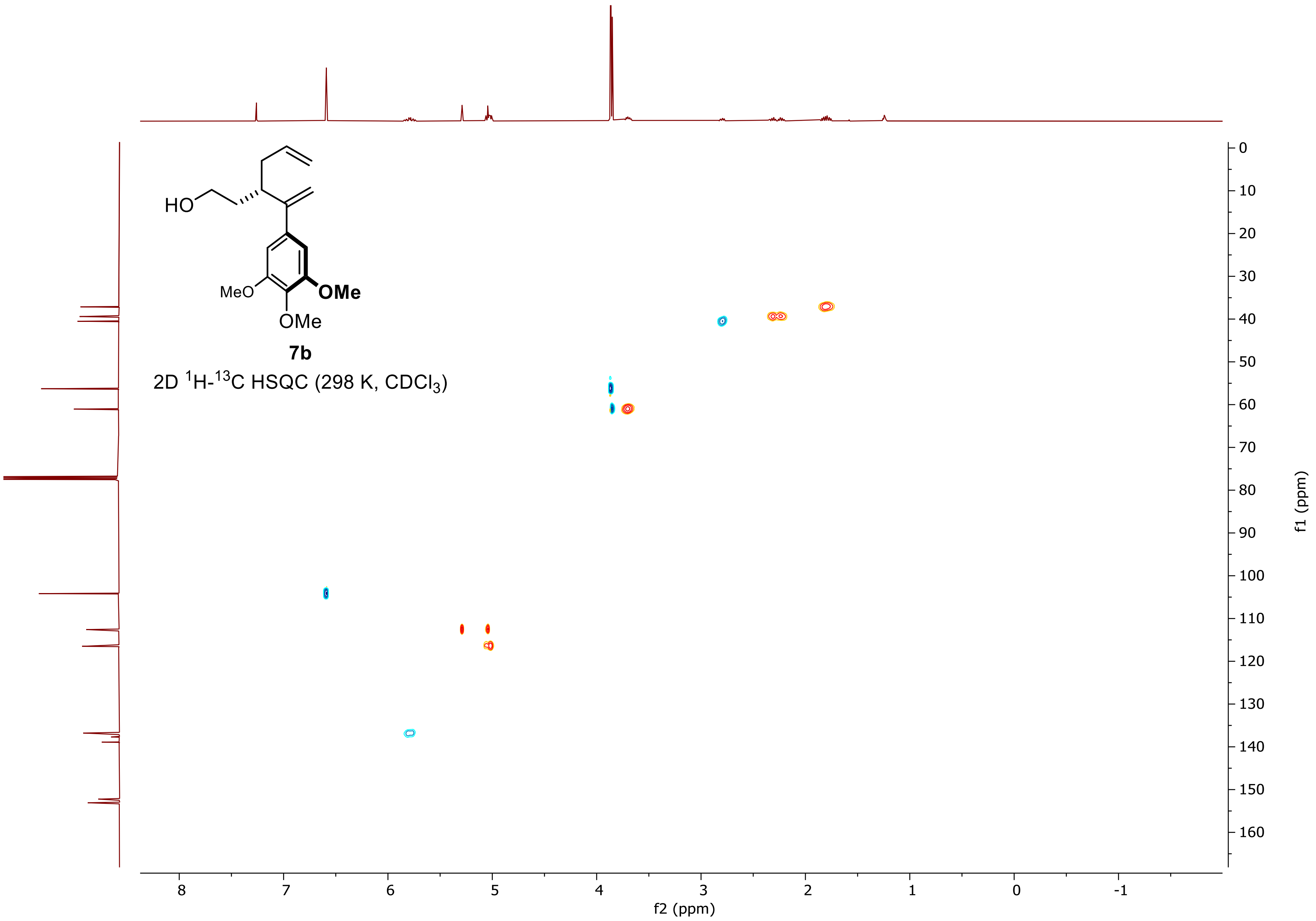
HO
MeO
OMe
OMe
7b
2D 1H-13C HSQC (298 K, CDCl3)
f1 (ppm)
f2 (ppm)

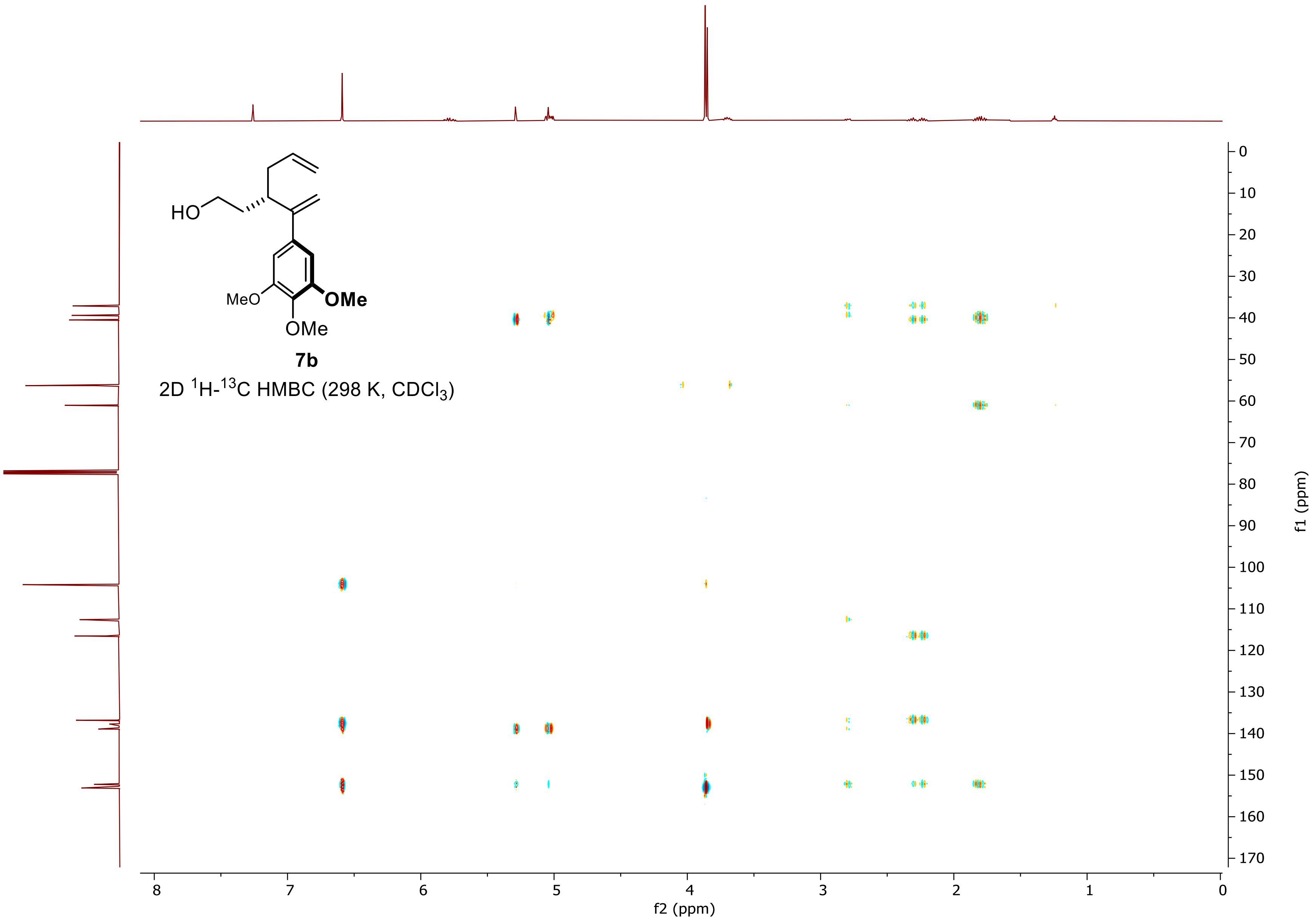
HO
MeO
OMe
OMe
7b
2D ¹H-¹³C HMBC (298 K, CDCl₃)
f1 (ppm)
f2 (ppm)

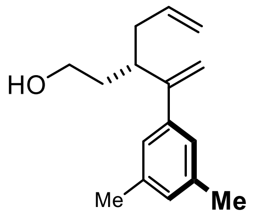

7c

^{1}H NMR (400 MHz, 298 K, $CDCl_3$)

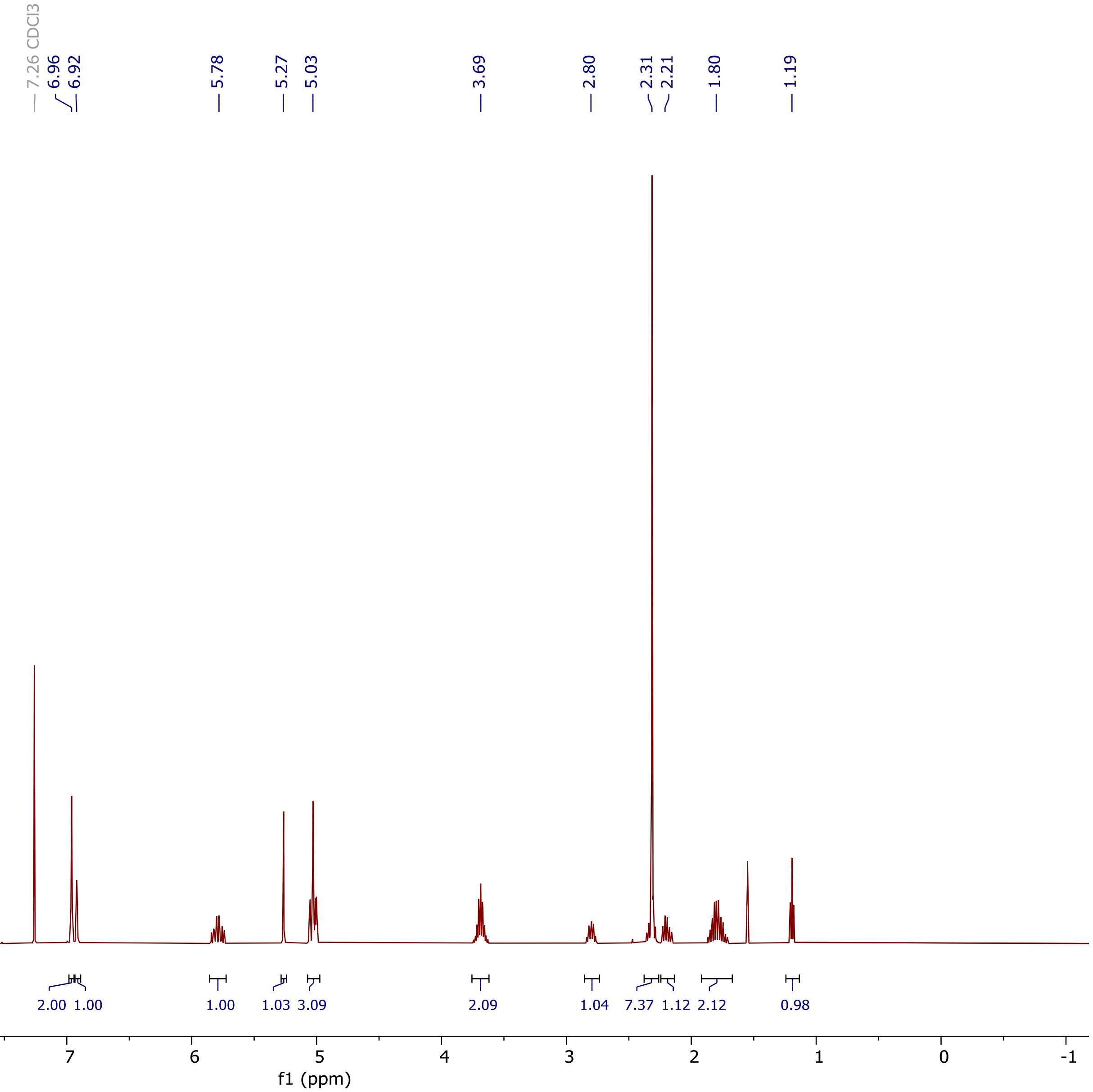

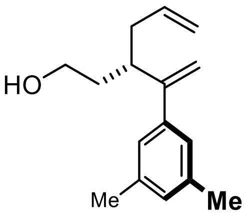

7c

$^{13}C\{^{1}H\}$ NMR (101 MHz, 298 K, $CDCl_3$)

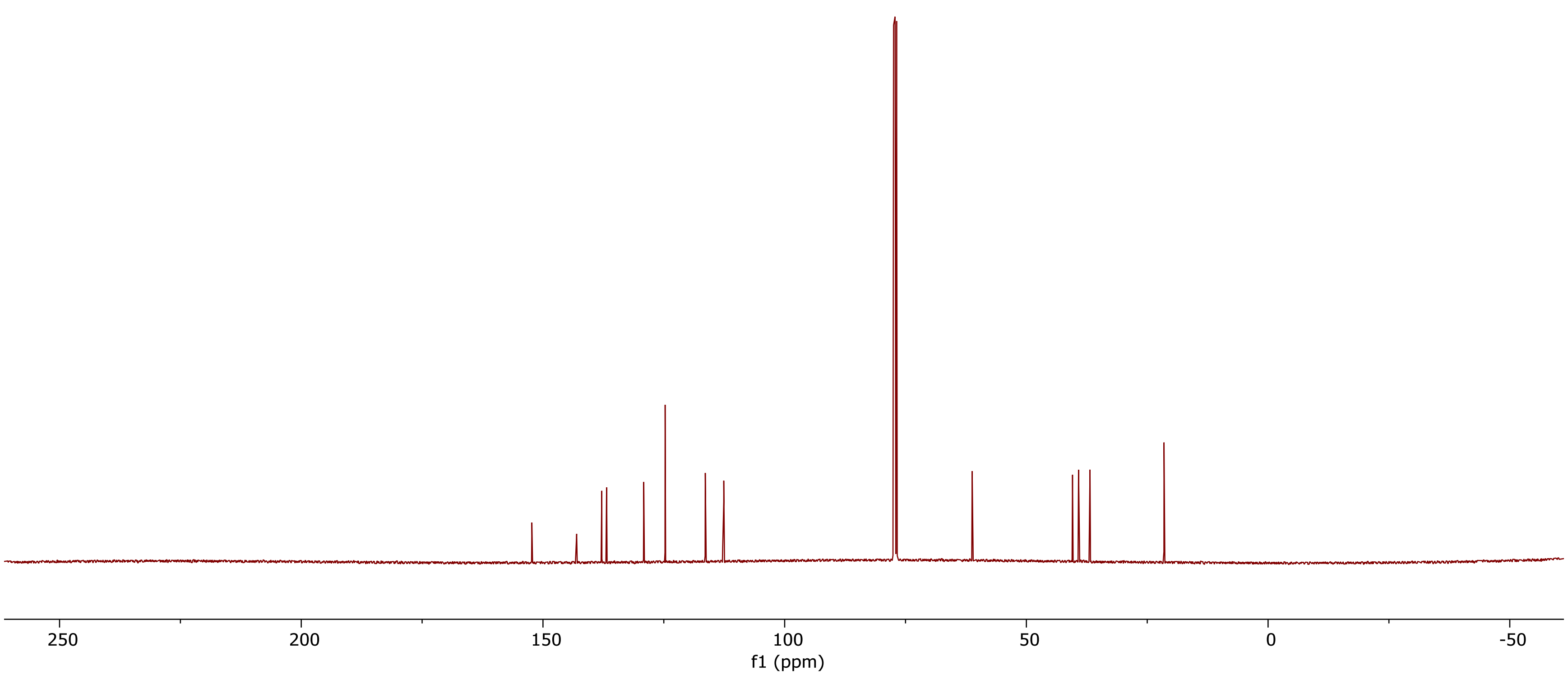

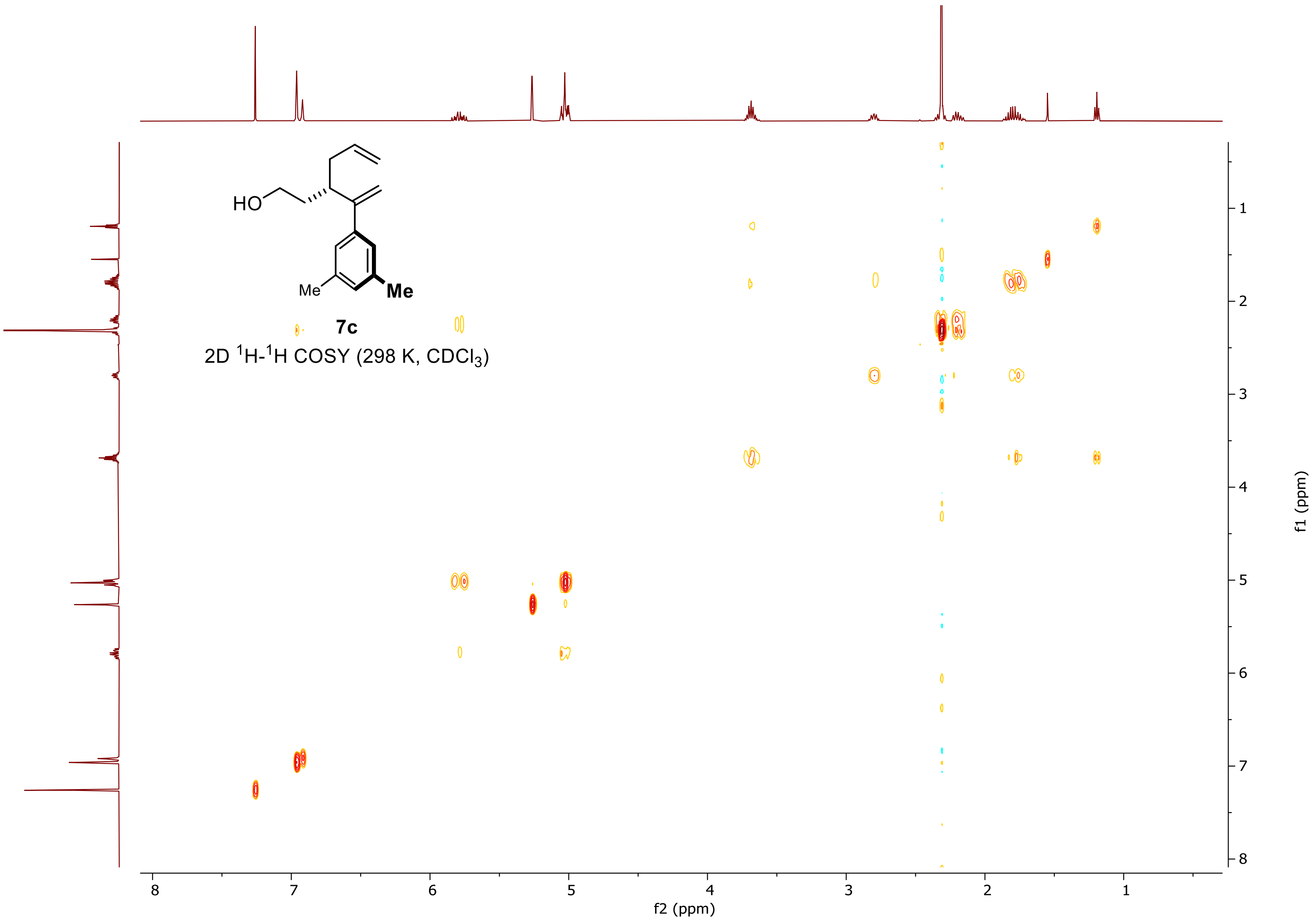

7c

2D ^{1}H-^{1}H COSY (298 K, $CDCl_3$)

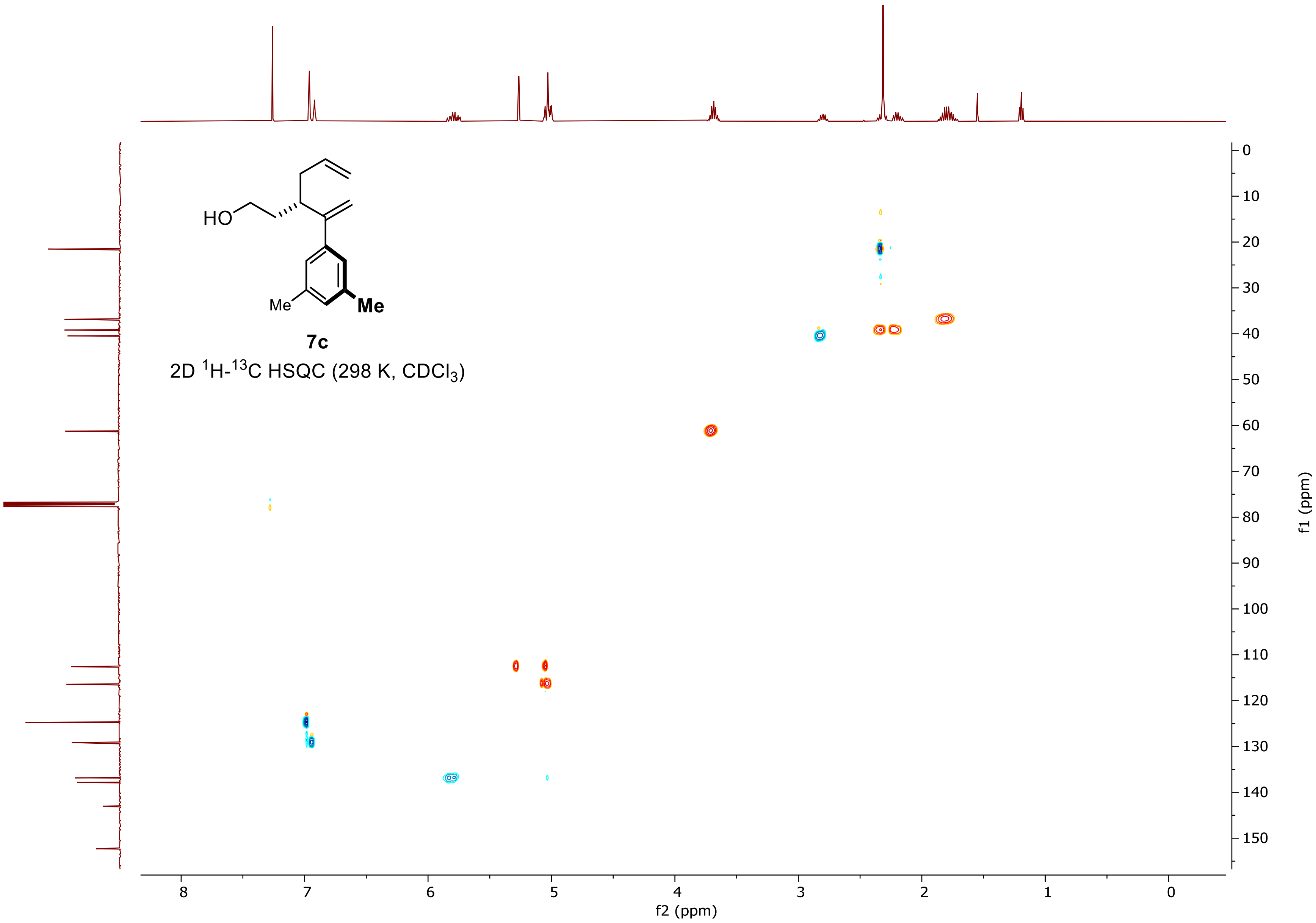
HO
Me
Me
7c
2D ¹H-¹³C HSQC (298 K, CDCl₃)
f2 (ppm)
f1 (ppm)
8
7
6
5
4
3
2
1
0
10
20
30
40
50
60
70
80
90
100
110
120
130
140
150

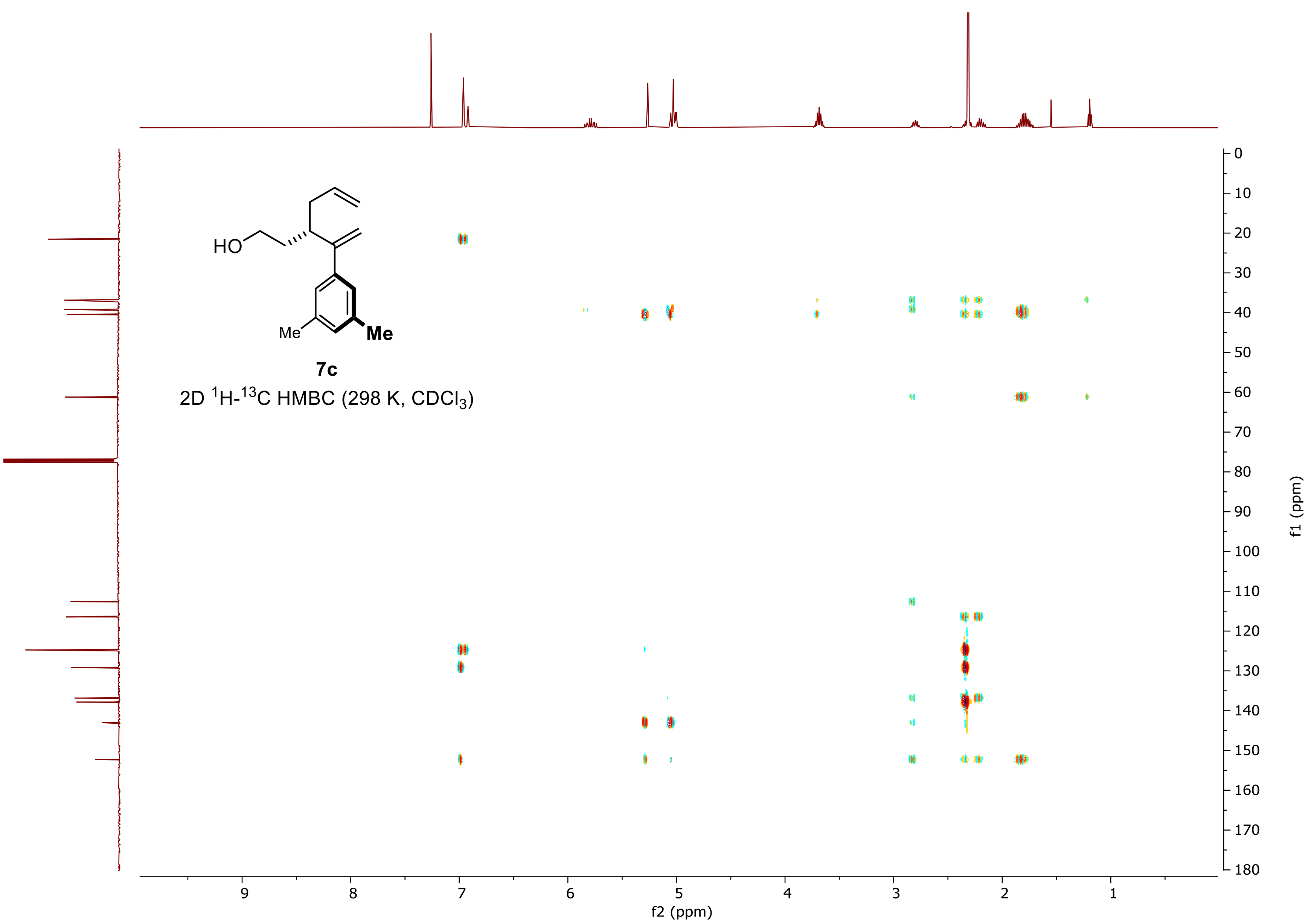
HO
Me
Me
7c
2D 1H-13C HMBC (298 K, CDCl3)
f2 (ppm)
f1 (ppm)

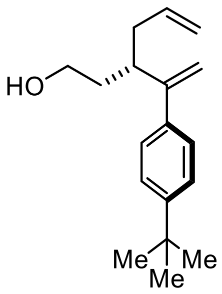

7d

^{1}H NMR (400 MHz, 298 K, $CDCl_3$)

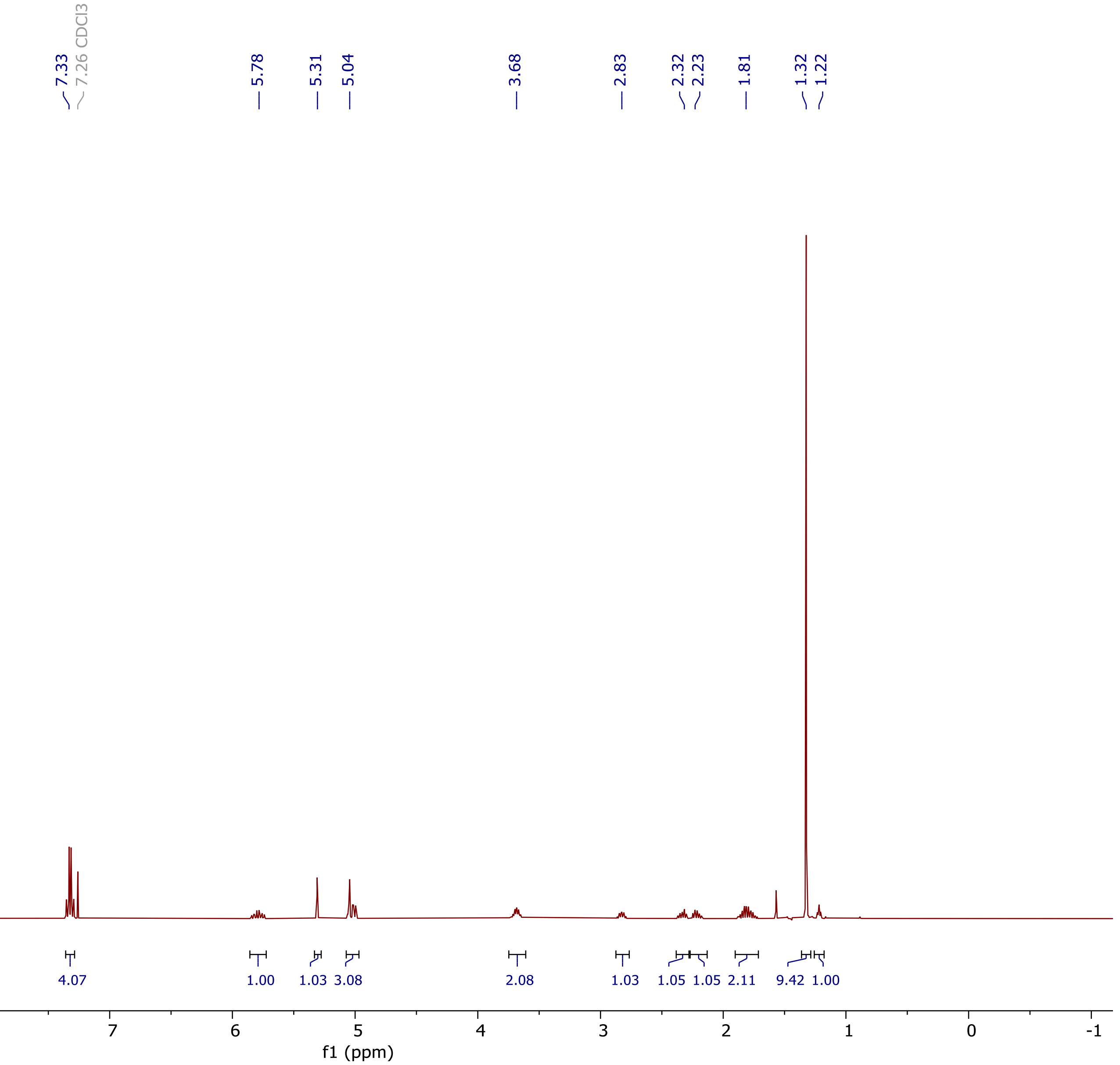

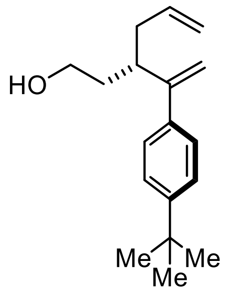

7d

$^{13}C\{^{1}H\}$ NMR (101 MHz, 298 K, $CDCl_3$)

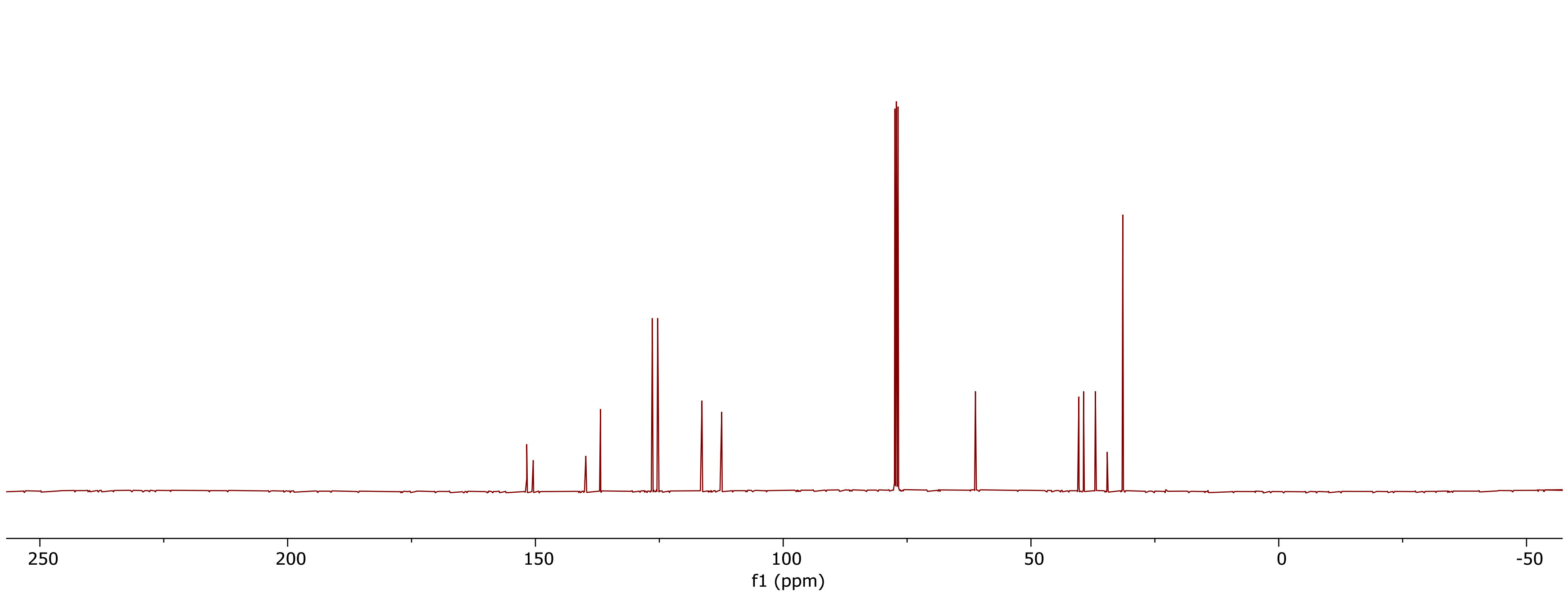

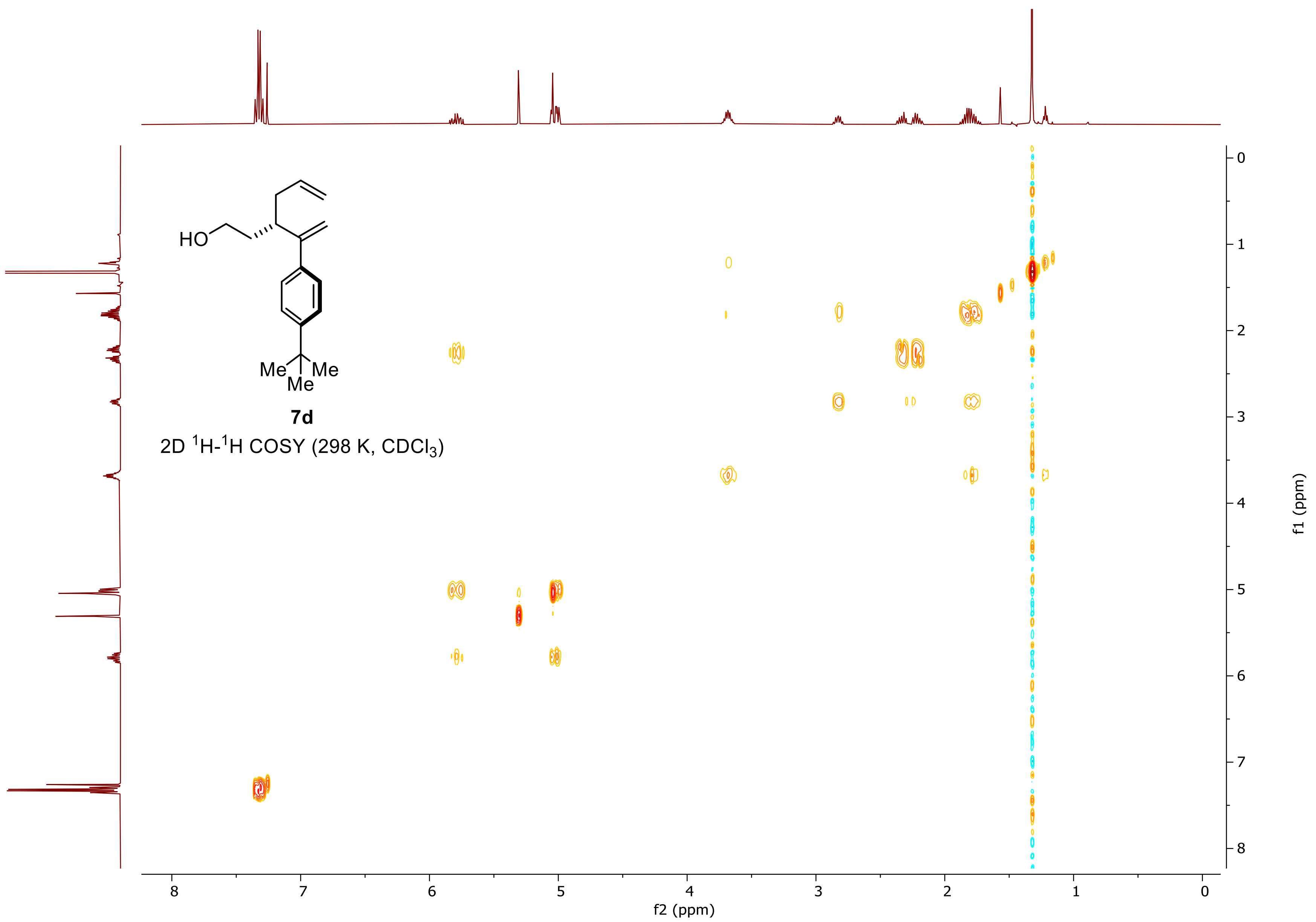
HO
Me
Me
Me
7d
2D 1H-1H COSY (298 K, CDCl3)
f2 (ppm)
f1 (ppm)
8
7
6
5
4
3
2
1
0

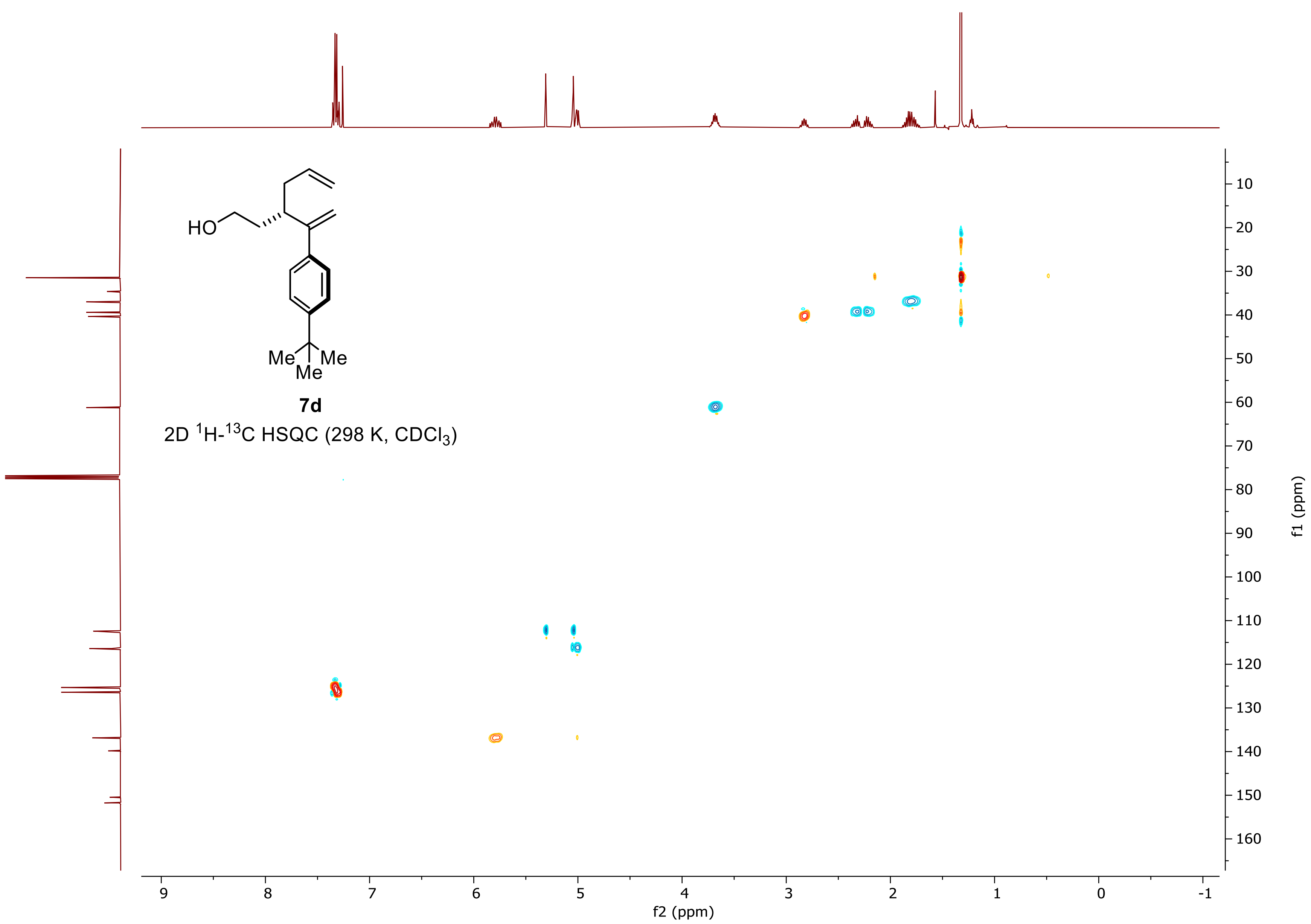

7d

2D ^{1}H-^{13}C HSQC (298 K, $CDCl_3$)

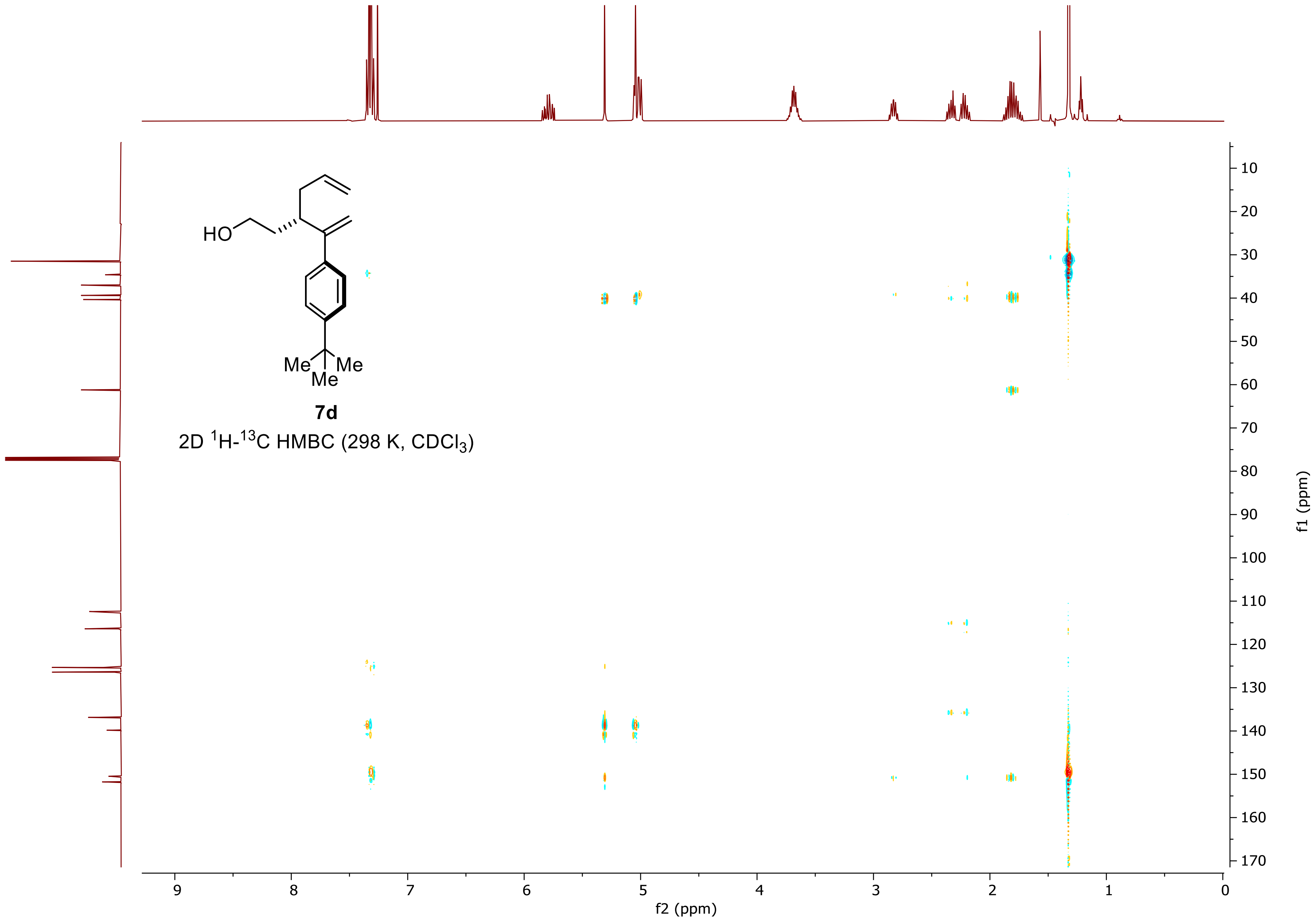
HO
Me
Me
Me
7d
2D 1H-13C HMBC (298 K, CDCl3)
f2 (ppm)
f1 (ppm)

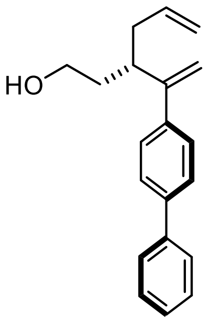

7e

^{1}H NMR (400 MHz, 298 K, $CDCl_3$)

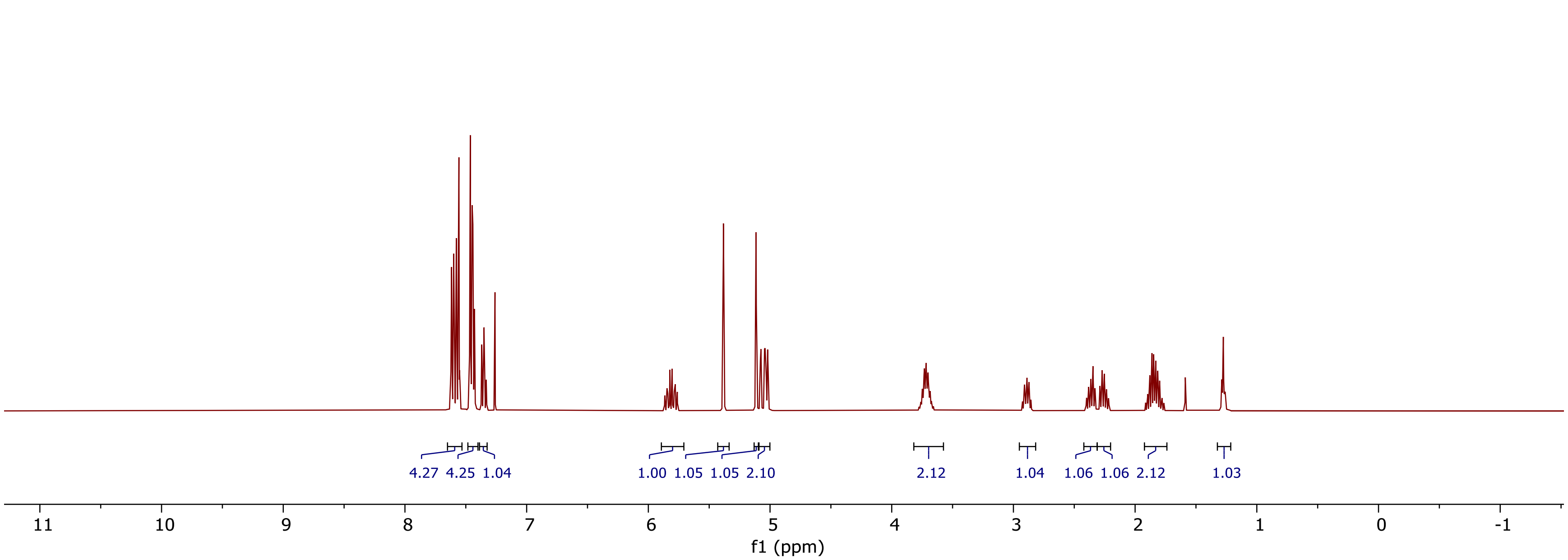

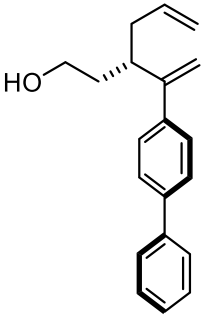

7e

$^{13}C\{^1H\}$ NMR (101 MHz, 298 K, $CDCl_3$)

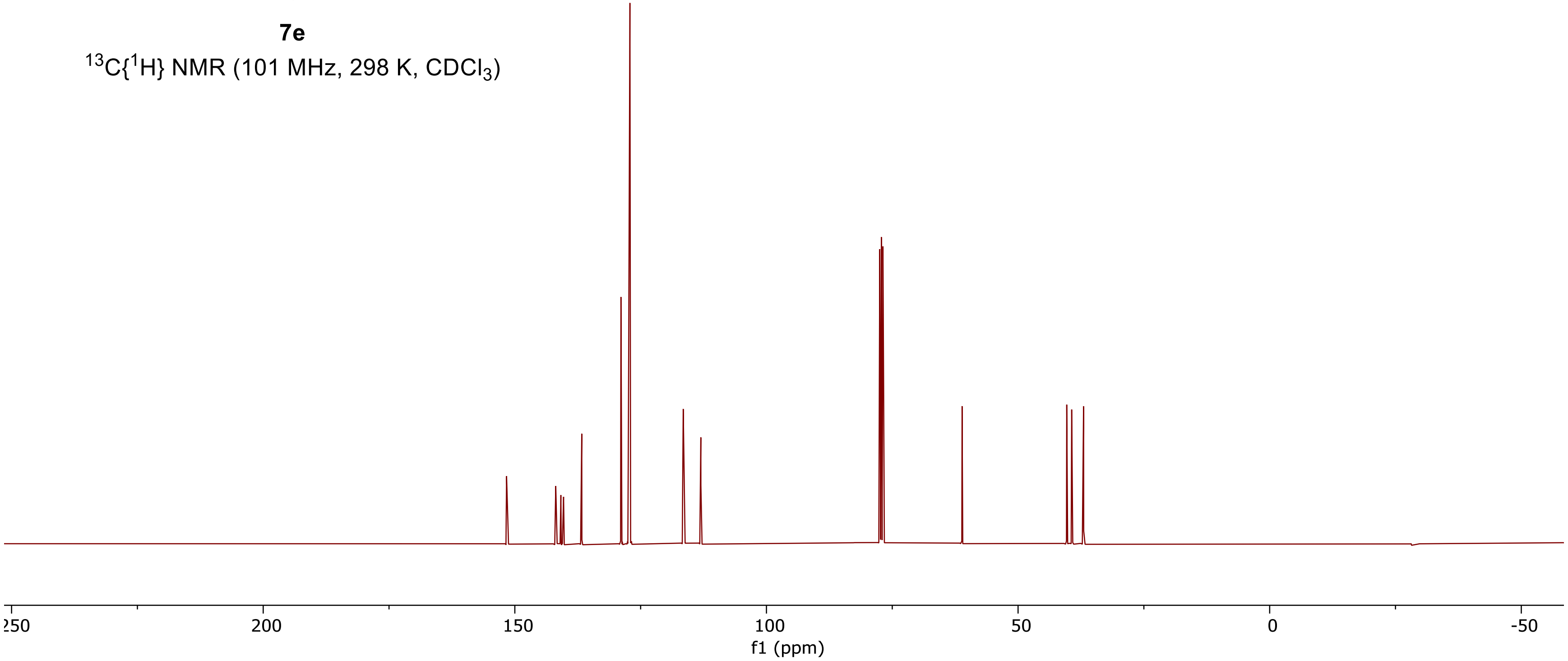

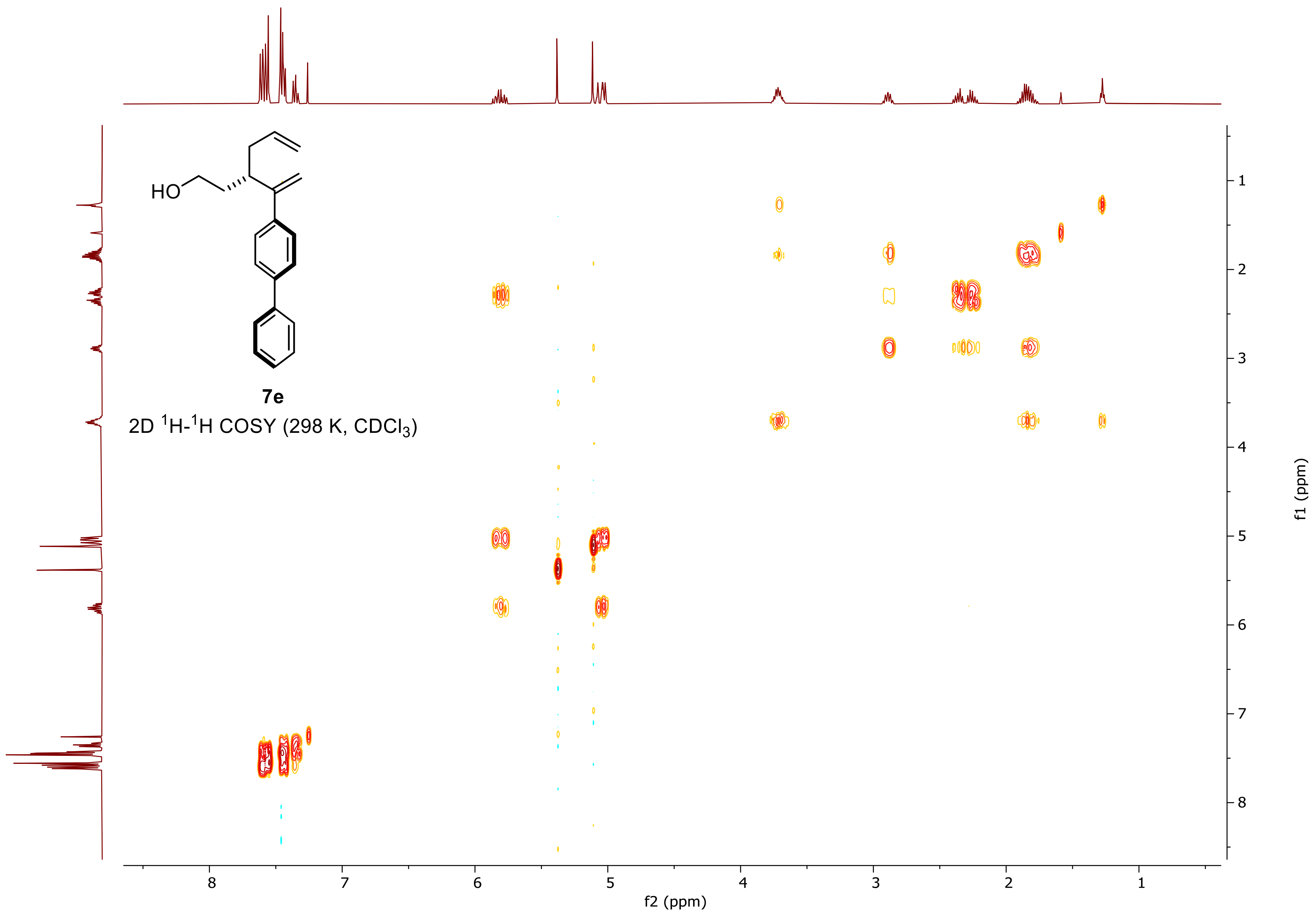
HO
7e
2D 1H-1H COSY (298 K, CDCl3)
f2 (ppm)
f1 (ppm)

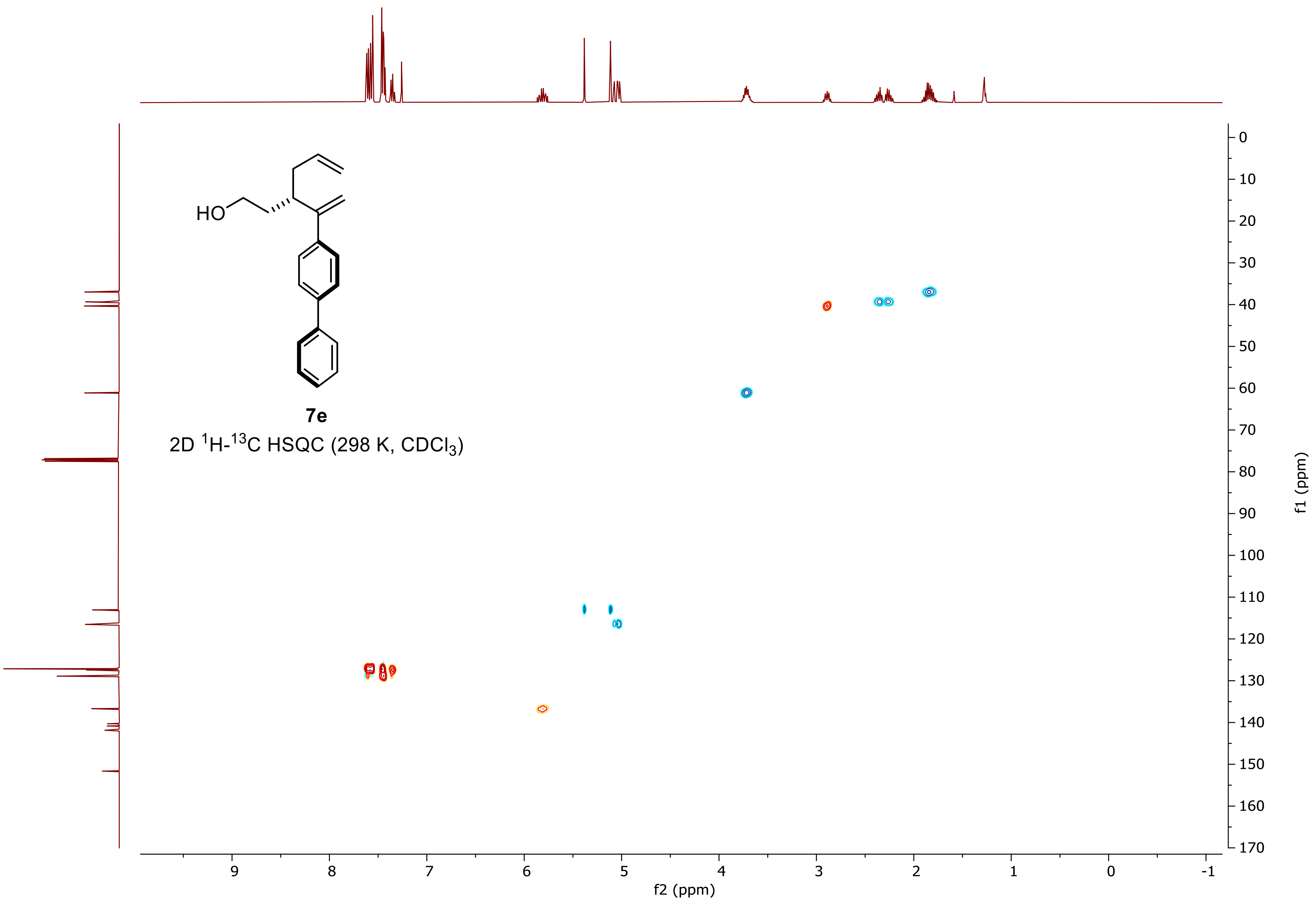
HO
7e
2D 1H-13C HSQC (298 K, CDCl3)
f1 (ppm)
f2 (ppm)

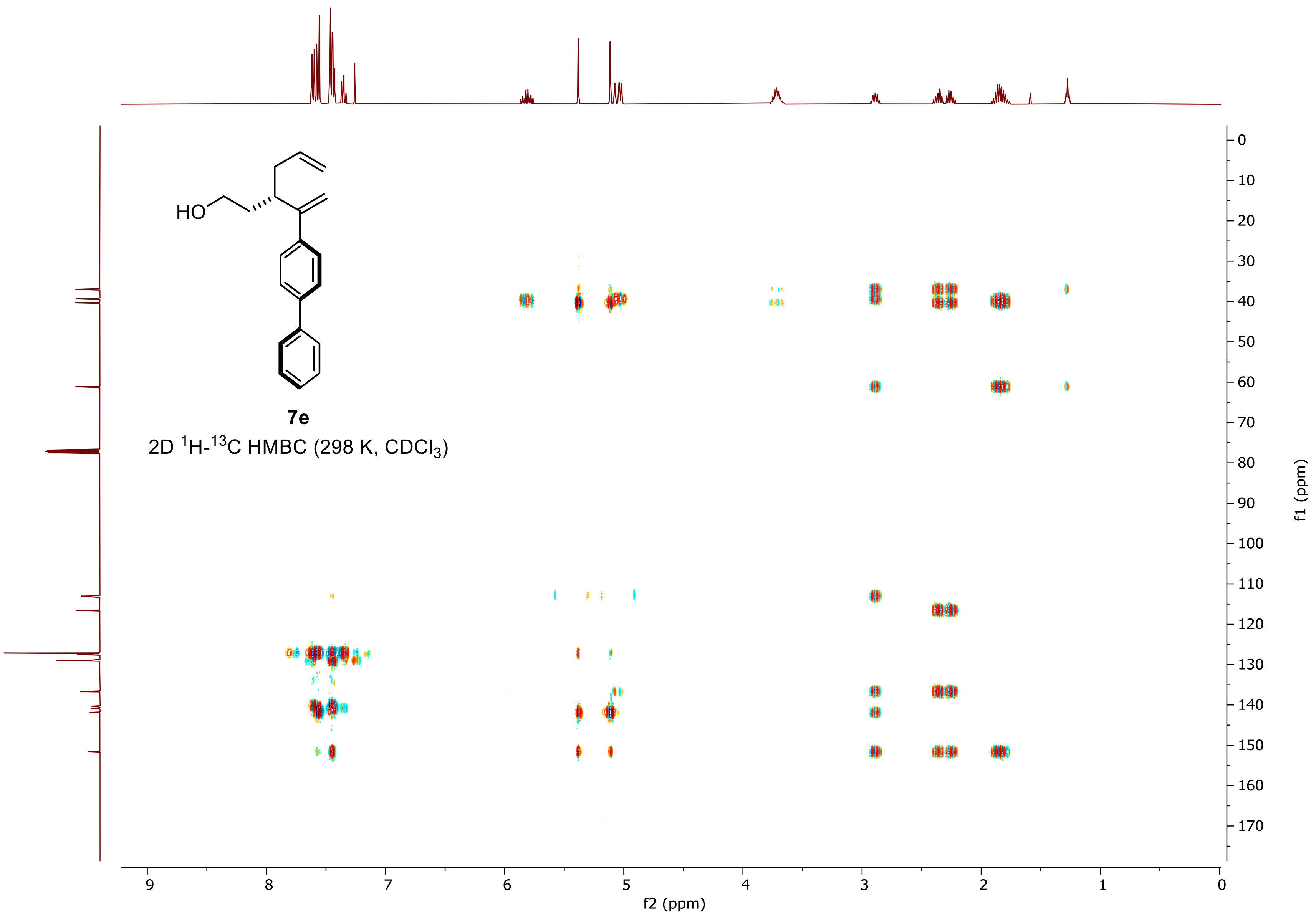

7e

2D ^{1}H-^{13}C HMBC (298 K, $CDCl_3$)

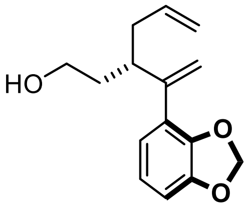

7f

^{1}H NMR (400 MHz, 298 K, $CDCl_3$)

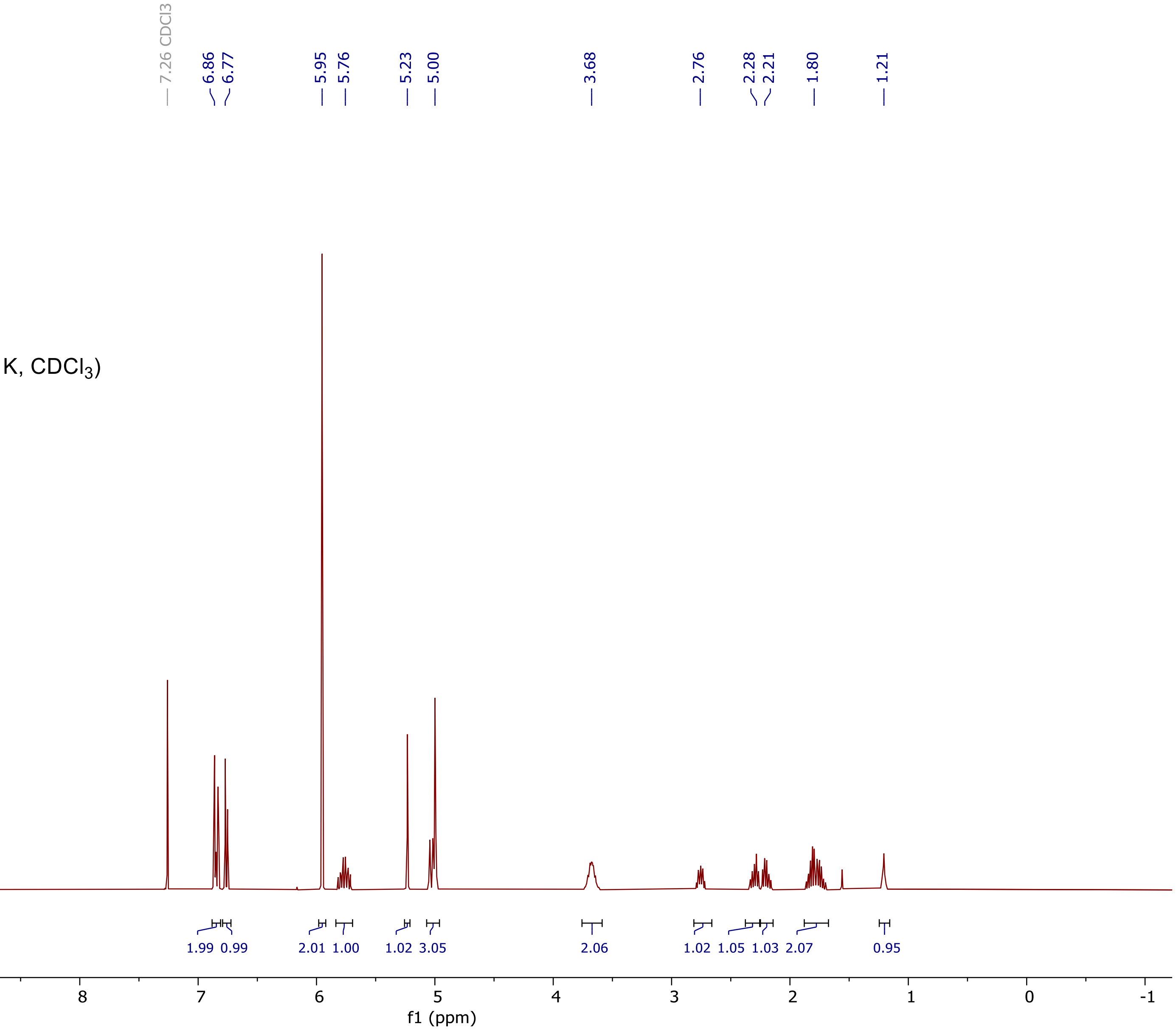

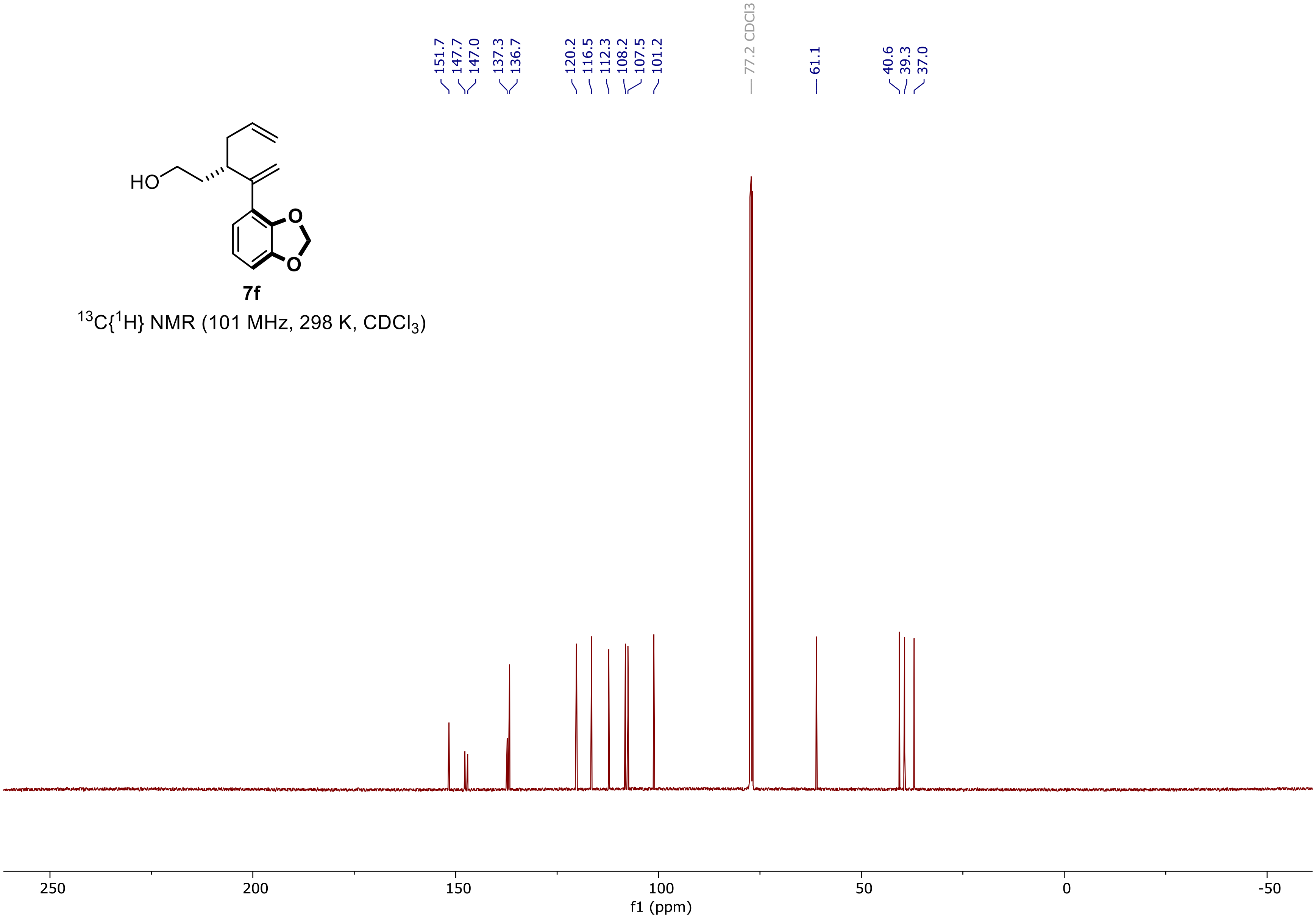

7f

$^{13}C\{^{1}H\}$ NMR (101 MHz, 298 K, $CDCl_3$)

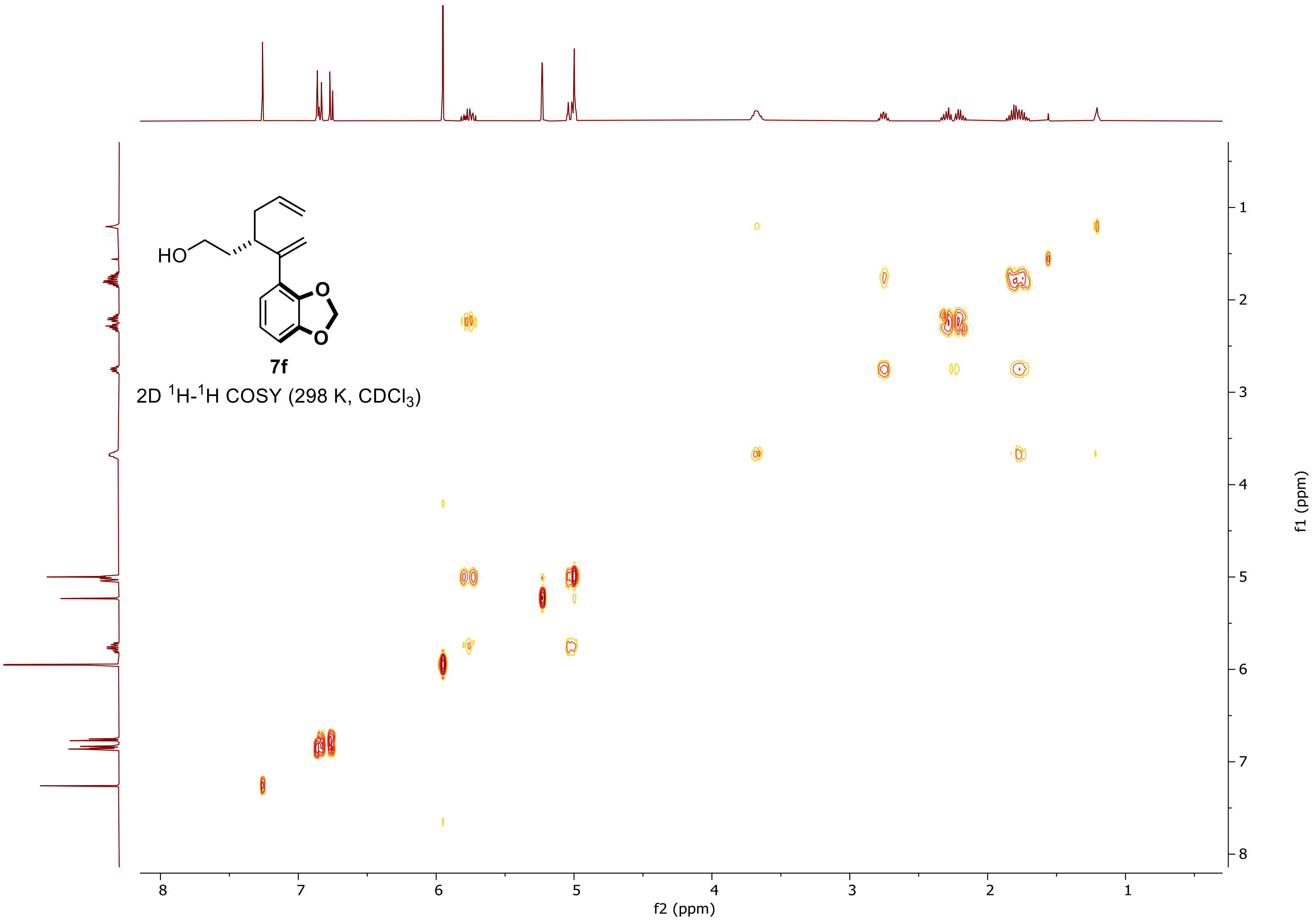
HO
O
O
7f
2D 1H-1H COSY (298 K, CDCl3)
f2 (ppm)
f1 (ppm)
8
7
6
5
4
3
2
1

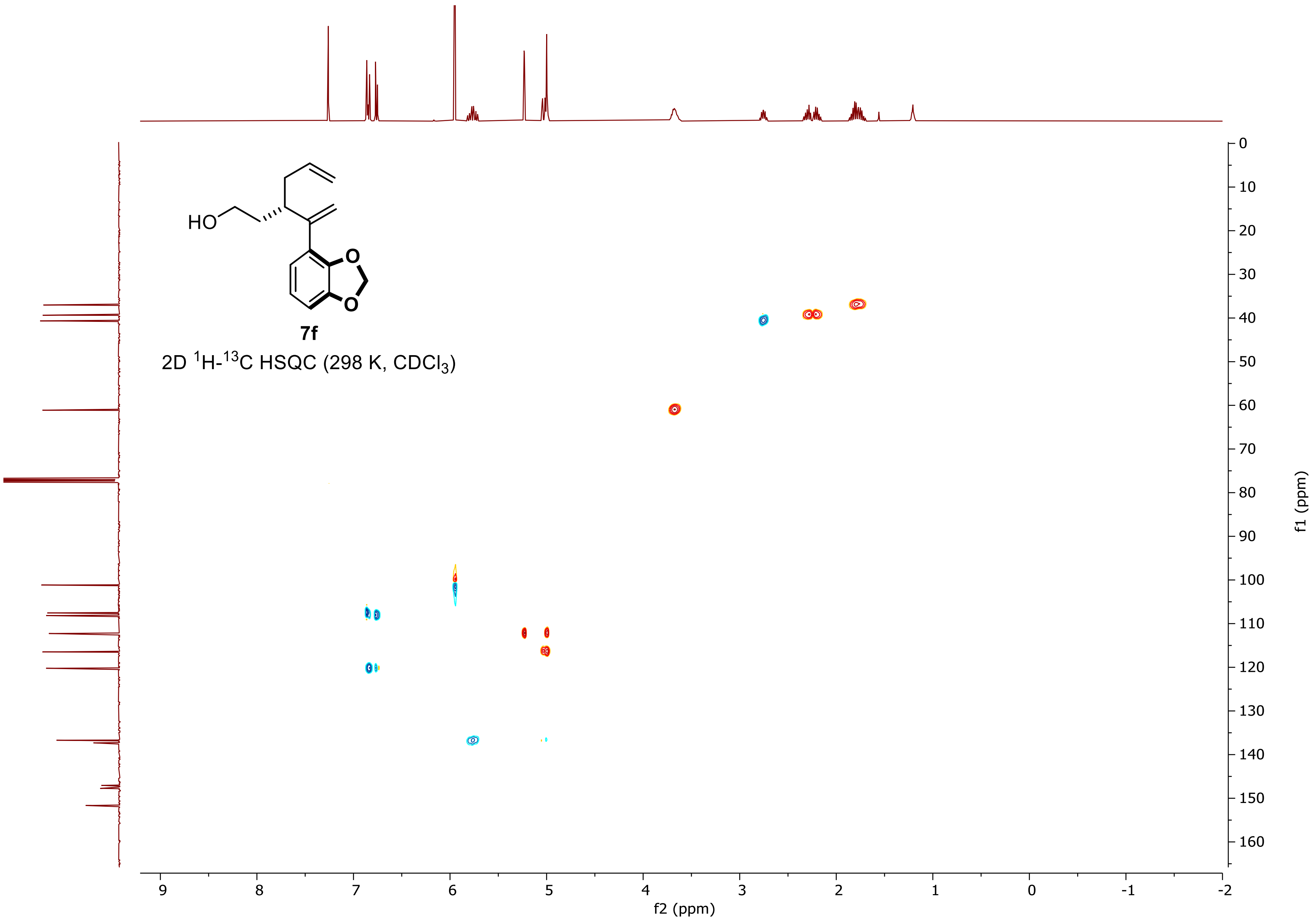
HO
O
O
7f
2D ^{1}H-^{13}C HSQC (298 K, $CDCl_3$)
f2 (ppm)
f1 (ppm)
9
8
7
6
5
4
3
2
1
0
-1
-2
0
10
20
30
40
50
60
70
80
90
100
110
120
130
140
150
160

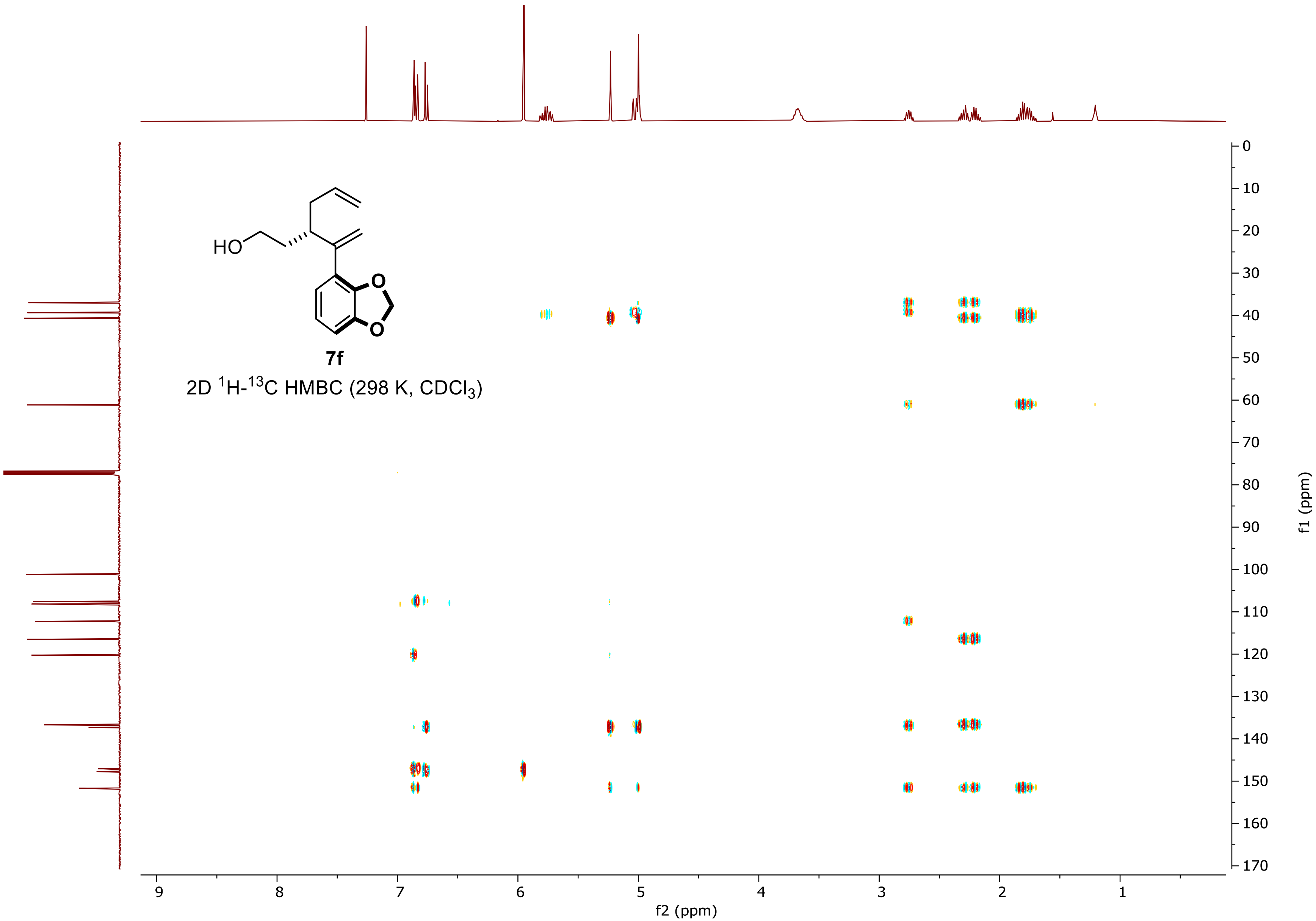
HO
O
O
7f
2D ¹H-¹³C HMBC (298 K, CDCl₃)
f2 (ppm)
f1 (ppm)
9
8
7
6
5
4
3
2
1
0
10
20
30
40
50
60
70
80
90
100
110
120
130
140
150
160
170

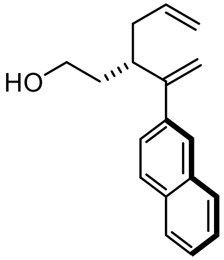

7g

^{1}H NMR (400 MHz, 298 K, $CDCl_3$)

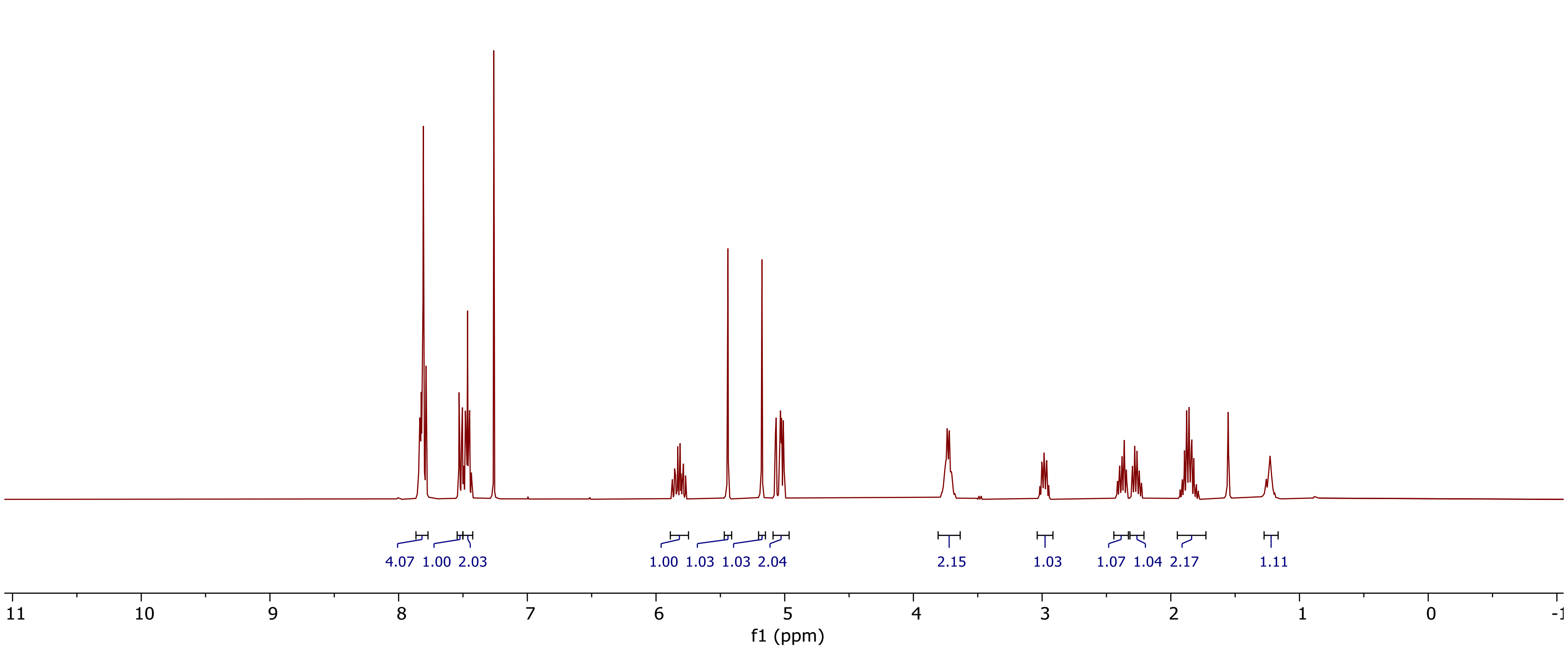

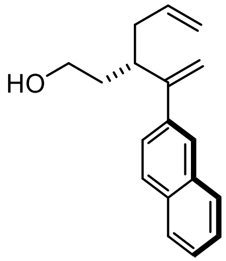

7g

$^{13}C\{^{1}H\}$ NMR (101 MHz, 298 K, $CDCl_3$)

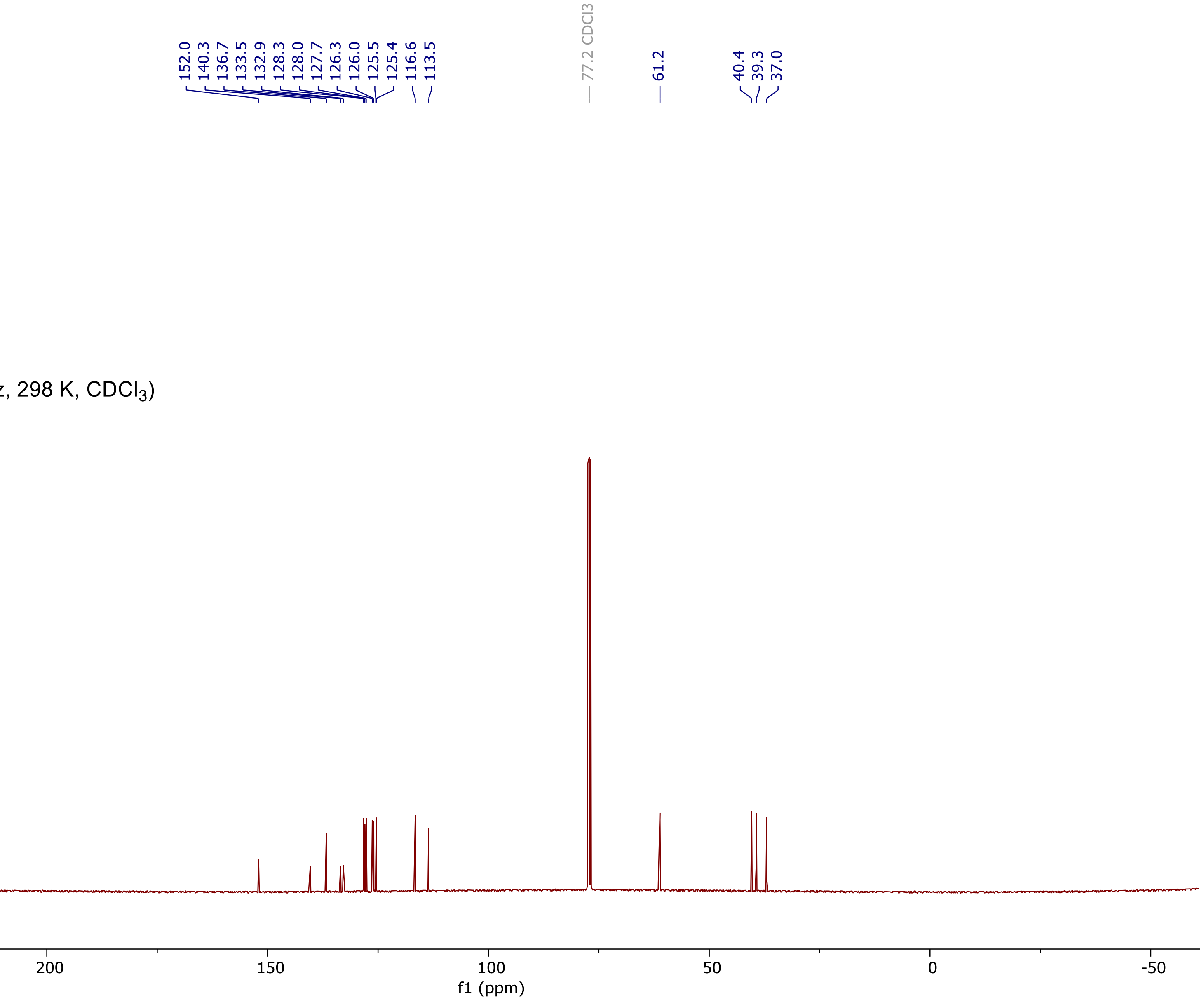

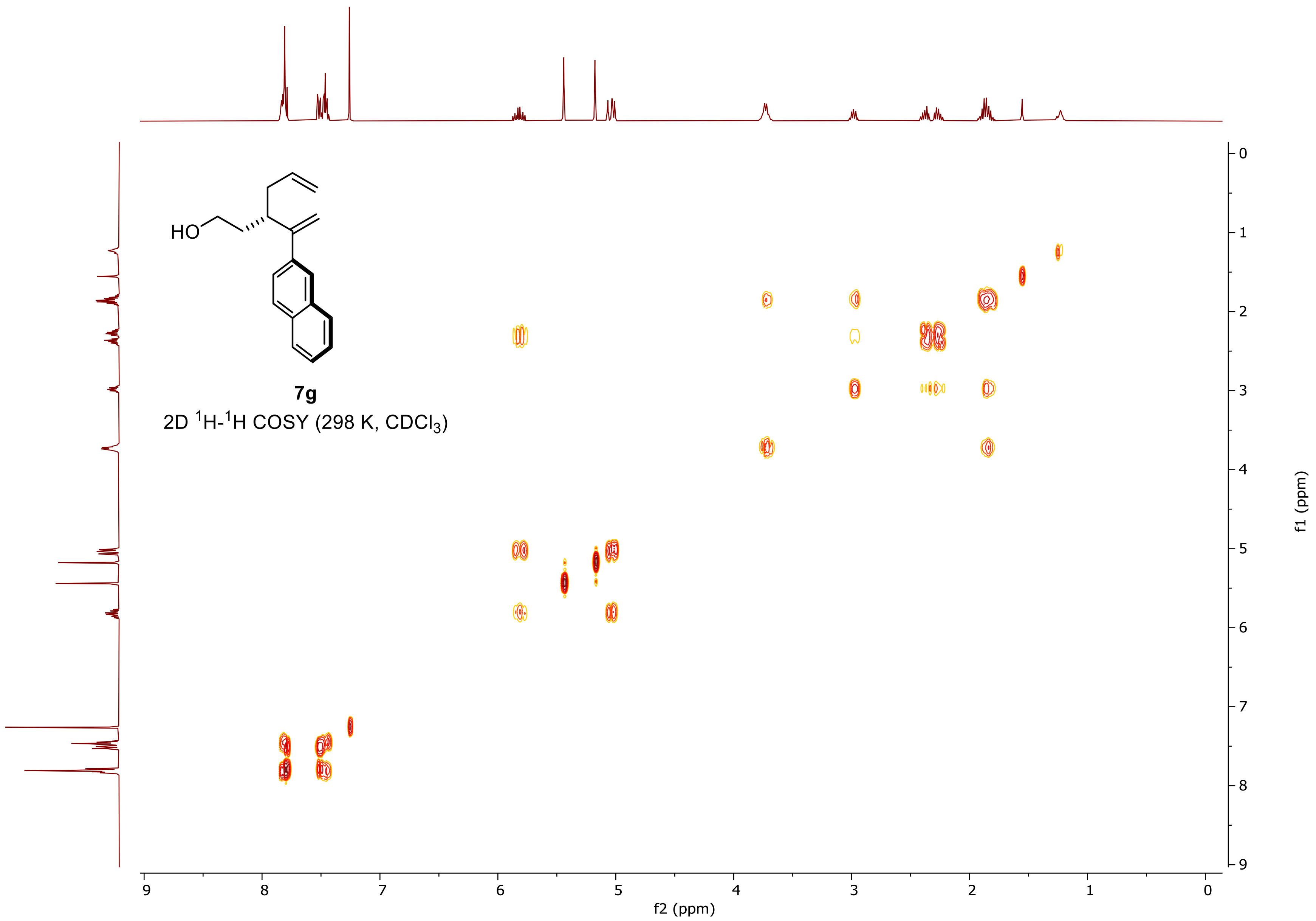
HO
7g
2D ^1H-^1H COSY (298 K, $CDCl_3$)
f2 (ppm)
f1 (ppm)
9
8
7
6
5
4
3
2
1
0

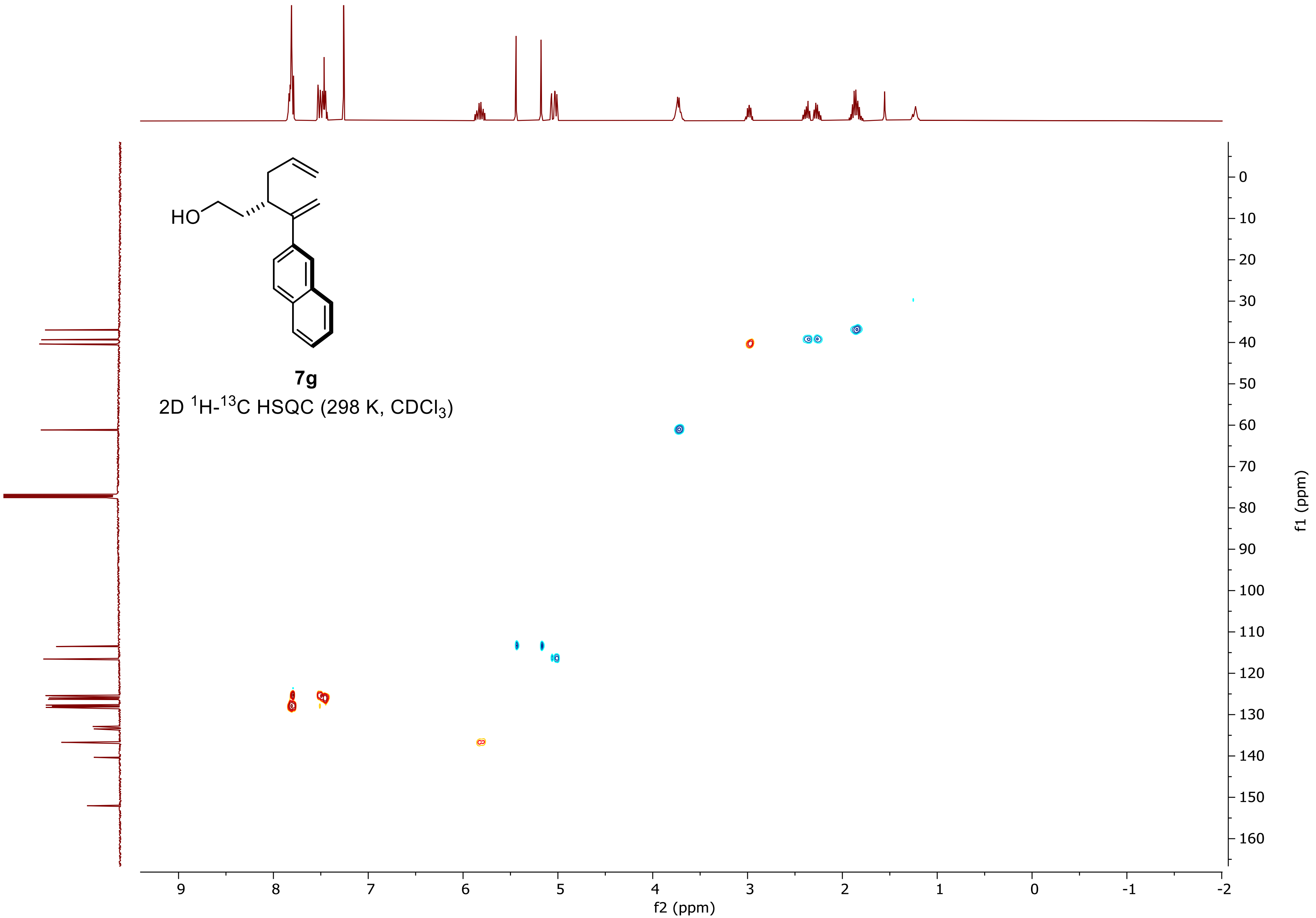

7g

2D ^{1}H-^{13}C HSQC (298 K, $CDCl_3$)

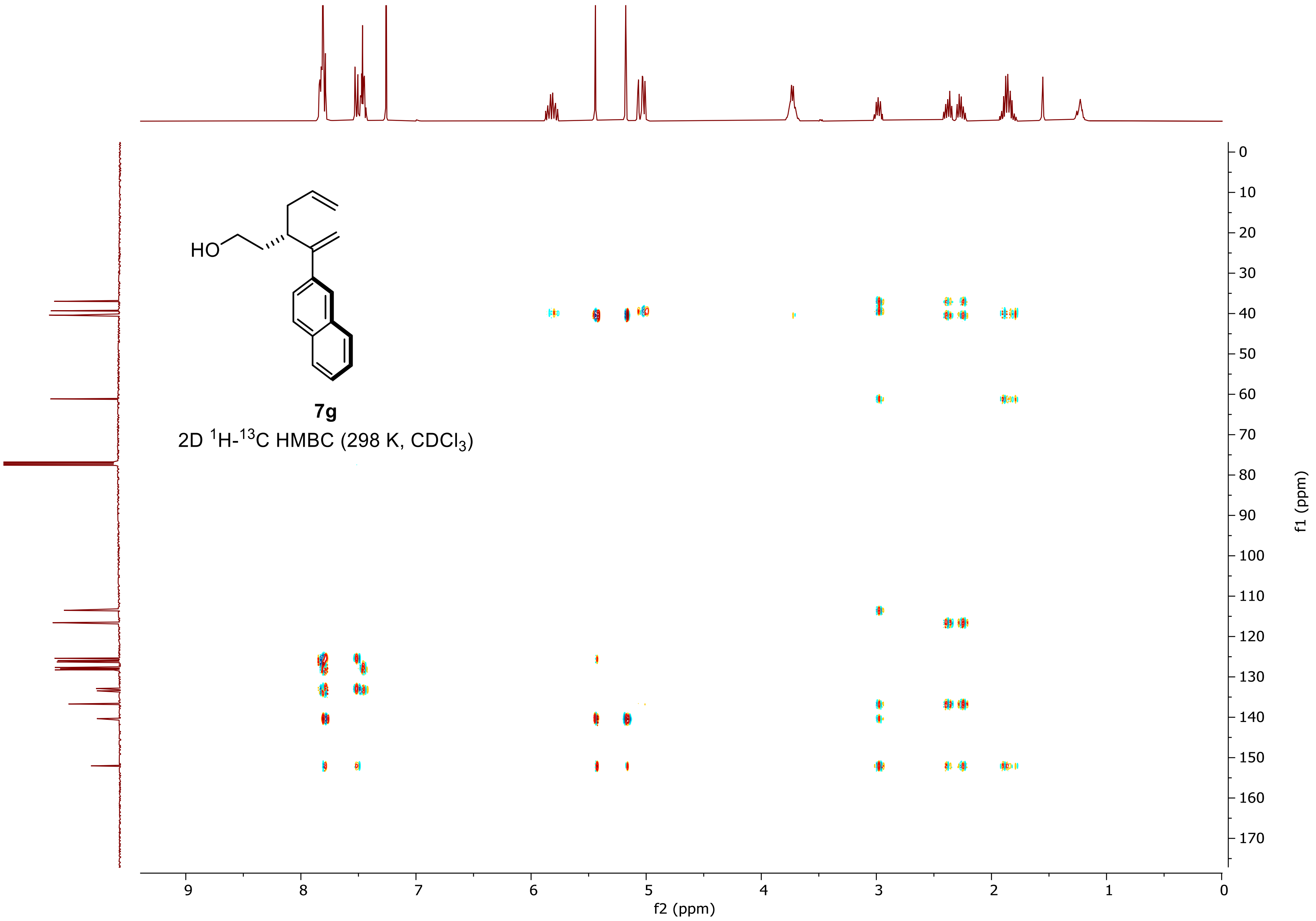
HO
7g
2D 1H-13C HMBC (298 K, CDCl3)
f1 (ppm)
f2 (ppm)

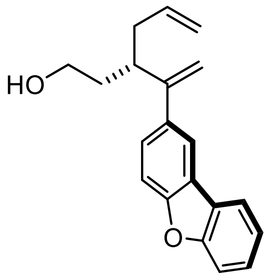

7h

^{1}H NMR (400 MHz, 298 K, $CDCl_3$)

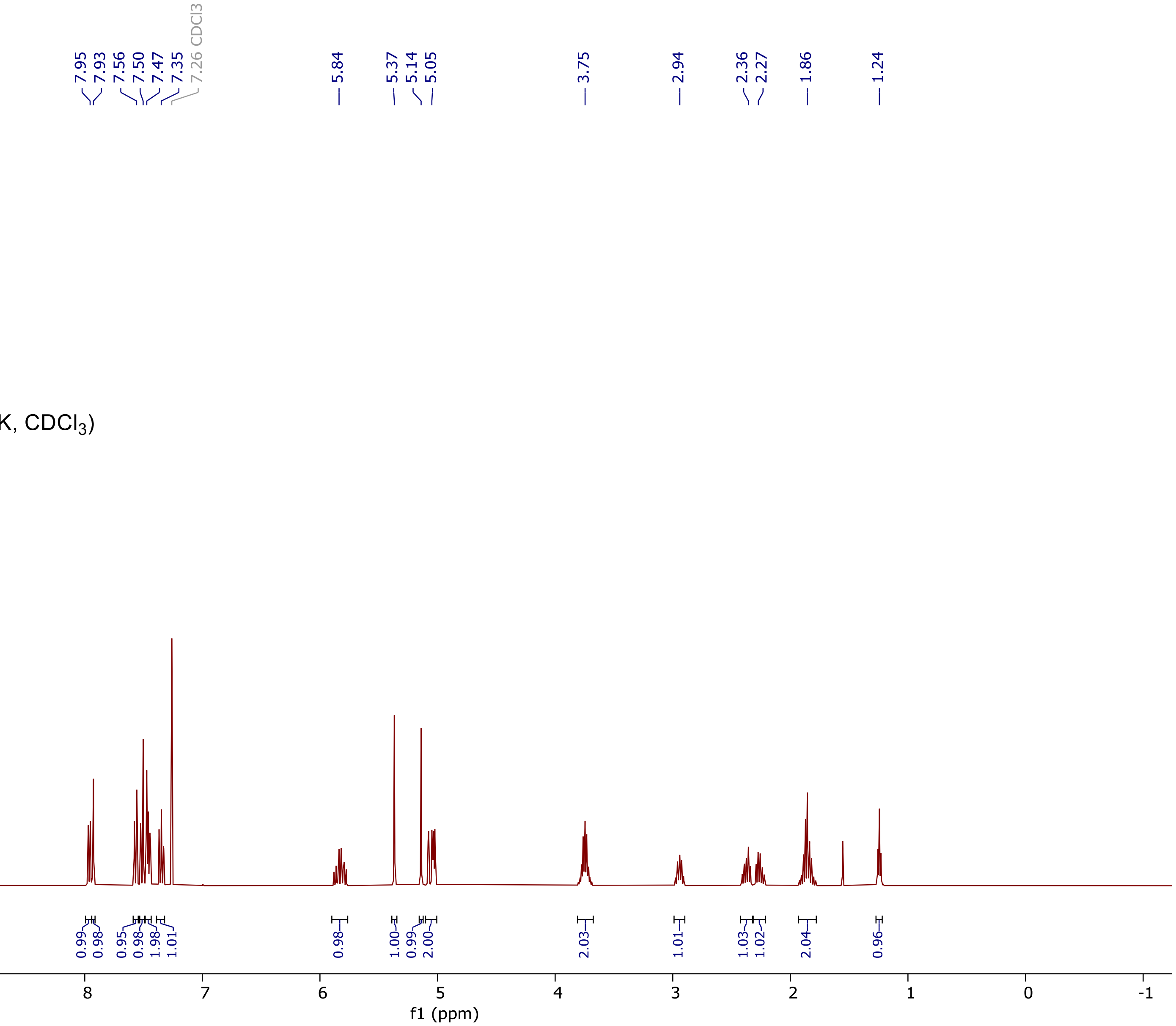

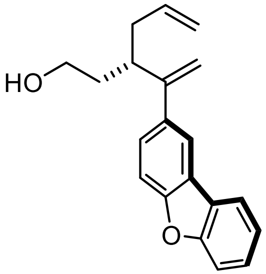

7h

$^{13}C\{^{1}H\}$ NMR (101 MHz, 298 K, $CDCl_3$)

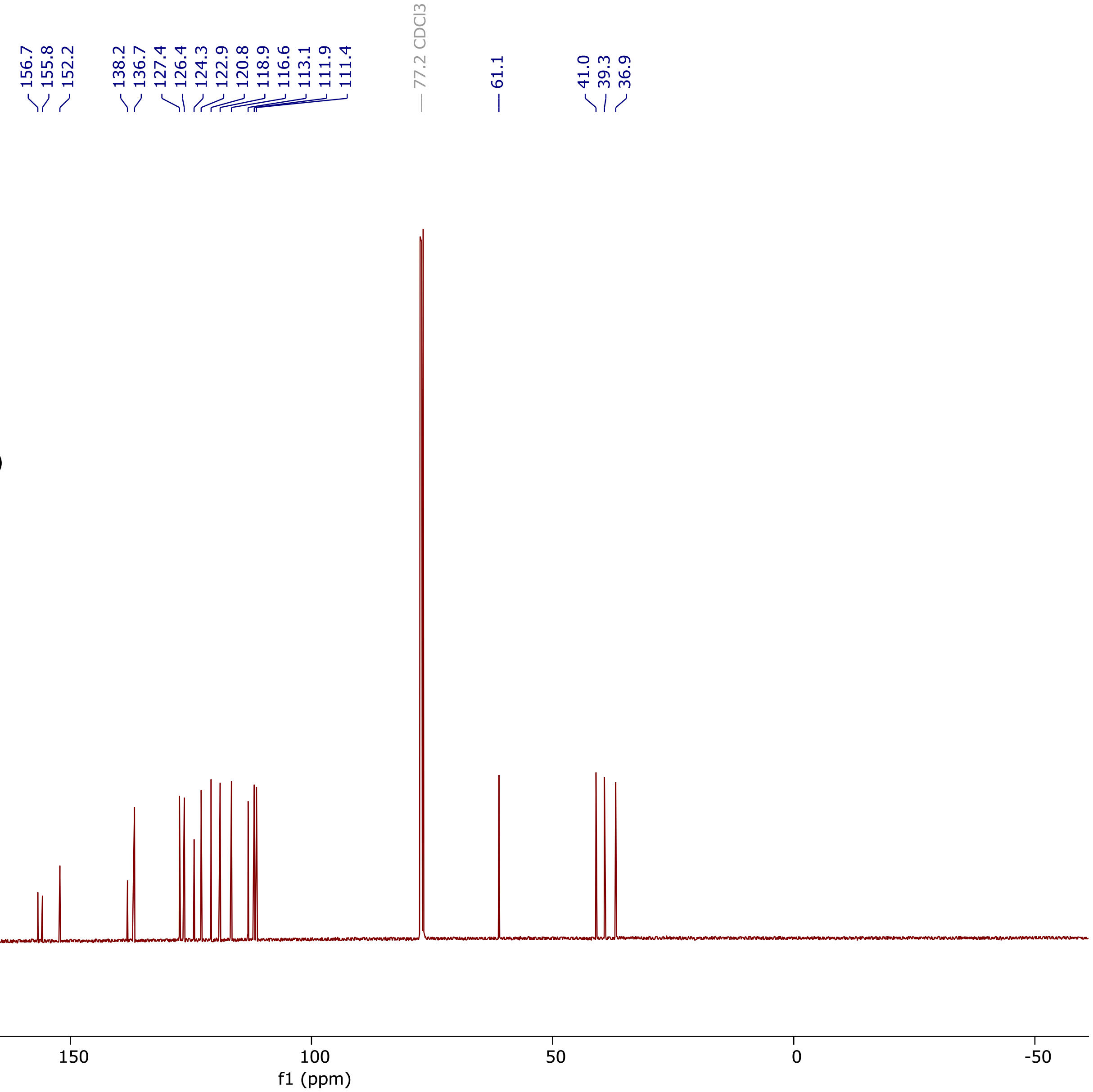

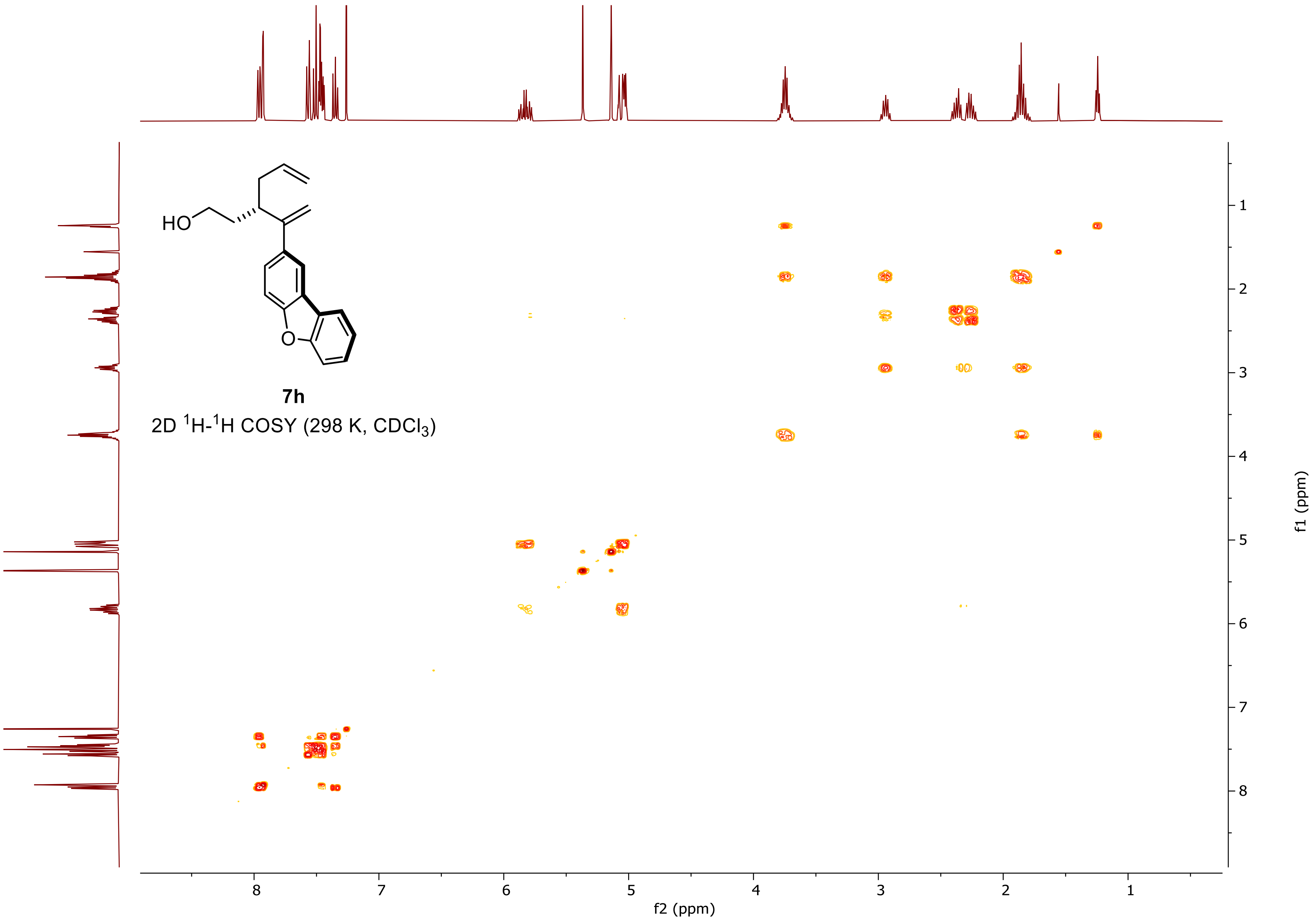

7h

2D ^{1}H-^{1}H COSY (298 K, $CDCl_3$)

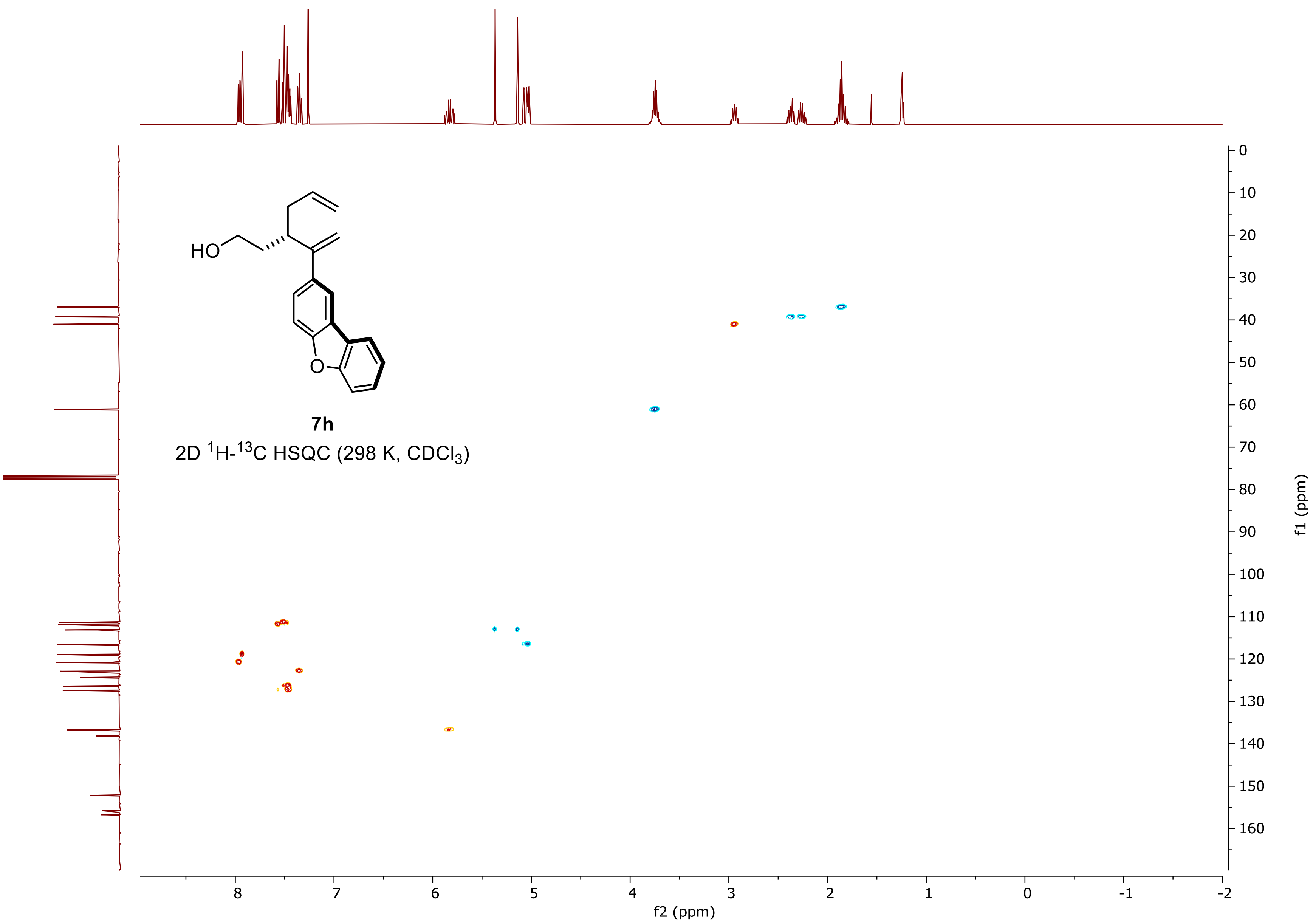

7h

2D ^{1}H-^{13}C HSQC (298 K, $CDCl_3$)

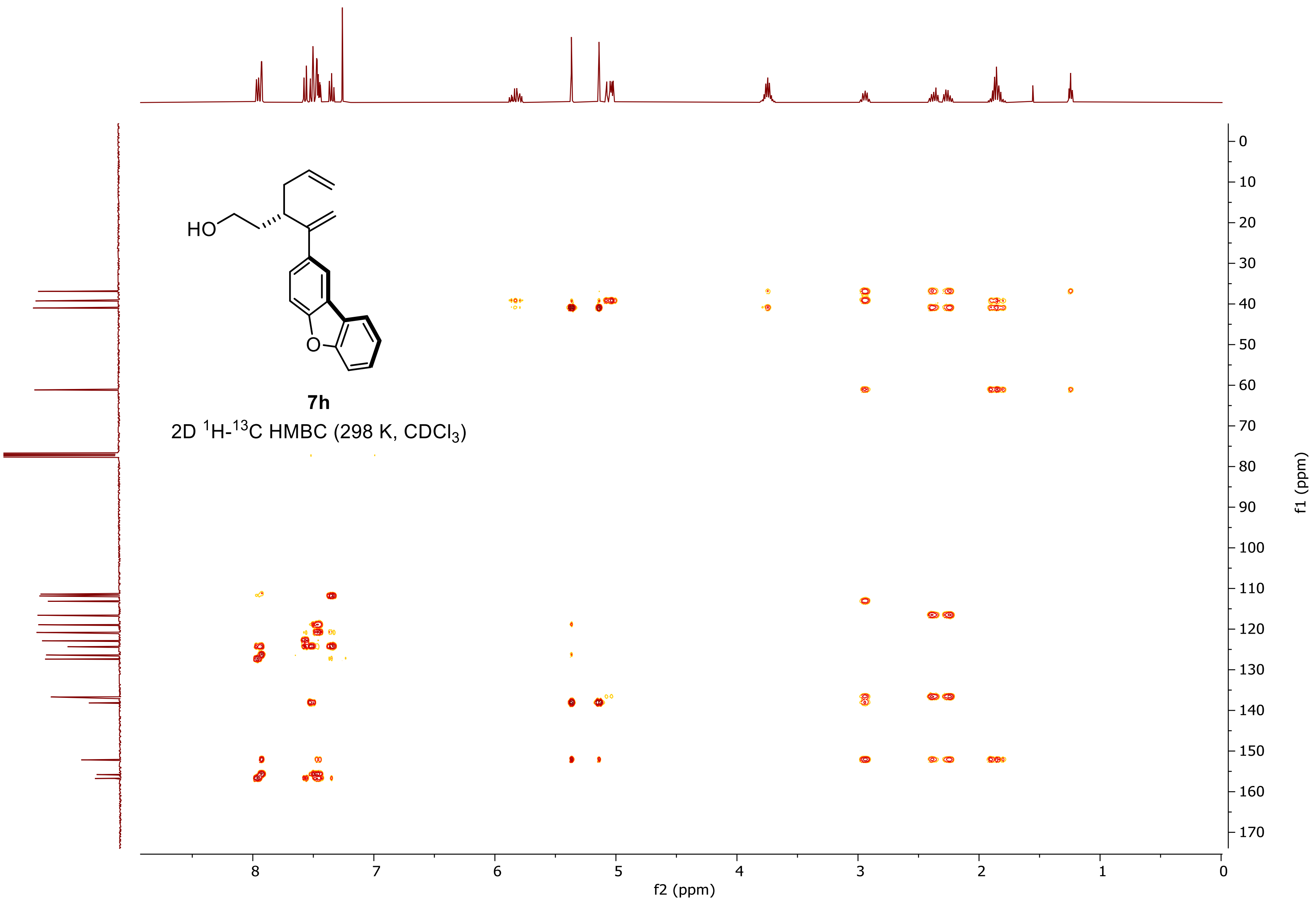
HO
O
7h
2D ^{1}H-^{13}C HMBC (298 K, $CDCl_3$)
f2 (ppm)
f1 (ppm)

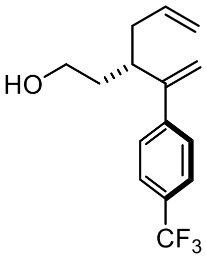

7i

^{1}H NMR (400 MHz, 298 K, $CDCl_3$)

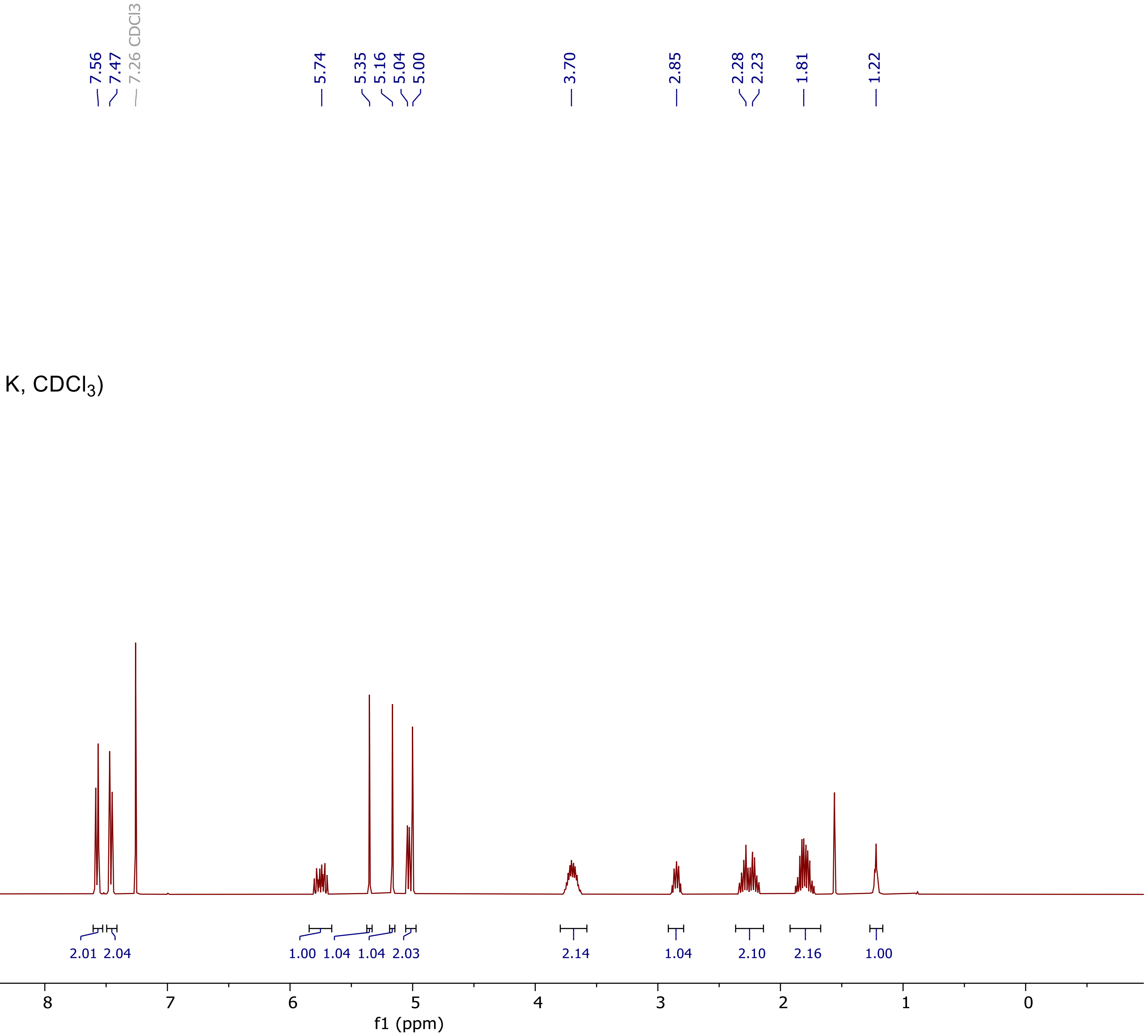

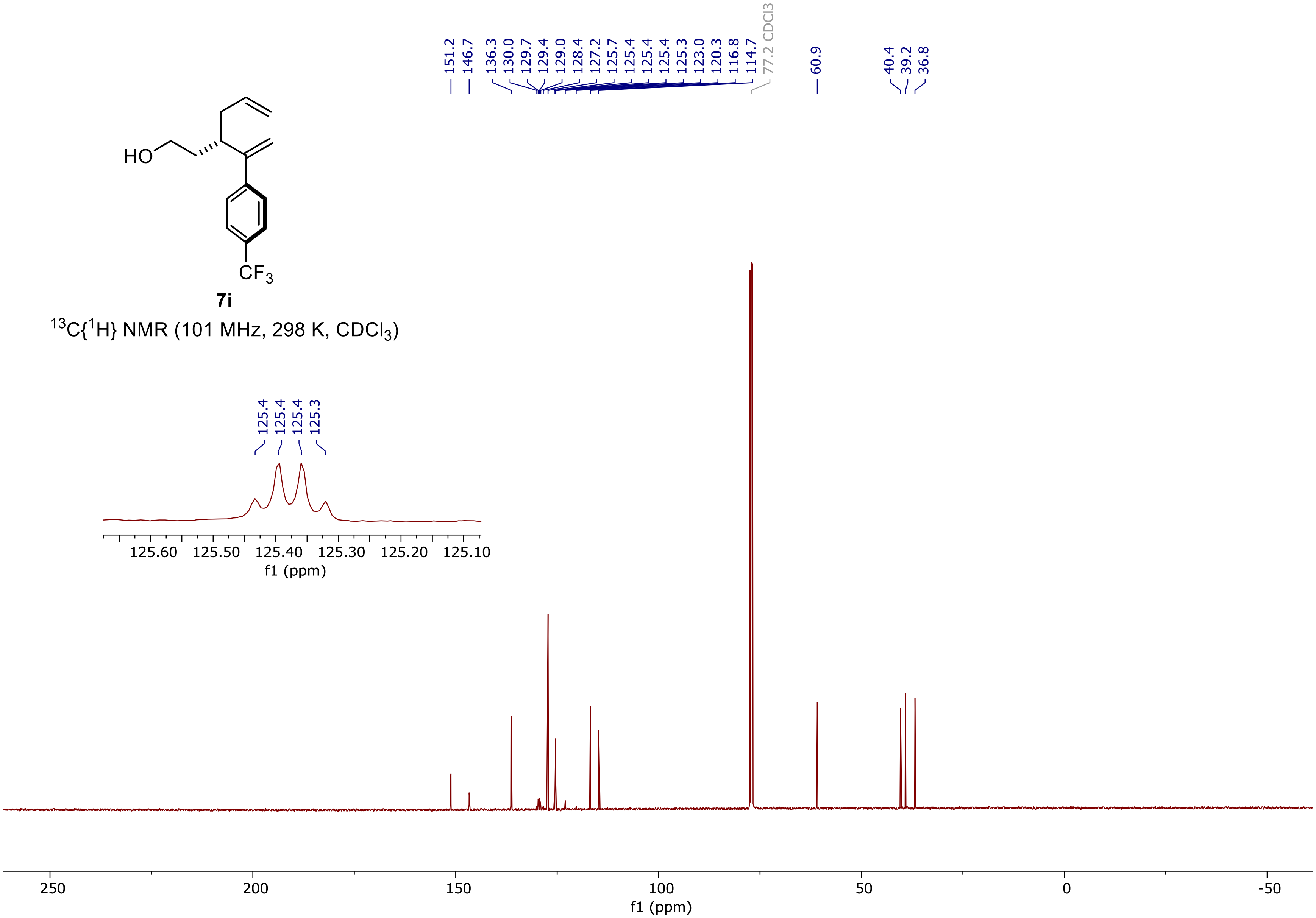

7i

$^{13}C\{^{1}H\}$ NMR (101 MHz, 298 K, $CDCl_3$)

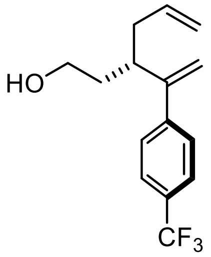

7i

$^{19}F\{^1H\}$ NMR (282 MHz, 298 K, $CDCl_3$)

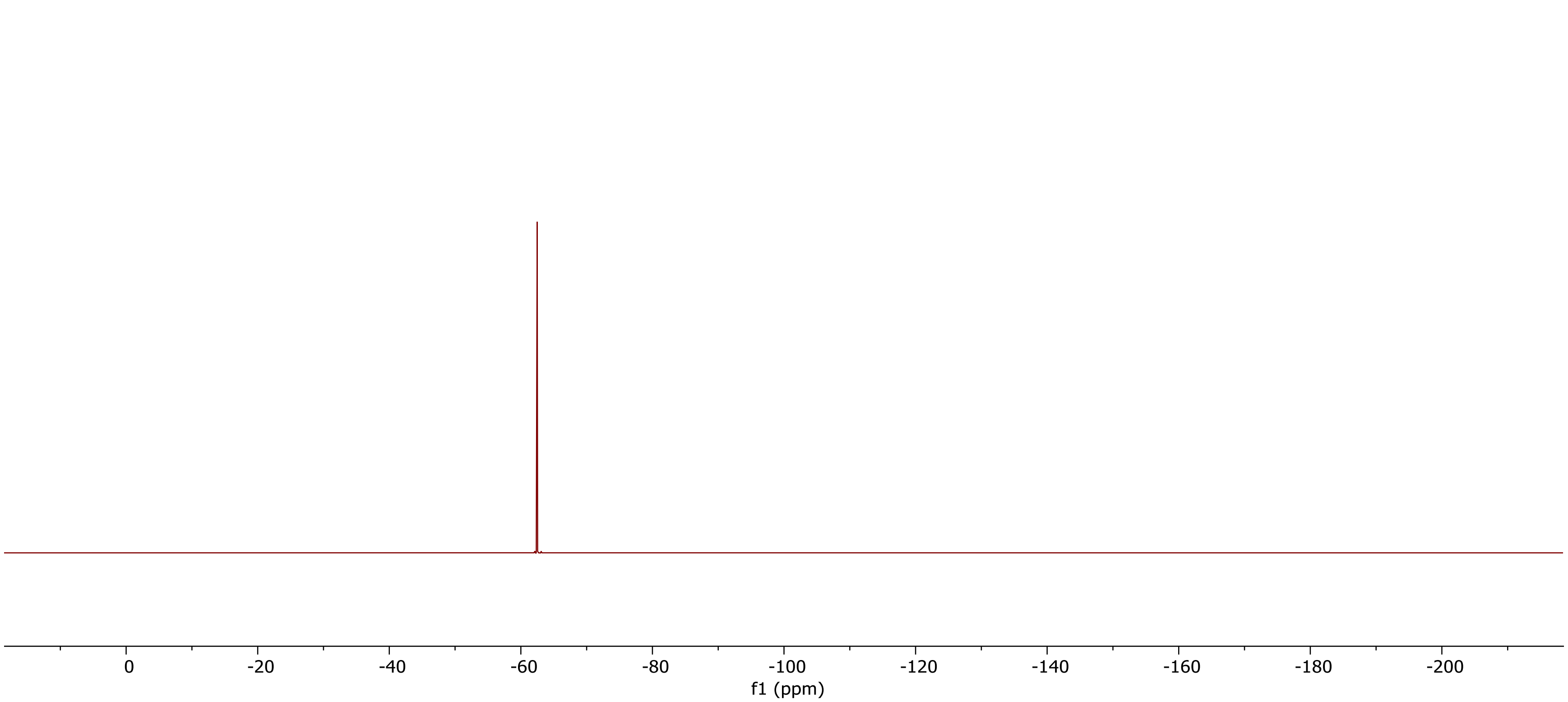

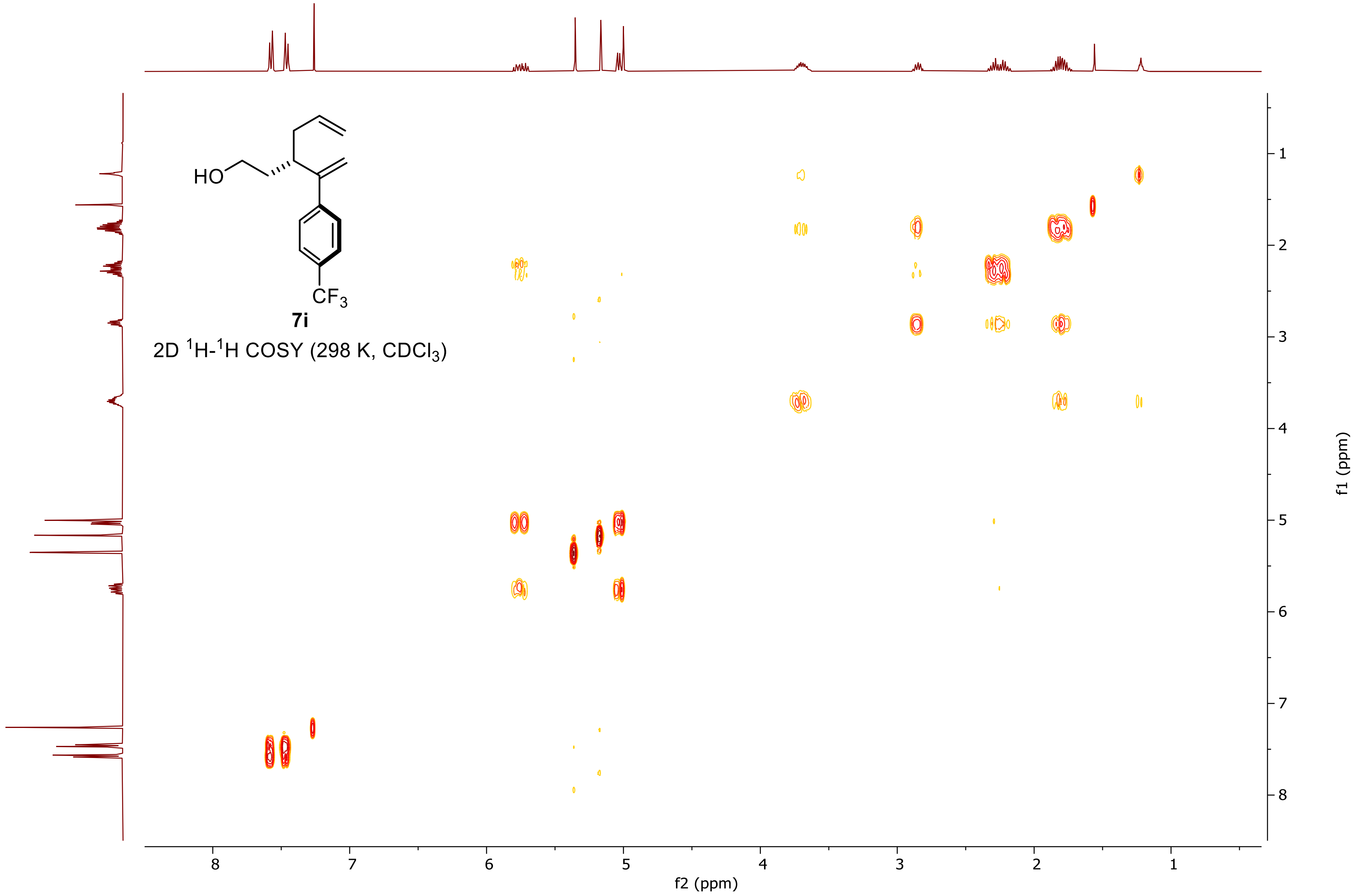
HO
CF_3
7i
2D ^1H-^1H COSY (298 K, $CDCl_3$)
f1 (ppm)
f2 (ppm)
8
7
6
5
4
3
2
1

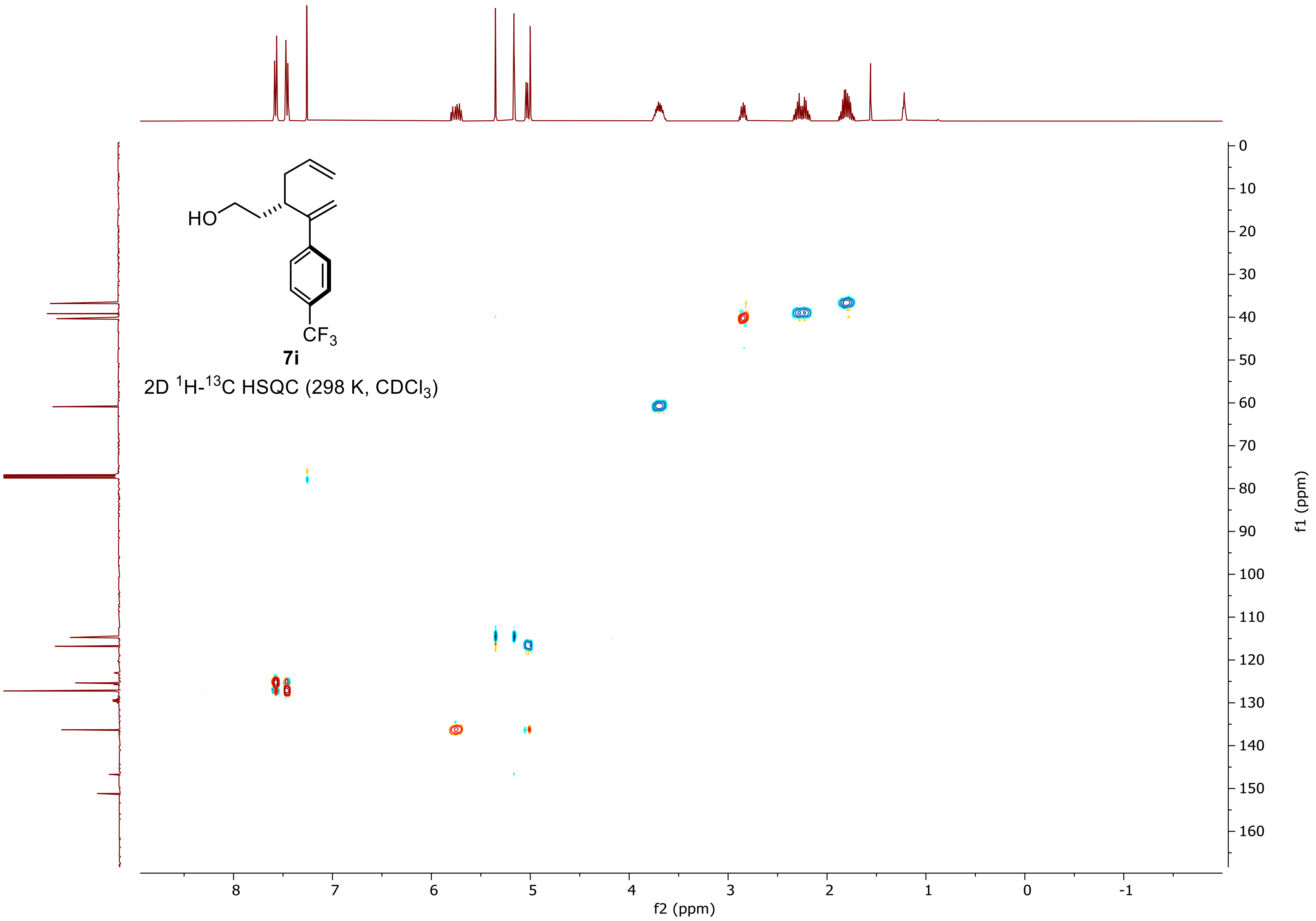
HO
CF3
7i
2D 1H-13C HSQC (298 K, CDCl3)
f2 (ppm)
f1 (ppm)

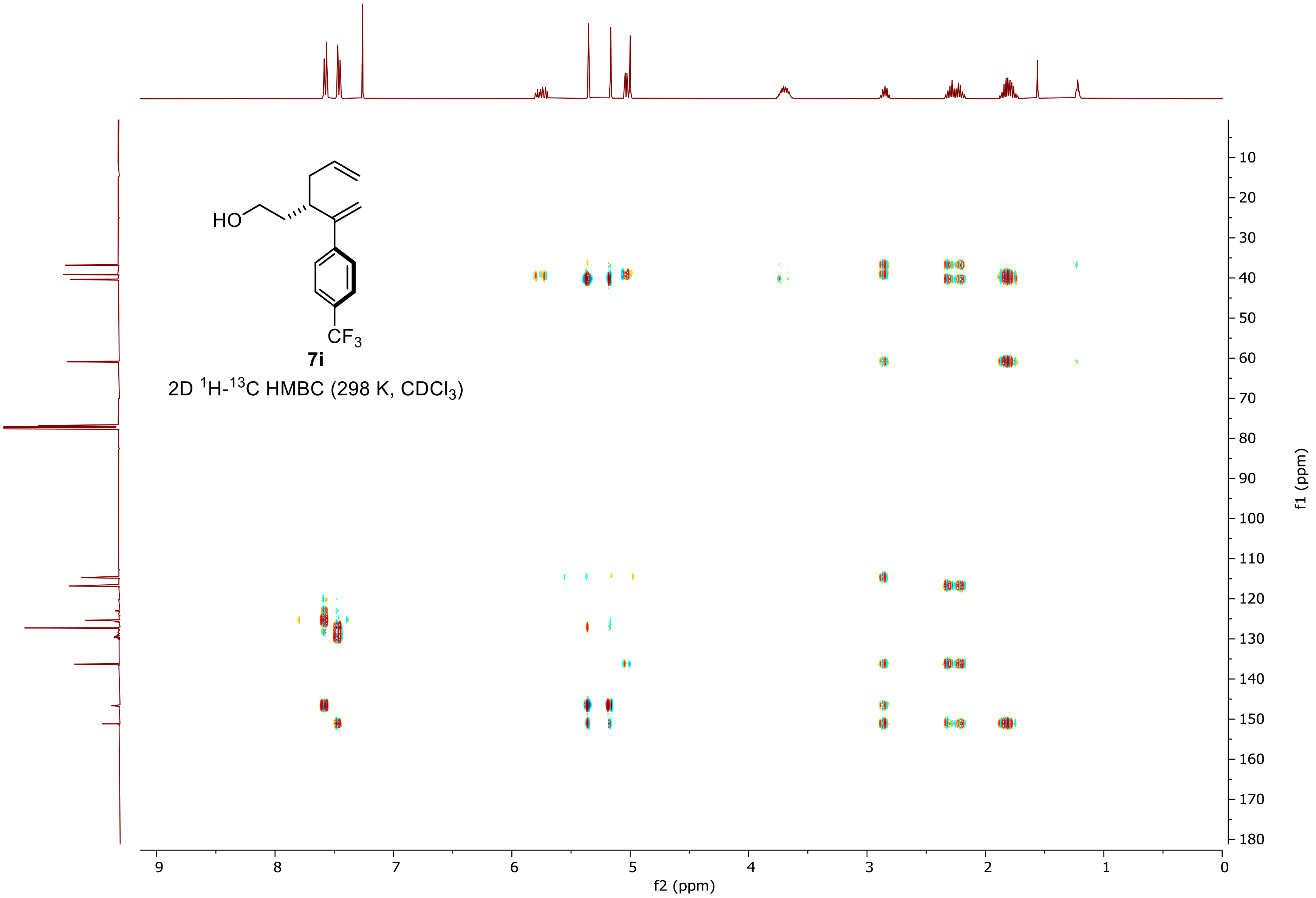
HO
CF_3
7i
2D ^{1}H-^{13}C HMBC (298 K, $CDCl_3$)
f1 (ppm)
f2 (ppm)
10
20
30
40
50
60
70
80
90
100
110
120
130
140
150
160
170
180
9
8
7
6
5
4
3
2
1
0

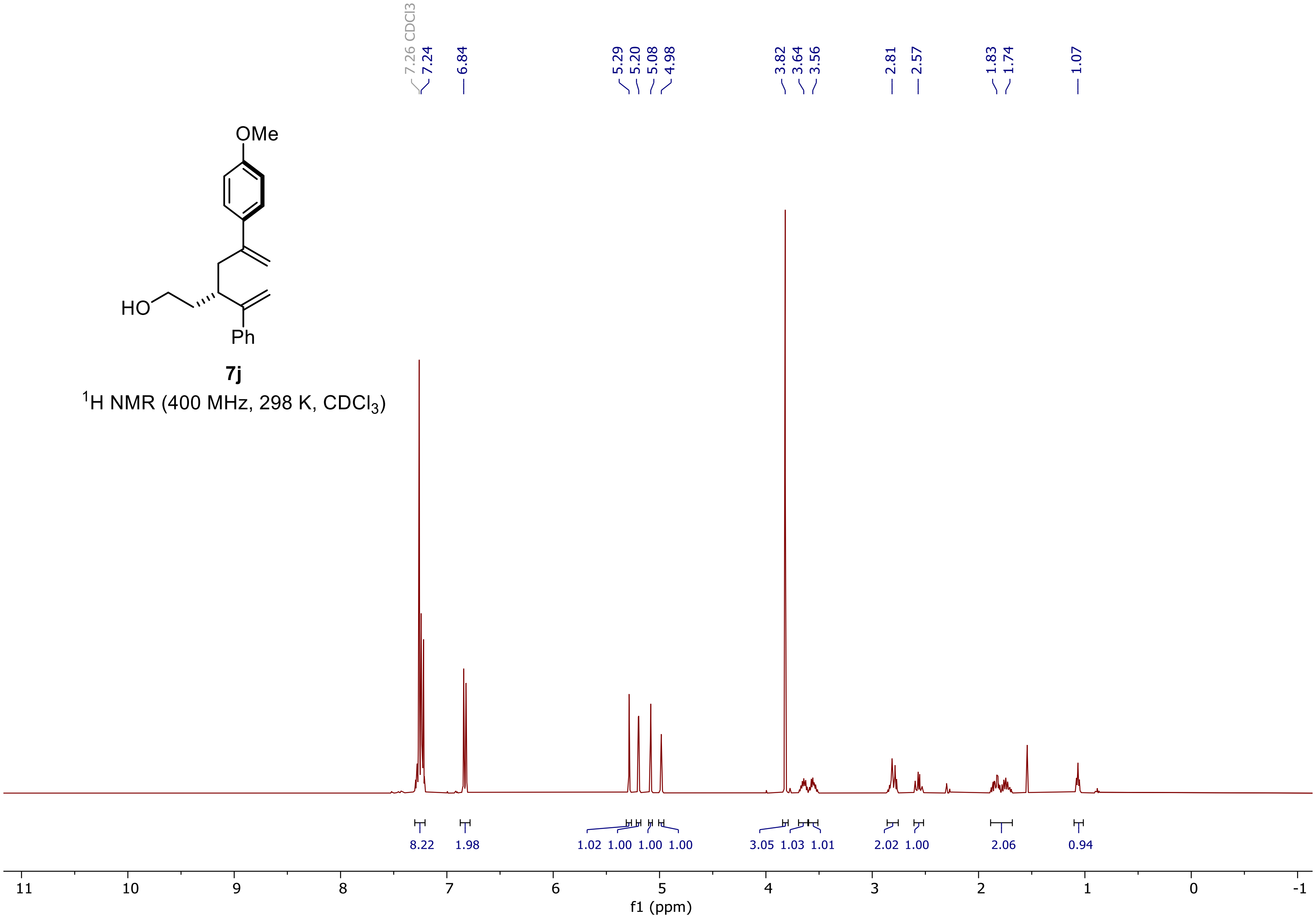

7j

^{1}H NMR (400 MHz, 298 K, $CDCl_3$)

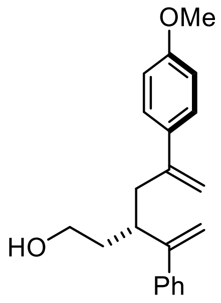

7j

$^{13}C\{^1H\}$ NMR (101 MHz, 298 K, $CDCl_3$)

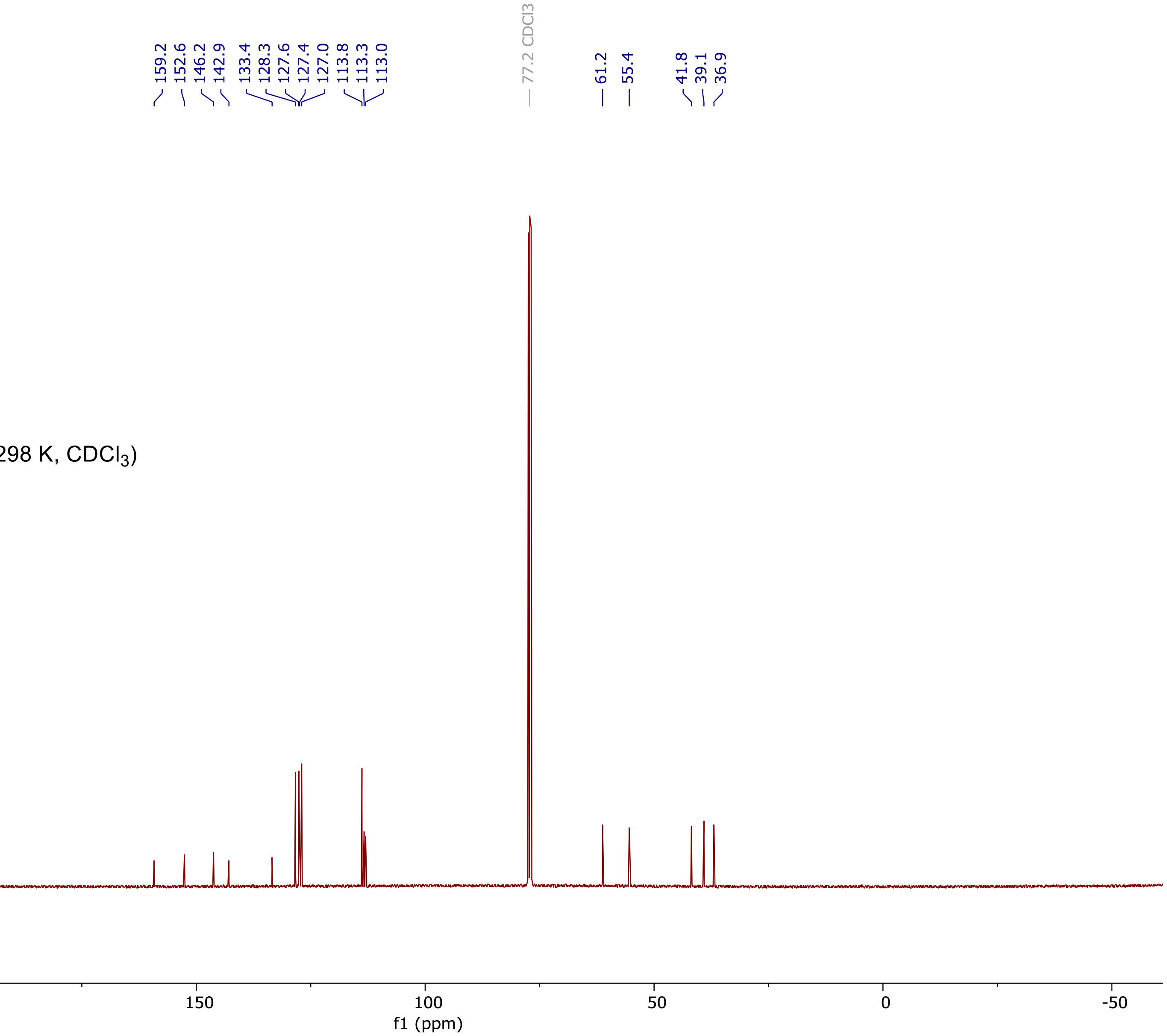

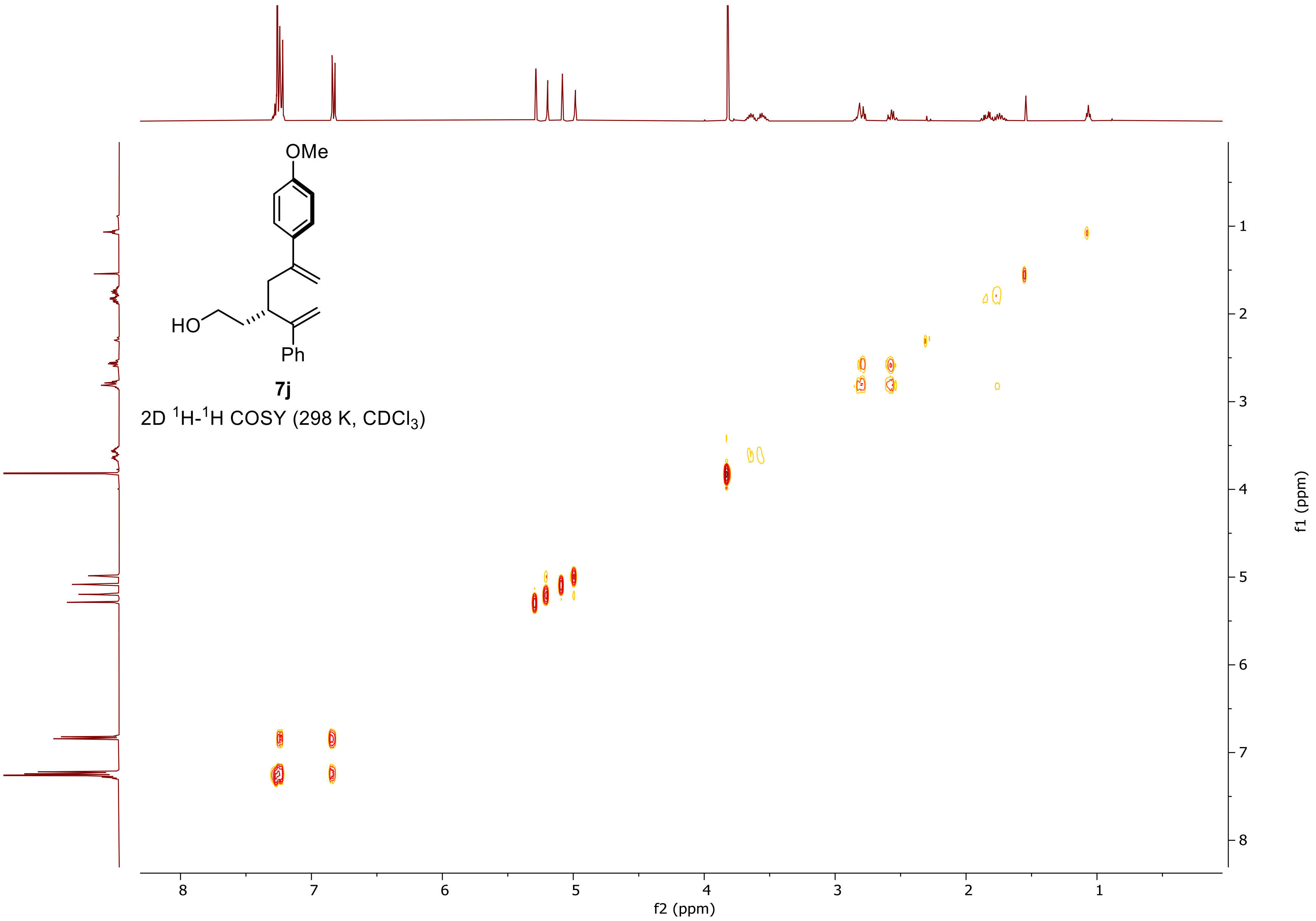

7j

2D ^1H-^1H COSY (298 K, $CDCl_3$)

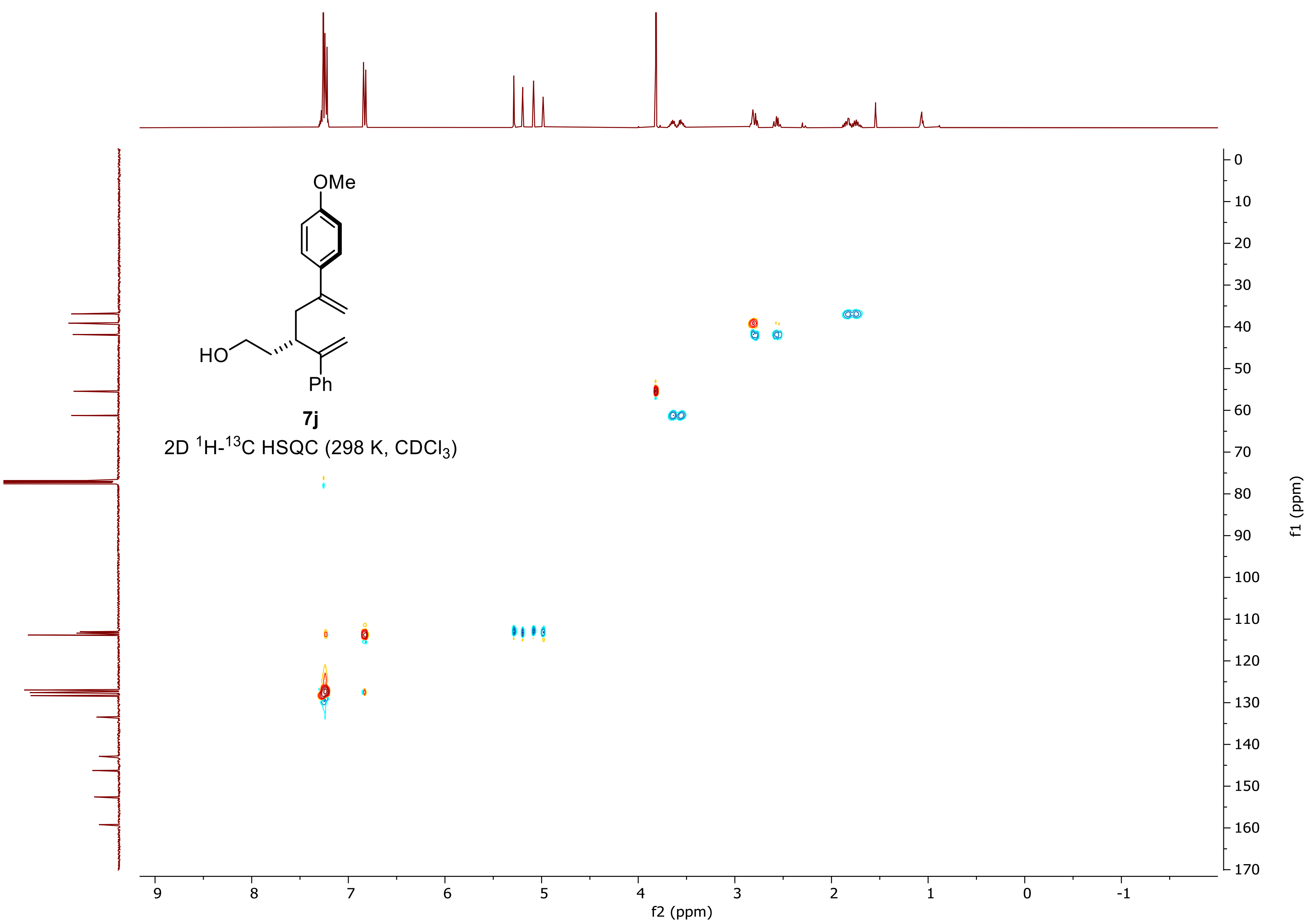
OMe
HO
Ph
7j
2D ^{1}H-^{13}C HSQC (298 K, CDCl$_3$)
f2 (ppm)
f1 (ppm)

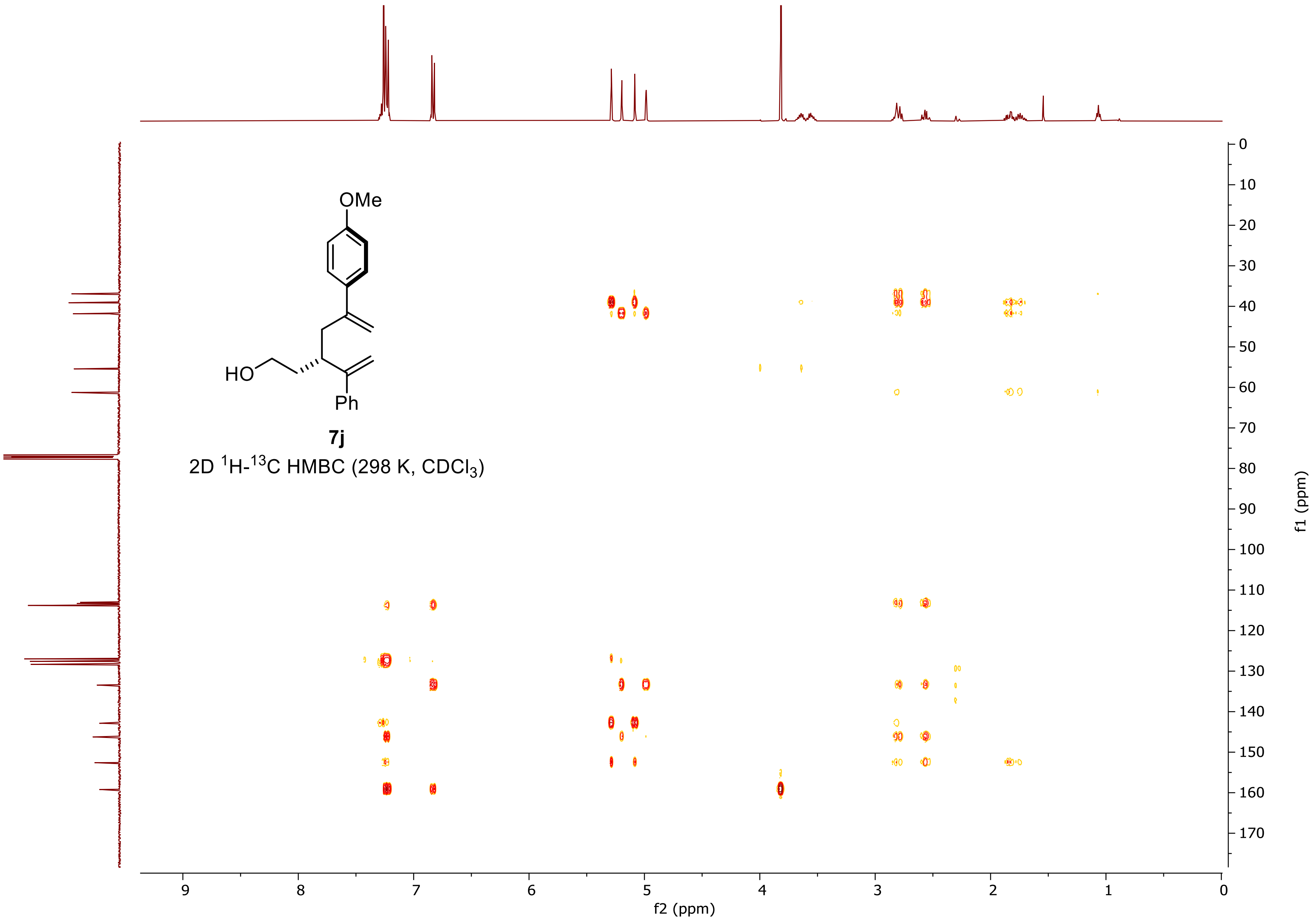

7j

2D ^{1}H-^{13}C HMBC (298 K, $CDCl_3$)

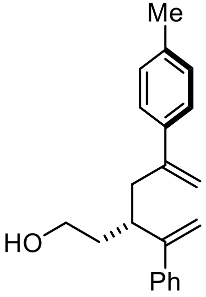

7k
^{1}H NMR (400 MHz, 298 K, $CDCl_3$)

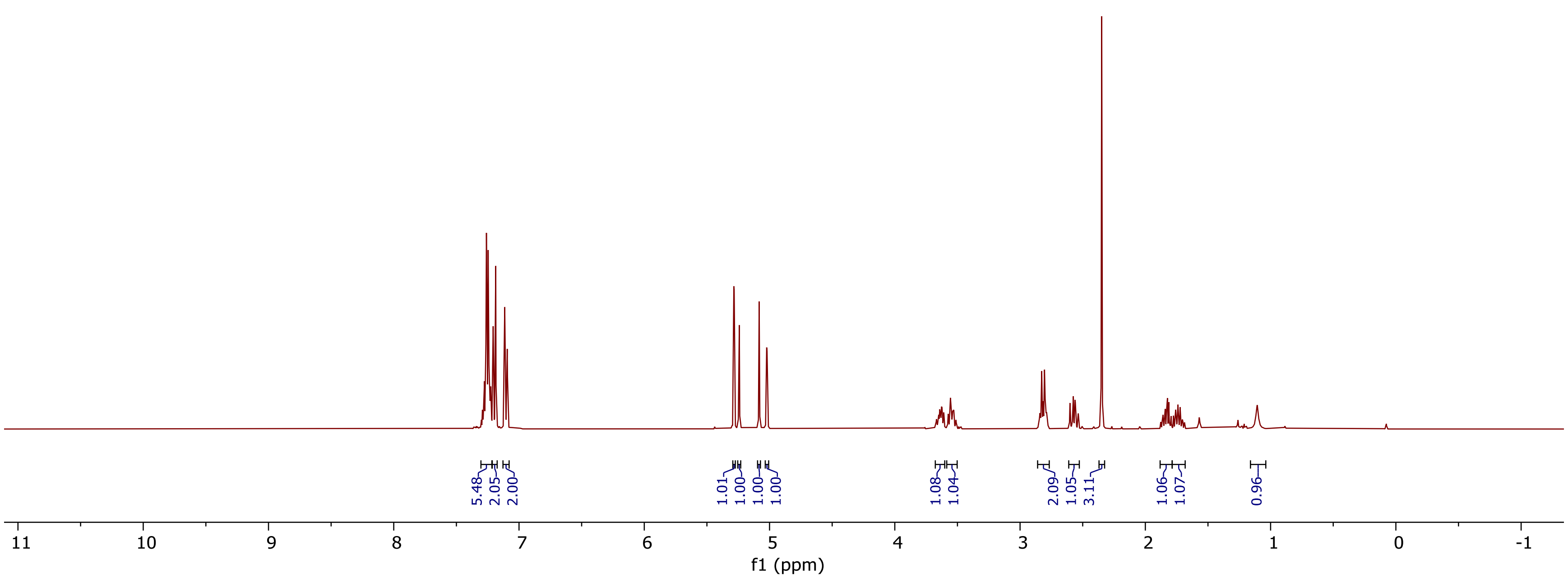

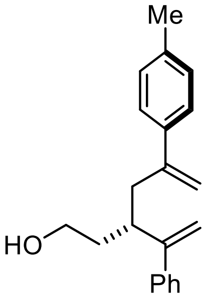

7k

$^{13}C\{^{1}H\}$ NMR (101 MHz, 298 K, $CDCl_3$)

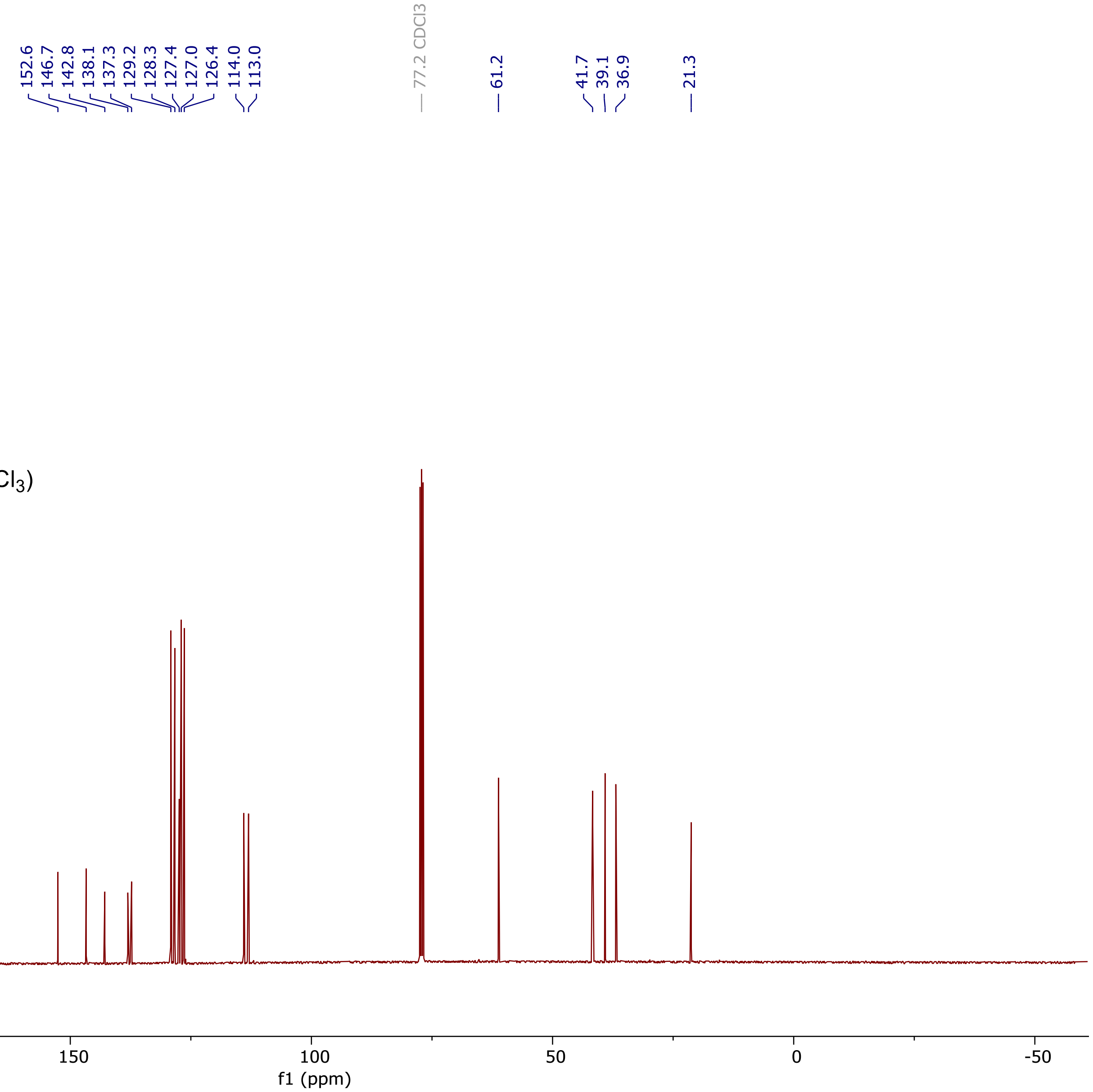

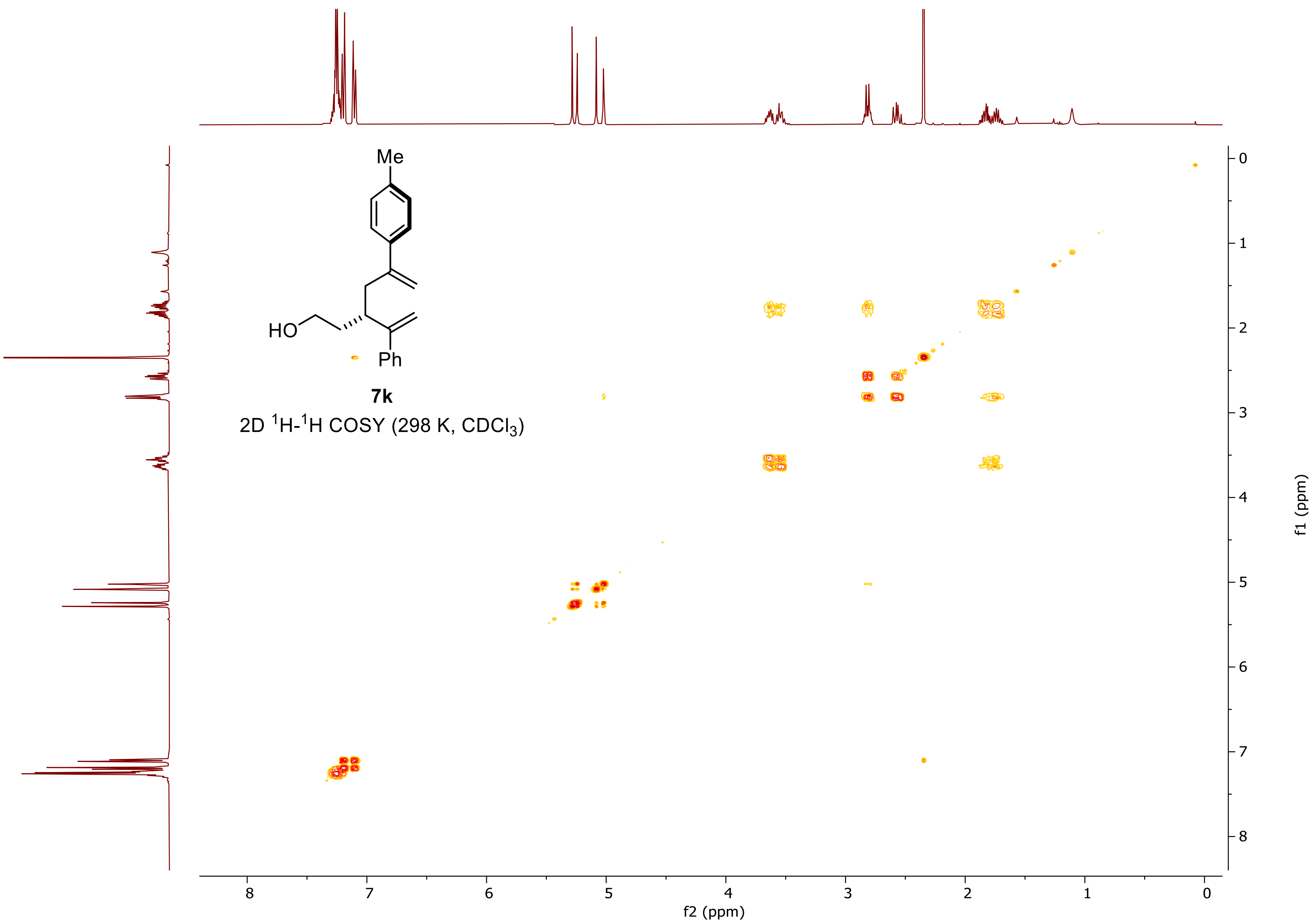
Me
HO
Ph
7k
2D ¹H-¹H COSY (298 K, CDCl3)
f2 (ppm)
f1 (ppm)
8
7
6
5
4
3
2
1
0

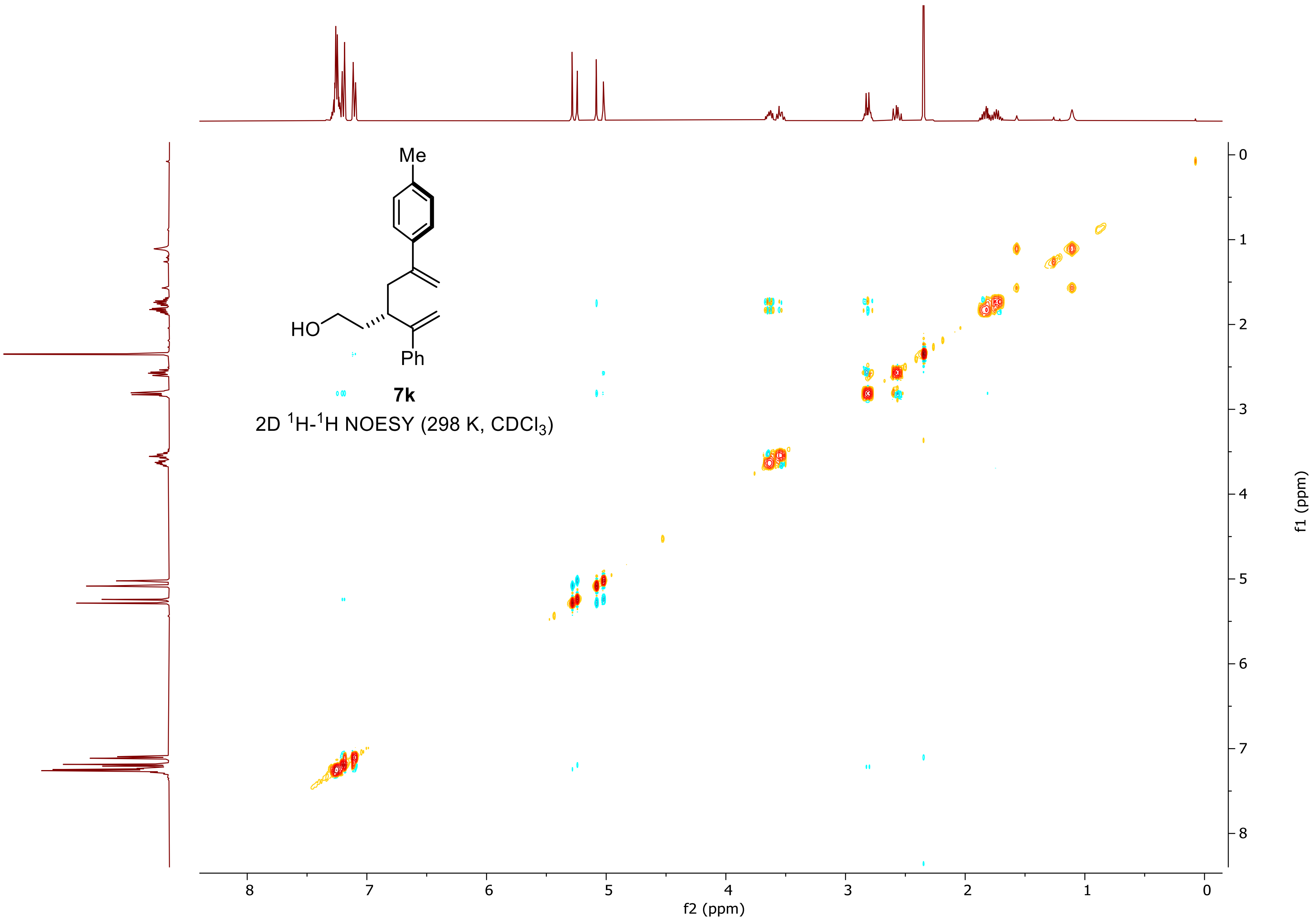
Me
HO
Ph
7k
2D ^{1}H-^{1}H NOESY (298 K, $CDCl_3$)
f2 (ppm)
f1 (ppm)
8
7
6
5
4
3
2
1
0

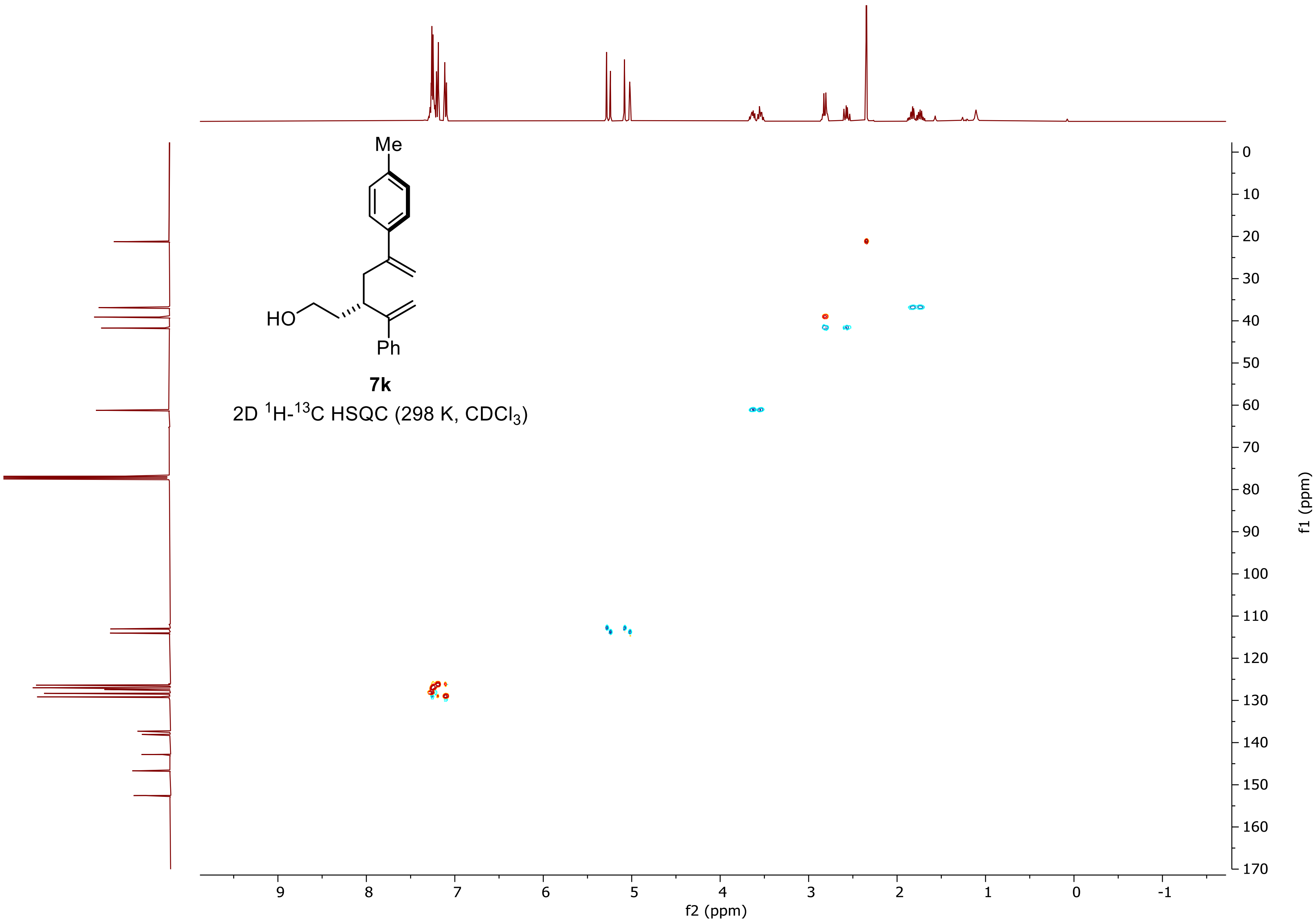

7k

2D ^{1}H-^{13}C HSQC (298 K, $CDCl_3$)

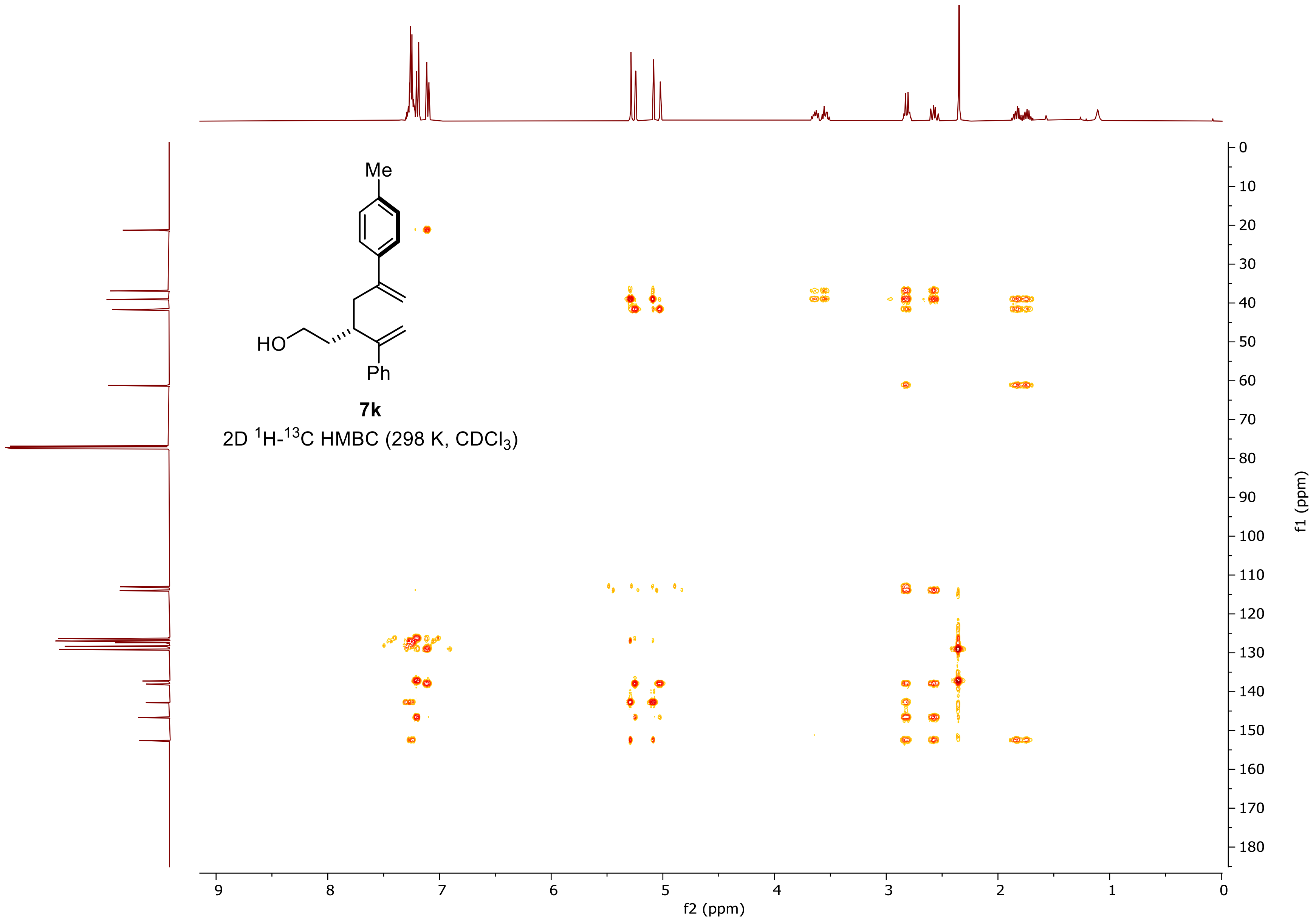

7k

2D ^{1}H-^{13}C HMBC (298 K, $CDCl_3$)

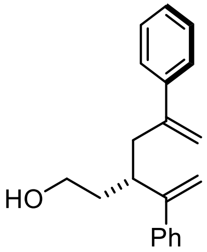

7l

^{1}H NMR (400 MHz, 298 K, CDCl$_3$)

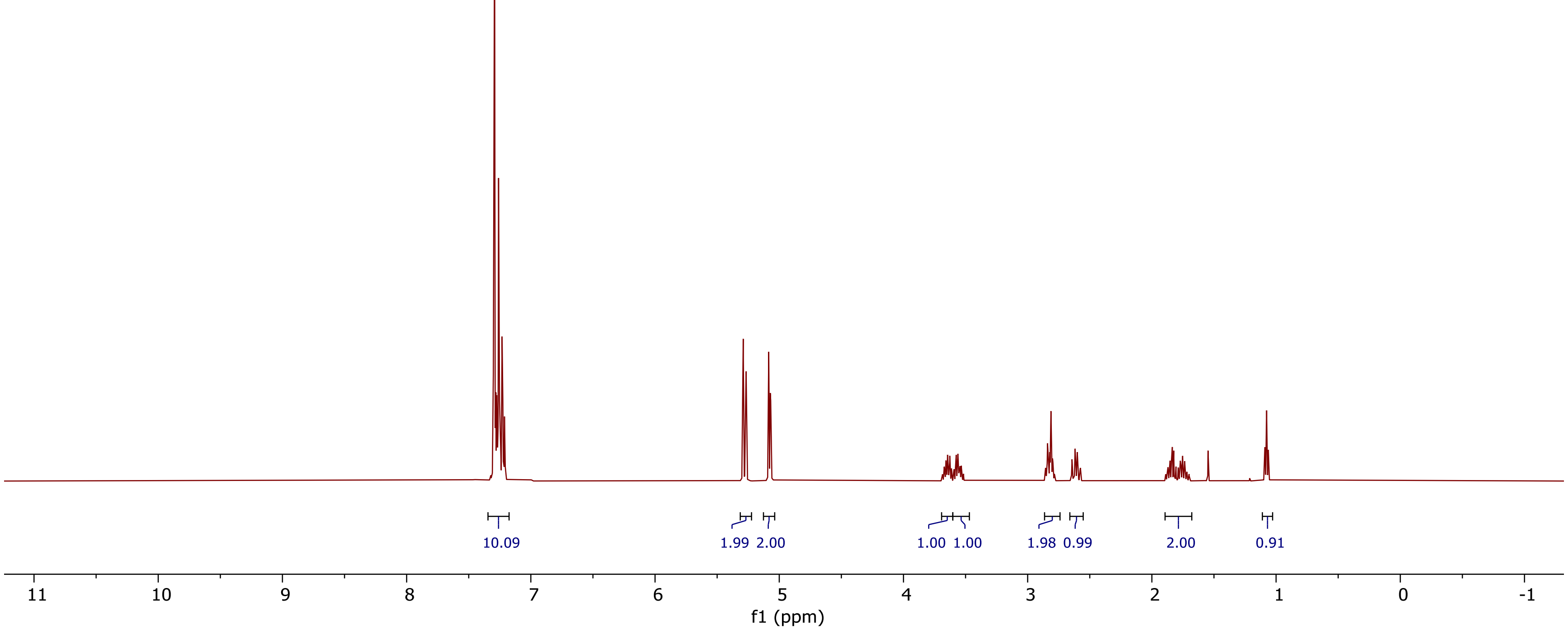

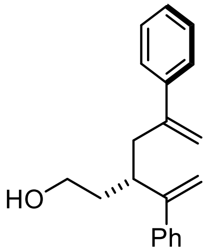

7l

$^{13}C\{^1H\}$ NMR (101 MHz, 298 K, $CDCl_3$)

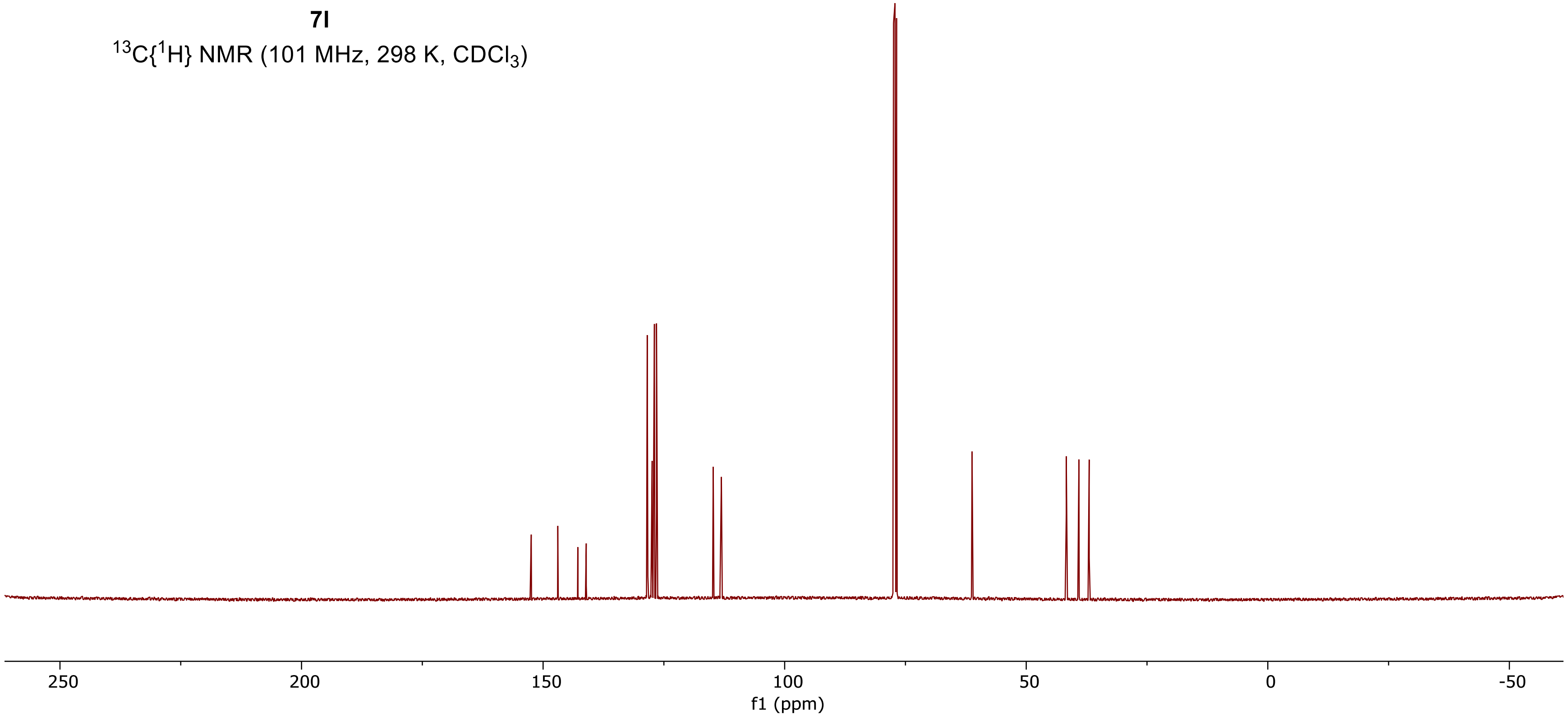

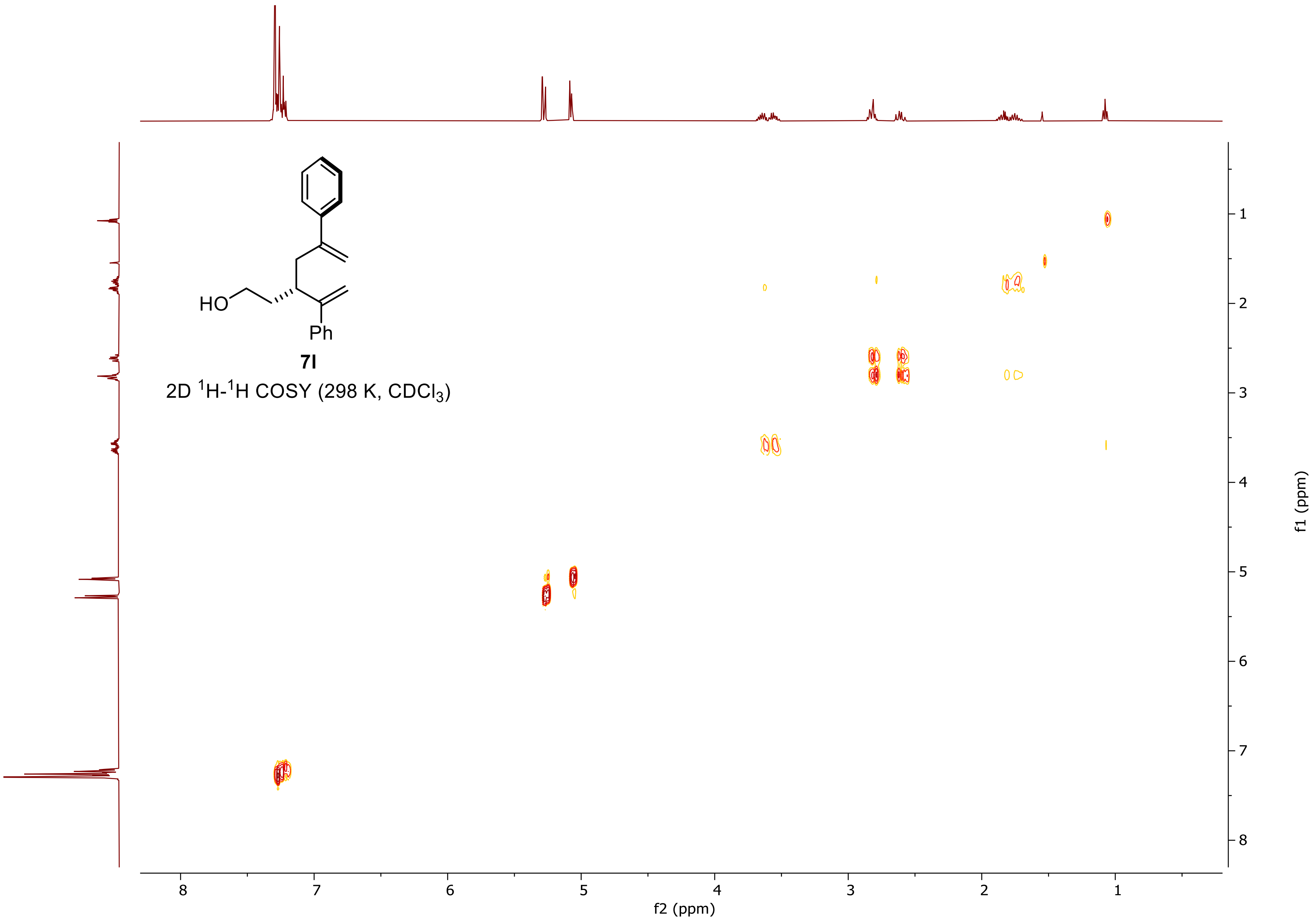
HO
Ph
7l
2D 1H-1H COSY (298 K, CDCl3)
8 7 6 5 4 3 2 1
f2 (ppm)
1 2 3 4 5 6 7 8
f1 (ppm)

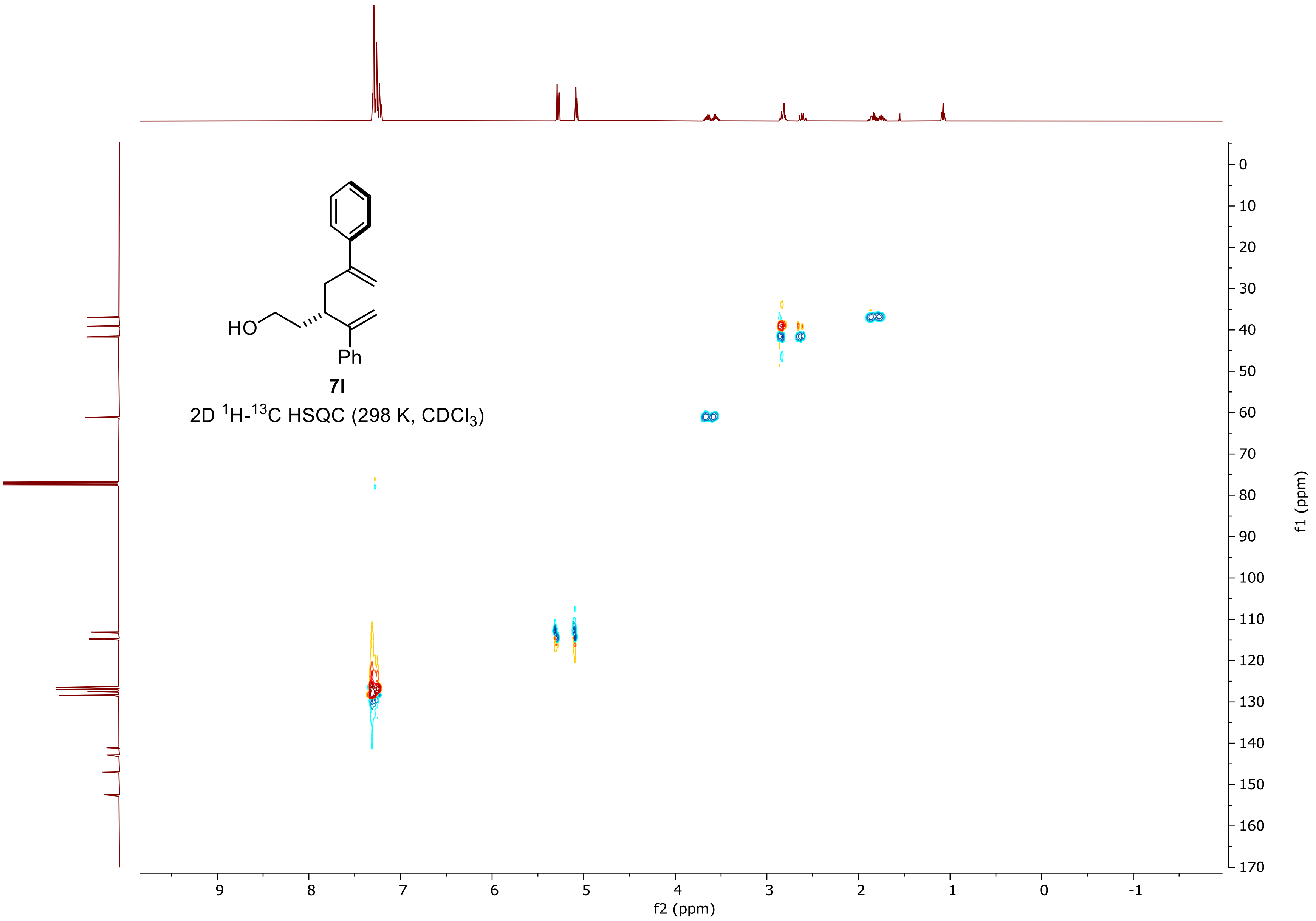
HO
Ph
7l
2D ¹H-¹³C HSQC (298 K, CDCl₃)
f2 (ppm)
f1 (ppm)

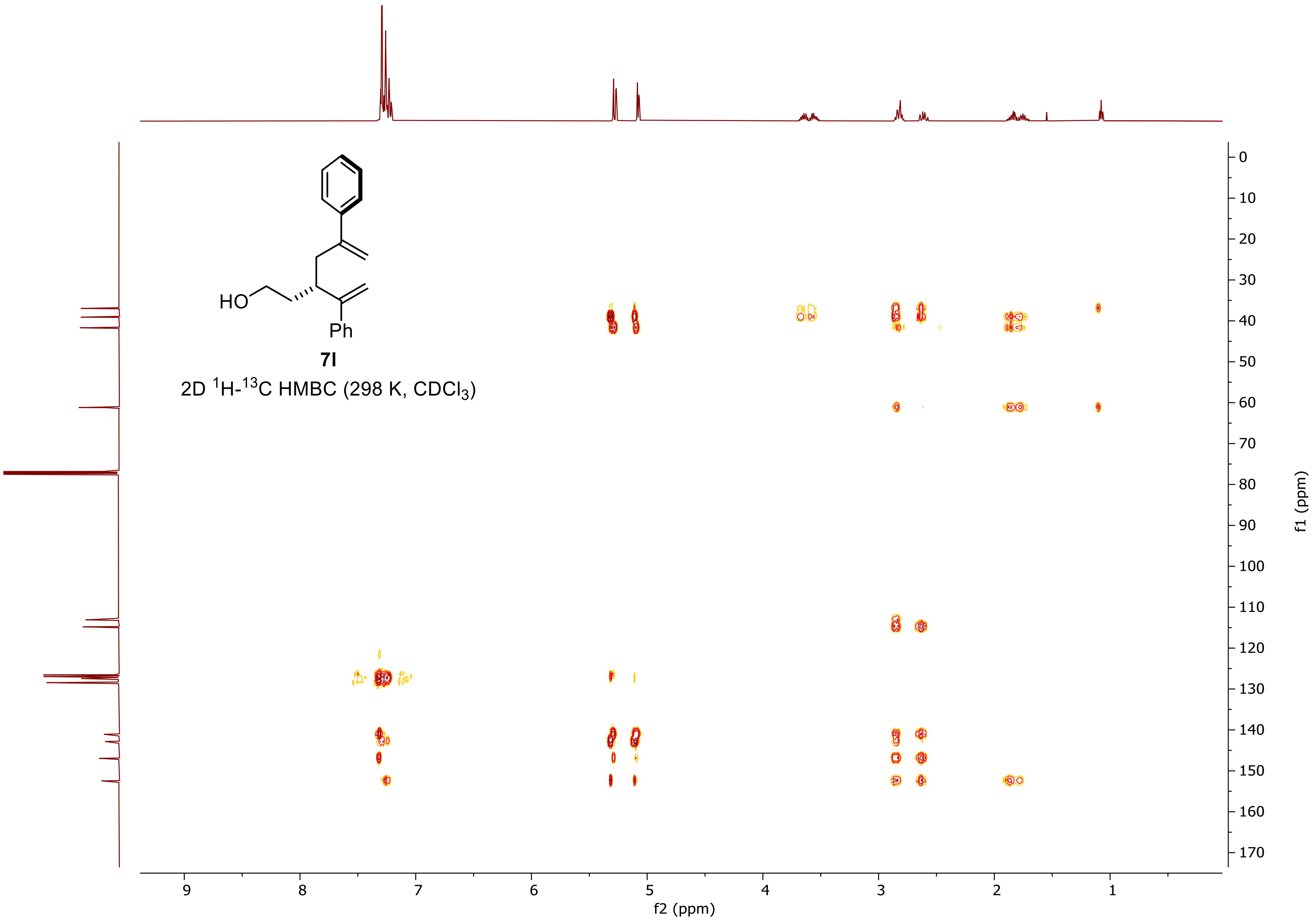

HO
Ph
7l
2D ¹H-¹³C HMBC (298 K, CDCl₃)
f1 (ppm)
f2 (ppm)

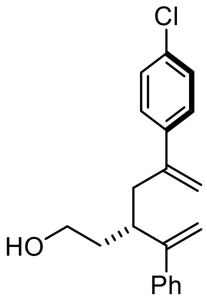

7m

^{1}H NMR (400 MHz, 298 K, $CDCl_3$)

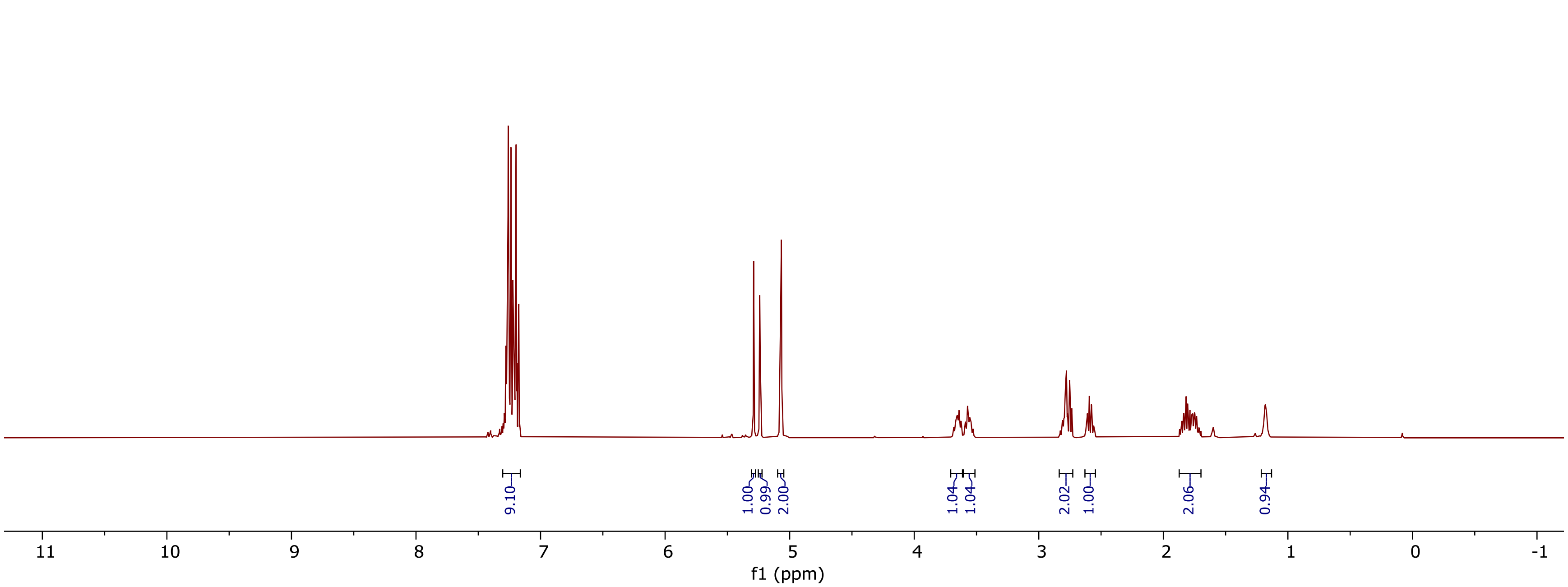

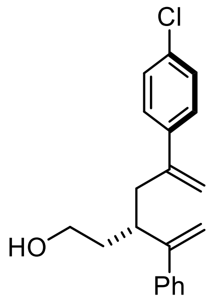

7m

$^{13}C\{^1H\}$ NMR (101 MHz, 298 K, $CDCl_3$)

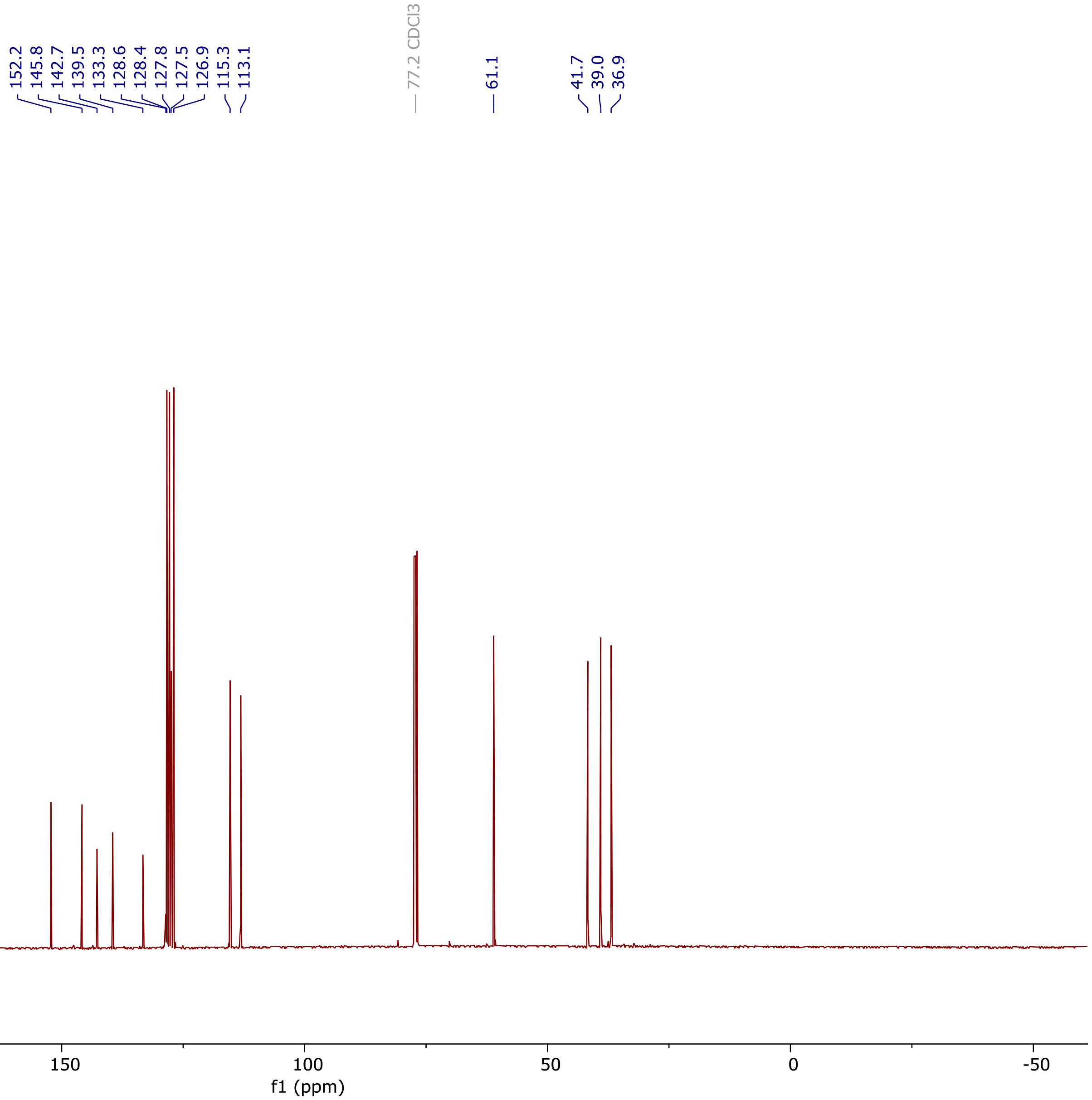

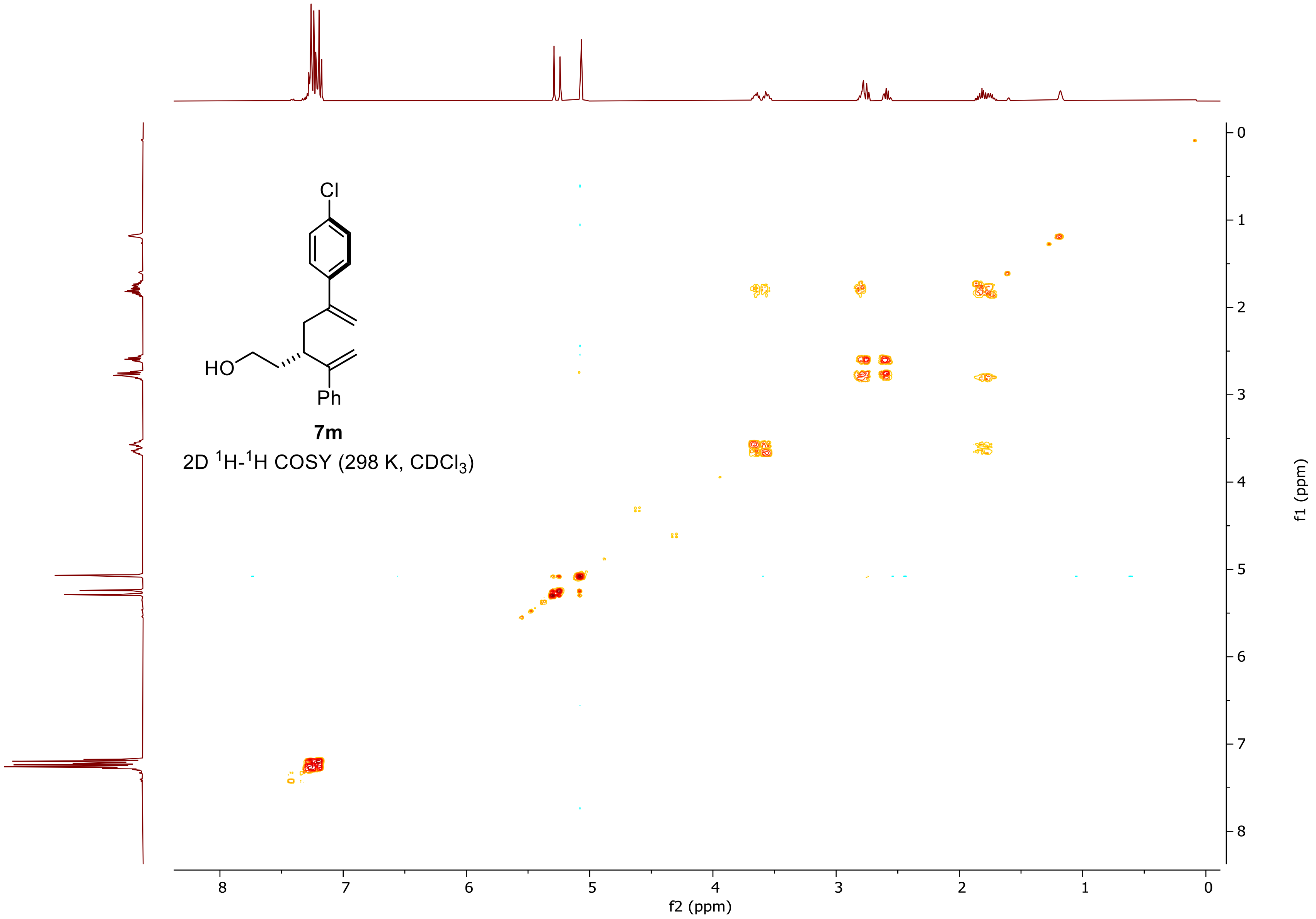

7m

2D ^{1}H-^{1}H COSY (298 K, $CDCl_3$)

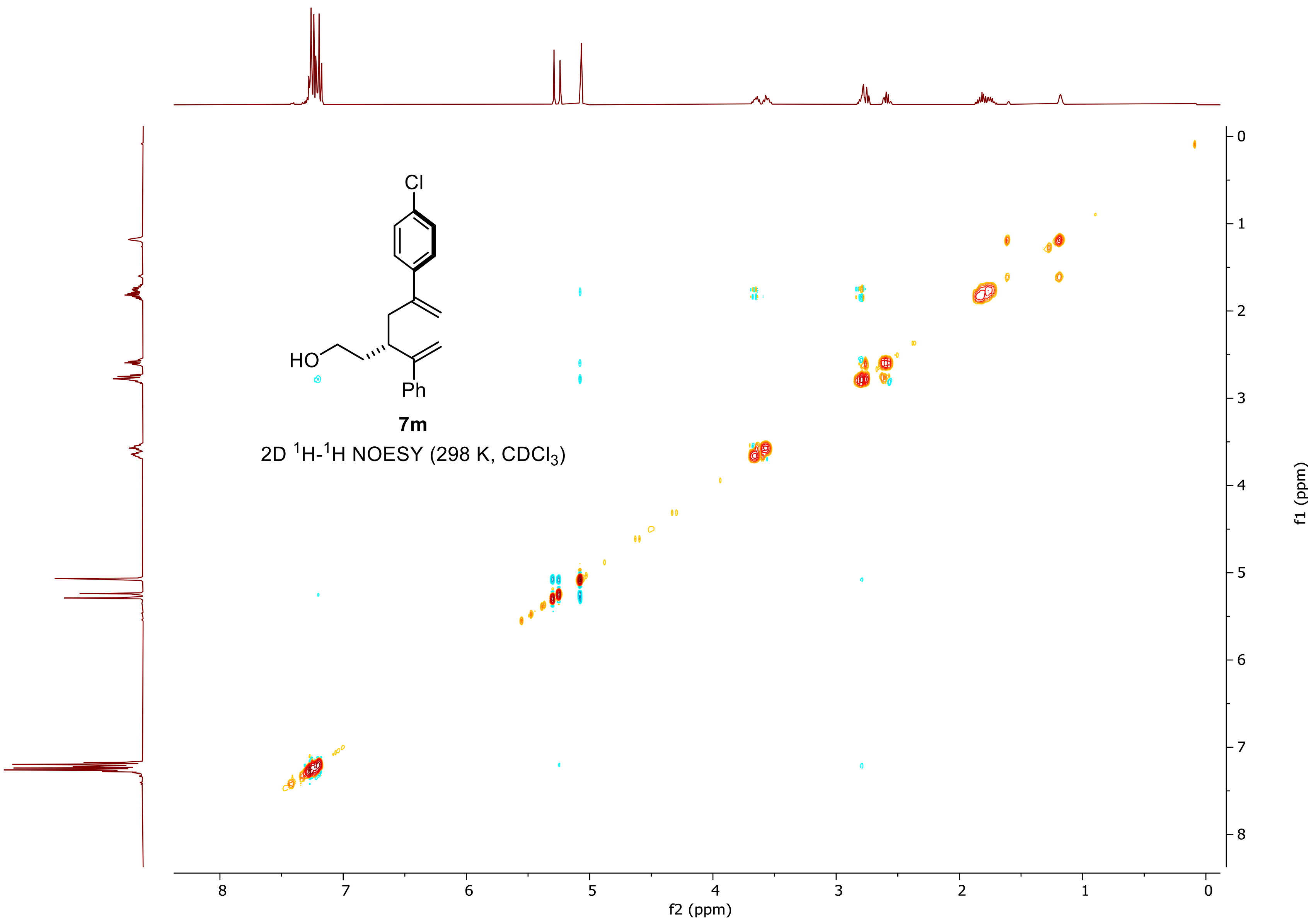

7m

2D ^{1}H-^{1}H NOESY (298 K, $CDCl_3$)

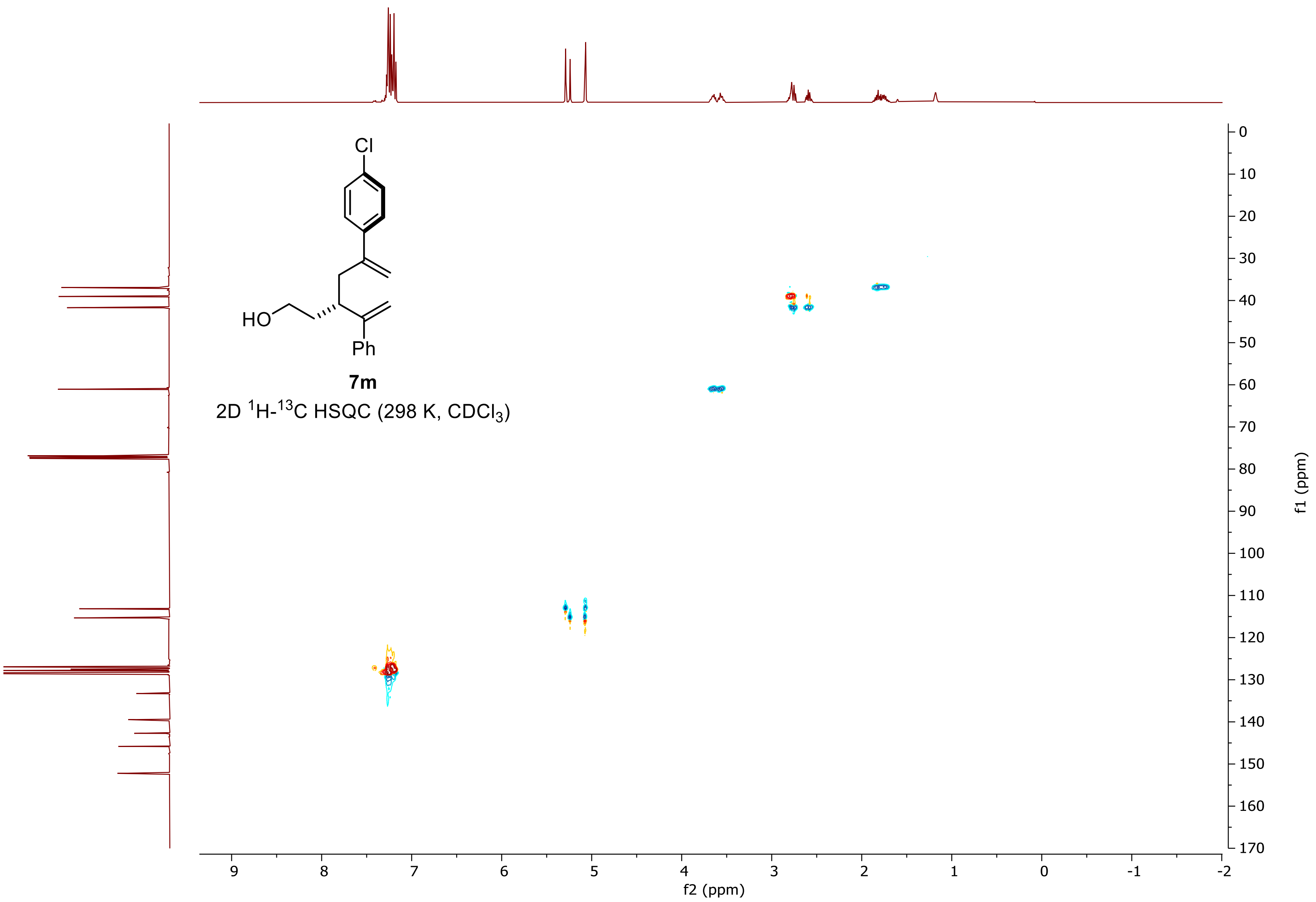

Cl
HO
Ph
7m
2D ¹H-¹³C HSQC (298 K, CDCl₃)
f1 (ppm)
f2 (ppm)

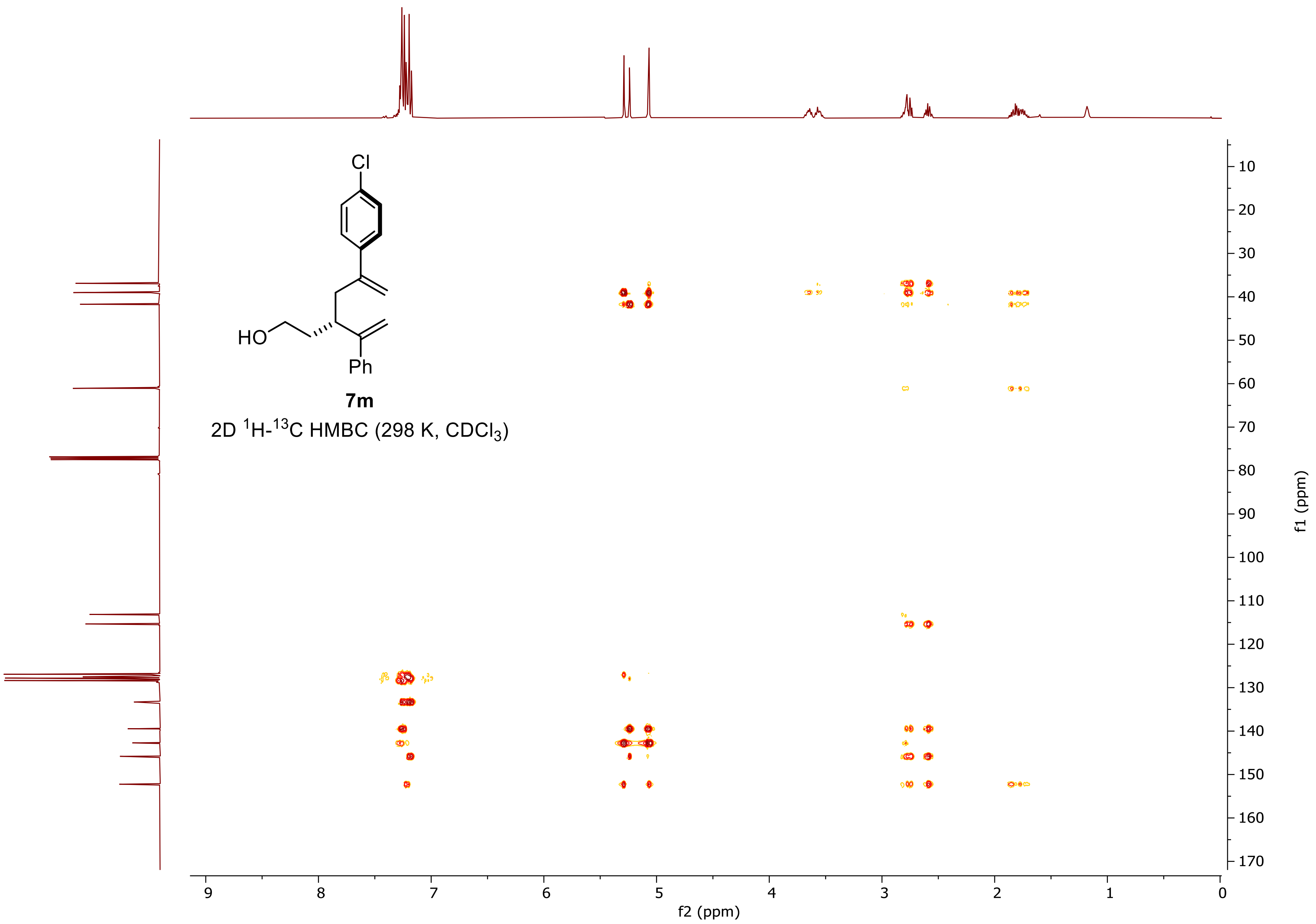
Cl
HO
Ph
7m
2D ¹H-¹³C HMBC (298 K, CDCl3)
f2 (ppm)
f1 (ppm)

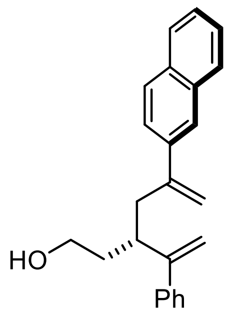

7n

^1H NMR (400 MHz, 298 K, $CDCl_3$)

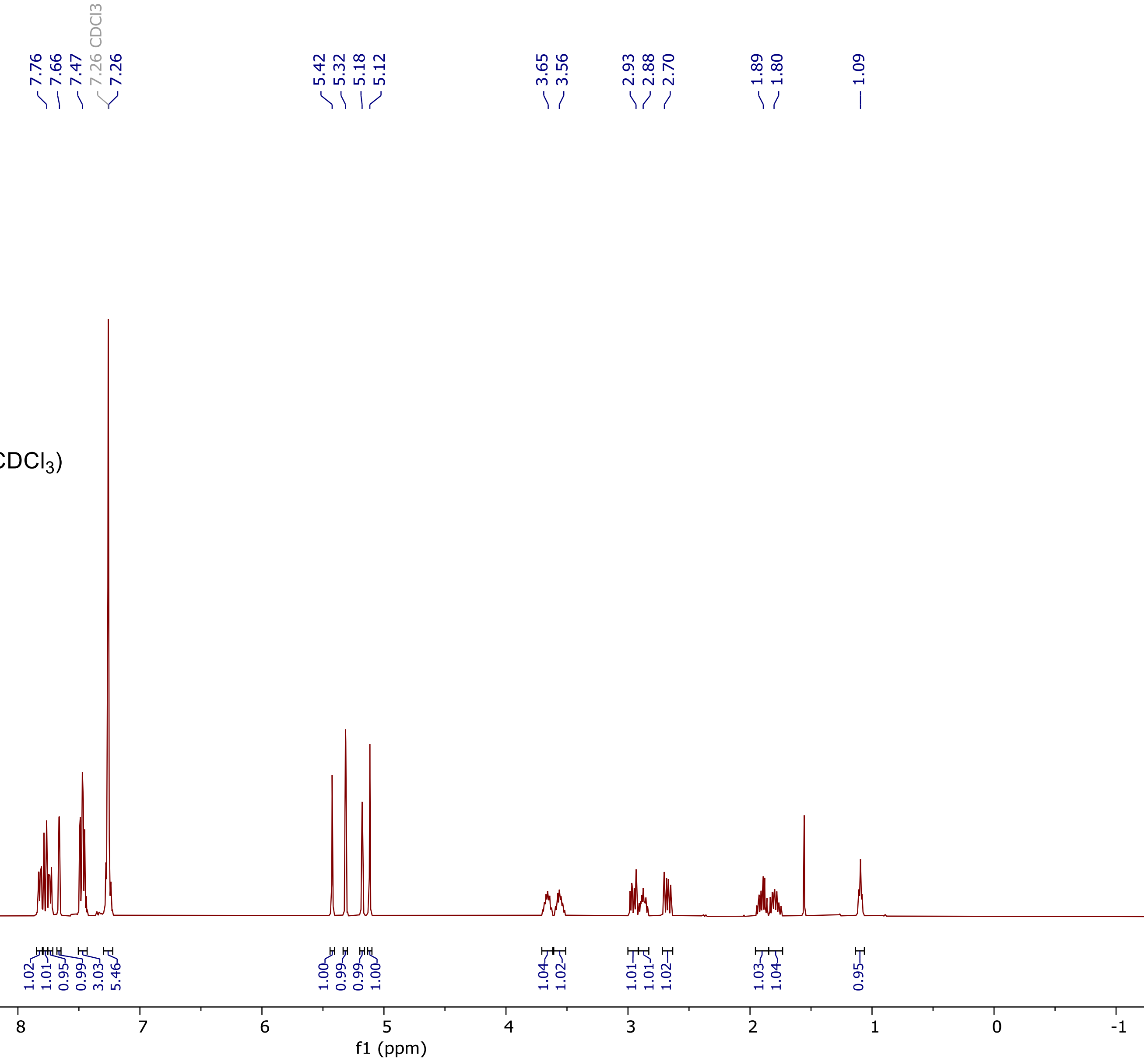

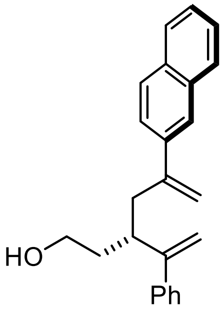

7n

$^{13}C\{^{1}H\}$ NMR (101 MHz, 298 K, $CDCl_3$)

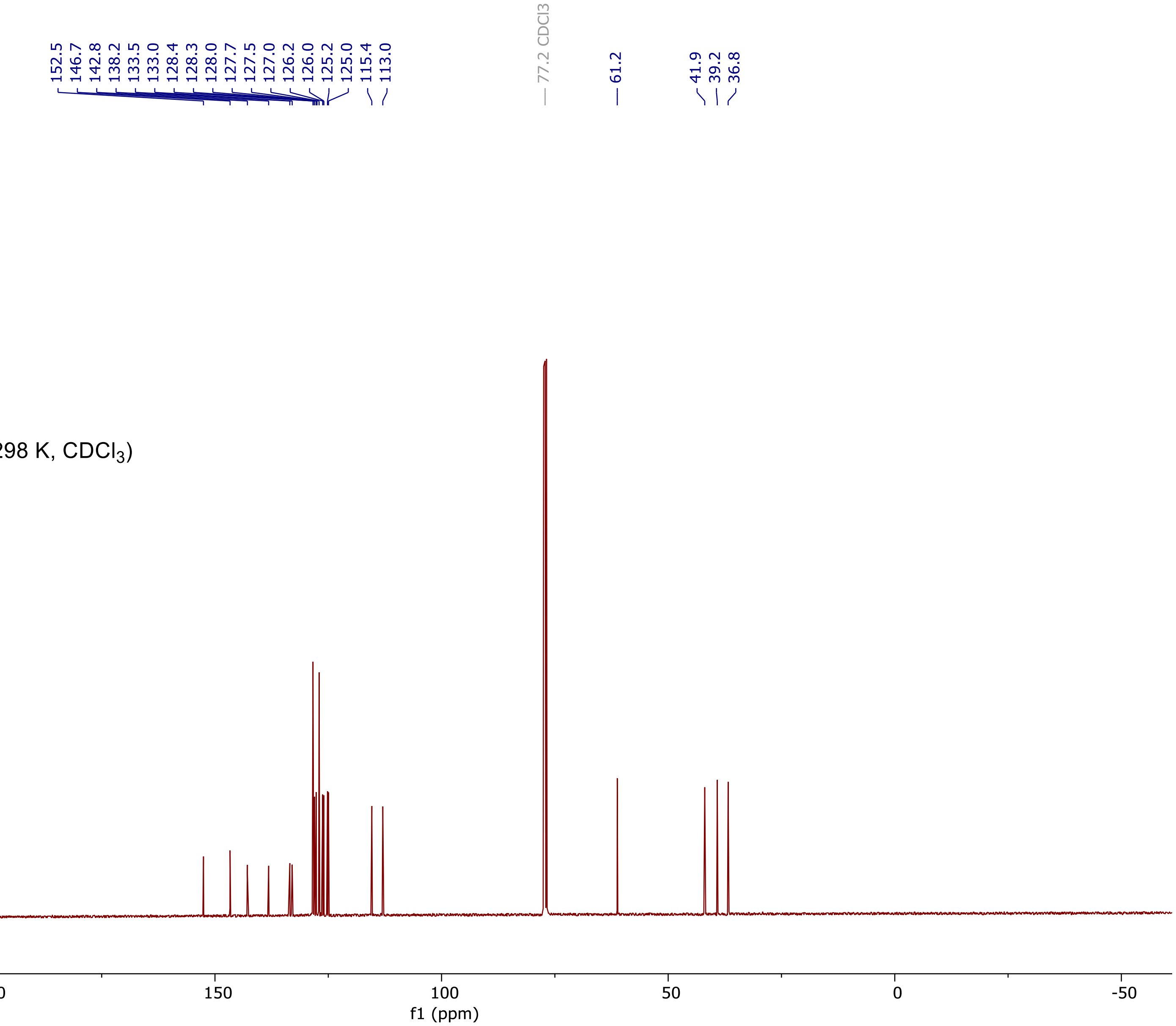

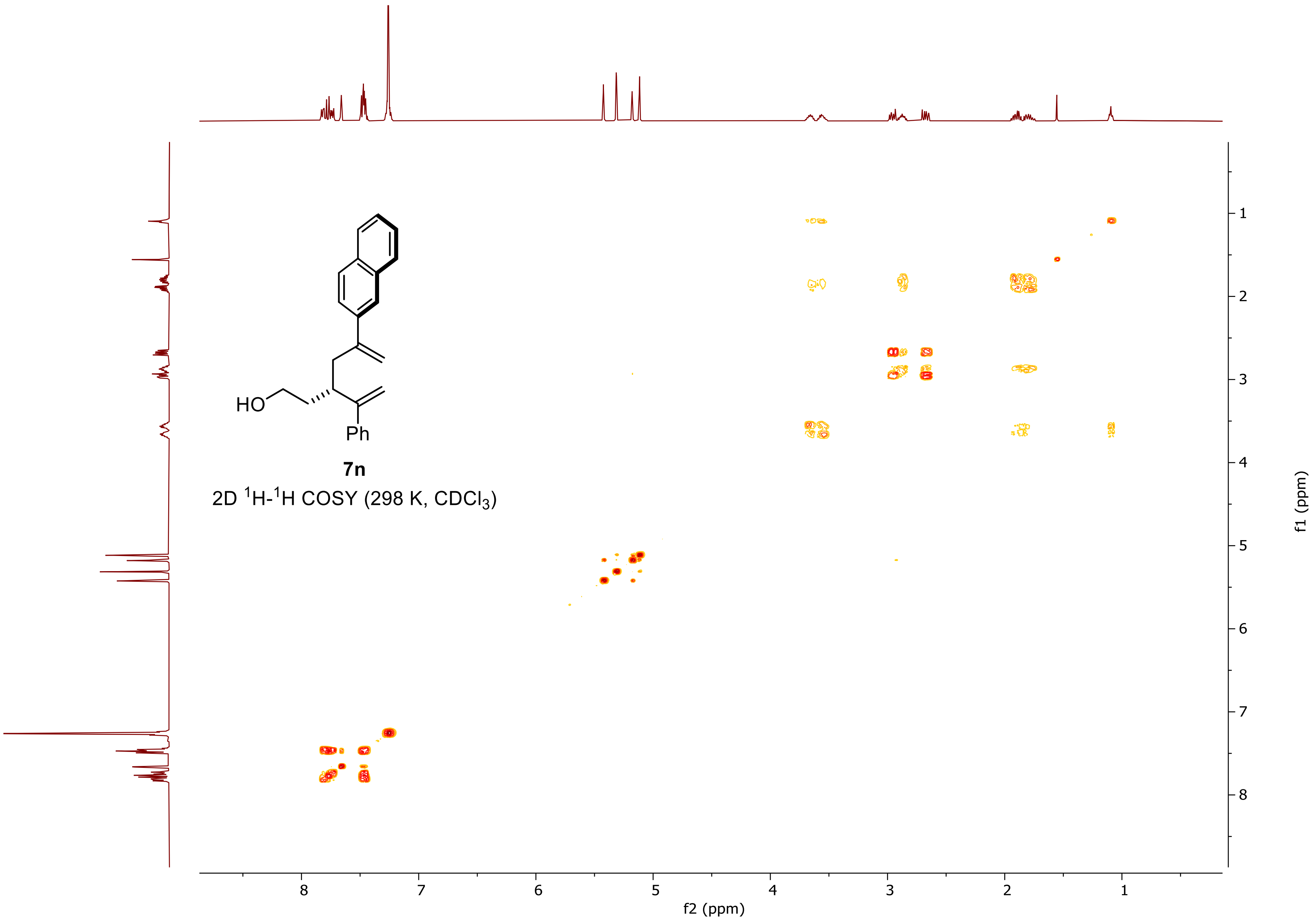

7n

2D ^{1}H-^{1}H COSY (298 K, $CDCl_3$)

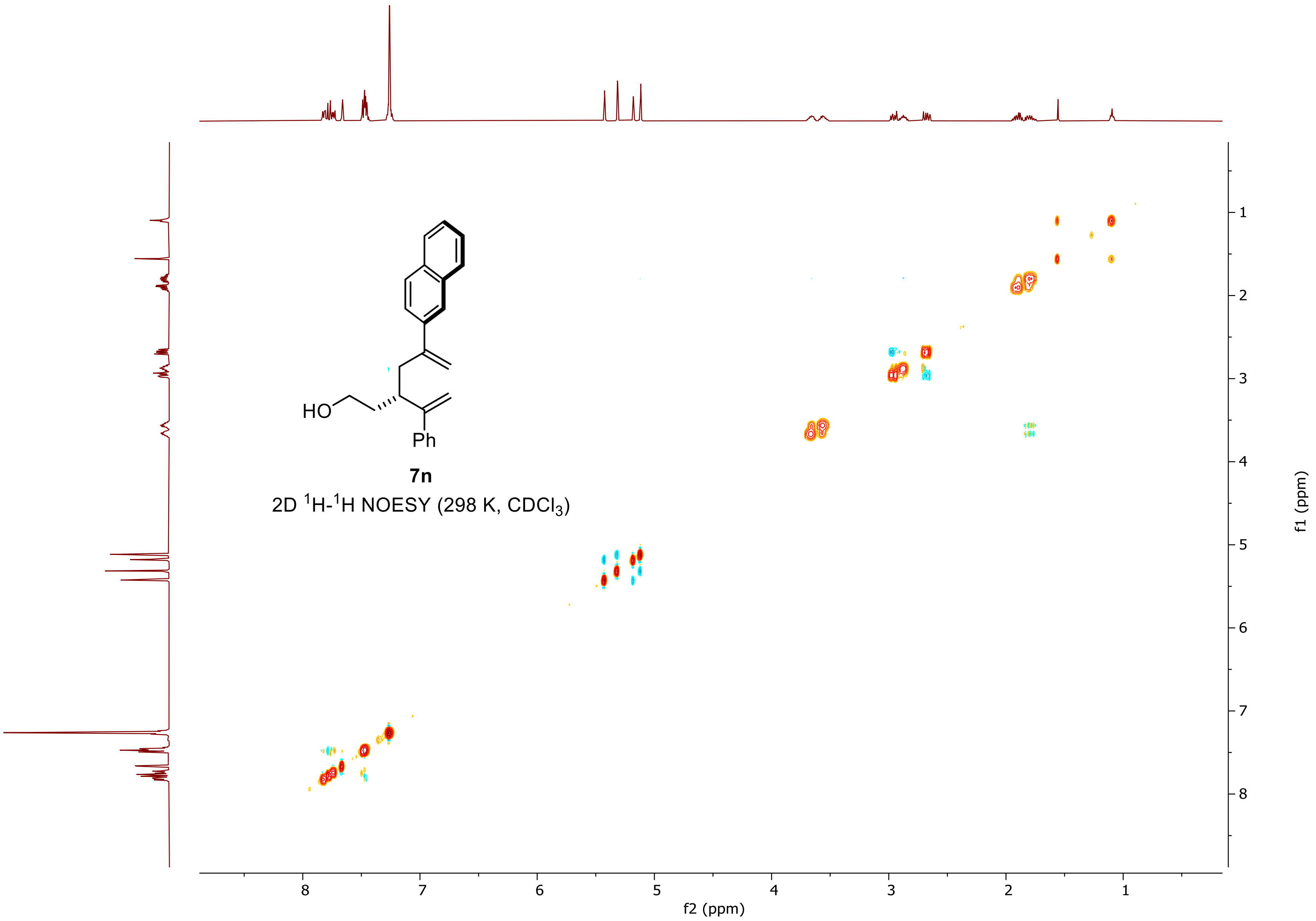

7n

2D ^{1}H-^{1}H NOESY (298 K, $CDCl_3$)

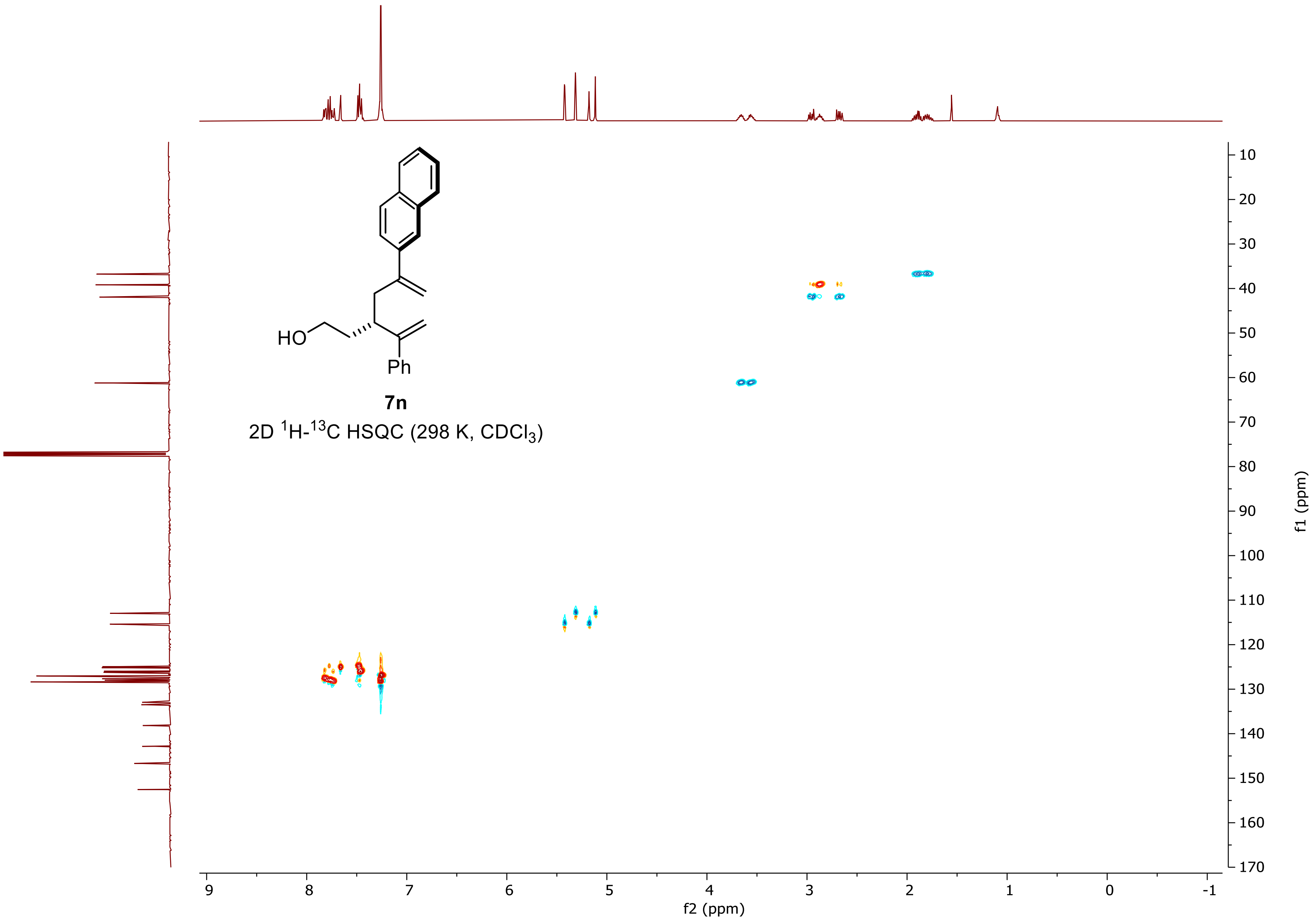

7n

2D ^{1}H-^{13}C HSQC (298 K, $CDCl_3$)

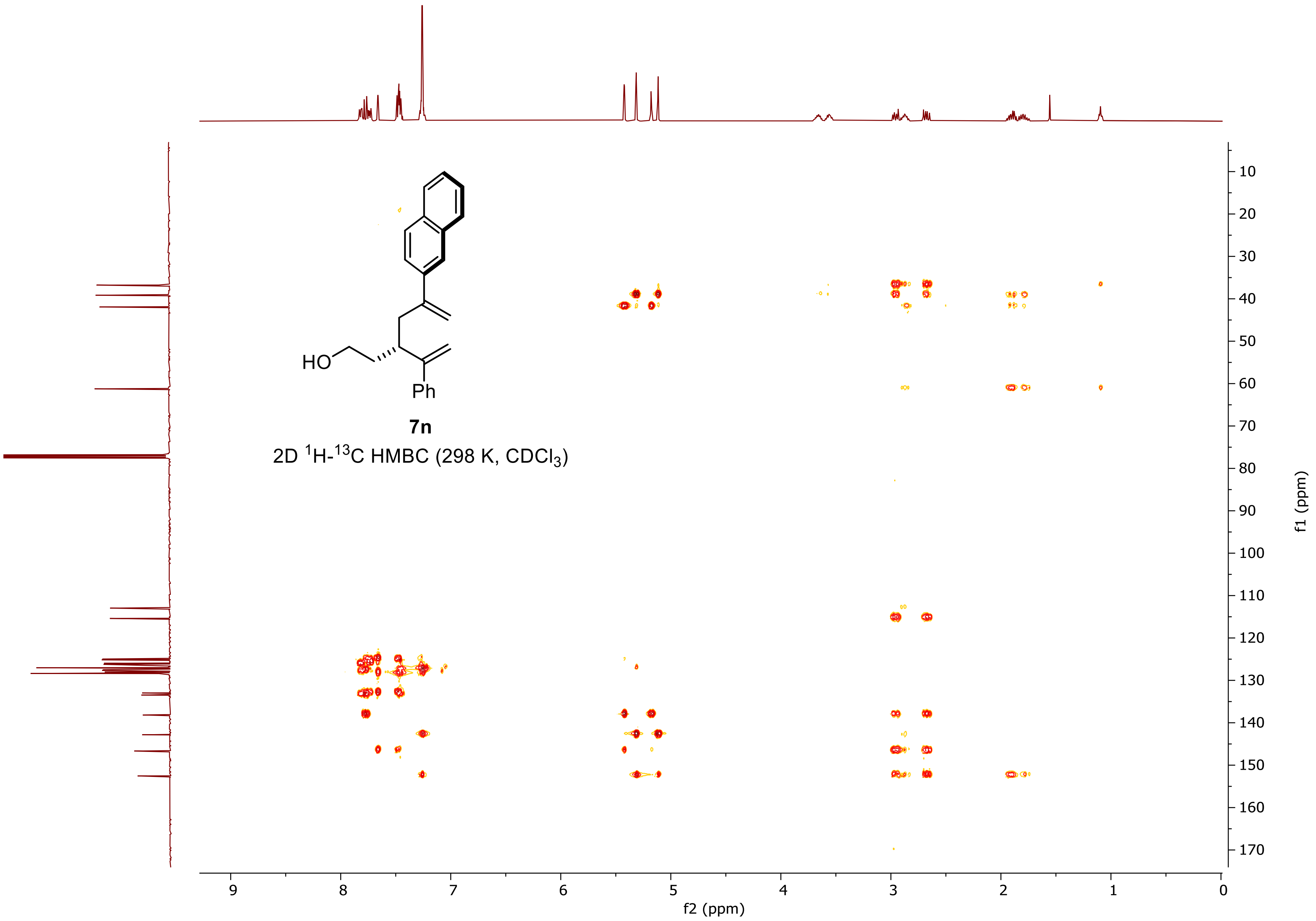
HO
Ph
7n
2D ¹H-¹³C HMBC (298 K, CDCl3)
f2 (ppm)
f1 (ppm)

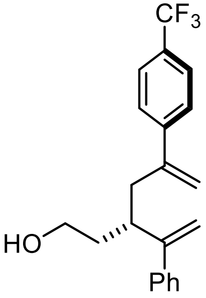

7o

^{1}H NMR (400 MHz, 298 K, $CDCl_3$)

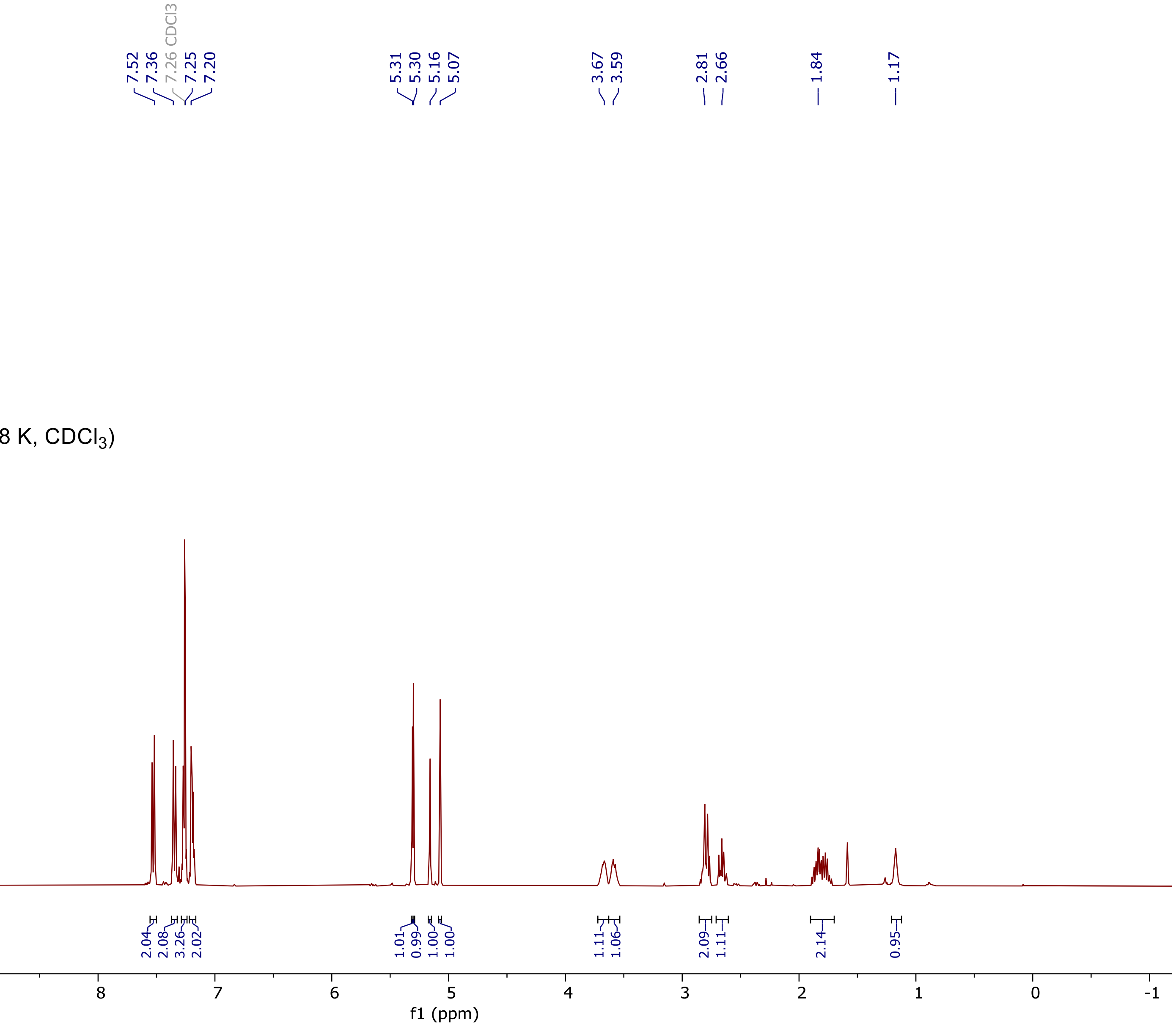

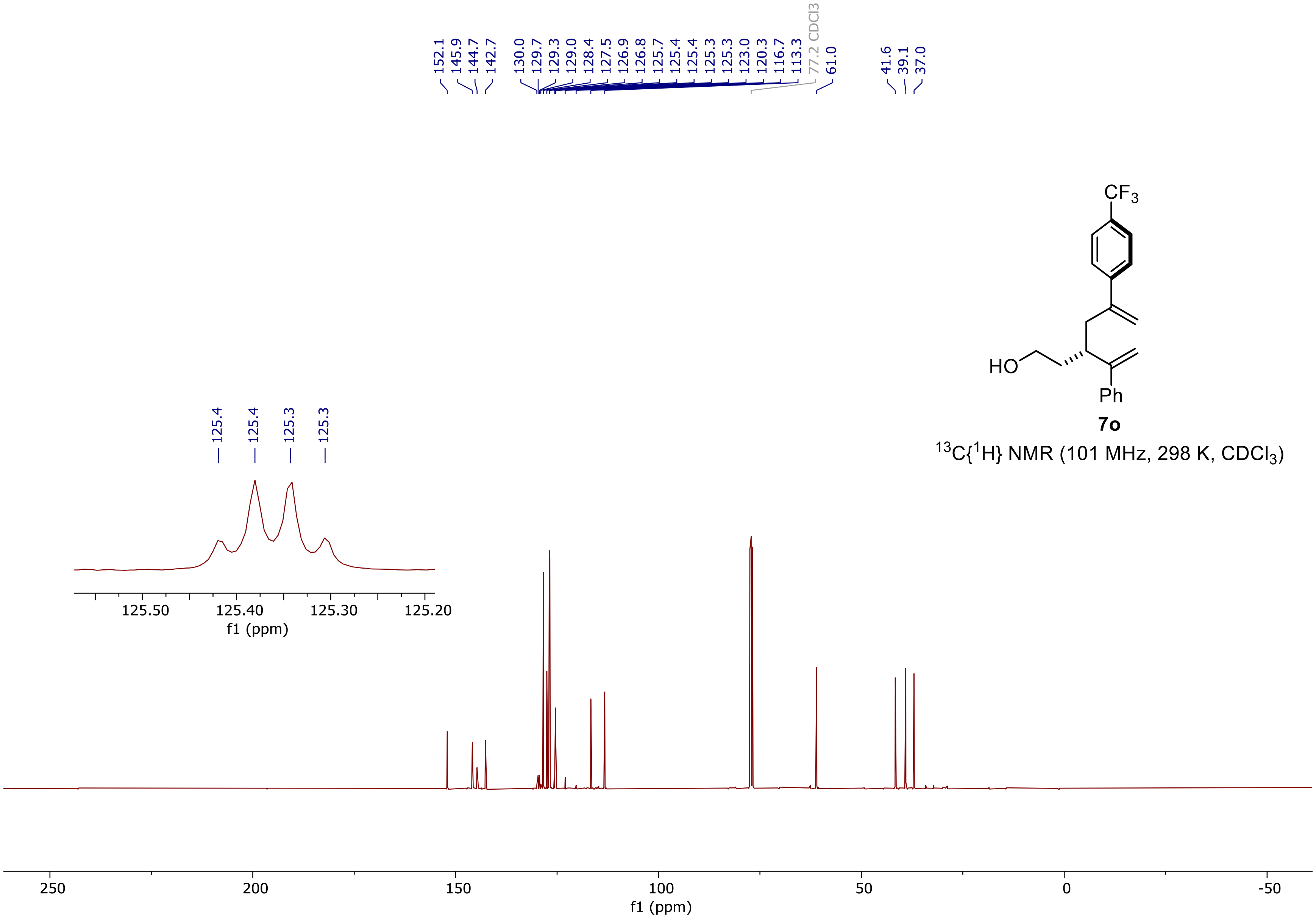

7o

$^{13}C\{^{1}H\}$ NMR (101 MHz, 298 K, $CDCl_3$)

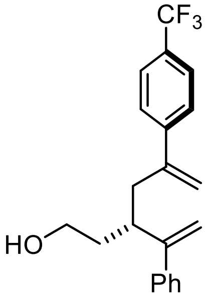

7o

$^{19}F\{^{1}H\}$ NMR (282 MHz, 298 K, $CDCl_3$)

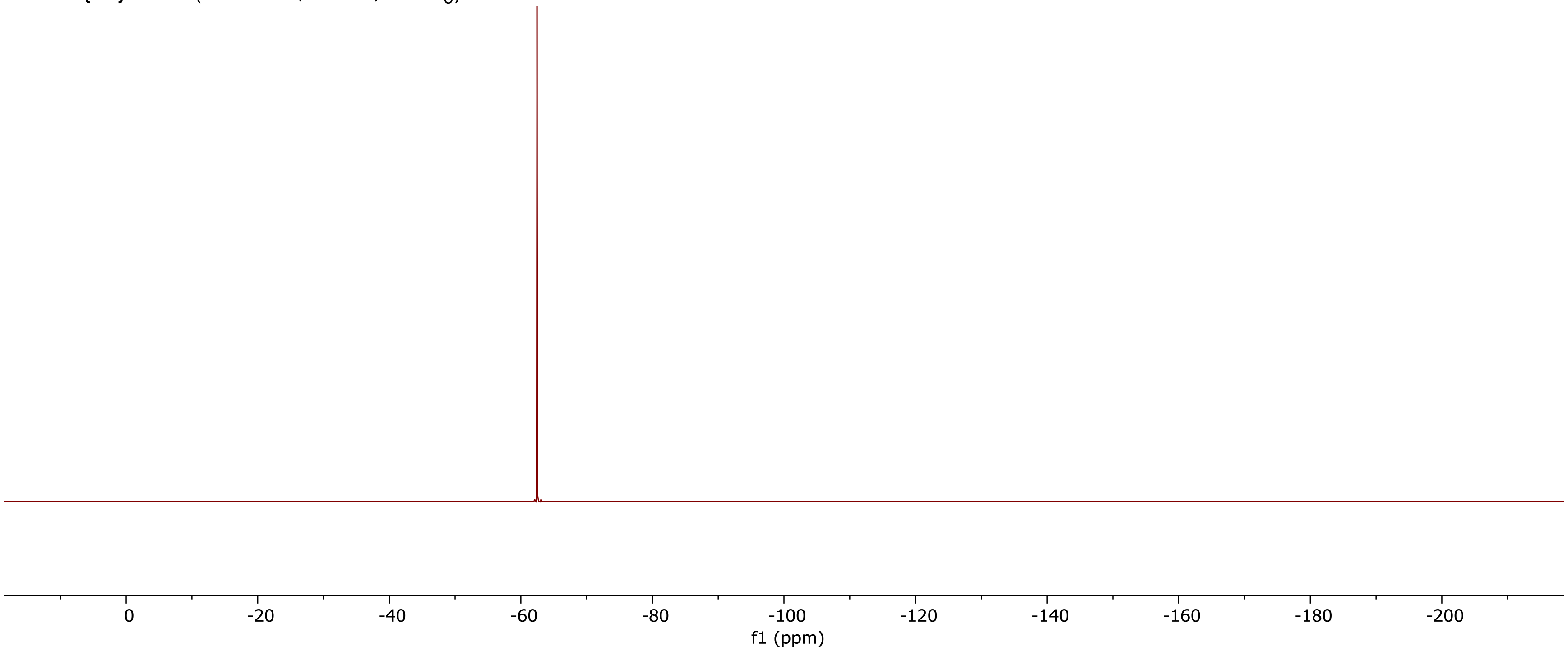

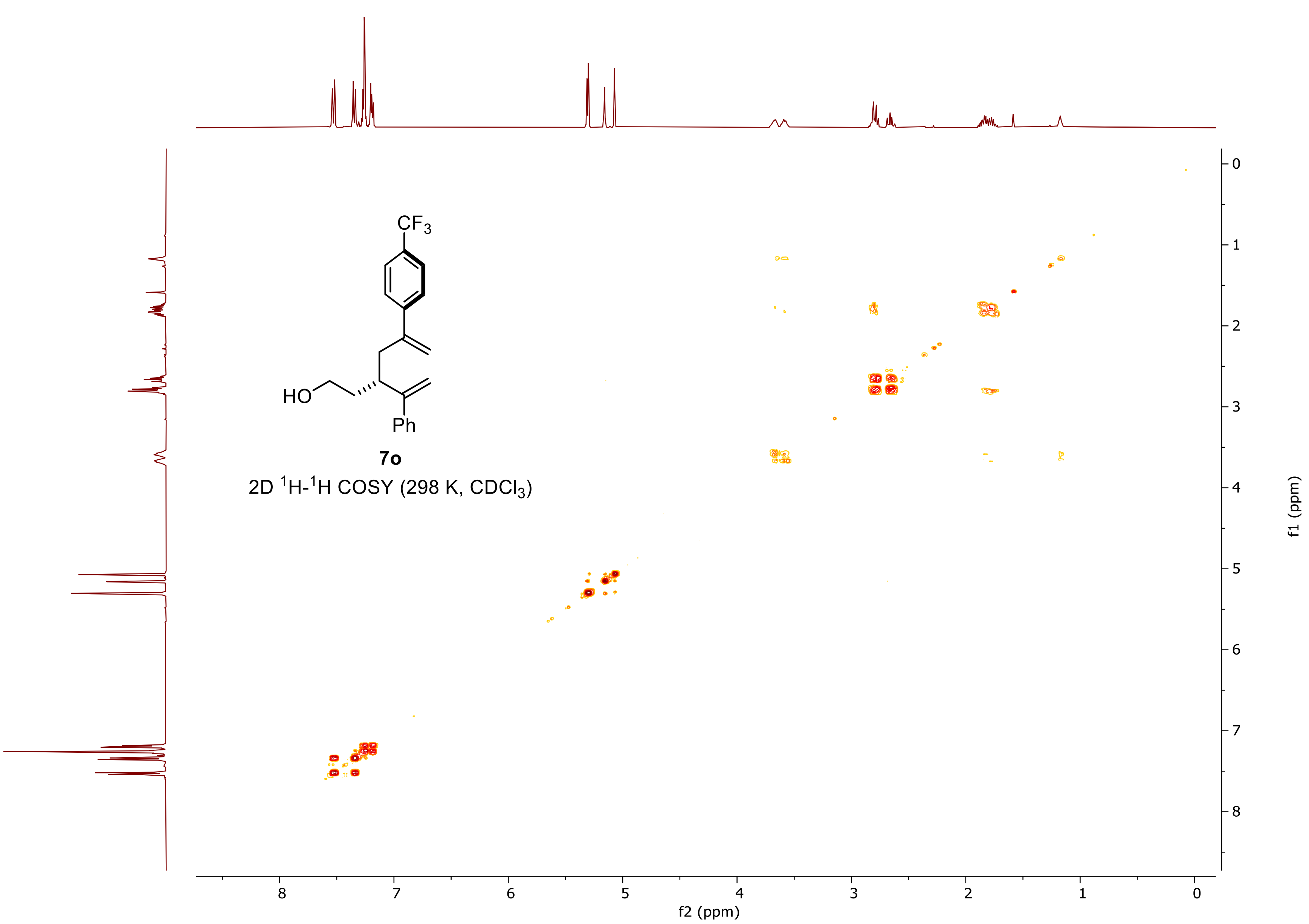

7o

2D ^{1}H-^{1}H COSY (298 K, $CDCl_3$)

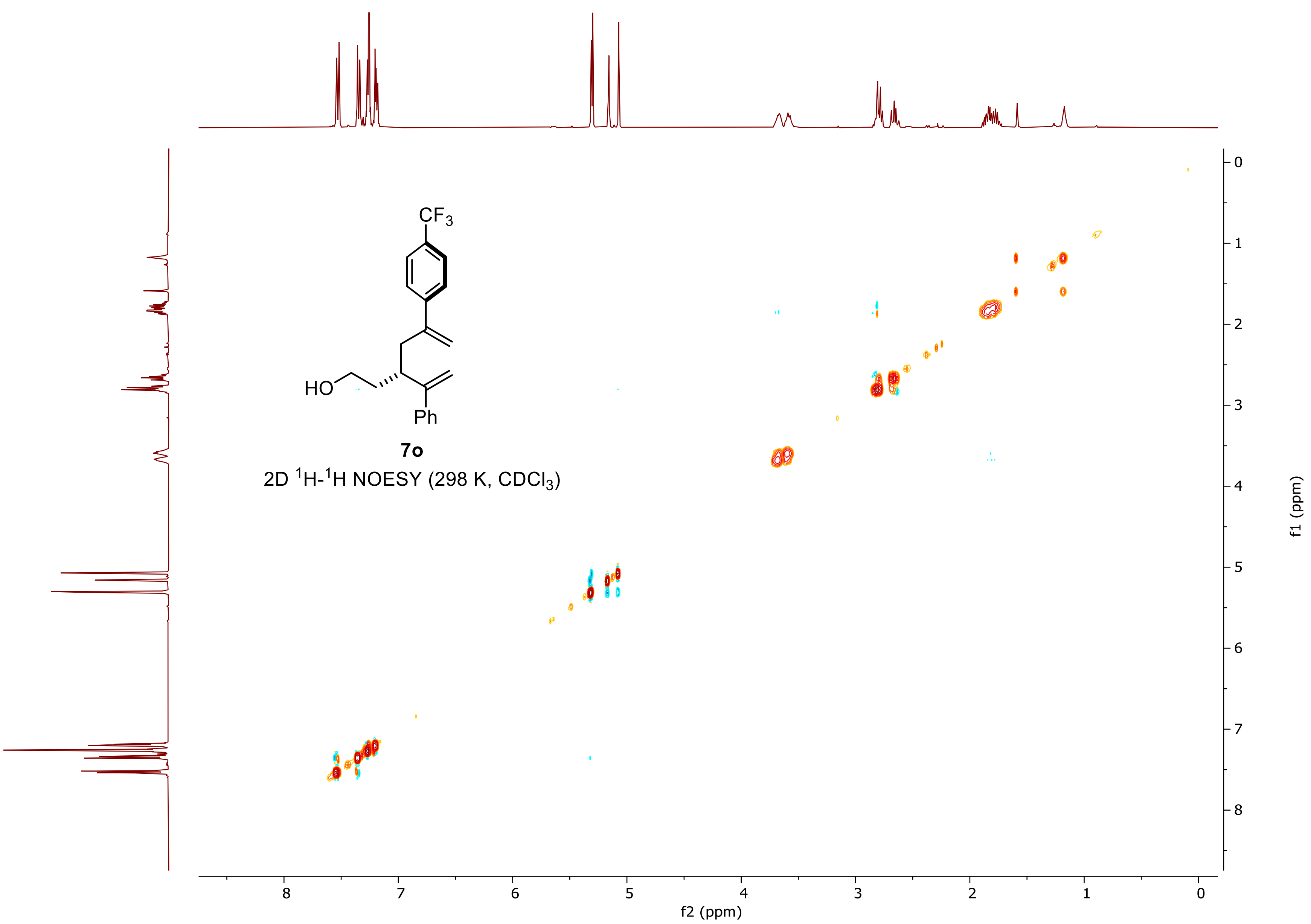

7o

2D ^1H-^1H NOESY (298 K, $CDCl_3$)

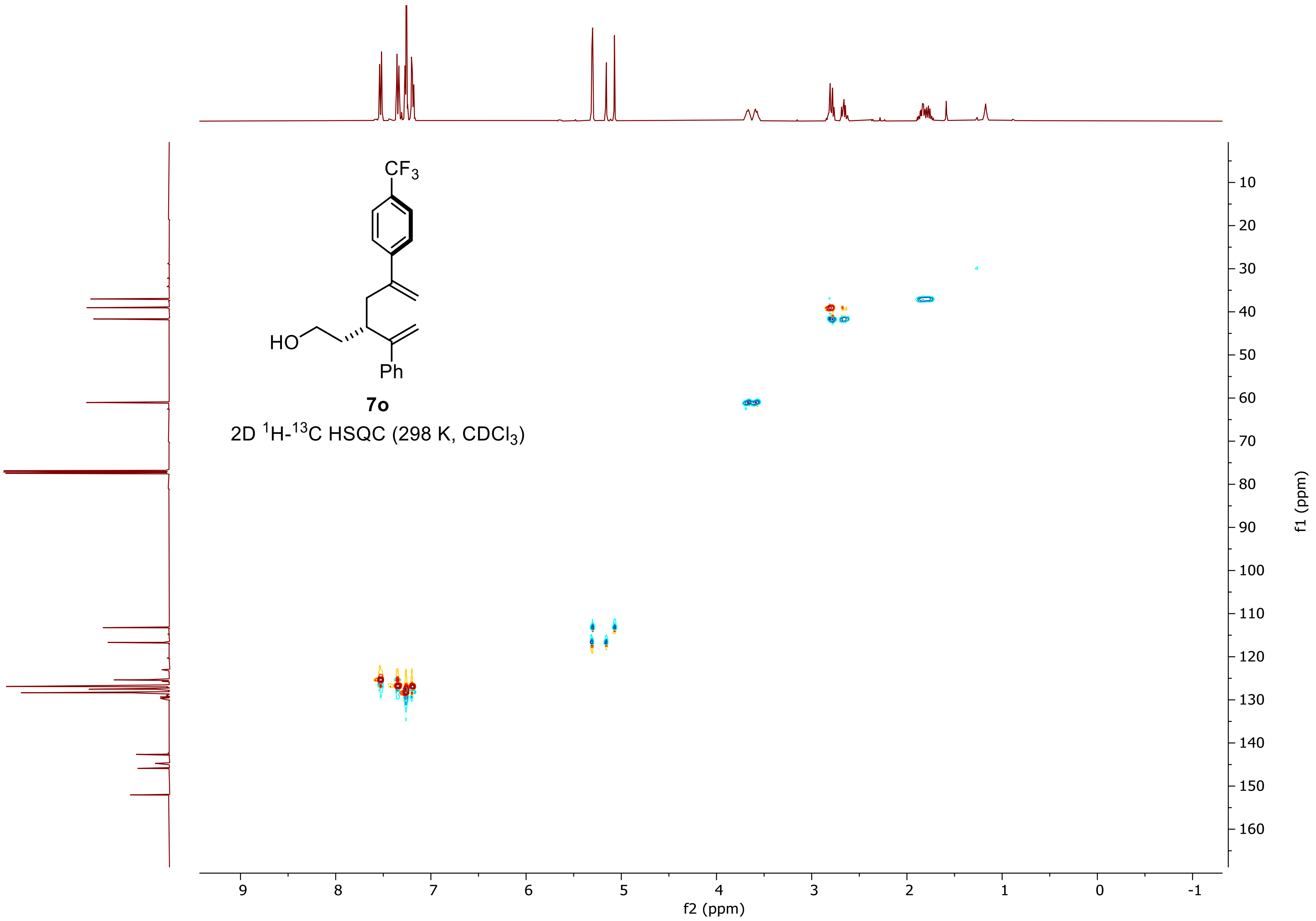

7o

2D ^{1}H-^{13}C HSQC (298 K, $CDCl_3$)

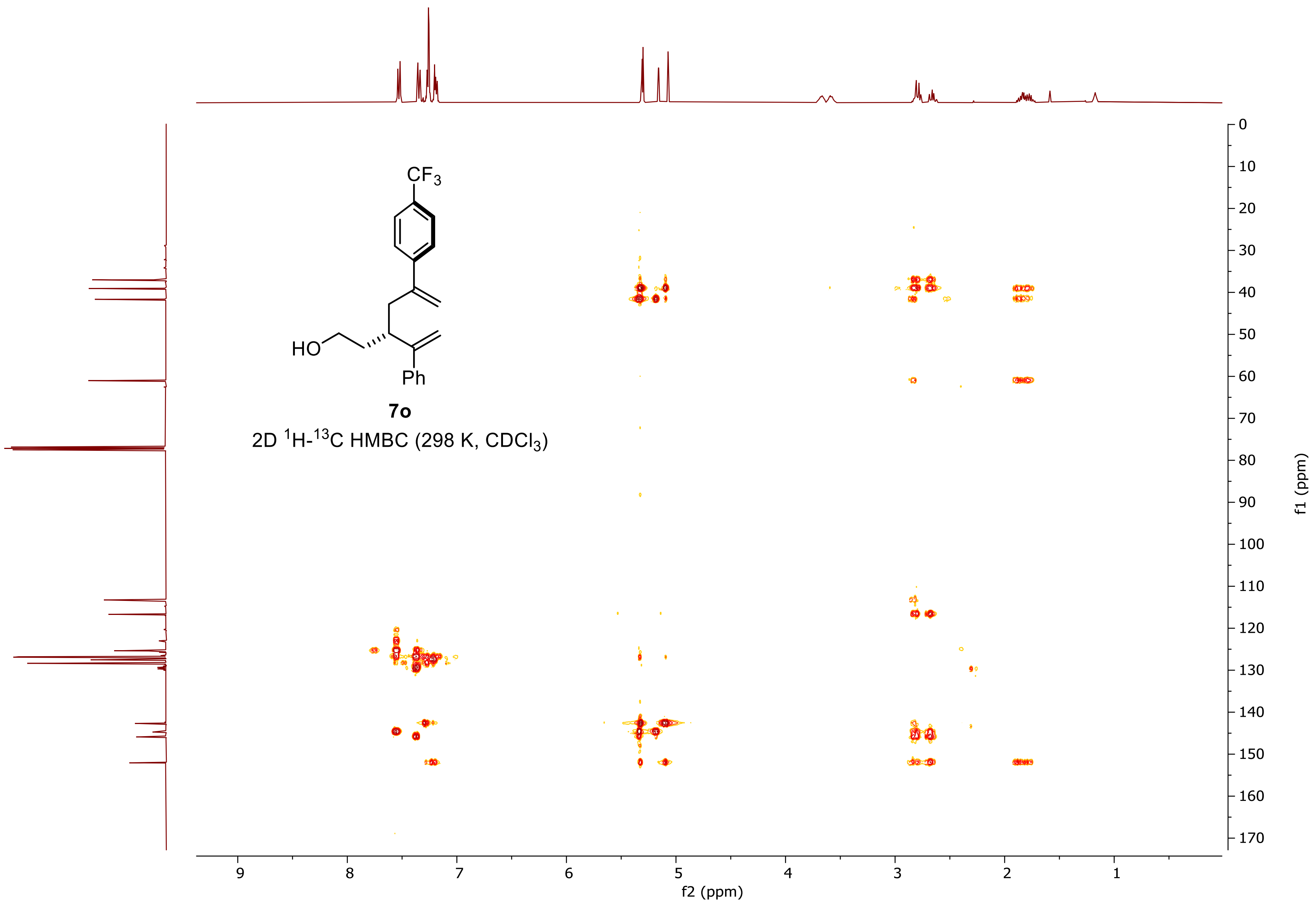
CF3
HO
Ph
7o
2D 1H-13C HMBC (298 K, CDCl3)
f1 (ppm)
f2 (ppm)

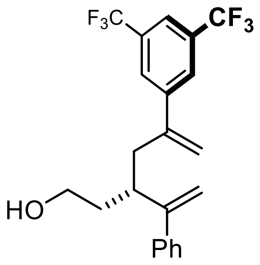

7p

^{1}H NMR (400 MHz, 298 K, $CDCl_3$)

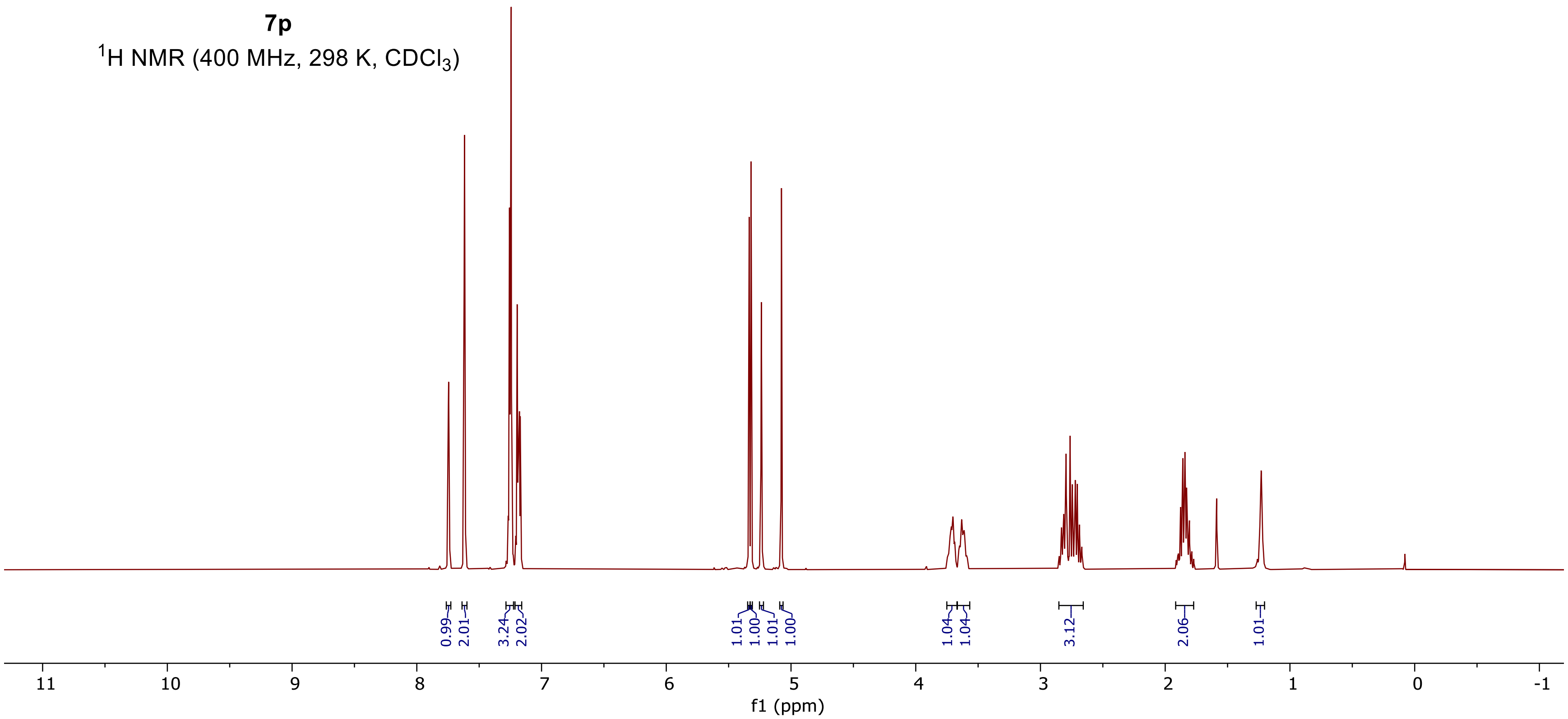

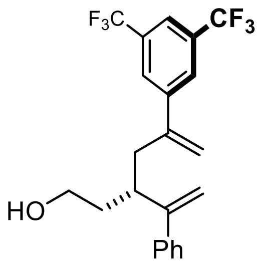

7p

$^{13}C\{^{1}H\}$ NMR (101 MHz, 298 K, $CDCl_3$)

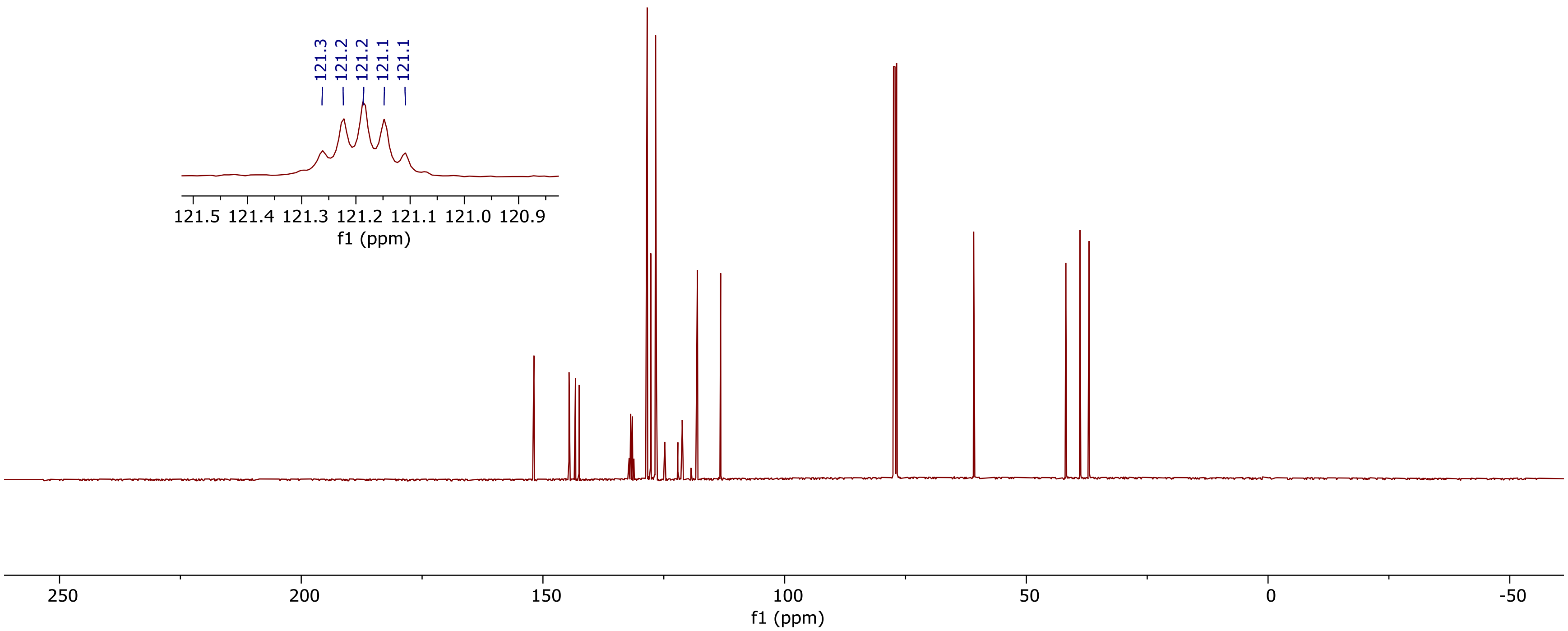

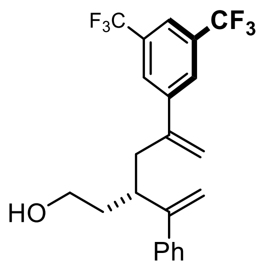

7p
$^{19}F\{^{1}H\}$ NMR (282 MHz, 298 K, $CDCl_3$)

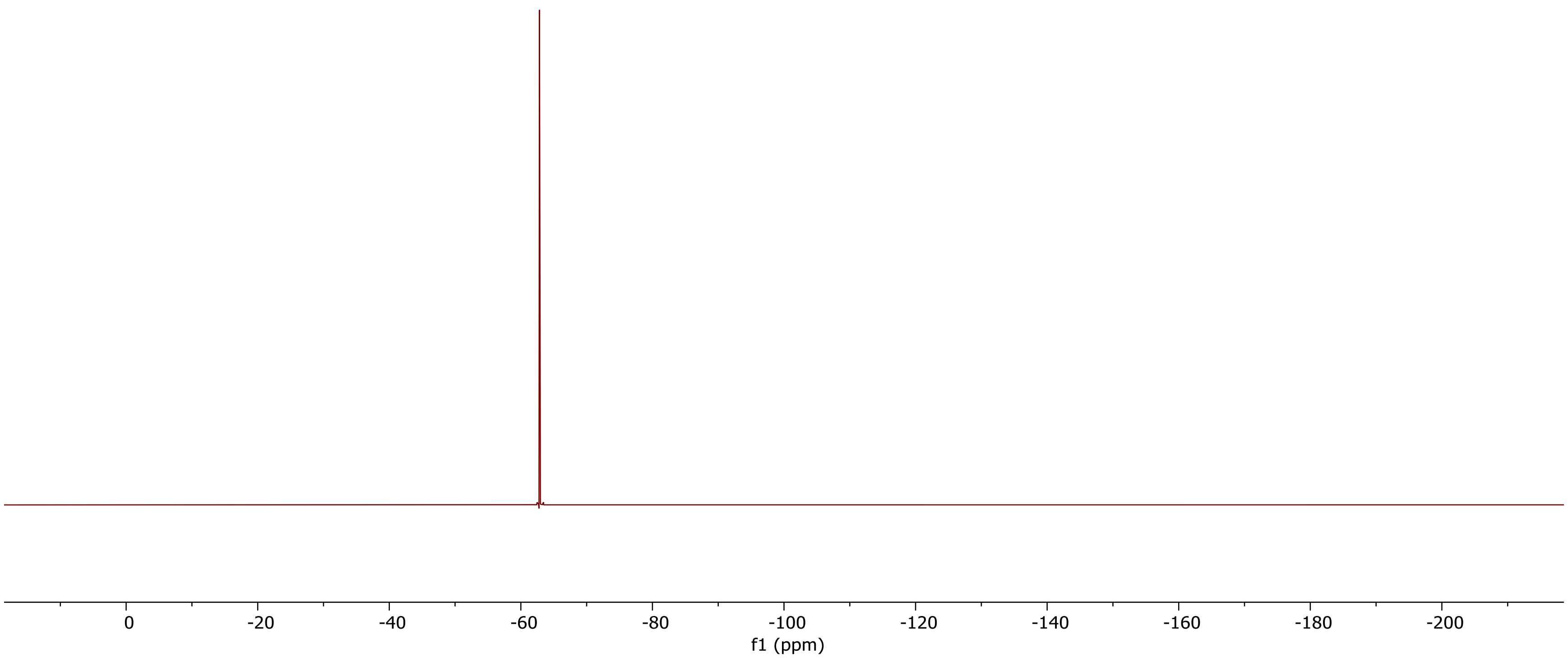

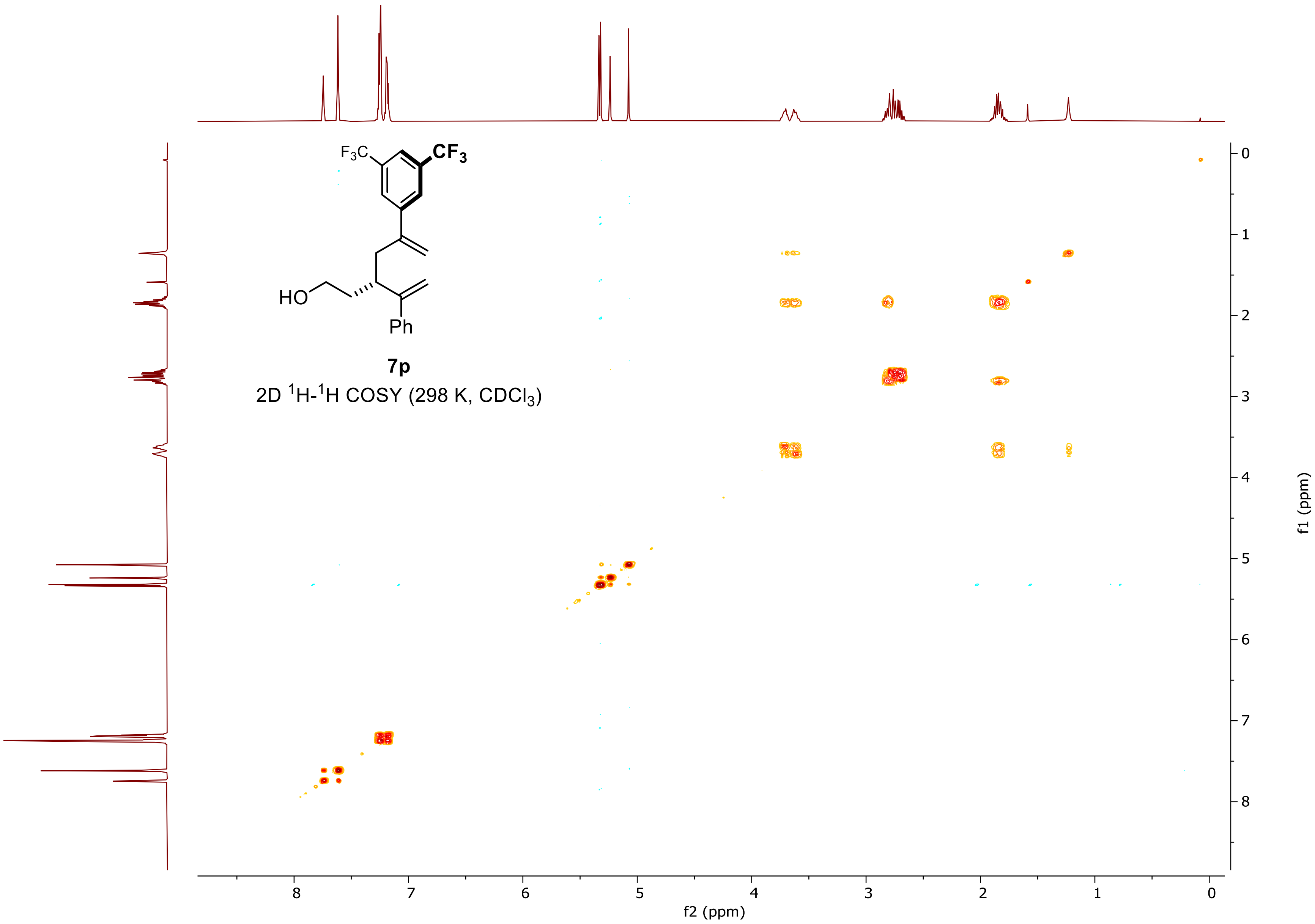

7p

2D ^{1}H-^{1}H COSY (298 K, $CDCl_3$)

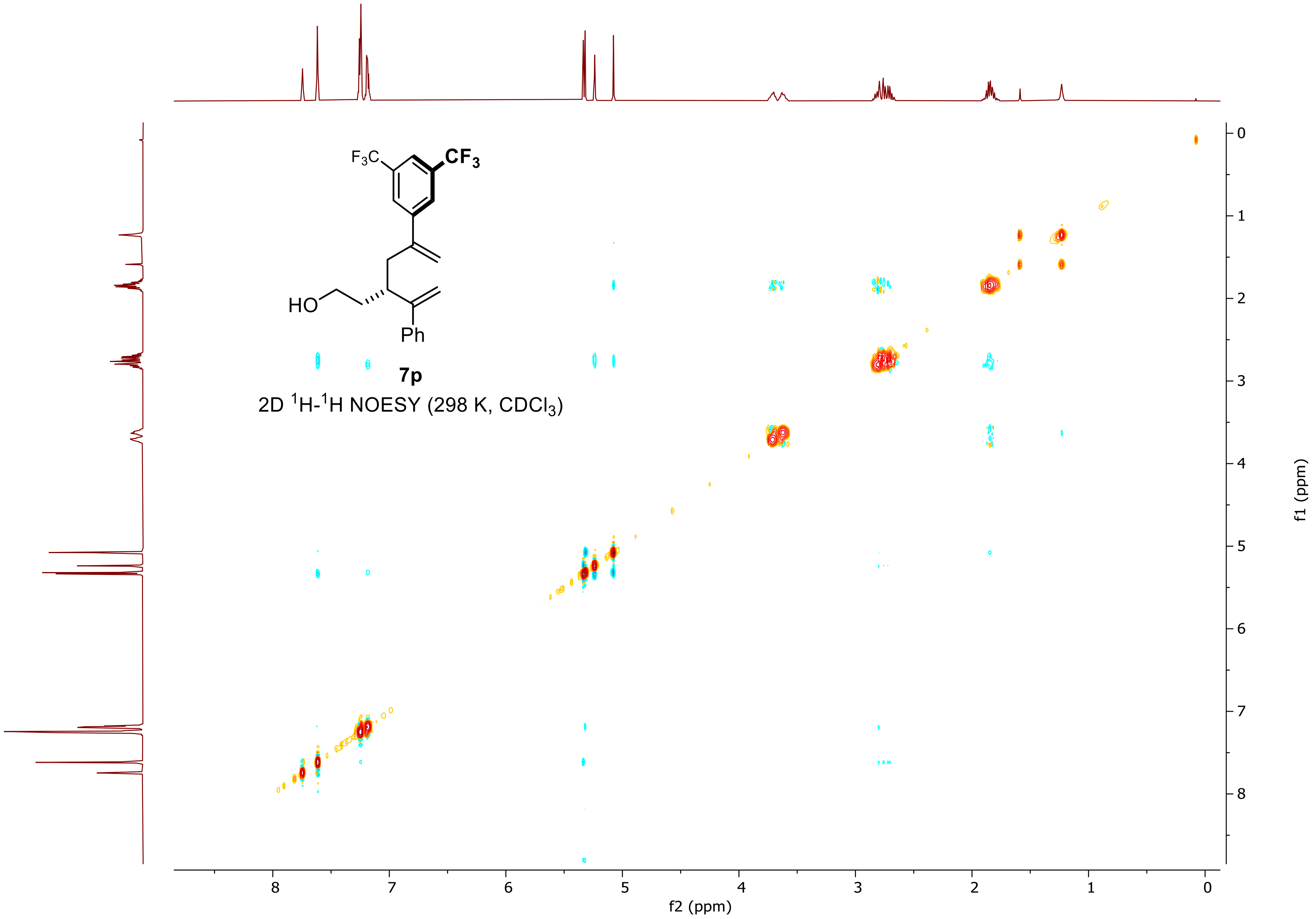
F3C
CF3
HO
Ph
7p
2D 1H-1H NOESY (298 K, CDCl3)
f2 (ppm)
f1 (ppm)

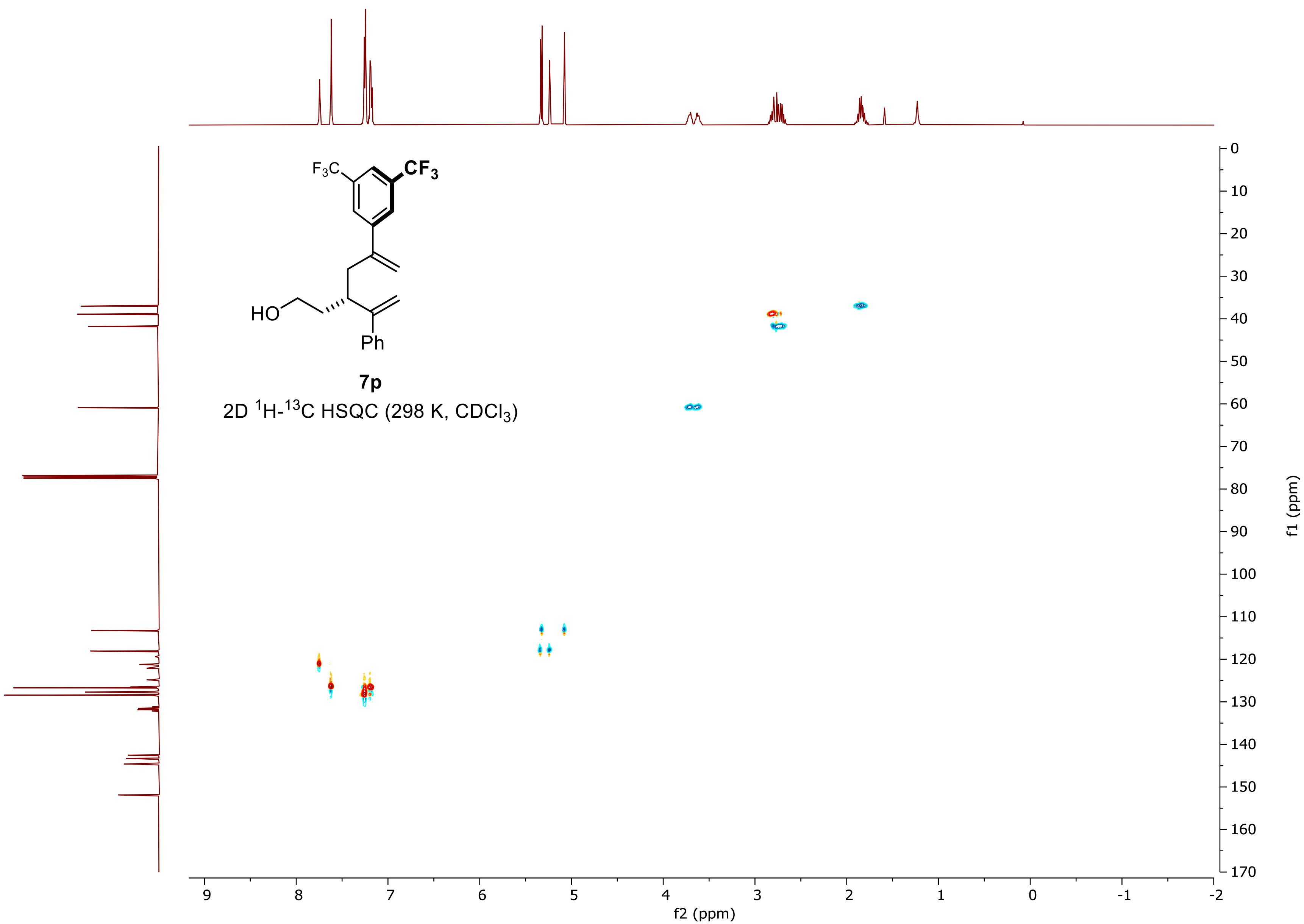

7p

2D ^{1}H-^{13}C HSQC (298 K, $CDCl_3$)

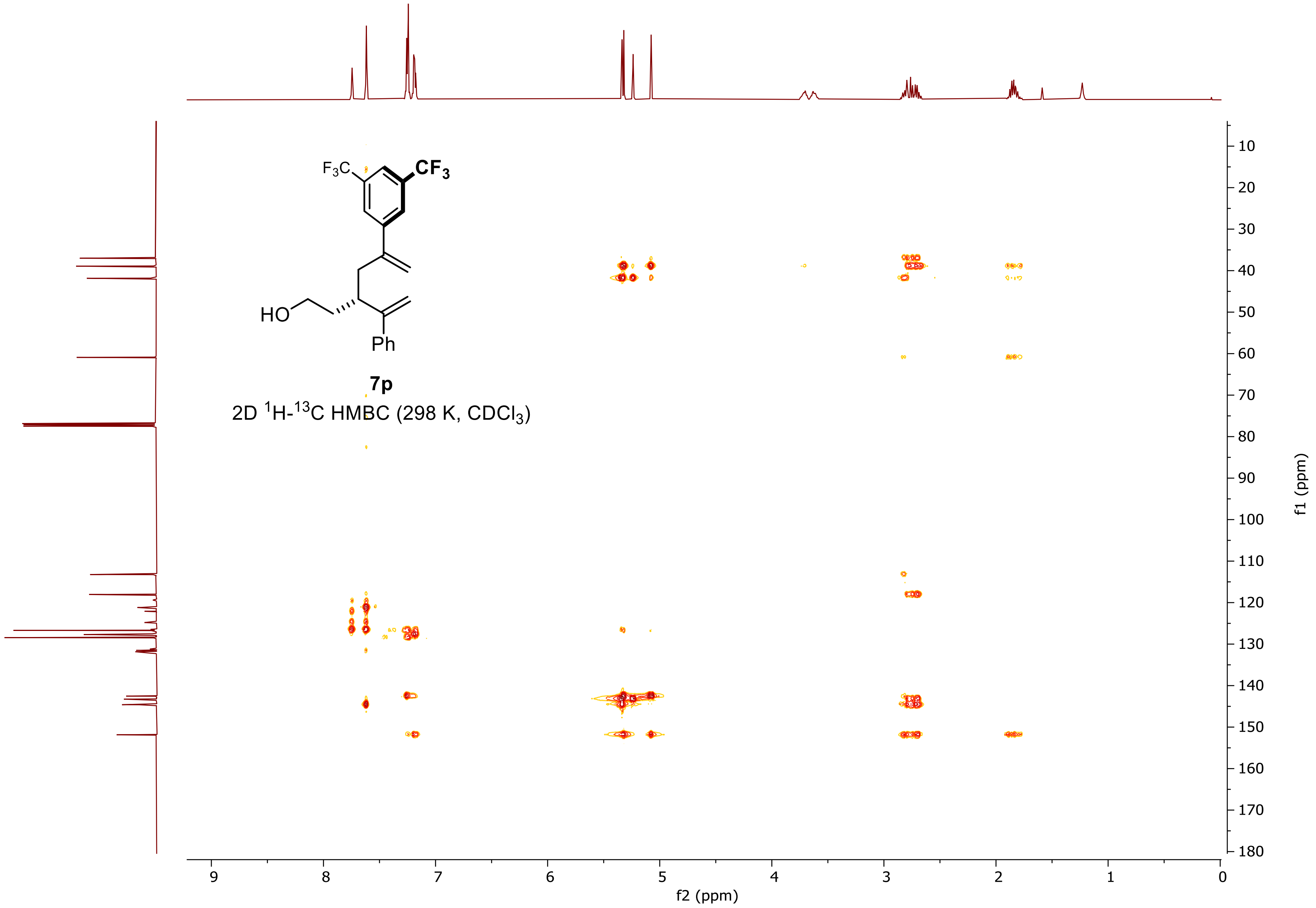

7p

2D ^{1}H-^{13}C HMBC (298 K, $CDCl_3$)

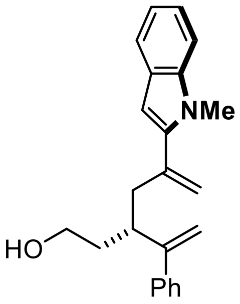

7q

^{1}H NMR (400 MHz, 298 K, $CDCl_3$)

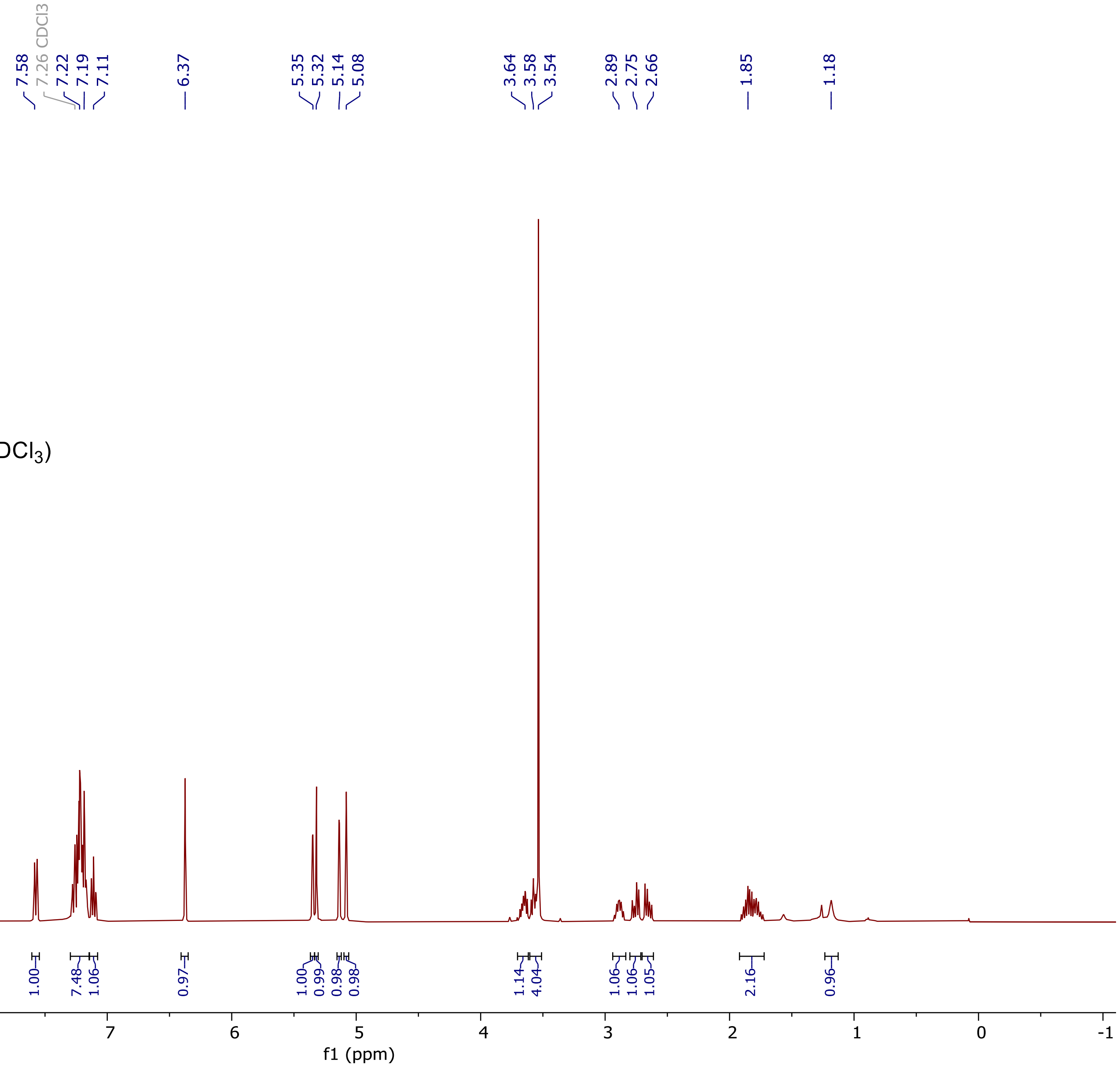

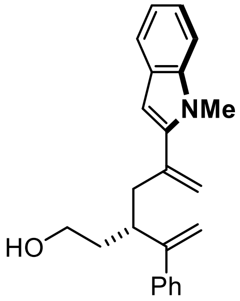

7q

$^{13}C\{^{1}H\}$ NMR (101 MHz, 298 K, $CDCl_3$)

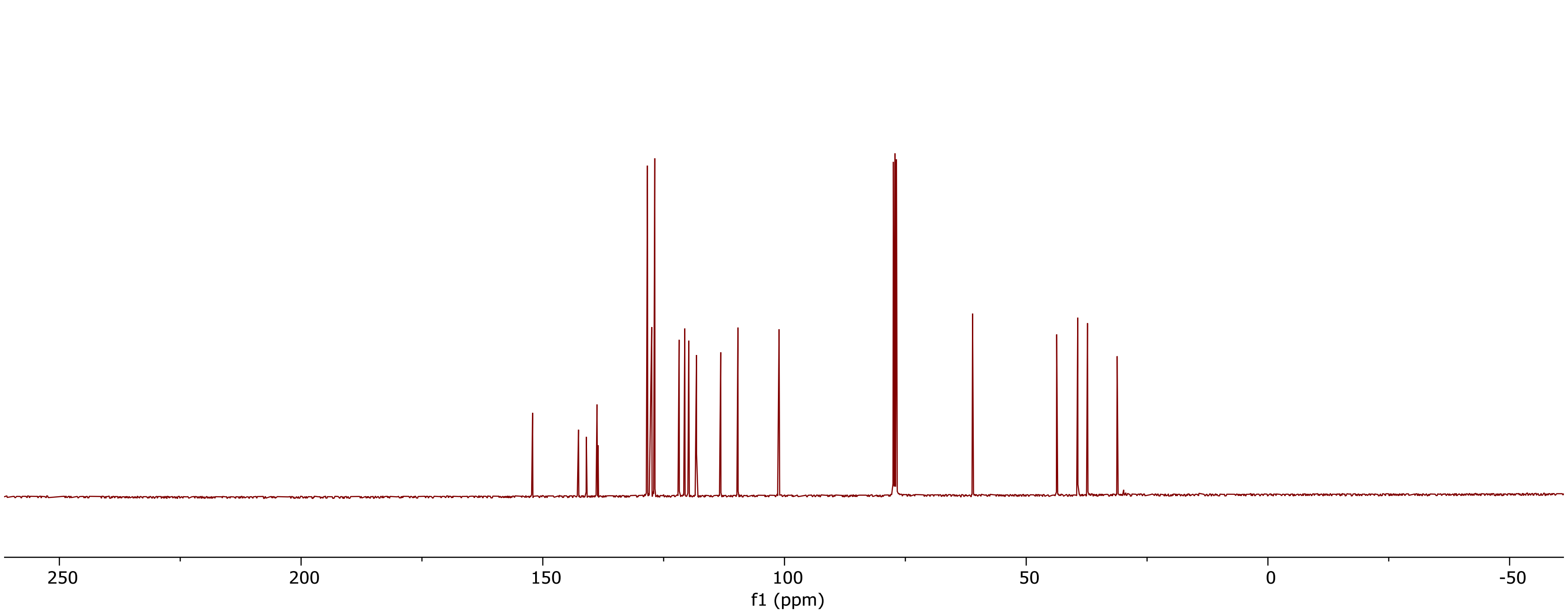

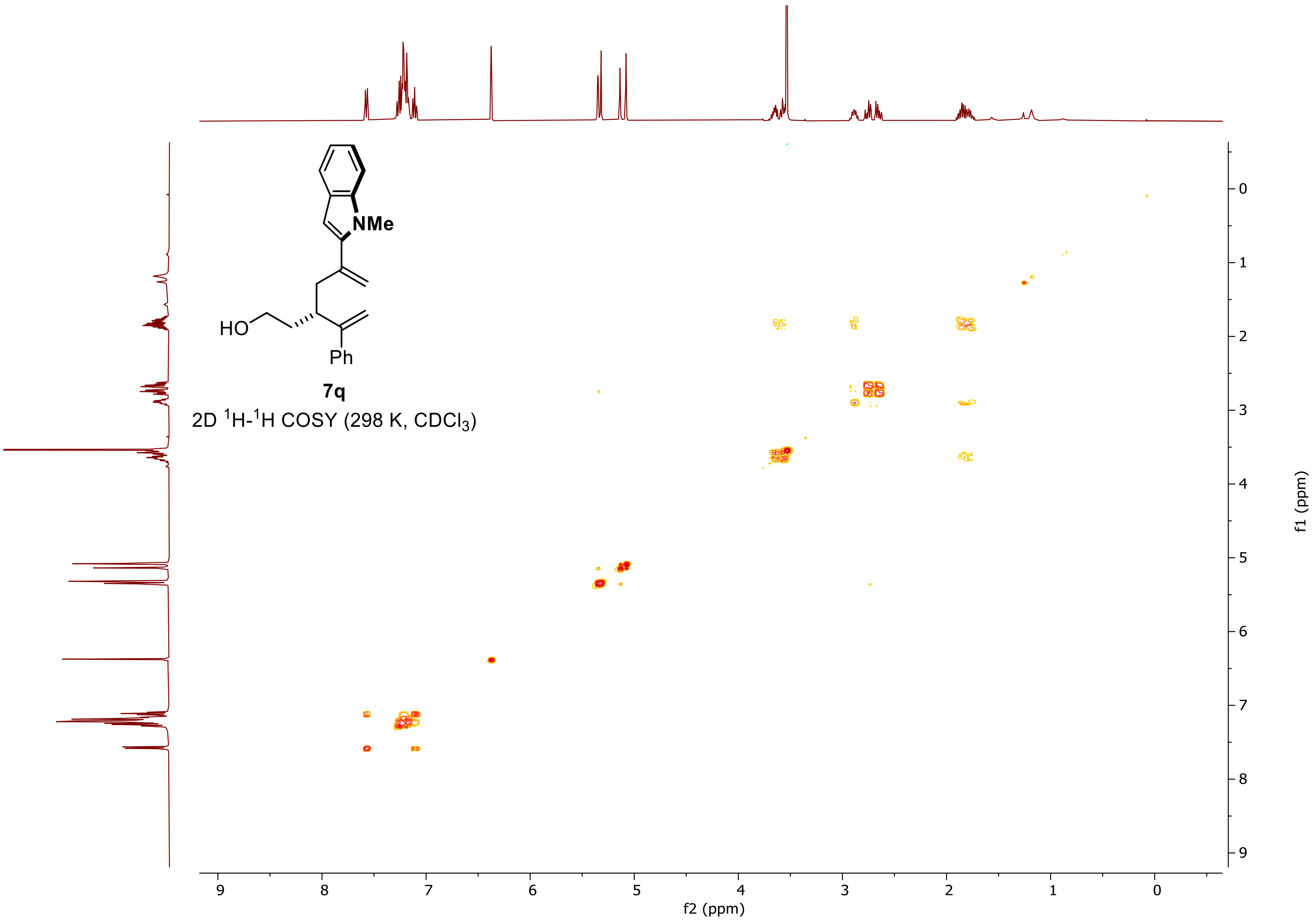

7q

2D ^{1}H-^{1}H COSY (298 K, $CDCl_3$)

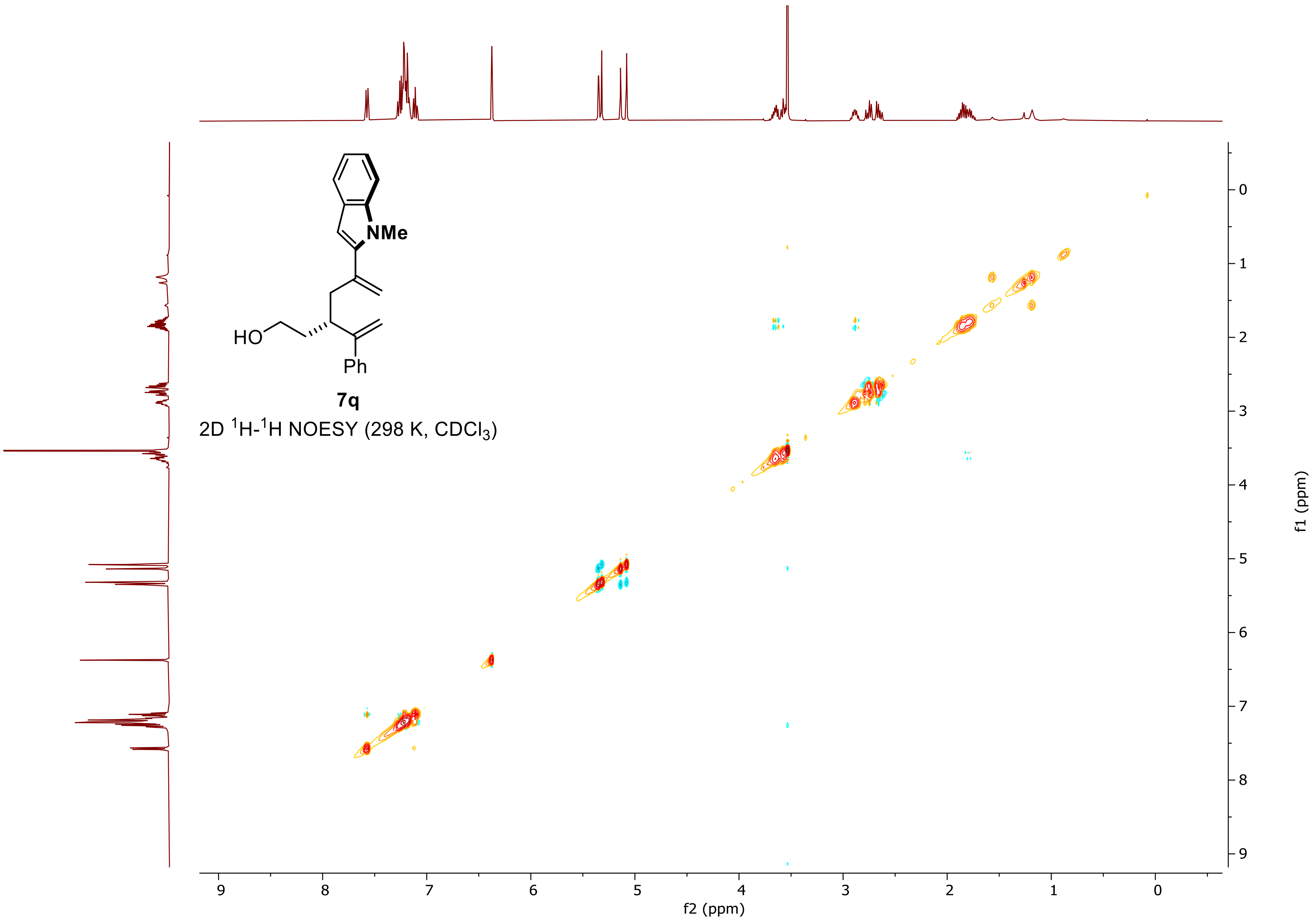
NMe
HO
Ph
7q
2D 1H-1H NOESY (298 K, CDCl3)
f2 (ppm)
f1 (ppm)
9
8
7
6
5
4
3
2
1
0

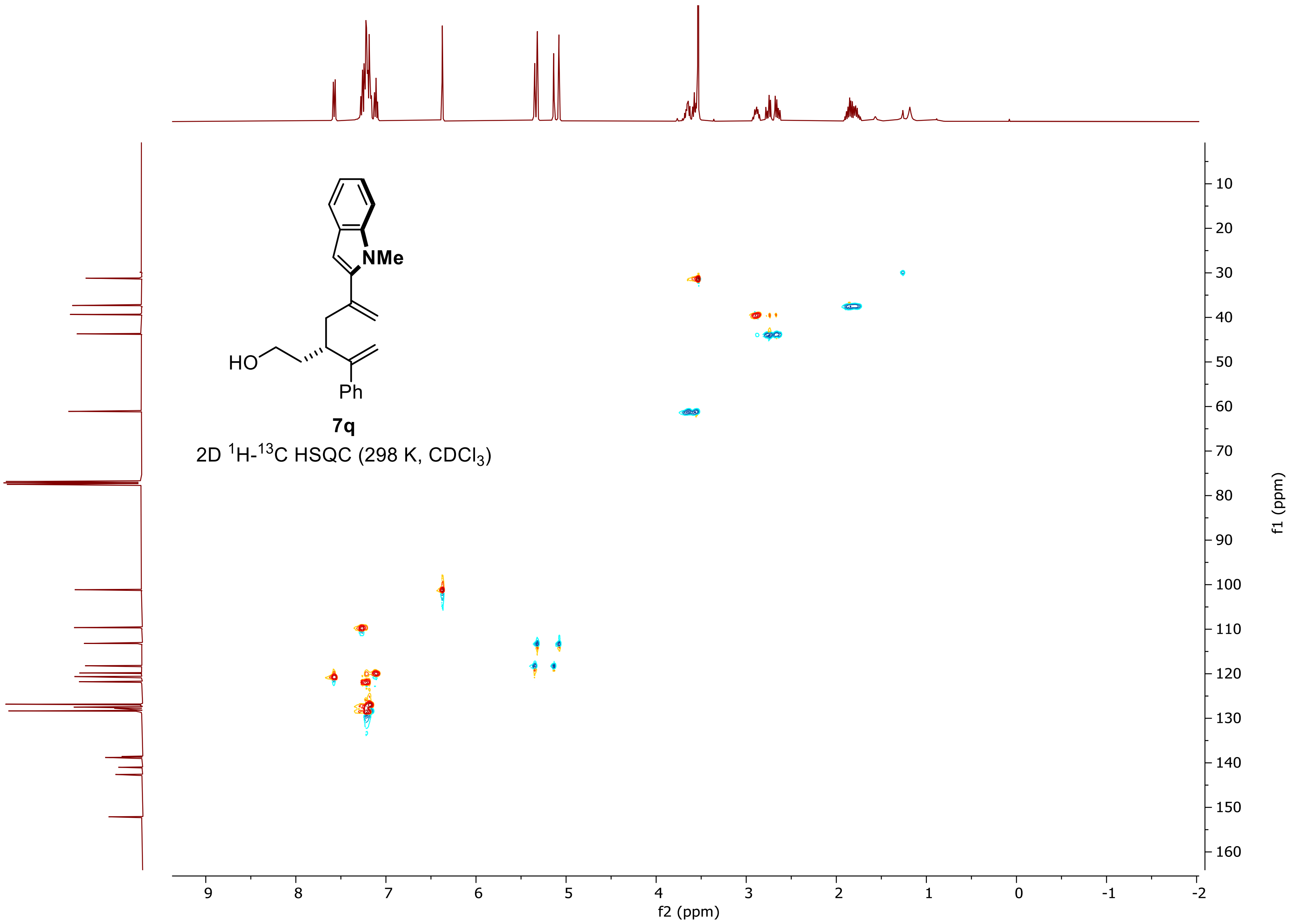
NMe
HO
Ph
7q
2D ^{1}H-^{13}C HSQC (298 K, CDCl$_3$)
f2 (ppm)
f1 (ppm)

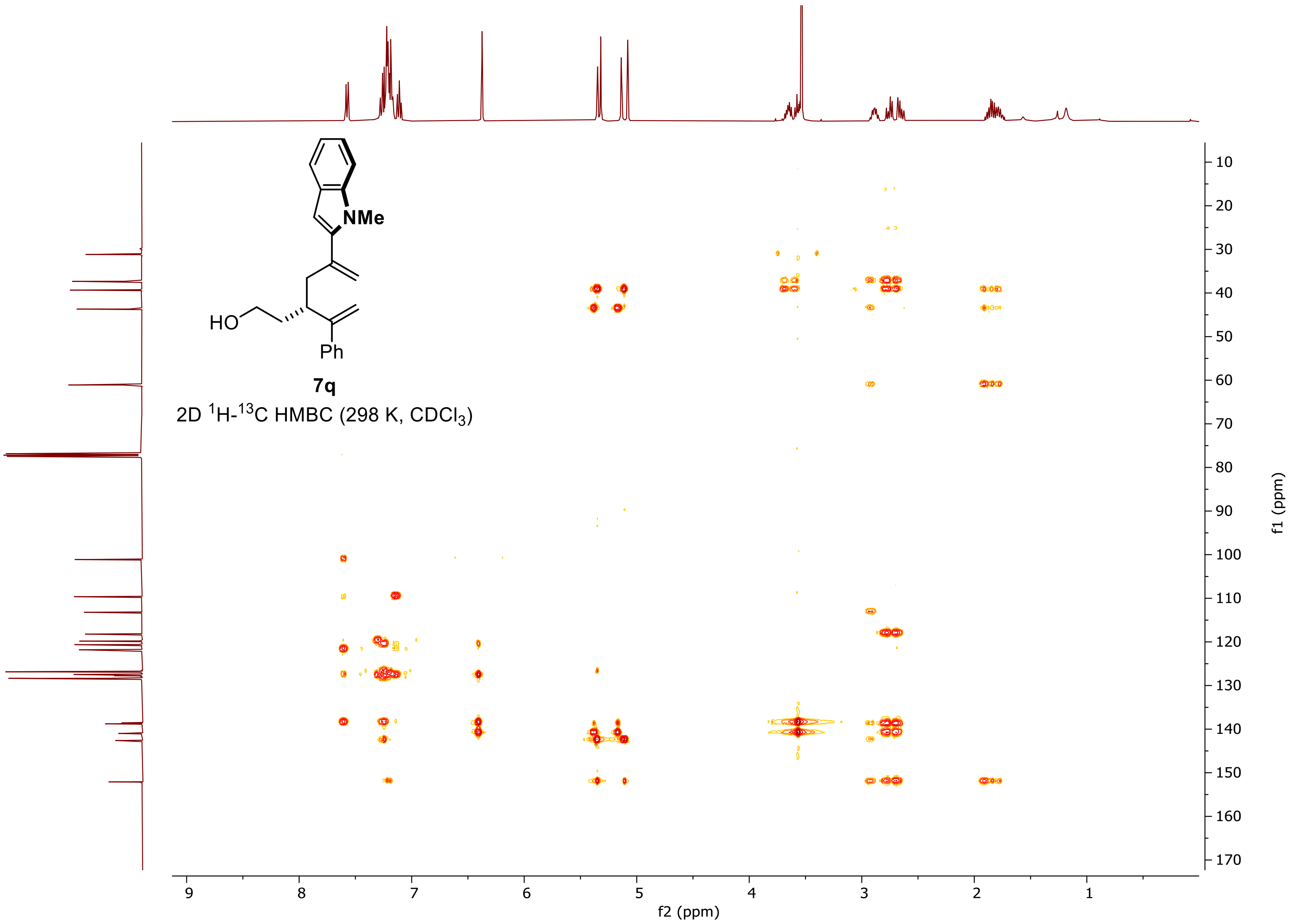
NMe
HO
Ph
7q
2D ^{1}H-^{13}C HMBC (298 K, CDCl$_3$)
f2 (ppm)
f1 (ppm)
9
8
7
6
5
4
3
2
1
10
20
30
40
50
60
70
80
90
100
110
120
130
140
150
160
170

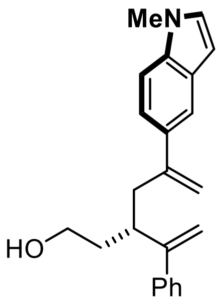

7r

^{1}H NMR (400 MHz, 298 K, $CDCl_3$)

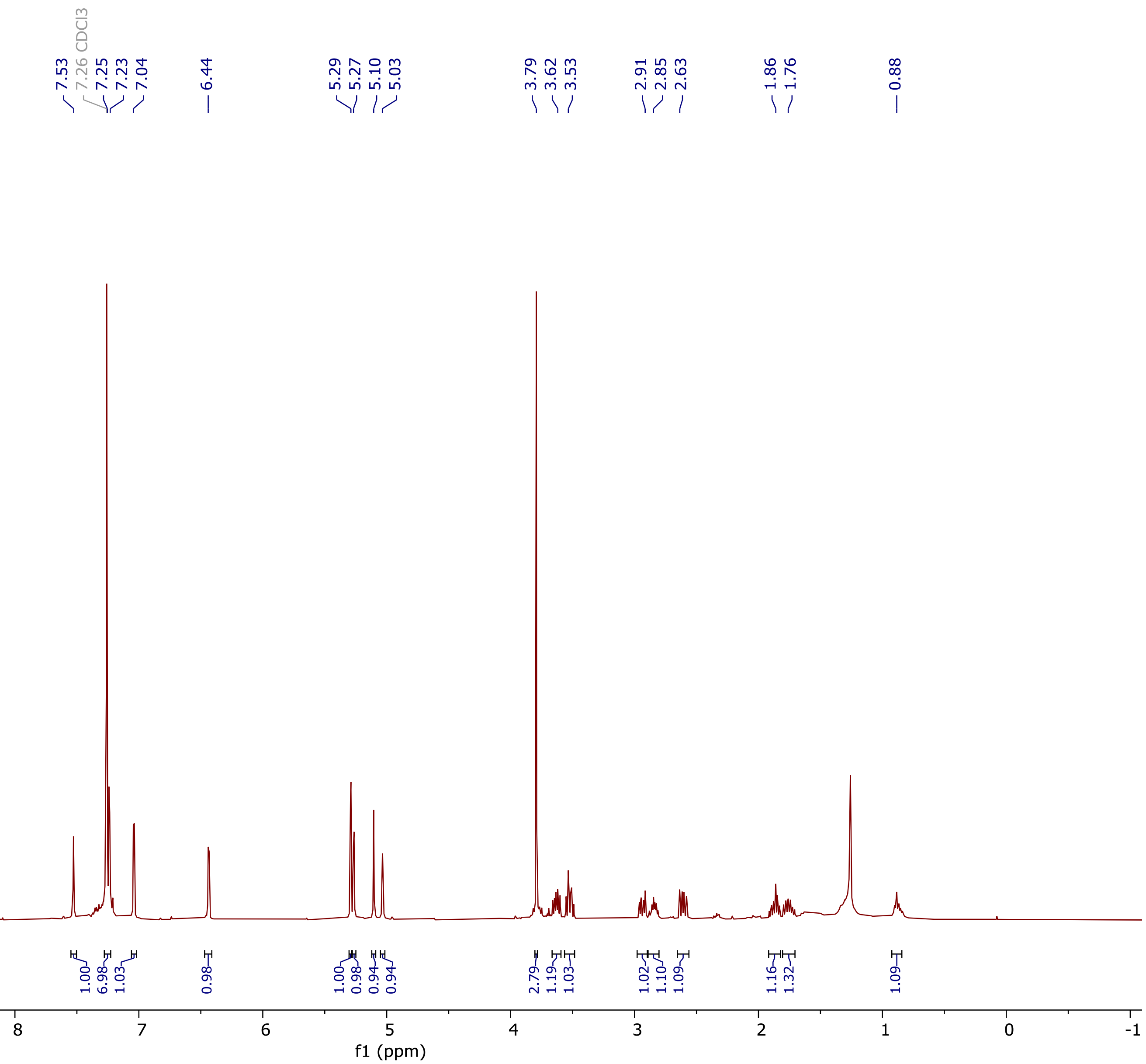

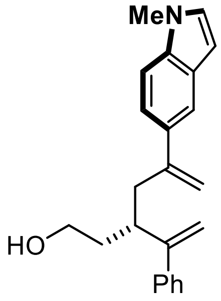

7r

$^{13}C\{^{1}H\}$ NMR (101 MHz, 298 K, $CDCl_3$)

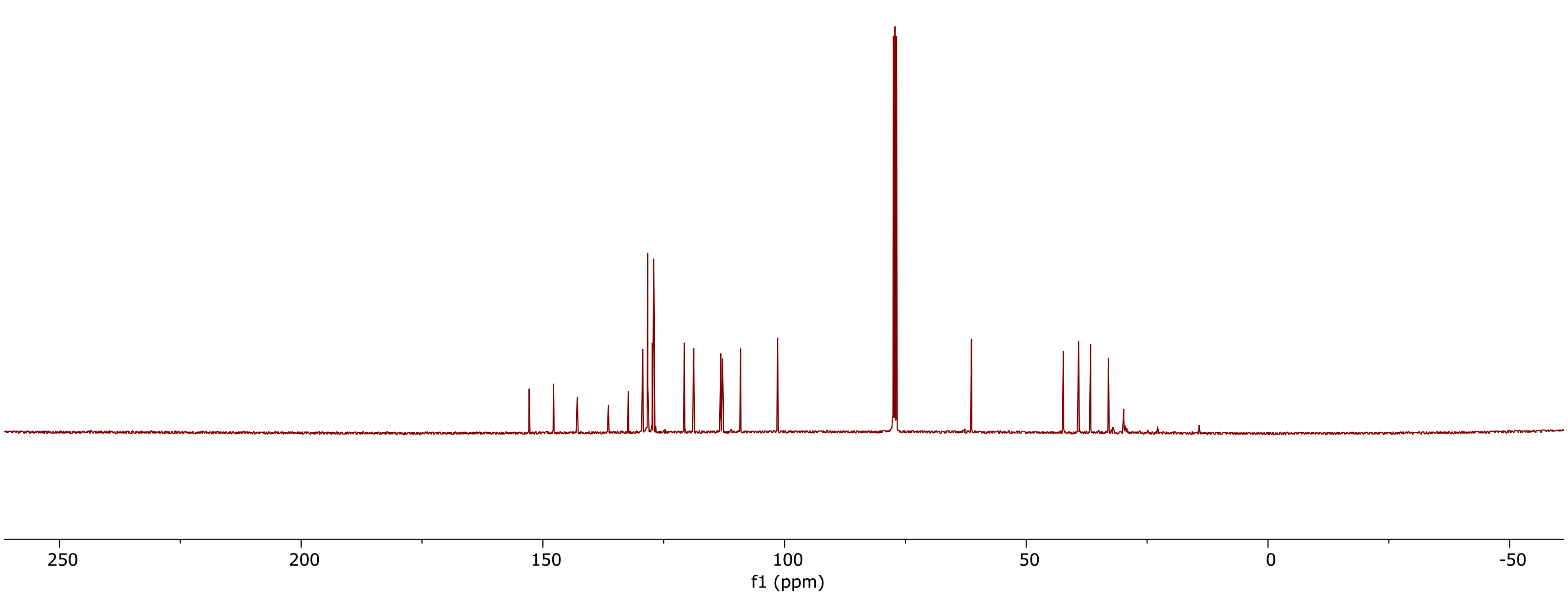

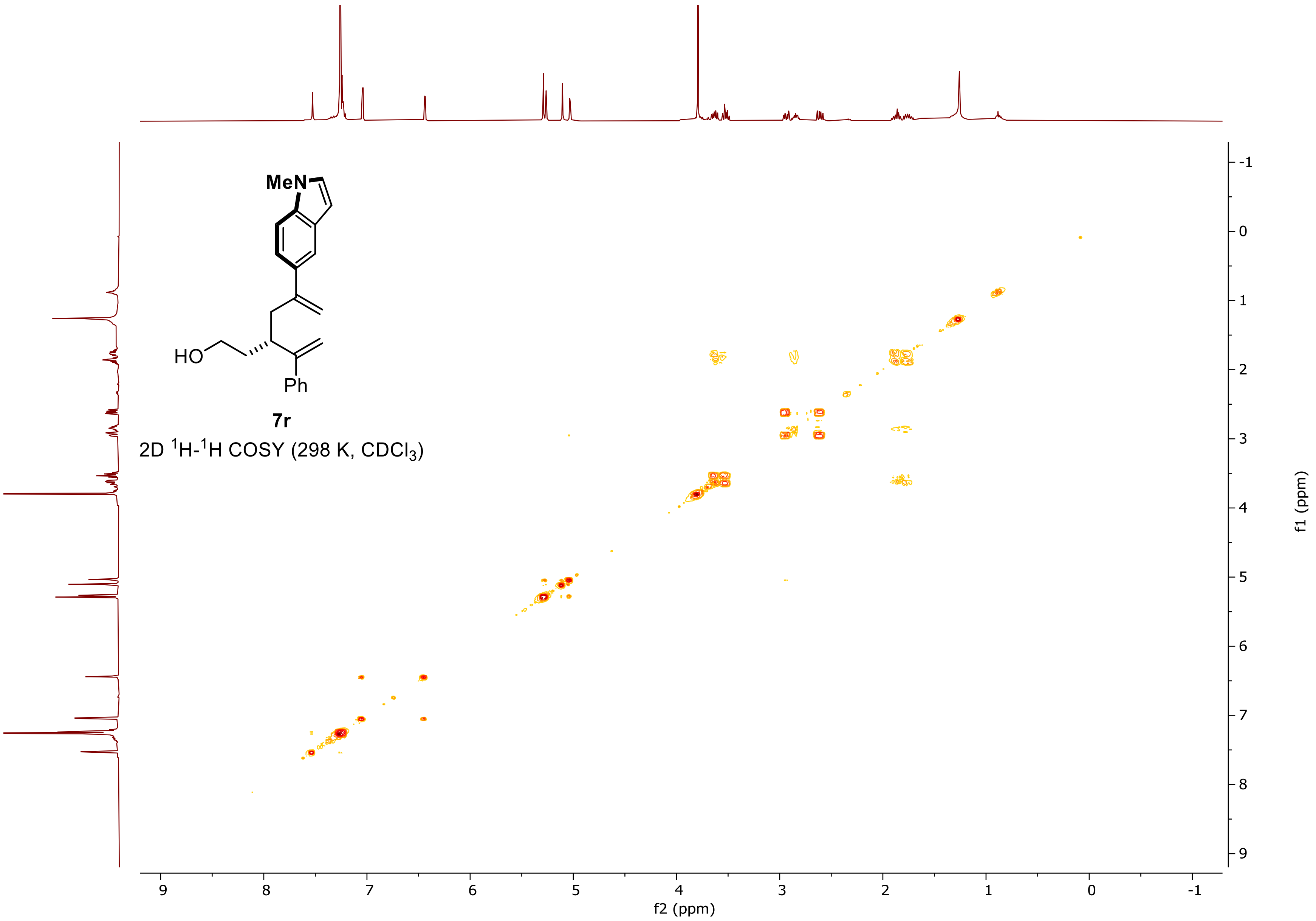
MeN
HO
Ph
7r
2D 1H-1H COSY (298 K, CDCl3)
f2 (ppm)
f1 (ppm)
9
8
7
6
5
4
3
2
1
0
-1

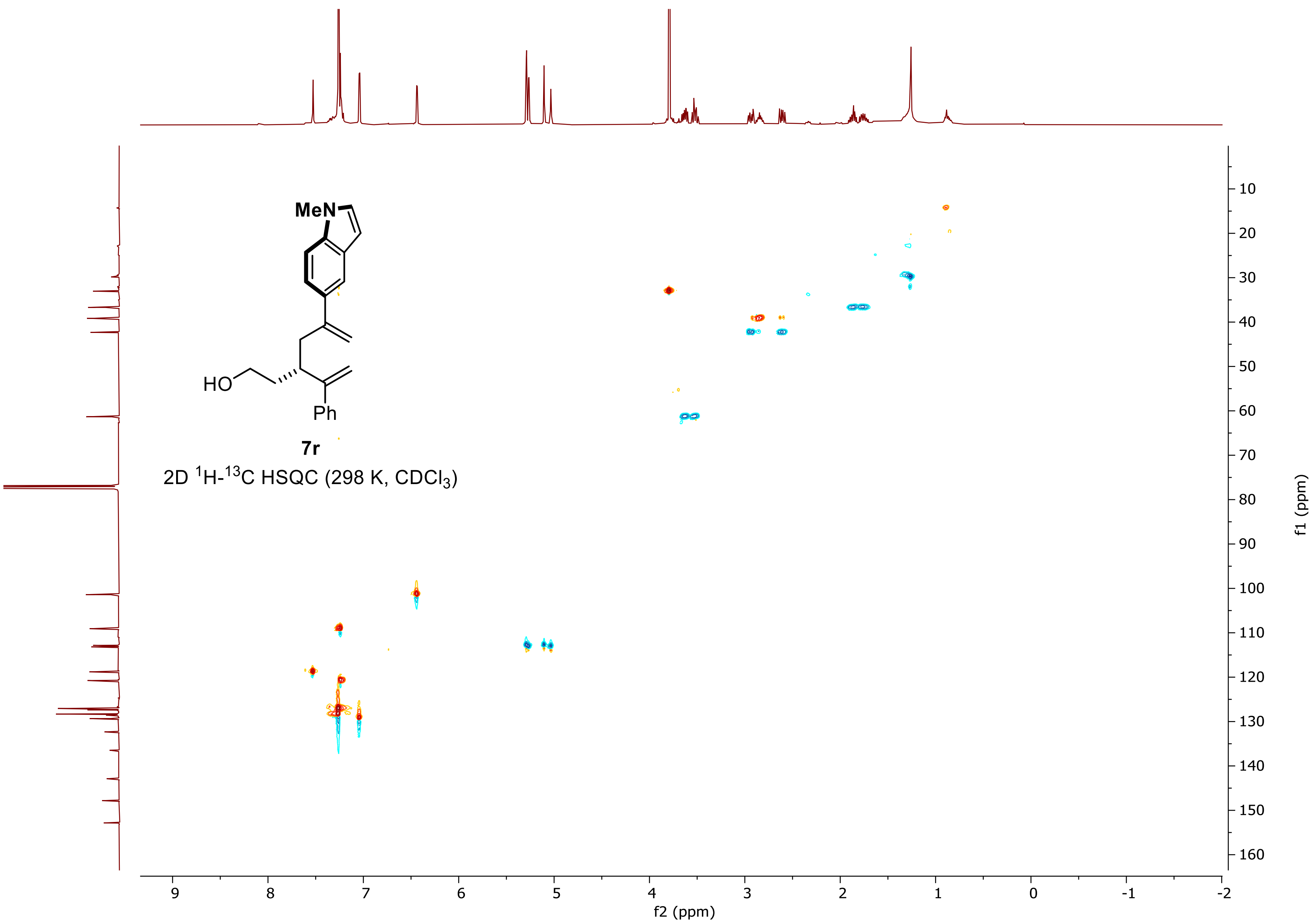

7r

2D ^{1}H-^{13}C HSQC (298 K, $CDCl_3$)

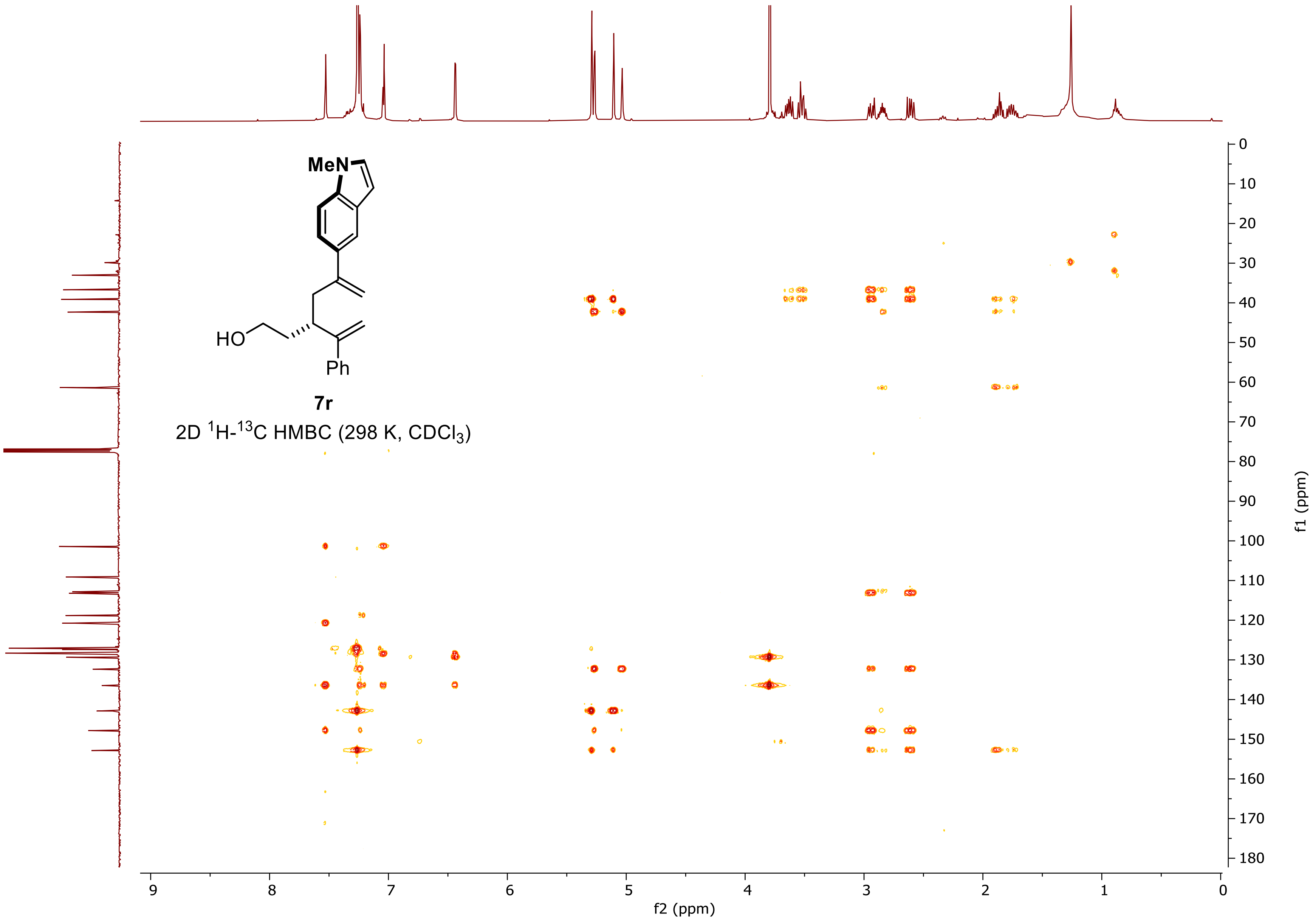
MeN
HO
Ph
7r
2D ¹H-¹³C HMBC (298 K, CDCl₃)
f2 (ppm)
f1 (ppm)

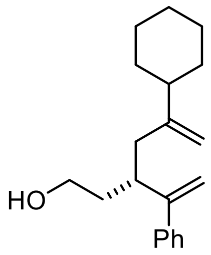

7s

^{1}H NMR (400 MHz, 298 K, $CDCl_3$)

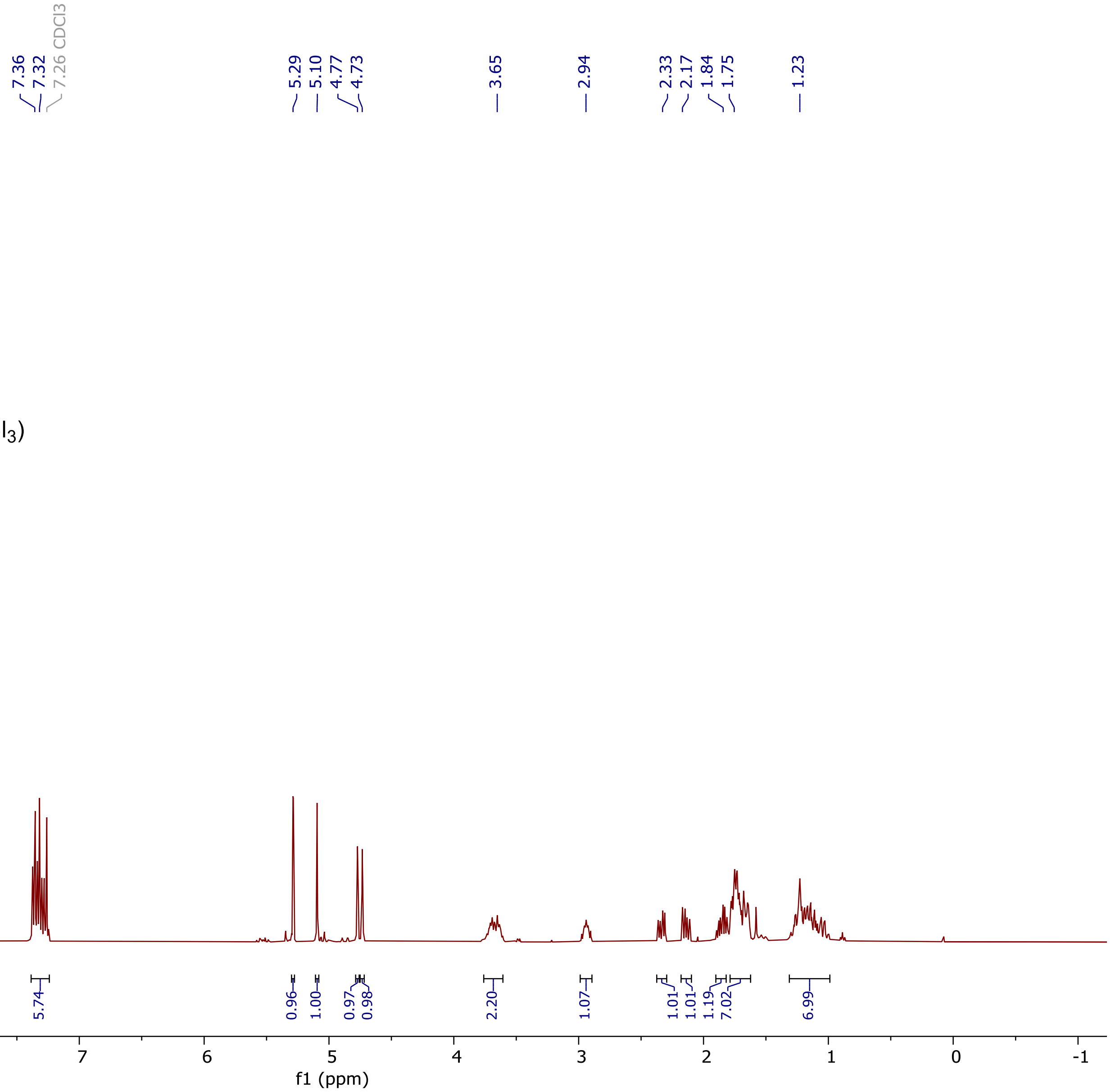

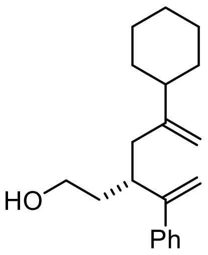

7s

$^{13}C\{^1H\}$ NMR (101 MHz, 298 K, $CDCl_3$)

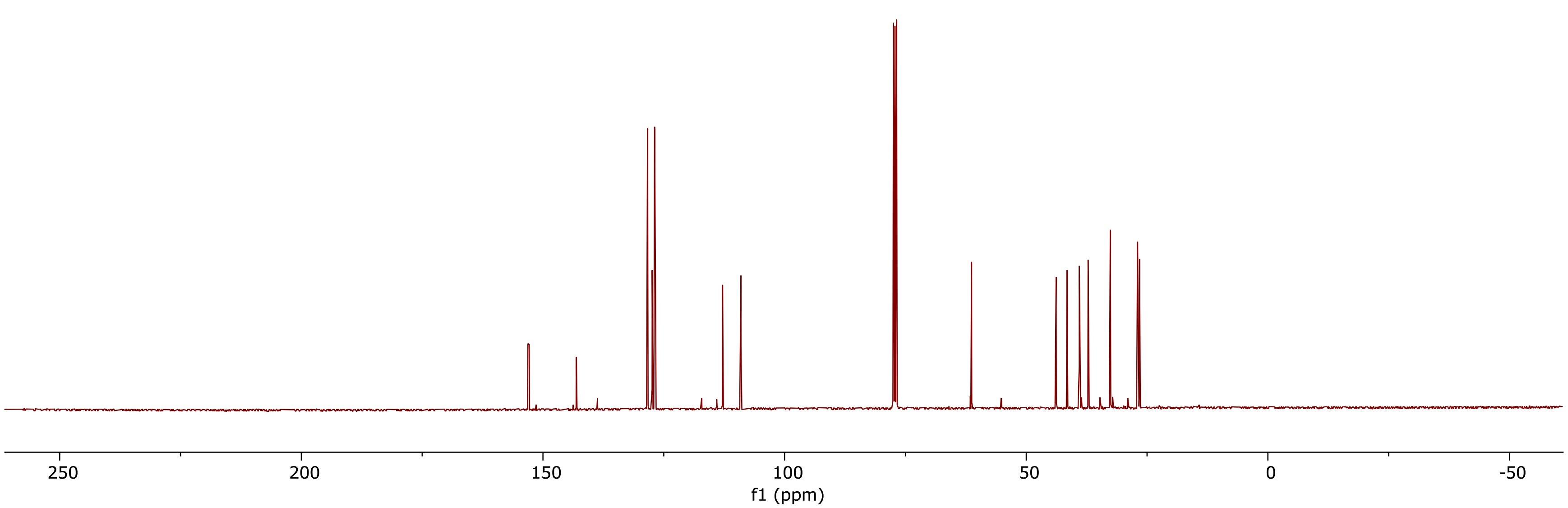

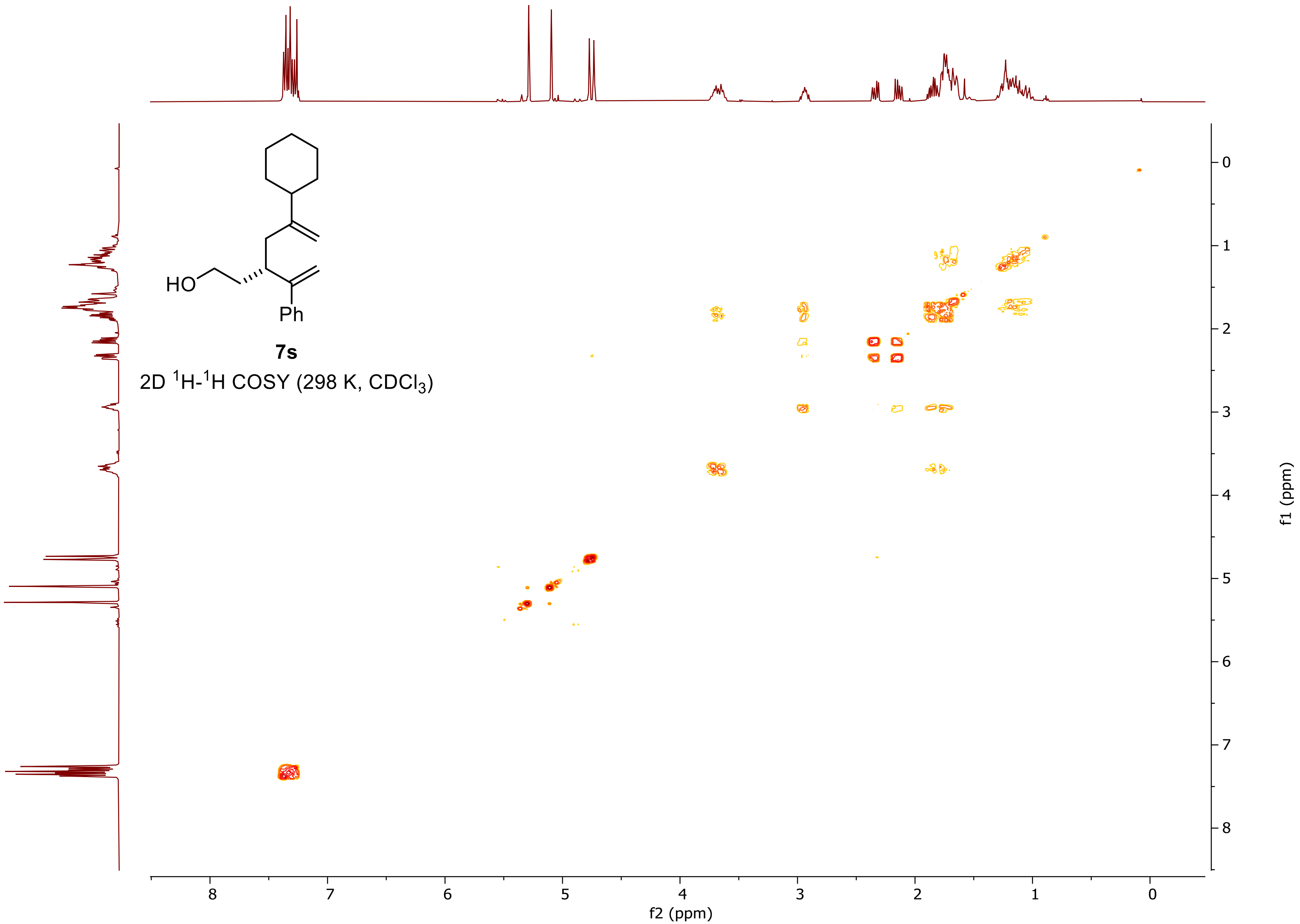

7s

2D ^{1}H-^{1}H COSY (298 K, $CDCl_3$)

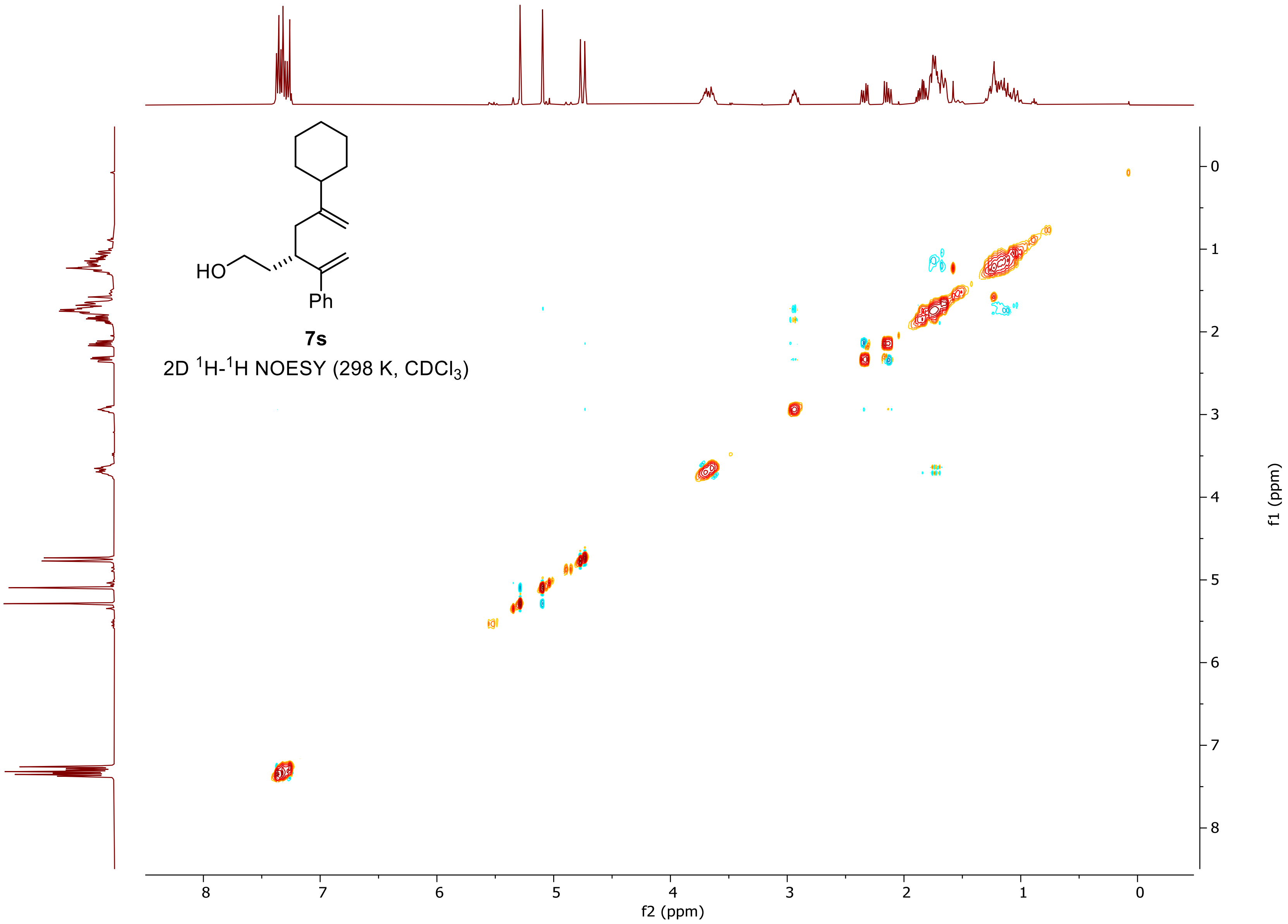
HO
Ph
7s
2D ¹H-¹H NOESY (298 K, CDCl₃)
f2 (ppm)
f1 (ppm)
8
7
6
5
4
3
2
1
0

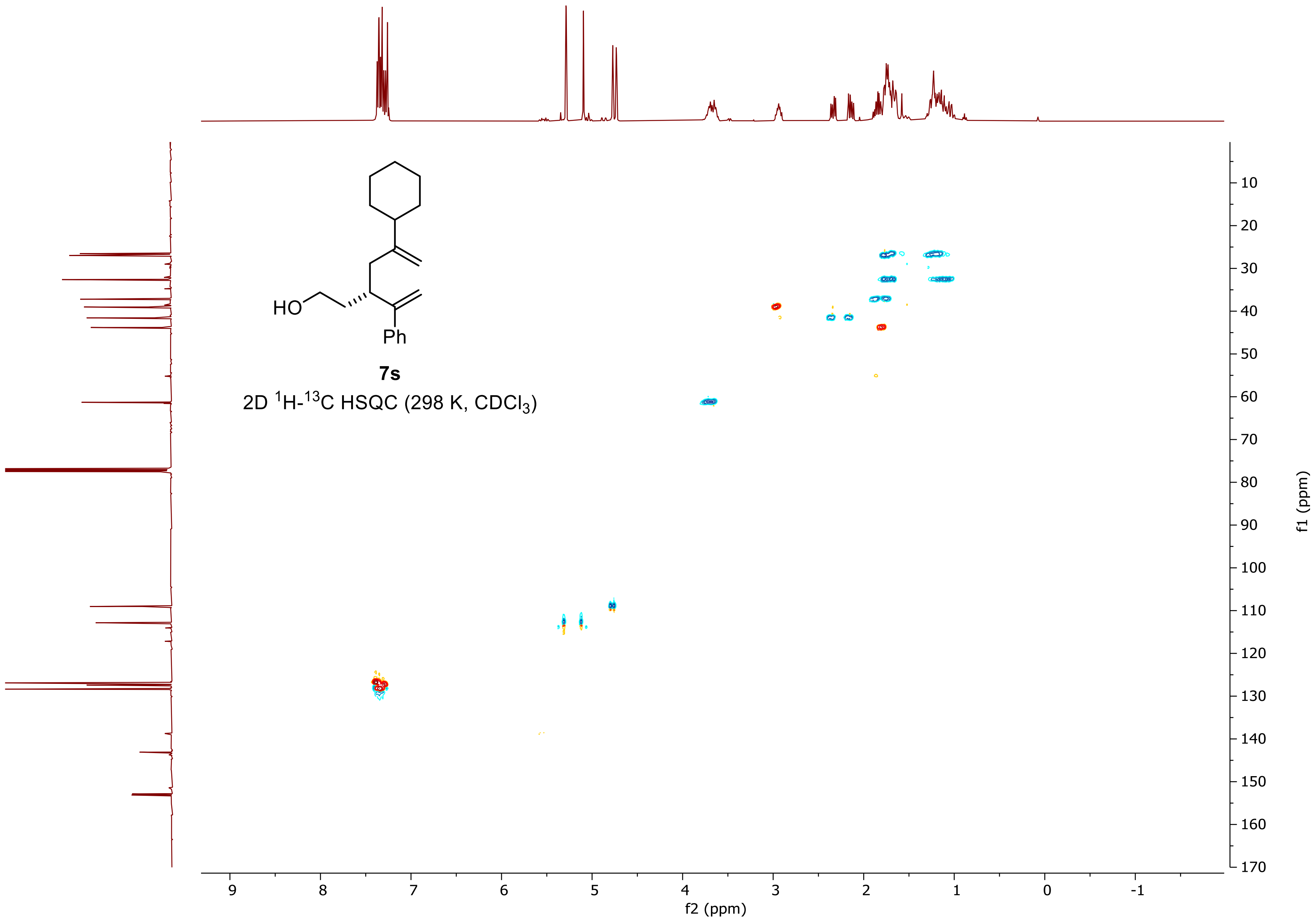
HO
Ph
7s
2D ¹H-¹³C HSQC (298 K, CDCl₃)
f2 (ppm)
f1 (ppm)

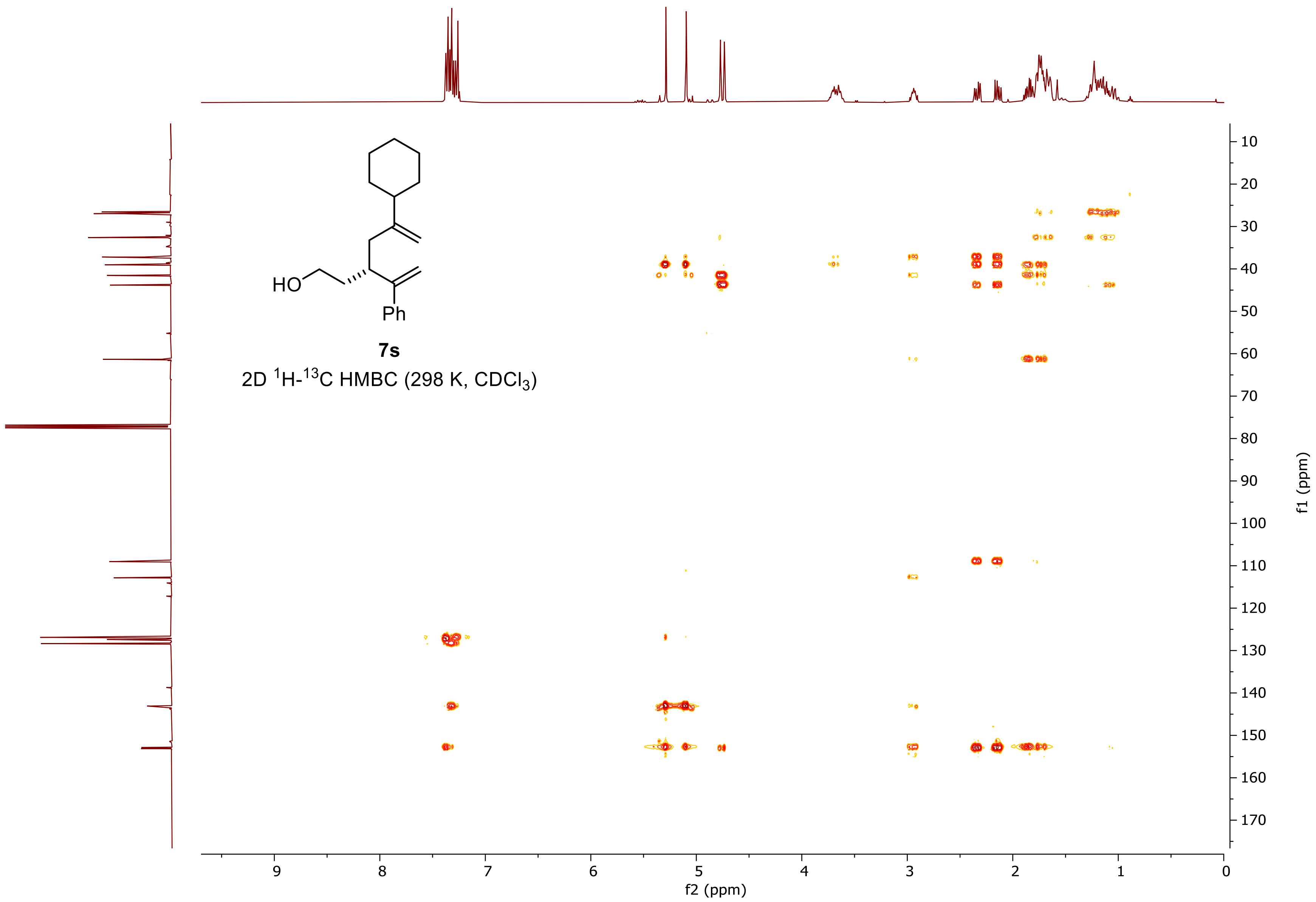

HO
Ph
7s
2D ^{1}H-^{13}C HMBC (298 K, $CDCl_3$)
f1 (ppm)
f2 (ppm)

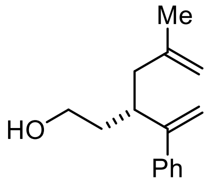

7t

^{1}H NMR (400 MHz, 298 K, $CDCl_3$)

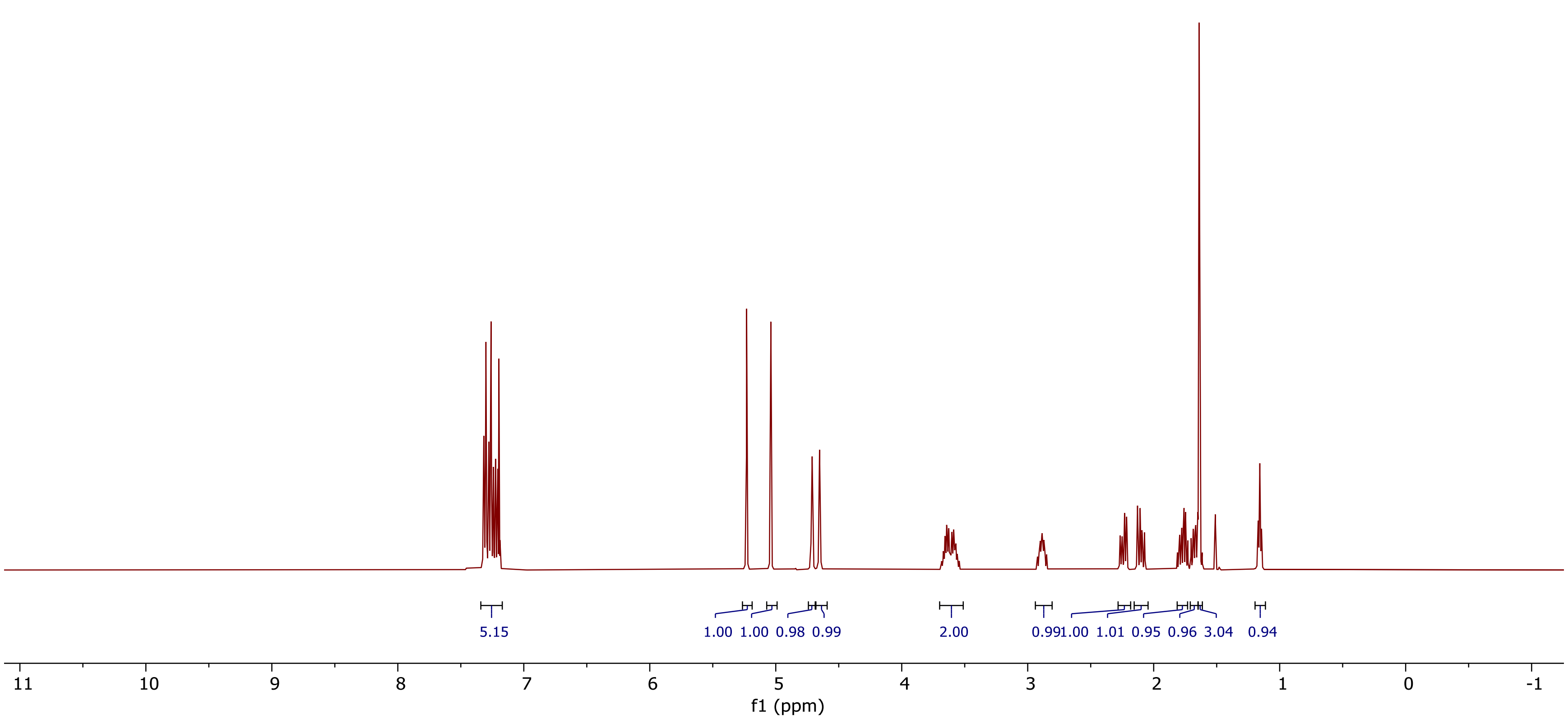

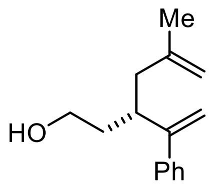

7t

$^{13}C\{^{1}H\}$ NMR (101 MHz, 298 K, $CDCl_3$)

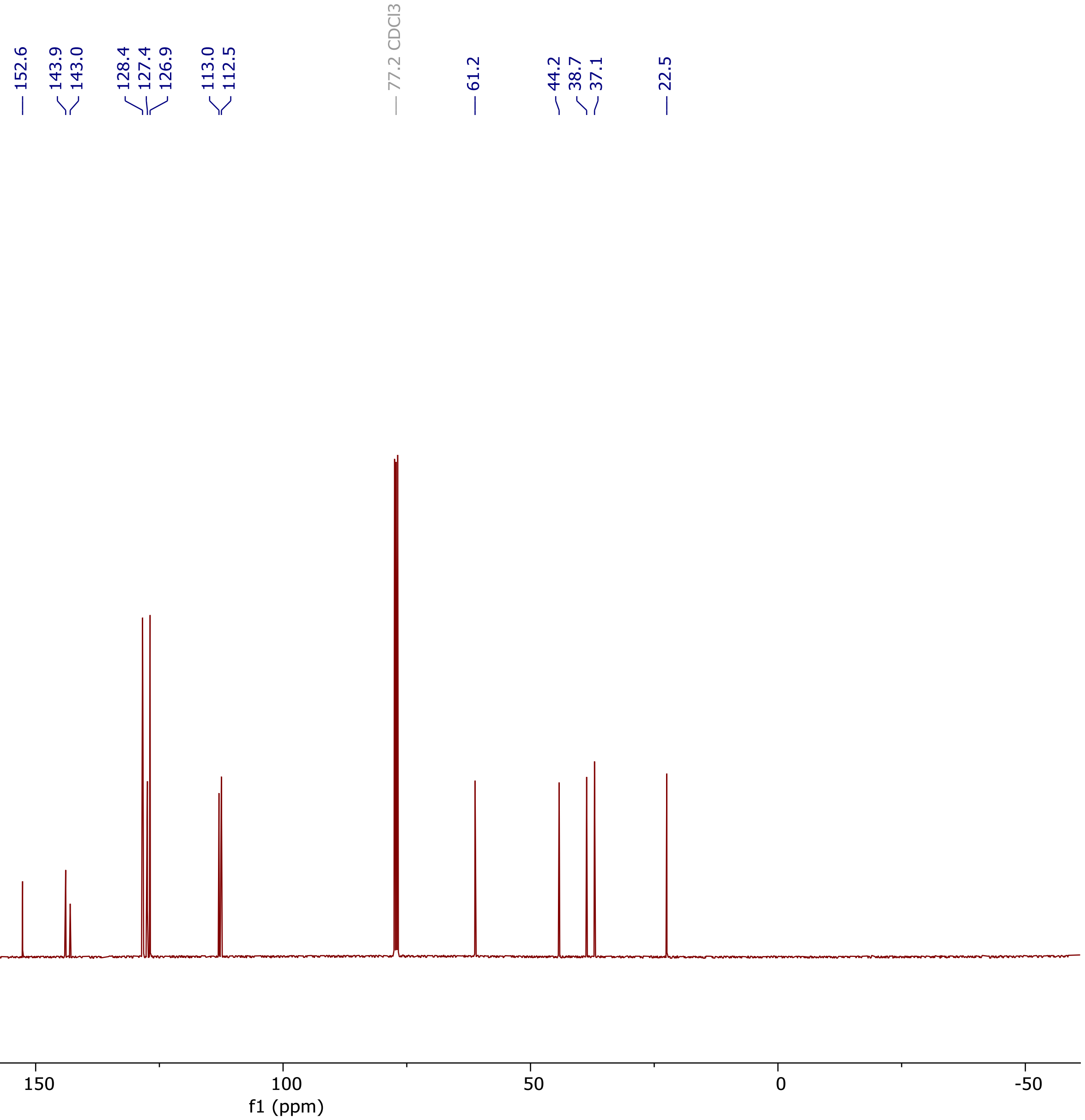

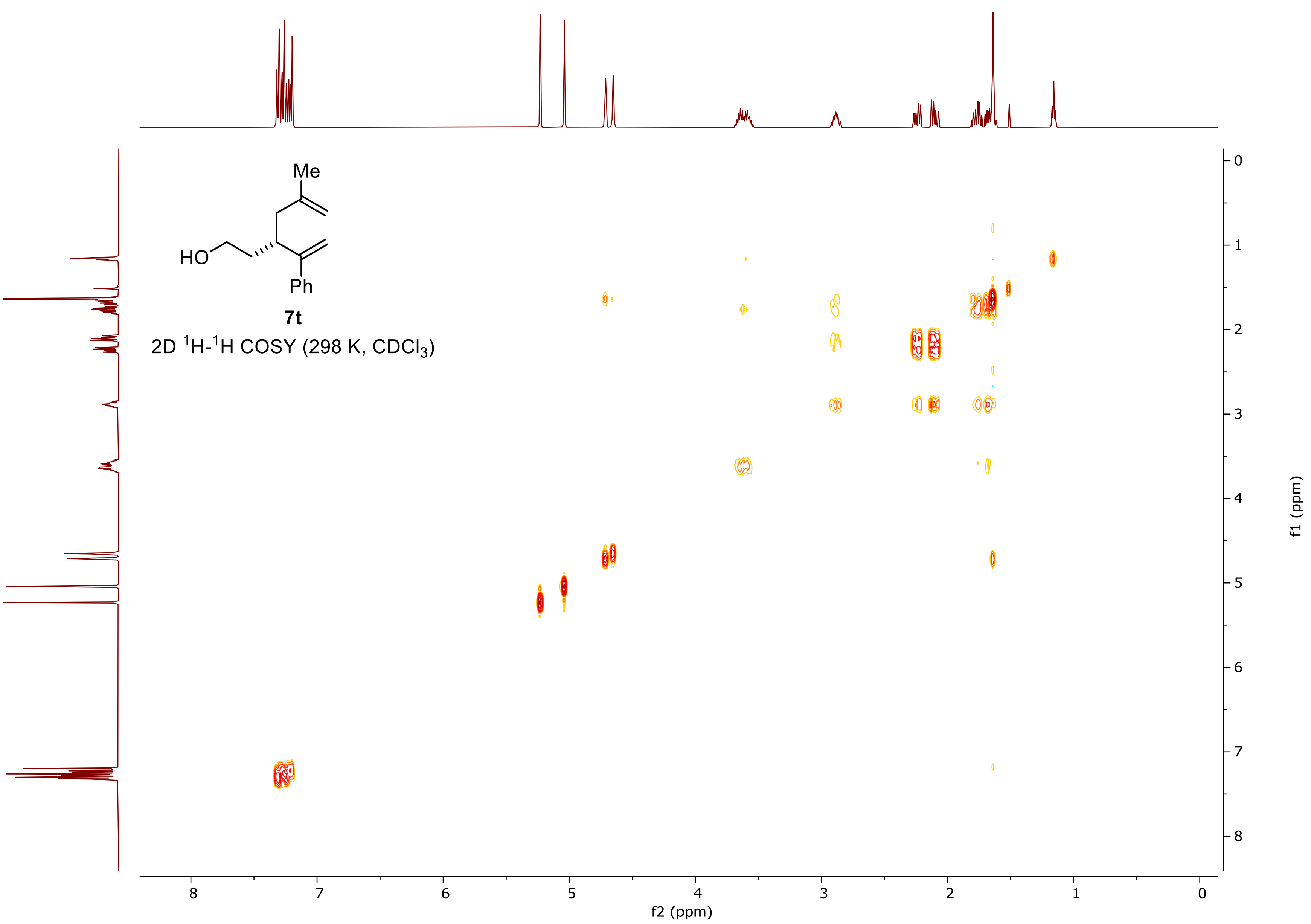

Me
HO
Ph
7t
2D ¹H-¹H COSY (298 K, CDCl₃)
f2 (ppm)
f1 (ppm)
8
7
6
5
4
3
2
1
0

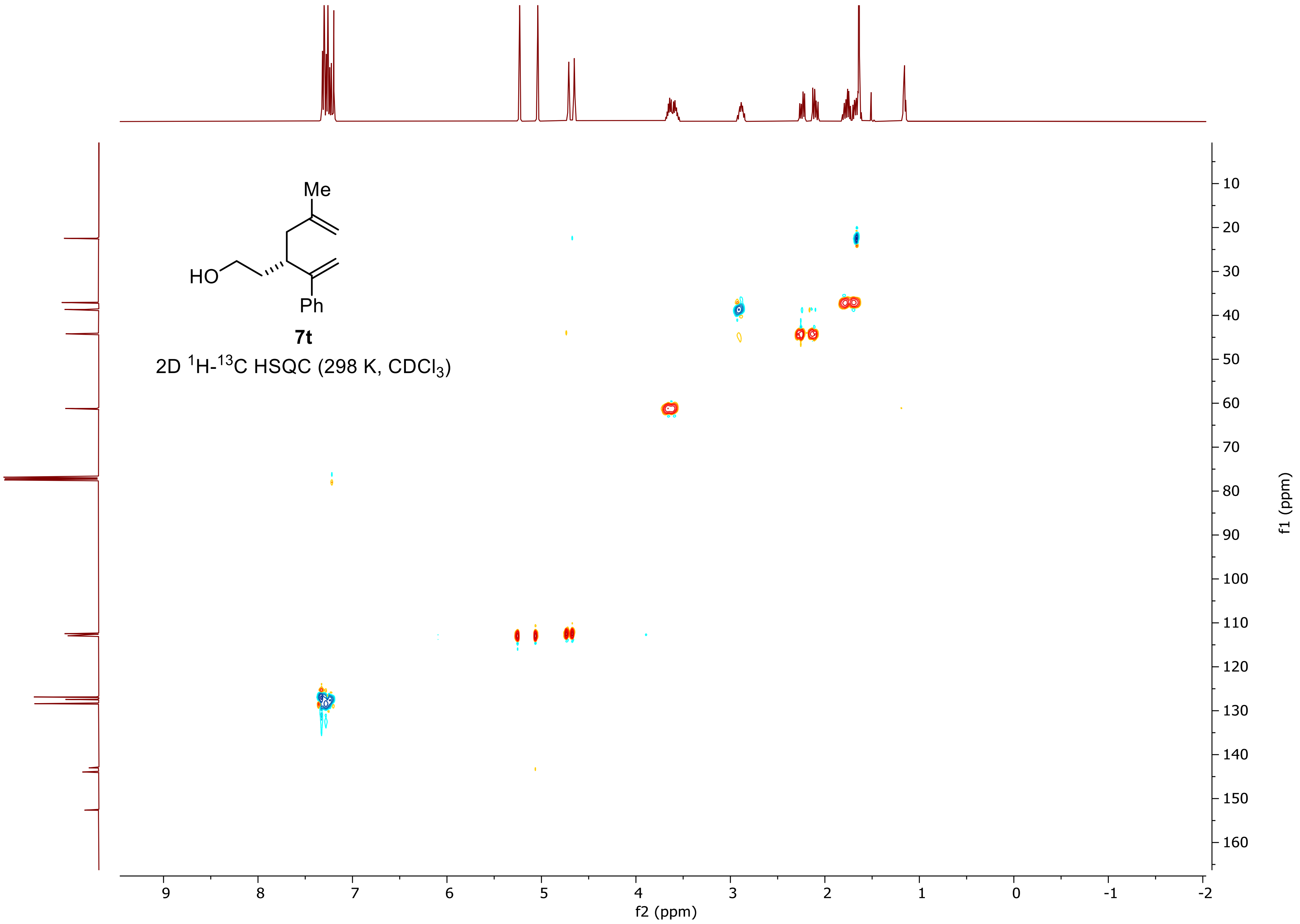
Me
HO
Ph
7t
2D 1H-13C HSQC (298 K, CDCl3)
f2 (ppm)
f1 (ppm)

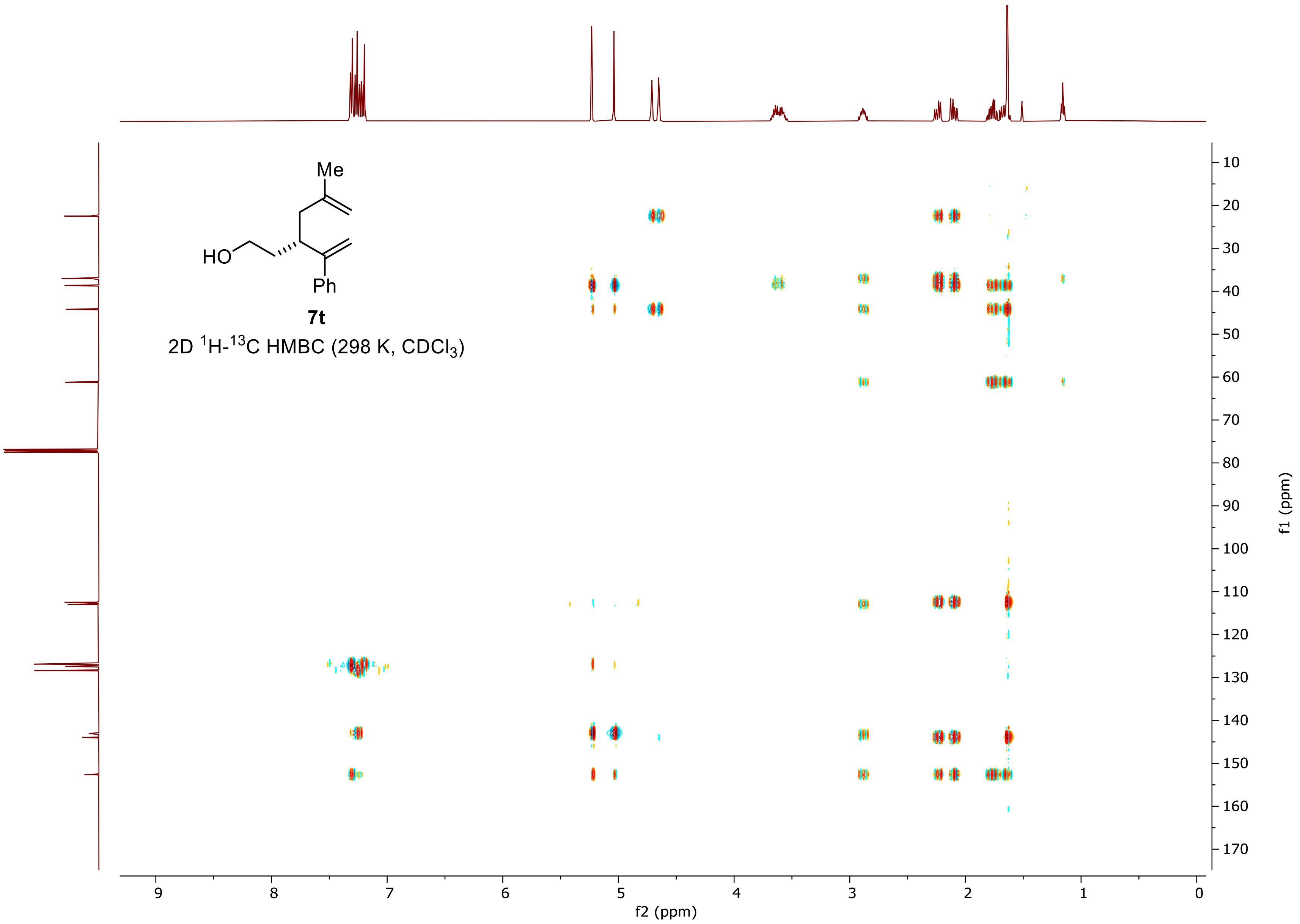
Me
HO
Ph
7t
2D ^{1}H-^{13}C HMBC (298 K, $CDCl_3$)
f2 (ppm)
f1 (ppm)
9
8
7
6
5
4
3
2
1
0
10
20
30
40
50
60
70
80
90
100
110
120
130
140
150
160
170

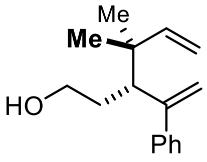

7u

^{1}H NMR (400 MHz, 298 K, $CDCl_3$)

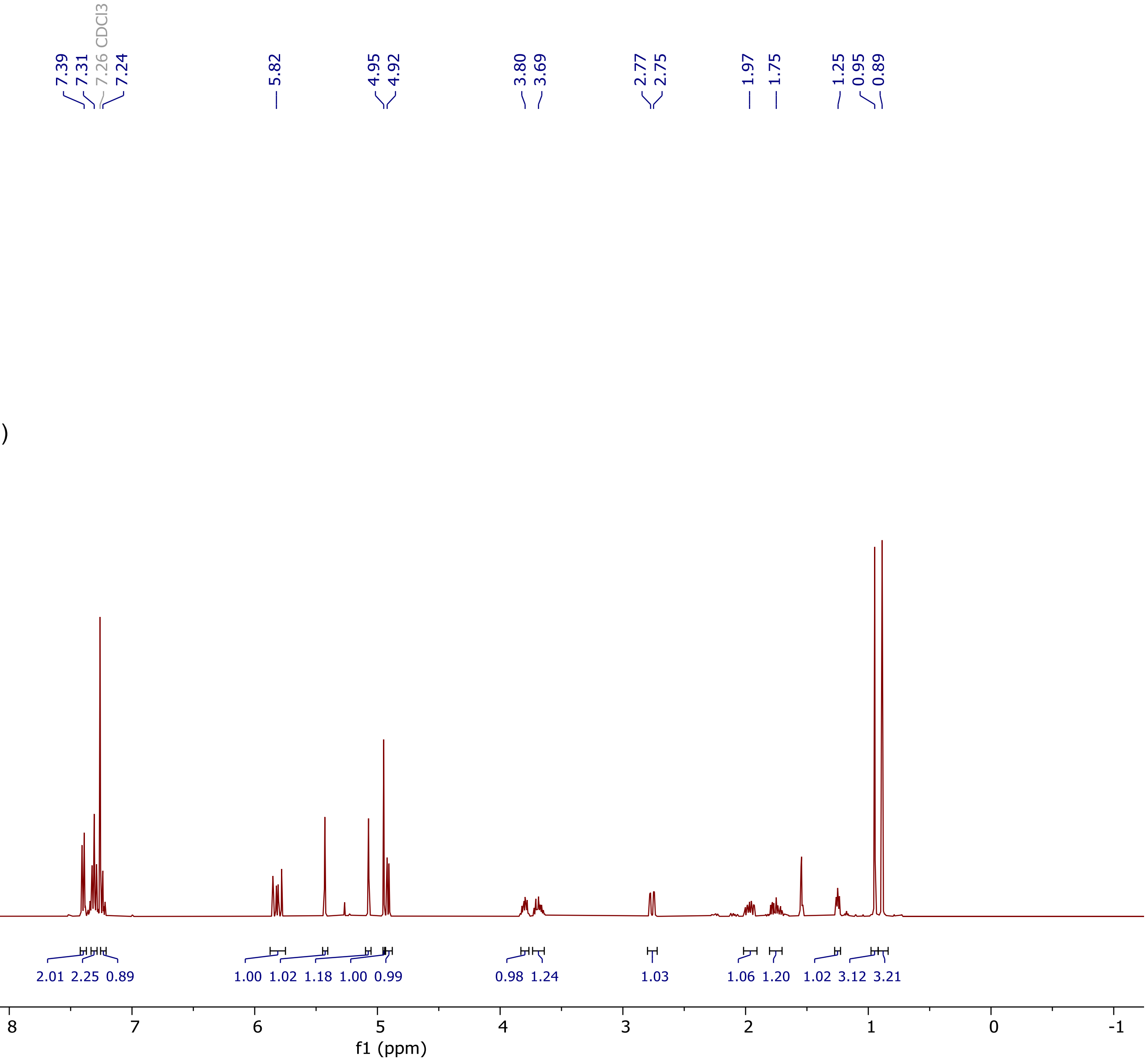

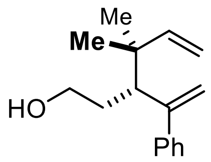

7u

$^{13}C\{^1H\}$ NMR (101 MHz, 298 K, $CDCl_3$)

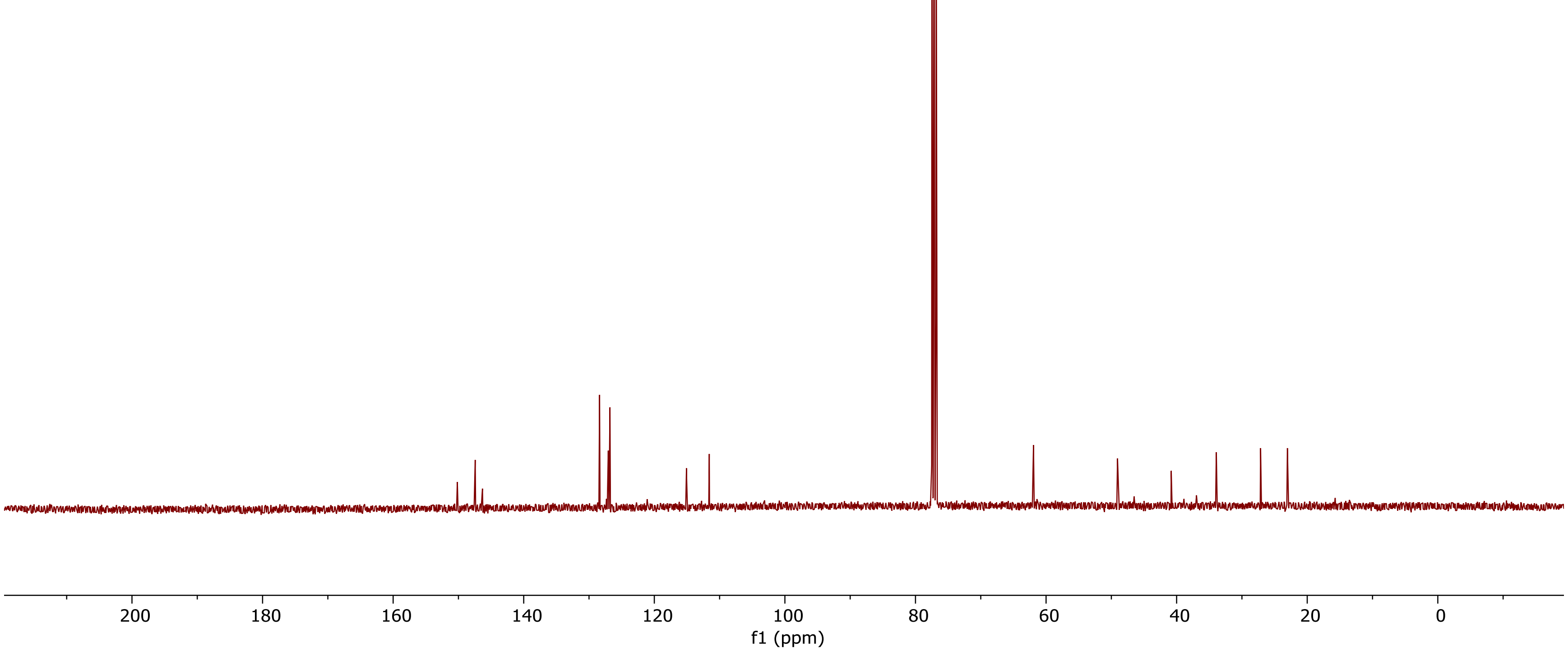

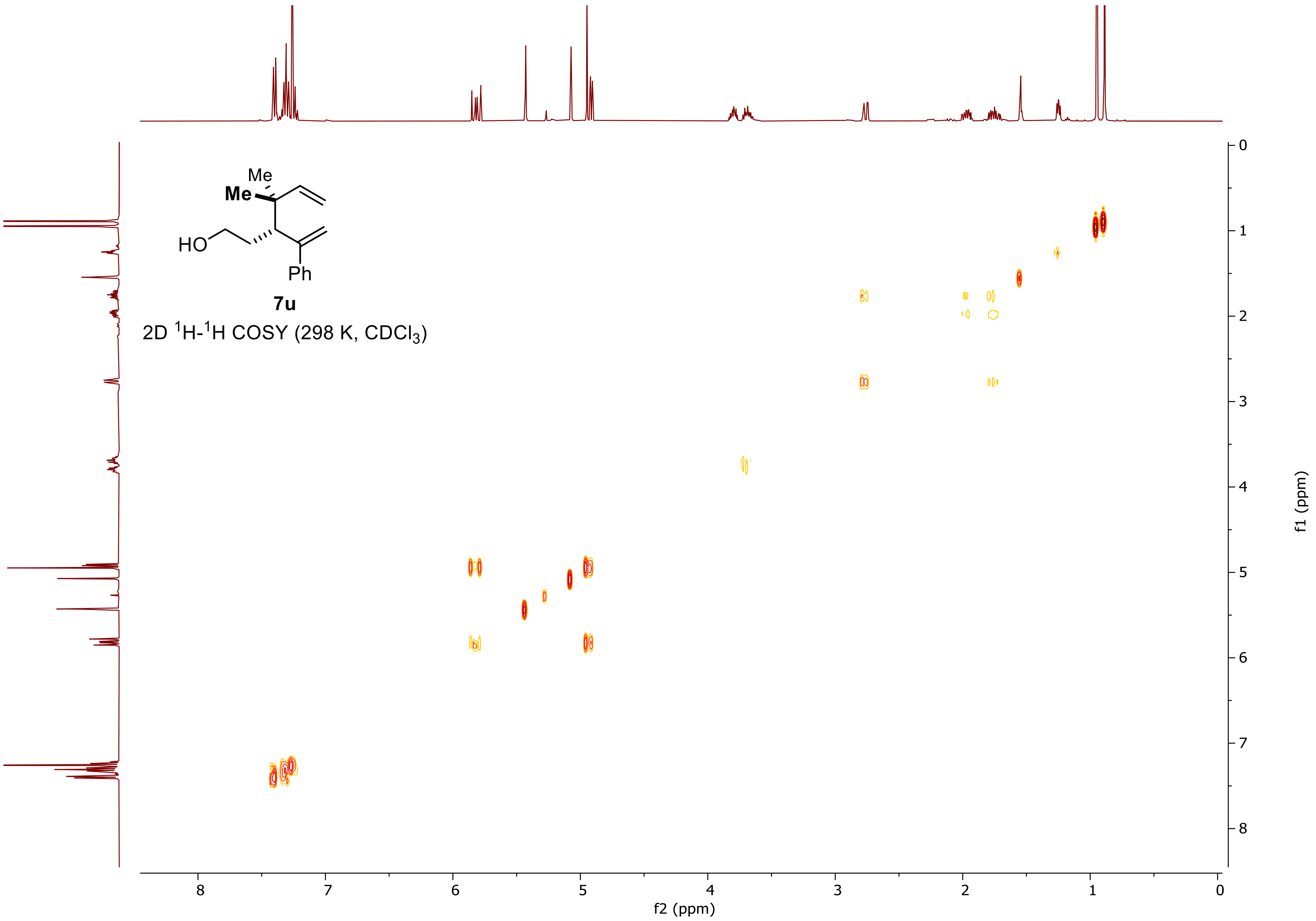
Me
Me
HO
Ph
7u
2D ¹H-¹H COSY (298 K, CDCl₃)
f2 (ppm)
f1 (ppm)
8
7
6
5
4
3
2
1
0

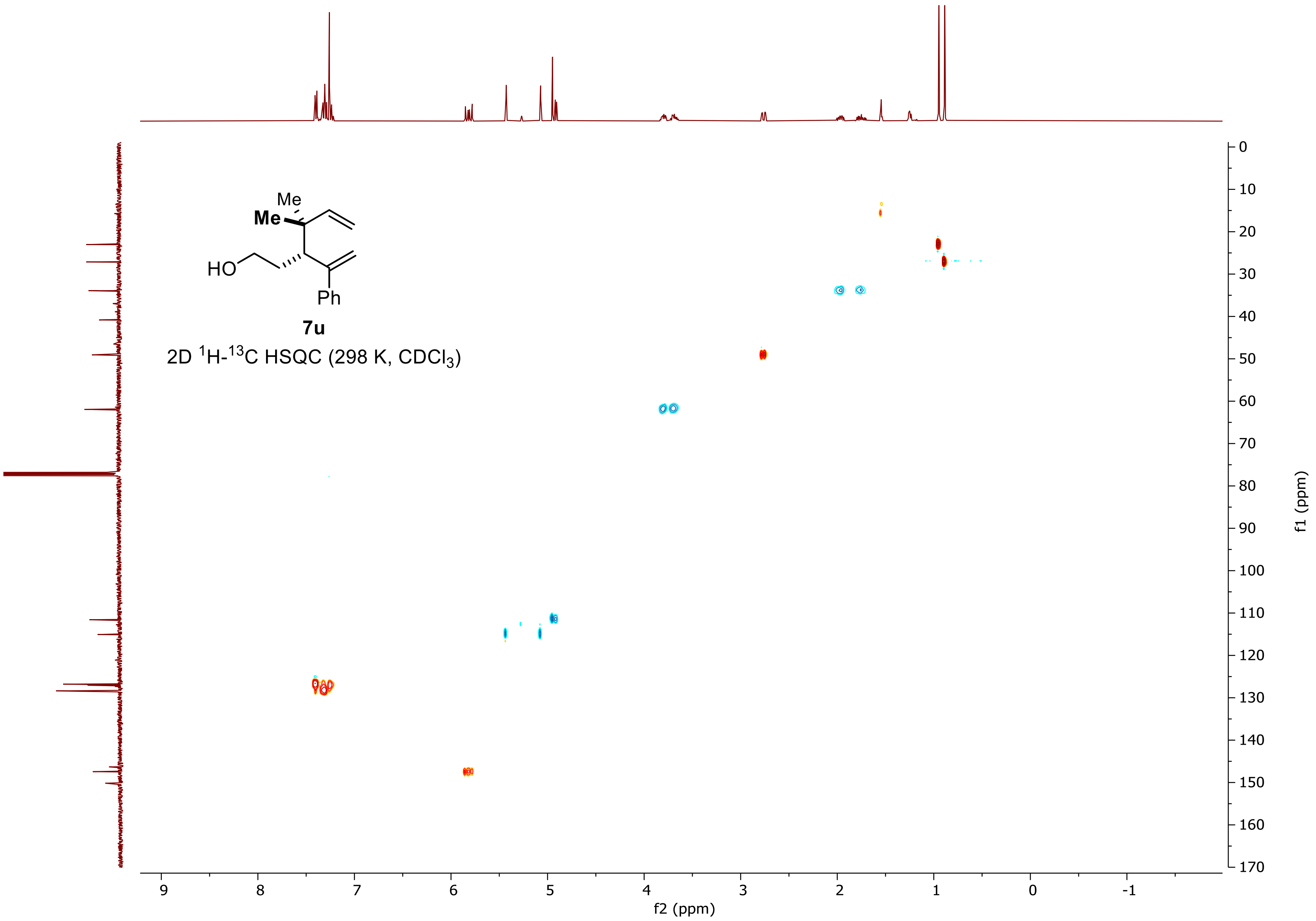

Me
Me
HO
Ph
7u
2D ^{1}H-^{13}C HSQC (298 K, CDCl$_3$)
f2 (ppm)
f1 (ppm)

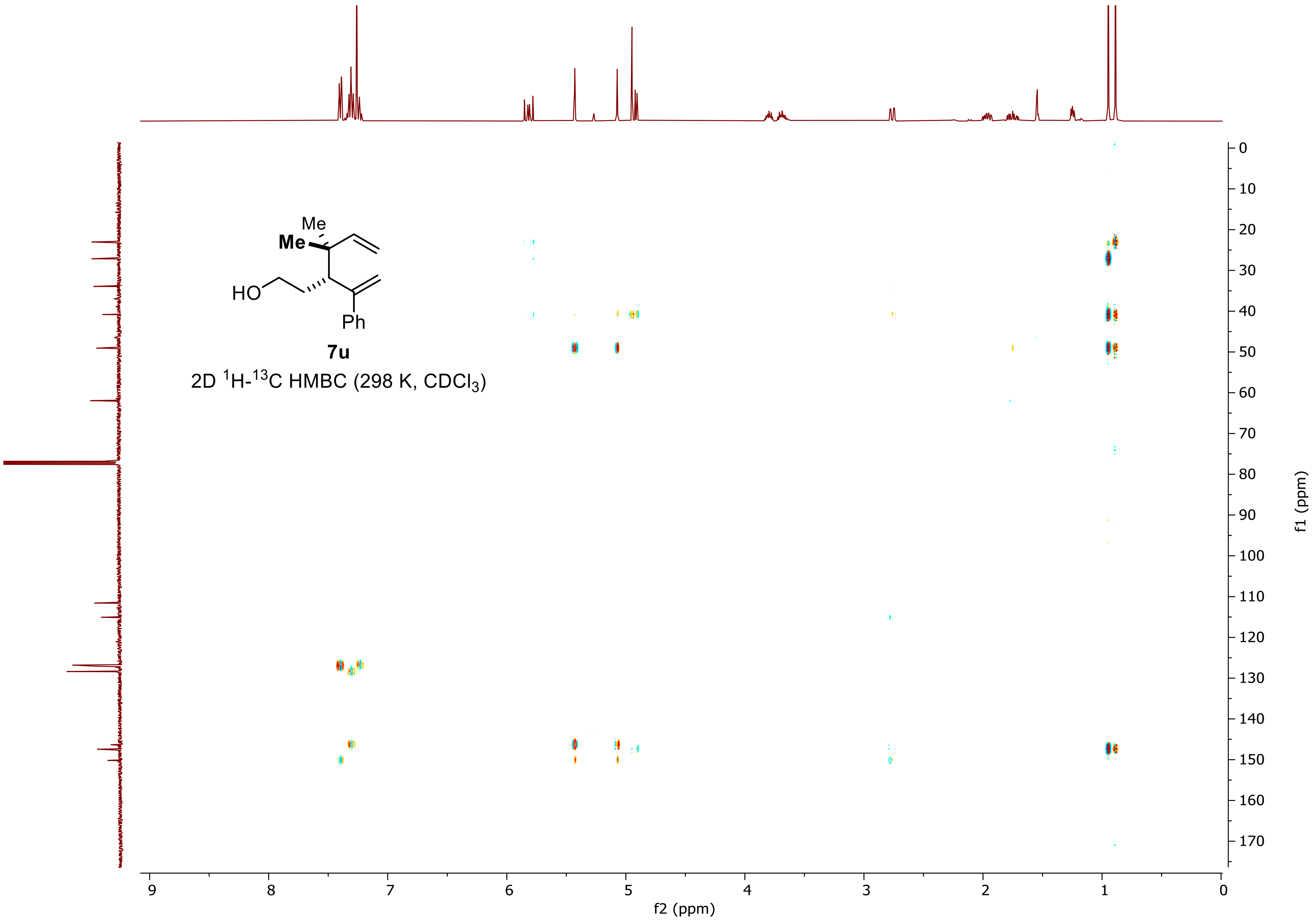
Me
Me
HO
Ph
7u
2D ¹H-¹³C HMBC (298 K, CDCl₃)
f1 (ppm)
f2 (ppm)

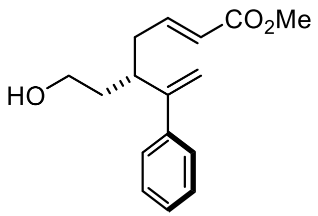

8a

^{1}H NMR (400 MHz, 298 K, $CDCl_3$)

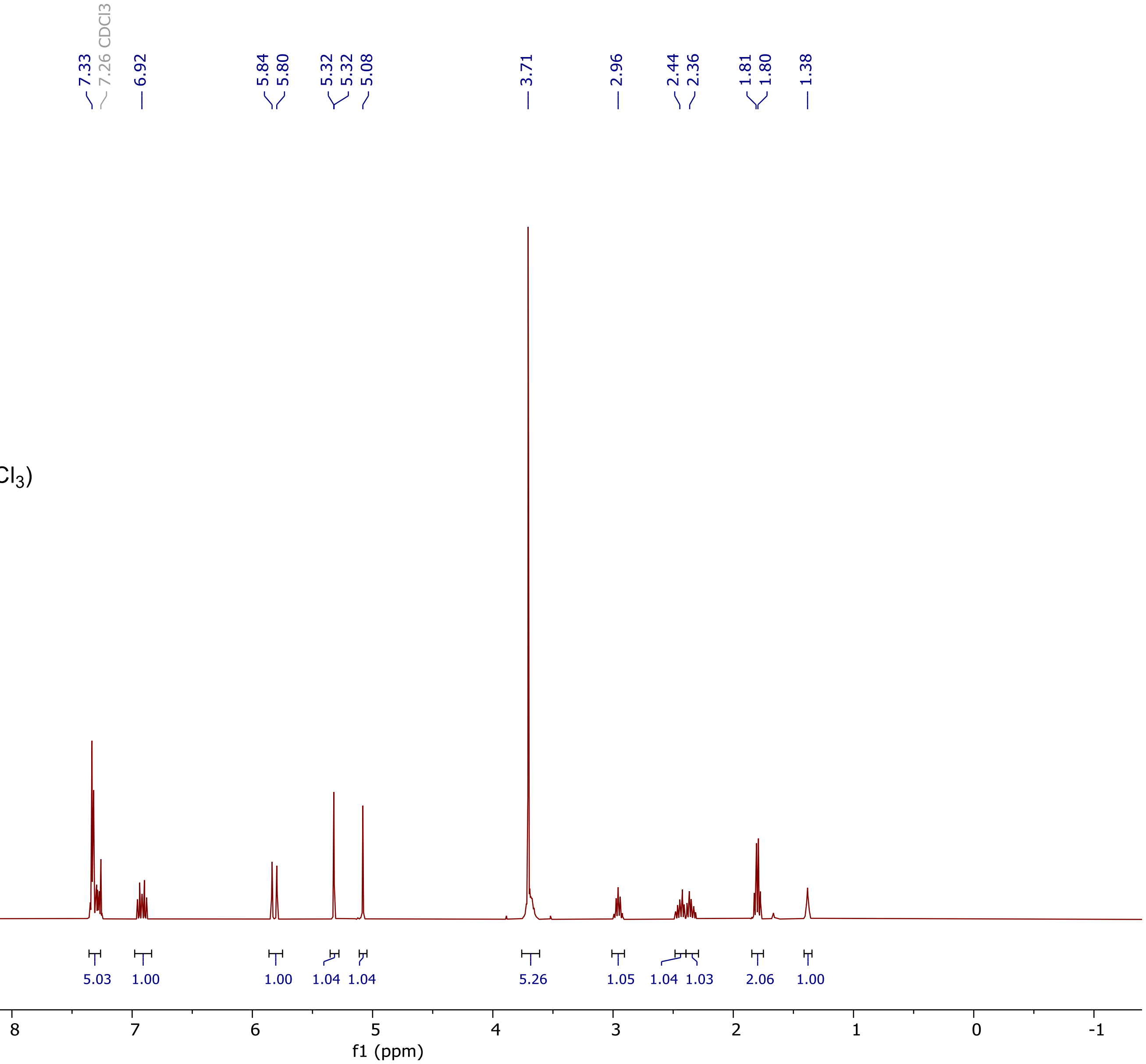

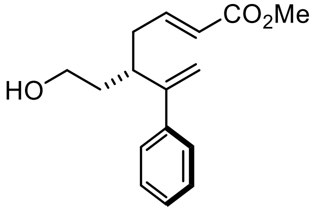

8a

$^{13}C\ \{^{1}H\}$ NMR (101 MHz, 298 K, $CDCl_3$)

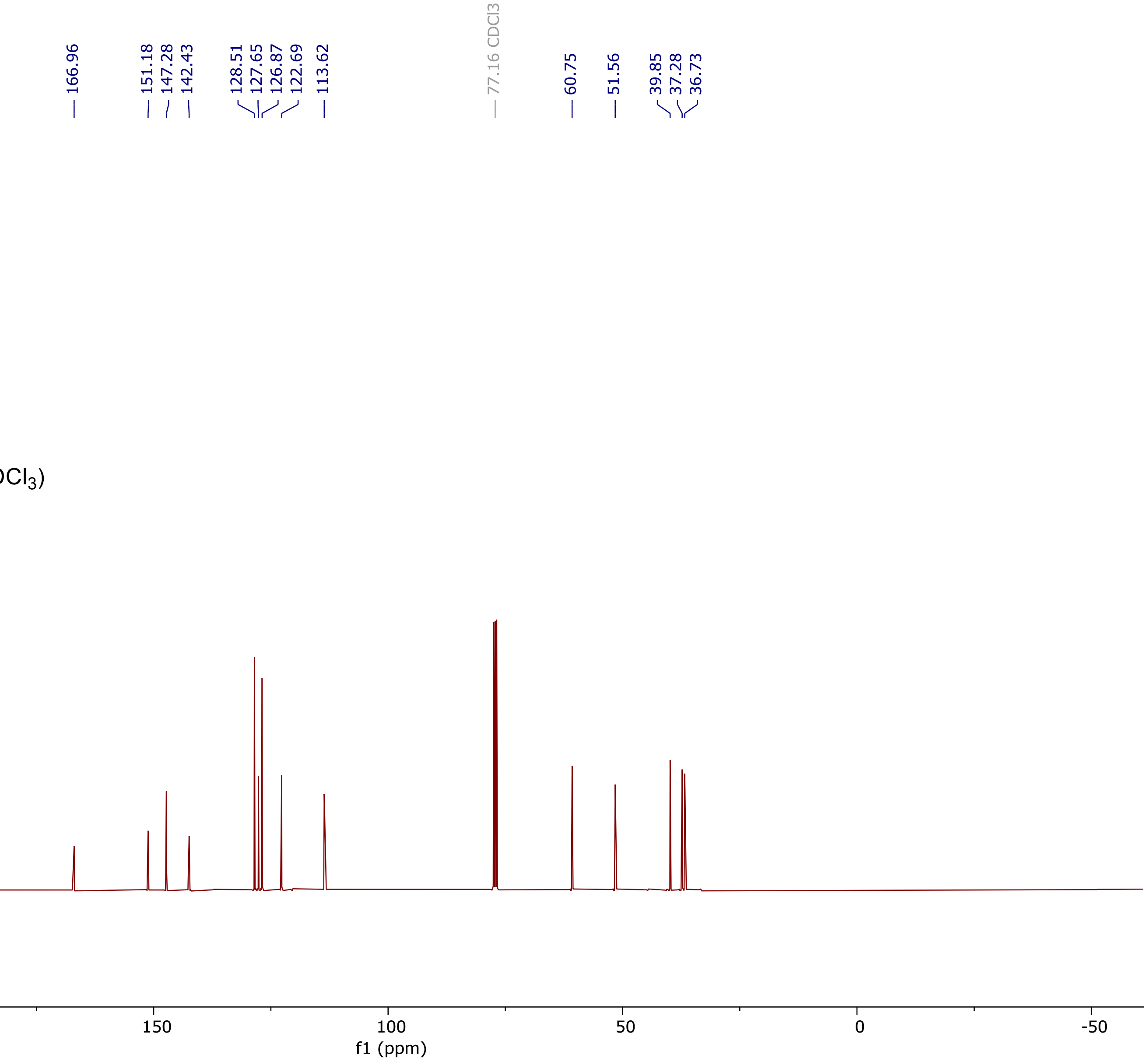

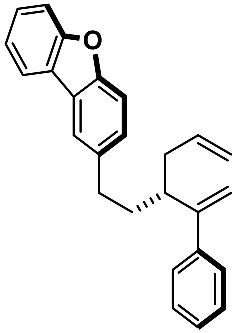

9a

^{1}H NMR (400 MHz, 298 K, $CDCl_3$)

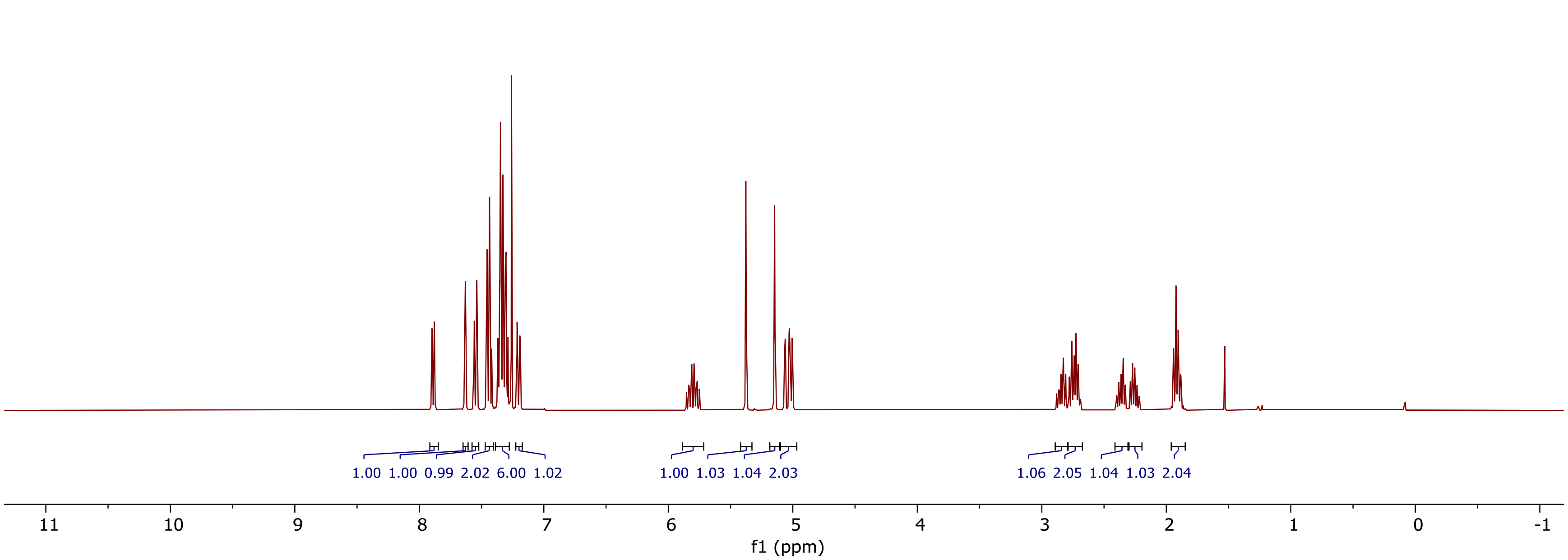

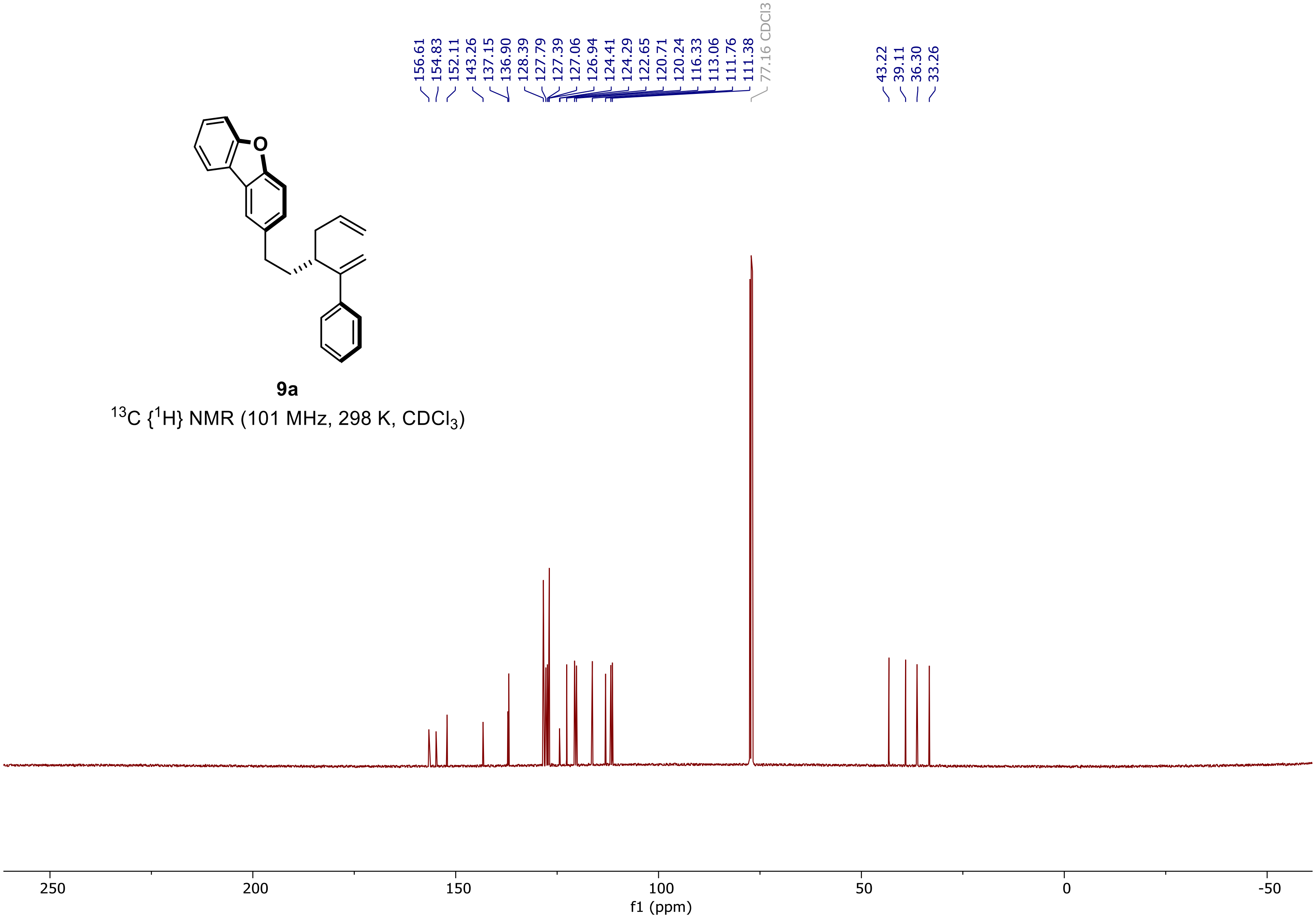

9a

$^{13}C\ \{^{1}H\}$ NMR (101 MHz, 298 K, $CDCl_3$)

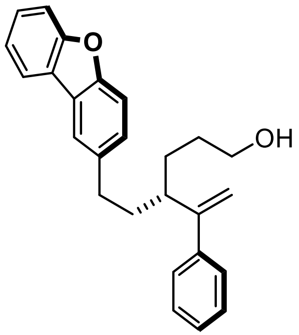

10a

^{1}H NMR (400 MHz, 298 K, $CDCl_3$)

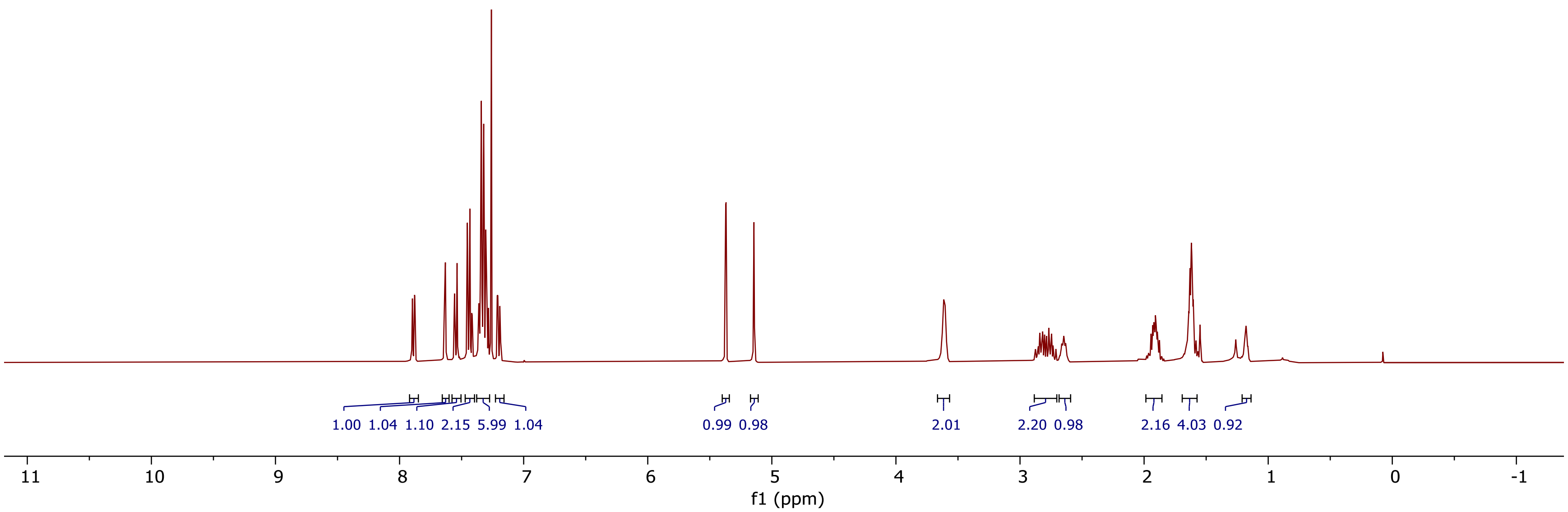

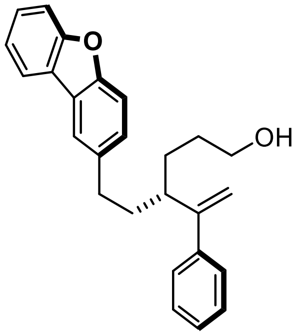

10a

$^{13}C\ \{^{1}H\}$ NMR (101 MHz, 298 K, $CDCl_3$)

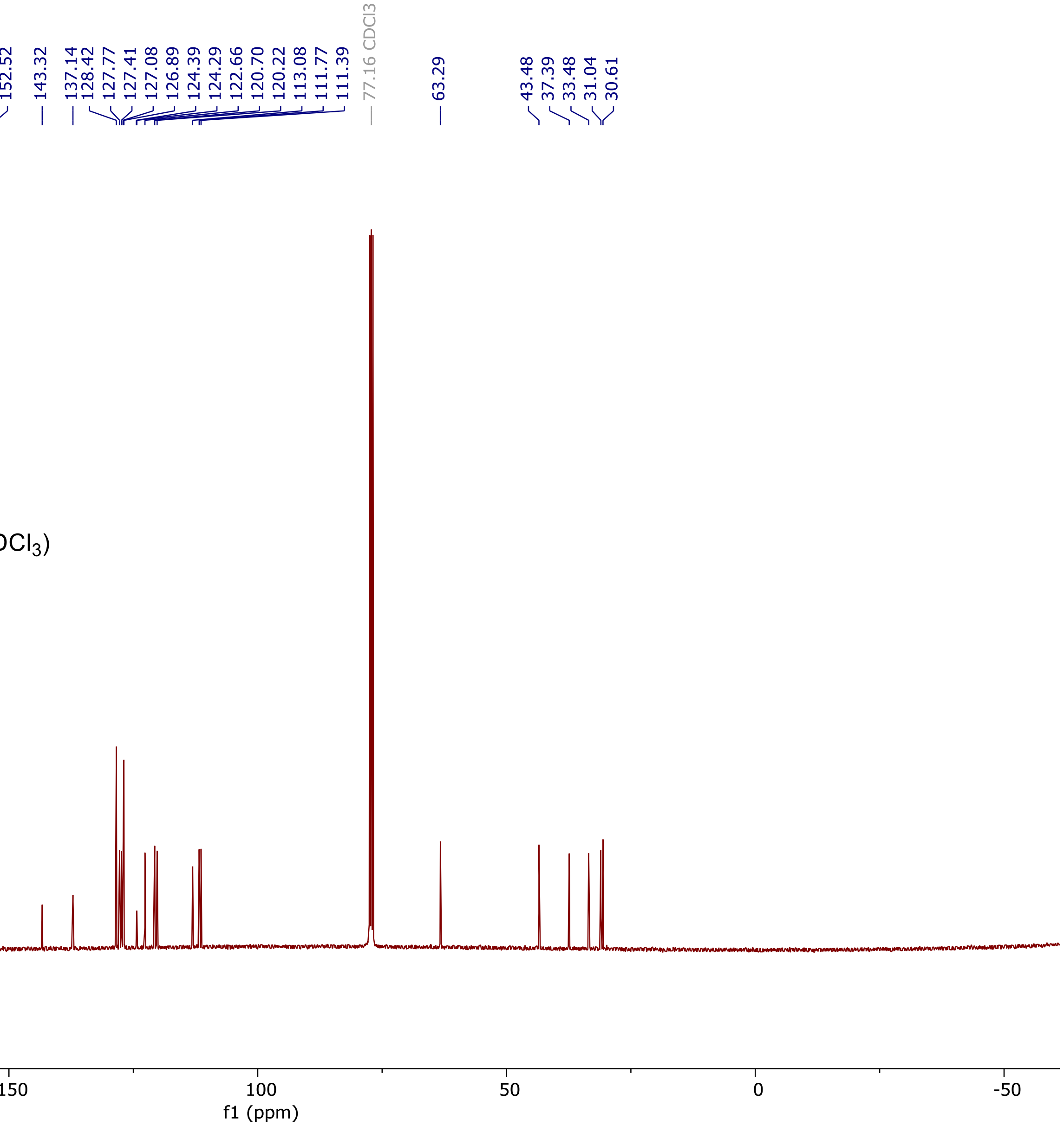

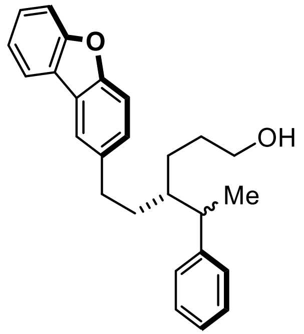

11a

^1H NMR (400 MHz, 298 K, $CDCl_3$)

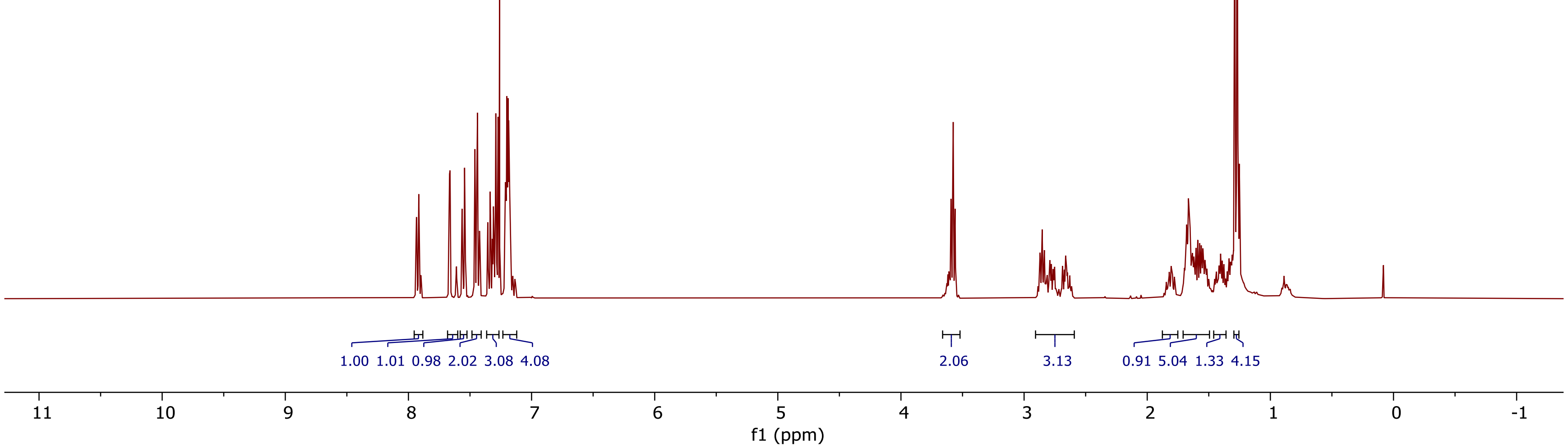

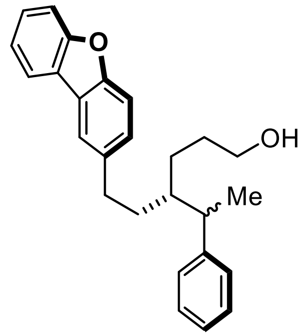

11a

$^{13}C\{^1H\}$ NMR (101 MHz, 298 K, $CDCl_3$)

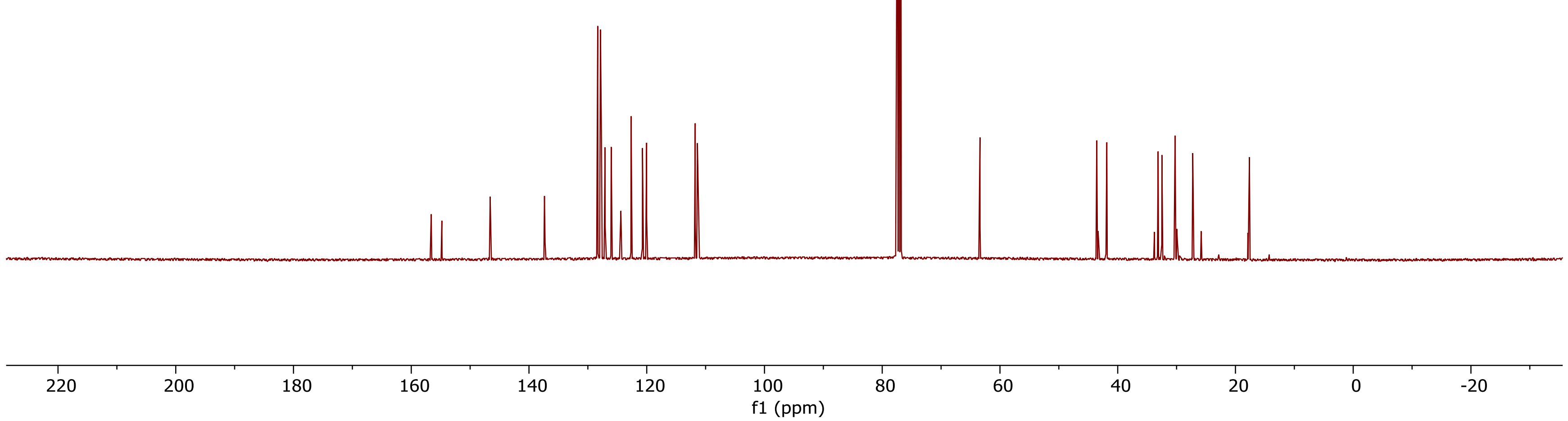

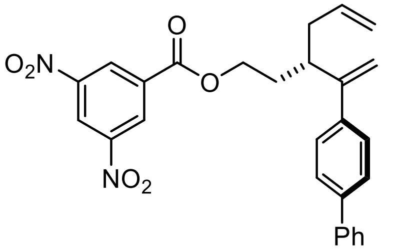

12a

^1H NMR (400 MHz, 298 K, $CDCl_3$)

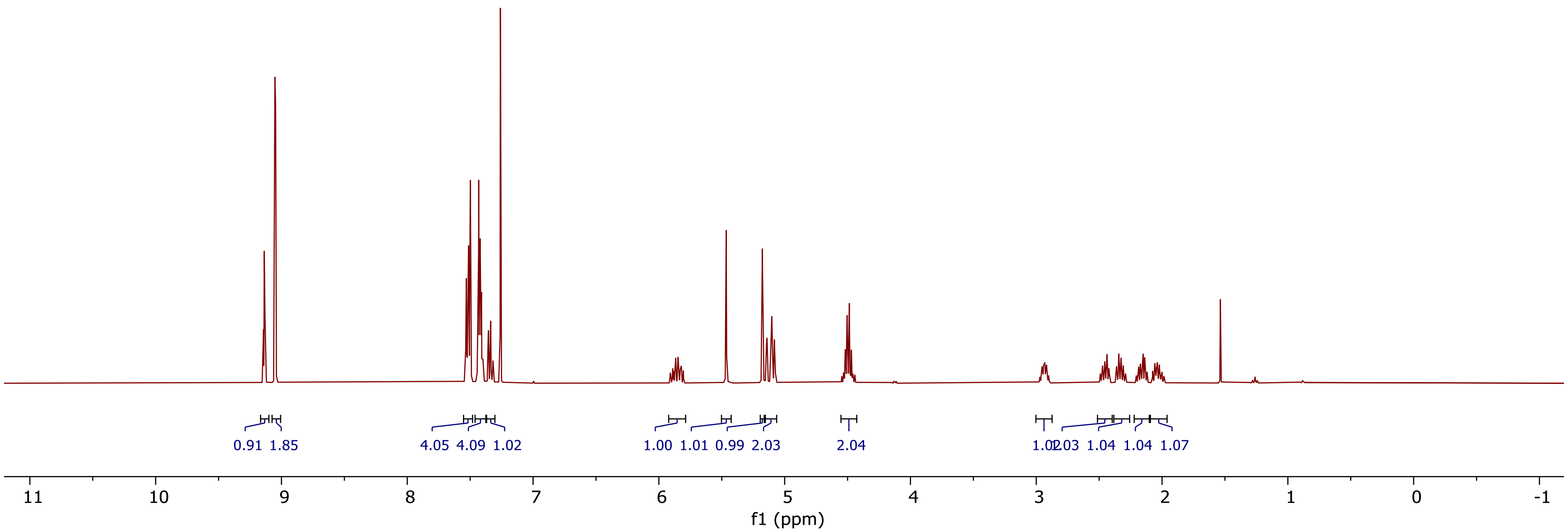

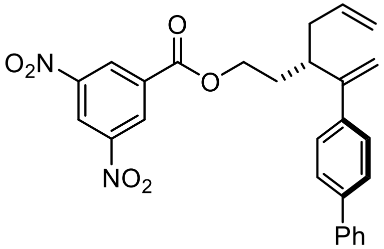

12a

$^{13}C\{^{1}H\}$ NMR (101 MHz, 298 K, $CDCl_3$)

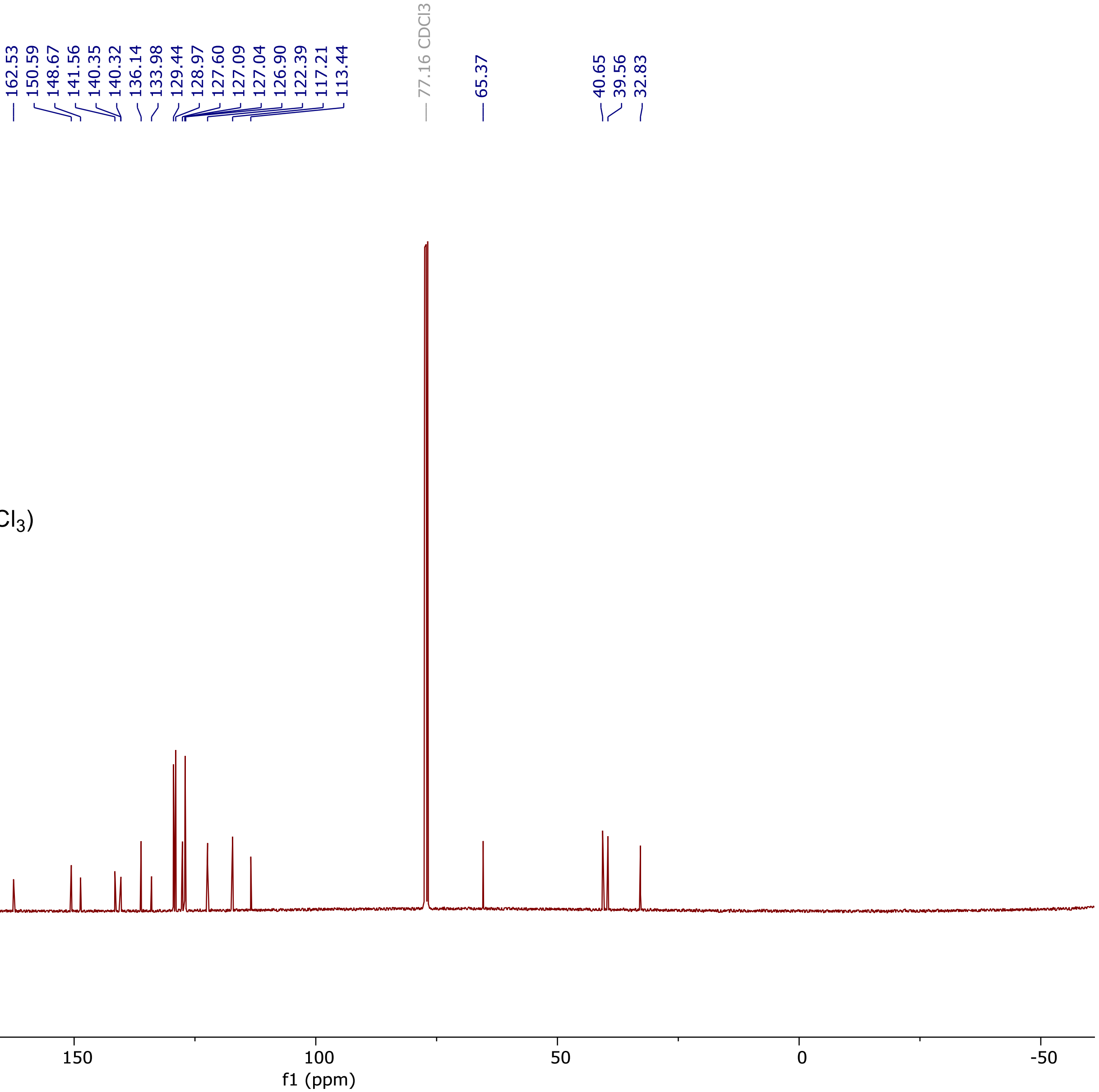

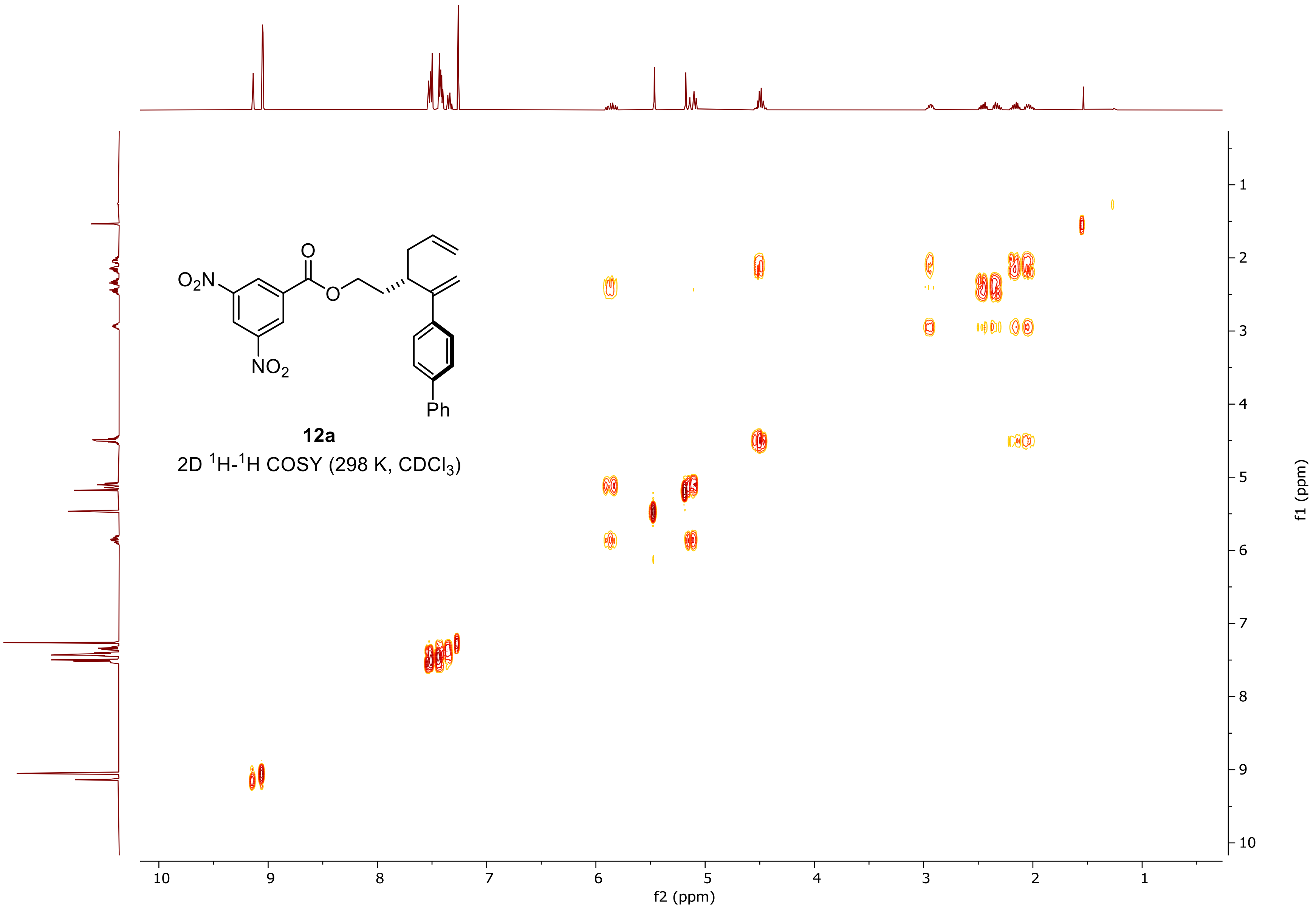

12a

2D ^{1}H-^{1}H COSY (298 K, $CDCl_3$)

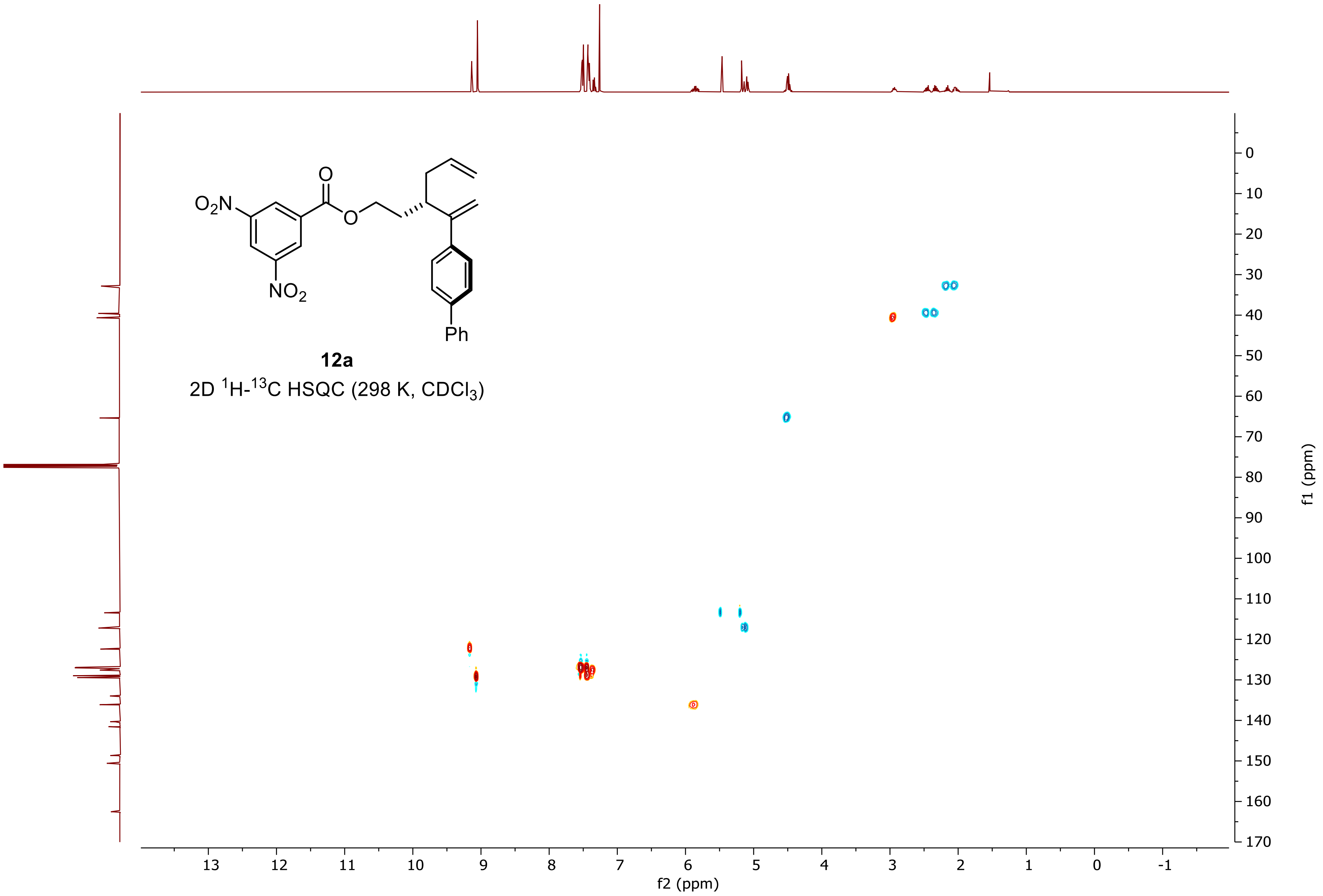
O
O_2N
O
NO_2
Ph
12a
2D ^1H-^{13}C HSQC (298 K, $CDCl_3$)
f2 (ppm)
f1 (ppm)

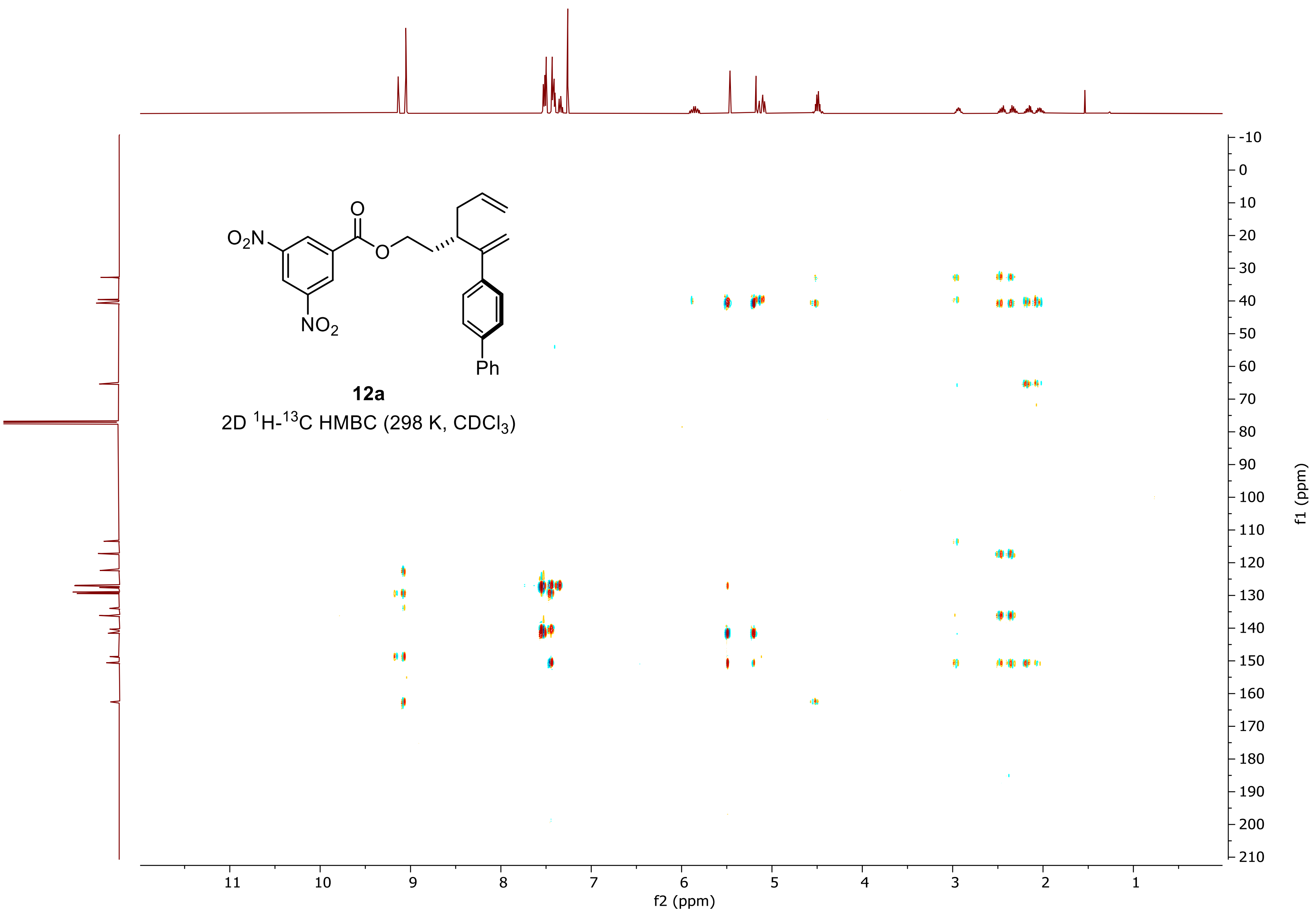

12a

2D ^{1}H-^{13}C HMBC (298 K, $CDCl_3$)

11. NMR spectra for known compounds

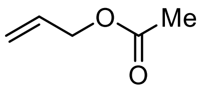

2a

^{1}H NMR (400 MHz, 298 K, $CDCl_3$)

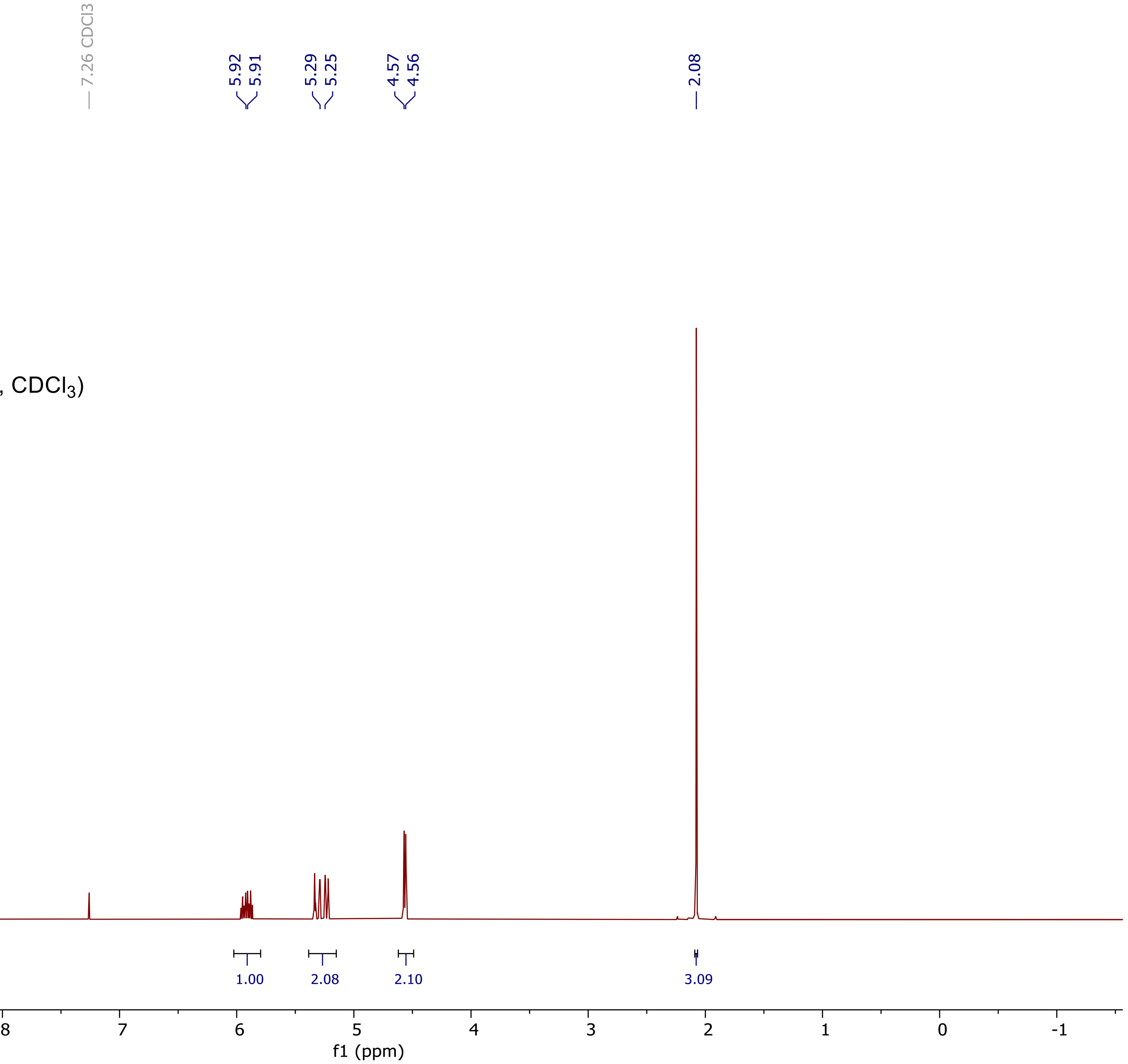

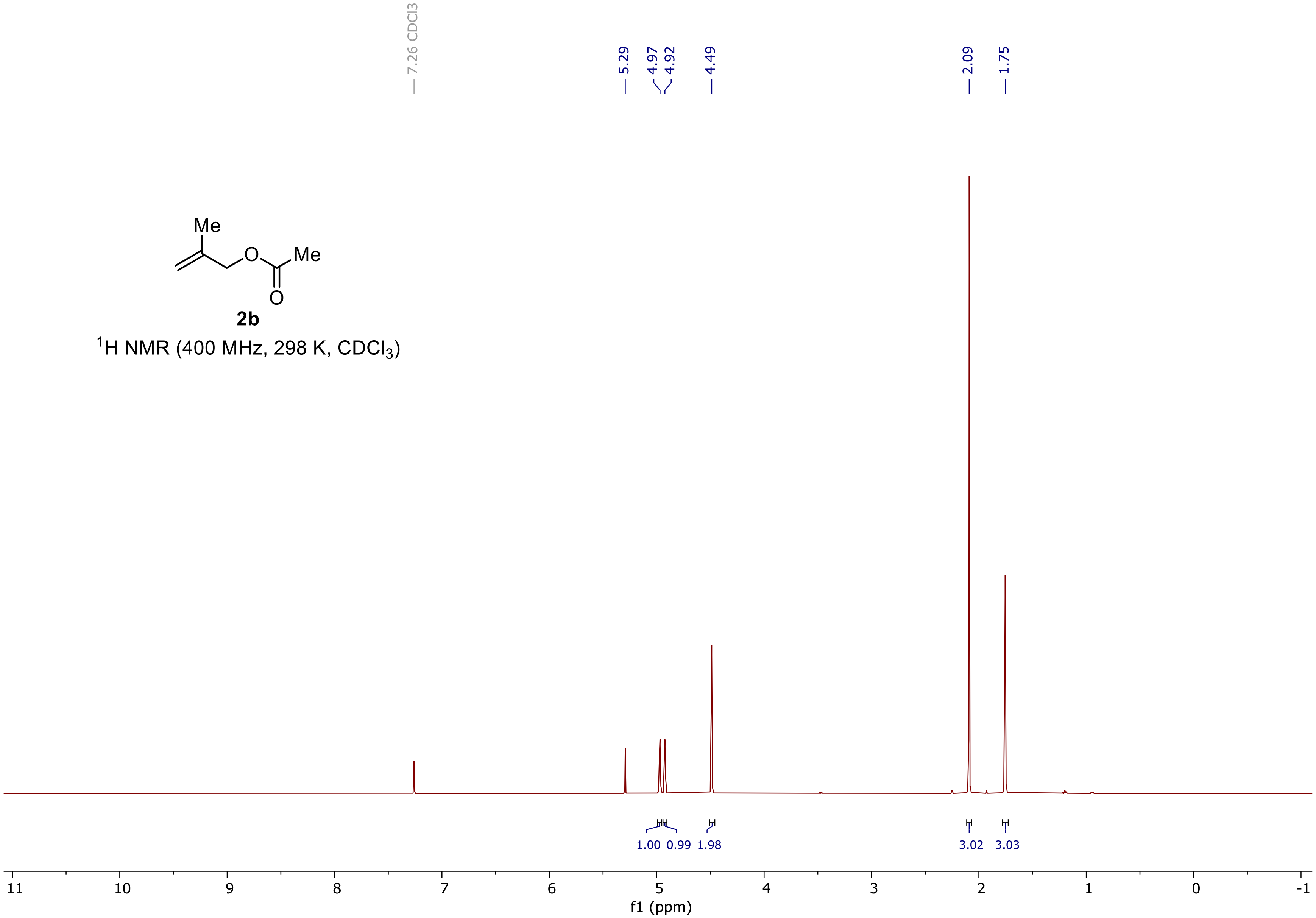

2b

^{1}H NMR (400 MHz, 298 K, $CDCl_3$)

2c

^{1}H NMR (400 MHz, 298 K, $CDCl_3$)

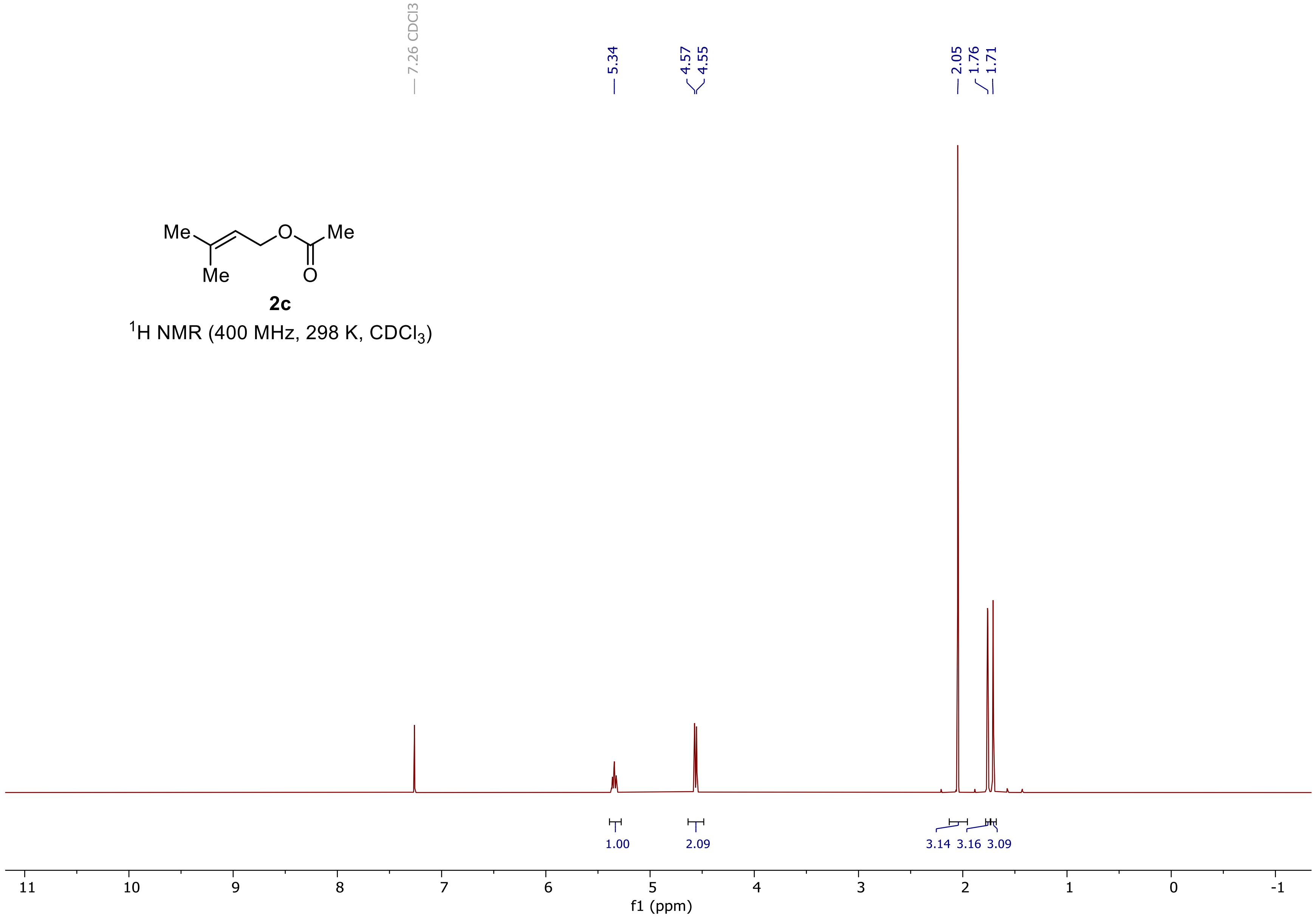

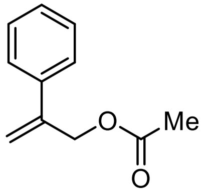

2d

^{1}H NMR (400 MHz, 298 K, $CDCl_3$)

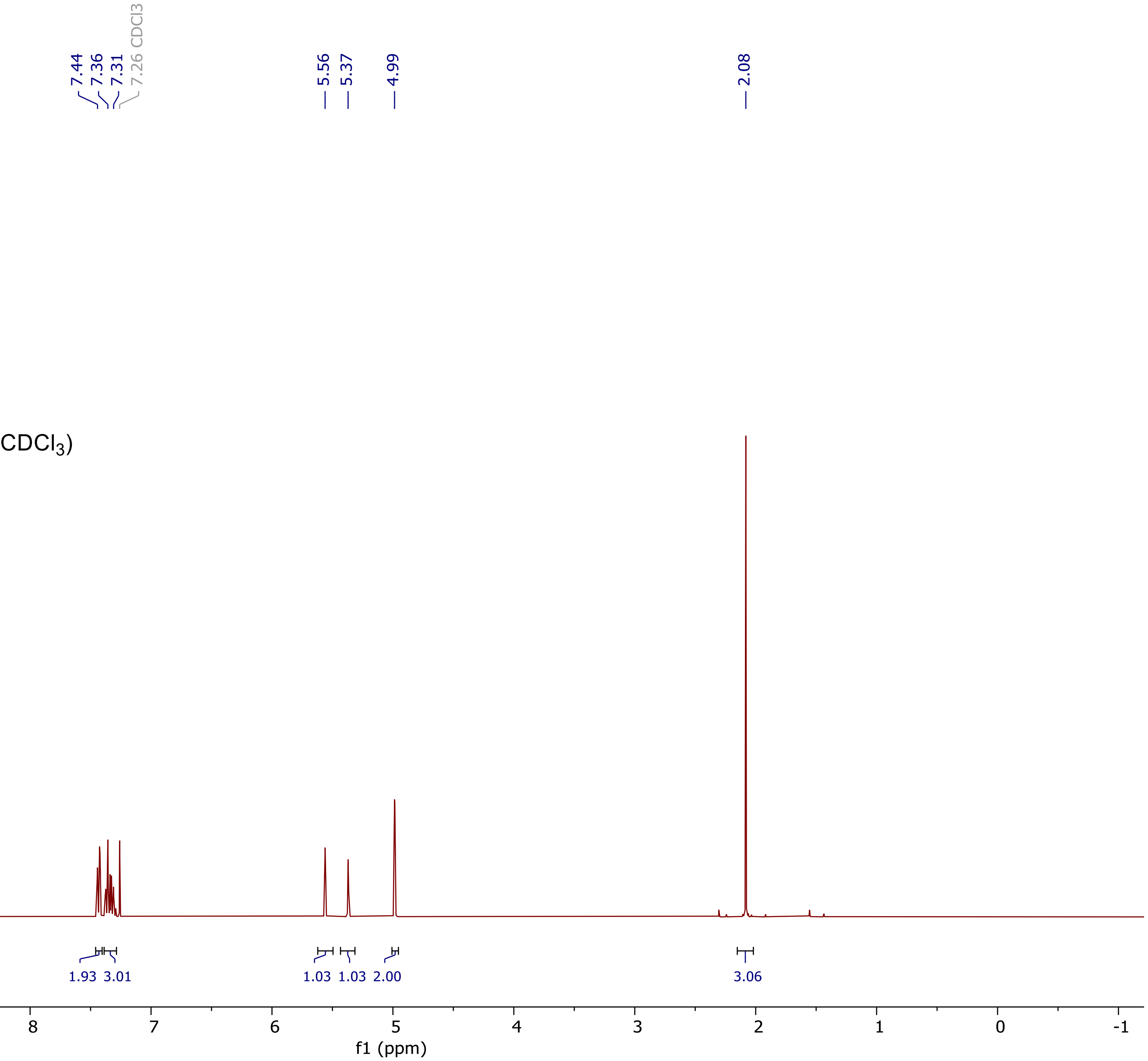

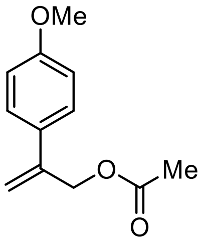

2e

^{1}H NMR (400 MHz, 298 K, $CDCl_3$)

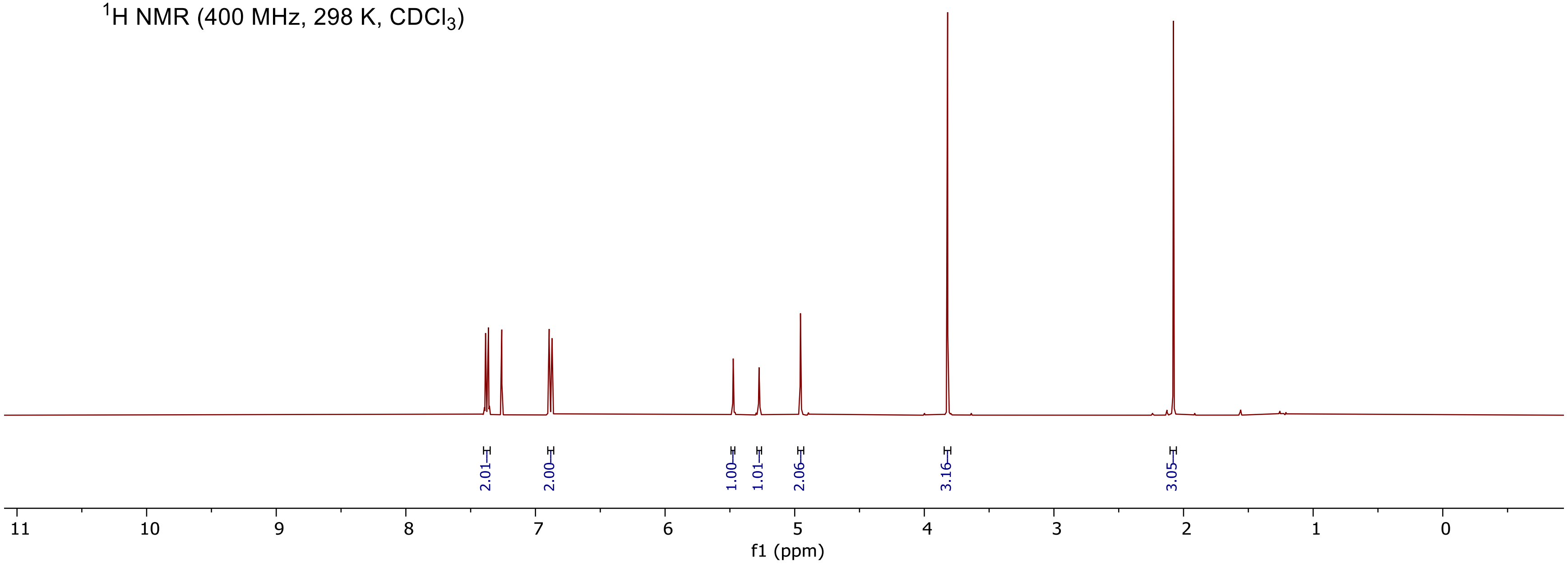

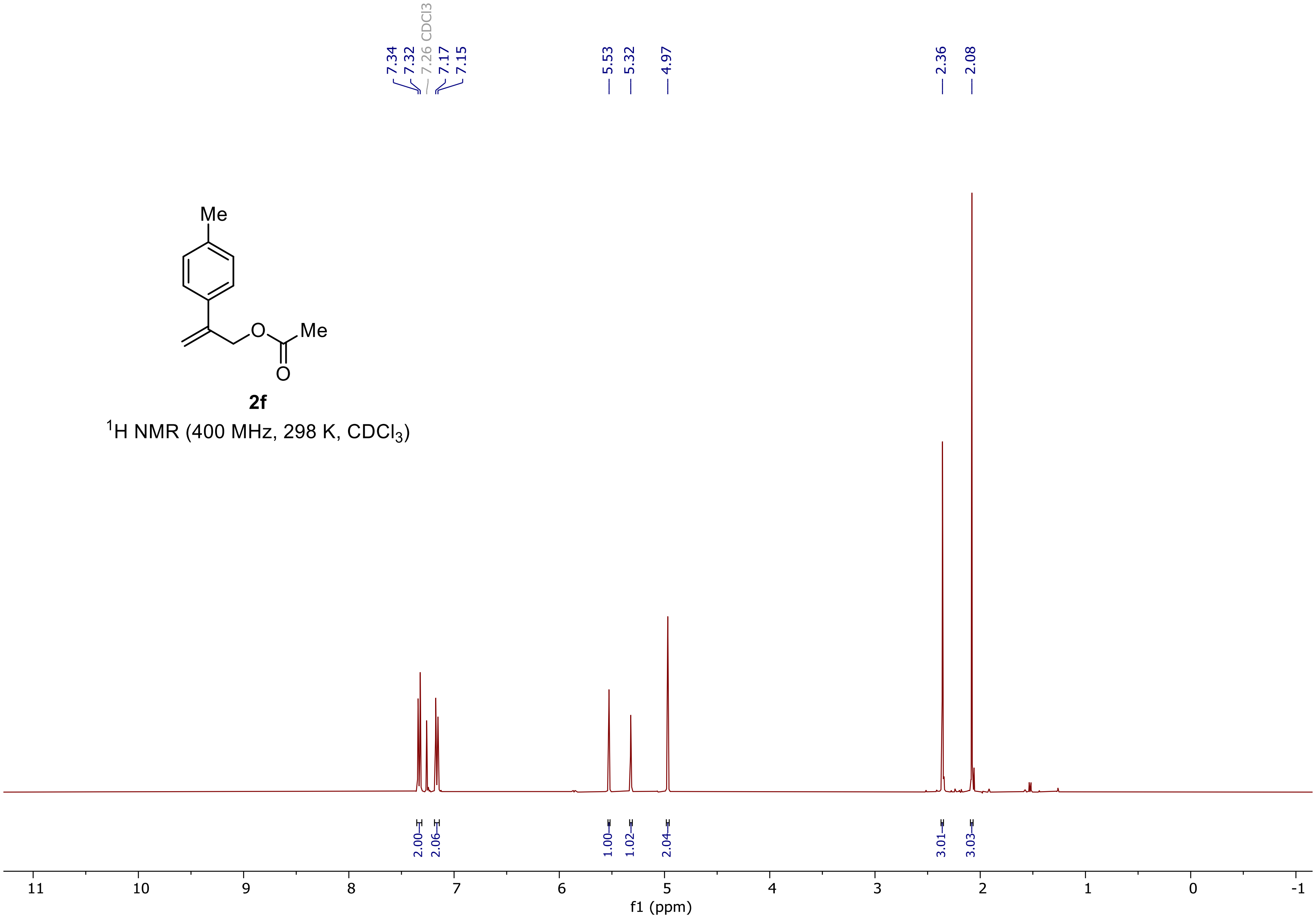

2f

^{1}H NMR (400 MHz, 298 K, $CDCl_3$)

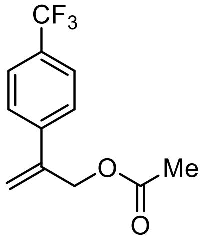

2g

^{1}H NMR (400 MHz, 298 K, $CDCl_3$)

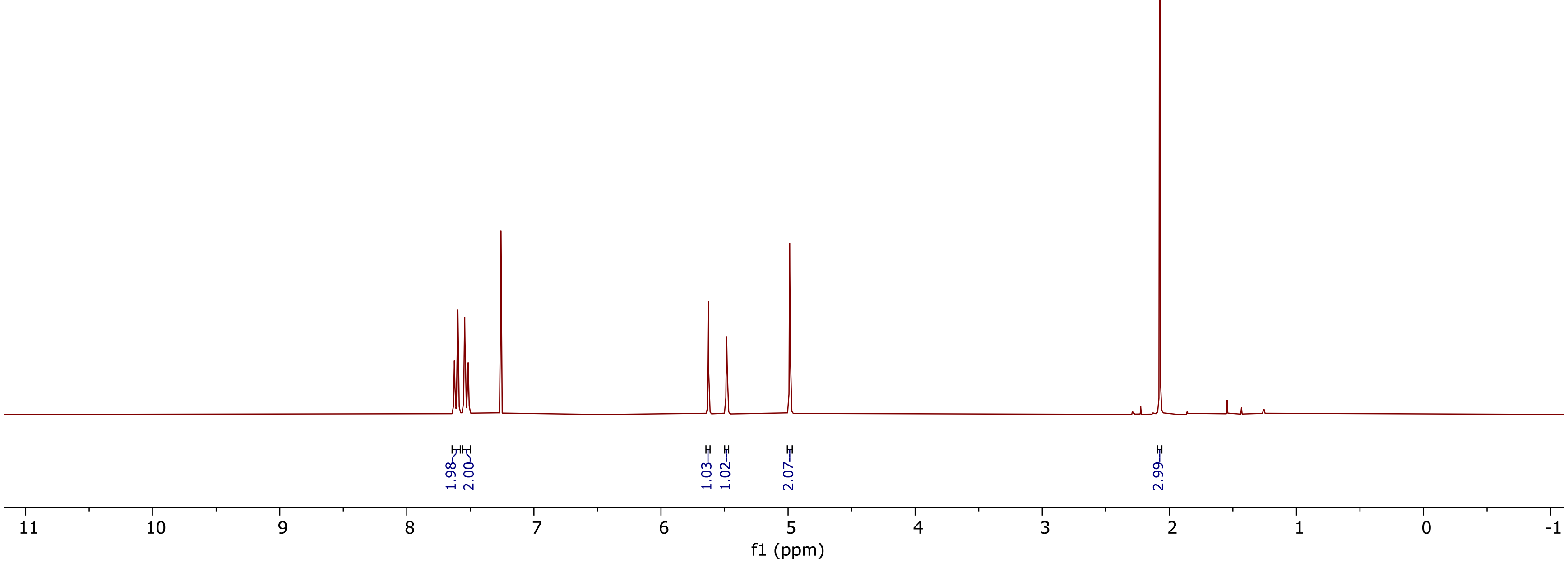

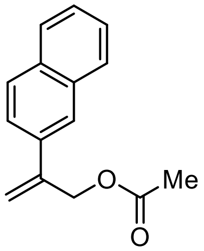

2h

^{1}H NMR (400 MHz, 298 K, $CDCl_3$)

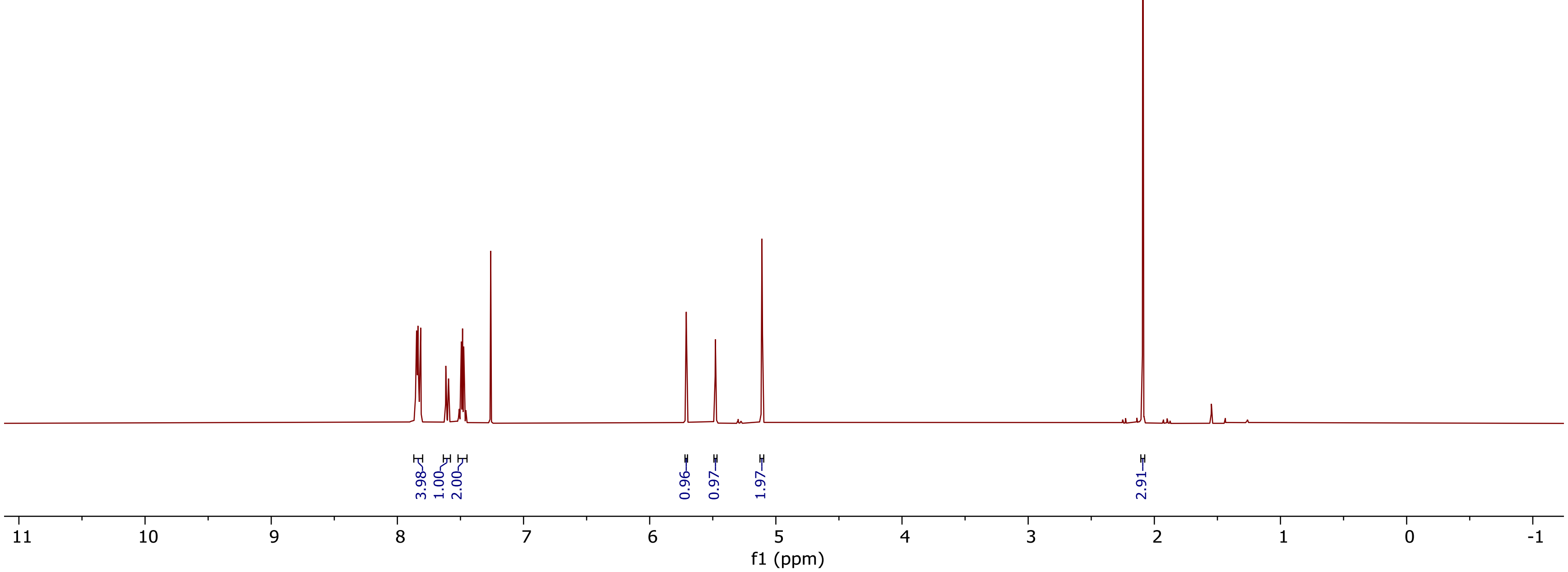

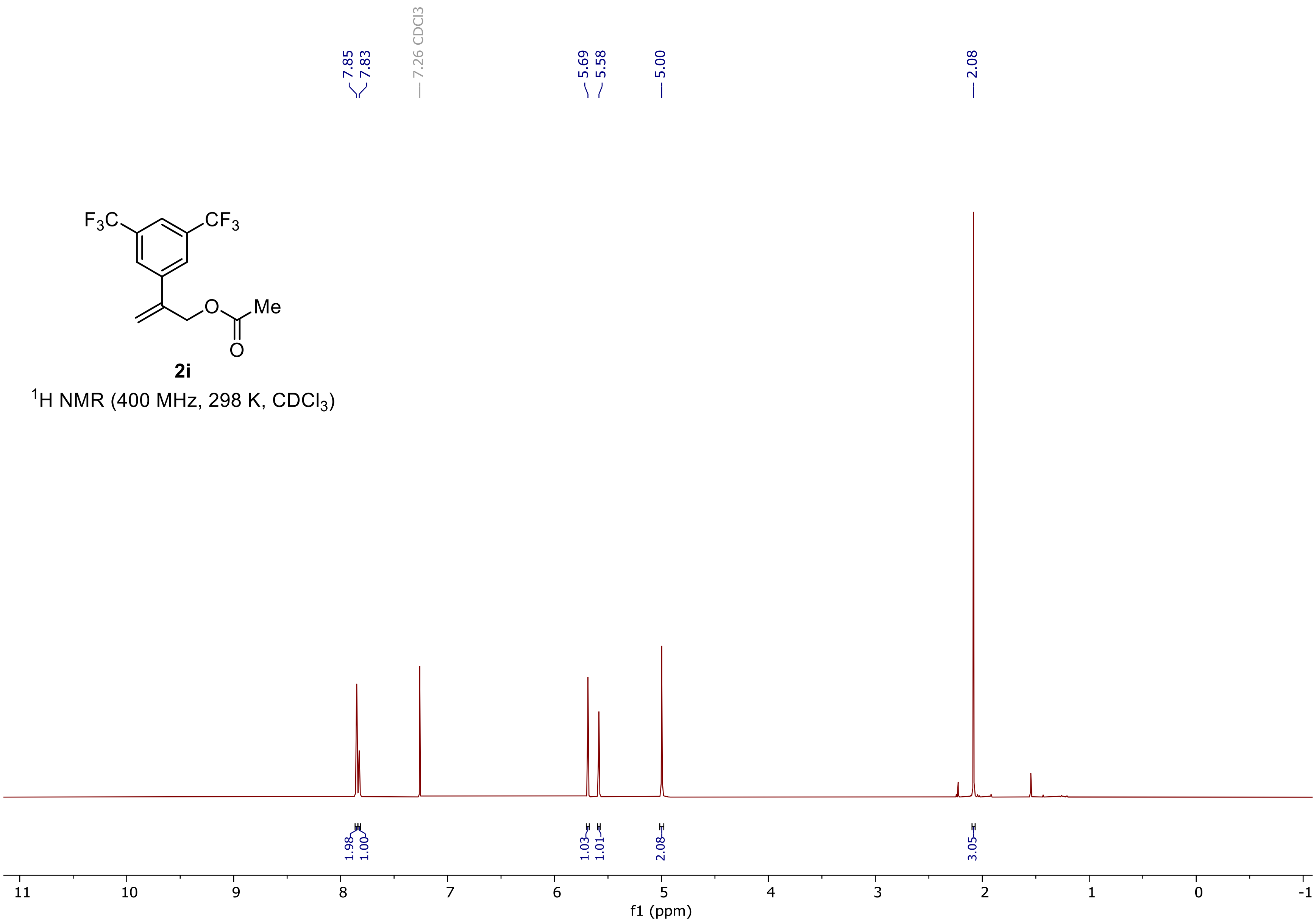

2i

^{1}H NMR (400 MHz, 298 K, $CDCl_3$)

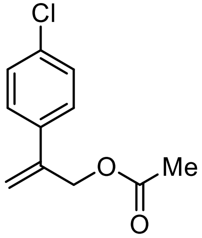

2j

^{1}H NMR (400 MHz, 298 K, $CDCl_3$)

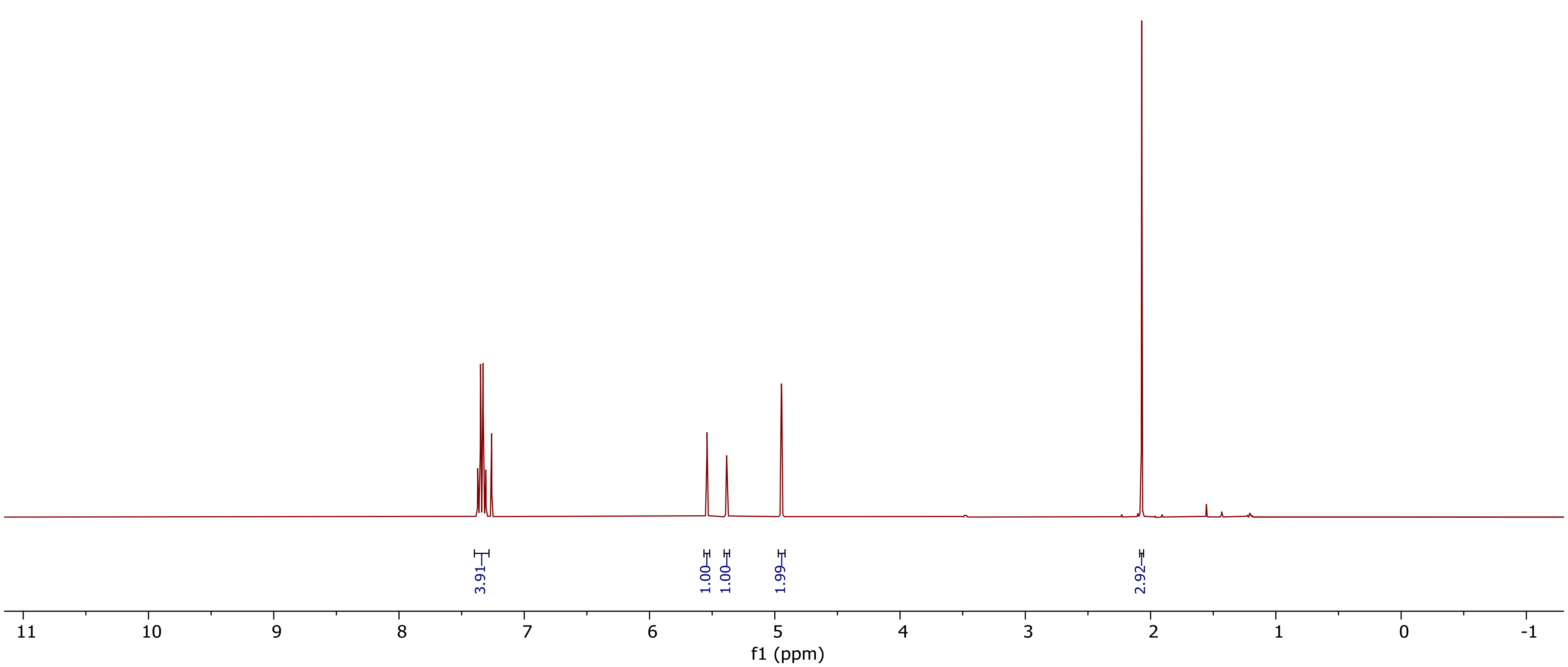

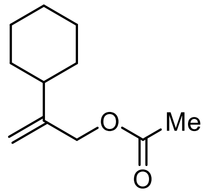

2k

^{1}H NMR (400 MHz, 298 K, $CDCl_3$)

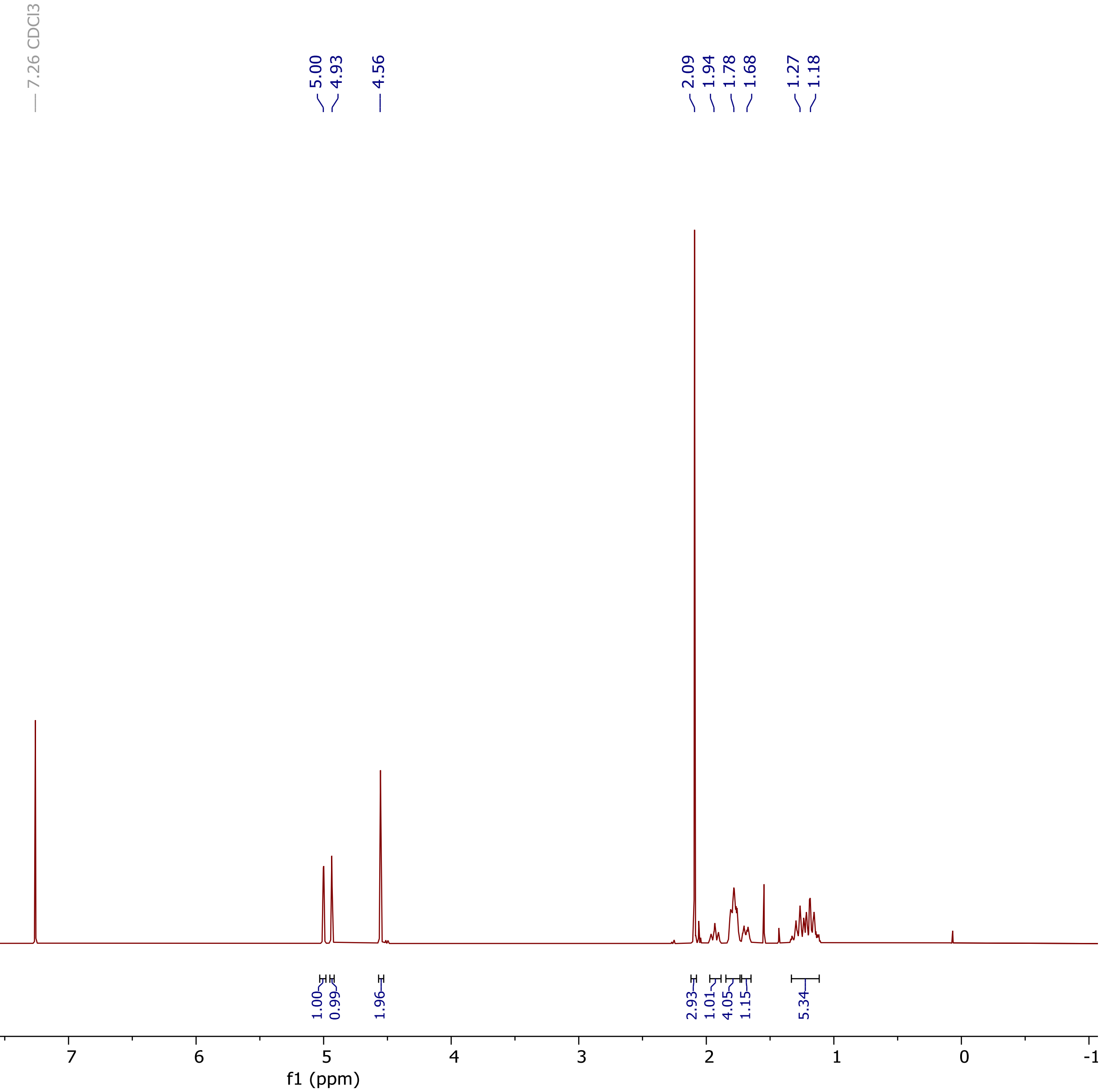

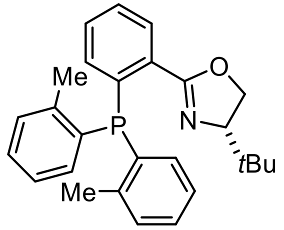

L_{11}
^{1}H NMR (400 MHz, 298 K, $CDCl_3$)

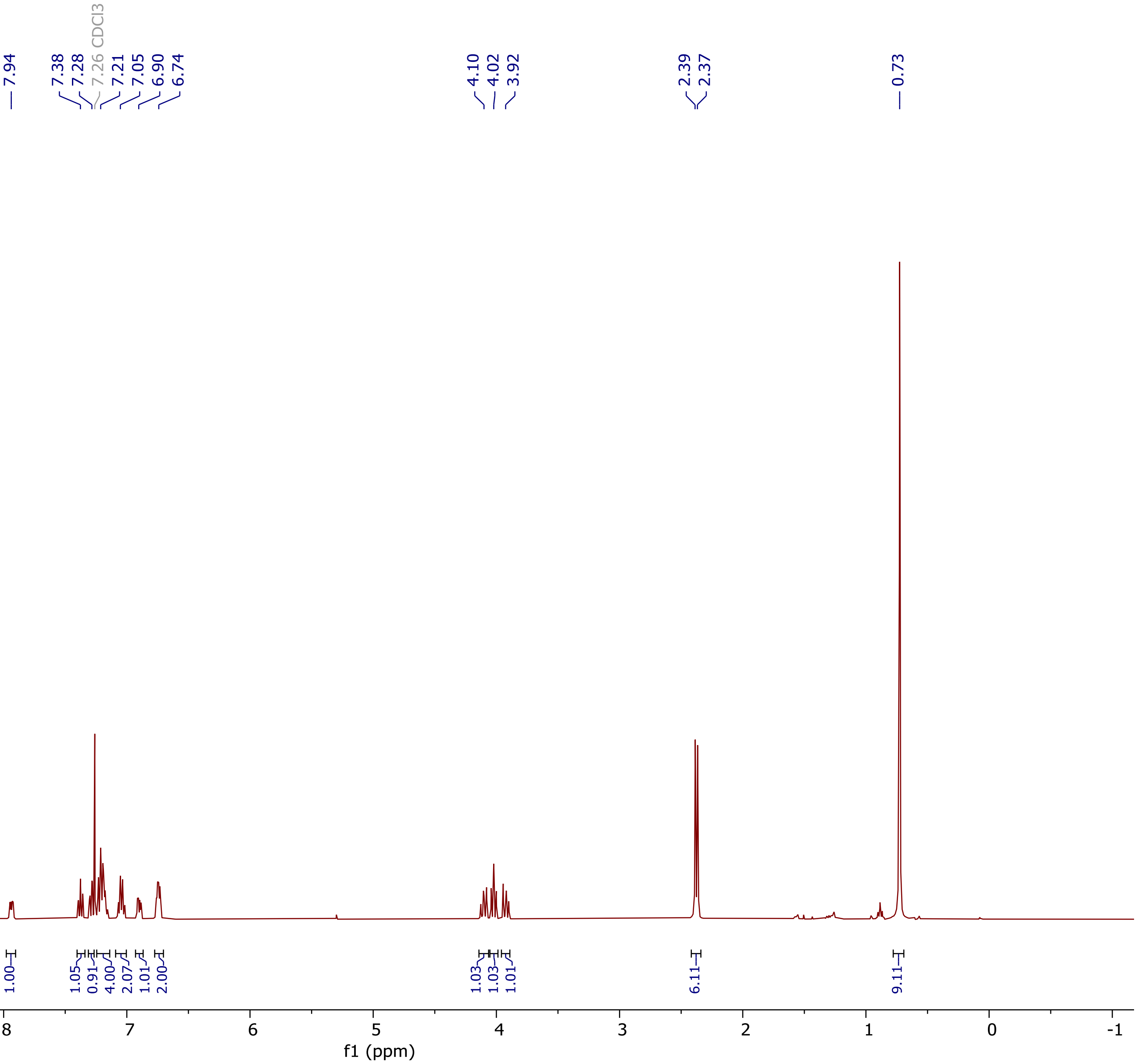

all_geoms_centered.zip
This file cannot be rendered in this PDF. Please download the source file.
